# Changes in health in England, with analysis by English regions and areas of deprivation, 1990–2013: a systematic analysis for the Global Burden of Disease Study 2013

**DOI:** 10.1016/S0140-6736(15)00195-6

**Published:** 2015-12-05

**Authors:** John N Newton, Adam D M Briggs, Christopher J L Murray, Daniel Dicker, Kyle J Foreman, Haidong Wang, Mohsen Naghavi, Mohammad H Forouzanfar, Summer Lockett Ohno, Ryan M Barber, Theo Vos, Jeffrey D Stanaway, Jürgen C Schmidt, Andrew J Hughes, Derek F J Fay, Russell Ecob, Charis Gresser, Martin McKee, Harry Rutter, Ibrahim Abubakar, Raghib Ali, H Ross Anderson, Amitava Banerjee, Derrick A Bennett, Eduardo Bernabé, Kamaldeep S Bhui, Stanley M Biryukov, Rupert R Bourne, Carol E G Brayne, Nigel G Bruce, Traolach S Brugha, Michael Burch, Simon Capewell, Daniel Casey, Rajiv Chowdhury, Matthew M Coates, Cyrus Cooper, Julia A Critchley, Paul I Dargan, Mukesh K Dherani, Paul Elliott, Majid Ezzati, Kevin A Fenton, Maya S Fraser, Thomas Fürst, Felix Greaves, Mark A Green, David J Gunnell, Bernadette M Hannigan, Roderick J Hay, Simon I Hay, Harry Hemingway, Heidi J Larson, Katharine J Looker, Raimundas Lunevicius, Ronan A Lyons, Wagner Marcenes, Amanda J Mason-Jones, Fiona E Matthews, Henrik Moller, Michele E Murdoch, Charles R Newton, Neil Pearce, Frédéric B Piel, Daniel Pope, Kazem Rahimi, Alina Rodriguez, Peter Scarborough, Austin E Schumacher, Ivy Shiue, Liam Smeeth, Alison Tedstone, Jonathan Valabhji, Hywel C Williams, Charles D A Wolfe, Anthony D Woolf, Adrian C J Davis

**Affiliations:** a Public Health England, London, UK; b London School of Economics, London, UK; c Institute for Health Metrics and Evaluation, Seattle, WA, USA; d University of Manchester, Manchester, UK; e Centre for Infectious Disease Epidemiology and MRC Clinical Trials Unit, London, UK; f INDOX Cancer Research Network, Oxford, UK; g John Radcliffe Hospital, Oxford, UK; h Green-Templeton College, University of Oxford, Oxford, UK; i Population Health Research Institute, Hamilton, ON, Canada; j MRC-PHE Centre for Environment and Health, London, UK; k St George's, University of London, London, UK; l University of Birmingham, Birmingham, UK; m Clinical Trials Service Unit and Epidemiological Studies Unit, Nuffield Department of Population Health, University of Oxford, Oxford, UK; n King's College London Dental Institute, London, UK; o Wolfson Institute of Preventive Medicine, Barts & The London School of Medicine, Queen Mary University of London, London, UK; p Vision & Eye Research Unit, Anglia Ruskin University, Cambridge, UK; q Cambridge Institute of Public Health, University of Cambridge, Cambridge, UK; r University of Oxford, Oxford, UK; s University of Liverpool, Liverpool, UK; t University of Leicester, Leicester, UK; u Great Ormond Street Hospital for Children, London, UK; v University of Cambridge, Cambridge, UK; w MRC Lifecourse Epidemiology Unit, University of Southampton, Southhampton, UK; x Guy's and St Thomas' NHS Foundation Trust, London, UK; y University College London, London, UK; z Department of Epidemiology and Biostatistics, MRC-PHE Centre for Environment and Health, Imperial College London, London, UK; aa MRC-PHE Centre for Population Health, School of Public Health, Imperial College London, London, UK; ab Department of Infectious Disease Epidemiology, Imperial College London, London, UK; ac Department of Primary Care and Public Health, Imperial College London, London, UK; ad School of Health and Related Research (ScHARR), University of Sheffield, Sheffield, UK; ae School of Social and Community Medicine, University of Bristol, Bristol, UK; af Ulster University, Coleraine, Northern Ireland; ag International Foundation for Dermatology, London, UK; ah Wellcome Trust Centre for Human Genetics, University of Oxford, Oxford, UK; ai Farr Institute of Health Informatics Research, London, UK; aj Department of Infectious Disease Epidemiology, London School of Hygiene & Tropical Medicine, London, UK; ak Department of Health Services Research and Policy, London School of Hygiene & Tropical Medicine, London, UK; al School of Social and Community Medicine, University of Bristol, Bristol, UK; am Aintree University Hospital NHS Foundation Trust, University of Liverpool, Liverpool, UK; an Queen Mary University of London; ao Department of Health Sciences, University of York, York, UK; ap Adolescent Health Research Unit, University of Cape Town, Cape Town, South Africa; aq Institute of Health and Society, Newcastle University, Newcastle, UK; ar Cancer Epidemiology and Population Health, King's College London, London, UK; as West Hertfordshire Hospitals NHS Trust, Hertfordshire, UK; at London School of Hygiene & Tropical Medicine, Oxford Martin School, University of Oxford, Oxford, UK; au George Institute for Global Health and Division of Cardiovascular Medicine, Oxford Martin School, University of Oxford, Oxford, UK; av NHS England, Leeds, UK; aw Imperial College Healthcare NHS Trust, London, UK; ax Imperial College London, London, UK; ay University of Edinburgh, Edinburgh, Scotland; az University of Nottingham, Nottingham, UK; ba King's College London, London, UK; bb Royal Cornwall Hospital, Treliske, UK; bc Mid Sweden University, Sundsvall, Sweden; bd British Heart Foundation Centre on Population Approaches for NCD Prevention, University of Oxford, Oxford, UK; be Northumbria University, Newcastle upon Tyne; bf Farr Institute, College of Medicine, Swansea University, Swansea, UK

## Abstract

**Background:**

In the Global Burden of Disease Study 2013 (GBD 2013), knowledge about health and its determinants has been integrated into a comparable framework to inform health policy. Outputs of this analysis are relevant to current policy questions in England and elsewhere, particularly on health inequalities. We use GBD 2013 data on mortality and causes of death, and disease and injury incidence and prevalence to analyse the burden of disease and injury in England as a whole, in English regions, and within each English region by deprivation quintile. We also assess disease and injury burden in England attributable to potentially preventable risk factors. England and the English regions are compared with the remaining constituent countries of the UK and with comparable countries in the European Union (EU) and beyond.

**Methods:**

We extracted data from the GBD 2013 to compare mortality, causes of death, years of life lost (YLLs), years lived with a disability (YLDs), and disability-adjusted life-years (DALYs) in England, the UK, and 18 other countries (the first 15 EU members [apart from the UK] and Australia, Canada, Norway, and the USA [EU15+]). We extended elements of the analysis to English regions, and subregional areas defined by deprivation quintile (deprivation areas). We used data split by the nine English regions (corresponding to the European boundaries of the Nomenclature for Territorial Statistics level 1 [NUTS 1] regions), and by quintile groups within each English region according to deprivation, thereby making 45 regional deprivation areas. Deprivation quintiles were defined by area of residence ranked at national level by Index of Multiple Deprivation score, 2010. Burden due to various risk factors is described for England using new GBD methodology to estimate independent and overlapping attributable risk for five tiers of behavioural, metabolic, and environmental risk factors. We present results for 306 causes and 2337 sequelae, and 79 risks or risk clusters.

**Findings:**

Between 1990 and 2013, life expectancy from birth in England increased by 5·4 years (95% uncertainty interval 5·0–5·8) from 75·9 years (75·9–76·0) to 81·3 years (80·9–81·7); gains were greater for men than for women. Rates of age-standardised YLLs reduced by 41·1% (38·3–43·6), whereas DALYs were reduced by 23·8% (20·9–27·1), and YLDs by 1·4% (0·1–2·8). For these measures, England ranked better than the UK and the EU15+ means. Between 1990 and 2013, the range in life expectancy among 45 regional deprivation areas remained 8·2 years for men and decreased from 7·2 years in 1990 to 6·9 years in 2013 for women. In 2013, the leading cause of YLLs was ischaemic heart disease, and the leading cause of DALYs was low back and neck pain. Known risk factors accounted for 39·6% (37·7–41·7) of DALYs; leading behavioural risk factors were suboptimal diet (10·8% [9·1–12·7]) and tobacco (10·7% [9·4–12·0]).

**Interpretation:**

Health in England is improving although substantial opportunities exist for further reductions in the burden of preventable disease. The gap in mortality rates between men and women has reduced, but marked health inequalities between the least deprived and most deprived areas remain. Declines in mortality have not been matched by similar declines in morbidity, resulting in people living longer with diseases. Health policies must therefore address the causes of ill health as well as those of premature mortality. Systematic action locally and nationally is needed to reduce risk exposures, support healthy behaviours, alleviate the severity of chronic disabling disorders, and mitigate the effects of socioeconomic deprivation.

**Funding:**

Bill & Melinda Gates Foundation and Public Health England.

**Research in context**

**Evidence before this study**

Responsibility for health and public health has been devolved to the constituent countries of the UK since 1998, but no single accessible source describing disease burden by cause in England exists. A published estimate of burden of disease for the UK, using GBD 2010 data and methodology, has been widely used by policy makers but has limitations and needs updating. Routine mortality statistics show progressive improvements in life expectancy, but inequalities persist between countries in the UK and regions within England. Routine measures of morbidity are not reported in a way that allow direct comparisons of burden between causes at a national or regional level. Attributable risks have been calculated for some causes (eg, smoking and air pollution) and for some disorders (eg, cancers) but are not available in a comprehensive framework covering multiple risks and diseases. Relevant literature reviews were undertaken to inform components of the GBD analysis in particular on the relation between risk factors and outcomes.

**Added value of this study**

The GBD 2013 analysis of causes of death, disease, and injury incidence and prevalence, YLDs, and risk factors is a major update and improvement in the evidence base underlying the quantification of comparable disease and risk factor estimates compared with the most recent GBD analyses in 2010. For the first time, GBD results for England are quantified at the national and subnational level (at the level of nine English regions) and by IMD-2010 deprivation quintile using a range of routine and published data sources.

**Implications**

Quantification of the continuing burden of preventable ill health in England more than justifies recent calls for a “radical upgrade in prevention and public health”. A huge opportunity exists for preventive public health: if levels of health in the worst performing regions in England matched the best performing ones, England would have one of the lowest burdens of disease of any advanced industrialised country. The scale of the increasing level of disability suggests the need for new, more integrated models of care spanning health and social services that respond to the specific needs and circumstances of individual patients. Inequalities in health are greater within English regions than between them and are therefore largely related to deprivation, not geography. The causes of inequalities need to be addressed wherever they occur. The importance of prevention argues for investment in robust, standardised comparative assessments of the cost-effectiveness of different public health policies to aid decision making, akin to those used in England for health technologies.

## Introduction

The Global Burden of Disease (GBD) project is a worldwide exercise to integrate the widest possible range of reliable knowledge about health and its determinants into a universal health framework. Outputs allow meaningful comparisons between countries and over time, and quantify the contributions of different diseases and risk factors to overall burden.

Investigators of a previous GBD study^1^ used data from 2010 to compare the UK with a group of other countries. The new analysis reported here uses data from 1990 through 2013 and focuses on England. Responsibility for health and public health has been devolved to the constituent countries of the UK (England, Scotland, Wales, and Northern Ireland) since 1998,^2^ so this geographical level of analysis is most meaningful. We compare England and the English regions with the remaining constituent countries of the UK, and with comparable countries in the European Union (EU) and beyond. This study comes at a time when both national and local health-policy makers are reviewing and reassessing priorities for action in the light of financial pressures. The potential for prevention and public health to secure improved population health and reduce demand on England's National Health Service (NHS) has also received further attention recently.^3^

The Global Burden of Diseases, Injuries, and Risk Factors Study 2013 (GBD 2013) provides both improvements in data and advances in methodology compared with previous versions of the GBD.^4^, ^5^, ^6^, ^7^ These advances include a greatly improved approach to identification of the contribution of various risk factors and combinations of risks, as explained in the Methods and reported in detail elsewhere,^6^ as well as updates to many data sources.

The subnational analysis reported here, based on English regions and areas within them that have differing levels of deprivation, makes it possible to map disease burden according to socioeconomic determinants of health. This report therefore not only uses an improved approach but also extends the scope of earlier work and increases its relevance to policy makers in several ways.

## Methods

### Overview

Here we use data from the GBD 2013 study of causes of death, disease, and injury incidence and prevalence as well as years lived with disability (YLDs) to analyse the burden of diseases and injuries in England by English region and, within each English region by deprivation quintile (defined nationally). The methods employed in the GBD 2013, including the systematic approach to collating cause of death from different countries, the mapping across different revisions and national variants of the International Classification of Diseases and Injuries (ICD), redistribution of deaths assigned to so-called garbage codes, and the cause of death modelling approach used for each cause, have been described in detail elsewhere.^4^ The GBD 2013 Collaborators^5^ describe the data and methods used to estimate incidence, prevalence, and YLDs for 306 causes and 2337 sequelae from 1990 to 2013, a substantial increase from 220 causes and 1160 sequelae in the GBD 2010 analysis.^1^ This GBD 2013 paper includes a description of the systematic reviews of the published literature, identification of unpublished data sources, efforts to map data to a consistent set of case definitions, and the general approach to Bayesian meta-regression using DisMod-MR 2.0, which allows the estimation of incidence, prevalence, remission, excess mortality, and cause-specific mortality rates that are internally consistent. Details of the method, the likelihood used in estimation, and the source code have been published elsewhere.^8^, ^9^ The analysis of risk factor-attributable burden uses the GBD 2013 framework and results.^6^ Sampling and non-sampling error as well as model uncertainty is propagated by estimating all steps in the calculations 1000 times. 95% uncertainty intervals (UIs) are presented by the 2·5 and 97·5 centile values.

Another new feature of the GBD 2013 study is the systematic aggregation of the burden attributed to five tiers of risk factors: the first tier is all GBD risks combined; the second tier consists of three large categories of metabolic, behavioural, and environmental and occupational risks; the third tier contains single risks, such as high blood pressure, and risk clusters, such as child and maternal under-nutrition or air pollution; the fourth tier includes single risks within such clusters, such as vitamin A deficiency or household air pollution; and the fifth tier is for individual occupational carcinogens or the division of childhood underweight into stunting, underweight, and wasting. At each level of the hierarchy, a decision is made whether the combined effects are independent and can be added, whether they are joint effects best represented by multiplication, or whether they share common pathways for which mediation needs to be taken into account. For each aggregation, the proportion of the effect shared with another risk or combination of risks can thus be made explicit, using modified Venn diagrams that show the overlaps between metabolic, behavioural, and environmental and occupational risks.

Here we focus on specific issues related to the analysis of causes of death, injury incidence and prevalence, and risk factor prevalence in England, the nine English regions, and 45 subregional areas defined by deprivation quintile (deprivation areas).

### Division of England into English regions and deprivation areas

Estimates of disease burden have been created for the nine English regions, as defined by the former government office regions in England, and correspond to the European boundaries of the Nomenclature for Territorial Statistics level 1 (NUTS 1) regions. In a further refinement, all English lower super output areas, relatively homogeneous areas containing about 1600 people on average,^10^ were ranked nationally using the Index of Multiple Deprivation (IMD-2010) and allocated to quintiles. The IMD-2010 is a composite measure estimated at a small geographical area and includes seven domains: income, employment, health and disability, education, skills and training, barriers to housing and services, living environment, and crime.^11^ The health and disability domain of the IMD-2010 contributes 13·5% to the score and encompasses four measures: years of potential life lost, comparative illness and disability ratio, rate of emergency admissions to hospital, and proportion of adults younger than 60 years who have mood or anxiety disorders. Although inevitably partially correlated with health, exclusion of the health component from an earlier version of the IMD has been shown to make little difference to ranking of areas by deprivation in practice.^12^ The lower super output areas in each quintile were then reallocated to their region, thereby dividing each of the nine English regions into five deprivation groups, or 45 regional deprivation areas in total. As the lower super output areas at each level of deprivation are unevenly distributed among the English regions, there will be a greater share of the regional population living in the most deprived and least deprived (nationally) areas in each English region. Thus, the proportion of the population in the most deprived group ranges from 7·3% in South East England to 32·8% in North West England. For the least deprived group, this proportion ranges from 7·9% in Greater London to 34·8% in South East England. The complete breakdown of these proportions is provided in the appendix (p 4). Within each English region, the most deprived area is referred to as deprivation level 1, and the least deprived area as deprivation level 5.

Mortality data for England from 1990 to 2012, available from the Office for National Statistics, were split into regional and deprivation groups based on the postcode of residence. Where a postcode for the deceased had not been provided, these deaths were discarded from the analysis of England mortality because no residence in England was assumed. These records make up less than 0·3% of all mortality records. Each death was assigned to a lower super output area, deprivation group, and English region on the basis of the person's postcode. GBD estimation of disease prevalence and incidence also makes use of social, cultural, economic, and environmental covariates; some covariate and morbidity data were available at the level of English region. The source data for England used at each level are provided in the appendix p 5; regional level covariates that have been included are listed in the appendix p 44.

### Analysis of cause of death by English region and deprivation area

As outlined in the GBD 2013 report about global mortality and cause of death,^4^ vital registration data covering the years 1980 to 2012 were analysed at the regional level. Registration of deaths that happen in England is a legal requirement, and because registration is necessary before disposal of the body, mortality data are assumed to be complete.^1^, ^13^, ^14^ We reclassified causes of death for deaths assigned to causes that cannot or should not be an underlying cause of death, so-called garbage codes.^15^, ^16^ Standard GBD 2013 redistribution of garbage-code algorithms was applied.^17^ Although data were available for all years between 1980 and 2012, to deal with stochastic variation at the regional level, while following the GBD 2013 methods, we modelled causes of death using cause of death ensemble modelling (CODEm).^4^, ^18^ CODEm has been used extensively to model causes of death; an ensemble model is developed by testing the performance of a wide array of models (mixed effects or space–time Gaussian process regression), different measures of mortality (rates or cause fractions) and varying combinations of covariates (drawing on a database created for GBD of more than 200 diverse characteristics for countries over time, such as gross domestic product, level of education, dietary factors, use of health-service, and environmental statistics), and by selecting the models with best out-of-sample performance. For example, GBD suicide estimates for the UK are lower than those produced by the Office for National Statistics by almost a quarter. The Office for National Statistics estimates include all deaths coded as suicide (ICD-10 X60–X84) and deaths coded as due to injury and poisoning of undetermined intent (ICD Y10–34),^19^ whereas GBD uses a redistribution approach, coding only a proportion of undetermined intent deaths as suicide.

The GBD places disease categories within a four-level cause hierarchy. The first level divides causes into communicable diseases, non-communicable diseases, and injuries; the second level consists of major disease or injury groups, such as cardiovascular diseases or transport injuries; the third level (at which most results are reported) further subdivides causes into disease or injury types, such as cerebrovascular disease or road injuries; and a final fourth level subdivides those disease types where appropriate. Further details can be found in the supplementary appendix of the GBD 2013 Mortality and Cause of Death report.^4^

Tabulations of deaths by cause were generated by deprivation area within each English region. Where the causes of deaths were identified as garbage codes, these were reclassified using the GBD 2013 algorithms. Owing to small sample size in some age–sex–cause groups, we sought to smooth stochastic variation over time. To estimate causes of death by age, sex, and year for a deprivation area within each English region, we first computed the fraction of deaths for a cause–sex–age–year in each deprivation area. To minimise the effect of stochastic fluctuations on the results, we used a 3-year moving average for age groups over 15 years, and a 5-year moving average for age groups under 15 years. We chose a longer time period for the moving average for childhood age groups because these data are most prone to fluctuations due to small numbers of annual deaths. Moving-average deprivation-area fractions within a cause–age–sex–year group were rescaled so that the sum of cause fractions equalled 100%. These deprivation-area fractions were multiplied by the regional level final estimates of death counts for an age–sex–year group for a given cause to generate estimates of final death counts for each deprivation area. Death counts were divided by deprivation area population to generate deprivation-level cause–age–sex–year rates.

### Disease and injury incidence, prevalence, and YLDs by English region and deprivation area

A list of sources used for the analysis of non-fatal health outcomes in England organised by disease is provided in the appendix (p 5). These sources include studies extracted from the published literature through the GBD systematic reviews as well as extractions from surveys, such as the Health Survey for England,^20^ and administrative sources, such as NHS hospital discharge data. We also used new data from the UK-based Cognitive Function and Ageing Studies.^21^ Most disease sequelae have been modelled in GBD 2013 using a Bayesian meta-regression method, DisMod-MR 2.0, in which each English region has been analysed as a distinct geographic unit. A prior for the Bayesian meta-regression is calculated for each English region using the data for all countries in western Europe, with random effects on countries and English regions and fixed effects that vary by the disease being modelled.

To analyse injuries, we made use of both the external cause of injury and the nature of injury. As detailed elsewhere,^5^ we used survey and hospital activity data to estimate incidence of injuries for which hospital admission was necessary and injuries for which hospital admission was not necessary. Hospital data dual-coded to nature of injury and external cause of injury were used to estimate the fraction of each injury with different types of disabling sequelae. Cohort studies from four countries were used to estimate the probability of long-term disability for each type of injury.^6^, ^22^, ^23^ DisMod-MR 2.0 was used to estimate the prevalence of injury in each birth cohort on the basis of long-term disability arising from past incidence. Given the absence of data on injury incidence before 1980, we assumed that age-specific incidence in cohorts before 1980 was equal to the rate in 1980.

Following the GBD 2013 methods, prevalence of individuals in each sequela was multiplied by the disability weight for the corresponding health state to calculate YLDs for the particular sequela. The sum of all the YLDs for relevant sequelae is the overall YLD for each disease. We based disability weights on the responses by the general public to questions about which health state of randomly chosen pairs represents a higher state of health. GBD 2013 disability weights were based on the pooled analysis of 60 890 responses from household surveys done in a wide range of settings (USA, Peru, Tanzania, Bangladesh, Indonesia, Italy, Hungary, Sweden, and the Netherlands, and an open access internet survey) to allow them to be generalised to the global population.^22^ We analysed the disability weight surveys to generate 235 health state weights on a scale of 0·0 (perfect health) to 1·0 (like death). Each of the 2337 sequelae in the study are mapped to a particular health state and its associated disability weight. Results showed little variation by country of survey or level of education of respondents, justifying the use of a single set of disability weights for all countries and time periods.

YLDs for deprivation areas have been estimated from regional level results. For causes where substantial mortality exists, we have assumed that the pattern of disease prevalence mirrors the pattern of mortality in an age–sex group. For causes where there is minimal mortality and no available data, we have assumed that YLD rates for an age–sex group are constant across deprivation levels in an English region. The threshold used to define minimal mortality is if the ratio of years of life lost (YLLs) to YLDs was less than 0·15.

### Age standardisation, multiple-decrement life tables, and benchmarking years of life lost

The GBD 2013 global age-standard population was used to compute age-standardised rates.^4^

We used multiple-decrement life tables to compute the contribution of changes in cause-specific mortality to changes in life expectancy for each English region and deprivation area from 1990 to 2013.^24^, ^25^

We computed YLLs by multiplying numbers of deaths from each cause in each age group by the reference life expectancy at the average age of death for those who die in the age group, as in the GBD 2010.^9^, ^13^, ^16^, ^26^, ^27^ The reference life expectancy at birth is 86·02 years and is based on the lowest age-specific death rates observed in all countries with populations greater than 5 million in 2010. We compared English regions and England as a whole with 18 other comparator nations (the first 15 members of the EU [apart from the UK], Australia, Canada, Norway, and the USA; EU15+). This set of countries has been used in previous benchmarking analyses for the UK.^1^

To describe the proportional share of the variation for deprivation group and English region, ANOVA was carried out at regional level and, for each deprivation area, within English regions. We did the analysis separately for each outcome measure (death, YLLs, YLDs, disability-adjusted life years [DALYs], life expectancy), and for specific years (1990, 1995, 2000, 2005, 2010, 2013). Models with main effects for region and deprivation, and of the interaction of English region and deprivation, were fitted to the data. Decomposition of variance was estimated using mixed-effects linear regression employing Gelman's methods.^28^ UIs for the decomposition of variance were based on two steps. First, we took 1000 draws from the standard error of the random effect across English regions and the random effect across deprivation levels. Second, we computed the fraction of variance explained by English region and deprivation level based on each of these draws. Table 1 shows the proportion of the variance due to deprivation and due to English region (with 95% UIs).

### Role of the funding source

The GBD 2013 database development, methods improvement, and global analysis is primarily funded by the Bill & Melinda Gates Foundation, which had no role in study design, data collection, data analysis, data interpretation, or writing of the report. Public Health England contributed to the interpretation of data, the writing of the report, and the decision to submit the paper for publication. The corresponding author had full access to all the data in the study and had final responsibility to submit the paper.

## Results

Between 1990 and 2013, life expectancy from birth in England increased by 5·4 years (95% UI 5·0–5·8) from 75·9 years (75·9–76·0) to 81·3 years (80·9–81·7), and age-standardised death rates were reduced by 33·6% (31·1–36·1; appendix p 21). During the same time period, the relative reduction in the rate of age-standardised YLLs was 41·1% (38·3–43·6), indicating a proportionately larger reduction in premature mortality when compared with overall mortality. In comparison, age-standardised rates of YLDs, which capture the burden of disability, decreased by only 1·4% (0·1–2·8). With DALYs, which combine mortality and disability, there was an overall reduction of 23·8% (20·9–27·1) between 1990 and 2013. Compared with the EU15+ countries, in 2013, England ranked eighth for age-standardised death rates, seventh for YLLs, seventh for YLDs, and sixth for DALYs; better than the UK as a whole on all measures. Corresponding results for the constituent countries of the UK and the English regions are provided in the appendix (p 21).

Figure appendix 1 shows the change in life expectancy from 1990 to 2013 by broad cause group for men and women separately and for both sexes combined in England, the English regions, Scotland, Northern Ireland, Wales, and the EU15+ countries. Between 1990 and 2013, England overall achieved one of the largest gains in life expectancy among men, with 6·4 gained years, less than Luxembourg but tying with Finland. All English regions except for South West England, achieved a gain of at least 6·0 years, equal to or greater than all comparator countries except Austria, Finland, Ireland, Germany, and Luxembourg. Among women, the increase in life expectancy in England overall was more modest than for men, with 4·4 years, yet still equalled or exceeded that of all countries except Finland, Germany, Ireland, Luxembourg, and Portugal.

In 1990, the life expectancy of men in England was lower than in many western countries, such as Canada, France, Norway, the Netherlands, and Spain; however, by 2013, life expectancy of men in England had surpassed that of each of these countries, reaching 79·5 years (95% UI 78·9–80·0). In three regions of England, South West England, East of England, and South East England, the life expectancy of men was 80 years or above, which is better than that in Australia and in Sweden. The main drivers for improvement in life expectancy in nearly all countries and in the English regions have been declines in cardiovascular disease and, to a lesser extent, cancer mortality. Decreases in chronic respiratory disease and road injuries have also been important contributors. Increased mortality from cirrhosis of the liver and from mental and substance use disorders (mostly attributed to alcohol use) made negative contributions to life expectancy in all the English regions except for Greater London and South East England.

For women, life expectancy at birth across countries and the English regions in 2013 ranged from 81·0 years (95% UI 80·3–81·7) in Scotland to 84·9 years (84·4–85·4) in France. Geographical patterns, however, are somewhat different from those for men, with the highest life expectancy for women in Spain, Italy, and France. Australia and South East England reached a female life expectancy of 84·0 years (83·5–84·5 and 83·3–84·6, respectively) in 2013, whereas two English regions and four countries have a female life expectancy at or below 82 years, namely: North East England, North West England, Scotland, USA, Northern Ireland, and Denmark. As with men, the key drivers of increases in female life expectancy have been reductions in cardiovascular diseases and cancers. For most countries, sex differences in life expectancy decreased between 1990 and 2013; in England, the gap closed by 2·2 years.

Figure appendix 2 shows a similar analysis of changes in life expectancy from 1990 to 2013 by cause for the 45 deprivation areas of England. For men, life expectancy in 2013 ranged from 74·9 years (95% UI 74·1–75·7) in North West England deprivation level 1 (most deprived) to 83·1 years (82·3–83·9) in East of England deprivation level 5 (least deprived; figure appendix 2). The range of 8·2 years between deprivation areas in 2013 is unchanged for men since 1990. For women, the equivalent figures in 2013 were 79·5 years (78·7–80·2) in North West England deprivation level 1 (most deprived) to 86·4 years (85·6–87·3) in East of England deprivation level 5 (least deprived; figure appendix 2); the range has therefore decreased for women from 7·2 years to 6·9 years. Overall, there has been little if any improvement in inequality in life expectancy across regions of England: by 2013, people living in the most deprived areas have not yet reached the levels of life expectancy that less deprived groups had in 1990.

The ordering of the 45 deprivation areas largely follows deprivation level across English regions. There are a few crossovers such as Greater London deprivation level 1, which has better life expectancy than North West England deprivation level 2 (figure appendix 2). Table 1 presents the results of the ANOVA analysis by year for various measures in this group. For men in 2013, at least 85% of variation was associated with IMD-2010 score, and less than 10% was associated with English region for all measures, whereas for women the values explained by level of deprivation of the area were slightly lower, especially for mortality.

Change across all deprivation areas was dominated by decreases in cardiovascular diseases and cancers. Increased death rates from cirrhosis of the liver, mental and substance use disorders, and neurological diseases, which were largest in the most deprived areas, reduced the progress that would otherwise have been achieved from reductions in other causes.

**Figure 1:**
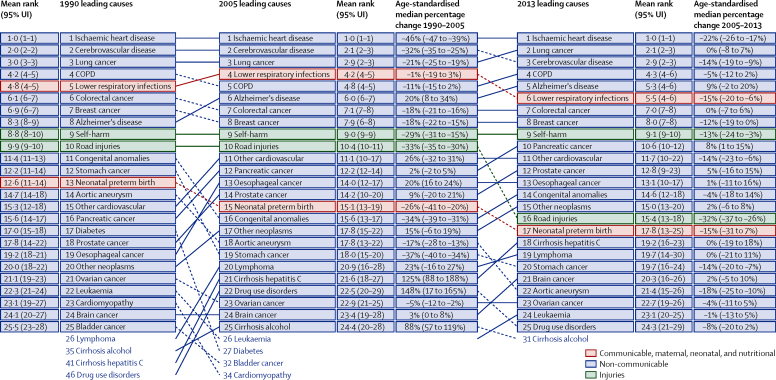
The 25 leading GBD level 3 causes of years of life lost (YLLs) in England, both sexes combined, 1990, 2005, and 2013, with age-standardised median percent change Ranks are based on the number of YLLs. 95% UIs for mean rank are from 1000 draws of YLLs. UI=uncertainty interval. COPD=chronic obstructive pulmonary disease.

Figure 1 shows the leading causes of YLLs using broad disease categories (level 3 in the GBD cause hierarchy). The leading causes of death overall are shown in the appendix (p 1). Ranking is based on the number of YLLs from each cause, which is a function of age-specific rates and the distribution of the population by age and sex in 1990 and 2013. Changes in the number of YLLs and the age-standardised YLL rate are shown to highlight the effect of demographic change on numbers of YLLs (eg, YLLs due to Alzheimer's disease and other dementias have increased by 31% despite a 3% decline in the age-standardised rate). The top four causes of death in 1990 (ischaemic heart disease, cerebrovascular disease, lung cancer, and chronic obstructive pulmonary disease [COPD]) remain at the top in 2013, despite substantial declines in their age-standardised rates. Lung cancer and cerebrovascular disease switched ranks because of the large change in cerebrovascular disease age-standardised rates (−55%, 95% UI −59 to −50) between 1990 and 2013. Alzheimer's disease and other dementias increased in rank (from eighth to fifth) even though there was a statistically non-significant decline in age-standardised rates; this needs to be considered in view of an increase in the recording of Alzheimer's disease on death certificates.^29^ Lower respiratory infections, colorectal cancer, breast cancer, and self-harm all remained highly ranked but still showed reductions in age-standardised YLL rates of more than 34% and a reduction in numbers of YLLs. Large reductions in both numbers and rates of YLLs were also observed for preterm birth, road injuries, aortic aneurysm, and diabetes. By contrast, as for Alzheimer's disease and other dementias, YLL numbers for several other cancer types increased even though age-standardised rates declined slightly. There were increases in both numbers and rates for only a small number of causes, notably cirrhosis of the liver due to hepatitis C and drug use disorders.

**Figure 2:**
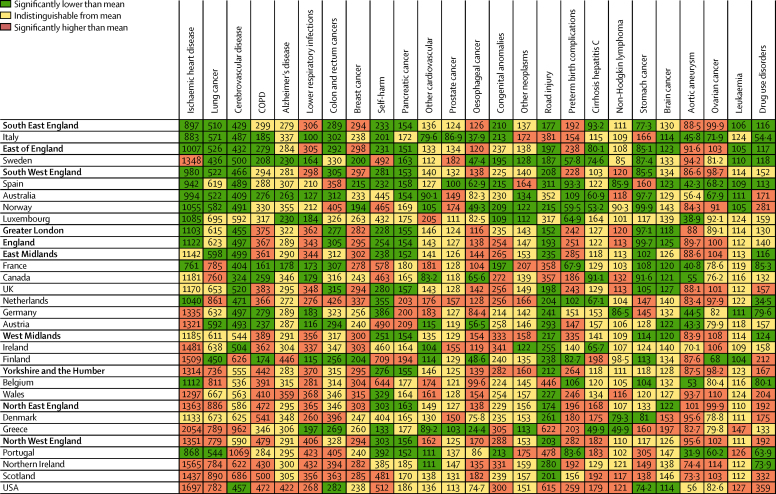
Age-standardised rates of years of life lost (YLLs) for England and the nine English regions relative to EU15 countries, Australia, Canada, Norway, the USA, Scotland, Northern Ireland, and Wales for both sexes combined in 2013 EU15+ countries, UK constituent countries, and English regions are ordered by the overall mean age-standardised YLL rate. To facilitate comparison, England and the nine English regions are shown in bold. For illustrative purposes only, UK constituent countries and the English regions have been included where they would rank if they were one of the EU15+ countries; this is not to suggest that the health system in a given English region is equivalent to that of any of the countries to which it is adjacent in the list. Rates are colour-coded to denote statistically significant differences from the mean across this set of English regions and countries. Lung cancer=lung, bronchus, and trachea cancer. COPD=chronic obstructive pulmonary disease. Alzheimer's disease=Alzheimer's disease and other dementias. Other cardiovascular=other cardiovascular and circulatory diseases. Cirrhosis hepatitis C=cirrhosis of the liver secondary to hepatitis C. Brain cancer=brain and central nervous system cancer.

Figure 2 shows age-standardised YLL rates for both sexes from GBD level 3 causes in England and its nine regions relative to the EU15+ countries. Across the EU15+ countries, the absolute and relative ranges in age-standardised rates for different causes are markedly different. Within the EU15+ countries, the ratio of maximum rate to minimum rate is less than 2·0 for eight of the top 25 causes: leukaemia, ovarian cancer, brain cancer, other neoplasms, pancreatic cancer, breast cancer, colorectal cancer, and lung cancer. Ratios of maximum-to-minimum rate greater than 4·0 are seen for drug use disorders (10·4), oesophageal cancer (5·8), lower respiratory infections (4·2), neonatal preterm birth (4·5), self-harm (5·3), and stomach cancer (4·1). Across English regions, the only cause with a ratio of maximum-to-minimum rate greater than 2·0 is cirrhosis of the liver due to hepatitis C (2·3). Eight other disorders have a ratio between 1·5 and 2·0: ischaemic heart disease, lung cancer, COPD, congenital abnormalities, road injuries, neonatal preterm birth, stomach cancer, and drug use disorders. For some causes, such as lower respiratory infections and breast cancer, all English regions are significantly above the international mean, indicating that England is performing poorly for these disorders. For self-harm and road injuries, the opposite is true with all English regions being significantly below the mean.

**Figure 3:**
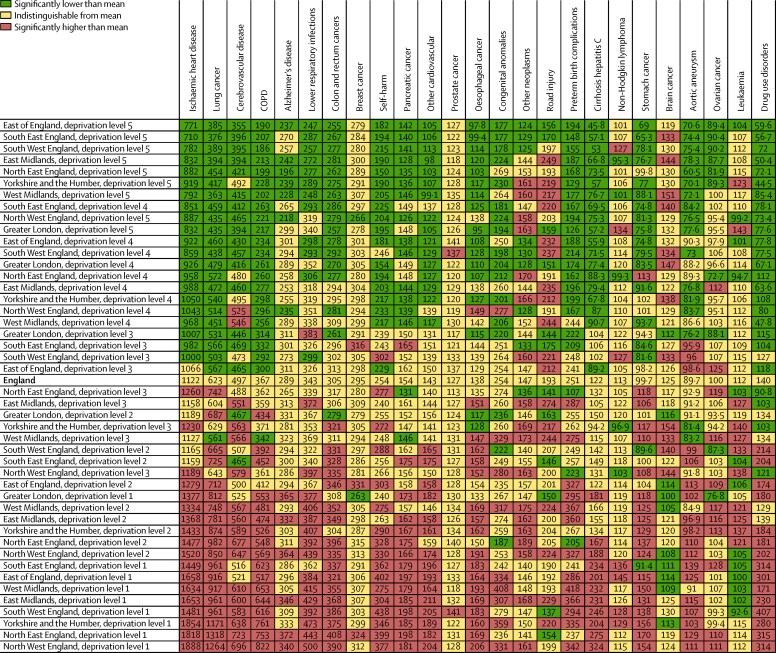
Age-standardised rates of years of life lost (YLLs) for England relative to the deprivation levels in the nine English regions for both sexes combined in 2013 To facilitate comparison, England is shown in bold. Lung cancer=lung, bronchus, and trachea cancer. COPD=chronic obstructive pulmonary disease. Alzheimer's disease=Alzheimer's disease and other dementias. Other cardiovascular=other cardiovascular and circulatory diseases. Cirrhosis hepatitis C=cirrhosis of the liver secondary to hepatitis C. Brain cancer=brain and central nervous system cancer.

Figure 3 is a comparison of age-standardised YLL rates for causes within each deprivation area with the England rate in 2013. Some causes such as ischaemic heart disease or COPD are largely ordered following the overall age-standardised YLL rate, whereas other causes have distinct patterns. Breast cancer and prostate cancer show much less variation across the deprivation areas. Other neoplasms and road injuries have variable patterns: in some regions, deprivation level 5 (least deprived) has higher rates than deprivation level 1 (most deprived) in other regions. Lymphoma, leukaemia, and brain cancer have distinctive patterns that do not follow levels of deprivation. The ratio of maximum age-standardised YLL rate to minimum age-standardised YLL rate across deprivation areas as a measure of relative inequality ranges from 1·3 (low) for prostate cancer to 9·2 (high) for drug use disorders. Other examples of high ratios include COPD (4·4), and cirrhosis from hepatitis C (7·1). In 2013, 91·1% of the variance in YLLs for men is explained by deprivation area and only 5·1% by region (table 1); for women, 78·7% of the variance is explained by deprivation area and 11·7% by region (table 1).

**Figure 4:**
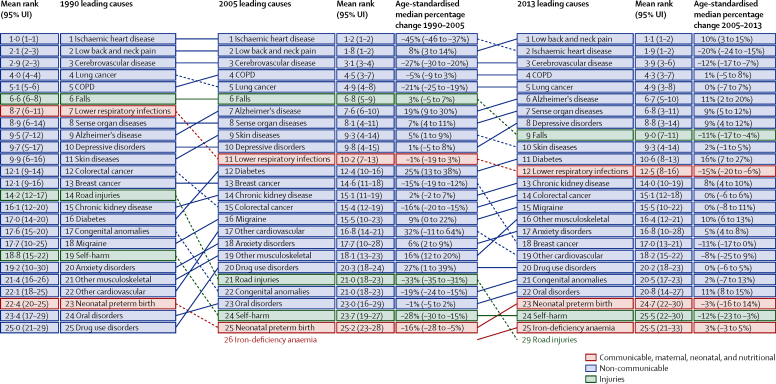
The 25 leading GBD level 3 causes of disability-adjusted life years (DALYs) in England, both sexes combined, 1990, 2005, and 2013, with age-standardised median percent change Ranks are based on the number of DALYs. 95% UIs for mean rank are from 1000 draws of DALYs. UI=uncertainty interval. COPD=chronic obstructive pulmonary disease.

Levels of YLDs from 1990 to 2013 have changed much less than YLLs for England (appendix p 21). Figure 4 provides the overall assessment of trends in DALYs using level 3 of the GBD cause hierarchy from 1990 to 2013. The leading cause of DALYs in 2013 is low back and neck pain. Sense organ diseases, consisting of hearing loss and vision loss, and depressive disorders are leading causes of DALYs although they do not cause substantial YLLs. Other causes such as chronic kidney disease, migraine, and eating disorders are important causes of YLDs and DALYs. Other musculoskeletal disorders, anxiety disorders, and drug use disorders are also leading causes of DALYs. Age-standardised DALY rates by deprivation areas for the leading causes of DALYs are provided in the appendix (p 2). Some causes, such as depressive disorders, show substantial variation among deprivation areas across English regions. For many causes that predominantly lead to YLDs, UIs are large such that nearly all deprivation areas have levels that are not significantly different from the England mean.

DALY trends are a composite of trends in YLLs and YLDs; trends that in some cases, might be going in opposite directions. Examples of disorders for which YLL and YLD rates are changing in different directions, or where all measures are increasing markedly, can be seen in table 2. For instance, age-standardised YLL rates for prostate cancer significantly declined by 20·9% (95% UI 6·46–31·37), whereas YLD rates significantly increased by 42·6% (2·52–72·19). Such examples show that only summary measures combining morbidity, mortality, and disability data provide a comprehensive understanding of the effect of a disorder on a population.

Estimates for deaths, YLLs, YLD, and DALYs in England in 1990 and 2013 for all ages and age-standardised rates are provided in the appendix (pp 23–40).

**Figure 5:**
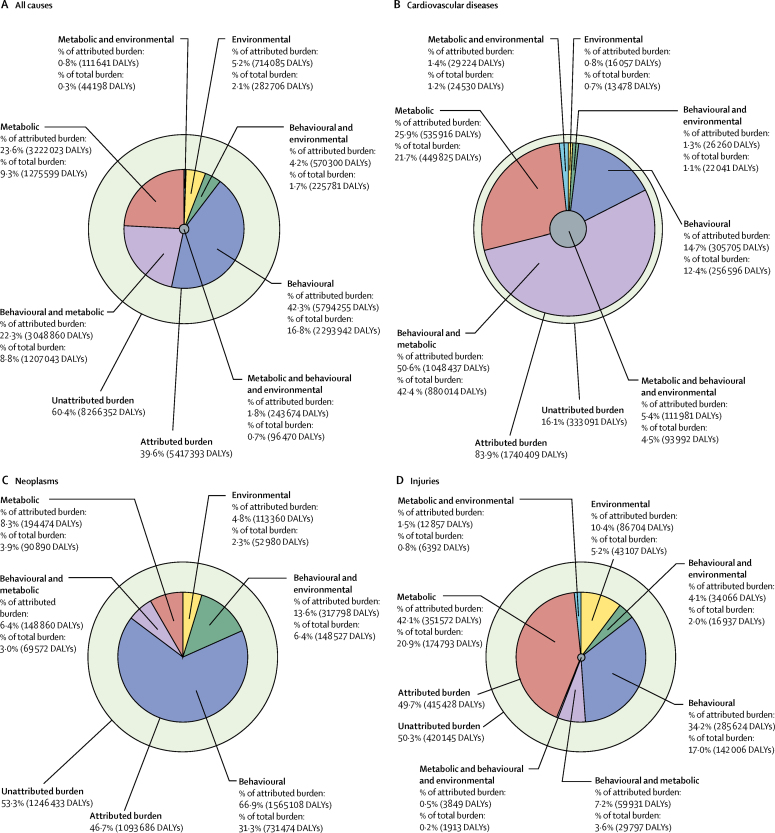
Proportion of all-cause DALYs (A), cardiovascular disease DALYs (B), neoplasm DALYs (C), and injury DALYs (D) attributable to behavioural, environmental and occupational, and metabolic risk factors and their overlaps for all ages in 2013

Overall for England in 2013, all identified risk factors jointly explain 39·6% (95% UI 37·7–41·7) of DALYs, with the remaining 60·4% DALYs as yet unexplained by the risk factors analysed (figure 5). Risk factors together explain 83·9% (81·6–86·2) of cardiovascular disease DALYs, 46·7% (44·5–49·3) of neoplasm DALYs, 49·7% (46·5–52·6) of injury DALYs, 62·0% (57·9–65·9) of chronic respiratory disease DALYs, but only 1·9% (1·4–2·4) of neurological disease DALYs (figure 5; DALYs attributable to risk factors for chronic respiratory disease and neurological disease are not presented; for DALYs attributable to all risk factors, see appendix p 41).

Behavioural risks account for 28·0% (95% UI 25·6–30·3) of DALYs, metabolic risks for 19·2% (18·0–20·5), and environmental and occupational risks for 4·7% (4·3–5·2). Almost half of DALYs due to metabolic risks overlap with behavioural risks, which is particularly large for cardiovascular disease. Behavioural risks (particularly tobacco and dietary risks) are the greatest contributor to cancer with more modest contributions from metabolic risks and environmental and occupational risks. Low bone mineral density and alcohol consumption are the dominant metabolic and behavioural risks, respectively, for injuries (figure 5).

**Figure 6:**
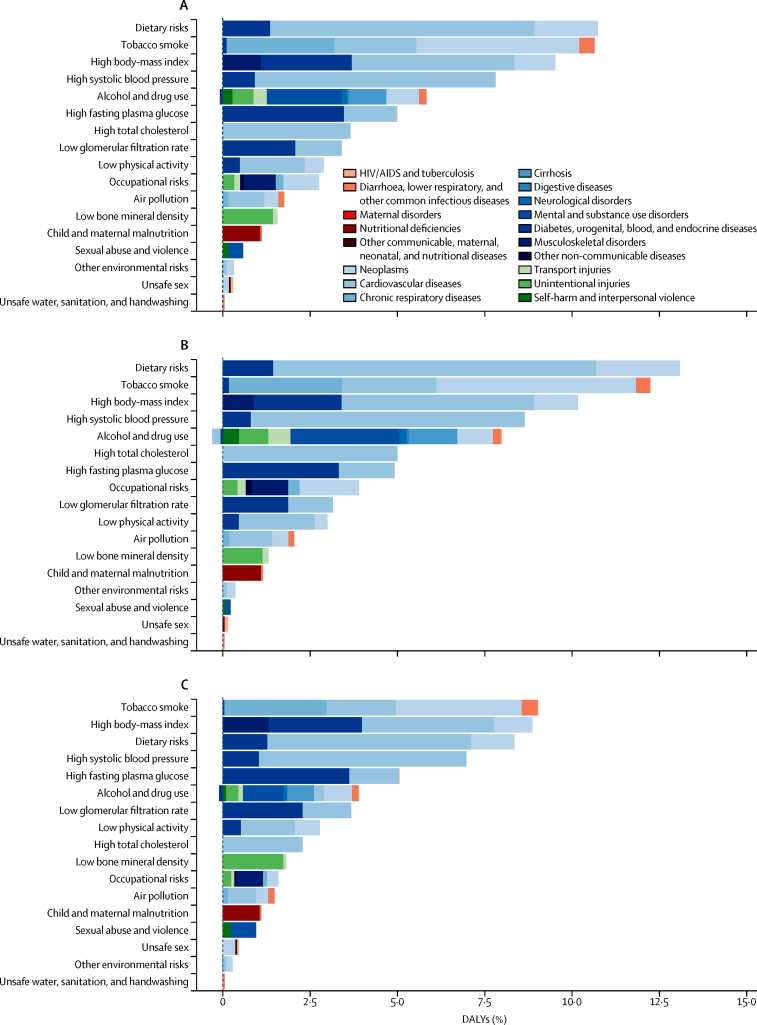
Disability-adjusted life-years (DALYs) attributed to level 2 risk factors in 2013 in England for both sexes combined (A), men (B), and women (C)

The largest contributor to DALYs are dietary risks (10·8%, 95% UI 9·1–12·7), an aggregate in the second tier of the GBD risk hierarchy of low fruit consumption, low vegetables consumption, low whole-grains consumption, low nuts and seeds consumption, low milk consumption, high red meat consumption, high processed meat consumption, high sugar-sweetened beverages consumption, low fibre consumption, suboptimal calcium intake, low seafood omega-3 fatty acids consumption, low polyunsaturated fatty acids consumptions, high trans fats intake, and high sodium intake, closely followed by tobacco (10·7%, 9·4–12·0) (figure 6).

The rates of DALYs attributed to each of the three major risk categories have declined between 1990 and 2013: by 28·3% (95% UI 25·0–31·5) for metabolic risks, by 23·7% (21·5–26·0) for behavioural risks, and by 28·8% (23·7–35·0) for environmental and occupational risks (data not shown). Declines are due to reductions in DALY rates attributable to most risk factors: between 1990 and 2013, DALY rates significantly declined for 11 of the 15 largest risks at tier 3 in the risk hierarchy (tobacco, high blood pressure, cholesterol, low glomerular filtration rate, low physical activity, diet low in fruit, vegetables, nuts, and seeds, diet high in sodium and processed meat, and air pollution), but remained unchanged for high body-mass index (BMI), high fasting plasma glucose, and alcohol. Illicit drug use is the only top 15 risk for which the DALY rates increased significantly. These changes in DALY rates attributed to major risks can be caused by a change in risk exposure, a change in outcomes associated with the risk, or a combination of the two. There was a significant decline in exposure to tobacco, high blood pressure, cholesterol, and air pollution. Exposure to the top dietary risks, physical activity, alcohol use, and low glomerular filtration rate changed little. Exposure to high BMI and high fasting plasma glucose increased significantly, but large declines in cardiovascular disease outcomes are mainly responsible for the difference in trends between exposure and attributable burden. Of the ten leading tier 2 risks, seven cause a greater proportion of total DALYs in men than in women. The exceptions are physical inactivity, fasting plasma glucose, and low glomerular filtration rate, with equal proportions of DALYs between men and women (figure 6).

The leading risk factors by deprivation level in England are shown in the appendix (p 3). There is a consistency of rank order of major risk factors: smoking, high BMI, and high blood pressure are the leading risks in all deprivation areas. Smoking is strongly socially stratified: it ranks above high BMI in deprivation level 1 (most deprived) in all English regions, but the converse is true in deprivation levels 4 and 5 across seven of those nine regions. In deprivation level 1 in six regions, alcohol use is the fourth leading risk factor, whereas it is high fasting plasma glucose for other regions. Drug use is a more highly ranked risk factor in most of the more deprived areas when compared with less deprived areas.

## Discussion

England had better than average outcomes in 2013 when compared with EU15+ countries and other countries of the UK. Despite sharing the same health and social care system, some English regions have outcomes commensurate with, or better than, those of the best-performing nations among the EU15+ countries, whereas others have outcomes worse than any of these countries. Life expectancy has increased at all levels of deprivation within English regions, but the variation in life expectancy within regions has decreased only slightly for women and not at all for men.

Routine annual mortality statistics show a year-on-year decline in age-standardised mortality rates in England and the rest of the UK from 1983 to 2013:^30^ mortality rates in Scotland are known to have been higher than in England, whereas mortality rates in Wales and Northern Ireland, although higher, have generally been closer to those in England. Routine statistics show that the range in life expectancy at birth between regions has reduced for men in the past 5 years, from 2·7 years to 2·4 years, but stayed the same for women, at 2·4 years.^31^ Our results show a larger range in life expectancy between deprivation areas, at 8·2 years for men and 6·9 years for women in 2013, with only a small reduction since 1990 for women and none for men. Routine mortality statistics undifferentiated by deprivation thus mask substantial inequalities within English regions (figure appendix 2). Consistent with other analyses,^32^, ^33^, ^34^, ^35^ geography accounted for only a small proportion of the variance between deprivation areas (table 1). At the district level, populations are more homogeneous, and inequalities in mortality rates more evident. District-level data reported by Bennett and colleagues^36^ suggest inequalities at this level are increasing. Inequality within regions is therefore greater than it is between them, and it is therefore important to address the problems caused by deprivation wherever they occur and in all regions of the country.

Many of the most common causes of DALYs show striking variation by deprivation area (appendix p 2); as for mortality, this inequality largely follows patterns determined by level of deprivation, not geography. For low-mortality disorders, such as musculoskeletal disease, DALYs tend to be based on incomplete data at subnational level, and the level of inequality for many disorders is likely to be underestimated.

The Greater London deprivation groups are an exception to the overall pattern of inequality because their burden of disease is less than expected for their level of deprivation (see figure appendix 2 and appendix p 2). Possible explanations include artifact due to the method of measuring deprivation, ethnic composition of the population, healthy migrant effect, and differential selective migration within England.^37^ London has also received more health investment than other English regions. The effect of health-care spending on the outputs from GBD would be a useful next stage of analysis.

Improvements in life expectancy in England, as elsewhere, have been driven by decreases in mortality from cardiovascular disease and cancers.^38^ Increasing mortality from neurological disorders and cirrhosis of the liver have partially offset these gains. In other countries, the negative effect of liver disease has been much less marked (see France, Spain, Sweden, and Italy in figure appendix 2). This rising burden of liver disease in England requires both a policy and health-care response.^39^ Among the other leading causes of premature death, Alzheimer's disease and other dementias are increasingly important, although age-specific death rates have declined slightly, consistent with a decline observed in serial prevalence surveys.^21^, ^40^, ^41^, ^42^

Significant differences in YLL rates by country for specific causes (figure 2) include various cancers. International and subnational differences in mortality are likely to be strongly driven by differences in incidence as well as in survival (eg, for lung cancer). Any national strategy for cancer needs to address primary prevention as well as effective care for people with cancer.

Musculoskeletal disorders are a dominant cause of YLDs, and consume a substantial amount of health-system resources. These disorders are strongly age-related and will become increasingly prevalent as the population ages.^43^ Another major contributor to YLDs are depressive and anxiety disorders, the rates of which have not declined. Given the epidemiological assessment of relative burden, services for these disorders rarely receive the attention they deserve. This relative neglect is beginning to change with calls for parity of esteem for mental and physical health. Opportunities to improve management of anxiety and depression in primary care exist;^44^, ^45^, ^46^ evidence suggests that prevention aimed at high-risk groups before depression arises is effective.^47^

For several disorders, although mortality rates may have reduced, the burden of ill health has either not declined by the same extent or is increasing. In England, and in other high-income countries, a larger share of DALYs now comes from YLDs, rather than YLLs. For example, in cerebrovascular disease and diabetes, YLLs have decreased, but YLDs have increased significantly; for COPD, YLLs have decreased significantly but with almost no change in YLDs. Whereas mortality rates from cardiovascular complications of diabetes have fallen, the prevalence of people living with diabetes is rising. Data from the National Diabetes Audit show that 36·0% of people with diagnosed diabetes living in England are estimated to have had their HbA_1c_, blood pressure, and cholesterol values treated to target in 2012–13.^48^ These data together indicate a high level of unmet need for prevention of the consequences of diabetes and for the active management of long-term disorders in general.

In some cases, the increasing relative contribution of YLDs might be the effect of improved survival (eg, from stroke and some cancers), leading to higher prevalence and many people living with disorders that previously would have proved fatal. Survivors will also be at risk of developing other disorders, particularly disorders associated with ageing, leading to steadily increasing lifetime risks of some cancers and of musculoskeletal disease, for example. One consequence of the growing phenomenon of survivorship, together with the effect of population ageing, will be much higher numbers of patients with multimorbidity than in the past.

A major strength of the GBD study is the ability to estimate the contribution of different causal factors to the burden of mortality and morbidity and, by implication, the extent to which that burden could be reduced by modifying those risks. GBD 2013 was developed to increase understanding of the individual and overlapping effects of different risk factors, generating outputs that are more informative to formulate preventive strategies. The overall proportion of preventable burden reduced between 1990 and 2013 because of the decline in the incidence of the more preventable diseases (ischaemic heart disease and some cancers) and a relative increase in other diseases with an apparently lower potential for prevention (neurological disorders, and Alzheimer's disease and other dementias).

Important metabolic risks, including high blood pressure, high fasting plasma glucose, low glomerular filtration rate, and high cholesterol, overlap significantly with modifiable behavioural risk factors, such as diet and physical activity; for example, mean systolic blood pressure in the UK has fallen by over 3 mm Hg in the last decade, mainly as a result of reduced dietary salt intake.^49^, ^50^ In general, behavioural risk factors make a greater contribution to DALYs (28%) than metabolic risks (19%) or environmental and occupational risks (5%). The relative contribution of individual risks and type of risk will vary by disorder. Environmental risks, such as air pollution, albeit smaller than the other two risk categories, remain quantitatively important and require specific consideration within national and local public health policy and strategy.

The combination of unhealthy diets, physical inactivity, and high BMI is the biggest overall contributor to DALYs. Tobacco smoking remains a leading attributable risk factor for DALYs in England, and is notably still the leading risk factor for women. In line with this, mortality from lung cancer for men declined by 24% between 1990 and 2013 but did not change significantly for women. The peak effect of smoking in women is probably only now being reached in England.^38^

Alcohol consumption is the third leading behavioural risk factor overall but is the leading behavioural cause of injury. In GBD 2013, only about a third of deaths from cirrhosis could categorically be assigned to alcohol as the underlying cause (cirrhosis due to alcohol accounts for only 29·4% of DALYs due to cirrhosis; appendix p 36); however, alcohol also contributes to cirrhosis where it is not the underlying cause. In the risk factor analysis, therefore, the proportion of cirrhosis of the liver DALYs attributed to alcohol in England was 69·5% in 2013. This estimate is comparable to findings of another study^39^ that showed 75% of cirrhosis of the liver to be attributable to alcohol. Improved data on cirrhosis of the liver and liver cancer by aetiology are needed, including a better assessment of the role of obesity as a cause of fatty liver disease.^51^, ^52^, ^53^, ^54^, ^55^ The contributions of various aetiologies to cirrhosis of the liver will probably vary geographically across England.

Most, if not all, behavioural, metabolic, and environmental and occupational risks are strongly related to the socioeconomic determinants of health, which need to be addressed in any credible public health strategy.^56^ In future rounds of the GBD study, we plan to quantify socioeconomic determinants of health more directly.

This study has several important limitations beyond those applying more generally to GBD studies and reported elsewhere.

First, the level of aggregation of causes affects their relative ranking (eg, splitting cirrhosis of the liver by cause affects where the individual causes appear in the rankings). The online data visualisations associated with this study allow the user to interrogate the results using different levels of the GBD cause hierarchy.

Second, subnational data at the regional level are not available for the estimation of prevalence and incidence for several diseases. Estimates depend on the GBD Bayesian models, borrowing from studies in other settings while using covariates as predictors. This limitation could be improved by collecting additional data on morbidity in the English population. Possible approaches include increasing the sample size of the Health Survey for England and augmenting the increased sample size with additional health examinations, incorporating data from large primary care surveys, such as the Clinical Practice Research Datalink, or further use of linked primary and secondary care datasets. These estimates might be affected by supply and access factors.

Third, we assigned each lower super output area on the basis of the level of deprivation in 2010 and used this assignment for the analysis in all time periods. As some of the lower super output areas may have changed their relative level of deprivation during the period, we might have underestimated some of the inequalities related to deprivation in 1990. Any reductions in inequality would probably be underestimated because of this approach. Such errors could be assessed by reference to longitudinal studies such as the ONS Longitudinal Study. Furthermore, deprivation is measured at the time that disease outcome is recorded, whereas the contribution of deprivation is relevant across the lifecourse. The estimation of the relation between deprivation and disease does not account for this, thereby potentially underestimating its effect in some areas and overestimating it in others.

Fourth, in view of the limited data by deprivation level for some causes that do not affect mortality, inequality across deprivation levels might be substantially underestimated in the analysis of YLDs.

Fifth, the current GBD method for risk factor attribution uses data on exposure to single risk factors and its effect on each outcome, whereas aggregated effects of risk factors are made on the basis of assumptions of additive, multiplicative, or mediated effects and not on the direct measurement of exposure to combined risk factors and the combined effect on outcomes.

Finally, in some analyses, comparisons are made between English regions and other countries. Although some English regions are similar in size to several of the countries in the EU, the potential for differential migration between English regions is greater than between countries; such comparisons are not straightforward.

Some limitations are specific to certain disorders. The comparative risk assessment, based on the criterion of convincing or probable evidence, did not identify any modifiable risk factors for Alzheimer's disease and other dementias as suitable targets for public health action. Some studies suggest that modifiable behavioural risks do exist,^57^, ^58^, ^59^, ^60^, ^61^, ^62^ but more research is needed to determine whether these findings would meet the criterion for inclusion in the GBD framework. In future rounds of GBD, we intend to make separate estimates for Alzheimer's disease and vascular dementia and will assess the evidence for the effect of some metabolic and behavioural risk factors on vascular dementia in particular. In the GBD 2013 framework, dementia in Parkinson's disease or in stroke is considered in the respective disease categories rather than in the Alzheimer's and other dementias category.

We estimate that about 3600 deaths were attributable to self-harm in England in 2013. This differs from official figures released by the Office for National Statistics, which indicate 4722 suicides in England in 2013.^63^ This difference is due to the partial redistribution in the GBD study of ICD-10 mortality codes for injury and poisoning of undetermined intent (ICD-10 codes Y10–Y34) to suicide as opposed to all these codes being counted as suicide by the Office for National Statistics; our estimates will therefore underestimate the overall suicide burden in England compared with official figures. Furthermore, when we estimated the attributable risk for mental and substance use disorders and self-harm, social risk factors (for example, divorce, low income, debt and job loss) were not included and therefore not attributed.

The contribution of diet components other than sugar-sweetened beverages to BMI has not been included in this analysis; therefore, the full consequences of diet might be greater than the effect shown in these results.

No comprehensive picture has been made of the level and distribution of the burden of disease in England, despite the fact that health policy is now devolved to individual countries within the UK. The outputs of such a framework can help test whether the effort being expended on a particular disease or risk factor is proportionate to burden or attributable risk, respectively. Our results therefore have implications for national government, local government, and health services in England.

For national government, the quantification by the GBD study of the continuing burden of preventable ill health more than justifies recent calls for a “radical upgrade in prevention and public health”.^3^ National government will also be concerned that improvements in premature mortality in England are diminished by increases in deaths from liver disease, by contrast with the declines seen in similar countries such as France.

For English local authorities in their new role as leaders for local population health, the most striking findings relate to inequalities, not only the size and nature of the effect of deprivation on health but also that the gap between the most deprived and least deprived areas of England shows little sign of reducing. The GBD 2013 results underline the fact that local authorities in the more disadvantaged regions of England are not the only ones that need to tackle the effects of deprivation, and to make this task a priority.

For health services, one important implication of our results is the scale of the increasing level of disability and, especially, the growth of multimorbidity. This new form of demand requires new and more integrated models of care spanning health and social services that respond to the specific needs and circumstances of individual patients. The other important implication for health services is that about 40% of their workload is potentially preventable, yet the proportion of health expenditure directed at prevention, although hard to estimate reliably, is probably closer to 4%.^64^

Combining GBD results with data on expected trends in population structure and risk factors could be used to anticipate future disease burden and thereby provide an important input to economic models and forecasts. This will be addressed in future GBD work. The importance of prevention argues for robust, standardised, comparative assessments of the cost-effectiveness of different public health policies to aid decision making, akin to those used in England for health technologies. Such assessments are likely to highlight the need for more empirical evidence of the effectiveness of specific preventive approaches. Finally, there are substantial gaps in routine and survey data on the prevalence of morbidity at the subnational level that could be addressed.

If the levels of health seen in the best performing English regions could be achieved in the worst performing regions, England would have a level of overall burden of disease as low as any country with a developed post-industrial economy. This interpretation provides a strong argument for implementing policies for effective prevention and treatment, but these policies must also be designed to reduce inequalities associated with the socioeconomic determinants of health.

## Figures and Tables

**Table 1:** Decomposition of variance in deaths, YLLs, YLDs, DALYs, and life expectancy for men and women, separately, into contributions from level of deprivation and English region for 1990, 1995, 2000, 2005, 2010, and 2013

	**Death**		**YLLs**		**YLDs**		**DALYs**		**Life expectancy**	
	Deprivation	Region	Deprivation	Region	Deprivation	Region	Deprivation	Region	Deprivation	Region
**Men**										
1990	86·8% (65·3–96·9)	7·8% (1·2–24·1)	92·5% (78·2–98·5)	5·2% (0·9–17·6)	86·4% (64·6–96·9)	8·5% (1·3–25·9)	96·3% (88·4–99·3)	2·2% (0·3–8·1)	94·2% (82·3–98·8)	4·3% (0·7–13·9)
1995	86·0% (64·6–96·6)	6·0% (0·8–19·5)	93·2% (80·7–98·6)	3·4% (0·5–11·9)	86·3% (66·3–96·6)	7·0% (1·0–20·8)	96·8% (90·7–99·3)	0·9% (0·1–3·7)	93·7% (82·0–98·6)	3·0% (0·4–10·2)
2000	89·7% (73·7–97·6)	4·6% (0·6–14·0)	94·5% (84·3–98·8)	3·2% (0·5–11·3)	89·5% (70·6–97·6)	6·8% (1·1–21·2)	97·7% (92·6–99·5)	0·6% (0·0–3·2)	95·0% (85·3–98·9)	2·4% (0·3–8·3)
2005	92·3% (78·6–98·4)	4·8% (0·7–16·1)	93·7% (81·7–98·6)	4·1% (0·7–13·2)	85·6% (62·9–96·3)	7·6% (1·1–24·9)	96·2% (89·0–99·1)	1·5% (0·1–5·3)	94·6% (83·8–98·9)	3·5% (0·5–11·5)
2010	92·1% (78·2–98·4)	4·5% (0·6–14·9)	92·6% (78·8–98·3)	4·1% (0·6–13·3)	84·0% (59·3–96·0)	9·9% (1·9–29·0)	95·5% (86·7–99·1)	1·2% (0·1–5·6)	94·4% (83·4–98·8)	3·3% (0·5–11·1)
2013	92·8% (80·9–98·5)	3·2% (0·4–10·4)	91·1% (75·1–98·3)	5·1% (0·7–16·0)	86·5% (63·3–97·0)	8·5% (1·4–24·7)	94·3% (84·7–98·8)	2·0% (0·2–7·3)	94·6% (84·5–98·8)	2·5% (0·3–8·6)
**Women**										
1990	80·1% (54·4–94·6)	9·8% (1·6–31·3)	86·4% (64·1–96·9)	6·8% (1·1–22·8)	86·2% (63·3–96·7)	5·6% (0·8–19·1)	89·2% (73·3–97·5)	4·5% (0·6–15·6)	86·0% (64·3–96·6)	6·7% (1·0–21·4)
1995	82·1% (56·6–95·6)	6·9% (0·9–21·6)	89·6% (70·5–97·4)	5·2% (0·7–17·9)	91·4% (75·2–98·2)	4·6% (0·6–14·8)	93·4% (82·1–98·6)	2·6% (0·3–8·9)	88·2% (68·9–97·2)	4·5% (0·5–15·2)
2000	79·1% (53·1–94·6)	6·2% (0·8–22·8)	85·8% (64·1–96·6)	6·8% (1·1–20·0)	87·4% (67·9–97·2)	4·8% (0·5–17·5)	90·8% (75·8–98·0)	3·2% (0·3–11·9)	85·0% (61·9–96·3)	3·6% (0·2–15·8)
2005	78·3% (48·1–95·3)	13·0% (2·1–35·3)	81·3% (53·3–95·3)	12·5% (2·0–35·5)	81·6% (55·1–95·8)	5·6% (0·6–22·8)	86·6% (66·9–96·9)	6·5% (0·9–20·9)	82·8% (57·4–95·8)	10·5% (1·9–29·3)
2010	80·9% (54·3–95·0)	9·6% (1·5–28·1)	79·1% (50·5–94·4)	12·8% (2·3–36·3)	70·8% (37·7–91·9)	22·1% (4·9–53·5)	85·6% (64·7–96·5)	5·9% (0·9–18·6)	84·5% (61·2–96·2)	7·9% (1·3–24·3)
2013	82·5% (56·0–95·7)	7·8% (1·2–27·4)	78·7% (50·3–94·1)	11·7% (2·3–32·8)	81·4% (57·1–95·5)	7·9% (1·0–26·3)	83·9% (59·8–96·1)	6·4% (0·7–22·7)	86·2% (64·7–96·6)	5·8% (0·8–18·8)

Data are percentage variance (95% uncertainty interval). YLLs=years of life lost. YLDs=years lived with disability. DALYs=disability-adjusted life-years. Deprivation=deprivation area. Region=English region.

**Table 2:** Change in age-standardised rates (per 100 000) of death, YLLs, YLDs, and DALYs for both sexes from 1990 to 2013 for select disorders in England and total DALYs in 2013

	**Deaths**	**YLLs**	**YLDs**	**DALYs**	**Total burden of DALYs 2013**
Cerebrovascular disease	−46·2% (−50·65 to −37·93)*	−54·9% (−59·01 to −49·67)*	14·2% (7·89 to 20·12)*	−49·8% (−54·40 to −44·69)*	535 900 (475 100 to 607 000)
Chronic obstructive pulmonary disease	−19·3% (−27·43 to −2·76)*	−32·4% (−39·25 to −21·41)*	−0·9% (−12·25 to 10·28)	−21·9% (−28·42 to −15·25)*	527 200 (448 800 to 620 400)
Alzheimer's disease and other dementias	6·9% (−6·42 to 20·11)	−3·4% (−14·31 to 6·87)	0·2% (−11·40 to 10·40)	−2·2% (−11·61 to 5·78)	463 400 (412 800 to 514 600)
Diabetes	−50·4% (−53·89 to −46·91)*	−55·8% (−58·66 to −52·73)*	75·3% (56·88 to 94·11)*	20·1% (5·51 to 34·98)*	360 200 (262 900 to 468 900)
Prostate cancer†	−16·2% (−44·83 to 0·52)	−20·9% (−51·37 to −6·46)*	42·6% (2·52 to 72·19)*	−14·2% (−45·70 to 1·21)	138 100 (93 300 to 186 200)
Liver cancer	56·6% (11·83 to 87·65)*	41·0% (6·19 to 66·01)*	72·3% (18·86 to 126·55)*	41·4% (6·34 to 66·45)*	49 400 (37 500 to 57 200)
Cirrhosis of the liver	41·7% (31·71 to 51·08)*	50·8% (40·64 to 61·31)*	26·2% (20·17 to 32·34)*	50·0% (40·15 to 60·25)*	181 000 (169 000 to 192 500)
Drug use disorders	113·5% (6·99 to 143·31)*	107·7% (4·07 to 137·47)*	−4·9% (−9·68 to 0·19)	18·2% (−1·56 to 28·47)	201 200 (157 300 to 246 500)

Data are percent change (95% UI). YLLs=years of life lost. YLDs=years lived with disability. DALYs=disability-adjusted life-years.

* Statistically significant.

† Men only.

## Supplementary Material

Supplementary appendix

Supplementary Newton_PullOutFig1

Supplementary Newton_PullOutFig2
