# Supplementary material for: Changes in health in England, with analysis by English regions and areas of deprivation, 1990–2013: a systematic analysis for the Global Burden of Disease Study 2013
Source: Lancet. 2015 Dec 5;386(10010):2257–74. doi: 10.1016/S0140-6736(15)00195-6 (PMC4672153; doi:10.1016/S0140-6736(15)00195-6)
Supplement: Supplementary appendix [file mmc1.pdf]

THE LANCET

Supplementary appendix

This appendix formed part of the original submission and has been peer reviewed. We post it as supplied by the authors.

Supplement to: Newton JN, Briggs ADM, Murray CJL, et al. Changes in health in England, with analysis by English regions and areas of deprivation, 1990–2013: a systematic analysis for the Global Burden of Disease Study 2013. *Lancet* 2015; published online September 15. http://dx.doi.org/10.1016/S0140-6736(15)00195-6.

Appendix to Changes in health in England, with analysis by English regions, and areas of deprivation, 1990–2013: a systematic analysis for the Global Burden of Disease Study 2013

This appendix provides three supplementary figures and nine supplementary tables.

Table of Contents

Section 1. Supplementary figures....1
Section 2. Supplementary tables....4

***Web Figure 1*: The 25 leading GBD level 3 causes of deaths in England, both sexes combined, 1990, 2005, and 2013, with age-standardized median percent change.** Ranks are based on the number of deaths. Mean rank across the 1000 draws of deaths are shown with 95% uncertainty intervals in ranks in parentheses. Changes in all age and in age-standardised deaths are also shown with 95% uncertainty intervals in parentheses. Communicable, maternal, neonatal, and nutritional disorders causes are shown in red, non-communicable causes in blue, and injuries in green.

Rank 1990	Leading causes 1990	Leading causes 2005	Rank 2005	Age-standardized % change 1990-2005	Leading causes 2013	Rank 2013	Age-standardized % change 2005-2013
1.0 (1-1)	1 Ischemic heart disease	1 Ischemic heart disease	1.0 (1-1)	-38% (-39 to -29%)	1 Ischemic heart disease	1.0 (1-1)	-14% (-18 to -8%)
2.0 (2-2)	2 Cerebrovascular disease	2 Cerebrovascular disease	2.0 (2-2)	-23% (-26 to -12%)	2 Cerebrovascular disease	2.1 (2-3)	-4% (-10 to 2%)
3.1 (3-4)	3 Lung cancer	3 Lower respiratory infections	3.4 (3-4)	10% (-5 to 17%)	3 Alzheimer disease	3.0 (2-4)	18% (5 to 32%)
3.9 (3-4)	4 Lower respiratory infections	4 Alzheimer disease	3.6 (3-4)	28% (13 to 47%)	4 Lower respiratory infections	4.0 (3-5)	-5% (-12 to 7%)
5.3 (5-6)	5 Alzheimer disease	5 Lung cancer	5.1 (5-6)	-14% (-21 to -11%)	5 Lung cancer	5.1 (4-6)	5% (-2 to 12%)
5.7 (5-6)	6 COPD	6 COPD	5.9 (5-6)	2% (-3 to 19%)	6 COPD	5.8 (4-6)	5% (-2 to 13%)
7.0 (7-7)	7 Colorectal cancer	7 Colorectal cancer	7.0 (7-7)	-13% (-16 to -10%)	7 Colorectal cancer	7.0 (7-7)	7% (0 to 13%)
8.0 (8-8)	8 Breast cancer	8 Breast cancer	8.1 (8-9)	-15% (-20 to -12%)	8 Breast cancer	8.2 (8-10)	-5% (-13 to 5%)
9.2 (9-11)	9 Stomach cancer	9 Other cardiovascular	9.2 (8-11)	37% (-24 to 46%)	9 Other cardiovascular	9.4 (8-13)	-4% (-16 to 5%)
10.4 (9-13)	10 Aortic aneurysm	10 Prostate cancer	10.1 (8-12)	16% (-10 to 32%)	10 Prostate cancer	9.8 (8-14)	13% (-8 to 24%)
11.0 (9-13)	11 Other cardiovascular	11 Aortic aneurysm	11.0 (10-14)	-4% (-17 to 1%)	11 Pancreatic cancer	11.1 (10-12)	13% (6 to 19%)
11.8 (9-15)	12 Prostate cancer	12 Pancreatic cancer	12.0 (11-13)	7% (2 to 10%)	12 Aortic aneurysm	12.8 (10-16)	-10% (-17 to -1%)
12.7 (11-13)	13 Diabetes	13 Stomach cancer	13.7 (11-16)	-31% (-37 to -27%)	13 Esophageal cancer	13.8 (10-16)	6% (-5 to 23%)
13.9 (13-14)	14 Pancreatic cancer	14 Esophageal cancer	14.0 (12-15)	25% (20 to 30%)	14 Urinary diseases	14.5 (11-27)	14% (-4 to 25%)
15.3 (15-18)	15 Self-harm	15 Urinary diseases	15.8 (13-27)	150% (1 to 167%)	15 Falls	15.0 (13-21)	10% (-1 to 21%)
16.4 (15-21)	16 Cardiomyopathy	16 Falls	16.3 (15-20)	72% (4 to 85%)	16 Stomach cancer	15.3 (13-17)	-6% (-12 to 3%)
17.0 (15-19)	17 Esophageal cancer	17 Diabetes	17.0 (15-18)	-29% (-33 to -23%)	17 Other neoplasms	17.1 (15-21)	9% (0 to 18%)
18.5 (16-23)	18 Bladder cancer	18 Other neoplasms	17.8 (16-21)	33% (-1 to 39%)	18 Diabetes	18.4 (16-20)	-6% (-12 to 0%)
19.3 (17-21)	19 Road injuries	19 Lymphoma	19.6 (16-27)	37% (-11 to 42%)	19 Lymphoma	19.5 (15-29)	7% (-14 to 17%)
19.7 (17-24)	20 Chronic kidney disease	20 Ovarian cancer	20.3 (18-23)	5% (-5 to 9%)	20 Chronic kidney disease	20.1 (16-23)	19% (6 to 28%)
21.4 (18-26)	21 Peptic ulcer disease	21 Self-harm	20.9 (17-23)	-27% (-29 to -12%)	21 Parkinson disease	21.5 (18-27)	12% (0 to 22%)
22.3 (18-25)	22 Other neoplasms	22 Parkinson disease	22.2 (19-29)	14% (5 to 22%)	22 Atrial fibrillation	22.5 (18-28)	43% (25 to 63%)
22.6 (20-25)	23 Ovarian cancer	23 Chronic kidney disease	23.1 (17-26)	-17% (-21 to 9%)	23 Ovarian cancer	23.7 (21-27)	0% (-8 to 10%)
24.8 (24-27)	24 Leukemia	24 Leukemia	24.0 (22-26)	7% (0 to 10%)	24 Leukemia	23.7 (21-26)	9% (-2 to 16%)
25.3 (17-27)	25 Falls	25 Bladder cancer	24.3 (19-27)	-22% (-25 to -11%)	25 Bladder cancer	25.0 (20-29)	5% (-6 to 14%)
	26 Parkinson disease	26 Peptic ulcer disease			26 Self-harm		
	27 Lymphoma	27 Road injuries					
	29 Urinary diseases	30 Atrial fibrillation					
		32 Cardiomyopathy					

Legend:
Communicable, maternal, neonatal and nutritional
Non-communicable
Injuries

Web Figure 2 **: Age-standardized rates of disability-adjusted life years (DALYs) for England relative to the deprivations in the nine regions for both sexes combined in 2013**

	Low back & neck pain	Ischemic heart disease	Cerebrovascular disease	COPD	Lung cancer	Alzheimer disease	Sense organ diseases	Depressive disorders	Falls	Skin diseases	Diabetes	Lower respiratory infections	Chronic kidney disease	Colorectal cancer	Migraine	Other musculoskeletal	Anxiety disorders	Breast cancer	Other cardiovascular	Drug use disorders	Congenital anomalies	Oral disorders	Neonatal preterm birth	Self-harm	Iron-deficiency anemia
East of England, Deprivation Level 5	2062	843	438	387	391	371	514	750	463	730	430	248	371	270	473	366	454	305	197	238	334	267	309	184	336
South East England, Deprivation Level 5	2041	778	487	401	382	421	509	690	463	688	404	289	399	282	495	366	454	311	190	220	327	268	261	196	339
South West England, Deprivation Level 5	2032	857	482	378	395	397	578	503	406	713	433	258	393	293	472	373	452	306	215	237	333	264	280	217	340
East Midlands, Deprivation Level 5	1957	911	470	390	400	373	589	712	436	742	413	273	380	297	484	359	454	328	185	221	372	265	295	192	340
North West England, Deprivation Level 5	1823	955	545	374	442	334	582	726	372	726	353	320	356	295	466	370	454	292	232	207	401	267	314	206	338
North East England, Deprivation Level 5	2077	956	492	329	461	302	577	848	401	689	289	279	441	277	478	380	453	315	201	204	460	265	267	152	338
Yorkshire and the Humber, Deprivation Lev	2079	999	577	378	423	371	574	702	435	746	448	290	403	291	485	354	452	317	206	169	392	265	241	192	367
West Midlands, Deprivation Level 5	2116	861	489	365	369	354	579	626	435	852	414	248	387	278	482	362	452	335	205	277	437	266	313	207	344
South East England, Deprivation Level 4	2042	931	507	496	467	414	508	690	475	696	426	295	390	302	495	374	455	325	243	270	333	268	281	228	340
Greater London, Deprivation Level 5	2103	901	478	407	442	395	560	691	466	710	309	342	488	272	495	390	454	304	212	255	391	262	235	197	332
South West England, Deprivation Level 4	2032	943	558	463	445	460	578	504	463	714	448	294	405	309	472	372	453	331	244	247	366	264	339	248	338
North West England, Deprivation Level 4	1824	1122	613	478	523	362	581	727	403	737	384	352	372	297	466	364	454	321	263	222	463	267	287	235	342
East of England, Deprivation Level 4	2061	1008	533	471	468	471	514	750	484	737	474	299	422	294	472	378	453	330	226	279	436	267	303	183	337
North East England, Deprivation Level 4	2076	1040	561	432	582	397	577	848	449	688	372	307	458	293	476	381	453	306	244	275	385	265	262	197	341
Greater London, Deprivation Level 4	2103	1004	508	477	487	383	560	691	502	715	388	353	391	285	493	382	454	334	257	240	385	262	283	155	334
East Midlands, Deprivation Level 4	1957	1080	551	496	480	394	589	711	453	750	453	320	395	311	483	370	453	333	243	245	431	265	307	206	341
Yorkshire and the Humber, Deprivation Lev	2079	1139	579	496	549	397	574	703	451	750	481	320	429	311	486	356	454	327	234	286	358	265	312	219	365
West Midlands, Deprivation Level 4	2116	1053	644	448	458	453	578	625	504	859	442	340	402	326	480	366	452	326	243	215	369	266	381	219	345
Greater London, Deprivation Level 3	2102	1091	544	562	539	411	560	691	473	717	438	385	409	276	492	388	453	318	264	347	396	262	333	242	335
South West England, Deprivation Level 3	2032	1095	580	568	511	427	578	504	480	721	489	301	393	319	472	373	453	334	264	350	438	264	376	306	339
South East England, Deprivation Level 3	2040	1075	578	622	575	469	508	690	531	697	474	328	400	313	494	374	454	346	268	333	433	268	323	245	344
East of England, Deprivation Level 3	2062	1165	579	587	577	488	513	751	510	745	519	328	416	332	473	382	454	327	280	367	445	267	357	232	337
North East England, Deprivation Level 3	2077	1367	569	573	754	405	576	848	472	692	417	341	402	334	476	376	453	308	271	258	456	265	206	281	343
**England**	**2029**	**1220**	**598**	**644**	**633**	**442**	**558**	**688**	**494**	**738**	**480**	**344**	**411**	**323**	**481**	**372**	**454**	**323**	**272**	**391**	**430**	**266**	**369**	**257**	**342**
East Midlands, Deprivation Level 3	1956	1267	659	624	613	488	589	711	452	756	482	374	425	324	482	364	453	339	269	322	432	265	401	242	339
North West England, Deprivation Level 3	1824	1281	677	578	653	440	581	727	475	735	422	399	445	354	465	370	454	308	282	305	466	267	352	269	340
Yorkshire and the Humber, Deprivation Lev	2078	1334	662	606	640	436	574	702	451	749	483	354	399	339	484	362	453	334	268	289	469	265	376	275	367
Greater London, Deprivation Level 2	2102	1288	570	741	698	439	560	691	500	719	486	368	402	295	493	378	454	306	312	398	407	262	364	257	332
West Midlands, Deprivation Level 3	2116	1225	669	579	570	505	578	626	524	858	521	370	396	328	480	368	452	322	291	380	517	266	414	250	348
South West England, Deprivation Level 2	2031	1277	621	751	676	460	577	504	533	722	536	324	436	350	471	375	453	326	311	531	391	264	376	291	341
South East England, Deprivation Level 2	2040	1269	574	831	736	468	508	690	505	699	504	342	431	347	493	375	454	314	309	594	422	268	372	259	340
East of England, Deprivation Level 2	2063	1399	627	800	724	463	512	751	488	747	568	369	415	366	473	394	454	361	296	532	411	267	444	306	342
Yorkshire and the Humber, Deprivation Lev	2078	1554	697	840	888	471	574	703	491	757	536	408	434	321	484	354	453	314	304	447	425	265	379	293	364
East Midlands, Deprivation Level 2	1956	1494	673	818	793	517	588	712	556	765	576	389	423	369	483	369	453	327	297	425	448	265	471	266	342
Greater London, Deprivation Level 1	2103	1491	642	943	825	482	560	691	517	722	580	378	421	325	493	388	454	289	364	484	452	262	403	242	333
West Midlands, Deprivation Level 2	2116	1450	672	813	760	458	577	626	543	858	592	408	475	372	480	371	452	335	302	400	523	266	506	278	350
North East England, Deprivation Level 2	2078	1601	794	880	998	481	576	849	500	694	448	394	415	418	478	376	454	345	303	428	357	265	304	332	343
North West England, Deprivation Level 2	1823	1636	760	892	864	562	581	727	549	740	491	441	419	353	464	372	454	344	327	451	448	267	448	334	341
South East England, Deprivation Level 1	2038	1584	643	1136	976	448	508	689	611	713	576	364	449	357	492	380	453	319	348	867	435	268	358	365	340
West Midlands, Deprivation Level 1	2116	1772	724	1087	931	475	578	627	583	863	750	416	459	375	481	373	453	337	338	491	594	266	557	278	348
East of England, Deprivation Level 1	2062	1809	658	992	930	464	512	751	493	746	662	386	498	340	472	381	453	336	356	879	560	267	403	406	337
East Midlands, Deprivation Level 1	1956	1803	726	1114	976	537	588	712	586	760	668	431	503	389	483	370	454	337	394	637	502	265	476	308	344
South West England, Deprivation Level 1	2030	1623	710	1162	976	484	578	504	512	727	584	394	487	407	471	380	453	333	384	957	460	264	421	443	343
North East England, Deprivation Level 1	2077	1967	858	1179	1339	575	576	849	584	687	509	445	416	431	478	380	454	355	348	663	420	265	337	404	341
Yorkshire and the Humber, Deprivation Lev	2078	2007	758	1219	1190	517	574	703	597	752	633	475	414	396	484	356	453	329	357	647	561	264	450	349	368
North West England, Deprivation Level 1	1823	2028	821	1297	1284	524	581	727	671	743	573	501	441	412	465	371	454	342	382	672	507	267	466	381	342

Significantly lower than mean — Indistinguishable from mean — Significantly higher than mean

Web Figure 3: Top 10 GBD Level 4 risks of DALYs for both sexes combined by location in 2013

The top 15 GBD Level 4 risks of DALYs in England have been assigned a specific color to aid in observing subnational patterns that differ from national pattern

	1	2	3	4	5	6	7	8	9	10
**England**	Smoking	High body-mass index	High blood pressure	High fasting plasma glucose	Alcohol use	High FPG (continuous)	High total cholesterol	Low glomerular filtration	Drug use	Low physical activity
**East Midlands**	High body-mass index	Smoking	High blood pressure	High fasting plasma glucose	Alcohol use	High FPG (continuous)	High total cholesterol	Low glomerular filtration	Low physical activity	Low fruit
East Midlands, Deprivation Level 1	Smoking	High body-mass index	High blood pressure	High fasting plasma glucose	Alcohol use	High FPG (continuous)	High total cholesterol	Drug use	Low glomerular filtration	Low physical activity
East Midlands, Deprivation Level 2	Smoking	High body-mass index	High blood pressure	High fasting plasma glucose	Alcohol use	High FPG (continuous)	High total cholesterol	Low glomerular filtration	Low physical activity	Drug use
East Midlands, Deprivation Level 3	High body-mass index	Smoking	High blood pressure	High fasting plasma glucose	Alcohol use	High FPG (continuous)	High total cholesterol	Low glomerular filtration	Low fruit	Low physical activity
East Midlands, Deprivation Level 4	High body-mass index	Smoking	High blood pressure	High fasting plasma glucose	Alcohol use	High FPG (continuous)	Low glomerular filtration	High total cholesterol	Low physical activity	Low fruit
East Midlands, Deprivation Level 5	High body-mass index	Smoking	High blood pressure	High fasting plasma glucose	Alcohol use	High FPG (continuous)	Low glomerular filtration	High total cholesterol	Low physical activity	Low fruit
**East of England**	High body-mass index	Smoking	High blood pressure	High fasting plasma glucose	Alcohol use	High FPG (continuous)	Low glomerular filtration	High total cholesterol	Low physical activity	Drug use
East of England, Deprivation Level 1	Smoking	High body-mass index	High blood pressure	Alcohol use	High fasting plasma glucose	High FPG (continuous)	Drug use	High total cholesterol	Low glomerular filtration	Low physical activity
East of England, Deprivation Level 2	Smoking	High body-mass index	High blood pressure	High fasting plasma glucose	Alcohol use	High FPG (continuous)	High total cholesterol	Low glomerular filtration	Drug use	Low physical activity
East of England, Deprivation Level 3	High body-mass index	Smoking	High blood pressure	High fasting plasma glucose	High FPG (continuous)	Alcohol use	Low glomerular filtration	High total cholesterol	Low physical activity	Drug use
East of England, Deprivation Level 4	High body-mass index	Smoking	High blood pressure	High fasting plasma glucose	High FPG (continuous)	Alcohol use	Low glomerular filtration	High total cholesterol	Low physical activity	Low fruit
East of England, Deprivation Level 5	High body-mass index	Smoking	High blood pressure	High fasting plasma glucose	High FPG (continuous)	Alcohol use	Low glomerular filtration	High total cholesterol	Low physical activity	Iron deficiency
**Greater London**	Smoking	High body-mass index	High blood pressure	High fasting plasma glucose	Alcohol use	High FPG (continuous)	Low glomerular filtration	High total cholesterol	Drug use	Low physical activity
Greater London, Deprivation Level 1	Smoking	High body-mass index	High blood pressure	High fasting plasma glucose	Alcohol use	High FPG (continuous)	High total cholesterol	Low glomerular filtration	Drug use	Low physical activity
Greater London, Deprivation Level 2	Smoking	High body-mass index	High blood pressure	High fasting plasma glucose	Alcohol use	High FPG (continuous)	High total cholesterol	Low glomerular filtration	Drug use	Low physical activity
Greater London, Deprivation Level 3	Smoking	High body-mass index	High blood pressure	High fasting plasma glucose	High FPG (continuous)	Alcohol use	Low glomerular filtration	High total cholesterol	Drug use	Low physical activity
Greater London, Deprivation Level 4	High body-mass index	Smoking	High blood pressure	High fasting plasma glucose	Alcohol use	High FPG (continuous)	Low glomerular filtration	High total cholesterol	Low physical activity	Low fruit
Greater London, Deprivation Level 5	High body-mass index	Smoking	High blood pressure	Alcohol use	High fasting plasma glucose	Low glomerular filtration	High FPG (continuous)	High total cholesterol	Low physical activity	Low fruit
**North East England**	Smoking	High body-mass index	High blood pressure	Alcohol use	High fasting plasma glucose	High FPG (continuous)	High total cholesterol	Low glomerular filtration	Low fruit	Low physical activity
North East England, Deprivation Level 1	Smoking	High body-mass index	High blood pressure	Alcohol use	High fasting plasma glucose	High total cholesterol	High FPG (continuous)	Drug use	Low glomerular filtration	Low fruit
North East England, Deprivation Level 2	Smoking	High body-mass index	High blood pressure	Alcohol use	High fasting plasma glucose	High FPG (continuous)	High total cholesterol	Low glomerular filtration	Low fruit	Low physical activity
North East England, Deprivation Level 3	Smoking	High body-mass index	High blood pressure	High fasting plasma glucose	Alcohol use	High FPG (continuous)	High total cholesterol	Low glomerular filtration	Low fruit	Low physical activity
North East England, Deprivation Level 4	Smoking	High body-mass index	High blood pressure	High fasting plasma glucose	High FPG (continuous)	Alcohol use	Low glomerular filtration	High total cholesterol	Low fruit	Low physical activity
North East England, Deprivation Level 5	High body-mass index	Smoking	High blood pressure	Alcohol use	High fasting plasma glucose	Low glomerular filtration	High FPG (continuous)	High total cholesterol	Low fruit	Low physical activity
**North West England**	Smoking	High body-mass index	High blood pressure	Alcohol use	High fasting plasma glucose	High FPG (continuous)	High total cholesterol	Low glomerular filtration	Drug use	Low fruit
North West England, Deprivation Level 1	Smoking	High body-mass index	High blood pressure	Alcohol use	High fasting plasma glucose	High total cholesterol	High FPG (continuous)	Drug use	Low glomerular filtration	Low fruit
North West England, Deprivation Level 2	Smoking	High body-mass index	High blood pressure	Alcohol use	High fasting plasma glucose	High FPG (continuous)	High total cholesterol	Low glomerular filtration	Drug use	Low fruit
North West England, Deprivation Level 3	Smoking	High body-mass index	High blood pressure	Alcohol use	High fasting plasma glucose	High FPG (continuous)	Low glomerular filtration	High total cholesterol	Low fruit	Low physical activity
North West England, Deprivation Level 4	Smoking	High body-mass index	High blood pressure	Alcohol use	High fasting plasma glucose	High FPG (continuous)	Low glomerular filtration	High total cholesterol	Low fruit	Low physical activity
North West England, Deprivation Level 5	High body-mass index	Smoking	High blood pressure	Alcohol use	High fasting plasma glucose	High FPG (continuous)	Low glomerular filtration	High total cholesterol	Low fruit	Low physical activity
**South East England**	High body-mass index	Smoking	High blood pressure	High fasting plasma glucose	Alcohol use	High FPG (continuous)	Low glomerular filtration	High total cholesterol	Drug use	Low physical activity
South East England, Deprivation Level 1	Smoking	High body-mass index	High blood pressure	Alcohol use	High fasting plasma glucose	Drug use	High FPG (continuous)	High total cholesterol	Low glomerular filtration	Low physical activity
South East England, Deprivation Level 2	Smoking	High body-mass index	High blood pressure	High fasting plasma glucose	Alcohol use	High FPG (continuous)	Drug use	High total cholesterol	Low glomerular filtration	Low physical activity
South East England, Deprivation Level 3	High body-mass index	Smoking	High blood pressure	High fasting plasma glucose	Alcohol use	High FPG (continuous)	Low glomerular filtration	High total cholesterol	Low physical activity	Drug use
South East England, Deprivation Level 4	High body-mass index	Smoking	High blood pressure	High fasting plasma glucose	Alcohol use	High FPG (continuous)	Low glomerular filtration	High total cholesterol	Low physical activity	Low fruit
South East England, Deprivation Level 5	High body-mass index	Smoking	High blood pressure	High fasting plasma glucose	High FPG (continuous)	Alcohol use	Low glomerular filtration	High total cholesterol	Low physical activity	Iron deficiency
**South West England**	High body-mass index	Smoking	High blood pressure	Alcohol use	High fasting plasma glucose	High FPG (continuous)	Low glomerular filtration	High total cholesterol	Drug use	Low physical activity
South West England, Deprivation Level 1	Smoking	High body-mass index	High blood pressure	Alcohol use	Drug use	High fasting plasma glucose	High FPG (continuous)	High total cholesterol	Low glomerular filtration	Low physical activity
South West England, Deprivation Level 2	Smoking	High body-mass index	High blood pressure	Alcohol use	High fasting plasma glucose	High FPG (continuous)	High total cholesterol	Drug use	Low glomerular filtration	Low physical activity
South West England, Deprivation Level 3	High body-mass index	Smoking	High blood pressure	Alcohol use	High fasting plasma glucose	High FPG (continuous)	Low glomerular filtration	High total cholesterol	Low physical activity	Drug use
South West England, Deprivation Level 4	High body-mass index	Smoking	High blood pressure	Alcohol use	High fasting plasma glucose	High FPG (continuous)	Low glomerular filtration	High total cholesterol	Low physical activity	Low fruit
South West England, Deprivation Level 5	High body-mass index	Smoking	High blood pressure	High fasting plasma glucose	Alcohol use	High FPG (continuous)	Low glomerular filtration	High total cholesterol	Low physical activity	Iron deficiency
**West Midlands**	High body-mass index	Smoking	High blood pressure	High fasting plasma glucose	Alcohol use	High FPG (continuous)	High total cholesterol	Low glomerular filtration	Low physical activity	Low fruit
West Midlands, Deprivation Level 1	Smoking	High body-mass index	High blood pressure	High fasting plasma glucose	Alcohol use	High FPG (continuous)	High total cholesterol	Low glomerular filtration	Low physical activity	Drug use
West Midlands, Deprivation Level 2	Smoking	High body-mass index	High blood pressure	High fasting plasma glucose	Alcohol use	High FPG (continuous)	High total cholesterol	Low glomerular filtration	Low physical activity	Drug use
West Midlands, Deprivation Level 3	High body-mass index	Smoking	High blood pressure	High fasting plasma glucose	Alcohol use	High FPG (continuous)	High total cholesterol	Low glomerular filtration	Low fruit	Low physical activity
West Midlands, Deprivation Level 4	High body-mass index	Smoking	High blood pressure	High fasting plasma glucose	Alcohol use	High FPG (continuous)	Low glomerular filtration	High total cholesterol	Low fruit	Low physical activity
West Midlands, Deprivation Level 5	High body-mass index	Smoking	High blood pressure	High fasting plasma glucose	Alcohol use	High FPG (continuous)	Low glomerular filtration	High total cholesterol	Low physical activity	Low fruit
**Yorkshire and the Humber**	Smoking	High body-mass index	High blood pressure	High fasting plasma glucose	Alcohol use	High FPG (continuous)	High total cholesterol	Low glomerular filtration	Low physical activity	Low fruit
Yorkshire and the Humber, Deprivation Level 1	Smoking	High body-mass index	High blood pressure	Alcohol use	High fasting plasma glucose	High total cholesterol	High FPG (continuous)	Drug use	Low glomerular filtration	Low physical activity
Yorkshire and the Humber, Deprivation Level 2	Smoking	High body-mass index	High blood pressure	High fasting plasma glucose	Alcohol use	High FPG (continuous)	High total cholesterol	Low glomerular filtration	Low physical activity	Low fruit
Yorkshire and the Humber, Deprivation Level 3	Smoking	High body-mass index	High blood pressure	Alcohol use	High fasting plasma glucose	High FPG (continuous)	High total cholesterol	Low glomerular filtration	Low fruit	Low physical activity
Yorkshire and the Humber, Deprivation Level 4	High body-mass index	Smoking	High blood pressure	High fasting plasma glucose	High FPG (continuous)	Alcohol use	Low glomerular filtration	High total cholesterol	Low physical activity	Low fruit
Yorkshire and the Humber, Deprivation Level 5	High body-mass index	Smoking	High blood pressure	High fasting plasma glucose	Alcohol use	High FPG (continuous)	Low glomerular filtration	High total cholesterol	Low fruit	Low physical activity

Legend

1	2	3	4	5	6	7	8	9	10
Smoking	High body-mass index	High blood pressure	High fasting plasma glucose	Alcohol use	High FPG (continuous)	High total cholesterol	Low glomerular filtration	Drug use	Low physical activity
11	12	13	14	15					
Low fruit	Iron deficiency	High processed meat	Low vegetables	Low whole grains					

Web Table 1 : Percentage by the Indices of Deprivation 2010 (ID2010) deprivation quintile, for regions in England, 2013					
Region	Deprivation Quintile				
	1 (most deprived)	2	3	4	5 (least deprived)
**North East England**	*32.5*	*23.4*	*15.6*	*15.0*	*13.4*
**North West England**	*32.8*	*18.9*	*16.6*	*16.8*	*14.9*
**Yorkshire & The Humber**	*27.8*	*19.8*	*18.1*	*19.6*	*14.7*
**East Midlands**	*17.0*	*19.5*	*19.9*	*21.1*	*22.4*
**West Midlands**	*28.9*	*18.8*	*20.1*	*18.2*	*13.9*
**East of England**	*7.7*	*16.7*	*21.7*	*25.3*	*28.6*
**Greater London**	*27.7*	*30.5*	*19.7*	*14.2*	*7.9*
**South East England**	*7.3*	*15.1*	*20.2*	*22.6*	*34.8*
**South West England**	*9.1*	*19.1*	*26.6*	*24.5*	*20.7*
Source: Public Health England, Office for National Statistics mid year population estimates					

Web Table 2 **: Citation List for England**

Citation	Year*	New for GBD 2013*
WHO Regional Office for Europe (EURO-WHO). European Hospital Morbidity Database. Copenhagen, Denmark: WHO Regional Office for Europ (EURO-WHO).	2000-2010	
United Kingdom General Household Survey 2002-2003 as it appears in P.N. Lee Statistics and Computing. International Mortality and Smokir Statistics.	2002-2003	
Centre for Research on the Epidemiology of Disasters (CRED). EM-DAT: The OFDA/CRED International Disaster Database. Brussels, Belgiun Catholic University of Leuven.	1952, 1960, 1966, 1968, 1977, 1981, 1983-1984, 1987, 1989-1991, 1993-2000, 2002, 2004-2005, 2007-2009, 2012	
United Kingdom Vital Registration - Deaths 1950 ICD7 as it appears in World Health Organization (WHO). WHO Mortality Database Versio February 2014. Geneva, Switzerland: World Health Organization (WHO), 2014.	1950	
United Kingdom Vital Registration - Deaths 1951 ICD7 as it appears in World Health Organization (WHO). WHO Mortality Database Versio February 2014. Geneva, Switzerland: World Health Organization (WHO), 2014.	1951	
United Kingdom Vital Registration - Deaths 1952 ICD7 as it appears in World Health Organization (WHO). WHO Mortality Database Versio February 2014. Geneva, Switzerland: World Health Organization (WHO), 2014.	1952	
United Kingdom Vital Registration - Deaths 1953 ICD7 as it appears in World Health Organization (WHO). WHO Mortality Database Versio February 2014. Geneva, Switzerland: World Health Organization (WHO), 2014.	1953	
United Kingdom Vital Registration - Deaths 1954 ICD7 as it appears in World Health Organization (WHO). WHO Mortality Database Versio February 2014. Geneva, Switzerland: World Health Organization (WHO), 2014.	1954	
United Kingdom Vital Registration - Deaths 1955 ICD7 as it appears in World Health Organization (WHO). WHO Mortality Database Versio February 2014. Geneva, Switzerland: World Health Organization (WHO), 2014.	1955	*
United Kingdom Vital Registration - Deaths 1956 ICD7 as it appears in World Health Organization (WHO). WHO Mortality Database Versio February 2014. Geneva, Switzerland: World Health Organization (WHO), 2014.	1956	
United Kingdom Vital Registration - Deaths 1957 ICD7 as it appears in World Health Organization (WHO). WHO Mortality Database Versio February 2014. Geneva, Switzerland: World Health Organization (WHO), 2014.	1957	
United Kingdom Vital Registration - Deaths 1958 ICD7 as it appears in World Health Organization (WHO). WHO Mortality Database Versio February 2014. Geneva, Switzerland: World Health Organization (WHO), 2014.	1958	
United Kingdom Vital Registration - Deaths 1959 ICD7 as it appears in World Health Organization (WHO). WHO Mortality Database Versio February 2014. Geneva, Switzerland: World Health Organization (WHO), 2014.	1959	
United Kingdom Vital Registration - Deaths 1960 ICD7 as it appears in World Health Organization (WHO). WHO Mortality Database Versio February 2014. Geneva, Switzerland: World Health Organization (WHO), 2014.	1960	
United Kingdom Vital Registration - Deaths 1961 ICD7 as it appears in World Health Organization (WHO). WHO Mortality Database Versio February 2014. Geneva, Switzerland: World Health Organization (WHO), 2014.	1961	*
United Kingdom Vital Registration - Deaths 1962 ICD7 as it appears in World Health Organization (WHO). WHO Mortality Database Versio February 2014. Geneva, Switzerland: World Health Organization (WHO), 2014.	1962	*
United Kingdom Vital Registration - Deaths 1963 ICD7 as it appears in World Health Organization (WHO). WHO Mortality Database Versio February 2014. Geneva, Switzerland: World Health Organization (WHO), 2014.	1963	
United Kingdom Vital Registration - Deaths 1964 ICD7 as it appears in World Health Organization (WHO). WHO Mortality Database Versio February 2014. Geneva, Switzerland: World Health Organization (WHO), 2014.	1964	*
United Kingdom Vital Registration - Deaths 1965 ICD7 as it appears in World Health Organization (WHO). WHO Mortality Database Versio February 2014. Geneva, Switzerland: World Health Organization (WHO), 2014.	1965	
United Kingdom Vital Registration - Deaths 1966 ICD7 as it appears in World Health Organization (WHO). WHO Mortality Database Versio February 2014. Geneva, Switzerland: World Health Organization (WHO), 2014.	1966	
United Kingdom Vital Registration - Deaths 1967 ICD7 as it appears in World Health Organization (WHO). WHO Mortality Database Versio February 2014. Geneva, Switzerland: World Health Organization (WHO), 2014.	1967	
United Kingdom Vital Registration - Deaths 1968 ICD8 as it appears in World Health Organization (WHO). WHO Mortality Database Versio February 2014. Geneva, Switzerland: World Health Organization (WHO), 2014.	1968	
United Kingdom Vital Registration - Deaths 1969 ICD8 as it appears in World Health Organization (WHO). WHO Mortality Database Versio February 2014. Geneva, Switzerland: World Health Organization (WHO), 2014.	1969	
United Kingdom Vital Registration - Deaths 1970 ICD8 as it appears in World Health Organization (WHO). WHO Mortality Database Versio February 2014. Geneva, Switzerland: World Health Organization (WHO), 2014.	1970	
United Kingdom Vital Registration - Deaths 1971 ICD8 as it appears in World Health Organization (WHO). WHO Mortality Database Versio February 2014. Geneva, Switzerland: World Health Organization (WHO), 2014.	1971	
United Kingdom Vital Registration - Deaths 1972 ICD8 as it appears in World Health Organization (WHO). WHO Mortality Database Versio February 2014. Geneva, Switzerland: World Health Organization (WHO), 2014.	1972	
United Kingdom Vital Registration - Deaths 1973 ICD8 as it appears in World Health Organization (WHO). WHO Mortality Database Versio February 2014. Geneva, Switzerland: World Health Organization (WHO), 2014.	1973	
United Kingdom Vital Registration - Deaths 1974 ICD8 as it appears in World Health Organization (WHO). WHO Mortality Database Versio February 2014. Geneva, Switzerland: World Health Organization (WHO), 2014.	1974	
United Kingdom Vital Registration - Deaths 1975 ICD8 as it appears in World Health Organization (WHO). WHO Mortality Database Versio February 2014. Geneva, Switzerland: World Health Organization (WHO), 2014.	1975	
United Kingdom Vital Registration - Deaths 1976 ICD8 as it appears in World Health Organization (WHO). WHO Mortality Database Versio February 2014. Geneva, Switzerland: World Health Organization (WHO), 2014.	1976	
United Kingdom Vital Registration - Deaths 1977 ICD8 as it appears in World Health Organization (WHO). WHO Mortality Database Versio February 2014. Geneva, Switzerland: World Health Organization (WHO), 2014.	1977	
United Kingdom Vital Registration - Deaths 1978 ICD8 as it appears in World Health Organization (WHO). WHO Mortality Database Versio February 2014. Geneva, Switzerland: World Health Organization (WHO), 2014.	1978	
United Kingdom Vital Registration - Deaths 1979 ICD9 as it appears in World Health Organization (WHO). WHO Mortality Database Versio February 2014. Geneva, Switzerland: World Health Organization (WHO), 2014.	1979	
United Kingdom Vital Registration - Deaths 1980 ICD9 as it appears in World Health Organization (WHO). WHO Mortality Database Versio February 2014. Geneva, Switzerland: World Health Organization (WHO), 2014.	1980	*
United Kingdom Vital Registration - Deaths 1981 ICD9 as it appears in World Health Organization (WHO). WHO Mortality Database Versio February 2014. Geneva, Switzerland: World Health Organization (WHO), 2014.	1981	*
United Kingdom Vital Registration - Deaths 1982 ICD9 as it appears in World Health Organization (WHO). WHO Mortality Database Versio February 2014. Geneva, Switzerland: World Health Organization (WHO), 2014.	1982	
United Kingdom Vital Registration - Deaths 1983 ICD9 as it appears in World Health Organization (WHO). WHO Mortality Database Versio February 2014. Geneva, Switzerland: World Health Organization (WHO), 2014.	1983	
United Kingdom Vital Registration - Deaths 1984 ICD9 as it appears in World Health Organization (WHO). WHO Mortality Database Versio February 2014. Geneva, Switzerland: World Health Organization (WHO), 2014.	1984	*
United Kingdom Vital Registration - Deaths 1985 ICD9 as it appears in World Health Organization (WHO). WHO Mortality Database Versio February 2014. Geneva, Switzerland: World Health Organization (WHO), 2014.	1985	
United Kingdom Vital Registration - Deaths 1986 ICD9 as it appears in World Health Organization (WHO). WHO Mortality Database Versio February 2014. Geneva, Switzerland: World Health Organization (WHO), 2014.	1986	
United Kingdom Vital Registration - Deaths 1987 ICD9 as it appears in World Health Organization (WHO). WHO Mortality Database Versio February 2014. Geneva, Switzerland: World Health Organization (WHO), 2014.	1987	
United Kingdom Vital Registration - Deaths 1988 ICD9 as it appears in World Health Organization (WHO). WHO Mortality Database Versio February 2014. Geneva, Switzerland: World Health Organization (WHO), 2014.	1988	
United Kingdom Vital Registration - Deaths 1989 ICD9 as it appears in World Health Organization (WHO). WHO Mortality Database Versio February 2014. Geneva, Switzerland: World Health Organization (WHO), 2014.	1989	
United Kingdom Vital Registration - Deaths 1990 ICD9 as it appears in World Health Organization (WHO). WHO Mortality Database Versio February 2014. Geneva, Switzerland: World Health Organization (WHO), 2014.	1990	*
United Kingdom Vital Registration - Deaths 1991 ICD9 as it appears in World Health Organization (WHO). WHO Mortality Database Versio February 2014. Geneva, Switzerland: World Health Organization (WHO), 2014.	1991	
United Kingdom Vital Registration - Deaths 1992 ICD9 as it appears in World Health Organization (WHO). WHO Mortality Database Versio February 2014. Geneva, Switzerland: World Health Organization (WHO), 2014.	1992	
United Kingdom Vital Registration - Deaths 1993 ICD9 as it appears in World Health Organization (WHO). WHO Mortality Database Versio February 2014. Geneva, Switzerland: World Health Organization (WHO), 2014.	1993	
United Kingdom Vital Registration - Deaths 1994 ICD9 as it appears in World Health Organization (WHO). WHO Mortality Database Versio February 2014. Geneva, Switzerland: World Health Organization (WHO), 2014.	1994	*
United Kingdom Vital Registration - Deaths 1995 ICD9 as it appears in World Health Organization (WHO). WHO Mortality Database Versio February 2014. Geneva, Switzerland: World Health Organization (WHO), 2014.	1995	
United Kingdom Vital Registration - Deaths 1996 ICD9 as it appears in World Health Organization (WHO). WHO Mortality Database Versio February 2014. Geneva, Switzerland: World Health Organization (WHO), 2014.	1996	

United Kingdom Vital Registration - Deaths 1997 ICD9 as it appears in World Health Organization (WHO). WHO Mortality Database Versio February 2014. Geneva, Switzerland: World Health Organization (WHO), 2014.	1997	*
United Kingdom Vital Registration - Deaths 1998 ICD9 as it appears in World Health Organization (WHO). WHO Mortality Database Versio February 2014. Geneva, Switzerland: World Health Organization (WHO), 2014.	1998	
United Kingdom Vital Registration - Deaths 1999 ICD9 as it appears in World Health Organization (WHO). WHO Mortality Database Versio February 2014. Geneva, Switzerland: World Health Organization (WHO), 2014.	1999	
United Kingdom Vital Registration - Deaths 2000 ICD9 as it appears in World Health Organization (WHO). WHO Mortality Database Versio February 2014. Geneva, Switzerland: World Health Organization (WHO), 2014.		
United Kingdom Vital Registration - Deaths 2001 ICD10 as it appears in World Health Organization (WHO). WHO Mortality Database Versio February 2014. Geneva, Switzerland: World Health Organization (WHO), 2014.	2001	
United Kingdom Vital Registration - Deaths 2002 ICD10 as it appears in World Health Organization (WHO). WHO Mortality Database Versio February 2014. Geneva, Switzerland: World Health Organization (WHO), 2014.	2002	
United Kingdom Vital Registration - Deaths 2003 ICD10 as it appears in World Health Organization (WHO). WHO Mortality Database Versio February 2014. Geneva, Switzerland: World Health Organization (WHO), 2014.	2003	
United Kingdom Vital Registration - Deaths 2004 ICD10 as it appears in World Health Organization (WHO). WHO Mortality Database Versio February 2014. Geneva, Switzerland: World Health Organization (WHO), 2014.	2004	*
United Kingdom Vital Registration - Deaths 2005 ICD10 as it appears in World Health Organization (WHO). WHO Mortality Database Versio February 2014. Geneva, Switzerland: World Health Organization (WHO), 2014.	2005	
United Kingdom Vital Registration - Deaths 2006 ICD10 as it appears in World Health Organization (WHO). WHO Mortality Database Versio February 2014. Geneva, Switzerland: World Health Organization (WHO), 2014.	2006	
United Kingdom Vital Registration - Deaths 2007 ICD10 as it appears in World Health Organization (WHO). WHO Mortality Database Versio February 2014. Geneva, Switzerland: World Health Organization (WHO), 2014.	2007	
Max Planck Institute for Demographic Research, University of California. Human Mortality Database. Berkeley, USA: University of Californ Rostock, Germany: Max Planck Institute for Demographic Research.	1950-2000	
World Health Organization (WHO). United Kingdom World Health Survey 2004. Geneva, Switzerland: World Health Organization (WHO), 2005.	2004	
United Kingdom General Household Survey 1998-1999 as it appears in P.N. Lee Statistics and Computing. International Mortality and Smokir Statistics.	1998-1999	
Office of Population Censuses and Surveys. Social Survey Division, Health Survey for England, 1991-1992: Combined Data File. Colchester, Esse UK Data Archive [distributor], October 1997. SN: 3238, http://dx.doi.org/10.5255/UKDA-SN-3238-1	1991-1992	
Office of Population Censuses and Surveys. Social Survey Division, Health Survey for England, 1993 [Computer file]. Colchester, Essex: UK Da Archive [distributor], April 1995. SN: 3316, http://dx.doi.org/10.5255/UKDA-SN-3316-1	1993	
Joint Health Surveys Unit of Social and Community Planning Research and University College London, Health Survey for England, 1994 [compu file]. 4th ed. Colchester, Essex: UK Data Archive [distributor], 26 March 2001. SN: 3640.	1994-1995	
Joint Health Surveys Unit of Social and Community Planning Research and University College London, Health Survey for England, 1995 [compu file]. 3rd ed. Colchester, Essex: UK Data Archive [distributor], 26 March 2001. SN: 3796.	1995-1996	
Joint Health Surveys Unit of Social and Community Planning Research and University College London, Health Survey for England, 1996 [compu file]. 3rd ed. Colchester, Essex: UK Data Archive [distributor], March 2001. SN: 3886.	1996-1997	
Joint Health Surveys Unit of Social and Community Planning Research and University College London, Health Survey for England, 1997 [compu file]. 2nd ed. Colchester, Essex: UK Data Archive [distributor], 4 December 2000. SN: 3979.	1997-1998	
National Centre for Social Research, University College London Department of Epidemiology and Public Health, Health Survey for England, 19 [computer file]. 4th ed. Colchester, Essex: UK Data Archive [distributor], 30 November 2002. SN: 4150.	1998-1999	
National Centre for Social Research and University College London. Department of Epidemiology and Public Health, Health Survey for England, 1999 [computer file]. 3rd Edition. Colchester, Essex: UK Data Archive [distributor], February 2002. SN: 4365.	1999-2000	*
National Centre for Social Research, University College London Department of Epidemiology and Public Health, Health Survey for England, 20 [computer file]. Colchester, Essex: UK Data Archive [distributor], 23 April 2002. SN: 4487.	2000-2001	
National Centre for Social Research and University College London. Department of Epidemiology and Public Health, Health Survey for England, 20 [computer file]. 2nd Edition. Colchester, Essex: UK Data Archive [distributor], June 2004. SN: 4628.	2001-2002	
National Centre for Social Research and University College London. Department of Epidemiology and Public Health, Health Survey for England, 20 [computer file]. Colchester, Essex: UK Data Archive [distributor], May 2004. SN: 4912.	2002-2003	
National Centre for Social Research and University College London. Department of Epidemiology and Public Health, Health Survey for England, 20 [computer file]. Colchester, Essex: UK Data Archive [distributor], March 2005. SN: 5098.	2003-2004	
National Centre for Social Research and University College London. Department of Epidemiology and Public Health, Health Survey for England, 20 [computer file]. Colchester, Essex: UK Data Archive [distributor], July 2006. SN: 5439.	2004-2005	*
National Centre for Social Research and University College London. Department of Epidemiology and Public Health, Health Survey for England, 20 [computer file]. Colchester, Essex: UK Data Archive [distributor], July 2007. SN: 5675.	2005-2006	
[illegible] [computer file]. 4th Edition. Colchester, Essex: UK Data Archive [distributor], July 2011. SN: 5809, http://dx.doi.org/10.5255/UKDA-SN-5809-1	2006-2007	
Office of Population Censuses and Surveys (United Kingdom), Minnesota Population Center. United Kingdom Census 1991 from the Integrated Public Use Microdata Series, International: [Machine-readable database]. Minneapolis: University of Minnesota.	1991	
United Kingdom Vital Registration - Deaths 2009 ICD10 as it appears in World Health Organization (WHO). WHO Mortality Database Versio February 2014. Geneva, Switzerland: World Health Organization (WHO), 2014.	2009	
United Kingdom Vital Registration - Deaths 2008 ICD10 as it appears in World Health Organization (WHO). WHO Mortality Database Versio February 2014. Geneva, Switzerland: World Health Organization (WHO), 2014.	2008	*
Institute for Social and Economic Research, University of Essex, Gfk NOP, Office for National Statistics (ONS) (United Kingdom), Economic and Social Research Council (ESRC). United Kingdom British Household Panel Survey 1992-1993. Essex, United Kingdom: Institute for Social and Economic Research, University of Essex.	1992-1993	
Institute for Social and Economic Research, University of Essex, Gfk NOP, Office for National Statistics (ONS) (United Kingdom), Economic and Social Research Council (ESRC). United Kingdom British Household Panel Survey 1993-1994. Essex, United Kingdom: Institute for Social and Economic Research, University of Essex.	1993-1994	
Institute for Social and Economic Research, University of Essex, Gfk NOP, Office for National Statistics (ONS) (United Kingdom), Economic and Social Research Council (ESRC), Health Education Authority (HEA) (United Kingdom). United Kingdom British Household Panel Survey 1994-1995. Essex, United Kingdom: Institute for Social and Economic Research, University of Essex.	1994-1995	
Institute for Social and Economic Research, University of Essex, Gfk NOP, Office for National Statistics (ONS) (United Kingdom), Economic and Social Research Council (ESRC), Health Education Authority (HEA) (United Kingdom). United Kingdom British Household Panel Survey 1995-1996. Essex, United Kingdom: Institute for Social and Economic Research, University of Essex.	1995-1996	
Institute for Social and Economic Research, University of Essex, Gfk NOP, Office for National Statistics (ONS) (United Kingdom), Economic and Social Research Council (ESRC), Health Education Authority (HEA) (United Kingdom). United Kingdom British Household Panel Survey 1996-1997. Essex, United Kingdom: Institute for Social and Economic Research, University of Essex.	1996-1997	
Institute for Social and Economic Research, University of Essex, Gfk NOP, Office for National Statistics (ONS) (United Kingdom), Economic and Social Research Council (ESRC), Health Education Authority (HEA) (United Kingdom). United Kingdom British Household Panel Survey 1997-1998. Essex, United Kingdom: Institute for Social and Economic Research, University of Essex.	1997-1998	
Institute for Social and Economic Research, University of Essex, Gfk NOP, Office for National Statistics (ONS) (United Kingdom), Economic and Social Research Council (ESRC), Health Education Authority (HEA) (United Kingdom). United Kingdom British Household Panel Survey 1998-1999. Essex, United Kingdom: Institute for Social and Economic Research, University of Essex.	1998-1999	
Institute for Social and Economic Research, University of Essex, Gfk NOP, Office for National Statistics (ONS) (United Kingdom), Economic and Social Research Council (ESRC), Health Education Authority (HEA) (United Kingdom). United Kingdom British Household Panel Survey 1999-2000. Essex, United Kingdom: Institute for Social and Economic Research, University of Essex.	1999-2000	
Institute for Social and Economic Research, University of Essex, Gfk NOP, Office for National Statistics (ONS) (United Kingdom), Economic and Social Research Council (ESRC), Health Development Agency (United Kingdom). United Kingdom British Household Panel Survey 2000-2001. Essex, United Kingdom: Institute for Social and Economic Research, University of Essex.	2000-2001	
Food and Agriculture Organization of the United Nations (FAO). United Kingdom FAOSTAT Food Balance Sheets. Rome, Italy: Food and Agricultu Organization of the United Nations (FAO).	1990, 1995, 2000, 2005, 2009	
United Nations Children's Fund (UNICEF), World Health Organization (WHO). WHO and UNICEF Reported Disease Incidence Time Series Pertussis. Geneva, Switzerland: World Health Organization (WHO).	1980-1998, 2000-2010	

Royal College of General Practitioners. United Kingdom - England and Wales Weekly Returns Service Annual Report 2009. London, United Kingdo Royal College of General Practitioners.	2000	
Royal College of General Practitioners. United Kingdom - England and Wales Weekly Returns Service Annual Report 2005. London, United Kingdo Royal College of General Practitioners.	2005	
United Nations Office on Drugs and Crime (UNODC). United Nations Surveys on Crime Trends and the Operations of Criminal Justice Systems 197 2006 as provided by Kavi Bhalla. [Unpublished].	1981-1994	
World Health Organization (WHO). Global Status Report on Road Safety 2009. Geneva, Switzerland: World Health Organization (WHO), 2009.	2007	
United Kingdom Vital Registration - Deaths 2010 ICD10 as it appears in World Health Organization (WHO). WHO Mortality Database Versio February 2014. Geneva, Switzerland: World Health Organization (WHO), 2014.	2010	*
National Centre for Social Research and University College London. Department of Epidemiology and Public Health, Health Survey for England, 2007 [computer file]. 2nd Edition. Colchester, Essex: UK Data Archive [distributor], April 2010. SN: 6112, http://dx.doi.org/10.5255/UKDA-SN-6112-1	2007-2008	
National Centre for Social Research and University College London. Department of Epidemiology and Public Health, Health Survey for England, 2008 [computer file]. 3rd Edition. Colchester, Essex: UK Data Archive [distributor], July 2011. SN: 6397, http://dx.doi.org/10.5255/UKDA-SN-6397-1	2008-2009	
National Centre for Social Research and University College London. Department of Epidemiology and Public Health, Health Survey for England, 2009 [computer file]. 2nd Edition. Colchester, Essex: UK Data Archive [distributor], July 2011. SN: 6732, http://dx.doi.org/10.5255/UKDA-SN-6732-1	2009-2010	*
NatCen Social Research and Royal Free and University College Medical School. Department of Epidemiology and Public Health, Health Survey for England, 2010 [computer file]. 2nd Edition. Colchester, Essex: UK Data Archive [distributor], July 2012. SN: 6986, http://dx.doi.org/10.5255/UKDA-SN-6986-2	2010	*
NatCen Social Research and University College London. Department of Epidemiology and Public Health, Health Survey for England, 2011 [computer file]. Colchester, Essex: UK Data Archive [distributor], April 2013. SN: 7260, http://dx.doi.org/10.5255/UKDA-SN-7260-1	2011-2012	*
Stewart S, Hart CL, Hole DJ, McMurray JJ. A population-based study of the long-term risks associated with atrial fibrillation: 20-year follow-up of t Renfrew/Paisley study. Am J Med. 2002; 113(5): 359-64.	1980-1996	
Lip GY, Golding DJ, Nazir M, Beevers DG, Child DL, Fletcher RI. A survey of atrial fibrillation in general practice: the West Birmingham Atri Fibrillation Project. Br J Gen Pract. 1997; 47(418): 285-9.	1995	
Lip GY, Bawden L, Hodson R, Rutland E, Snatchfold J, Beevers DG. Atrial fibrillation amongst the Indo-Asian general practice population: The We Birmingham Atrial Fibrillation Project. Int J Cardiol. 1998; 65(2): 187-92.	1996	
O'Connell JE, Gray CS. Atrial Fibrillation and Stroke Prevention in the Community. Age Ageing. 1996; 25(4): 307 -30	1995	
Rietbrock S, Heeley E, Plumb J, van Staa T. Chronic atrial fibrillation: Incidence, prevalence, and prediction of stroke using the Congestive heart failure, Hypertension, Age >75, Diabetes mellitus, and prior Stroke or transient ischemic attack (CHADS2) risk stratification scheme. Am Heart J. 2008; 156(1): 57-64.	1987-2005	
Carroll K, Majeed A. Comorbidity associated with atrial fibrillation: a general practice-based study. Br J Gen Pract. 2001; 51(472): 884-91.	1999	
Sudlow M, Thomson R, Thwaites B, Rodgers H, Kenny RA. Prevalence of atrial fibrillation and eligibility for anticoagulants in the community. Lanc 1998; 352(9135): 1167-71.	1996-1997	
De Lusignan S, Van Vlymen J, Hague N, Thana L, Dzregah B, Chan T. Preventing stroke in people with atrial fibrillation: a cross-sectional study Public Health (Oxf). 2005; 27(1): 85 -92.	2004	
Hill JD, Mottram EM, Killeen PD. Study of the prevalence of atrial fibrillation in general practice patients over 65 years of age. J R Coll Gen Pra 1987; 37(297): 172-3.	1983-1984	
Majeed A, Moser K, Carroll K. Trends in the prevalence and management of atrial fibrillation in general practice in England and Wales, 1994-199 analysis of data from the general practice research database. Heart. 2001; 86(3): 284 -288.	1994-1998	
DeWilde S, Carey IM, Emmas C, Richards N, Cook DG. Trends in the prevalence of diagnosed atrial fibrillation, its treatment with anticoagulation a predictors of such treatment in UK primary care. Heart. 2006; 92(8): 1064-70.	1994-2003	
Almeida AM, Henthorn JS, Davies SC. Neonatal screening for haemoglobinopathies: the results of a 10-year programme in an English Health Regio Br J Haematol. 2001; 112(1): 32-5.	1990-2000	
Davies SC, Cronin E, Gill M, Greengross P, Hickman M, Normand C. Screening for sickle cell disease and thalassaemia: a systematic review wi supplementary research. Health Technol Assess. 2000; 4(3): 1-99.	1998	
Mann JR. Sickle cell haemoglobinopathies in England. Arch Dis Child. 1981; 56(9): 676-83	1969-1979	
Modell B, Darlison M, Birgens H, Cario H, Faustino P, Giordano PC, Gulbis B, Hopmeier P, Lena-Russo D, Romao L, Theodorsson E. Epidemiolo of haemoglobin disorders in Europe: an overview. Scand J Clin Lab Invest. 2007; 67(1): 39-69.	1988, 2006	
Centre for Health Informatics and Multiprofessional Education, University College London. Modell's Haemoglobinopathologist's Almanac. London, United Kingdom: Centre for Health Informatics and Multiprofessional Education, University College London, 2008.	1988, 2003, 2004-2007	
Streetly A, Clarke M, Downing M, Farrar L, Foo Y, Hall K, Kemp H, Newbold J, Walsh P, Yates J, Henthorn J. Implementation of the newbo screening programme for sickle cell disease in England: results for 2003-2005. J Med Screen. 2008; 15(1): 9-13.	2004-2007	
Streetly A, Latinovic R, Hall K, Henthorn J. Implementation of universal newborn bloodspot screening for sickle cell disease and other clinica significant haemoglobinopathies in England: screening results for 2005-7. J Clin Pathol. 2009; 62(1): 26-30.	2005-2007	
Weatherall D. Sickle Cell and Thalassemias Prevalence Data, Personal Correspondence with David Weatherall. [Unpublished	2009	
Garraway WM, Collins GN, Lee RJ. High prevalence of benign prostatic hypertrophy in the community. Lancet. 1991; 338(8765): 469-71.	1990	
Mair FS, Crowley TS, Bundred PE. Prevalence, aetiology and management of heart failure in general practice. Br J Gen Pract. 1996; 46(403): 77-9.	1994	
Berzon DB. Ear disease in a group general practice A review of world communities. J Laryngol Otol. 1983; 97(9): 817-2	1970-1976	
Browning GG, Gatehouse S. The prevalence of middle ear disease in the adult British population. Clin Otolaryngol Allied Sci. 1992; 17(4): 317-21.	1980-1986	
Stathakis V, Kilkenny M, Marks R. Descriptive epidemiology of acne vulgaris in the community. Australas J Dermatol. 1997; 38(3): 115-23.	1987-1988	
Williams H, Stewart A, Von Mutius E, Cookson W, Anderson HR. Is eczema really on the increase worldwide. J Allergy Clin Immunol. 2008; 121(4 947-954.	1992, 1995-1996, 2001-2002	
United Nations Office on Drugs and Crime (UNODC). United Nations Office on Drugs and Crime Global Study on Homicides 2011. Vienna, Austri United Nations Office on Drugs and Crime (UNODC), 2011.	1998-2010	
Deshpande SA, Northern V. The clinical and health economic burden of respiratory syncytial virus disease among children under 2 years of age i defined geographical area. Arch Dis Child. 2003; 12(88): 1065-9.	1996-1999	
Fox KF, Cowie MR, Wood DA, Coats AJS, Gibbs JSR, Underwood SR, Turner RM, Poole-Wilson PA, Davies SW, Sutton GC. Coronary arte disease as the cause of incident heart failure in the population. Eur Heart J. 2001; 22(3): 228-36.	1998, 2000-2001	
de Giuli F, Khaw KT, Cowie MR, Sutton GC, Ferrari R, Poole-Wilson PA. Incidence and outcome of persons with a clinical diagnosis of heart failure in a general practice population of 696,884 in the United Kingdom. Eur J Heart Fail. 2005; 7(3): 295-302.	1991-1994	
Jones IR, Urwin G, Feldman RA, Banatvala N. Social deprivation and bacterial meningitis in north east Thames region: three year study using sm area statistics. BMJ. 1997; 314(7083): 794-5.	1991-1993	
Forster J, Guarino A, Parez N, Moraga F, Román E, Mory O, Tozzi AE, de Aguileta AL, Wahn U, Graham C, Berner R, Ninan T, Barberousse C, Meyer N, Soriano-Gabarró M. Hospital-based surveillance to estimate the burden of rotavirus gastroenteritis among European children younger than 5 years of age. Pediatrics. 2009; 123(3): e393-400.	2005-2006	
Watson B, Ellis M, Mandal B, Dunbar E, Whale K, Brennand J. A comparison of the clinico-pathological features with stool pathogens in patien hospitalised with the symptom of diarrhoea. J Infect Dis. 1986; 18(6): 553-9.	1981-1982	
Lacey RJ, Lewis M, Sim J. Presentation of pain drawings in questionnaire surveys: influence on prevalence of neck and upper limb pain in t community. Pain. 2003; 105(1-2): 293-301.	2000	
Palmer KT, Walker-Bone K, Griffin MJ, Syddall H, Pannett B, Coggon D, Cooper C. Prevalence and occupational associations of neck pain in t British population. Scand J Work Environ Health. 2001; 27(1): 49-56.	1997-1998	
Sim J, Lacey RJ, Lewis M. The impact of workplace risk factors on the occurrence of neck and upper limb pain: a general population study. BM Public Health. 2006; 6(1): 234.	2001-2002	
Thomas E, Peat G, Harris L, Wilkie R, Croft PR. The prevalence of pain and pain interference in a general population of older adults: cross-section findings from the North Staffordshire Osteoarthritis Project (NorStOP). Pain. 2004; 110(1-2): 361-8.	2001	
Urwin M, Symmons D, Allison T, Brammah T, Busby H, Roxby M, Simmons A, Williams G. Estimating the burden of musculoskeletal disorders in the community: the comparative prevalence of symptoms at different anatomical sites, and the relation to social deprivation. Ann Rheum Dis. 1998; 57(11): 649-55.	1995	
Webb R, Brammah T, Lunt M, Urwin M, Allison T, Symmons D. Prevalence and predictors of intense, chronic, and disabling neck and back pain in t UK general population. Spine. 2003; 28(11): 1195-202.	2000	
Lampe FC, Whincup PH, Wannamethee SG, Shaper AG, Walker M, Ebrahim S. The natural history of prevalent ischaemic heart disease in middle-ag men. Eur Heart J. 2000; 21(13): 1052-62.	1980-1995	

Lampe F, Morris R, Whincup P, Walker M, Ebrahim S, Shaper A. Is the prevalence of coronary heart disease falling in British men? Heart. 200 86(5): 499-505.	1992, 1996	
Murphy NF, Stewart S, Hart CL, MacIntyre K, Hole D, McMurray JJV. A population study of the long-term consequences of Rose angina: 20-ye follow-up of the Renfrew-Paisley study. Heart. 2006; 92(12): 1739-46.	1980-1996	
Douglas KA, Redman CWG. Eclampsia in the United Kingdom. BMJ. 1994; 309(6966): 1395-400	1992	
Telfer P, Coen P, Chakravorty S, Wilkey O, Evans J, Newell H, Smalling B, Amos R, Stephens A, Rogers D, Kirkham F. Clinical outcomes in children with sickle cell disease living in England: a neonatal cohort in East London. Haematologica. 2007; 92(7): 905-12.	1983-2005	
Bath-Hextall F, Leonardi-Bee J, Smith C, Meal A, Hubbard R. Trends in incidence of skin basal cell carcinoma. Additional evidence from a UK prima care database study. Int J Cancer. 2007; 121(9): 2105-8.	1996-2003	
Modell B, Khan M, Darlison M. Survival in beta-thalassaemia major in the UK. Lancet. 2000; 355(9220): 2051-2	1950-1998	
Modell B, Khan M, Darlison M, Westwood MA, Ingram D, Pennell DJ. Improved survival of thalassaemia major in the UK and relation to T2 cardiovascular magnetic resonance. J Cardiovasc Magn Reson. 2008; 10(1): 42.	1960-2003	
World Health Organization (WHO). Yellow Fever Reported Cases, WHO Vaccine-Preventable Diseases: Monitoring System. Geneva, Switzerlanc World Health Organization (WHO).	2000-2003, 2005-2009	
Mikuls TR, Farrar JT, Bilker WB, Fernandes S, Schumacher HR Jr, Saag KG. Gout epidemiology: results from the UK General Practice Researc Database, 1990-1999. Ann Rheum Dis. 2005; 64(2): 267-72.	1990-1999	
Currie WJ. Prevalence and incidence of the diagnosis of gout in Great Britain. Ann Rheum Dis. 1979; 38(2): 101-(	1971-1975	
Cea Soriano L, Rothenbacher D, Choi HK, García Rodríguez LA. Contemporary epidemiology of gout in the UK general population. Arthritis Res The 2011; 13(2): R39.	2000-2007	
Annemans L, Spaepen E, Gaskin M, Bonnemaire M, Malier V, Gilbert T, Nuki G. Gout in the UK and Germany: prevalence, comorbidities ar management in general practice 2000-2005. Ann Rheum Dis. 2008; 67(7): 960-6.	2000-2005	
Harris CM, Lloyd DC, Lewis J. The prevalence and prophylaxis of gout in England. J Clin Epidemiol. 1995; 48(9): 1153-	1993	
Gardner MJ, Power C, Barker DJ, Padday R. The prevalence of gout in three English towns. Int J Epidemiol. 1982; 11(1): 71-:	1979	
Gribbin J, Hubbard RB, Le Jeune I, Smith CJ, West J, Tata LJ. Incidence and mortality of idiopathic pulmonary fibrosis and sarcoidosis in the U Thorax. 2006; 61(11): 980-5.	1991-2003	
Rice-Oxley M, Williams ES, Rees JE. A prevalence survey of multiple sclerosis in Sussex. J Neurol Neurosurg Psychiatry. 1995; 58(1): 27-30.	1991	
Sharpe G, Price SE, Last A, Thompson RJ. Multiple sclerosis in island populations: prevalence in the Bailiwicks of Guernsey and Jersey. J Neur Neurosurg Psychiatry. 1995; 58(1): 22-6.	1991	
Mumford CJ, Fraser MB, Wood NW, Compston DA. Multiple sclerosis in the Cambridge health district of east Anglia. J Neurol Neurosurg Psychiatr 1992; 55(10): 877-82.	1989-1991	
Williams ES, McKeran RO. Prevalence of multiple sclerosis in a south London borough. Br Med J (Clin Res Ed). 1986; 293(6541): 237-9.	1976-1985	
Lockyer MJ. Prevalence of multiple sclerosis in five rural Suffolk practices. BMJ. 1991; 303(6798): 347-8	1988	
Shepherd DI, Summers A. Prevalence of multiple sclerosis in Rochdale. J Neurol Neurosurg Psychiatry. 1996; 61(4): 415-'	1989	
Ford HL, Gerry E, Airey CM, Vail A, Johnson MH, Williams DRR. The prevalence of multiple sclerosis in the Leeds Health Authority. J Neur Neurosurg Psychiatry. 1998; 64(5): 605-10.	1996	
Roberts MH, Martin JP, McLellan DL, McIntosh-Michaelis SA, Spackman AJ. The prevalence of multiple sclerosis in the Southampton and Sou West Hampshire Health Authority. J Neurol Neurosurg Psychiatry. 1991; 54(1): 55-9.	1987	
Worldwide variations in the prevalence of asthma symptoms: the International Study of Asthma and Allergies in Childhood (ISAAC). Eur Respir 1998; 12(2): 315-35.	1993-1995	
Lai CK, Beasley R, Crane J, Foliaki S, Shah J, Weiland S. Global variation in the prevalence and severity of asthma symptoms: phase three of t International Study of Asthma and Allergies in Childhood (ISAAC). Thorax. 2009; 64(6): 476-483.	2002	
Stamatakis E, Primatesta P, Department of Epidemiology and Public Health, University College London, National Centre for Social Research (NatCen). Health Survey for England 2001: Respiratory Symptoms, Atopic Conditions and Lung Function. London, United Kingdom: The Stationery Office, 2003.	2001	
Tesfaye S, Stevens LK, Stephenson JM, Fuller JH, Plater M, Ionescu-Tirgoviste C, Nuber A, Pozza G, Ward JD. Prevalence of diabetic peripheral neuropathy and its relation to glycaemic control and potential risk factors: the EURODIAB IDDM Complications Study. Diabetologia. 1996; 39(11): 1377-84.	1993-1995	
Young MJ, Boulton AJ, MacLeod AF, Williams DR, Sonksen PH. A multicentre study of the prevalence of diabetic peripheral neuropathy in the Unit Kingdom hospital clinic population. Diabetologia. 1993; 36(2): 150-4.	1990	
Sampson MJ, Shepstone L, Greenwood RH, Harvey I, Humphries J, Heyburn PJ, Temple RC, Dole G. An integrated mobile foot and retinal screenii programme for people with Type 2 diabetes managed in primary care. Diabet Med. 2002; 19(1): 74-6.	1997-2001	
Walters DP, Gatling W, Mullee MA, Hill RD. The prevalence of diabetic distal sensory neuropathy in an English community. Diabet Med. 1992; 9(4 349-53.	1989-1991	
World Health Organization (WHO), WHO MONICA Project Investigators 2000. MONICA Population Survey Data Book 1979-1997. Geneva Switzerland: World Health Organization (WHO), 2000.	1984-1995	
United Kingdom Smoking and Drinking Among Adults 2007 as it appears in World Health Organization (WHO). WHO Global Infobase - Tobacco Us Prevalence.	2003-2007	
United Kingdom General Household Survey 1992 as it appears in P.N. Lee Statistics and Computing. International Mortality and Smoking Statistics.	1992	
United Kingdom General Household Survey 1990 as it appears in P.N. Lee Statistics and Computing. International Mortality and Smoking Statistics.	1990	
United Kingdom General Household Survey 1988 as it appears in P.N. Lee Statistics and Computing. International Mortality and Smoking Statistics.	1988	
United Kingdom General Household Survey 1986 as it appears in P.N. Lee Statistics and Computing. International Mortality and Smoking Statistics.	1986	
United Kingdom General Household Survey 1982 as it appears in P.N. Lee Statistics and Computing. International Mortality and Smoking Statistics.	1982	
United Kingdom General Household Survey 1984 as it appears in P.N. Lee Statistics and Computing. International Mortality and Smoking Statistics.	1984	
United Kingdom General Household Survey 2001 as it appears in P.N. Lee Statistics and Computing. International Mortality and Smoking Statistics.	2001	
United Kingdom General Household Survey 2000 as it appears in P.N. Lee Statistics and Computing. International Mortality and Smoking Statistics.	2000	
United Kingdom General Household Survey 1996 as it appears in P.N. Lee Statistics and Computing. International Mortality and Smoking Statistics.	1996	
United Kingdom General Household Survey 1994 as it appears in P.N. Lee Statistics and Computing. International Mortality and Smoking Statistics.	1994	
Edmonds M, Boulton A, Buckenham T, Every N, Foster A, Freeman D, Gadsby R, Gibby O, Knowles A, Pooke M, Tovey F, Unwin N, Wolfe J. Repo of the Diabetic Foot and Amputation Group. Diabet Med. 1996; 13(9 Suppl 4): S27-42.	1993	
Abbott CA, Malik RA, van Ross ER, Kulkarni J, Boulton AJ. Prevalence and characteristics of painful diabetic neuropathy in a large community-bas diabetic population in the U.K. Diabetes Care. 2011; 34(10): 2220-4.	2006-2010	
Rubino A, Rousculp MD, Davis K, Wang J, Girach A. Diagnosed diabetic retinopathy in France, Italy, Spain, and the United Kingdom. Prim Ca Diabetes. 2007; 1(2): 75-80.	2006	
Pannell RS, Fleming DM, Cross KW. The incidence of molluscum contagiosum, scabies and lichen planus. Epidemiol Infect. 2005; 133(6): 985-91.	1994-2003	
DECODE Study Group. Age- and sex-specific prevalences of diabetes and impaired glucose regulation in 13 European cohorts. Diabetes Care. 2003 26(1): 61-9.	1993	
Swerdlow AJ, Jones ME. Mortality during 25 years of follow-up of a cohort with diabetes. Int J Epidemiol. 1996; 25(6): 1250-61.	1966-1992	
Laing SP, Swerdlow AJ, Slater SD, Botha JL, Burden AC, Waugh NR, Smith AW, Hill RD, Bingley PJ, Patterson CC, Qiao Z, Keen H. The British Diabetic Association Cohort Study, I: all-cause mortality in patients with insulin-treated diabetes mellitus. Diabet Med. 1999; 16(6): 459-65.	1972-1997	
Croxson SC, Burden AC, Bodington M, Botha JL. The prevalence of diabetes in elderly people. Diabet Med. 1991; 8(1): 28-3	1987	
Pierce MB, Zaninotto P, Steel N, Mindell J. Undiagnosed diabetes-data from the English longitudinal study of ageing. Diabet Med. 2009; 26(7): 67 85.	2005	
Abu-Arefeh I, Russell G. Prevalence of headache and migraine in schoolchildren. BMJ. 1994; 309(6957): 765-9	1991	
Steiner TJ, Scher AI, Stewart WF, Kolodner K, Liberman J, Lipton RB. The prevalence and disability burden of adult migraine in England and the relationships to age, gender and ethnicity. Cephalalgia. 2003; 23(7): 519-27.	2000	
Royal College of General Practitioners. United Kingdom - England and Wales Weekly Returns Service Annual Prevalence Report 2004. Londo United Kingdom: Royal College of General Practitioners.	2001	

Nevitt GJ, Hutchinson PE. Psoriasis in the community: prevalence, severity and patients' beliefs and attitudes towards the disease. Br J Dermatol. 199 135(4): 533-7.	1995	
Badley EM, Tennant A. Changing profile of joint disorders with age: findings from a postal survey of the population of Calderdale, West Yorkshi United Kingdom. Ann Rheum Dis. 1992; 51(3): 366-71.	1986	
Birrell F, Lunt M, Macfarlane G, Silman A. Association between pain in the hip region and radiographic changes of osteoarthritis: results from population-based study. Rheumatology. 2005; 44(3): 337-41.	2002	
Croft P, Coggon D, Cruddas M, Cooper C. Osteoarthritis of the hip: an occupational disease in farmers. BMJ. 1992; 304(6837): 1269-72.	1989	
Dawson J, Linsell L, Zondervan K, Rose P, Randall T, Carr A, Fitzpatrick R. Epidemiology of hip and knee pain and its impact on overall health stat in older adults. Rheumatology. 2004; 43(4): 497-504.	2002	
Nüesch E, Dieppe P, Reichenbach S, Williams S, Iff S, Jüni P. All cause and disease specific mortality in patients with knee or hip osteoarthriti population based cohort study. BMJ. 2011; 342: d1165.	1994-2008	
Watson DJ, Rhodes T, Guess HA. All-cause mortality and vascular events among patients with rheumatoid arthritis, osteoarthritis, or no arthritis in tl UK General Practice Research Database. J Rheumatol. 2003; 30(6): 1196-202.	1987-2000	
Konstantinou GN, Papadopoulos NG, Tavladaki T, Tsekoura T, Tsilimigaki A, Grattan CEH. Childhood acute urticaria in northern and southern Europ shows a similar epidemiological pattern and significant meteorological influences. Pediatr Allergy Immunol. 2011; 22(1 Pt 1): 36-42.	2005-2007	*
Jarvis D, Newson R, Lotvall J, Hastan D, Tomassen P, Keil T, Gjomarkaj M, Forsberg B, Gunnbjornsdottir M, Minov J, Brozek G, Dahlen SE, Toskala E, Kowalski ML, Olze H, Howarth P, Krämer U, Baelum J, Loureiro C, Kasper L, Bousquet PJ, Bousquet J, Bachert C, Fokkens W, Burney P. Asthma in adults and its association with chronic rhinosinusitis: the GA2LEN survey in Europe. Allergy. 2012; 67(1): 91-8.	2008-2009	*
Shiue I. Associated social factors of prevalent asthma in adults and the very old in the UK. Allergy. 2013; 68(3): 392-	2009-2010	*
Duncan R, Francis RM, Collerton J, Davies K, Jagger C, Kingston A, Kirkwood T, Robinson L, Birrell F. Prevalence of arthritis and joint pain in tl oldest old: findings from the Newcastle 85+ study. Age Ageing. 2011; 40(6): 752-5.	2008-2010	*
Leyland KM, Hart DJ, Javaid MK, Judge A, Kiran A, Soni A, Goulston LM, Cooper C, Spector TD, Arden NK. The natural history of radiographic kn osteoarthritis: a fourteen-year population-based cohort study. Arthritis Rheum. 2012; 64(7): 2243-51.	1990-2004	*
Fleming KM, Aithal GP, Solaymani-Dodaran M, Card TR, West J. Incidence and prevalence of cirrhosis in the United Kingdom, 1992-2001: a gener population-based study. J Hepatol. 2008; 49(5): 732-8.	1992-2001	
Mathur R, Pollara E, Hull S, Schofield P, Ashworth M, Robson J. Ethnicity and stroke risk in patients with atrial fibrillation. Heart. 2013; 99(15): 108 92.	2008-2011	*
BMRB. Social Research, Infant Feeding Survey, 2005 [computer file]. Colchester, Essex: UK Data Archive [distributor], October 2007. SN: 5727 http://dx.doi.org/10.5255/UKDA-SN-5727-1	2005	
BMRB International and BMRB. Social Research, Infant Feeding Survey, 2000 [computer file]. Colchester, Essex: UK Data Archive [distributor November 2003. SN: 4746 , http://dx.doi.org/10.5255/UKDA-SN-4746-1	2000	
Office of Population Censuses and Surveys. Social Survey Division, Infant Feeding Survey, 1995 [computer file]. Colchester, Essex: UK Data Archi [distributor], August 1998. SN: 3778 , http://dx.doi.org/10.5255/UKDA-SN-3778-1	1995	
Mallen CD, Mottram S, Wynne-Jones G, Thomas E. Birth-related exposures and asthma and allergy in adulthood: a population-based cross-section study of young adults in North Staffordshire. J Asthma. 2008; 45(4): 309-12.	2002	*
Simpson CR, Newton J, Hippisley-Cox J, Sheikh A. Trends in the epidemiology and prescribing of medication for eczema in England. J R Soc Me 2009; 102(3): 108-17.	2001-2005	*
Ziyab AH, Raza A, Karmaus W, Tongue N, Zhang H, Matthews S, Arshad SH, Roberts G. Trends in eczema in the first 18 years of life: results from t Isle of Wight 1989 birth cohort study. Clin Exp Allergy. 2010; 40(12): 1776-84.	1989-1990, 1993, 2007	*
Davies GM, Jones CM, Monaghan N, Morgan MZ, Neville JS, Pitts NB. The caries experience of 11 to 12 year-old children in Scotland and Wales and 12 year-olds in England in 2008-2009: reports of co-ordinated surveys using BASCD methodology. Community Dent Health. 2012; 29(1): 8-13.	2008-2009	*
Davies GM, Jones CM, Monaghan N, Morgan MZ, Pine CM, Pitts NB, Neville JS, Rooney E. The caries experience of 5 year-old children in Scotland, Wales and England in 2007-2008 and the impact of consent arrangements. Reports of co-ordinated surveys using BASCD criteria. Community Dent Health. 2011; 28(1): 5-11.	2007-2008	*
Naftalin J, Hoo W, Pateman K, Mavrelos D, Holland T, Jurkovic D. How common is adenomyosis? A prospective study of prevalence usin transvaginal ultrasound in a gynaecology clinic. Hum Reprod. 2012; 27(12): 3432-9.	2009-2010	*
Oakley L, Doyle P, Maconochie N. Lifetime prevalence of infertility and infertility treatment in the UK: results from a population-based survey reproduction. Hum Reprod. 2008; 23(2): 447-50.	2001	*
Wilkes S, Chinn DJ, Murdoch A, Rubin G. Epidemiology and management of infertility: a population-based study in UK primary care. Fam Pract. 200 26(4): 269-74.	2005-2006	*
Day TG, Thein SL, Drasar E, Dick MC, Height SE, O'Driscoll S, Rees DC. Changing pattern of hospital admissions of children with sickle cell disea over the last 50 years. J Pediatr Hematol Oncol. 2011; 33(7): 491-5.	2008-2009	
Huerta C, Rivero E, Rodríguez LAG. Incidence and risk factors for psoriasis in the general population. Arch Dermatol. 2007; 143(12): 1559-65.	1996-1997	*
Emerson E. Deprivation, ethnicity and the prevalence of intellectual and developmental disabilities. J Epidemiol Community Health. 2012; 66(3): 21 24.	2008	*
Zimmermann A, Bernuit D, Gerlinger C, Schaefers M, Geppert K. Prevalence, symptoms and management of uterine fibroids: an international intern based survey of 21,746 women. BMC Womens Health. 2012; 12: 6.	2009	*
Macfarlane GJ, Beasley M, Jones EA, Prescott GJ, Docking R, Keeley P, McBeth J, Jones GT. The prevalence and management of low back pain acro adulthood: Results from a population-based cross-sectional study (the MUSICIAN study). Pain. 2012; 153(1): 27-32.	2008	*
Docking RE, Fleming J, Brayne C, Zhao J, Macfarlane GJ, Jones GT, Cambridge City over-75s Cohort Study collaboration. Epidemiology of back pain in older adults: prevalence and risk factors for back pain onset. Rheumatology. 2011; 50(9): 1645-53.	1988-1989, 1992-1993	*
Giaquinto C, van Damme P, REVEAL Study Group. Age distribution of paediatric rotavirus gastroenteritis cases in Europe: the REVEAL study. Scan J Infect Dis. 2010; 42(2): 142-7.	2004-2005	*
Copeland JR, McCracken CF, Dewey ME, Wilson KC, Doran M, Gilmore C, Scott A, Larkin BA. Undifferentiated dementia, Alzheimer's disease and vascular dementia: age- and gender-related incidence in Liverpool. The MRC-ALPHA Study. Br J Psychiatry. 1999; 175(5): 433-8.	1989-1998	
Livingston G, Sax K, Willison J, Blizard B, Mann A. The Gospel Oak Study stage II: the diagnosis of dementia in the community. Psychol Med. 199 20(4): 881-91.	1987-1989	
Davies AR, Grundy E, Nitsch D, Smeeth L. Constituent country inequalities in myocardial infarction incidence and case fatality in men and women the United Kingdom, 1996-2005. J Public Health (Oxf). 2011; 33(1): 131-8.	1996, 2000, 2005	*
Smolina K, Wright FL, Rayner M, Goldacre MJ. Incidence and 30-day case fatality for acute myocardial infarction in England in 2010: national-link database study. Eur J Public Health. 2012; 22(6): 848-53.	2010	*
Matthews FE, Arthur, Antony, Barnes, Linda E, Bond, John, Jagger, Carol, Robinson, Louise, Brayne, Carol. A two-decade comparison of prevalence dementia in individuals aged 65 years and older from three geographical areas of England: results of the Cognitive Function and Ageing Study I and II Lancet. 2013; 382(9902): 1405-1412.	1990-1993, 2008-2011	
Hart DJ, Spector TD. The relationship of obesity, fat distribution and osteoarthritis in women in the general population: the Chingford Study Rheumatol. 1993; 20(2): 331-5.	1989-1993	
Cooper C, Snow S, McAlindon TE, Kellingray S, Stuart B, Coggon D, Dieppe PA. Risk factors for the incidence and progression of radiographic kne osteoarthritis. Arthritis Rheum. 2000; 43(5): 995-1000.	1991-1996	
Neame RL, Carr AJ, Muir K, Doherty M. UK community prevalence of knee chondrocalcinosis: evidence that correlation with osteoarthritis is through shared association with osteophyte. Ann Rheum Dis. 2003; 62(6): 513-8.	2000	
Symmons DP, Jones MA, Scott DL, Prior P. Longterm mortality outcome in patients with rheumatoid arthritis: early presenters continue to do well Rheumatol. 1998; 25(6): 1072-7.	1982-1990	
Symmons DP, Prior P, Scott DL, Brown R, Hawkins CF. Factors influencing mortality in rheumatoid arthritis. J Chronic Dis. 1986; 39(2): 137-45.	1964-1981	
Chehata JC, Hassell AB, Clarke SA, Mattey DL, Jones MA, Jones PW, Dawes PT. Mortality in rheumatoid arthritis: relationship to single an composite measures of disease activity. Rheumatology. 2001; 40(4): 447-52.	1981-1998	
Goodson N, Marks J, Lunt M, Symmons D. Cardiovascular admissions and mortality in an inception cohort of patients with rheumatoid arthritis wi onset in the 1980s and 1990s. Ann Rheum Dis. 2005; 64(11): 1595-601.	1981-2002	
Goodson NJ, Wiles NJ, Lunt M, Barrett EM, Silman AJ, Symmons DPM. Mortality in early inflammatory polyarthritis: cardiovascular mortality increased in seropositive patients. Arthritis Rheum. 2002; 46(8): 2010-9.	1990-1999	
Young A, Koduri G, Batley M, Kulinskaya E, Gough A, Norton S, Dixey J. Mortality in rheumatoid arthritis. Increased in the early course of disease, ischaemic heart disease and in pulmonary fibrosis. Rheumatology. 2007; 46(2): 350-7.	1986-2004	
Symmons D, Turner G, Webb R, Asten P, Barrett E, Lunt M, Scott D, Silman A. The prevalence of rheumatoid arthritis in the United Kingdom: ne estimates for a new century. Rheumatology. 2002; 41(7): 793-800.	1999	

Rodríguez LAG, Tolosa LB, Ruigómez A, Johansson S, Wallander M-A. Rheumatoid arthritis in UK primary care: incidence and prior morbidit Scand J Rheumatol. 2009; 38(3): 173-7.	1996-1997	
Symmons DP, Barrett EM, Bankhead CR, Scott DG, Silman AJ. The incidence of rheumatoid arthritis in the United Kingdom: results from the Norfc Arthritis Register. Br J Rheumatol. 1994; 33(8): 735-9.	1990-1991	
Wiles N, Symmons DP, Harrison B, Barrett E, Barrett JH, Scott DG, Silman AJ. Estimating the incidence of rheumatoid arthritis: trying to hit a movi target? Arthritis Rheum. 1999; 42(7): 1339-46.	1996	
Davis A, Smith P, Ferguson M, Stephens D, Gianopoulos I. Acceptability, benefit and costs of early screening for hearing disability: a study of potent screening tests and models. Health Technol Assess. 2007; 11(42): 1-294.	1996	
Smith P, Davis A, Ferguson M, Lutman M. Hearing in Young Adults: Report to ISO/TC43/WG1. Noise Health. 1999; 1(4): 1-10.	1994	
Davis AC. The prevalence of hearing impairment and reported hearing disability among adults in Great Britain. Int J Epidemiol. 1989; 18(4): 911-7.	1983	
Office for National Statistics (United Kingdom). United Kingdom Adult Dental Health Survey 2009-2010 - ON	2009-2010	
Office for National Statistics (United Kingdom). United Kingdom Children's Dental Health Survey 2003 - ON	2003	
European Commission (2012): Eurobarometer 72.3 (Oct 2009). TNS OPINION & SOCIAL, Brussels [Producer]. GESIS Data Archive, Cologne. ZA4977 Data file Version 2.0.0, doi:10.4232/1.11140	2009	
European Commission (2012): Eurobarometer 66.2 (Oct-Nov 2006). TNS OPINION & SOCIAL, Brussels [Producer]. GESIS Data Archive, Cologne ZA4527 Data file Version 1.0.1, doi:10.4232/1.10981	2006	
European Commission (2012): Eurobarometer 64.3 (Nov-Dec 2005). TNS OPINION & SOCIAL, Brussels [Producer]. GESIS Data Archive, Cologne ZA4415 Data file Version 1.0.1, doi:10.4232/1.10971	2005	
European Commission (2012): Eurobarometer 58.2 (Oct-Dec 2002). European Opinion Research Group (EORG), Brussels. GESIS Data Archive, Cologne. ZA3886 Data file Version 1.0.1, doi:10.4232/1.10954	2002	
Crocombe LA, Mejia GC, Koster CR, Slade GD. Comparison of adult oral health in Australia, the USA, Germany and the UK. Aust Dent J. 200 54(2): 147-53.	1998	
Bolin AK, Bolin A, Koch G. Children's dental health in Europe: caries experience of 5- and 12-year-old children from eight EU countries. Int J Paedi Dent. 1996; 6(3): 155-62.	1993	
Anderson RJ, Bradnock G, James PM. The dental health of Guernsey school children 1984. Br Dent J. 1986; 160(9): 323-	1984	
Silver DH. Improvements in the dental health of 3-year-old Hertfordshire children after 8 years. Br Dent J. 1982; 153(5): 179-83.	1973, 1981	
Jenkins P. West Midlands caries prevalence studies, 15 year old schoolchildren 1986. Community Dent Health. 1989; 6(1): 67-8.	1986	
Andlaw RJ, Burchell CK, Tucker GJ. Comparison of dental health of 11-year-old children in 1970 and 1979, and of 14-year-old children in 1973 ar 1979: studies in Bristol, England. Caries Res. 1982; 16(3): 257-64.	1970-1973	
Zoitopoulos L, Athanassouli T, Gelbier S, Apostolopoulos A. Caries prevalence of 5-year-old children in Athens and in South London. Int J Paedi Dent. 1996; 6(1): 3-6.	1993	
Holt RD, Winter GB, Downer MC, Bellis WJ, Hay IS. Caries in pre-school children in Camden 1993/94. Br Dent J. 1996; 181((11-12)): 405-10.	1986, 1993-1994	
Silver DH. A comparison of 3-year-olds' caries experience in 1973, 1981 and 1989 in a Hertfordshire town, related to family behaviour and social clas Br Dent J. 1992; 172(5): 191-7.	1973, 1981, 1989	
Jones SG, Nunn JH. The dental health of 3-year-old children in east Cumbria 1993. Community Dent Health. 1995; 12(3): 161-6.	1993	
Davies GM, Worthington HV, Ellwood RP, Bentley EM, Blinkhorn AS, Taylor GO, Davies RM. A randomised controlled trial of the effectiveness of providing free fluoride toothpaste from the age of 12 months on reducing caries in 5-6 year old children. Community Dent Health. 2002; 19(3): 131-6.	1993	
Marchment MD, Gray MM. A caries prevalence study of 5-year-old children in South-East Staffordshire in 1985 which resulted in the unexpected finding of a naturally fluoridated water supply to the town of Uttoxeter. Community Dent Health. 1987; 4(2): 117-9.	1984-1986	
Davies GM, Blinkhorn FA, Duxbury JT. Caries among 3-year-olds in greater Manchester. Br Dent J. 2001; 190(7): 381-	1999	
Commission of the European Communities (2012): Eurobarometer 38.0 (Sep-Oct 1992). INRA, Brussels. GESIS Data Archive, Cologne. ZA229 Data file Version 1.1.0, doi:10.4232/1.10903	1992	
Commission of the European Communities (2012): Eurobarometer 36 (Oct-Nov 1991). INRA, Brussels. GESIS Data Archive, Cologne. ZA2081 Dat file Version 1.1.0, doi:10.4232/1.10848	1991	
Commission of the European Communities (2012): Eurobarometer 32 (Oct-Nov 1989). INRA, Brussels. GESIS Data Archive, Cologne. ZA1752 Dat file Version 1.1.0, doi:10.4232/1.10890	1989	
European Commission (2012): Eurobarometer 43.0 (Mar-Apr 1995). INRA, Brussels. GESIS Data Archive, Cologne. ZA2636 Data file Version 1.0.1 doi:10.4232/1.10912	1995	
European Commission (2012): Eurobarometer 41.0 (Mar-May 1994). INRA, Brussels. GESIS Data Archive, Cologne. ZA2490 Data file Versior 1.1.0, doi:10.4232/1.10909	1994	
Commission of the European Communities (2012): Eurobarometer 27 (Mar-May 1987). Faits et Opinions, Paris. GESIS Data Archive, Cologne ZA1712 Data file Version 1.0.1, doi:10.4232/1.10884	1987	
Crisp AH, Callender JS, Halek C, Hsu LK. Long-term mortality in anorexia nervosa. A 20-year follow-up of the St George's and Aberdeen cohorts. B Psychiatry. 1992; 161(1): 104-7.	1968-1990	
Ford T, Goodman R, Meltzer H. The British Child and Adolescent Mental Health Survey 1999: the prevalence of DSM-IV disorders. J Am Acad Chi Adolesc Psychiatry. 2003; 42(10): 1203-11.	1999	
Messer J, Goodman, Robert, Rowe, Richard, Meltzer, Howard, Maughan, Barbara. Preadolescent Conduct Problems in Girls and Boys. J Am Ac Child Adolesc Psychiatry. 2006; 45(2): 184-91.	1997	
Kim-Cohen J, Arseneault L, Caspi A, Tomás MP, Taylor A, Moffitt TE. Validity of DSM-IV conduct disorder in 41/2-5-year-old children: longitudinal epidemiological study. Am J Psychiatry. 2005; 162(6): 1108-17.	1997	
Elliott PM, Gimeno JR, Thaman R, Shah J, Ward D, Dickie S, Tome Esteban MT, McKenna WJ. Historical trends in reported survival rates in patien with hypertrophic cardiomyopathy. Heart. 2006; 92(6): 785-91.	1988-2002	
Office for National Statistics (United Kingdom). United Kingdom - England and Wales Mortality Data 1981-1994. Newport, United Kingdom: Offi for National Statistics (United Kingdom). [Unpublished].	1981-1994	
Office for National Statistics (United Kingdom). United Kingdom - England and Wales Mortality Data 1995-2000. Newport, United Kingdom: Offi for National Statistics (United Kingdom). [Unpublished].	1995-2000	
Office for National Statistics (United Kingdom). United Kingdom - England and Wales Mortality Data 2001-2012. Newport, United Kingdom: Offi for National Statistics (United Kingdom). [Unpublished].	2001-2012	
Robertson WG, Peacock M, Baker M, Marshall DH, Pearlman B, Speed R, Sergeant V, Smith A. Studies on the prevalence and epidemiology of urina stone disease in men in Leeds. Br J Urol. 1983; 55(6): 595-8.	1981-1983	
Ayuso-Mateos JL, Vázquez-Barquero JL, Dowrick C, Lehtinen V, Dalgard OS, Casey P, Wilkinson C, Lasa L, Page H, Dunn G, Wilkinson Depressive disorders in Europe: prevalence figures from the ODIN study. Br J Psychiatry. 2001; 179(4): 308-16.	1999-2001	
Skehan JD, Murray M, Mills PG. Infective endocarditis: incidence and mortality in the North East Thames Region. Br Heart J. 1988; 59(1): 62-8.	1982-1984	
Waterstone M, Bewley S, Wolfe C. Incidence and predictors of severe obstetrics morbidity: case-control study. BMJ. 2001; 322(7294): 1089-94.	1997-1998	
de Marco R, Accordini S, Cerveri I, Corsico A, Sunyer J, Neukirch F, Künzli N, Leynaert B, Janson C, Gislason T, Vermeire P, Svanes C, Anto JM, Burney P, European Community Respiratory Health Survey Study Group. An international survey of chronic obstructive pulmonary disease in young adults according to GOLD stages. Thorax. 2004; 59(2): 120-125.	1991-1993	
Buist AS, McBurnie MA, Vollmer WM, Gillespie S, Burney P, Mannino DM, Menezes AM, Sullivan SD, Lee TA, Weiss KB, Jensen RL, Marks GB, Gulsvik A,Nizankowska-Mogilnicka E, BOLD Collaborative Research Group. International variation in the prevalence of COPD (the BOLD Study): a population-based prevalence study. Lancet. 2007; 9589(9589): 741-50.	1991-2000, 2005-2006	
Higham J, Kang J-Y, Majeed A. Recent trends in admissions and mortality due to peptic ulcer in England: increasing frequency of haemorrhage amo older subjects. Gut. 2002; 50(4): 460-4.	1989-1998	
Chakrabarti S, Fombonne E. Pervasive developmental disorders in preschool children. JAMA. 2001; 285(24): 3093-9	1998-2003	
Chakrabarti S, Fombonne E. Pervasive Developmental Disorders in Preschool Children: Confirmation of High Prevalence. Am J Psychiatry. 200 162(6): 1133-41.	2000-2003	
Powell J, Edwards A, Edwards M, Pandit B, Sungum-Paliwal S, Whitehouse W. Changes in the incidence of childhood autism and other autistic spectrum disorders in preschool children from two areas of the West Midlands, UK. Dev Med Child Neurol. 2000; 42(9): 624-8.	1991-1996	
Baird G, Simonoff E, Pickles A, Chandler S, Loucas T, Meldrum D, Charman T. Prevalence of disorders of the autism spectrum in a population cohor of children in South Thames: the Special Needs and Autism Project (SNAP). Lancet. 2006; 368(9531): 210-5.	2003	
Selo-Ojeme D, Lawal O, Shah J, Mandal R, Pathak S, Selo-Ojeme U, Samuel D. The incidence of uterine leiomyoma and other pelvic ultrasonographic findings in 2,034 consecutive women in a north London hospital. J Obstet Gynaecol. 2008; 28(4): 421-3.	2005	

Downes E, Sikirica V, Gilabert-Estelles J, Bolge SC, Dodd SL, Maroulis C, Subramanian D. The burden of uterine fibroids in five European countrie Eur J Obstet Gynecol Reprod Biol. 2010; 152(1): 96-102.	2007	
Stones W, Lim W, Al-Azzawi F, Kelly M. An investigation of maternal morbidity with identification of life-threatening 'near miss' episodes. Hea Trends. 1991; 23(1): 13-5.	1986	
Sebire NJ, Jolly M, Harris JP, Wadsworth J, Joffe M, Beard RW, Regan L, Robinson S. Maternal obesity and pregnancy outcome: a study of 2872 pregnancies in London. Int J Obes Relat Metab Disord. 2001; 25(8): 1175-1182.	1989-1997	
Goodman R FT, Richards H, Gatward R, Meltzer H. The Development and Well-Being Assessment: description and initial validation of an integrated assessment of child and adolescent psychopathology. J Child Psychol Psychiatry. 2000; 41(5): 645-55.	1998	
McArdle P, Prosser J, Kolvin I. Prevalence of psychiatric disorder: with and without psychosocial impairment. Eur Child Adolesc Psychiatry. 200 13(6): 347-53.	2002	
United States Renal Data System Coordinating Center. USRDS 2010 Annual Data Report: Atlas of Chronic Kidney Disease and End-Stage Renal Disease in the United States. Bethesda, United States: National Institute of Diabetes and Digestive and Kidney Diseases (NIDDK), National Institutes Health, 2010.	2004-2008	
Human Nutrition Research, Medical Research Council, National Centre for Social Research (NatCen), Northern Ireland Statistics and Research Agency (NISRA), University College London Medical School. United Kingdom National Diet and Nutrition Survey 2008-2011 - Public Health England.	2008-2011	*
United States Renal Data System Coordinating Center. USRDS 2009 Annual Data Report: Atlas of Chronic Kidney Disease and End-Stage Renal Disease in the United States. Bethesda, United States: National Institute of Diabetes and Digestive and Kidney Diseases (NIDDK), National Institutes Health, 2009.	2000-2003	
United States Renal Data System Coordinating Center. USRDS 2003 Annual Data Report: Atlas of End-Stage Renal Disease in the United States. Bethesda, United States: National Institute of Diabetes and Digestive and Kidney Diseases (NIDDK), National Institutes of Health, 2003.	1998-1999	
LaMontagne DS, Fenton KA, Randall S, Anderson S, Carter P. Establishing the National Chlamydia Screening Programme in England: results from t first full year of screening. Sex Transm Infect. 2004; 80(5): 335-41.	2004	
NHS England. United Kingdom - England Hospital Episode Statistics 2002-2003	2002	
NHS England. United Kingdom - England Hospital Episode Statistics 2003-2004	2003	
NHS England. United Kingdom - England Hospital Episode Statistics 2004-2005	2004-2005	
NHS England. United Kingdom - England Hospital Episode Statistics 2005-2006	2005	
NHS England. United Kingdom - England Hospital Episode Statistics 2006-2007	2006	
NHS England. United Kingdom - England Hospital Episode Statistics 2007-2008	1981-1990, 2005, 2007-2008	
NHS England. United Kingdom - England Hospital Episode Statistics 2008-2009	2008	
NHS England. United Kingdom - England Hospital Episode Statistics 2009-2010	2009	
NHS England. United Kingdom - England Hospital Episode Statistics 2010-2011	2010	
NHS England. United Kingdom - England Hospital Episode Statistics 2011-2012	2011-2012	
General Register Office for Scotland, Northern Ireland Statistics and Research Agency (NISRA), Office for National Statistics (United Kingdom United Kingdom All-Cause Mortality Data 1950-2012. [Unpublished].	1950-2012	
United Kingdom Cancer Information Service (UKCIS). United Kingdom - England Cancer Incidence Data 1990-2010. [Unpublished].	1990-2010	
Office of Population Censuses and Surveys. Social Survey Division, OPCS Surveys of Psychiatric Morbidity : Private Household Survey, 1993 [computer file]. Colchester, Essex: UK Data Archive [distributor], September 1996. SN: 3560, http://dx.doi.org/10.5255/UKDA-SN-3560-1	1993	*
Office for National Statistics, Psychiatric Morbidity among Adults Living in Private Households, 2000 [computer file]. Colchester, Essex: UK Da Archive [distributor], May 2003. SN: 4653, http://dx.doi.org/10.5255/UKDA-SN-4653-1	2000	
Foltynie T, Brayne CEG, Robbins TW, Barker RA. The cognitive ability of an incident cohort of Parkinson's patients in the UK. The CamPaIGN stu Brain. 2004; 127(3): 550-60.	2000-2003	
Hobson P, Gallacher J, Meara J. Cross-sectional survey of Parkinson's disease and parkinsonism in a rural area of the United Kingdom. Mov Disor 2005; 20(8): 995-8.	1998	
Cockerel O, Goodridge DM, Brodie D, Sander JWA, Shorvon S. Neurological Disease in a Defined Population: The Results of a Pilot Study in Tw General Practices. Neuroepidemiology. 1996; 15(2): 73-82.	1993	
Prettyman R. Extrapyramidal signs in cognitively intact elderly people. Age Ageing. 1998; 27(5): 557-6	1995	
Schrag A, Ben-Shlomo Y, Quinn NP. Cross sectional prevalence survey of idiopathic Parkinson's disease and parkinsonism in London. BMJ. 200 321(7252): 21-2.	1997	
Sutcliffe RL, Prior R, Mawby B, McQuillan WJ. Parkinson's disease in the district of the Northampton Health Authority, United Kingdom. A study prevalence and disability. Acta Neurol Scand. 1985; 72(4): 363-79.	1982	
Sutcliffe RLG, Meara JR. Parkinson's disease epidemiology in the Northampton District, England, 1992. Acta Neurol Scand. 1995; 92(6): 443-50.	1989	
Wade DT, Hewer RL. Epidemiology of some neurological diseases with special reference to work load on the NHS. Disabil Rehabil. 1986; 8(3): 12 37.	1984	
Goldacre M, Shiwach R, Yeates D. Estimating incidence and prevalence of treated psychiatric disorders from routine statistics: the example schizophrenia in Oxfordshire. J Epidemiol Community Health. 1994; 48(3): 318-22.	1986	
McCreadie RG, Leese M, Tilak-Singh D, Loftus L, MacEwan T, Thornicroft G. Nithsdale, Nunhead and Norwood: similarities and differences prevalence of schizophrenia and utilisation of services in rural and urban areas. Br J Psychiatry. 1997; 31-6.	1992-1993	
Bamrah JS, Freeman HL, Goldberg DP. Epidemiology of schizophrenia in Salford, 1974-84. Changes in an urban community over ten years. Br Psychiatry. 1991; 802-10.	1983-1984	
Jeffreys SE, Harvey CA, McNaught AS, Quayle AS, King MB, Bird AS. The Hampstead Schizophrenia Survey 1991. I: Prevalence and service u comparisons in an inner London health authority, 1986-1991. Br J Psychiatry. 1997; 301-6.	1991	
Harvey CA, Pantelis C, Taylor J, McCabe PJ, Lefevre K, Campbell PG, Hirsch SR. The Camden schizophrenia surveys. II. High prevalence of schizophrenia in an inner London borough and its relationship to socio-demographic factors. Br J Psychiatry. 1996; 168(4): 418-26.	1981, 1985-1986	
King M, Coker E, Leavey G, Hoare A, Johnson-Sabine E. Incidence of psychotic illness in London: comparison of ethnic groups. BMJ. 199 309(6962): 1115-9.	1991-1992	
McNaught AS, Jeffreys SE, Harvey CA, Quayle AS, King MB, Bird AS. The Hampstead Schizophrenia Survey 1991. II: Incidence and migration inner London. Br J Psychiatry. 1997; 307-11.	1986-1991	
Harrison G, Hopper K, Craig T, Laska E, Siegel C, Wanderling J, Dube KC, Ganev K, Giel R, an der Heiden W, Holmberg SK, Janca A, Lee PW, Leon CA, Malhotra S, Marsella AJ, Nakane Y, Sartorius N, Shen Y, Skoda C, Thara R, Tsirkin SJ, Varma VK, Walsh D, Wiersma D. Recovery from psychotic illness: a 15- and 25-year international follow-up study. Br J Psychiatry. 2001; 178: 506-17.	1978-1993	
European School Survey Project on Alcohol and Other Drugs (ESPAD), Pompidou Group, Council of Europe, Swedish Council for Information on Alcohol and Other Drugs (CAN). ESPAD Report 2003: Alcohol and Other Drug Use Among Students in 35 European Countries. Stockholm, Sweden: Swedish Council for Information on Alcohol and Other Drugs (CAN), 2004.	2003	
Singleton N, Bumpstead R, O'Brien M, Lee A, Meltzer H. Psychiatric morbidity among adults living in private households, 2000. Int Rev Psychiat 2003; 15(1-2): 65-73.	2000	
Department of Health (United Kingdom), Office for National Statistics (United Kingdom). The Prevalence of Back Pain in Great Britain in 199 United Kingdom: Department of Health (United Kingdom), 1999.	1998	
Murphy S, Buckle P, Stubbs D. A cross-sectional study of self-reported back and neck pain among English schoolchildren and associated physical a psychological risk factors. Arch Dis Child. 2007; 38(6): 797-804.	2001	
Palmer KT, Walsh K, Bendall H, Cooper C, Coggon D. Back pain in Britain: comparison of two prevalence surveys at an interval of 10 years. BM 2000; 320(7249): 1577-8.	1997-1998	
Croft PR, Rigby AS. Socioeconomic influences on back problems in the community in Britain. J Epidemiol Community Health. 1994; 48(2): 166-70.	1984-1985, 1989, 1993	
Watson KD, Papageorgiou AC, Jones GT, Taylor S, Symmons DPM, Silman AJ, Macfarlane GJ. Low back pain in schoolchildren: occurrence a characteristics. Pain. 2002; 97(1-2): 87-92.	1994-1997, 1999-2000	
Harkness EF, Macfarlane GJ, Silman AJ, McBeth J. Is musculoskeletal pain more common now than 40 years ago?: Two population-based cro sectional studies. Rheumatology. 2005; 44(7): 890-5.	1994-1995	
Papageorgiou AC, Croft PR, Ferry S, Jayson MI, Silman AJ. Estimating the prevalence of low back pain in the general population. Evidence from t South Manchester Back Pain Survey. Spine. 1995; 20(17): 1889-94.	1992	
Wormald RP, Wright LA, Courtney P, Beaumont B, Haines AP. Visual problems in the elderly population and implications for services. BMJ. 199 304(6836): 1226-9.	1989-1990	
Van der Pols JC, Bates CJ, McGraw PV, Thompson JR, Reacher M, Prentice A, Finch S. Visual acuity measurements in a national sample of Britis elderly people. Br J Ophthalmol. 2000; 84(2): 165-70.	1994-1995	
Stewart-Brown S, Butler N. Visual acuity in a national sample of 10 year old children. J Epidemiol Community Health. 1985; 39(2): 107-12.	1980	
Gibson JM, Lavery JR, Rosenthal AR. Blindness and partial sight in an elderly population. Br J Ophthalmol. 1986; 70(9): 700-	1982-1984	

Kangro HO, Osman HK, Lau YL, Heath RB, Yeung CY, Ng MH. Seroprevalence of antibodies to human herpesviruses in England and Hong Kong Med Virol. 1994; 43(1): 91-6.	1991	
Vyse AJ, Gay NJ, Hesketh LM, Morgan-Capner P, Miller E. Seroprevalence of antibody to varicella zoster virus in England and Wales in children ar young adults. Epidemiol Infect. 2004; 132(6): 1129-34.	1996	
Kudesia G, Partridge S, Farrington CP, Soltanpoor N. Changes in age related seroprevalence of antibody to varicella zoster virus: impact on vaccir strategy. J Clin Pathol. 2002; 55(2): 154-5.	1966-1992	
Chandramouli K, Steer CD, Ellis M, Emond AM. Effects of early childhood lead exposure on academic performance and behaviour of school a children. Arch Dis Child. 2009; 94(11): 844-8.	1991-1992	
Taylor CM, Golding J, Hibbeln J, Emond AM. Environmental Factors Predicting Blood Lead Levels in Pregnant Women in the UK: The ALSPA Study. PLoS One. 2013; 8(9): e72371.	1991-1992	
European Monitoring Centre for Drugs and Drug Addiction (EMCDDA). European Monitoring Centre for Drugs and Drug Addiction Statistica Bulletin 2008. Lisbon, Portugal: European Monitoring Centre for Drugs and Drug Addiction (EMCDDA).	1994-1996, 1998-2007	
Home Office (United Kingdom). Drug Misuse Declared: Findings from the 2006/07 British Crime Survey England and Wales. London, Unite Kingdom: Home Office (United Kingdom), 2007.	1996, 1998, 2000-2003, 2006-2007	
Das VK. Aetiology of bilateral sensorineural hearing impairment in children: a 10 year study. Arch Dis Child. 1996; 74(1): 8-1	1981-1994	
European Monitoring Centre for Drugs and Drug Addiction (EMCDDA). European Monitoring Centre for Drugs and Drug Addiction Statistica Bulletin 2013. Lisbon, Portugal: European Monitoring Centre for Drugs and Drug Addiction (EMCDDA).	2003-2007, 2011-2012	
Home Office (United Kingdom). Drug Misuse Declared: Results of the 1994 British Crime Survey. London, United Kingdom: Home Office (Unit Kingdom), 1996.	1994	
WHO Regional Office for Europe (EURO-WHO). Young People's Health in Context. Health Behaviour in School-aged Children (HBSC) Study: International Report from the 2001-2002 Survey. Copenhagen, Denmark: WHO Regional Office for Europe (EURO-WHO), 2004.	2001-2002	
WHO Regional Office for Europe (EURO-WHO). Inequalities in Young People's Health: HBSC International Report from the 2005/2006 Survey Copenhagen, Denmark: WHO Regional Office for Europe (EURO-WHO), 2008.	2005-2006	
ESPAD Report 1995: Alcohol and Other Drug Use Among Students in 26 European Countries as it appears in P.N. Lee Statistics and Computing International Mortality and Smoking Statistics.	1995	
ESPAD Report 1999: Alcohol and Other Drug Use Among Students in 30 European Countries as it appears in P.N. Lee Statistics and Computing International Mortality and Smoking Statistics.	1999	
United Kingdom National Diet and Nutrition Survey: Adults Aged 19 to 64 Years 2000-2001 as it appears in Global Dietary Database Consortium, Nutrition and Chronic Disease Expert Group (NutriCoDE). Global Dietary Database 1980-2011. [Unpublished].	2000-2001	
Office for National Statistics. Social and Vital Statistics Division and Food Standards Agency, National Diet and Nutrition Survey : Adults Aged 19 to 64 Years, 2000-2001 [computer file]. Colchester, Essex: UK Data Archive [distributor], May 2005. SN: 5140 , http://dx.doi.org/10.5255/UKDA-SN-5140-1.	2000-2001	
United Kingdom Dietary and Nutritional Survey of British Adults 1986-1987 as it appears in Global Dietary Database Consortium, Nutrition ar Chronic Disease Expert Group (NutriCoDE). Global Dietary Database 1980-2011. [Unpublished].	1986-1987	
Office of Population Censuses and Surveys. Social Survey Division, Dietary and Nutritional Survey of British Adults, 1986-1987 [computer file]. Colchester, Essex: UK Data Archive [distributor], September 1991. SN: 2836, http://dx.doi.org/10.5255/UKDA-SN-2836-1.	1986-1987	
Eltze CM, Chong WK, Cox T, Whitney A, Cortina-Borja M, Chin RFM, Scott RC, Cross JH. A population-based study of newly diagnosed epilepsy infants. Epilepsia. 2013; 54(3): 437-45.	2005-2006	*
National Centre for Social Research (NatCen), National Foundation for Educational Research (NFER) (UK). United Kingdom - Smoking, Drinking and Drug Use Among Young People in England in 2006: Headline Figures. Leeds, England: Information Centre for Health and Social Care, NHS, 2007.	2001-2006	
Hawkins NM, Scholes S, Bajekal M, Love H, O'Flaherty M, Raine R, Capewell S. Community care in England: reducing socioeconomic inequalities heart failure. Circulation. 2012; 126(9): 1050-7.	1999, 2007	
The INTERSALT Co-operative Research Group. Appendix tables. Centre-specific results by age and sex. J Hum Hypertens 1989;3(5):331-407. as it appears in Global Dietary Database Consortium, Nutrition and Chronic Disease Expert Group (NutriCoDE). Global Dietary Database 1980-2011. [Unpublished].	1985-1987	
Tomkinson J, Turnbull A, Robson G, Dawson I, Cloake E, Adelstein AM, Ashley J. Report on confidential enquiries into maternal deaths in Englar and Wales 1976-1978. Rep Health Soc Subj (Lond). 1982; 26: 1-180.	1976-1978	*
Turnbull AC, Tindall VR, Robson G, Dawson IM, Cloake EP, Ashley JS. Report on confidential enquiries into maternal deaths in England and Wal 1979-1981. Rep Health Soc Subj (Lond). 1986; 29: 1-147.	1979-1981	*
Turnbull A, Tindall VR, Beard RW, Robson G, Dawson IM, Cloake EP, Ashley JS, Botting B. Report on Confidential Enquiries into Maternal Deatl in England and Wales 1982-1984. Rep Health Soc Subj (Lond). 1989; 34: 1-166.	1982-1984	*
Wildman K, Bouvier-Colle M-H, MOMS Group. Maternal mortality as an indicator of obstetric care in Europe. BJOG. 2004; 111(2): 164-9.	1992-1995	*
Eagles JM, Lee AJ, Raja EA, Millar HR, Bhattacharya S. Pregnancy outcomes of women with and without a history of anorexia nervosa. Psychol Me 2012; 42(12): 2651-60.	1965-2007	*
Oteng-Ntim E, Kopeika J, Seed P, Wandiembe S, Doyle P. Impact of obesity on pregnancy outcome in different ethnic groups: calculating populati attributable fractions. PLoS One. 2013; 8(1): e53749.	2004-2008	*
Akolekar R, Syngelaki A, Sarquis R, Zvanca M, Nicolaides KH. Prediction of early, intermediate and late pre-eclampsia from maternal factor biophysical and biochemical markers at 11-13 weeks. Prenat Diagn. 2011; 31(1): 66-74.	2006-2009	*
Dennedy MC, Avalos G, O'Reilly MW, O'Sullivan EP, Gaffney G, Dunne F. ATLANTIC-DIP: raised maternal body mass index (BMI) adversely affe maternal and fetal outcomes in glucose-tolerant women according to International Association of Diabetes and Pregnancy Study Groups (IADPSG) criteria. J Clin Endocrinol Metab. 2012; 97(4): E608-12.	2006-2009	*
Kayem G, Kurinczuk JJ, Spark P, Brocklehurst P, Knight M, UK Obstetric Surveillance System. Maternal and obstetric factors associated with delaye postpartum eclampsia: a national study population. Acta Obstet Gynecol Scand. 2011; 90(9): 1017-23.	2005-2006	*
Khalil A, Akolekar R, Syngelaki A, Elkhouli M, Nicolaides KH. Maternal hemodynamics at 11-13 weeks' gestation and risk of pre-eclampsi Ultrasound Obstet Gynecol. 2012; 40(1): 28-34.	2009-2011	*
Khalil A, Rezende J, Akolekar R, Syngelaki A, Nicolaides KH. Maternal racial origin and adverse pregnancy outcome: a cohort study. Ultrasou Obstet Gynecol. 2013; 41(3): 278-85.	2009-2011	*
North RA, McCowan LME, Dekker GA, Poston L, Chan EHY, Stewart AW, Black MA, Taylor RS, Walker JJ, Baker PN, Kenny LC. Clinical risk prediction for pre-eclampsia in nulliparous women: development of model in international prospective cohort. BMJ. 2011; d1875.	2004-2008	*
Roberts CL, Ford JB, Algert CS, Antonsen S, Chalmers J, Cnattingius S, Gokhale M, Kotelchuck M, Melve KK, Langridge A, Morris C, Morris JM, Nassar N, Norman JE, Norrie J, Sørensen HT, Walker R, Weir CJ. Population-based trends in pregnancy hypertension and pre-eclampsia: an international comparative study. BMJ Open. 2011; 1(1): e000101.	1997-2006	*
Smits LJM, North RA, Kenny LC, Myers J, Dekker GA, McCowan LME. Patterns of vaginal bleeding during the first 20 weeks of pregnancy and risk pre-eclampsia in nulliparous women: results from the SCOPE study. Acta Obstet Gynecol Scand. 2012; 91(11): 1331-8.	2004-2008	*
Wright D, Akolekar R, Syngelaki A, Poon LCY, Nicolaides KH. A competing risks model in early screening for preeclampsia. Fetal Diagn Ther. 201 32(3): 171-8.	2006-2010	*
Livshits G, Zhai G, Hart DJ, Kato BS, Wang H, Williams FM, Spector TD. Interleukin-6 is a significant predictor of radiographic knee osteoarthriti The Chingford study. Arthritis Rheum. 2009; 60(7): 2037-45.	1989	*
Ipsos MORI, NHS England. United Kingdom - England General Practitioner Patient Survey 2012-2013. Redditch, England: NHS England.	2012-2013	*
Kurtz Z, Tookey P, Ross E. Epilepsy in young people: 23 year follow up of the British national child development study. BMJ. 1998; 316(7128): 33 42.	1958-1981, 1984	*
MacDonald BK, Cockerell OC, Sander JW, Shorvon SD. The incidence and lifetime prevalence of neurological disorders in a prospective communit based study in the UK. Brain. 2000; 123 (Pt 4): 665-76.	1996	*
Cockerell OC, Eckle I, Goodridge DM, Sander JW, Shorvon SD. Epilepsy in a population of 6000 re-examined: secular trends in first attendance rate prevalence, and prognosis. J Neurol Neurosurg Psychiatry. 1995; 58(5): 570-6.	1983	*
Heaney DC, MacDonald BK, Everitt A, Stevenson S, Leonardi GS, Wilkinson P, Sander JW. Socioeconomic variation in incidence of epileps prospective community based study in south east England. BMJ. 2002; 325(7371): 1013-6.	1995-1997	*
Hamdy NA, Ginby D, Feltbower R, Ferrie CD. Ethnic differences in the incidence of seizure disorders in children from Bradford, United kingdo Epilepsia. 2007; 48(5): 913-6.	1999-2000	*
Tallis R, Hall G, Craig I, Dean A. How common are epileptic seizures in old age? Age Ageing. 1991; 20(6): 442-8	1989	*
Hart YM, Shorvon SD. The nature of epilepsy in the general population. I. Characteristics of patients receiving medication for epilepsy. Epilepsy Re 1995; 21(1): 43-9.	1994	*
Tidman L, Saravanan K, Gibbs J. Epilepsy in mainstream and special educational primary school settings. Seizure. 2003; 12(1): 47-51.	1996-1997	*
Verity CM, Ross EM, Golding J. Epilepsy in the first 10 years of life: findings of the child health and education study. BMJ. 1992; 305(6858): 857-61	1980	*

Wallace H, Shorvon S, Tallis R. Age-specific incidence and prevalence rates of treated epilepsy in an unselected population of 2,052,922 and ag specific fertility rates of women with epilepsy. Lancet. 1998; 352(9145): 1970-3.	1995	*
Wright J, Pickard N, Whitfield A, Hakin N. A population-based study of the prevalence, clinical characteristics and effect of ethnicity in epileps Seizure. 2000; 9(5): 309-13.	1998	*
Mohanraj R, Norrie J, Stephen LJ, Kelly K, Hitiris N, Brodie MJ. Mortality in adults with newly diagnosed and chronic epilepsy: a retrospecti comparative study. Lancet Neurol. 2006; 5(6): 481-7.	1981-2001	*
Raymond NT, Zehnder D, Smith SC, Stinson JA, Lehnert H, Higgins RM. Elevated relative mortality risk with mild-to-moderate chronic kidney disea decreases with age. Nephrol Dial Transplant. 2007; 22(11): 3214-20.	2000-2003	
Adamson JA, Price GM, Breeze E, Bulpitt CJ, Fletcher AE. Are older people dying of depression? Findings from the Medical Research Council trial of the assessment and management of older people in the community. J Am Geriatr Soc. 2005; 53(7): 1128-32.	1996-2001	
Copeland JR, Beekman AT, Dewey ME, Hooijer C, Jordan A, Lawlor BA, Lobo A, Magnusson H, Mann AH, Meller I, Prince MJ, Reischies F, Turrina C, deVries MW, Wilson KC. Depression in Europe. Geographical distribution among older people. Br J Psychiatry. 1999; 174: 312-21.	1993-1994	
Jenkins R, Lewis G, Bebbington P, Brugha T, Farrell M, Gill B, Meltzer H. The National Psychiatric Morbidity surveys of Great Britain-initial findin from the household survey. Psychol Med. 1997; 27(4): 775-89.	1993	
Lépine JP, Gastpar M, Mendlewicz J, Tylee A. Depression in the community: the first pan-European study DEPRES (Depression Research in Europea Society). Int Clin Psychopharmacol. 1997; 12(1): 19-29.	1995	
Meltzer H, Gatward R, Goodman R, Ford T. Mental health of children and adolescents in Great Britain. Int Rev Psychiatry. 2003; 15(1-2): 185-7.	1999	
Saunders PA, Copeland JR, Dewey ME, Gilmore C, Larkin BA, Phaterpekar H, Scott A. The prevalence of dementia, depression and neurosis in late life: the Liverpool MRC-ALPHA Study. Int J Epidemiol. 1993; 22(5): 838-47.	1986-1989	
McDougall FA, Kvaal K, Matthews FE, Paykel E, Jones PB, Dewey ME, Brayne C, Medical Research Council Cognitive Function and Ageing Study. Prevalence of depression in older people in England and Wales: the MRC CFA Study. Psychol Med. 2007; 37(12): 1787-95.	1990-1994	*
Baron-Cohen S, Scott FJ, Allison C, Williams J, Bolton P, Matthews FE, Brayne C. Prevalence of autism-spectrum conditions: UK school-base population study. Br J Psychiatry. 2009; 194(6): 500-9.	2005-2006	*
Howlin P, Goode S, Hutton J, Rutter M. Adult outcome for children with autism. J Child Psychol Psychiatry. 2004; 45(2): 212-29.	1970-1988	
Brewin J, Cantwell R, Dalkin T, Fox R, Medley I, Glazebrook C, Kwiecinski R, Harrison G. Incidence of schizophrenia in Nottingham. A compariso of two cohorts, 1978-80 and 1992-94. Br J Psychiatry. 1997; 171: 140-4.	1992-1994	*
Brown S, Kim M, Mitchell C, Inskip H. Twenty-five year mortality of a community cohort with schizophrenia. Br J Psychiatry. 2010; 196(2): 116-21.	1981-1982	*
Kirkbride JB, Croudace T, Brewin J, Donoghue K, Mason P, Glazebrook C, Medley I, Harrison G, Cooper JE, Doody GA, Jones PB. Is the incidence of psychotic disorder in decline? Epidemiological evidence from two decades of research. Int J Epidemiol. 2009; 38(5): 1255-64.	1992-1994	*
Pantelis C, Taylor J, Campbell P. The South Camden schizophrenia survey – an experience of community-based research. Psychiatric Bulletin. 198 12(3): 98-101.	1985	
World Health Organization (WHO). WHO Tuberculosis Case Notifications. Geneva, Switzerland: World Health Organization (WHO).	1998, 2001, 2004, 2007, 2010, 2012	
Office for National Statistics. Social and Vital Statistics Division et al. , Mental Health of Children and Young People in Great Britain, 2004 [computer file]. Colchester, Essex: UK Data Archive [distributor], October 2005. SN: 5269, http://dx.doi.org/10.5255/UKDA-SN-5269-1	2004	
National Centre for Social Research and University of Leicester, Adult Psychiatric Morbidity Survey, 2007 [computer file]. 3rd Edition. Colchester, Essex: UK Data Archive [distributor], January 2011. SN: 6379, http://dx.doi.org/10.5255/UKDA-SN-6379-1	2007	
Bevan CD, Ridgway GL, Rothermel CD. Efficacy and safety of azithromycin as monotherapy or combined with metronidazole compared with two standard multidrug regimens for the treatment of acute pelvic inflammatory disease. J Int Med Res. 2003; 31(1): 45-54.	2001-2002	*
Piyadigamage A, Wilson JD. An audit of outpatient management of pelvic inflammatory disease. Int J STD AIDS. 2002; 13(8): 577-9.	2000	*
Bevan CD, Johal BJ, Mumtaz G, Ridgway GL, Siddle NC. Clinical, laparoscopic and microbiological findings in acute salpingitis: report on a Unit Kingdom cohort. Br J Obstet Gynaecol. 1995; 102(5): 407-14.	1989-1993	*
Simms I, Stephenson JM, Mallinson H, Peeling RW, Thomas K, Gokhale R, Rogers PA, Hay P, Oakeshott P, Hopwood J, Birley H, Hernon M. Ri factors associated with pelvic inflammatory disease. Sex Transm Infect. 2006; 82(6): 452-7.	2000-2002	*
Office for National Statistics. Social Survey Division, Mental Health of Young People Looked After by Local Authorities in Great Britain, 2001-2003[computer file]. Colchester, Essex: UK Data Archive [distributor], December 2005. SN: 5280 , http://dx.doi.org/10.5255/UKDA-SN-5280-1	2001-2003	*
Office for National Statistics, ONS Survey of Psychiatric Morbidity among Prisoners in England and Wales, 1997 [computer file]. Colchester, Esse UK Data Archive [distributor], February 2001. SN: 4320, http://dx.doi.org/10.5255/UKDA-SN-4320-1	1997	*
Evans JR, Fletcher AE, Wormald RPL. Age-related macular degeneration causing visual impairment in people 75 years or older in Britain: an add-on study to the Medical Research Council Trial of Assessment and Management of Older People in the Community. Ophthalmology. 2004; 111(3): 513-7.	1995-2000	
United Kingdom - England and Wales National Congenital Anomaly System Data 1993-1998 - WHO as it appears in the European Surveillance of Congenital Anomalies (EUROCAT), International Centre on Birth Defects, World Health Organization (WHO). World Atlas of Birth Defects. 2nd ed. Geneva, Switzerland: World Health Organization (WHO), 2003.	1993-1998	*
Morris-Cunnington M, Brown D, Pimenta J, Robinson NJ, Miller E. New estimates of herpes simplex virus type 2 seroprevalence in England: "higl but stable seroprevalence over the last decade. Sex Transm Dis. 2004; 31(4): 243-6.	1991, 2000	*
Narouz N, Allan PS, Wade AH, Wagstaffe S. Genital herpes serotesting: a study of the epidemiology and patients' knowledge and attitude among ST clinic attenders in Coventry, UK. Sex Transm Infect. 2003; 79(1): 35-41.	1993-2002	*
Evans BA, Kell PD, Bond RA, MacRae KD, Slomka MJ, Brown DWG. Predictors of seropositivity to herpes simplex virus type 2 in women. Int J ST AIDS. 2003; 14(1): 30-6.	1992, 2000-2002	*
Opaneye AA, Bashford J. Seroprevalence of antibodies to herpes simplex virus types 1 and 2 among two sexually active female populations Middlesbrough, England. J R Soc Promot Health. 2002; 122(2): 108-11.	1992, 1996-1997	*
Yates HL, McCullough S, Harrison C, Gill AB. Hypoxic ischaemic encephalopathy: accuracy of the reported incidence. Arch Dis Child Fetal Neona Ed. 2012; 97(1): F77-78.	2006-2009	*
Yeh P, Emary K, Impey L. The relationship between umbilical cord arterial pH and serious adverse neonatal outcome: analysis of 51,519 consecuti validated samples. BJOG. 2012; 119(7): 824-31.	1991-2009	*
Becher J-C, Stenson BJ, Lyon AJ. Is intrapartum asphyxia preventable? BJOG. 2007; 114(11): 1442-4	1994-2005	*
Hull J, Dodd KL. Falling incidence of hypoxic-ischaemic encephalopathy in term infants. Br J Obstet Gynaecol. 1992; 99(5): 386-91.	1984-1988	*
Levene ML, Kornberg J, Williams TH. The incidence and severity of post-asphyxial encephalopathy in full-term infants. Early Hum Dev. 1985; 11(21-6.	1980-1983	*
Smith J, Wells L, Dodd K. The continuing fall in incidence of hypoxic-ischaemic encephalopathy in term infants. BJOG. 2000; 107(4): 461-6.	1992-1996	*
Azzopardi DV, Strohm B, Edwards AD, Dyet L, Halliday HL, Juszczak E, Kapellou O, Levene M, Marlow N, Porter E, Thoresen M, Whitelaw A, Brocklehurst P, TOBY Study Group. Moderate hypothermia to treat perinatal asphyxial encephalopathy. N Engl J Med. 2009; 361(14): 1349-58.	2002-2006	*
Barnett A, Mercuri E, Rutherford M, Haataja L, Frisone MF, Henderson S, Cowan F, Dubowitz L. Neurological and perceptual-motor outcome at 5 - 6 years of age in children with neonatal encephalopathy: relationship with neonatal brain MRI. Neuropediatrics. 2002; 33(5): 242-8.	1991-1996	*
Williams L, Parker H. Alcohol, cannabis, ecstasy and cocaine: Drugs of reasoned choice amongst young adult recreational drug users in England. In Drug Policy. 1999; 12(5-6): 397-413.	1995, 1999	*
United Kingdom - England and Wales National Congenital Anomaly System Data 2001 - ICBDMS as it appears in the International Clearinghouse for Birth Defects Monitoring Systems. International Clearinghouse for Birth Defects Monitoring Systems Annual Report 2003. Rome, Italy: International Centre on Birth Defects.	2001	*
Song SH, Gray TA. Early-onset type 2 diabetes: high risk for premature diabetic retinopathy. Diabetes Res Clin Pract. 2011; 94(2): 207-11.	2008-2010	
Abbott CA, Carrington AL, Ashe H, Bath S, Every LC, Griffiths J, Hann AW, Hussein A, Jackson N, Johnson KE, Ryder CH, Torkington R, Van Ross ERE, Whalley AM, Widdows P, Williamson S, Boulton AJM, North-West Diabetes Foot Care Study. The North-West Diabetes Foot Care Study: incidence of, and risk factors for, new diabetic foot ulceration in a community-based patient cohort. Diabet Med. 2002; 19(5): 377-84.	1994-1996	

Canavan RJ, Unwin NC, Kelly WF, Connolly VM. Diabetes- and nondiabetes-related lower extremity amputation incidence before and after the introduction of better organized diabetes foot care: continuous longitudinal monitoring using a standard method. Diabetes Care. 2008; 31(3): 459-63.	1995-2000	
Cohen DL, Neil HA, Thorogood M, Mann JI. A population-based study of the incidence of complications associated with type 2 diabetes in the elder Diabet Med. 1991; 8(10): 928-33.	1982-1988	
Vanderpump MP, Tunbridge WM, French JM, Appleton D, Bates D, Rodgers H, Evans JG, Clark F, Tunbridge F, Young ET. The incidence of diabete mellitus in an English community: a 20-year follow-up of the Whickham Survey. Diabet Med. 1996; 13(8): 741-7.	1992-1993	
Button EJ, Chadalavada B, Palmer RL. Mortality and predictors of death in a cohort of patients presenting to an eating disorders service. Int J E Disord. 2010; 43(5): 387-92.	1992-2007	*
Crisp A. 1.6. Death, survival and recovery in anorexia nervosa: a thirty five year study. Eur Eat Disord Rev. 2006; 14(3): 168-75.	1960-1990	*
Collings S, King M. Ten-year follow-up of 50 patients with bulimia nervosa. Br J Psychiatry. 1994; 164(1): 80-'	1981-1991	*
Fairburn CG, Cooper Z, Doll HA, Norman P, O'Connor M. The natural course of bulimia nervosa and binge eating disorder in young women. Arch Ge Psychiatry. 2000; 57(7): 659-65.	1993-1998	*
Bargagli AM, Hickman M, Davoli M, Perucci CA, Schifano P, Buster M, Brugal T, Vicente J, COSMO European Group. Drug-related mortality and i impact on adult mortality in eight European countries. Eur J Public Health. 2006; 16(2): 198-202.	1997-2001	*
De Angelis D, Hickman M, Yang S. Estimating long-term trends in the incidence and prevalence of opiate use/injecting drug use and the number of former users: back-calculation methods and opiate overdose deaths. Am J Epidemiol. 2004; 160(10): 994-1004.	2000	*
Frischer M, Hickman M, Kraus L, Mariani F, Wiessing L. A comparison of different methods for estimating the prevalence of problematic drug misu in Great Britain. Addiction. 2001; 96(10): 1465-76.	1996	*
Ghodse H, Oyefeso A, Kilpatrick B. Mortality of drug addicts in the United Kingdom 1967-1993. Int J Epidemiol. 1998; 27(3): 473-8.	1967-1993	*
Oppenheimer E, Tobutt C, Taylor C, Andrew T. Death and survival in a cohort of heroin addicts from London clinics: a 22-year follow-up stud Addiction. 1994; 89(10): 1299-308.	1969-1991	*
Rathod NHR, Addenbrooke WM, Rosenbach AF. Heroin dependence in an English town: 33-year follow-up. Br J Psychiatry. 2005; 187(5): 421-5.	1966-1999	*
Erdohazi M, Highman W. Glucose-6-phosphate-dehydrogenase deficiency in Britain. Lancet. 1962; 280(7268): 127	1960-1962	
Bebbington P, Jonas S, Kuipers E, King M, Cooper C, Brugha T, Meltzer H, McManus S, Jenkins R. Childhood sexual abuse and psychosis: data fro a cross-sectional national psychiatric survey in England. Br J Psychiatry. 2011; 199(1): 29-37.	2006-2007	*
United Kingdom - England and Wales National Congenital Anomaly System Data 2003 - ICBDSR as it appears in International Clearinghouse for Birth Defects Surveillance and Research. International Clearinghouse for Birth Defects Surveillance and Research Annual Report 2005. Rome, Italy: International Clearinghouse for Birth Defects Surveillance and Research, 2007.	2003	*
United Kingdom - England and Wales National Congenital Anomaly System Data 2004 - ICBDSR as it appears in International Clearinghouse for Birth Defects Surveillance and Research. International Clearinghouse for Birth Defects Surveillance and Research Annual Report 2006. Rome, Italy: International Clearinghouse for Birth Defects Surveillance and Research, 2007.	2004	*
United Kingdom - Wessex Antenatally Detected Anomalies Register Data 2007 - ICBDSR as it appears in International Clearinghouse for Birth Defec Surveillance and Research. International Clearinghouse for Birth Defects Surveillance and Research Annual Report 2009. Rome, Italy: International Clearinghouse for Birth Defects Surveillance and Research.	2007	*
United Kingdom - Wessex Antenatally Detected Anomalies Register Data 2008 - ICBDSR as it appears in International Clearinghouse for Birth Defec Surveillance and Research. International Clearinghouse for Birth Defects Surveillance and Research Annual Report 2010. Rome, Italy: International Clearinghouse for Birth Defects Surveillance and Research, 2011.	2008	*
Demography of dialysis and transplantation in Europe in 1985 and 1986: trends over the previous decade. Report from the European Dialysis ar Transplant Association Registry. Nephrol Dial Transplant. 1988; 3(6): 714-27.	1985	
Van Dijk PC, Jager KJ, Stengel B, Grönhagen-Riska C, Feest TG, Briggs JD. Renal replacement therapy for diabetic end-stage renal disease: data fro 10 registries in Europe (1991-2000). Kidney Int. 2005; 67(4): 1489-99.	1991-1992, 1999-2000	
Trends in the incidence of renal replacement therapy for end-stage renal disease in Europe, 1990-1999. Trends in the incidence of renal replacement therapy for end-stage renal disease in Europe, 1990-1999. Nephrol Dial Transplant. 2003; 18(9): 1824-33.	1990-1991, 1998-1999	
European Surveillance of Congenital Anomalies (EUROCAT). United Kingdom EUROCAT Prevalence Tables, Individual Registries and Countrie Newtownabbey, Northern Ireland: European Surveillance of Congenital Anomalies (EUROCAT).	1980-2011	
Global Lower Extremity Amputation Study Group. Epidemiology of lower extremity amputation in centres in Europe, North America and East As The Global Lower Extremity Amputation Study Group. Br J Surg. 2000; 87(3): 328-37.	1995-1997	
Schofield CJ, Yu N, Jain AS, Leese GP. Decreasing amputation rates in patients with diabetes-a population-based study. Diabet Med. 2009; 26(8): 77 7.	2000-2006	
Leggetter S, Chaturvedi N, Fuller JH, Edmonds ME. Ethnicity and risk of diabetes-related lower extremity amputation: a population-based, case-contro study of African Caribbeans and Europeans in the United kingdom. Arch Intern Med. 2002; 162(1): 73-8.	1992-1997	
Michelmore KF, Balen AH, Dunger DB, Vessey MP. Polycystic ovaries and associated clinical and biochemical features in young women. Cl Endocrinol (Oxf). 1999; 51(6): 779-86.	1996-1997	
Cleland JGF, Swedberg K, Follath F, Komajda M, Cohen-Solal A, Aguilar JC, Dietz R, Gavazzi A, Hobbs R, Korewicki J, Madeira HC, Moiseyev VS, Preda I, Gilst WH van, Widimsky J, Freemantle N, Eastaugh J, Mason J. The EuroHeart Failure survey programme – a survey on the quality of care among patients with heart failure in Europe Part 1: patient characteristics and diagnosis. Eur Heart J. 2003; 24(5): 442-63.	2000-2001	
Parkin DM, International Agency for Research on Cancer, International Association of Cancer Registries. Cancer incidence in five continents. Volum I to VIII. Lyon, France, IARCPress, 2005.	1985	
United Kingdom - England and Wales Congenital Malformation Monitoring Program Data 1981 - ICBDMS as it appears in International Clearinghous for Birth Defects Monitoring Systems. Congenital Malformations Worldwide: A Report from the International Clearinghouse for Birth Defects Monitoring Systems. Amsterdam, Netherlands: Elsevier, 1991.	1981	*
Morris AJ, Steele J, White DA. The oral cleanliness and periodontal health of UK adults in 1998. Br Dent J. 2001; 191(4): 186-92.	1998	
United Kingdom National Diet and Nutrition Survey: Young People Aged 4 to 18 Years 1997 as it appears in Global Dietary Database Consortium, Nutrition and Chronic Disease Expert Group (NutriCoDE). Global Dietary Database 1980-2011. [Unpublished].	1997	
United Kingdom National Diet and Nutrition Survey: People Aged 65 Years and Over 1994-1995 as it appears in Global Dietary Database Consortium Nutrition and Chronic Disease Expert Group (NutriCoDE). Global Dietary Database 1980-2011. [Unpublished].	1994-1995	
United Kingdom National Diet, Nutrition, and Dental Survey of Children Aged 1 1/2 to 4 1/2 Years 1992-1993 as it appears in Global Dietary Database Consortium, Nutrition and Chronic Disease Expert Group (NutriCoDE). Global Dietary Database 1980-2011. [Unpublished].	1992-1993	
United Kingdom Low Income Diet and Nutrition Survey 2003-2005 as it appears in Global Dietary Database Consortium, Nutrition and Chroni Disease Expert Group (NutriCoDE). Global Dietary Database 1980-2011. [Unpublished].	2003-2005	
National Centre for Social Research (NatCen), University of Leicester. Adult Psychiatric Morbidity in England, 2007: Results of a Household Surve Leeds, England: Information Centre for Health and Social Care, NHS, 2009.	2007	
Office for National Statistics (United Kingdom). United Kingdom General Lifestyle Survey 2010 - ONS. Newport, United Kingdom: Office for Natior Statistics (United Kingdom), 2012.	2010	
Nicholls P, Edwards G, Kyle E. Alcoholics admitted to four hospitals in England. II. General and cause-specific mortality. Q J Stud Alcohol. 197 35(3): 841-55.	1953-1967	
Hirst C, Swingler R, Compston DA, Ben-Shlomo Y and Robertson NP. Survival and cause of death in multiple sclerosis: a prospective populatio based study. J Neurol Neurosurg Psychiatry. 2008; 79(9): 1016-21.	1985-2005	*
Roberts SE, Williams JG, Meddings D, Goldacre MJ. Incidence and case fatality for acute pancreatitis in England: geographical variation, social deprivation, alcohol consumption and aetiology – a record linkage study. Aliment Pharmacol Ther. 2008; 28(7): 931-41.	1998-2003	*
Walker RW, Hand A, Jones C, Wood BH, Gray WK. The prevalence of Parkinson's disease in a rural area of North-East England. Parkinsonism Rel Disord. 2010; 16(9): 572-5.	2007-2008	*
Yarnell JW, Voyle GJ, Sweetnam PM, Milbank J, Richards CJ, Stephenson TP. Factors associated with urinary incontinence in women. J Epidemi Community Health. 1982; 36(1): 58-63.	1979	
Walsh K, Cruddas M, Coggon D. Low back pain in eight areas of Britain. J Epidemiol Community Health. 1992; 46(3): 227-3	1989	*
Goldwater LJ, Hoover AW. An international study of "normal" levels of lead in blood and urine. Arch Environ Health. 1967; 15(1): 60-3.	1964	
Thomas VM, Socolow RH, Fanelli JJ, Spiro TG. Effects of Reducing Lead in Gasoline:? An Analysis of the International Experience. Environ S Technol. 1999; 33(22): 3942-8.	1979-1980, 1984, 1995	

Quinn MJ, Delves HT. UK Blood Lead Monitoring Programme 1984-1987: protocol and results for 1984. Hum Toxicol. 1987; 6(6): 459-74.	1984	
O'Donohoe J, Chalkley S, Richmond J, Barltrop D. Blood lead in U.K. children--time for a lower action level? Clin Sci (Lond). 1998; 95(2): 219-23.	1991	
Lewendon G, Kinra S, Nelder R, Cronin T. Should children with developmental and behavioural problems be routinely screened for lead? Arch D Child. 2001; 85(4): 286-8.	1998	
Dennison EM, Syddall HE, Sayer AA, Martin HJ, Cooper C. Lipid profile, obesity and bone mineral density: the Hertfordshire Cohort Study. QJI 2007; 100(5): 297-303.	1998-2003	
Kaptoge S, Reid D, Scheidt-Nave C, Poor G, Pols HA., Khaw K., Felsenberg D, Benevolenskaya L, Diaz M, Stepan J, Eastell R, Boonen S, Cannata J Glueer C, Crabtree N, Kaufman J, Reeve J. Geographic and other determinants of BMD change in European men and women at the hip and spine. A population-based study from the Network in Europe for Male Osteoporosis (NEMO). Bone. 2007; 40(3): 662-73.	2004	
Lunt M, Felsenberg D, Adams J, Benevolenskaya L, Cannata J, Dequeker J, Dodenhof C, Falch JA, Johnell O, Khaw KT, Masaryk P, Pols H, Poor G, Reid D, Scheidt-Nave C, Weber K, Silman AJ, Reeve J. Population-based geographic variations in DXA bone density in Europe: the EVOS Study. European Vertebral Osteoporosis. Osteoporos Int. 1997; 7(3): 175-89.	1994	
Macdonald HM, McGuigan FE, Stewart A, Black AJ, Fraser WD, Ralston S, Reid DM. Large-scale population-based study shows no evidence of association between common polymorphism of the VDR gene and BMD in British women. J Bone Miner Res. 2006; 21(1): 151-62.	1997-2000	
Petley GW, Cotton AM, Murrills AJ, Taylor PA, Cooper C, Cawley MI, Wilkin TJ. Reference ranges of bone mineral density for women in southe England: the impact of local data on the diagnosis of osteoporosis. Br J Radiol. 1996; 69(823): 655-60.	1993	
Spector TD, McCloskey EV, Doyle DV, Kanis JA. Prevalence of vertebral fracture in women and the relationship with bone density and symptoms: t Chingford Study. J Bone Miner Res. 1993; 8(7): 817-22.	1989-1991	
Trivedi DP, Khaw KT. Bone mineral density at the hip predicts mortality in elderly men. Osteoporos Int. 2001; 12(4): 259-6	1991-1999	
Truscott JG, Simpson DS, Fordham JN. A suggested methodology for the construction of national bone densitometry reference ranges: 1372 Caucasia women from four UK sites. Br J Radiol. 1997; 70(840): 1245-51.	1994	
Haberman S, Benjamin B, Capildeo R, Rose FC. North West Thames registry of neurological disease. J R Soc Med. 1982; 75(6): 443-9.	1978	
Hughes RA, Charlton J, Latinovic R, Gulliford MC. No association between immunization and Guillain-Barré syndrome in the United Kingdom, 19 to 2000. Arch Intern Med. 2006; 166(12): 1301-4.	1992-2000	
Rees JH, Thompson RD, Smeeton NC, Hughes RA. Epidemiological study of Guillain-Barré syndrome in south east England. J Neurol Neurosu Psychiatry. 1998; 64(1): 74-7.	1993-1994	
Sridharan GV, Tallis RC, Gautam PC. Guillain-Barré syndrome in the elderly. A retrospective comparative study. Gerontology. 1993; 39(3): 170-5.	1980-1988	
Winner SJ, Evans JG. Guillain-Barré syndrome in Oxfordshire: clinical features in relation to age. Age Ageing. 1993; 22(3): 164-70.	1990-1992	
Winner SJ, Evans JG. Age specific incidence of Guillain-Barre syndrome in Oxfordshire. Q J Med. 1990; 77(3): 1297-130	1974-1986	
Reddy S, Rangaiah J, Addiman S, Wareham D, Wilson P, Sefton A. Epidemiology, antibiotic resistance trends and the cost of enteric fever in Ea London, 2005-2010. Travel Med Infect Dis. 2011; 9(4): 206-12.	2006-2009	
Wagner KS, White JM, Lucenko I, Mercer D, Crowcroft NS, Neal S, Efstratiou A, Diphtheria Surveillance Network. Diphtheria in the postepidem period, Europe, 2000-2009. Emerg Infect Dis. 2012; 18(2): 217-25.	2000-2009	*
Department of Peace and Conflict Research, Uppsala University. UCDP Battle-Related Deaths Database Version 5, 2013. Uppsala, Sweden Department of Peace and Conflict Research, Uppsala University, 2013.	1989-2003	
Pugsley Z, Ballard K. Management of endometriosis in general practice: the pathway to diagnosis. Br J Gen Pract. 2007; 57(539): 470-6.	2004	
Ballard KD, Seaman HE, de Vries CS, Wright JT. Can symptomatology help in the diagnosis of endometriosis? Findings from a national case-contr study – Part I. BJOG. 2008; 115(11): 1382-91.	1992-2001	
Cox, B.D., Health and Lifestyle Survey, 1984-1985 [computer file]. Colchester, Essex: UK Data Archive [distributor], October 1988. SN: 2218 http://dx.doi.org/10.5255/UKDA-SN-2218-1.	1984-1985	
Cox, B.D., Health and Lifestyle Survey: Seven-Year Follow-Up, 1991-1992 [computer file]. Colchester, Essex: UK Data Archive [distributor], Januar 1995. SN: 3279 , http://dx.doi.org/10.5255/UKDA-SN-3279-1	1991-1992	
Office for National Statistics. Social Survey Division, Adult Dental Health Survey, 1998 [computer file]. 2nd Edition. Colchester, Essex: UK Da Archive [distributor], October 2013. SN: 4226 , http://dx.doi.org/10.5255/UKDA-SN-4226-2	1998	
Davis A, Wood S, Healy R, Webb H, Rowe S. Risk factors for hearing disorders: epidemiologic evidence of change over time in the UK. J Am Ac Audiol. 1995; 6(5): 365-70.	1983-1988	*
Fortnum HM, Marshall DH, Summerfield AQ. Epidemiology of the UK population of hearing-impaired children, including characteristics of those with and without cochlear implants – audiology, aetiology, comorbidity and affluence. Int J Audiol. 2002; 41(3): 170-9.	1980-1998	*
Parving A, Stephens D. Profound permanent hearing impairment in childhood: causative factors in two European countries. Acta Otolaryngol. 199 117(2): 158-60.	1975-1980	*
Watkin P, Hasan J, Baldwin M, Ahmed M. Neonatal hearing screening: Have we taken the right road? Results from a 10-year targeted scre longitudinally followed up in a single district. Audiol Med. 2005; 3(3).	1990-2000	*
European Union Agency for Fundamental Rights. European Union Violence Against Women Study 201	2012	*
Health Protection Agency (United Kingdom), Joint United Nations Program on HIV/AIDS (UNAIDS). United Kingdom Global AIDS Response Repo 2012. Geneva, Switzerland: Joint United Nations Program on HIV/AIDS (UNAIDS), 2012.	2010	*
Matthews F, Brayne C, Medical Research Council Cognitive Function and Ageing Study Investigators. The incidence of dementia in England a Wales: findings from the five identical sites of the MRC CFA Study. PLoS Med. 2005; 2(8): e193.	1990-1996	
Paykel ES, Brayne C, Huppert FA, Gill C, Barkley C, Gehlhaar E, Beardsall L, Girling DM, Pollitt P, O'Connor D. Incidence of dementia in population older than 75 years in the United Kingdom. Arch Gen Psychiatry. 1994; 51(4): 325-32.	1985-1990	
Copeland JR, Dewey ME, Davidson IA, Saunders PA, Scott A. Geriatric Mental State-AGECAT: prevalence, incidence and long-term outcome of dementia and organic disorders in the Liverpool study of continuing health in the community. Neuroepidemiology. 1992; 84-7.	1982-1985	
Brayne C, Best N, Muir M, Richards SJ, Gill C. Five-year incidence and prediction of dementia and cognitive decline in a population sample of wom aged 70-79 at baseline. Int J Geriatr Psychiatry. 1997; 12(11): 1107-18.	1994-1996	
O'Connor DW, Pollitt PA, Hyde JB, Fellows JL, Miller ND, Brook CP, Reiss BB, Roth M. The prevalence of dementia as measured by the Cambridg Mental Disorders of the Elderly Examination. Acta Psychiatr Scand. 1989; 79(2): 190-8.	1983-1986	
United Kingdom - England and Wales Vital Registration - Deaths 2011 ICD10 as it appears in World Health Organization (WHO). WHO Mortalit Database Version February 2014. Geneva, Switzerland: World Health Organization (WHO), 2014.	2011	
Child Abuse Studies Unit, University of North London. United Kingdom - Great Britain Child Sexual Abuse Prevalence Survey 1989.	1989	
Baker AW, Duncan SP. Child sexual abuse: A study of prevalence in Great Britain. Child Abuse Negl. 1985; 9(4): 457-6	1982	
Gallagher B, Bradford M, Pease K. Attempted and completed incidents of stranger-perpetrated child sexual abuse and abduction. Child Abuse Neg 2008; 32(5): 517-28.	1996-1997	
May-Chahal C, Cawson P. Measuring child maltreatment in the United Kingdom: a study of the prevalence of child abuse and neglect. Child Abu Negl. 2005; 29(9): 969-84.	1998-1999	
European Centre for Disease Prevention and Control, WHO Regional Office for Europe (EURO-WHO). HIV/AIDS Surveillance in Europe 2012 Stockholm, Sweden: European Centre for Disease Prevention and Control, 2012.	2006-2012	*
Kuo C-F, Grainge MJ, Mallen C, Zhang W, Doherty M. Rising burden of gout in the UK but continuing suboptimal management: a nationwi population study. Ann Rheum Dis. 2014.	1997-2012	*
Sanmartin J, Queen Sofia Center for the Study of Violence. Partner Violence against Women: Statistics and Legislation, 2nd International Report 200 Valencia, Spain: Queen Sofia Center for the Study of Violence, 2007.	2003	
Sanmartin J, Molina A, Garcia Y, Queen Sofia Center for the Study of Violence. Partner Violence against Women: Statistics and Legislatio International Report 2003. Valencia, Spain: Queen Sofia Center for the Study of Violence, 2003.	2000	
Queen Sofia Center for the Study of Violence. Partner Violence Against Women: Statistics and Legislation, 3rd International Report 2010. Valenci Spain: Queen Sofia Center for the Study of Violence, 2010.	2006	
Gregory MJ, Milroy CM. Homicide and Suicide in Yorkshire and the Humber: 1975-1992 and 1993-2007. Am J Forensic Med Pathol. 2010; 31(1): 58 63.	2006-2007	
European Environment Agency (EEA). EEA Airbase European Air Quality Database Version 7, 2013. Copenhagen, Denmark: European Environmen Agency (EEA), 2013.	2008-2011	*
Convention on Long-Range Transboundary Air Pollution, United Nations Economic Commission for Europe. EBAS Database EMEP Framework European Monitoring and Evaluation Programme PM2.5 and PM10 Data 1999-2014.	2008-2011	*
World Health Organization (WHO). Progress towards the elimination of leprosy as a public health problem. Part I. Wkly Epidemiol Rec. 1995: 70(25 177-82.	1994	*
Health Protection Agency (United Kingdom). Tuberculosis in the UK: Report on Tuberculosis Surveillance in the UK 2010. London, England: Heal Protection Agency (United Kingdom), 2010.	2001, 2004, 2007	*

Ades AE, Peckham CS, Dale GE, Best JM, Jeansson S. Prevalence of antibodies to herpes simplex virus types 1 and 2 in pregnant women, an estimated rates of infection. J Epidemiol Community Health. 1989; 43(1): 53-60.	1980-1981	*
Cowan FM, Johnson AM, Ashley R, Corey L, Mindel A. Antibody to herpes simplex virus type 2 as serological marker of sexual lifestyle populations. BMJ. 1994; 309(6965): 1325-9.	1990-1992	*
Jha PK, Beral V, Peto J, Hack S, Hermon C, Deacon J, Mant D, Chilvers C, Vessey MP, Pike MC. Antibodies to human papillomavirus and to othe genital infectious agents and invasive cervical cancer risk. Lancet. 1993; 341(8853): 1116-8.	1990	*
Pebody RG, Andrews N, Brown D, Gopal R, De Melker H, François G, Gatcheva N, Hellenbrand W, Jokinen S, Klavs I, Kojouharova M, Kortbeek T, Kriz B, Prosenc K, Roubalova K, Teocharov P, Thierfelder W, Valle M, Van Damme P, Vranckx R. The seroepidemiology of herpes simplex virus type 1 and 2 in Europe. Sex Transm Infect. 2004; 80(3): 185-91.	1994	*
Khawaja AP, Chan MPY, Hayat S, Broadway DC, Luben R, Garway-Heath DF, Sherwin JC, Yip JLY, Dalzell N, Wareham NJ, Khaw K-T, Foster PJ. The EPIC-Norfolk Eye Study: rationale, methods and a cross-sectional analysis of visual impairment in a population-based cohort. BMJ Open. 2013; 3(3).	2004-2011	*
World Health Organization (WHO). WHO Global Health Observatory - Cholera: Number of Reported Cases by Country. Geneva, Switzerland: Wor Health Organization (WHO).	2000-2012	*
Wagner KS, White JM, Crowcroft NS, De Martin S, Mann G, Efstratiou A. Diphtheria in the United Kingdom, 1986-2008: the increasing role o Corynebacterium ulcerans. Epidemiol Infect. 2010; 138(11): 1519-30.	1986-2008	
National Centre for Social Research and National Foundation for Educational Research, Smoking, Drinking and Drug Use among Young People, 2012 [computer file]. Colchester, Essex: UK Data Archive [distributor], October 2013. SN: 7393, http://dx.doi.org/10.5255/UKDA-SN-7393-1	2012	*
Fogarty AW, Lewis SA, McKeever TM, Britton JR. Is higher sodium intake associated with elevated systemic inflammation? A population-based study. Am J Clin Nutr. 2009; 89(6): 1901-4. as it appears in Global Dietary Database Consortium, Nutrition and Chronic Disease Expert Group (NutriCoDE) Global Dietary Database 1980-2011. [Unpublished].	1991	
Khaw K-T, Bingham S, Welch A, Luben R, O'Brien E, Wareham N, Day N. Blood pressure and urinary sodium in men and women: the Norfolk Cohort of the European Prospective Investigation into Cancer (EPIC-Norfolk). Am J Clin Nutr. 2004; 80(5): 1397-403. as it appears in Global Dietary Database Consortium, Nutrition and Chronic Disease Expert Group (NutriCoDE). Global Dietary Database 1980-2011. [Unpublished].	1993-1997	
Garraway WM, Russell EB, Lee RJ, Collins GN, McKelvie GB, Hehir M, Rogers AC, Simpson RJ. Impact of previously unrecognized benign prostatic hyperplasia on the daily activities of middle-aged and elderly men. Br J Gen Pract. 1993; 43(373): 318-21.	1990	
Ng Y, Shaw D, Fielder A, Levene M. Epidemiology of retinopathy of prematurity. Lancet. 1988; 332(8622): 1235-:	1986	*
Fortnum HM, Davis AC. Epidemiology of bacterial meningitis. Arch Dis Child. 1993; 68(6): 763-7	1980-1989	
Holt D, Halket S, de Louvois J, Harvey D. Neonatal meningitis in England and Wales: 10 years on. Arch Dis Child Fetal Neonatal Ed. 2001; 84(2): F8 F89.	1996-1997	
De Louvois J, Blackbourn J, Hurley R, Harvey D. Infantile meningitis in England and Wales: a two year study. Arch Dis Child. 1991; 66(5): 603-7.	1985-1987	
Department of Health (United Kingdom), Office of Population Censuses and Surveys (United Kingdom), Royal College of General Practitioners. Morbidity Statistics from General Practice: Fourth National Study 1991-1992. London, United Kingdom: Her Majesty's Stationery Office (HMSO), 1995.	1991-1992	
Gauthier A, Breuer J, Carrington D, Martin M, Rémy V. Epidemiology and cost of herpes zoster and post-herpetic neuralgia in the United Kingdor Epidemiol Infect. 2009; 137(1): 38-47.	2000-2006	
Fleming DM, Cross KW, Cobb WA, Chapman RS. Gender difference in the incidence of shingles. Epidemiol Infect. 2004; 132(1): 1-5.	1994-2001	
Hope-Simpson RE. The Nature of Herpes Zoster: A Long-term Study and a New Hypothesis. Proc R Soc Med. 1965; 58(1): 9-20.	1950-1962	
Glynn C, Crockford G, Gavaghan D, Cardno P, Price D, Miller J. Epidemiology of Shingles. J R Soc Med. 1990; 83(10): 617-'	1978-1986	
Ross CAC, Brown WK, Clarke A, Caldwell WF, Gordon ER, Harvey J, McAlister AMT, McGlone J, Prentice RTW, Thorburn W, Tobias C. Herpe zoster in general practice. J R Coll Gen Pract. 1975; 25(150): 29-32.	1972-1973	
Fairley CK, Miller E. Varicella-zoster virus epidemiology – A changing scene? J Infect Dis. 1996; 174(Suppl 3): S314-1!	1980-1989	
Wilson JB. Thirty one years of herpes zoster in a rural practice. Br Med J (Clin Res Ed). 1986; 293(6558): 1349-51	1955-1985	
Cunningham R, Mutton K. Dengue haemorrhagic fever. BMJ. 1991; 302(6784): 1083-4	1990	
European Community Respiratory Health Survey Study Group. European Community Respiratory Health Survey 1992-2003 as provided by the Glob Burden of Disease 2010 Respiratory Disease Expert Group. [Unpublished].	1992-1994, 1998-2003	
Global Allergy and Asthma European Network (GA²LEN). Global Allergy and Asthma European Network Collaboration Center Data 2007-2008 a provided by the Global Burden of Disease 2010 Respiratory Disease Expert Group. [Unpublished].	2008	
United Kingdom - England UK Renal Registry (UKRR) Renal Replacement Therapy Data 2010 as it appears in European Renal Association-European Dialysis and Transplant Association (ERA-EDTA). European Renal Association-European Dialysis and Transplant Association Registry Annual Repo 2010. Parma, Italy: European Renal Association-European Dialysis and Transplant Association (ERA-EDTA), 2012.	2010	
United Kingdom - England UK Renal Registry (UKRR) Renal Replacement Therapy Data 2011 as it appears in European Renal Association-European Dialysis and Transplant Association (ERA-EDTA). European Renal Association-European Dialysis and Transplant Association Registry Annual Repo 2011. Parma, Italy: European Renal Association-European Dialysis and Transplant Association (ERA-EDTA), 2013.	2011	
Davison KL, Crowcroft NS, Ramsay ME, Brown DWG, Andrews NJ. Viral encephalitis in England, 1989-1998: what did we miss? Emerg Infect Di 2003; 9(2): 234-40.	1989-1998	
Cohn M, Stewart P. Prevalence of potential pathogens in cervical canal before termination of pregnancy. BMJ. 1992; 304(6840): 1479.	1992	
Wright D, Barrow S, Fisher AD, Horsley SD, Jayson MI. Influence of physical, psychological and behavioural factors on consultations for back pain. J Rheumatol. 1995; 34(2): 156-61.	1992	*
World Health Organization (WHO). Mental Illness in General Health Care: An International Study. Geneva, Switzerland: World Health Organizatio (WHO), 1995.	1991-1992	
Kendell RE, Staton MC. The fate of untreated alcoholics. Q J Stud Alcohol. 1966; 27(1): 30-41	1950-1970	
Adelstein A, White G. Alcoholism and mortality. Popul Trends. 1976; 6: 13-Ju	1953-1974	
Marshall EJ, Edwards G, Taylor C. Mortality in men with drinking problems: a 20-year follow-up. Addiction. 1994; 89(10): 1293-8.	1968-1990	
Plant M. Women, Drinking, and Pregnancy. London, United Kingdom: Tavistock Publications, 198	1980-1996, 1998-2008	*
Van Veldhuisen DJ, Dickstein K, Cohen-Solal A, Lok DJA, Wasserman SM, Baker N, Rosser D, Cleland JGF, Ponikowski P. Randomized, double-blind, placebo-controlled study to evaluate the effect of two dosing regimens of darbepoetin alfa in patients with heart failure and anaemia. Eur Heart J. 2007; 28(18): 2208-16.	1992-1995	
EUVAC-NET. EUVAC-NET Pertussis Surveillance Report 2008. Stockholm, Sweden: European Centre for Disease Prevention and Control, 2009.	2008	
EUVAC-NET. EUVAC-NET Pertussis Surveillance Report 2003-2007. Stockholm, Sweden: European Centre for Disease Prevention and Control 2009.	2003-2007	
EUVAC-NET. EUVAC-NET Pertussis Surveillance Report 2009. Stockholm, Sweden: European Centre for Disease Prevention and Control, 2010.	2009	
United Nations Statistics Division (UNSD). United Nations Demographic Yearbook - Historical Supplement 1997. New York City, United States United Nations Statistics Division (UNSD).	1990	
Margolis DJ, Bilker W, Knauss J, Baumgarten M, Strom BL. The incidence and prevalence of pressure ulcers among elderly patients in general medic practice. Ann Epidemiol. 2002; 12(5): 321-5.	1988-1996	*
Flohr C, Weiland SK, Weinmayr G, Björkstén B, Bråbäck L, Brunekreef B, Büchele G, Clausen M, Cookson WOC, von Mutius E, Strachan DP, Williams HC, ISAAC Phase Two Study Group. The role of atopic sensitization in flexural eczema: findings from the International Study of Asthma and Allergies in Childhood Phase Two. J Allergy Clin Immunol. 2008; 121(1): 141-147.	2005-2007	*
Gelfand JM, Weinstein R, Porter SB, Neimann AL, Berlin JA, Margolis DJ. Prevalence and treatment of psoriasis in the United Kingdom: a populatio based study. Arch Dermatol. 2005; 141(12): 1537-41.	1987-2002	*
EUVAC-NET. EUVAC-NET Measles Surveillance Report 2010. Stockholm, Sweden: European Centre for Disease Prevention and Control, 2011.	2010	
World Health Organization (WHO). Measles surveillance in 1989. Wkly Epidemiol Rec. 1990; 65(15): 112-3	1989	*
EUVAC-NET. EUVAC-NET Measles Surveillance Report 2007. Stockholm, Sweden: European Centre for Disease Prevention and Control, 2008.	2007	
EUVAC-NET. EUVAC-NET Measles Surveillance Report 2005. Stockholm, Sweden: European Centre for Disease Prevention and Control, 2006.	2005	
EUVAC-NET. EUVAC-NET Measles Surveillance Report 2006. Stockholm, Sweden: European Centre for Disease Prevention and Control, 2007.	2006	
EUVAC-NET. EUVAC-NET Measles Surveillance Report 2008. Stockholm, Sweden: European Centre for Disease Prevention and Control, 2009.	2008	

World Health Organization (WHO). Expanded programme on immunization. Measles outbreak. Wkly Epidemiol Rec. 1990; 65(49): 379-81.	1988	*
WHO Regional Office for Europe (EURO-WHO). Social Determinants of Health and Well-being Among Young People: Health Behaviour in School-aged Children (HBSC) Study: International Report from the 2009/2010 Survey. Copenhagen, Denmark: WHO Regional Office for Europe (EURO-WHO), 2012.	2009	*
Mathers BM, Degenhardt L, Phillips B, Wiessing L,Hickman M, Strathdee SA, Wodak A, Panda S, Tyndall M, Toufik A, Mattick RP. Global epidemiology of injecting drug use and HIV among people who inject drugs: a systematic review. Lancet. 2008; 372: 1733-1745.	2007	*
Henthorn J, Anionwu E, Brozovic M. Screening cord blood for sickle haemoglobinopathies in Brent. Br Med J (Clin Res Ed). 1984; 289(6443): 47[illegible] 80.	1981-1983	*
McCarthy HD, Jarrett KV, Emmett PM, Rogers I. Trends in waist circumferences in young British children: a comparative study. Int J Obes (Lond 2005; 29(2): 157-62.	1995	
Jebb SA, Rennie KL, Cole TJ. Prevalence of overweight and obesity among young people in Great Britain. Public Health Nutr. 2004; 7(3): 461-5.	1997	
Wardle J, Williamson S, Johnson F, Edwards C. Depression in adolescent obesity: cultural moderators of the association between obesity and depressi symptoms. Int J Obes (Lond). 2006; 30(4): 634-43.	1999	
Fisher L, Fraser J, Alexander C. Caregivers' inability to identify childhood adiposity: a cross-sectional survey of rural children and their caregive attitudes. Aust J Rural Health. 2006; 14(2): 56-61.	2002	
Steele RM, van Sluijs EMF, Cassidy A, Griffin SJ, Ekelund U. Targeting sedentary time or moderate- and vigorous-intensity activity: independent relations with adiposity in a population-based sample of 10-y-old British children. Am J Clin Nutr. 2009; 90(5): 1185-92.	2007	
Sandercock GRH, Ogunleye A, Voss C. Comparison of cardiorespiratory fitness and body mass index between rural and urban youth: findings from t East of England Healthy Hearts Study. Pediatr Int. 2011; 53(5): 718-24.	2007	
Ottova V, Erhart M, Rajmil L, Dettenborn-Betz L, Ravens-Sieberer U. Overweight and its impact on the health-related quality of life in children an adolescents: results from the European KIDSCREEN survey. Qual Life Res. 2012; 21(1): 59-69.	2003	
Buchan IE, Bundred PE, Kitchiner DJ, Cole TJ. Body mass index has risen more steeply in tall than in short 3-year olds: serial cross-sectional surve 1988-2003. Int J Obes (Lond). 2007; 31(1): 23-9.	1988-2003	
Löfdahl HE, Lane A, Lu Y, Lagergren P, Harvey RF, Blazeby JM, Lagergren J. Increased population prevalence of reflux and obesity in the United Kingdom compared with Sweden: a potential explanation for the difference in incidence of esophageal adenocarcinoma. Eur J Gastroenterol Hepatol. 2011; 23(2): 128-32.	1996	
Mitchell RT, McDougall CM, Crum JE. Decreasing prevalence of obesity in primary schoolchildren. Arch Dis Child. 2007; 92(2): 153-4.	2004	
Shiue I. Associated social factors of body mass index in adults and the very old in the UK. Int J Cardiol. 2013; 168(1): 543-	2009	
ISSP Research Group (2013): International Social Survey Programme: Health and Health Care - ISSP 2011. GESIS Data Archive, Cologne. ZA580[illegible] Data file version 2.0.0, doi:10.4232/1/11759.	2011	*
Ahmed ML, Allen AD, Dunger DB, Macfarlane A. The Oxford growth study: a district growth surveillance programme 1988-1994. J Med Screen. 1995; 2(3): 160-3. as it appears in World Health Organization (WHO). WHO Global Database on Child Growth and Malnutrition - Historical.	1988-1994	
Trichopoulos D, Bamia C, Lagiou P, Fedirko V, Trepo E, Jenab M, Pischon T, Nöthlings U, Overved K, Tjønneland A, Outzen M, Clavel-Chapelon F Kaaks R, Lukanova A, Boeing H, Aleksandrova K, Benetou V, Zylis D, Palli D, Pala V, Panico S, Tumino R, Sacerdote C, Bueno-De-Mesquita HB, Van Kranen HJ, Peeters PHM, Lund E, Quirós JR, González CA, Sanchez Perez M-J, Navarro C, Dorronsoro M, Barricarte A, Lindkvist B, Regnér S, Werner M, Hallmans G, Khaw K-T, Wareham N, Key T, Romieu I, Chuang S-C, Murphy N, Boffetta P, Trichopoulou A, Riboli E. Hepatocellular carcinoma risk factors and disease burden in a European cohort: a nested case-control study. J Natl Cancer Inst. 2011; 103(22): 1686-95.	1992-2006	
Fowkes FG, Leng GC, Donnan PT, Deary IJ, Riemersma RA, Housley E. Serum cholesterol, triglycerides, and aggression in the general populatic Lancet. 1992; 340(8826): 995-8.	1987	
Forrest RD, Jackson CA, Yudkin JS. Glucose intolerance and hypertension in north London: the Islington Diabetes Survey. Diabet Med. 1986; 3(4).	1984-1985	
Cattaneo A. Breastfeeding in Europe: a blueprint for action. J Public Health. 2005; 13(2): 89-96	2000	
Kelly YJ, Watt RG. Breast-feeding initiation and exclusive duration at 6 months by social class--results from the Millennium Cohort Study. Pub Health Nutr. 2005; 8(4): 417-21.	2001	
Department of Health (United Kingdom), Health Protection Agency (United Kingdom), Joint United Nations Program on HIV/AIDS (UNAIDS). United Kingdom UNGASS Country Progress Report 2010. Geneva, Switzerland: Joint United Nations Program on HIV/AIDS (UNAIDS), 2010.	2009	
Gay NJ, Morgan-Capner P, Wright J, Farrington CP, Miller E. Age-specific antibody prevalence to hepatitis A in England: implications for disea control. Epidemiol Infect. 1994; 113(1): 113-20.	1986-1987	
Masterton RG, Strike PW, Tettmar RE. Hepatitis A immunity and travel history. J Infect. 1991; 23(3): 321-[illegible]	1987-1988	*
Scott NJ, Harrison JF, Zuckerman AJ. Hepatitis A antibody in blood donors in North East Thames region: implications to prevention policie Epidemiol Infect. 1989; 103(2): 377-82.	1988	
Bernal W, Smith H, Williams R. A Community Prevalence of Antibodies to Hepatitis A and E in Inner-City London. J Med Virol. 1996; 230-4.	1988-1989	
Cumberland NS, Masterton RG, Green AD, Sims MM. Prevalence of immunity to hepatitis A in recruits to the British Army and Royal Air Force. J Army Med Corps. 1994; 140(2): 71-5.	1989-1992	
Zuckerman JN, Powell L. Hepatitis A antibodies in attenders of London travel clinics: Cost-benefit of screening prior to hepatitis A immunisation Med Virol. 1994; 44(4): 393-4.	1991-1993	
Morris MC, Gay NJ, Hesketh LM, Morgan-Capner P, Miller E. The changing epidemiological pattern of hepatitis A in England and Wales. Epidemi Infect. 2002; 128(3): 457-63.	1996	
Ross JDC, Ghanem M, Tariq A, Gilleran G, Winter AJ. Seroprevalence of hepatitis A immunity in male genitourinary medicine clinic attenders: a ca control study of heterosexual and homosexual men. Sex Transm Infect. 2002; 78(3): 174-9.	2000	
Morris-Cunnington MC, Edmunds WJ, Miller E, Brown DWG. A population-based seroprevalence study of hepatitis A virus using oral fluid in Englar and Wales. Am J Epidemiol. 2004; 159(8): 786-94.	2001-2003	
Jarvis D, Luczynska C, Chinn S, Burney P. The association of hepatitis A and Helicobacter pylori with sensitization to common allergens, asthma a hay fever in a population of young British adults. Allergy. 2004; 59(10): 1063-7.	2003	
Dalton HR, Stableforth W, Hazeldine S, Thurairajah P, Ramnarace R, Warshow U, Ijaz S, Ellis V, Bendall R. Autochthonous hepatitis E in Southwe England: a comparison with hepatitis A. Eur J Clin Microbiol Infect Dis. 2008; 27(7): 579-85.	2005-2006	
Bates CJ, Mansoor MA, Pentieva KD, Hamer M, Mishra GD. Biochemical risk indices, including plasma homocysteine, that prospectively predict mortality in older British people: the National Diet and Nutrition Survey of People Aged 65 Years and Over. Br J Nutr. 2010; 104(6): 893-9.	1994-1995	*
Tunstall-Pedoe H, Kuulasmaa K, Mähönen M, Tolonen H, Ruokokoski E. Contribution of trends in survival and coronary-event rates to changes in coronary heart disease mortality: 10-year results from 37 WHO MONICA Project populations. Lancet. 1999; 353(9164): 1547-57.	1980-1995	
Abusheikha N, Akagbosu F, Marcus S, Lass A, Cousins C, Brinsden P. Viral screening and assisted conception treatment--the Bourn Hall experience. Assist Reprod Genet. 1999; 16(6): 337-9.	1987-1998	
Anderson SR, Righarts A, Maguire H. Surveillance of antenatal infections--HIV, hepatitis B, syphilis and rubella susceptibility in London. Commun D Public Health. 2004; 7(4): 251-7.	2002	
Balogun MA, Ramsay ME, Hesketh LM, Andrews N, Osborne KP, Gay NJ, Morgan-Capner P. The prevalence of hepatitis C in England and Wales. Infect. 2002; 45(4): 219-26.	1986, 1991, 1996	
Bracebridge S, Irwin D, Millership S. Prevention of perinatal hepatitis B transmission in a health authority area: an audit. Commun Dis Public Healt 2004; 7(2): 138-41.	1996-2002	
Brant LJ, Hurrelle M, Balogun MA, Klapper P, Ahmad F, Boxall E, Hale A, Hollyoak V, Ibrahim IB, Irving W, Meigh R, Mutton KJ, Patel BC, Paver WK, Pugh S, Taylor C, Turner AJ, Ramsay ME. Sentinel laboratory surveillance of hepatitis C antibody testing in England: understanding the epidemiology of HCV infection. Epidemiol Infect. 2007; 135(3): 417-26.	2002-2003	
Chrystie I, Sumner D, Palmer S, Kenney A, Banatvala J. Screening of pregnant women for evidence of current hepatitis B infection: selective universal? Health Trends. 1992; 24(1): 13-5.	1988-1990	
Cunningham R, Northwood JL, Kelly CD, Boxall EH, Andrews NJ. Routine antenatal screening for hepatitis B using pooled sera: validation and revie of 10 years experience. J Clin Pathol. 1998; 51(5): 392-5.	1986-1996	
Gay NJ, Hesketh LM, Osborne KP, Farrington CP, Morgan-Capner P, Miller E. The prevalence of hepatitis B infection in adults in England and Wale Epidemiol Infect. 1999; 122(1): 133-8.	1996	
Gore SM, Brettle RP, Burns SM, Lewis SC. Pilot study to estimate survivors to 1995 of 1983-1984 prevalent hepatitis C infections in Lothian patients who tested positive or negative for hepatitis B surface antigen in 1983-1984. J Infect. 1998; 37(2): 159-65.	1983-1984	
Ijaz S, Vyse AJ, Morgan D, Pebody RG, Tedder RS, Brown D. Indigenous hepatitis E virus infection in England: more common than it seems. J Cl Virol. 2009; 44(4): 272-6.	1991,2004	

MacLean AB, Cameron S, Follett EAC. Prevalence of hepatisis B and C viruses and human immunodeficiency virus infections in women reproductive age. Br J Obstet Gynaecol. 1993; 100(7): 702-3.	1991, 1993	
Sturrock CJ, Currie MJ, Vally H, OÕkeefe EJ, Primrose R, Habel P, Schamburg K, Bowden FJ. Community-based sexual health care works: a revie of the ACT outreach program. Sex Health. 2007; 4(3): 201-4.	2002-2005	
Castillo Taucher S. [Services for the care and prevention of birth defects. Reduced report of a World Health Organization and March of Dim Foundation meeting]. Rev Med Chil. 2007; 135(6): 806-13.	1955-2000	
Curtis V. Hand Washing at the London School of Hygiene and Tropical Medicine (LSHTM): The Shocking Truth. 200	2004	*
Giraudon I, Forde J, Maguire H, Arnold J, Permalloo N. Antenatal screening and prevalence of infection: surveillance in London, 2000-2007. Eu Surveill. 2009; 14(9): 8-12.	2000-2007	
Gunby JA, Darby SC, Miles JC, Green BM, Cox DR. Factors affecting indoor radon concentrations in the United Kingdom. Health Phys. 1993; 64(1): 12.	2005	
Curtis V, Biran A, Deverell K, Hughes C, Bellamy K, Drasar B. Hygiene in the home: relating bugs and behaviour. Soc Sci Med. 2003; 57(4): 657-72	2003	*
Judah G, Aunger R, Schmidt WP, Michie S, Granger S, Curtis V. Experimental pretesting of hand-washing interventions in a natural setting. Am Public Health. 2009; 99(Suppl 2): S405-11.	2009	*
Hull MG, Glazener CM, Kelly NJ, Conway DI, Foster PA, Hinton RA, Coulson C, Lambert PA, Watt EM, Desai KM. Population study of caus treatment, and outcome of infertility. Br Med J (Clin Res Ed). 1985; 291(6510): 1693-7.	1982-1983	*
Flohr C, Weinmayr G, Weiland SK, Addo-Yobo E, Annesi-Maesano I, Björkstén B, Bråbäck L, Büchele G, Chico M, Cooper P, Clausen M, El Sharif N, Martinez Gimeno A, Mathur RS, von Mutius E, Morales Suarez-Varela M, Pearce N, Svabe V, Wong GWK, Yu M, Zhong NS, Williams HC, ISAAC Phase Two Study Group. How well do questionnaires perform compared with physical examination in detecting flexural eczema? Findings from the International Study of Asthma and Allergies in Childhood (ISAAC) Phase Two. Br J Dermatol. 2009; 161(4): 846-53.	2005-2007	*
Stamler J, Elliott P, Chan Q. INTERMAP Appendix Tables. J Hum Hypertens. 2003; 17: 665-775. as it appears in Global Dietary Database Consortium, Nutrition and Chronic Disease Expert Group (NutriCoDE). Global Dietary Database 1980-2011. [Unpublished].	1997-1998	
United Kingdom Dietary Sodium 24 Hour Urine Sample Survey 2008 as it appears in Global Dietary Database Consortium, Nutrition and Chron Disease Expert Group (NutriCoDE). Global Dietary Database 1980-2011. [Unpublished].	2008	
Muir MS, Brayne C. A longitudinal study in progress: a five-year follow-up of women aged 70-79 years living in a rural communit Neuroepidemiology. 1992; 11(Suppl 1): 67-70.	1982-1985	
Kettil Bruun Society for Social and Epidemiological Research on Alcohol, University of North Dakota, Aarhus University, Alcohol Research Group/Public Health Institute, Centre for Addiction and Mental Health, University of Melbourne, Swiss Institute for the Prevention of Alcohol and Drug Problems. Gender, Alcohol, and Culture: an International Study (GENACIS) Childhood Sexual Abuse and Intimate Partner Violence Data as provided by the Global Burden of Disease 2010 Childhood Sexual Abuse and Intimate Partner Violence Expert Group. [Unpublished].	2000, 2002, 2005	
Home Office (United Kingdom). Domestic Violence: Findings From a New British Crime Survey Self-completion Questionnaire. London, Unit Kingdom: Home Office (United Kingdom), 1999.	1996	
Kennedy CR, Kimm L, Cafarelli Dees D. Controlled trial of universal neonatal screening for early identification of permanent childhood heari impairment. Lancet. 1998; 352(9145): 1957-64.	1993-1996	*
Surman G, Newdick H, King A, Gallagher M, Kurinczuk JJ. 4Child: Four Counties Database of Cerebral Palsy, Vision Loss and Hearing Loss in Children. Annual Report 2009, including data for births 1984 to 2003. Oxford, United Kingdom: National Perinatal Epidemiology Unit, 2009.	1984-2003	
Watkin P, Baldwin M. The longitudinal follow up of a universal neonatal hearing screen: the implications for confirming deafness in childhood. In Audiol. 2012; 51(7): 519-28.	1992-2002	*
Wang Y, Rudd AG, Wolfe CD. Age and ethnic disparities in incidence of stroke over time: the South London Stroke Register. Stroke. 2013; 44(12 3298-304. [Unpublished data].	1995-2010	*
Markowe HL, Bulpitt CJ, Shipley MJ, Rose G, Crombie DL, Fleming DM. Prognosis in adult asthma: a national study. Br Med J (Clin Res Ed). 198 295(6604): 949-52.	1970-1976	*
Fine PE, Clarkson JA. Distribution of immunity to pertussis in the population of England and Wales. J Hyg (Lond). 1984; 92(1): 21-36.	1950-1981	
Peace Research Institute Oslo (PRIO). Battle Deaths Dataset Version 3, 2009. Oslo, Norway: Peace Research Institute Oslo (PRIO), 2009.	1971-1988	
United Kingdom Dementia Data 1988-1991 from literature review, as provided by the Global Burden of Disease 2010 Dementia Expert Group [Unpublished].	1988-1991	
Williams DR, Bingham SA. Sodium and potassium intakes in a representative population sample: estimation from 24 h urine collections known to be complete in a Cambridgeshire village. Br J Nutr. 1986; 55(1): 13-22. as it appears in Global Dietary Database Consortium, Nutrition and Chronic Disease Expert Group (NutriCoDE). Global Dietary Database 1980-2011. [Unpublished].	1985	
European Surveillance of Congenital Anomalies (EUROCAT), Netherlands Organisation for Applied Scientific Research (TNO), Surveillance o Cerebral Palsy in Europe (SCPE). European Perinatal Health Report 2008.	2004	
Confidential Enquiry into Maternal and Child Health (CEMACH). Perinatal Mortality 2005: England, Wales and Northern Ireland. London, Unit Kingdom: Confidential Enquiry into Maternal and Child Health (CEMACH), 2007.	2005	
Jones RA, Cummins M, Davies PA. Infants of very low birthweight. A 15-year analysis. Lancet. 1979; 1(8130): 1332–	1963	
Pharoah PO, Alberman ED. Mortality of low birthweight infants in England and Wales 1953 to 1979. Arch Dis Child. 1981; 56(2): 86–9.	1965, 1971, 1974, 1977-1978	
Brown ER, Taeusch HW. Intensive care and the very low birthweight infant. Lancet. 1979; 2(8138): 362–3	1973	
Tin W, Wariyar U, Hey E. Changing prognosis for babies of less than 28 weeks' gestation in the north of England between 1983 and 1994. Norther Neonatal Network. BMJ. 1997; 314(7074): 107–11.	1988, 1992	
Cartlidge PH, Stewart JH. Survival of very low birthweight and very preterm infants in a geographically defined population. Acta Paediatr. 1997; 86(1 105–10.	1993	
Wariyar U, Richmond S, Hey E. Pregnancy outcome at 24-31 weeks' gestation: neonatal survivors. Arch Dis Child. 1989; 64(5): 678–86.	1983	
Lumley J, Kitchen WH, Roy RN, Yu VY, Drew JH. The survival of extremely-low-birthweight infants in Victoria: 1982-1985. Med J Aust. 198 149(5): 242-6.	1984	
Partially Hydrogenated Vegetable Oil Industry Estimates of Total Retail and Food Service Volume as it appears in Global Dietary Database Consortium, Nutrition and Chronic Disease Expert Group (NutriCoDE). Global Dietary Database 1980-2011. [Unpublished].	2000, 2005, 2009	
United Kingdom General Household Survey 1980 as it appears in P.N. Lee Statistics and Computing. International Mortality and Smoking Statistics.	1980	
TRANSFAIR study as it appears in Global Dietary Database Consortium, Nutrition and Chronic Disease Expert Group (NutriCoDE). Global Dietar Database 1980-2011. [Unpublished].	1996	
United Kingdom ONS Omnibus Survey 1996 as it appears in P.N. Lee Statistics and Computing. International Mortality and Smoking Statistics.	1996	
England Teenage Smoking Attitudes Survey 1996 as it appears in P.N. Lee Statistics and Computing. International Mortality and Smoking Statistics.	1996	
United Kingdom ONS Omnibus Survey 1997 as it appears in P.N. Lee Statistics and Computing. International Mortality and Smoking Statistics.	1997	
England Teenage Smoking Attitudes Survey 1997 as it appears in P.N. Lee Statistics and Computing. International Mortality and Smoking Statistics.	1997	
England Teenage Smoking Attitudes Survey 1998 as it appears in P.N. Lee Statistics and Computing. International Mortality and Smoking Statistics.	1998	
Eurostat Database as it appears in World Health Organization (WHO). WHO Global Infobase - Tobacco Use Prevalence	1999	
United Kingdom ONS Omnibus Survey 1999 as it appears in P.N. Lee Statistics and Computing. International Mortality and Smoking Statistics.	1999	
United Kingdom ONS Omnibus Survey 2000 as it appears in P.N. Lee Statistics and Computing. International Mortality and Smoking Statistics.	2000	
United Kingdom ONS Omnibus Survey 2001 as it appears in P.N. Lee Statistics and Computing. International Mortality and Smoking Statistics.	2001	
United Kingdom ONS Omnibus Survey 2002 as it appears in P.N. Lee Statistics and Computing. International Mortality and Smoking Statistics.	2002	
United Kingdom ONS Omnibus Survey 2003 as it appears in P.N. Lee Statistics and Computing. International Mortality and Smoking Statistics.	2003	
United Kingdom ONS Omnibus Survey 2004 as it appears in P.N. Lee Statistics and Computing. International Mortality and Smoking Statistics.	2004	

United Kingdom ONS Omnibus Survey 2005 as it appears in P.N. Lee Statistics and Computing. International Mortality and Smoking Statistics.	2005	
Sales of Tobacco Goods to the Trade 1980-89 as it appears in P.N. Lee Statistics and Computing. International Mortality and Smoking Statistics.	1980-1989	
General Health Related Behaviour Questionnaire as it appears in P.N. Lee Statistics and Computing. International Mortality and Smoking Statistics.	1984-1987	
United Kingdom - England North Thames (West) Congenital Malformation Register Data 1997-1998 - WHO as it appears in the European Surveillance of Congenital Anomalies (EUROCAT), International Centre on Birth Defects, World Health Organization (WHO). World Atlas of Birth Defects. 2nd ed. Geneva, Switzerland: World Health Organization (WHO), 2003.	1997-1998	*
United Kingdom - England Mersey Congenital Anomaly Survey Data 1998 - WHO as it appears in the European Surveillance of Congenital Anomalies (EUROCAT), International Centre on Birth Defects, World Health Organization (WHO). World Atlas of Birth Defects. 2nd ed. Geneva, Switzerland: World Health Organization (WHO), 2003.	1998	*
United Kingdom - England and Wales Congenital Malformation Monitoring Program Data 1980 - ICBDMS as it appears in International Clearinghous for Birth Defects Monitoring Systems. Congenital Malformations Worldwide: A Report from the International Clearinghouse for Birth Defects Monitoring Systems. Amsterdam, Netherlands: Elsevier, 1991.	1980	
United Kingdom - England and Wales Congenital Malformation Monitoring Program Data 1982 - ICBDMS as it appears in International Clearinghous for Birth Defects Monitoring Systems. Congenital Malformations Worldwide: A Report from the International Clearinghouse for Birth Defects Monitoring Systems. Amsterdam, Netherlands: Elsevier, 1991.	1982	*
United Kingdom - England and Wales Congenital Malformation Monitoring Program Data 1983 - ICBDMS as it appears in International Clearinghous for Birth Defects Monitoring Systems. Congenital Malformations Worldwide: A Report from the International Clearinghouse for Birth Defects Monitoring Systems. Amsterdam, Netherlands: Elsevier, 1991.	1983	*
United Kingdom - England and Wales Congenital Malformation Monitoring Program Data 1984 - ICBDMS as it appears in International Clearinghous for Birth Defects Monitoring Systems. Congenital Malformations Worldwide: A Report from the International Clearinghouse for Birth Defects Monitoring Systems. Amsterdam, Netherlands: Elsevier, 1991.	1984	*
United Kingdom - England and Wales Congenital Malformation Monitoring Program Data 1985 - ICBDMS as it appears in International Clearinghous for Birth Defects Monitoring Systems. Congenital Malformations Worldwide: A Report from the International Clearinghouse for Birth Defects Monitoring Systems. Amsterdam, Netherlands: Elsevier, 1991.	1985	*
United Kingdom - England and Wales Congenital Malformation Monitoring Program Data 1986 - ICBDMS as it appears in International Clearinghous for Birth Defects Monitoring Systems. Congenital Malformations Worldwide: A Report from the International Clearinghouse for Birth Defects Monitoring Systems. Amsterdam, Netherlands: Elsevier, 1991.	1986	*
United Kingdom - England and Wales Congenital Malformation Monitoring Program Data 1987 - ICBDMS as it appears in International Clearinghous for Birth Defects Monitoring Systems. Congenital Malformations Worldwide: A Report from the International Clearinghouse for Birth Defects Monitoring Systems. Amsterdam, Netherlands: Elsevier, 1991.	1987	*
United Kingdom - England and Wales Congenital Malformation Monitoring Program Data 1988 - ICBDMS as it appears in International Clearinghous for Birth Defects Monitoring Systems. Congenital Malformations Worldwide: A Report from the International Clearinghouse for Birth Defects Monitoring Systems. Amsterdam, Netherlands: Elsevier, 1991.	1988	*
United Nations Population Division. United Nations World Population Prospects 1950-2100 - 2010 Revision. New York City, United States: Unite Nations Population Division, 2011.	2005	
Measles Supplementary Immunization Activity Data as provided by the World Health Organization. [Unpublishec	1994	
Office of Population Censuses and Surveys. Notification of Infectious Diseases in England and Wal	1970-2008	
United Nations Children's Fund (UNICEF), World Health Organization (WHO). WHO and UNICEF Reported Disease Incidence Time Series Measles. Geneva, Switzerland: World Health Organization (WHO).	1980-1998, 2000-2013	
Bojesen A, Juul S, Birkebaek N, Gravholt CH. Increased mortality in Klinefelter syndrome. J Clin Endocrinol Metab. 2004; 89(8): 3830-4.	2004^	
Hunt GM, Oakeshott P. Outcome in people with open spina bifida at age 35: prospective community based cohort study. BMJ. 2003; 326(7403): 136 6.	2003^	
Bowman RM, McLone DG, Grant JA, Tomita T, Ito JA. Spina bifida outcome: a 25-year prospective. Pediatr Neurosurg. 2001; 34(3): 114-20.	2001^	
Christensen K, Juel K, Herskind AM, Murray JC. Long term follow up study of survival associated with cleft lip and palate at birth. BMJ. 2004; 32 1405. and	2004^	
Smile Train. China and India Smile Train Congenital Anomalies Data 2004-2009	2004-2009^	
Frid C, Drott P, Otterblad Olausson P, Sundelin C, Annerén G. Maternal and neonatal factors and mortality in children with Down syndrome born 1973-1980 and 1995-1998. Acta Paediatr. 2004; 93(1): 106-12.	2004^	
Baird PA, Sadovnick AD. Life expectancy in Down syndrome. J Pediatr. 1987; 110(6): 849-54	1987^	
Tennant PWG, Pearce MS, Bythell M, Rankin J. 20-year survival of children born with congenital anomalies: a population-based study. Lancet. 201 375(9715): 649-56.	2010^	
Congenital Heart Anomalies Mortality Risk With No Diagnosis or Care Estimates as provided by the Global Burden of Disease 2010 congenit anomaly expert group. [Unpublished].	2010^	
Siciliani L, Hurst J. Explaining Waiting-Time Variations for Elective Surgery Across OECD Countries. OECD Economic Studies. 2004; 38(1): 95-12	2004^	
Price WH, Clayton JF, Collyer S, De Mey R, Wilson J. Mortality ratios, life expectancy, and causes of death in patients with Turner's syndrome Epidemiol Community Health. 1986; 40(2): 97-102.	1986^	
Sinaii N, Plumb K, Cotton L, Lambert A, Kennedy S, Zondervan K, Stratton P. Differences in characteristics among 1,000 women with endometrio based on extent of disease. Fertil Steril. 2007; 89(3): 538-45.	2007^	
Bushmakin AG, Cappelleri JC, Taylor-Stokes G, Sayers J, Sadosky A, Carroll D, Gosden T, Emery P. Relationship between patient-reported disease severity and other clinical outcomes in osteoarthritis: a European perspective. J Med Econ. 2011; 14(4): 381-9.	2011^	*
Fortnum H, Davis A. Hearing impairment in children after bacterial meningitis: incidence and resource implications. Br J Audiol. 1993; 27(1): 43-52.	1993^	
Richardson M, Williamson T, Reid A, Tarlow M, Rudd P. Testing for hearing loss after meningitis. J Pediatr. 1998; 132(4): 749-50.	1998^	
Bedford H, de Louvois J, Halket S, Peckham C, Hurley R, Harvey D. Meningitis in infancy in England and Wales: follow up at age 5 years. BMJ. 20 323(7312): 533-6.	2001^	
Archer LN, Levene MI, Evans DH. Cerebral artery Doppler ultrasonography for prediction of outcome after perinatal asphyxia. Lancet. 1986; 2(8516 1116-8.	1986^	
Gluckman PD, Wyatt JS, Azzopardi D, Ballard R, Edwards AD, Ferriero DM, Polin RA, Robertson CM, Thoresen M, Whitelaw A, Gunn AJ. Selective head cooling with mild systemic hypothermia after neonatal encephalopathy: multicentre randomised trial. Lancet. 2005; 365(9460): 663-70.	2005^	
Brayne C, Calloway P. An epidemiological study of dementia in a rural population of elderly women. Br J Psychiatry. 1989; 155: 214-9.	1989^	
Clarke M, Jagger C, Anderson J, Battcock T, Kelly F, Stern MC. The prevalence of dementia in a total population: a comparison of two screeni instruments. Age Ageing. 1991; 20(6): 396-403.	1991^	
Schrag A, Jahanshahi M, Quinn N. What contributes to quality of life in patients with Parkinson's disease? J Neurol Neurosurg Psychiatry. 2000; 69(3 308-12.	2000^	
Cruz N, Vieta E, Comes M, Haro JM, Reed C, Bertsch J, EMBLEM Advisory Board. Rapid-cycling bipolar I disorder: course and treatment outcome a large sample across Europe. J Psychiatr Res. 2008; 42(13): 1068-75.	2008^	
Mutch LM, Brown NJ, Speidel BD, Dunn PM. Perinatal mortality and neonatal survival in Avon: 1976-9. Br Med J (Clin Res Ed). 1981; 282(6258) 119–22.	1981^	
Adeyoju AB, Olujohungbe AB, Morris J, Yardumian A, Bareford D, Akenova A, Akinyanju O, Cinkotai K, O'Reilly PH. Priapism in sickle-cell disea incidence, risk factors and complications – an international multicentre study. BJU Int. 2002; 90(9): 898–902.	2002^	
Global Parkinson's Disease Survey Steering Committee. Factors impacting on quality of life in Parkinson's disease: results from an international surve Mov Disord. 2002; 17(1): 60-7.	2002^	
Tsakos G, Herrick K, Sheiham A, Watt RG. Edentulism and fruit and vegetable intake in low-income adults. J Dent Res. 2010; 89(5): 462-7.	2010^	
Nuttall NM, Steele JG, Evans D, Chadwick B, Morris AJ, Hill K. The reported impact of oral condition on children in the United Kingdom, 2003. I Dent J. 2006; 200(10): 551-5.	2006^	
Sheiham A, Steele J. Does the condition of the mouth and teeth affect the ability to eat certain foods, nutrient and dietary intake and nutritional sta amongst older people? Public Health Nutr. 2001; 4(3): 797-803.	2001^	
Mason C, Porter SR, Madland G, Parry J. Early management of dental pain in children and adolescents. J Dent. 1997; 25(1): 31-4.	1997^	
Bradbury J, Thomason JM, Jepson NJA, Walls AWG, Mulvaney CE, Allen PF, Moynihan PJ. Perceived chewing ability and intake of fruit a vegetables. J Dent Res. 2008; 87(8): 720-5.	2008^	

Shepherd MA, Nadanovsky P, Sheiham A. The prevalence and impact of dental pain in 8-year-old school children in Harrow, England. Br Dent 1999; 187(1): 38-41.	1999^		
Galloway H, Clark NS, Blackhall M. Paediatric aspects of the Aberdeen typhoid outbreak. Arch Dis Child. 1966; 41(215): 63-8	1966^		
Ispahani P, Slack RC. Enteric fever and other extraintestinal salmonellosis in University Hospital, Nottingham, UK, between 1980 and 1997. Eur J Cli Microbiol Infect Dis. 2000; 19(9): 679-87.	2000^		
Sloan N, Durocher J, Aldrich T, Blum J, Winikoff B. What measured blood loss tells us about postpartum bleeding: a systematic review. BJOG. 201 117(7): 788-800.	2010^		
Institute for Health Metrics and Evaluation (IHME), Joint United Nations Program on HIV/AIDS (UNAIDS). Institute for Health Metrics ar Evaluation HIV Mortality Estimates Based on the UNAIDS Spectrum Model Framework.			

*For databases, multinational reports, hospital data and scientific literature, the Year column represents the year that the data were used for (the estimate year). For all other data types (surveys, vital registration, etc.), Year indicates the source year or year of data collection.
^For these sources, Year indicates the source year or year of data collection. For all others, Year represents the year that the data were used for (the estimate year).

Web Table 3 **: Age-standardized death, years of life lost (YLL), years lived with disability (YLD), and disability-adjusted life years (DALY) rates with life expectancy at birth for 1990 and 2013 for both sexes combined**																				
	**Age-standardized death rate (per 100,000)**				**Age-standardized YLL rate (per 100,000)**				**Age-standardized YLD rate (per 100,000)**				**Age-standardized DALY rate (per 100,000)**				**Life expectancy at birth (years)**			
	**1990**		**2013**		**1990**		**2013**		**1990**		**2013**		**1990**		**2013**		**1990**		**2013**	
	**Rate**	**Rank**	**Rate**	**Rank**	**Rate**	**Rank**	**Rate**	**Rank**	**Rate**	**Rank**	**Rate**	**Rank**	**Rate**	**Rank**	**Rate**	**Rank**	**Life Expectancy**	**Rank**	**Life Expectancy**	**Rank**
United Kingdom	771 (768-775)	19 (18-20)	516 (497-535)	16 (13-20)	15,028 (14,962-15,101)	17 (16-17)	8,975 (8,578-9,373)	15 (12-19)	11,445 (8,547-14,709)	13 (10-19)	11,281 (8,392-14,542)	16 (11-19)	26,474 (23,567-29,754)	16 (15-18)	20,256 (17,367-23,463)	15 (11-19)	75.7 (75.7-75.8)	17 (16-18)	81.0 (80.6-81.4)	15 (12-18)
England	759 (756-763)	15 (15-17)	504 (486-522)	12 (9-15)	14,743 (14,669-14,817)	13 (13-15)	8,687 (8,298-9,077)	11 (8-15)	11,346 (8,474-14,613)	8 (5-15)	11,185 (8,321-14,423)	11 (7-15)	26,089 (23,211-29,349)	13 (12-15)	19,873 (17,014-23,060)	10 (6-14)	75.9 (75.9-76.0)	14 (13-15)	81.3 (80.9-81.7)	12 (8-14)
East Midlands	764 (756-773)	17 (15-19)	507 (484-531)	13 (9-19)	14,804 (14,607-15,004)	14 (13-16)	8,762 (8,310-9,266)	12 (8-18)	11,357 (8,415-14,631)	9 (3-23)	11,155 (8,325-14,380)	9 (2-21)	26,161 (23,221-29,518)	14 (12-17)	19,918 (17,022-23,120)	11 (5-17)	75.9 (75.7-76.0)	15 (13-17)	81.2 (80.7-81.7)	13 (8-17)
East of England	701 (693-709)	10 (8-11)	469 (446-488)	3 (1-8)	13,085 (12,899-13,260)	2 (2-5)	7,860 (7,441-8,268)	3 (1-6)	11,263 (8,384-14,416)	5 (1-19)	11,199 (8,349-14,416)	12 (3-23)	24,349 (21,409-27,549)	2 (1-6)	19,059 (16,209-22,245)	2 (1-6)	77.1 (77.0-77.2)	6 (3-10)	82.2 (81.8-82.8)	2 (1-6)
Greater London	777 (763-789)	20 (17-23)	501 (475-527)	11 (7-18)	15,435 (15,138-15,735)	20 (18-23)	8,563 (8,049-9,076)	10 (5-16)	11,400 (8,550-14,672)	12 (3-23)	11,275 (8,335-14,571)	15 (4-24)	26,835 (23,931-30,176)	19 (16-22)	19,839 (16,977-23,133)	8 (4-17)	75.5 (75.3-75.7)	19 (17-24)	81.4 (80.8-82.0)	10 (5-16)
North East England	861 (851-872)	31 (30-31)	557 (533-586)	27 (22-30)	16,863 (16,609-17,124)	30 (30-30)	9,744 (9,186-10,325)	25 (21-29)	11,512 (8,597-14,728)	18 (4-28)	11,306 (8,415-14,605)	17 (3-26)	28,376 (25,460-31,665)	30 (27-30)	21,051 (18,056-24,360)	25 (17-29)	74.4 (74.2-74.5)	30 (30-31)	80.1 (79.5-80.6)	25 (20-29)
North West England	827 (819-836)	29 (28-29)	557 (534-581)	26 (22-29)	16,297 (16,126-16,477)	28 (25-29)	9,954 (9,411-10,480)	28 (23-30)	11,298 (8,457-14,488)	7 (2-17)	11,070 (8,283-14,202)	4 (2-16)	27,596 (24,793-30,795)	25 (22-27)	21,024 (18,075-24,214)	24 (18-29)	74.8 (74.7-74.9)	29 (27-29)	80.0 (79.5-80.5)	26 (22-30)
South East England	691 (685-698)	7 (7-9)	463 (444-483)	2 (1-7)	13,103 (12,953-13,248)	3 (2-5)	7,639 (7,263-8,039)	1 (1-4)	11,018 (8,214-14,234)	1 (1-8)	10,960 (8,133-14,190)	3 (1-15)	24,121 (21,350-27,376)	1 (1-3)	18,599 (15,722-21,725)	1 (1-2)	77.2 (77.1-77.3)	3 (3-6)	82.4 (81.9-82.8)	1 (1-4)
South West England	693 (686-700)	8 (6-9)	477 (456-496)	7 (3-10)	13,173 (12,994-13,339)	4 (2-6)	8,136 (7,723-8,545)	5 (3-9)	11,455 (8,469-14,803)	15 (2-26)	11,083 (8,194-14,355)	7 (2-21)	24,629 (21,688-27,954)	6 (2-8)	19,220 (16,287-22,399)	3 (2-7)	77.1 (77.0-77.3)	5 (3-8)	82.0 (81.5-82.4)	4 (2-9)
West Midlands	784 (776-792)	22 (20-23)	520 (495-541)	17 (12-21)	15,409 (15,218-15,605)	19 (18-22)	9,258 (8,744-9,753)	19 (15-23)	11,496 (8,610-14,798)	17 (7-25)	11,417 (8,523-14,723)	21 (12-26)	26,906 (23,987-30,141)	20 (18-21)	20,675 (17,659-23,950)	20 (15-25)	75.5 (75.3-75.6)	20 (19-23)	80.9 (80.4-81.4)	16 (13-21)
Yorkshire and the Humber	794 (786-804)	23 (22-27)	534 (510-557)	20 (16-24)	15,567 (15,371-15,774)	22 (19-23)	9,404 (8,913-9,910)	22 (17-25)	11,544 (8,635-14,831)	20 (5-27)	11,343 (8,431-14,598)	19 (4-25)	27,111 (24,167-30,442)	21 (18-23)	20,747 (17,815-24,032)	21 (15-26)	75.3 (75.2-75.5)	23 (20-25)	80.6 (80.1-81.1)	21 (16-24)
Northern Ireland	800 (787-812)	27 (23-27)	578 (550-607)	29 (26-31)	15,716 (15,421-16,013)	23 (20-24)	10,237 (9,625-10,865)	30 (25-30)	11,840 (8,813-15,148)	26 (16-32)	11,596 (8,598-14,920)	24 (14-31)	27,556 (24,508-30,863)	23 (21-28)	21,833 (18,682-25,202)	30 (27-30)	75.2 (75.0-75.4)	24 (22-27)	79.6 (79.1-80.2)	30 (25-31)
Scotland	881 (872-889)	32 (32-32)	600 (576-626)	32 (29-32)	17,611 (17,385-17,844)	31 (31-32)	10,948 (10,435-11,552)	31 (31-31)	12,134 (9,008-15,581)	30 (24-32)	12,064 (8,890-15,432)	30 (25-32)	29,746 (26,561-33,187)	32 (31-32)	23,013 (19,840-26,521)	31 (31-32)	73.9 (73.8-74.1)	32 (32-32)	79.1 (78.5-79.5)	31 (30-32)
Wales	771 (760-781)	18 (16-21)	545 (520-569)	24 (18-27)	14,881 (14,646-15,113)	15 (13-17)	9,739 (9,132-10,262)	24 (20-29)	11,640 (8,692-15,027)	23 (9-30)	11,397 (8,517-14,704)	20 (4-29)	26,522 (23,664-29,870)	17 (14-20)	21,136 (18,106-24,517)	26 (17-29)	75.8 (75.6-76.0)	16 (14-18)	80.3 (79.8-80.9)	23 (18-27)
Australia	679 (675-684)	5 (4-6)	472 (455-491)	5 (1-9)	13,615 (13,506-13,736)	9 (7-10)	8,288 (7,923-8,687)	7 (4-11)	12,077 (9,032-15,487)	29 (23-32)	12,071 (8,996-15,547)	31 (26-32)	25,692 (22,630-29,132)	12 (11-14)	20,359 (17,328-23,820)	16 (9-23)	77.0 (76.9-77.1)	8 (5-11)	81.8 (81.4-82.2)	5 (2-11)
Austria	761 (753-768)	16 (15-18)	524 (500-548)	18 (11-24)	15,271 (15,110-15,432)	18 (18-20)	9,133 (8,652-9,598)	18 (11-23)	11,082 (8,295-14,330)	3 (1-18)	10,851 (8,049-13,987)	2 (1-14)	26,354 (23,571-29,635)	15 (13-19)	19,985 (17,095-23,156)	13 (4-19)	75.7 (75.6-75.8)	18 (16-19)	80.8 (80.3-81.3)	17 (12-23)
Belgium	744 (737-751)	13 (13-14)	538 (508-570)	22 (13-28)	14,897 (14,764-15,025)	16 (14-17)	9,717 (9,095-10,401)	23 (18-30)	11,842 (8,835-15,261)	27 (19-29)	11,535 (8,595-14,812)	23 (13-27)	26,739 (23,654-30,130)	18 (15-21)	21,253 (18,099-24,689)	28 (18-30)	76.0 (75.9-76.1)	13 (13-15)	80.2 (79.5-80.8)	24 (17-30)
Canada	660 (656-663)	2 (1-2)	488 (469-506)	9 (3-13)	13,267 (13,176-13,350)	5 (3-6)	8,817 (8,398-9,225)	14 (8-18)	11,193 (8,295-14,398)	4 (1-18)	11,129 (8,237-14,278)	8 (2-21)	24,461 (21,581-27,678)	3 (1-6)	19,946 (17,047-23,221)	12 (6-17)	77.4 (77.3-77.5)	2 (2-2)	81.5 (81.1-81.9)	9 (5-15)
Denmark	798 (791-805)	26 (23-27)	571 (549-601)	28 (23-32)	16,147 (15,984-16,329)	26 (24-28)	9,852 (9,384-10,408)	26 (20-30)	11,458 (8,635-14,822)	14 (3-26)	11,154 (8,349-14,421)	10 (3-22)	27,605 (24,765-30,993)	24 (21-28)	21,006 (18,089-24,313)	23 (17-29)	75.1 (74.9-75.2)	26 (24-27)	79.9 (79.4-80.4)	27 (21-30)
Finland	797 (788-806)	25 (23-27)	514 (492-537)	15 (9-22)	16,047 (15,866-16,253)	24 (24-27)	9,401 (8,884-9,900)	21 (14-26)	12,135 (9,103-15,585)	31 (26-32)	11,788 (8,813-15,195)	27 (20-31)	28,183 (25,103-31,693)	29 (26-30)	21,190 (18,130-24,590)	27 (18-30)	75.0 (74.9-75.2)	27 (24-27)	80.6 (80.1-81.1)	20 (14-25)
France	658 (654-663)	1 (1-2)	471 (456-488)	4 (1-9)	13,852 (13,784-13,918)	12 (10-12)	8,759 (8,395-9,126)	13 (8-18)	11,268 (8,453-14,462)	6 (1-18)	11,084 (8,314-14,204)	5 (1-18)	25,121 (22,289-28,340)	8 (6-11)	19,843 (16,954-23,049)	9 (5-16)	77.1 (77.1-77.2)	4 (3-6)	81.7 (81.3-82.1)	8 (3-12)
Germany	779 (774-783)	21 (19-22)	528 (519-536)	19 (15-23)	15,527 (15,457-15,598)	21 (19-23)	9,069 (8,887-9,256)	17 (13-21)	12,265 (9,158-15,820)	32 (27-32)	11,910 (8,832-15,364)	29 (25-32)	27,793 (24,675-31,401)	27 (22-28)	20,980 (17,879-24,449)	22 (18-28)	75.4 (75.4-75.5)	21 (19-23)	80.7 (80.5-80.9)	18 (15-22)
Greece	722 (716-727)	12 (12-12)	583 (564-604)	30 (27-32)	13,542 (13,425-13,656)	8 (7-9)	9,928 (9,507-10,359)	27 (22-30)	11,067 (8,273-14,316)	2 (1-15)	10,735 (8,006-13,866)	1 (1-9)	24,609 (21,790-27,848)	4 (2-7)	20,663 (17,796-23,777)	19 (13-27)	77.0 (76.9-77.1)	9 (6-11)	79.8 (79.4-80.2)	29 (23-30)
Ireland	847 (836-858)	30 (30-31)	547 (503-595)	23 (12-31)	16,202 (15,952-16,426)	27 (24-29)	9,398 (8,552-10,391)	20 (10-30)	11,357 (8,475-14,593)	10 (2-22)	11,071 (8,260-14,265)	6 (1-21)	27,559 (24,730-30,814)	22 (21-27)	20,469 (17,403-23,756)	17 (6-29)	74.8 (74.7-75.0)	28 (27-29)	80.4 (79.5-81.3)	22 (11-30)
Italy	683 (679-688)	6 (5-7)	457 (433-484)	1 (1-8)	13,523 (13,447-13,605)	7 (7-9)	7,767 (7,261-8,278)	2 (1-7)	11,628 (8,727-14,949)	22 (6-27)	11,504 (8,554-14,804)	22 (10-28)	25,151 (22,244-28,441)	9 (6-11)	19,271 (16,264-22,575)	4 (1-10)	77.0 (76.9-77.0)	10 (7-11)	82.1 (81.5-82.7)	3 (1-9)
Luxembourg	795 (774-815)	24 (20-27)	482 (445-521)	8 (1-18)	16,033 (15,538-16,505)	25 (22-29)	8,443 (7,671-9,189)	9 (2-18)	11,704 (8,807-15,147)	24 (8-30)	11,648 (8,725-15,008)	26 (12-31)	27,737 (24,640-31,274)	26 (21-30)	20,091 (16,982-23,518)	14 (4-23)	75.1 (74.8-75.4)	25 (21-28)	81.1 (80.3-81.9)	14 (4-23)

Netherlands	700 (695-705)	9 (8-11)	535 (514-558)	21 (15-26)	13,327 (13,220-13,442)	6 (4-6)	8,992 (8,559-9,449)	16 (10-21)	11,625 (8,616-14,925)	21 (9-26)	11,612 (8,694-14,953)	25 (17-28)	24,952 (21,955-28,215)	7 (5-10)	20,604 (17,558-24,065)	18 (13-25)	77.0 (76.9-77.1)	7 (5-11)	80.6 (80.1-81.0)	19 (14-25)
Norway	705 (697-714)	11 (9-11)	508 (492-522)	14 (9-18)	13,727 (13,541-13,924)	10 (8-12)	8,442 (8,157-8,726)	8 (5-13)	11,466 (8,517-14,630)	16 (4-24)	11,208 (8,314-14,384)	13 (4-22)	25,194 (22,288-28,358)	10 (6-12)	19,650 (16,748-22,762)	6 (3-13)	76.8 (76.7-76.9)	12 (11-12)	81.4 (81.1-81.7)	11 (7-15)
Portugal	818 (811-824)	28 (27-29)	548 (523-569)	25 (19-28)	17,750 (17,600-17,887)	32 (31-32)	10,006 (9,512-10,482)	29 (22-30)	11,535 (8,522-14,855)	19 (5-27)	11,315 (8,343-14,597)	18 (4-25)	29,285 (26,299-32,593)	31 (31-32)	21,322 (18,226-24,539)	29 (19-30)	74.3 (74.2-74.4)	31 (30-31)	79.8 (79.4-80.3)	28 (22-30)
Spain	674 (668-680)	4 (3-5)	474 (456-492)	6 (1-9)	13,821 (13,724-13,910)	11 (10-12)	8,202 (7,855-8,566)	6 (3-10)	11,380 (8,491-14,574)	11 (4-21)	11,207 (8,355-14,482)	14 (3-22)	25,201 (22,325-28,423)	11 (7-11)	19,410 (16,472-22,695)	5 (2-10)	77.0 (76.9-77.1)	11 (7-11)	81.7 (81.3-82.1)	7 (2-12)
Sweden	668 (662-673)	3 (3-4)	499 (477-521)	10 (6-18)	12,605 (12,477-12,731)	1 (1-1)	7,971 (7,550-8,379)	4 (1-8)	12,031 (9,036-15,408)	28 (22-32)	11,813 (8,777-15,134)	28 (19-32)	24,636 (21,602-27,984)	5 (2-9)	19,785 (16,714-23,079)	7 (3-16)	77.6 (77.5-77.7)	1 (1-1)	81.8 (81.3-82.2)	6 (2-12)
United States	745 (742-747)	14 (13-14)	594 (564-623)	31 (26-32)	16,364 (16,317-16,410)	29 (27-29)	11,930 (11,260-12,682)	32 (32-32)	11,812 (8,871-15,077)	25 (15-29)	12,074 (9,026-15,444)	32 (27-32)	28,177 (25,207-31,454)	28 (26-30)	24,004 (20,866-27,371)	32 (31-32)	75.4 (75.4-75.4)	22 (20-23)	78.9 (78.3-79.5)	32 (30-32)

Web Table 4 : Deaths in England in 1990 and 2013 for all ages and age-standardized rates (per 100,000) by sex with 95% UI and percentage change. Percent changes in bold denote a statistically significant change.

Both sexes combined

Cause	Deaths (in thousands) 1990	Deaths (in thousands) 2013	Deaths (in thousands) % change	Age-standardized rate (per 100,000) 1990	Age-standardized rate (per 100,000) 2013	Age-standardized rate (per 100,000) % change
**All causes**	535.4 (533.0 to 537.9)	479.3 (462.4 to 495.8)	**-10.5 (-13.65 to -7.27)**	759.6 (756.4 to 763.1)	504.3 (486.1 to 522.8)	**-33.6 (-36.07 to -31.09)**
**Communicable, maternal, neonatal, and nutritional diseases**	38.6 (36.5 to 46.5)	39.4 (35.9 to 47.7)	2.1 (-9.27 to 11.99)	56.1 (53.3 to 66.7)	40.3 (36.9 to 47.2)	**-28.0 (-35.88 to -22.02)**
**HIV/AIDS and tuberculosis**	0.9 (0.8 to 1.0)	0.6 (0.5 to 0.7)	**-35.7 (-47.72 to -23.53)**	1.5 (1.4 to 1.7)	0.8 (0.6 to 0.9)	**-49.1 (-58.68 to -39.01)**
Tuberculosis	0.6 (0.5 to 0.7)	0.4 (0.3 to 0.4)	**-34.5 (-49.51 to -24.71)**	0.9 (0.8 to 1.0)	0.4 (0.3 to 0.5)	**-50.8 (-61.86 to -43.80)**
HIV/AIDS	0.3 (0.3 to 0.4)	0.2 (0.1 to 0.3)	**-37.4 (-59.91 to -10.77)**	0.7 (0.5 to 0.8)	0.4 (0.3 to 0.5)	**-46.6 (-64.62 to -26.37)**
HIV/AIDS resulting in mycobacterial infection	<0.05 (0.0 to <0.05)	<0.05 (0.0 to <0.05)	**-60.0 (-74.51 to -42.40)**	0.1 (0.0 to 0.1)	<0.05 (0.0 to <0.05)	**-65.8 (-77.54 to -52.15)**
HIV/AIDS resulting in other diseases	0.3 (0.3 to 0.4)	0.2 (0.1 to 0.3)	**-35.4 (-58.58 to -8.13)**	0.6 (0.5 to 0.7)	0.3 (0.2 to 0.4)	**-44.9 (-63.45 to -23.91)**
**Diarrhea, lower respiratory, and other common infectious diseases**	33.5 (31.5 to 41.3)	36.2 (32.8 to 44.6)	8.1 (-4.52 to 19.41)	44.8 (42.2 to 55.0)	34.2 (31.1 to 41.5)	**-23.5 (-32.61 to -16.04)**
Diarrheal diseases	0.2 (0.2 to 0.2)	1.7 (1.5 to 2.0)	**779.3 (652.23 to 917.10)**	0.3 (0.3 to 0.3)	1.7 (1.5 to 2.0)	**462.3 (384.77 to 545.60)**
Intestinal infectious diseases	0.1 (0.0 to 0.1)	0.1 (0.0 to 0.1)	15.3 (-12.07 to 41.88)	0.1 (0.1 to 0.3)	0.1 (0.0 to 0.3)	5.6 (-19.73 to 31.70)
Typhoid fever	<0.05 (0.0 to 0.1)	<0.05 (0.0 to 0.1)	**21.3 (1.19 to 40.51)**	0.1 (0.0 to 0.2)	0.1 (0.0 to 0.2)	10.0 (-9.44 to 30.10)
Paratyphoid fever	<0.05 (0.0 to <0.05)	<0.05 (0.0 to <0.05)	**74.7 (42.53 to 109.92)**	<0.05 (0.0 to <0.05)	<0.05 (0.0 to 0.1)	**62.3 (30.71 to 96.93)**
Other intestinal infectious diseases	<0.05 (0.0 to <0.05)	<0.05 (0.0 to <0.05)	-37.8 (-85.47 to 62.09)	<0.05 (0.0 to <0.05)	<0.05 (0.0 to <0.05)	-46.8 (-88.17 to 46.92)
Lower respiratory infections	32.6 (30.6 to 40.3)	33.9 (30.5 to 42.4)	4.2 (-8.36 to 15.80)	43.0 (40.4 to 53.2)	31.6 (28.5 to 38.9)	**-26.3 (-35.33 to -18.88)**
Upper respiratory infections	<0.05 (0.0 to <0.05)	<0.05 (0.0 to <0.05)	-24.2 (-56.78 to 22.22)	<0.05 (0.0 to <0.05)	<0.05 (0.0 to <0.05)	**-44.8 (-65.12 to -15.58)**
Otitis media	<0.05 (0.0 to <0.05)	<0.05 (0.0 to <0.05)	**-56.1 (-61.69 to -50.55)**	0.1 (0.1 to 0.1)	<0.05 (0.0 to <0.05)	**-64.3 (-70.30 to -58.98)**
Meningitis	0.5 (0.4 to 0.5)	0.3 (0.2 to 0.3)	**-44.8 (-50.68 to -27.55)**	1.0 (0.9 to 1.1)	0.4 (0.4 to 0.5)	**-56.3 (-61.29 to -46.29)**
Pneumococcal meningitis	0.1 (0.1 to 0.1)	<0.05 (0.0 to 0.1)	**-43.2 (-51.43 to -19.63)**	0.2 (0.1 to 0.2)	0.1 (0.1 to 0.1)	**-55.4 (-61.79 to -41.93)**
H influenzae type B meningitis	0.1 (0.1 to 0.1)	<0.05 (0.0 to <0.05)	**-49.5 (-57.65 to -40.18)**	0.2 (0.1 to 0.2)	0.1 (0.1 to 0.1)	**-57.8 (-65.28 to -50.09)**
Meningococcal meningitis	0.2 (0.1 to 0.2)	0.1 (0.1 to 0.1)	**-43.4 (-50.39 to -22.84)**	0.3 (0.3 to 0.3)	0.1 (0.1 to 0.2)	**-55.7 (-61.39 to -43.75)**
Other meningitis	0.2 (0.2 to 0.2)	0.1 (0.1 to 0.1)	**-44.5 (-50.43 to -25.06)**	0.3 (0.3 to 0.4)	0.2 (0.1 to 0.2)	**-56.2 (-61.30 to -44.00)**
Encephalitis	0.1 (0.1 to 0.1)	0.1 (0.1 to 0.1)	37.0 (-32.22 to 78.09)	0.2 (0.1 to 0.2)	0.2 (0.1 to 0.2)	10.4 (-44.45 to 44.82)
Diphtheria	<0.05 (0.0 to <0.05)	<0.05 (0.0 to <0.05)	**-58.3 (-80.41 to -21.36)**	<0.05 (0.0 to <0.05)	<0.05 (0.0 to <0.05)	**-64.0 (-83.93 to -28.94)**
Whooping cough	<0.05 (0.0 to <0.05)	<0.05 (0.0 to <0.05)	33.6 (-49.82 to 156.24)	<0.05 (0.0 to <0.05)	<0.05 (0.0 to <0.05)	34.6 (-56.65 to 175.35)
Tetanus	<0.05 (0.0 to <0.05)	<0.05 (0.0 to <0.05)	-1.3 (-43.49 to 21.41)	<0.05 (0.0 to <0.05)	<0.05 (0.0 to <0.05)	**-13.5 (-53.81 to -0.18)**
Measles	<0.05 (0.0 to <0.05)	<0.05 (0.0 to <0.05)	**-96.2 (-98.39 to -92.35)**	<0.05 (0.0 to <0.05)	<0.05 (0.0 to <0.05)	**-96.3 (-98.43 to -92.53)**
Varicella and herpes zoster	<0.05 (0.0 to 0.1)	0.1 (0.0 to 0.1)	18.5 (-47.31 to 121.11)	0.1 (0.0 to 0.1)	0.1 (0.0 to 0.1)	-13.5 (-54.07 to 42.06)
**Neglected tropical diseases and malaria**	<0.05 (0.0 to <0.05)	<0.05 (0.0 to 0.1)	**61.1 (4.45 to 86.51)**	0.1 (0.0 to 0.1)	0.1 (0.1 to 0.1)	**36.9 (-13.78 to 56.32)**
Cysticercosis	<0.05 (0.0 to <0.05)	<0.05 (0.0 to <0.05)	**-76.3 (-78.65 to -73.85)**	<0.05 (0.0 to <0.05)	<0.05 (0.0 to <0.05)	**-81.1 (-82.97 to -79.13)**
Cystic echinococcosis	<0.05 (0.0 to <0.05)	<0.05 (0.0 to <0.05)	**-53.7 (-57.42 to -50.13)**	<0.05 (0.0 to <0.05)	<0.05 (0.0 to <0.05)	**-63.6 (-66.56 to -60.70)**
Dengue	<0.05 (0.0 to <0.05)	<0.05 (0.0 to <0.05)	-3.0 (-38.67 to 7.90)	<0.05 (0.0 to <0.05)	<0.05 (0.0 to <0.05)	**-14.7 (-47.26 to -4.84)**
Rabies	<0.05 (0.0 to <0.05)	<0.05 (0.0 to <0.05)	-11.1 (-50.03 to 7.40)	<0.05 (0.0 to <0.05)	<0.05 (0.0 to <0.05)	**-22.1 (-56.59 to -5.26)**
Other neglected tropical diseases	<0.05 (0.0 to <0.05)	<0.05 (0.0 to <0.05)	**427.9 (117.92 to 539.42)**	<0.05 (0.0 to <0.05)	<0.05 (0.0 to 0.1)	**307.1 (67.66 to 382.56)**
**Maternal disorders**	0.1 (0.1 to 0.1)	<0.05 (0.0 to <0.05)	**-44.6 (-52.69 to -36.11)**	0.1 (0.1 to 0.1)	0.1 (0.1 to 0.1)	**-46.9 (-54.65 to -38.67)**
Maternal hemorrhage	<0.05 (0.0 to <0.05)	<0.05 (0.0 to <0.05)	**-51.0 (-62.69 to -37.37)**	<0.05 (0.0 to <0.05)	<0.05 (0.0 to <0.05)	**-53.0 (-64.20 to -39.73)**
Maternal sepsis and other maternal infections	<0.05 (0.0 to <0.05)	<0.05 (0.0 to <0.05)	**-54.6 (-64.73 to -42.49)**	<0.05 (0.0 to <0.05)	<0.05 (0.0 to <0.05)	**-56.4 (-66.16 to -44.83)**
Maternal hypertensive disorders	<0.05 (0.0 to <0.05)	<0.05 (0.0 to <0.05)	**-45.4 (-55.80 to -31.77)**	<0.05 (0.0 to <0.05)	<0.05 (0.0 to <0.05)	**-46.7 (-56.74 to -33.23)**
Obstructed labor	<0.05 (0.0 to <0.05)	<0.05 (0.0 to <0.05)	**-43.2 (-53.58 to -29.64)**	<0.05 (0.0 to <0.05)	<0.05 (0.0 to <0.05)	**-45.5 (-55.33 to -32.60)**
Complications of abortion	<0.05 (0.0 to <0.05)	<0.05 (0.0 to <0.05)	**-43.3 (-53.43 to -30.11)**	<0.05 (0.0 to <0.05)	<0.05 (0.0 to <0.05)	**-47.1 (-56.59 to -34.68)**
Indirect maternal deaths	<0.05 (0.0 to <0.05)	<0.05 (0.0 to <0.05)	**-38.7 (-52.75 to -22.68)**	<0.05 (0.0 to <0.05)	<0.05 (0.0 to <0.05)	**-40.6 (-54.15 to -25.09)**
Late maternal deaths	<0.05 (0.0 to <0.05)	<0.05 (0.0 to <0.05)	**-33.5 (-49.70 to -11.05)**	<0.05 (0.0 to <0.05)	<0.05 (0.0 to <0.05)	**-36.2 (-51.64 to -14.28)**
Maternal deaths aggravated by HIV/AIDS	<0.05 (0.0 to <0.05)	<0.05 (0.0 to <0.05)	**26.0 (6.01 to 49.12)**	<0.05 (0.0 to <0.05)	<0.05 (0.0 to <0.05)	**17.4 (-0.91 to 39.47)**
Other maternal disorders	<0.05 (0.0 to <0.05)	<0.05 (0.0 to <0.05)	**-41.7 (-53.17 to -28.49)**	<0.05 (0.0 to <0.05)	<0.05 (0.0 to <0.05)	**-43.4 (-54.43 to -30.61)**
**Neonatal disorders**	2.6 (2.4 to 2.8)	1.4 (1.1 to 1.7)	**-45.7 (-56.49 to -31.87)**	7.4 (7.0 to 7.9)	3.9 (3.2 to 4.9)	**-46.9 (-57.50 to -33.43)**
Preterm birth complications	1.7 (1.5 to 2.0)	1.0 (0.8 to 1.3)	**-39.1 (-53.54 to -20.68)**	4.9 (4.4 to 5.7)	2.9 (2.3 to 3.7)	**-40.5 (-54.60 to -22.51)**
Neonatal encephalopathy due to birth asphyxia and trauma	0.6 (0.4 to 0.7)	0.2 (0.1 to 0.3)	**-67.2 (-76.66 to -50.67)**	1.8 (1.3 to 2.1)	0.6 (0.4 to 0.8)	**-67.9 (-77.20 to -51.80)**
Neonatal sepsis and other neonatal infections	0.1 (0.0 to 0.1)	0.1 (0.0 to 0.1)	-29.3 (-64.56 to 31.00)	0.3 (0.1 to 0.4)	0.2 (0.1 to 0.4)	-30.9 (-65.38 to 27.96)
Hemolytic disease and other neonatal jaundice	<0.05 (0.0 to <0.05)	<0.05 (0.0 to <0.05)	**-58.3 (-79.24 to -25.27)**	<0.05 (0.0 to <0.05)	<0.05 (0.0 to <0.05)	**-59.3 (-79.74 to -26.96)**
Other neonatal disorders	0.1 (0.1 to 0.2)	0.1 (0.1 to 0.1)	-27.1 (-55.05 to 5.60)	0.4 (0.3 to 0.5)	0.3 (0.2 to 0.4)	**-28.8 (-56.05 to 3.15)**
**Nutritional deficiencies**	1.0 (0.8 to 1.1)	0.6 (0.5 to 0.7)	**-43.0 (-51.33 to -18.71)**	1.3 (1.1 to 1.5)	0.5 (0.5 to 0.7)	**-59.5 (-65.20 to -43.40)**
Protein-energy malnutrition	0.1 (0.1 to 0.2)	0.1 (0.0 to 0.1)	-19.1 (-52.38 to 12.66)	0.1 (0.1 to 0.2)	0.1 (0.0 to 0.1)	**-41.7 (-64.62 to -20.17)**
Iodine deficiency	<0.05 (0.0 to <0.05)	<0.05 (0.0 to <0.05)	-22.5 (-79.10 to 108.29)	<0.05 (0.0 to <0.05)	<0.05 (0.0 to <0.05)	-44.9 (-81.90 to 33.53)
Iron-deficiency anemia	0.7 (0.5 to 0.8)	0.4 (0.3 to 0.6)	**-40.0 (-50.53 to -16.97)**	0.9 (0.7 to 1.1)	0.4 (0.3 to 0.5)	**-57.7 (-65.02 to -42.52)**
Other nutritional deficiencies	0.2 (0.1 to 0.3)	0.1 (0.0 to 0.1)	**-66.9 (-81.81 to -20.47)**	0.2 (0.1 to 0.4)	0.1 (0.0 to 0.1)	**-76.1 (-86.49 to -44.79)**
**Other communicable, maternal, neonatal, and nutritional diseases**	0.5 (0.4 to 0.6)	0.5 (0.4 to 0.6)	13.2 (-24.64 to 41.97)	0.8 (0.7 to 1.1)	0.7 (0.5 to 0.9)	-11.3 (-41.00 to 12.27)
Sexually transmitted diseases excluding HIV	0.1 (0.1 to 0.1)	0.1 (0.0 to 0.1)	-8.6 (-34.30 to 30.67)	0.1 (0.1 to 0.1)	0.1 (0.1 to 0.1)	**-30.5 (-49.68 to -5.61)**
Syphilis	<0.05 (0.0 to 0.1)	<0.05 (0.0 to 0.1)	-8.4 (-35.03 to 32.33)	0.1 (0.1 to 0.1)	0.1 (0.0 to 0.1)	**-30.8 (-51.22 to -3.81)**
Chlamydial infection	<0.05 (0.0 to <0.05)	<0.05 (0.0 to <0.05)	-0.5 (-28.87 to 33.18)	<0.05 (0.0 to <0.05)	<0.05 (0.0 to <0.05)	**-22.9 (-44.13 to 0.91)**

Males

Cause	Deaths (in thousands) 1990	Deaths (in thousands) 2013	Deaths (in thousands) % change	Age-standardized rate (per 100,000) 1990	Age-standardized rate (per 100,000) 2013	Age-standardized rate (per 100,000) % change
**All causes**	261.6 (259.8 to 263.2)	232.2 (220.5 to 243.4)	**-11.2 (-15.92 to -6.97)**	962.2 (955.9 to 968.4)	580.4 (551.4 to 609.7)	**-39.7 (-42.87 to -36.66)**
**Communicable, maternal, neonatal, and nutritional diseases**	15.1 (14.1 to 19.3)	16.2 (14.5 to 18.9)	7.5 (-8.59 to 19.26)	61.8 (57.7 to 77.7)	41.4 (37.1 to 47.1)	**-32.7 (-42.44 to -25.66)**
**HIV/AIDS and tuberculosis**	0.6 (0.6 to 0.7)	0.4 (0.3 to 0.5)	**-38.5 (-53.07 to -24.46)**	2.4 (2.1 to 2.6)	1.1 (0.9 to 1.3)	**-53.5 (-63.86 to -42.31)**
Tuberculosis	0.4 (0.3 to 0.4)	0.2 (0.2 to 0.3)	**-34.7 (-51.68 to -21.98)**	1.3 (1.2 to 1.5)	0.6 (0.4 to 0.7)	**-54.4 (-65.64 to -45.95)**
HIV/AIDS	0.3 (0.2 to 0.3)	0.2 (0.1 to 0.2)	**-42.9 (-63.96 to -15.69)**	1.1 (0.9 to 1.3)	0.5 (0.4 to 0.7)	**-52.0 (-69.08 to -31.31)**
HIV/AIDS resulting in mycobacterial infection	<0.05 (0.0 to <0.05)	<0.05 (0.0 to <0.05)	**-62.8 (-76.46 to -44.93)**	0.1 (0.1 to 0.1)	<0.05 (0.0 to <0.05)	**-68.7 (-79.65 to -54.30)**
HIV/AIDS resulting in other diseases	0.3 (0.2 to 0.3)	0.1 (0.1 to 0.2)	**-41.0 (-62.68 to -13.03)**	1.0 (0.8 to 1.2)	0.5 (0.3 to 0.7)	**-50.4 (-67.94 to -29.08)**
**Diarrhea, lower respiratory, and other common infectious diseases**	12.5 (11.5 to 16.6)	14.6 (12.9 to 17.3)	**17.3 (-2.01 to 31.32)**	48.9 (45.0 to 64.7)	34.6 (30.6 to 40.6)	**-28.9 (-40.16 to -20.56)**
Diarrheal diseases	0.1 (0.1 to 0.1)	0.6 (0.5 to 0.7)	**658.9 (518.57 to 829.34)**	0.3 (0.3 to 0.4)	1.5 (1.3 to 1.8)	**359.3 (277.00 to 458.79)**
Intestinal infectious diseases	<0.05 (0.0 to 0.1)	<0.05 (0.0 to 0.1)	27.6 (-10.86 to 71.65)	0.1 (0.1 to 0.3)	0.2 (0.1 to 0.3)	15.3 (-20.36 to 55.50)
Typhoid fever	<0.05 (0.0 to <0.05)	<0.05 (0.0 to 0.1)	**32.3 (1.04 to 64.28)**	0.1 (0.0 to 0.2)	0.1 (0.0 to 0.3)	19.4 (-9.59 to 51.55)
Paratyphoid fever	<0.05 (0.0 to <0.05)	<0.05 (0.0 to <0.05)	**115.9 (62.33 to 169.84)**	<0.05 (0.0 to <0.05)	<0.05 (0.0 to 0.1)	**98.4 (46.99 to 153.78)**
Other intestinal infectious diseases	<0.05 (0.0 to <0.05)	<0.05 (0.0 to <0.05)	-26.9 (-91.04 to 165.92)	<0.05 (0.0 to <0.05)	<0.05 (0.0 to <0.05)	-38.7 (-93.32 to 114.66)
Lower respiratory infections	12.0 (11.0 to 16.2)	13.7 (12.0 to 16.4)	14.4 (-4.87 to 28.97)	46.9 (43.1 to 62.7)	32.1 (28.3 to 38.3)	**-31.2 (-42.51 to -22.57)**
Upper respiratory infections	<0.05 (0.0 to <0.05)	<0.05 (0.0 to <0.05)	-25.9 (-61.88 to 37.67)	<0.05 (0.0 to <0.05)	<0.05 (0.0 to <0.05)	**-49.1 (-73.12 to -9.31)**
Otitis media	<0.05 (0.0 to <0.05)	<0.05 (0.0 to <0.05)	**-66.9 (-71.77 to -61.63)**	0.1 (0.1 to 0.1)	<0.05 (0.0 to <0.05)	**-73.0 (-77.70 to -68.13)**
Meningitis	0.3 (0.2 to 0.3)	0.1 (0.1 to 0.2)	**-45.0 (-52.47 to -30.02)**	1.1 (0.9 to 1.3)	0.5 (0.4 to 0.6)	**-56.3 (-62.54 to -47.38)**
Pneumococcal meningitis	<0.05 (0.0 to <0.05)	<0.05 (0.0 to <0.05)	**-45.7 (-55.32 to -29.18)**	0.2 (0.1 to 0.2)	0.1 (0.1 to 0.1)	**-57.3 (-65.39 to -44.36)**
H influenzae type B meningitis	<0.05 (0.0 to 0.1)	<0.05 (0.0 to <0.05)	**-48.3 (-59.65 to -36.63)**	0.2 (0.1 to 0.3)	0.1 (0.1 to 0.1)	**-56.4 (-66.68 to -45.53)**
Meningococcal meningitis	0.1 (0.1 to 0.1)	<0.05 (0.0 to 0.1)	**-43.3 (-54.11 to -25.16)**	0.3 (0.3 to 0.4)	0.2 (0.1 to 0.2)	**-55.6 (-64.19 to -44.20)**
Other meningitis	0.1 (0.1 to 0.1)	0.1 (0.0 to 0.1)	**-44.3 (-52.55 to -28.29)**	0.4 (0.3 to 0.5)	0.2 (0.1 to 0.2)	**-56.0 (-62.54 to -45.86)**
Encephalitis	<0.05 (0.0 to 0.1)	0.1 (0.0 to 0.1)	33.4 (-35.58 to 74.12)	0.2 (0.1 to 0.3)	0.2 (0.1 to 0.2)	4.3 (-48.45 to 37.54)
Diphtheria	<0.05 (0.0 to <0.05)	<0.05 (0.0 to <0.05)	**-57.1 (-85.10 to -6.57)**	<0.05 (0.0 to <0.05)	<0.05 (0.0 to <0.05)	**-62.7 (-87.77 to -15.25)**
Whooping cough	<0.05 (0.0 to <0.05)	<0.05 (0.0 to <0.05)	36.5 (-55.90 to 191.76)	<0.05 (0.0 to <0.05)	<0.05 (0.0 to <0.05)	37.7 (-60.86 to 214.74)
Tetanus	<0.05 (0.0 to <0.05)	<0.05 (0.0 to <0.05)	4.4 (-50.32 to 33.62)	<0.05 (0.0 to <0.05)	<0.05 (0.0 to <0.05)	-12.4 (-59.23 to 3.70)
Measles	<0.05 (0.0 to <0.05)	<0.05 (0.0 to <0.05)	**-96.1 (-98.34 to -92.12)**	<0.05 (0.0 to <0.05)	<0.05 (0.0 to <0.05)	**-96.2 (-98.39 to -92.32)**
Varicella and herpes zoster	<0.05 (0.0 to <0.05)	<0.05 (0.0 to <0.05)	32.9 (-43.14 to 173.11)	0.1 (0.0 to 0.1)	0.1 (0.0 to 0.1)	-11.8 (-59.41 to 71.76)
**Neglected tropical diseases and malaria**	<0.05 (0.0 to <0.05)	<0.05 (0.0 to <0.05)	**92.8 (48.19 to 137.20)**	0.1 (0.1 to 0.1)	0.1 (0.1 to 0.1)	**55.3 (9.74 to 87.41)**
Cysticercosis	<0.05 (0.0 to <0.05)	<0.05 (0.0 to <0.05)	**-77.3 (-79.56 to -74.85)**	<0.05 (0.0 to <0.05)	<0.05 (0.0 to <0.05)	**-82.2 (-84.02 to -80.27)**
Cystic echinococcosis	<0.05 (0.0 to <0.05)	<0.05 (0.0 to <0.05)	**-55.7 (-59.29 to -51.93)**	<0.05 (0.0 to <0.05)	<0.05 (0.0 to <0.05)	**-66.1 (-68.87 to -63.18)**
Dengue	<0.05 (0.0 to <0.05)	<0.05 (0.0 to <0.05)	-2.4 (-42.28 to 9.94)	<0.05 (0.0 to <0.05)	<0.05 (0.0 to <0.05)	**-15.0 (-49.96 to -3.06)**
Rabies	<0.05 (0.0 to <0.05)	<0.05 (0.0 to <0.05)	-13.8 (-54.84 to 8.76)	<0.05 (0.0 to <0.05)	<0.05 (0.0 to <0.05)	**-25.3 (-61.28 to -5.29)**
Other neglected tropical diseases	<0.05 (0.0 to <0.05)	<0.05 (0.0 to <0.05)	**534.1 (224.10 to 703.19)**	<0.05 (0.0 to <0.05)	0.1 (0.0 to 0.1)	**381.6 (124.85 to 501.51)**
**Maternal disorders**	--	--	--	--	--	--
Maternal hemorrhage	--	--	--	--	--	--
Maternal sepsis and other maternal infections	--	--	--	--	--	--
Maternal hypertensive disorders	--	--	--	--	--	--
Obstructed labor	--	--	--	--	--	--
Complications of abortion	--	--	--	--	--	--
Indirect maternal deaths	--	--	--	--	--	--
Late maternal deaths	--	--	--	--	--	--
Maternal deaths aggravated by HIV/AIDS	--	--	--	--	--	--
Other maternal disorders	--	--	--	--	--	--
**Neonatal disorders**	1.5 (1.4 to 1.6)	0.8 (0.6 to 1.0)	**-46.5 (-57.49 to -32.48)**	8.4 (7.9 to 9.1)	4.4 (3.5 to 5.5)	**-47.8 (-58.44 to -34.01)**
Preterm birth complications	1.0 (0.9 to 1.2)	0.6 (0.5 to 0.8)	**-39.1 (-55.56 to -18.76)**	5.6 (4.9 to 6.8)	3.3 (2.5 to 4.2)	**-40.5 (-56.55 to -20.59)**
Neonatal encephalopathy due to birth asphyxia and trauma	0.4 (0.2 to 0.5)	0.1 (0.1 to 0.2)	**-69.3 (-80.11 to -47.36)**	2.1 (1.2 to 2.6)	0.6 (0.4 to 0.9)	**-70.0 (-80.56 to -48.53)**
Neonatal sepsis and other neonatal infections	0.1 (0.0 to 0.1)	<0.05 (0.0 to 0.1)	-31.5 (-68.70 to 30.90)	0.3 (0.1 to 0.5)	0.2 (0.1 to 0.5)	-33.1 (-69.42 to 27.92)
Hemolytic disease and other neonatal jaundice	<0.05 (0.0 to <0.05)	<0.05 (0.0 to <0.05)	**-53.2 (-75.34 to -17.32)**	<0.05 (0.0 to <0.05)	<0.05 (0.0 to <0.05)	**-54.3 (-75.89 to -19.10)**
Other neonatal disorders	0.1 (0.1 to 0.1)	0.1 (0.0 to 0.1)	-27.9 (-58.89 to 8.80)	0.4 (0.3 to 0.7)	0.3 (0.2 to 0.5)	-29.5 (-59.80 to 6.35)
**Nutritional deficiencies**	0.3 (0.2 to 0.3)	0.2 (0.1 to 0.3)	**-32.9 (-49.19 to 1.09)**	1.1 (0.6 to 1.3)	0.4 (0.3 to 0.7)	**-59.0 (-68.79 to -37.86)**
Protein-energy malnutrition	<0.05 (0.0 to 0.1)	<0.05 (0.0 to 0.1)	12.7 (-30.24 to 70.85)	0.1 (0.1 to 0.2)	0.1 (0.0 to 0.2)	-27.3 (-53.87 to 8.98)
Iodine deficiency	<0.05 (0.0 to <0.05)	<0.05 (0.0 to <0.05)	-12.5 (-80.13 to 147.65)	<0.05 (0.0 to <0.05)	<0.05 (0.0 to <0.05)	-40.4 (-86.08 to 60.07)
Iron-deficiency anemia	0.2 (0.1 to 0.3)	0.1 (0.1 to 0.2)	**-35.2 (-54.41 to 6.33)**	0.8 (0.4 to 1.0)	0.3 (0.2 to 0.5)	**-61.0 (-72.57 to -35.79)**
Other nutritional deficiencies	<0.05 (0.0 to 0.1)	<0.05 (0.0 to 0.1)	-50.2 (-75.29 to 20.35)	0.2 (0.1 to 0.3)	0.1 (0.0 to 0.1)	**-69.3 (-84.37 to -26.53)**
**Other communicable, maternal, neonatal, and nutritional diseases**	0.3 (0.2 to 0.4)	0.3 (0.2 to 0.3)	12.6 (-29.88 to 49.55)	1.0 (0.9 to 1.4)	0.8 (0.6 to 1.0)	-13.9 (-46.15 to 13.95)
Sexually transmitted diseases excluding HIV	<0.05 (0.0 to <0.05)	<0.05 (0.0 to <0.05)	**-48.0 (-67.10 to -13.51)**	0.1 (0.0 to 0.1)	<0.05 (0.0 to 0.1)	**-54.7 (-70.98 to -26.55)**
Syphilis	<0.05 (0.0 to <0.05)	<0.05 (0.0 to <0.05)	**-47.2 (-68.11 to -11.30)**	0.1 (0.0 to 0.1)	<0.05 (0.0 to <0.05)	**-53.6 (-71.97 to -23.16)**
Chlamydial infection	--	--	--	--	--	--

Females

Cause	Deaths (in thousands) 1990	Deaths (in thousands) 2013	Deaths (in thousands) % change	Age-standardized rate (per 100,000) 1990	Age-standardized rate (per 100,000) 2013	Age-standardized rate (per 100,000) % change
**All causes**	273.8 (272.1 to 275.5)	247.0 (235.6 to 260.9)	**-9.8 (-13.97 to -4.90)**	616.5 (612.4 to 620.5)	439.1 (417.8 to 465.1)	**-28.8 (-32.34 to -24.66)**
**Communicable, maternal, neonatal, and nutritional diseases**	23.5 (21.9 to 28.7)	23.1 (20.1 to 30.0)	-1.3 (-14.96 to 13.99)	51.6 (48.3 to 62.1)	38.9 (34.2 to 47.8)	**-24.5 (-34.01 to -15.22)**
**HIV/AIDS and tuberculosis**	0.3 (0.3 to 0.3)	0.2 (0.2 to 0.2)	**-29.7 (-44.20 to -16.04)**	0.9 (0.8 to 1.0)	0.5 (0.4 to 0.6)	**-42.0 (-54.04 to -28.80)**
Tuberculosis	0.2 (0.2 to 0.3)	0.1 (0.1 to 0.2)	**-33.9 (-50.14 to -22.35)**	0.6 (0.6 to 0.7)	0.3 (0.2 to 0.4)	**-48.2 (-60.76 to -39.91)**
HIV/AIDS	0.1 (0.1 to 0.1)	0.1 (0.0 to 0.1)	-15.9 (-47.51 to 31.72)	0.3 (0.2 to 0.3)	0.2 (0.1 to 0.3)	-28.2 (-53.34 to 8.67)
HIV/AIDS resulting in mycobacterial infection	<0.05 (0.0 to <0.05)	<0.05 (0.0 to <0.05)	**-45.6 (-66.41 to -14.86)**	<0.05 (0.0 to <0.05)	<0.05 (0.0 to <0.05)	**-53.4 (-70.27 to -28.33)**
HIV/AIDS resulting in other diseases	0.1 (0.0 to 0.1)	0.1 (0.0 to 0.1)	-13.8 (-46.26 to 34.53)	0.3 (0.2 to 0.3)	0.2 (0.1 to 0.3)	-26.4 (-52.22 to 11.35)
**Diarrhea, lower respiratory, and other common infectious diseases**	21.0 (19.5 to 26.1)	21.6 (18.6 to 28.6)	2.9 (-11.47 to 19.74)	41.9 (38.9 to 52.1)	33.5 (29.1 to 43.4)	**-19.9 (-30.94 to -7.91)**
Diarrheal diseases	0.1 (0.1 to 0.1)	1.1 (1.0 to 1.4)	**863.2 (689.46 to 1,074.22)**	0.3 (0.3 to 0.3)	1.9 (1.6 to 2.2)	**548.6 (440.48 to 679.52)**
Intestinal infectious diseases	<0.05 (0.0 to 0.1)	<0.05 (0.0 to 0.1)	3.7 (-28.39 to 32.79)	0.1 (0.0 to 0.3)	0.1 (0.0 to 0.3)	-4.3 (-33.13 to 21.95)
Typhoid fever	<0.05 (0.0 to <0.05)	<0.05 (0.0 to <0.05)	10.3 (-10.11 to 32.34)	0.1 (0.0 to 0.2)	0.1 (0.0 to 0.2)	0.2 (-18.86 to 21.33)
Paratyphoid fever	<0.05 (0.0 to <0.05)	<0.05 (0.0 to <0.05)	**35.4 (9.58 to 67.35)**	<0.05 (0.0 to <0.05)	<0.05 (0.0 to <0.05)	**27.2 (1.44 to 57.57)**
Other intestinal infectious diseases	<0.05 (0.0 to <0.05)	<0.05 (0.0 to <0.05)	-26.3 (-93.19 to 207.92)	<0.05 (0.0 to <0.05)	<0.05 (0.0 to <0.05)	-34.9 (-94.79 to 191.08)
Lower respiratory infections	20.6 (19.0 to 25.6)	20.2 (17.3 to 27.2)	-1.5 (-15.87 to 15.55)	40.3 (37.3 to 50.4)	30.9 (26.6 to 40.7)	**-23.3 (-34.22 to -11.56)**
Upper respiratory infections	<0.05 (0.0 to <0.05)	<0.05 (0.0 to <0.05)	-18.3 (-67.41 to 70.78)	<0.05 (0.0 to <0.05)	<0.05 (0.0 to <0.05)	-38.9 (-70.00 to 12.77)
Otitis media	<0.05 (0.0 to <0.05)	<0.05 (0.0 to <0.05)	**-41.6 (-50.20 to -32.58)**	<0.05 (0.0 to 0.1)	<0.05 (0.0 to <0.05)	**-52.5 (-61.00 to -43.47)**
Meningitis	0.2 (0.2 to 0.3)	0.1 (0.1 to 0.2)	**-44.4 (-52.57 to -17.90)**	0.9 (0.7 to 0.9)	0.4 (0.3 to 0.5)	**-56.5 (-62.72 to -40.39)**
Pneumococcal meningitis	<0.05 (0.0 to <0.05)	<0.05 (0.0 to <0.05)	**-40.1 (-54.10 to 0.24)**	0.1 (0.1 to 0.2)	0.1 (0.1 to 0.1)	**-53.1 (-63.06 to -29.94)**
H influenzae type B meningitis	<0.05 (0.0 to <0.05)	<0.05 (0.0 to <0.05)	**-50.5 (-60.73 to -37.11)**	0.1 (0.1 to 0.2)	0.1 (0.0 to 0.1)	**-59.7 (-68.28 to -49.30)**
Meningococcal meningitis	0.1 (0.1 to 0.1)	<0.05 (0.0 to 0.1)	**-43.3 (-53.67 to -12.32)**	0.3 (0.2 to 0.3)	0.1 (0.1 to 0.2)	**-55.7 (-63.62 to -36.73)**
Other meningitis	0.1 (0.1 to 0.1)	<0.05 (0.0 to 0.1)	**-44.6 (-53.71 to -21.10)**	0.3 (0.3 to 0.3)	0.1 (0.1 to 0.2)	**-56.8 (-64.05 to -40.54)**
Encephalitis	<0.05 (0.0 to 0.1)	0.1 (0.0 to 0.1)	41.4 (-30.91 to 90.98)	0.1 (0.1 to 0.2)	0.2 (0.1 to 0.2)	17.3 (-41.34 to 59.38)
Diphtheria	<0.05 (0.0 to <0.05)	<0.05 (0.0 to <0.05)	**-53.5 (-85.68 to 14.21)**	<0.05 (0.0 to <0.05)	<0.05 (0.0 to <0.05)	**-59.7 (-88.04 to 1.58)**
Whooping cough	<0.05 (0.0 to <0.05)	<0.05 (0.0 to <0.05)	31.7 (-51.34 to 166.22)	<0.05 (0.0 to <0.05)	<0.05 (0.0 to <0.05)	32.5 (-58.71 to 189.73)
Tetanus	<0.05 (0.0 to <0.05)	<0.05 (0.0 to <0.05)	-6.0 (-46.49 to 16.24)	<0.05 (0.0 to <0.05)	<0.05 (0.0 to <0.05)	**-13.9 (-54.18 to 0.49)**
Measles	<0.05 (0.0 to <0.05)	<0.05 (0.0 to <0.05)	**-96.2 (-98.42 to -92.55)**	<0.05 (0.0 to <0.05)	<0.05 (0.0 to <0.05)	**-96.3 (-98.46 to -92.72)**
Varicella and herpes zoster	<0.05 (0.0 to 0.1)	<0.05 (0.0 to 0.1)	17.8 (-63.67 to 186.26)	0.1 (0.0 to 0.1)	0.1 (0.0 to 0.1)	-10.6 (-61.59 to 78.15)
**Neglected tropical diseases and malaria**	<0.05 (0.0 to <0.05)	<0.05 (0.0 to <0.05)	**31.0 (-39.92 to 50.09)**	0.1 (0.0 to 0.1)	0.1 (0.0 to 0.1)	18.5 (-47.32 to 35.49)
Cysticercosis	<0.05 (0.0 to <0.05)	<0.05 (0.0 to <0.05)	**-75.4 (-78.20 to -72.57)**	<0.05 (0.0 to <0.05)	<0.05 (0.0 to <0.05)	**-80.1 (-82.41 to -77.74)**
Cystic echinococcosis	<0.05 (0.0 to <0.05)	<0.05 (0.0 to <0.05)	**-51.9 (-56.91 to -47.36)**	<0.05 (0.0 to <0.05)	<0.05 (0.0 to <0.05)	**-61.4 (-65.10 to -57.59)**
Dengue	<0.05 (0.0 to <0.05)	<0.05 (0.0 to <0.05)	-3.1 (-43.99 to 10.56)	<0.05 (0.0 to <0.05)	<0.05 (0.0 to <0.05)	**-14.0 (-49.91 to -1.15)**
Rabies	<0.05 (0.0 to <0.05)	<0.05 (0.0 to <0.05)	-8.0 (-48.93 to 9.77)	<0.05 (0.0 to <0.05)	<0.05 (0.0 to <0.05)	**-18.4 (-55.39 to -2.19)**
Other neglected tropical diseases	<0.05 (0.0 to <0.05)	<0.05 (0.0 to <0.05)	**311.5 (-35.53 to 395.74)**	<0.05 (0.0 to <0.05)	<0.05 (0.0 to <0.05)	**227.2 (-46.59 to 292.45)**
**Maternal disorders**	0.1 (0.1 to 0.1)	<0.05 (0.0 to <0.05)	**-44.6 (-52.69 to -36.11)**	0.3 (0.3 to 0.3)	0.1 (0.1 to 0.2)	**-46.8 (-54.63 to -38.63)**
Maternal hemorrhage	<0.05 (0.0 to <0.05)	<0.05 (0.0 to <0.05)	**-51.0 (-62.69 to -37.37)**	<0.05 (0.0 to <0.05)	<0.05 (0.0 to <0.05)	**-53.0 (-64.16 to -39.71)**
Maternal sepsis and other maternal infections	<0.05 (0.0 to <0.05)	<0.05 (0.0 to <0.05)	**-54.6 (-64.73 to -42.49)**	<0.05 (0.0 to <0.05)	<0.05 (0.0 to <0.05)	**-56.3 (-66.12 to -44.85)**
Maternal hypertensive disorders	<0.05 (0.0 to <0.05)	<0.05 (0.0 to <0.05)	**-45.4 (-55.80 to -31.77)**	<0.05 (0.0 to <0.05)	<0.05 (0.0 to <0.05)	**-46.6 (-56.67 to -33.12)**
Obstructed labor	<0.05 (0.0 to <0.05)	<0.05 (0.0 to <0.05)	**-43.2 (-53.58 to -29.64)**	<0.05 (0.0 to <0.05)	<0.05 (0.0 to <0.05)	**-45.5 (-55.28 to -32.58)**
Complications of abortion	<0.05 (0.0 to <0.05)	<0.05 (0.0 to <0.05)	**-43.3 (-53.43 to -30.11)**	0.1 (0.0 to 0.1)	<0.05 (0.0 to <0.05)	**-47.2 (-56.63 to -34.73)**
Indirect maternal deaths	<0.05 (0.0 to <0.05)	<0.05 (0.0 to <0.05)	**-38.7 (-52.75 to -22.68)**	<0.05 (0.0 to <0.05)	<0.05 (0.0 to <0.05)	**-40.6 (-54.09 to -25.01)**
Late maternal deaths	<0.05 (0.0 to <0.05)	<0.05 (0.0 to <0.05)	**-33.5 (-49.70 to -11.05)**	<0.05 (0.0 to <0.05)	<0.05 (0.0 to <0.05)	**-36.2 (-51.65 to -14.25)**
Maternal deaths aggravated by HIV/AIDS	<0.05 (0.0 to <0.05)	<0.05 (0.0 to <0.05)	**26.0 (6.01 to 49.12)**	<0.05 (0.0 to <0.05)	<0.05 (0.0 to <0.05)	**17.1 (-1.17 to 39.12)**
Other maternal disorders	<0.05 (0.0 to <0.05)	<0.05 (0.0 to <0.05)	**-41.7 (-53.17 to -28.49)**	0.1 (0.1 to 0.1)	<0.05 (0.0 to <0.05)	**-43.3 (-54.38 to -30.50)**
**Neonatal disorders**	1.1 (0.9 to 1.2)	0.6 (0.5 to 0.8)	**-44.5 (-55.44 to -30.39)**	6.3 (5.6 to 7.2)	3.4 (2.7 to 4.3)	**-45.8 (-56.49 to -32.02)**
Preterm birth complications	0.7 (0.6 to 0.9)	0.4 (0.3 to 0.6)	**-39.0 (-52.84 to -21.76)**	4.2 (3.5 to 5.1)	2.5 (1.9 to 3.2)	**-40.4 (-53.93 to -23.56)**
Neonatal encephalopathy due to birth asphyxia and trauma	0.3 (0.2 to 0.4)	0.1 (0.1 to 0.1)	**-63.7 (-73.98 to -49.20)**	1.6 (1.1 to 2.1)	0.6 (0.4 to 0.8)	**-64.5 (-74.59 to -50.40)**
Neonatal sepsis and other neonatal infections	<0.05 (0.0 to 0.1)	<0.05 (0.0 to <0.05)	-24.1 (-59.77 to 39.20)	0.2 (0.1 to 0.4)	0.1 (0.1 to 0.3)	-25.8 (-60.71 to 35.94)
Hemolytic disease and other neonatal jaundice	<0.05 (0.0 to <0.05)	<0.05 (0.0 to <0.05)	**-61.6 (-88.73 to -18.57)**	<0.05 (0.0 to <0.05)	<0.05 (0.0 to <0.05)	**-62.5 (-89.01 to -20.36)**
Other neonatal disorders	<0.05 (0.0 to 0.1)	<0.05 (0.0 to 0.1)	-24.9 (-61.62 to 11.28)	0.3 (0.2 to 0.5)	0.2 (0.1 to 0.3)	-26.6 (-62.50 to 8.70)
**Nutritional deficiencies**	0.7 (0.5 to 0.8)	0.4 (0.3 to 0.5)	**-46.5 (-56.28 to -18.67)**	1.5 (1.1 to 1.6)	0.6 (0.5 to 0.7)	**-58.3 (-65.53 to -38.62)**
Protein-energy malnutrition	0.1 (0.0 to 0.1)	<0.05 (0.0 to 0.1)	**-32.5 (-63.76 to 0.71)**	0.2 (0.1 to 0.3)	0.1 (0.0 to 0.1)	**-47.6 (-70.76 to -23.63)**
Iodine deficiency	<0.05 (0.0 to <0.05)	<0.05 (0.0 to <0.05)	-15.9 (-87.09 to 210.17)	<0.05 (0.0 to <0.05)	<0.05 (0.0 to <0.05)	-40.5 (-86.21 to 80.28)
Iron-deficiency anemia	0.5 (0.3 to 0.6)	0.3 (0.2 to 0.4)	**-41.2 (-54.85 to -13.50)**	1.0 (0.7 to 1.3)	0.5 (0.4 to 0.6)	**-54.3 (-64.36 to -33.98)**
Other nutritional deficiencies	0.1 (0.0 to 0.2)	<0.05 (0.0 to 0.1)	**-72.7 (-87.36 to -32.28)**	0.3 (0.1 to 0.5)	0.1 (0.0 to 0.1)	**-78.4 (-89.33 to -49.14)**
**Other communicable, maternal, neonatal, and nutritional diseases**	0.2 (0.2 to 0.3)	0.3 (0.2 to 0.3)	14.3 (-23.42 to 43.48)	0.7 (0.6 to 0.9)	0.6 (0.4 to 0.7)	-8.6 (-39.09 to 15.91)
Sexually transmitted diseases excluding HIV	<0.05 (0.0 to 0.1)	<0.05 (0.0 to 0.1)	6.0 (-24.81 to 47.92)	0.1 (0.1 to 0.2)	0.1 (0.1 to 0.1)	-18.2 (-41.34 to 9.60)
Syphilis	<0.05 (0.0 to <0.05)	<0.05 (0.0 to 0.1)	7.7 (-24.54 to 52.69)	0.1 (0.1 to 0.1)	0.1 (0.1 to 0.1)	-17.7 (-42.24 to 13.02)
Chlamydial infection	<0.05 (0.0 to <0.05)	<0.05 (0.0 to <0.05)	-0.5 (-28.87 to 33.18)	<0.05 (0.0 to <0.05)	<0.05 (0.0 to <0.05)	-20.3 (-42.07 to 4.55)

Gonococcal infection	<0.05 (0.0 to <0.05)	<0.05 (0.0 to <0.05)	-10.1 (-35.39 to 28.13)	<0.05 (0.0 to <0.05)	<0.05 (0.0 to <0.05)	**-31.1 (-48.72 to -5.28)**	<0.05 (0.0 to <0.05)	<0.05 (0.0 to <0.05)	**-52.1 (-66.70 to -3.62)**	<0.05 (0.0 to <0.05)	<0.05 (0.0 to <0.05)	**-61.7 (-70.65 to -27.10)**	<0.05 (0.0 to <0.05)	<0.05 (0.0 to <0.05)	4.5 (-26.80 to 43.93)	<0.05 (0.0 to <0.05)	<0.05 (0.0 to <0.05)	-17.1 (-40.57 to 9.76)
Other sexually transmitted diseases	<0.05 (0.0 to <0.05)	<0.05 (0.0 to <0.05)	-13.8 (-35.81 to 16.98)	<0.05 (0.0 to <0.05)	<0.05 (0.0 to <0.05)	**-31.8 (-47.30 to -10.61)**	<0.05 (0.0 to <0.05)	<0.05 (0.0 to <0.05)	**-51.4 (-61.15 to -7.89)**	<0.05 (0.0 to <0.05)	<0.05 (0.0 to <0.05)	**-58.0 (-66.08 to -25.83)**	<0.05 (0.0 to <0.05)	<0.05 (0.0 to <0.05)	-1.9 (-29.86 to 30.50)	<0.05 (0.0 to <0.05)	<0.05 (0.0 to <0.05)	-21.0 (-42.57 to 3.99)
Hepatitis	0.1 (0.1 to 0.1)	0.1 (0.1 to 0.1)	**45.0 (28.45 to 72.01)**	0.1 (0.1 to 0.1)	0.1 (0.1 to 0.1)	**16.9 (3.43 to 38.81)**	<0.05 (0.0 to <0.05)	0.1 (0.0 to 0.1)	**29.5 (11.52 to 58.35)**	0.2 (0.2 to 0.2)	0.2 (0.2 to 0.2)	2.7 (-11.12 to 25.17)	<0.05 (0.0 to <0.05)	<0.05 (0.0 to <0.05)	**90.0 (58.75 to 128.84)**	<0.05 (0.0 to 0.1)	0.1 (0.1 to 0.1)	**56.3 (33.63 to 85.43)**
Acute hepatitis A	<0.05 (0.0 to <0.05)	<0.05 (0.0 to <0.05)	**98.4 (70.53 to 133.59)**	<0.05 (0.0 to <0.05)	<0.05 (0.0 to 0.1)	**60.5 (38.31 to 89.47)**	<0.05 (0.0 to <0.05)	<0.05 (0.0 to <0.05)	**75.6 (44.47 to 112.52)**	<0.05 (0.0 to 0.1)	<0.05 (0.0 to 0.1)	**42.9 (18.20 to 72.52)**	<0.05 (0.0 to <0.05)	<0.05 (0.0 to <0.05)	**148.8 (108.75 to 203.89)**	<0.05 (0.0 to <0.05)	<0.05 (0.0 to <0.05)	**99.0 (69.06 to 143.05)**
Acute hepatitis B	<0.05 (0.0 to 0.1)	0.1 (0.0 to 0.1)	**22.3 (2.75 to 46.52)**	0.1 (0.1 to 0.1)	0.1 (0.1 to 0.1)	-3.0 (-18.71 to 16.61)	<0.05 (0.0 to <0.05)	<0.05 (0.0 to <0.05)	10.3 (-9.87 to 37.60)	0.1 (0.1 to 0.2)	0.1 (0.1 to 0.1)	-14.0 (-29.87 to 7.42)	<0.05 (0.0 to <0.05)	<0.05 (0.0 to <0.05)	**60.2 (26.96 to 97.58)**	<0.05 (0.0 to <0.05)	<0.05 (0.0 to 0.1)	**30.2 (5.65 to 57.56)**
Acute hepatitis C	<0.05 (0.0 to <0.05)	<0.05 (0.0 to <0.05)	**178.4 (108.49 to 301.52)**	<0.05 (0.0 to <0.05)	<0.05 (0.0 to <0.05)	**114.9 (61.35 to 203.86)**	<0.05 (0.0 to <0.05)	<0.05 (0.0 to <0.05)	**164.4 (80.64 to 302.14)**	<0.05 (0.0 to <0.05)	<0.05 (0.0 to <0.05)	**102.0 (39.81 to 201.80)**	<0.05 (0.0 to <0.05)	<0.05 (0.0 to <0.05)	**216.9 (98.05 to 380.45)**	<0.05 (0.0 to <0.05)	<0.05 (0.0 to <0.05)	**146.6 (62.72 to 257.00)**
Other infectious diseases	0.4 (0.3 to 0.5)	0.4 (0.2 to 0.5)	13.4 (-35.79 to 55.69)	0.6 (0.5 to 0.9)	0.5 (0.3 to 0.7)	-11.7 (-49.65 to 22.34)	0.2 (0.2 to 0.3)	0.2 (0.1 to 0.3)	16.0 (-38.94 to 65.49)	0.8 (0.6 to 1.2)	0.6 (0.3 to 0.8)	-12.5 (-53.35 to 24.75)	0.2 (0.1 to 0.3)	0.2 (0.1 to 0.2)	11.1 (-37.04 to 52.62)	0.5 (0.4 to 0.7)	0.4 (0.3 to 0.6)	-11.4 (-49.00 to 23.70)
**Non-communicable diseases**	479.5 (470.9 to 483.2)	424.3 (409.4 to 439.6)	**-11.5 (-14.74 to -8.25)**	672.7 (661.3 to 677.9)	443.5 (427.3 to 460.3)	**-34.1 (-36.62 to -31.54)**	235.7 (231.2 to 237.8)	207.0 (196.1 to 217.5)	**-12.2 (-16.70 to -7.75)**	857.2 (840.3 to 865.1)	511.3 (483.6 to 537.2)	**-40.3 (-43.47 to -37.26)**	243.8 (238.3 to 246.4)	217.3 (206.2 to 229.3)	**-10.8 (-15.26 to -5.85)**	546.2 (535.4 to 551.6)	386.8 (366.1 to 409.4)	**-29.2 (-32.87 to -25.06)**
**Neoplasms**	132.1 (128.2 to 138.6)	134.0 (125.2 to 140.8)	1.5 (-5.11 to 6.59)	193.4 (187.9 to 202.3)	151.0 (141.5 to 158.7)	**-21.9 (-26.75 to -18.06)**	68.8 (66.7 to 71.5)	70.7 (65.8 to 76.0)	2.7 (-4.46 to 9.38)	245.3 (237.4 to 255.2)	177.6 (165.2 to 190.4)	**-27.6 (-32.74 to -22.93)**	63.3 (60.2 to 69.7)	63.4 (55.8 to 68.5)	0.2 (-11.69 to 9.41)	162.2 (154.3 to 177.0)	130.5 (116.0 to 141.4)	**-19.5 (-28.16 to -12.16)**
Esophageal cancer	5.0 (4.6 to 5.5)	6.6 (5.9 to 8.3)	**32.8 (17.83 to 55.77)**	7.2 (6.7 to 7.9)	7.5 (6.6 to 9.2)	3.1 (-9.06 to 20.85)	3.0 (2.6 to 3.4)	4.5 (3.8 to 6.0)	**50.7 (28.37 to 86.08)**	10.5 (9.1 to 11.9)	11.4 (9.7 to 15.0)	8.4 (-7.90 to 33.12)	2.0 (1.9 to 2.2)	2.1 (1.9 to 2.4)	6.6 (-4.61 to 19.74)	4.7 (4.5 to 5.1)	4.0 (3.6 to 4.5)	**-13.8 (-22.74 to -2.80)**
Stomach cancer	9.3 (8.4 to 10.3)	6.0 (5.3 to 6.9)	**-35.4 (-40.90 to -28.32)**	13.0 (11.8 to 14.4)	6.4 (5.6 to 7.2)	**-51.2 (-55.09 to -46.25)**	5.6 (5.4 to 5.8)	3.5 (3.2 to 3.9)	**-38.0 (-43.23 to -30.10)**	19.9 (19.3 to 20.5)	8.6 (7.9 to 9.5)	**-57.0 (-60.66 to -51.51)**	3.7 (2.7 to 4.7)	2.5 (1.9 to 3.4)	**-31.2 (-41.80 to -13.56)**	8.2 (6.2 to 10.6)	4.5 (3.3 to 6.0)	**-45.0 (-52.56 to -31.64)**
Liver cancer	1.4 (1.3 to 1.8)	2.9 (2.1 to 3.3)	**103.3 (42.62 to 145.40)**	2.1 (1.8 to 2.6)	3.3 (2.4 to 3.8)	**56.6 (11.83 to 87.65)**	0.8 (0.7 to 1.2)	1.6 (1.1 to 2.0)	**98.4 (17.80 to 156.03)**	3.0 (2.5 to 4.2)	4.2 (2.7 to 5.2)	43.8 (-14.71 to 84.84)	0.6 (0.5 to 0.8)	1.2 (0.8 to 1.4)	**112.5 (25.73 to 148.91)**	1.4 (1.3 to 1.9)	2.4 (1.6 to 2.8)	**68.7 (2.87 to 96.26)**
Liver cancer due to hepatitis B	0.3 (0.2 to 0.3)	0.6 (0.4 to 0.7)	**132.1 (51.59 to 223.54)**	0.4 (0.3 to 0.5)	0.7 (0.5 to 0.9)	**80.0 (20.84 to 147.95)**	0.2 (0.1 to 0.2)	0.3 (0.2 to 0.5)	**100.5 (17.07 to 199.81)**	0.6 (0.5 to 0.9)	0.9 (0.6 to 1.2)	50.9 (-10.77 to 123.31)	0.1 (0.1 to 0.1)	0.2 (0.1 to 0.3)	**208.8 (61.73 to 393.36)**	0.2 (0.2 to 0.3)	0.5 (0.3 to 0.7)	**138.5 (33.12 to 282.44)**
Liver cancer due to hepatitis C	0.3 (0.3 to 0.4)	1.5 (1.1 to 1.8)	**359.5 (219.29 to 535.78)**	0.5 (0.4 to 0.6)	1.7 (1.3 to 2.0)	**255.3 (150.28 to 386.82)**	0.2 (0.1 to 0.3)	0.8 (0.5 to 1.1)	**330.6 (146.61 to 557.91)**	0.7 (0.5 to 1.0)	2.2 (1.4 to 2.8)	**210.9 (80.60 to 373.79)**	0.1 (0.1 to 0.2)	0.7 (0.5 to 0.8)	**416.9 (194.51 to 572.10)**	0.3 (0.2 to 0.4)	1.3 (0.9 to 1.6)	**313.2 (145.31 to 443.77)**
Liver cancer due to alcohol use	0.7 (0.6 to 0.9)	0.6 (0.4 to 0.8)	-7.9 (-42.40 to 22.56)	1.0 (0.9 to 1.3)	0.7 (0.5 to 0.9)	**-30.4 (-55.69 to -8.33)**	0.4 (0.3 to 0.6)	0.4 (0.2 to 0.6)	3.8 (-42.55 to 50.67)	1.5 (1.1 to 2.1)	1.1 (0.6 to 1.4)	-26.5 (-58.75 to 7.24)	0.3 (0.2 to 0.4)	0.2 (0.1 to 0.3)	**-23.5 (-57.05 to 1.39)**	0.7 (0.6 to 0.9)	0.4 (0.3 to 0.5)	**-41.4 (-65.45 to -22.54)**
Liver cancer due to other causes	0.1 (0.1 to 0.2)	0.1 (0.1 to 0.2)	8.8 (-33.42 to 51.10)	0.2 (0.2 to 0.2)	0.2 (0.1 to 0.2)	-17.2 (-47.87 to 13.91)	<0.05 (0.0 to 0.1)	<0.05 (0.0 to <0.05)	-28.2 (-62.64 to 16.11)	0.2 (0.1 to 0.2)	0.1 (0.0 to 0.1)	**-46.9 (-72.09 to -14.17)**	0.1 (0.1 to 0.1)	0.1 (0.1 to 0.1)	31.8 (-30.34 to 102.10)	0.2 (0.2 to 0.3)	0.2 (0.1 to 0.3)	3.8 (-41.35 to 57.54)
Larynx cancer	0.8 (0.6 to 1.0)	0.7 (0.6 to 0.9)	**-14.3 (-26.91 to -2.07)**	1.2 (1.0 to 1.5)	0.8 (0.6 to 1.0)	**-34.2 (-43.45 to -25.65)**	0.7 (0.5 to 0.9)	0.6 (0.4 to 0.7)	**-16.9 (-31.44 to -2.91)**	2.4 (1.7 to 3.0)	1.4 (1.0 to 1.9)	**-40.3 (-50.37 to -30.78)**	0.2 (0.1 to 0.2)	0.1 (0.1 to 0.2)	-3.0 (-22.08 to 19.79)	0.4 (0.3 to 0.6)	0.3 (0.2 to 0.5)	**-20.1 (-36.56 to -1.29)**
Lung, bronchus, and trachea cancer	33.8 (33.0 to 35.9)	30.3 (27.5 to 32.7)	**-10.2 (-19.74 to -2.07)**	49.2 (48.0 to 51.9)	34.2 (31.3 to 37.0)	**-30.3 (-37.00 to -24.13)**	23.1 (22.5 to 23.5)	17.4 (15.6 to 19.2)	**-24.5 (-33.04 to -16.17)**	80.7 (78.8 to 82.2)	43.6 (39.0 to 48.1)	**-46.0 (-52.26 to -40.04)**	10.7 (10.2 to 13.0)	12.9 (10.0 to 14.4)	21.4 (-10.25 to 38.84)	27.3 (26.1 to 32.3)	26.8 (21.4 to 29.8)	-1.4 (-23.45 to 11.75)
Breast cancer	13.8 (11.4 to 16.3)	11.2 (8.8 to 13.3)	**-18.8 (-26.36 to -9.46)**	21.4 (17.7 to 25.2)	13.2 (10.4 to 15.8)	**-38.2 (-43.64 to -30.72)**	0.1 (0.0 to 0.2)	0.1 (0.0 to 0.2)	-4.6 (-28.32 to 34.82)	0.3 (0.1 to 0.6)	0.2 (0.1 to 0.4)	**-33.1 (-49.57 to -6.00)**	13.7 (11.4 to 16.2)	11.1 (8.7 to 13.2)	**-18.9 (-26.44 to -9.51)**	38.2 (31.7 to 45.0)	24.5 (19.2 to 29.2)	**-36.0 (-41.62 to -28.15)**
Cervical cancer	1.6 (1.3 to 1.7)	1.0 (0.8 to 1.2)	**-37.0 (-45.29 to -27.66)**	2.6 (2.0 to 2.8)	1.3 (1.1 to 1.5)	**-50.2 (-56.88 to -43.31)**	--	--	--	--	--	--	1.6 (1.3 to 1.7)	1.0 (0.8 to 1.2)	**-37.0 (-45.29 to -27.66)**	4.7 (3.7 to 5.1)	2.4 (2.0 to 2.8)	**-48.7 (-55.67 to -41.70)**
Uterine cancer	1.0 (0.9 to 1.2)	1.5 (1.1 to 1.7)	**56.0 (10.52 to 79.44)**	1.4 (1.3 to 1.7)	1.7 (1.3 to 1.9)	20.6 (-14.66 to 39.25)	--	--	--	--	--	--	1.0 (0.9 to 1.2)	1.5 (1.1 to 1.7)	**56.0 (10.52 to 79.44)**	2.4 (2.2 to 3.0)	3.0 (2.3 to 3.5)	28.0 (-9.91 to 48.07)
Prostate cancer	7.6 (5.8 to 10.2)	9.8 (6.5 to 13.9)	29.2 (-15.88 to 55.78)	10.1 (7.8 to 13.6)	9.9 (6.6 to 13.9)	-1.5 (-36.75 to 17.68)	7.6 (5.8 to 10.2)	9.8 (6.5 to 13.9)	29.2 (-15.88 to 55.78)	27.9 (21.3 to 37.5)	23.3 (15.5 to 32.8)	**-16.2 (-44.83 to 0.52)**	--	--	--	--	--	--
Colon and rectum cancer	17.9 (17.4 to 18.7)	16.7 (15.6 to 17.7)	**-6.9 (-13.70 to -1.12)**	25.5 (24.7 to 26.5)	18.1 (16.9 to 19.1)	**-28.9 (-34.17 to -24.36)**	8.7 (8.4 to 8.9)	9.0 (8.3 to 9.7)	4.0 (-3.84 to 11.49)	31.0 (30.2 to 31.7)	22.5 (20.7 to 24.1)	**-27.5 (-33.14 to -22.26)**	9.3 (8.8 to 10.1)	7.7 (6.9 to 8.4)	**-17.1 (-26.09 to -8.68)**	21.7 (20.7 to 23.6)	14.4 (13.1 to 15.6)	**-33.6 (-40.73 to -27.50)**
Mouth cancer	1.2 (1.1 to 1.2)	1.3 (1.1 to 1.4)	12.1 (-10.97 to 22.95)	1.8 (1.7 to 1.9)	1.6 (1.3 to 1.7)	**-11.6 (-31.27 to -3.20)**	0.8 (0.7 to 0.8)	0.8 (0.6 to 0.9)	9.9 (-26.19 to 22.95)	2.7 (2.6 to 2.9)	2.2 (1.6 to 2.5)	**-19.0 (-45.10 to -9.58)**	0.4 (0.4 to 0.5)	0.5 (0.4 to 0.6)	**16.4 (0.72 to 31.31)**	1.0 (0.9 to 1.2)	1.0 (0.8 to 1.2)	-4.5 (-17.74 to 7.26)
Nasopharynx cancer	0.2 (0.2 to 0.3)	0.1 (0.1 to 0.2)	**-38.3 (-45.89 to -22.86)**	0.4 (0.3 to 0.5)	0.2 (0.1 to 0.3)	**-50.9 (-56.66 to -39.18)**	0.1 (0.1 to 0.2)	0.1 (0.1 to 0.1)	**-36.7 (-45.41 to -18.88)**	0.5 (0.4 to 0.6)	0.2 (0.2 to 0.3)	**-51.2 (-57.74 to -37.92)**	0.1 (0.0 to 0.1)	<0.05 (0.0 to 0.1)	**-40.9 (-52.05 to -18.33)**	0.2 (0.1 to 0.4)	0.1 (0.1 to 0.2)	**-51.3 (-59.72 to -35.25)**
Other pharynx cancer	0.5 (0.4 to 0.6)	0.6 (0.4 to 0.7)	12.8 (-20.77 to 34.73)	0.8 (0.7 to 0.9)	0.7 (0.5 to 0.8)	-10.5 (-36.64 to 6.66)	0.3 (0.3 to 0.4)	0.4 (0.3 to 0.5)	17.5 (-28.08 to 48.39)	1.2 (1.1 to 1.5)	1.1 (0.7 to 1.4)	-11.7 (-45.14 to 10.85)	0.2 (0.1 to 0.2)	0.2 (0.1 to 0.2)	2.9 (-13.34 to 19.15)	0.4 (0.3 to 0.5)	0.4 (0.3 to 0.4)	**-14.3 (-27.21 to -0.64)**
Gallbladder and biliary tract cancer	1.3 (1.0 to 1.5)	1.0 (0.8 to 1.3)	**-19.2 (-31.83 to 0.34)**	1.8 (1.3 to 2.1)	1.1 (0.8 to 1.4)	**-38.3 (-47.69 to -23.40)**	0.4 (0.3 to 0.4)	0.3 (0.2 to 0.4)	**-20.0 (-33.79 to 4.52)**	1.4 (1.0 to 1.6)	0.8 (0.6 to 1.0)	**-44.4 (-53.94 to -26.91)**	0.9 (0.6 to 1.1)	0.7 (0.5 to 1.0)	**-18.7 (-33.78 to 1.03)**	2.1 (1.5 to 2.6)	1.4 (0.9 to 1.8)	**-33.5 (-45.10 to -17.33)**
Pancreatic cancer	6.4 (6.2 to 6.7)	7.7 (7.2 to 8.1)	**20.3 (11.26 to 28.08)**	9.2 (9.0 to 9.6)	8.6 (8.0 to 9.1)	**-6.4 (-13.58 to -0.32)**	3.1 (3.0 to 3.1)	3.8 (3.5 to 4.1)	**22.4 (12.90 to 31.57)**	10.9 (10.6 to 11.1)	9.5 (8.8 to 10.3)	**-12.6 (-19.44 to -5.88)**	3.3 (3.2 to 3.6)	3.9 (3.6 to 4.3)	**18.3 (5.55 to 29.39)**	7.9 (7.6 to 8.6)	7.7 (7.0 to 8.4)	-2.1 (-12.82 to 6.97)
Malignant skin melanoma	1.4 (1.0 to 2.1)	1.9 (1.3 to 2.7)	**35.0 (-3.60 to 53.75)**	2.3 (1.6 to 3.3)	2.4 (1.6 to 3.3)	4.9 (-25.47 to 18.80)	0.7 (0.4 to 1.2)	1.0 (0.5 to 1.7)	**60.8 (-26.59 to 93.79)**	2.4 (1.3 to 4.2)	2.8 (1.3 to 4.6)	18.4 (-44.88 to 41.89)	0.8 (0.5 to 1.0)	0.9 (0.6 to 1.1)	13.7 (-5.03 to 28.53)	2.1 (1.5 to 2.9)	1.9 (1.3 to 2.5)	-8.9 (-23.29 to 2.73)
Non-melanoma skin cancer	0.5 (0.4 to 0.6)	0.6 (0.5 to 0.8)	**31.6 (18.42 to 44.51)**	0.6 (0.5 to 0.8)	0.6 (0.5 to 0.8)	-4.0 (-12.48 to 4.71)	0.2 (0.2 to 0.4)	0.3 (0.2 to 0.5)	**35.6 (22.89 to 51.31)**	0.9 (0.6 to 1.4)	0.8 (0.5 to 1.2)	**-12.2 (-20.17 to -3.03)**	0.2 (0.2 to 0.3)	0.3 (0.2 to 0.4)	**27.4 (2.87 to 48.93)**	0.5 (0.4 to 0.6)	0.5 (0.3 to 0.6)	0.3 (-16.28 to 15.36)
Ovarian cancer	3.9 (3.7 to 4.4)	4.1 (3.6 to 4.5)	4.7 (-9.08 to 15.64)	6.0 (5.7 to 6.6)	4.8 (4.2 to 5.3)	**-20.2 (-29.76 to -11.94)**	--	--	--	--	--	--	3.9 (3.7 to 4.4)	4.1 (3.6 to 4.5)	4.7 (-9.08 to 15.64)	10.9 (10.3 to 12.0)	8.9 (7.9 to 9.9)	**-17.6 (-27.18 to -9.12)**
Testicular cancer	0.1 (0.1 to 0.1)	0.1 (0.1 to 0.1)	**-32.8 (-45.24 to -8.94)**	0.2 (0.2 to 0.3)	0.1 (0.1 to 0.2)	**-41.5 (-52.31 to -21.34)**	0.1 (0.1 to 0.1)	0.1 (0.1 to 0.1)	**-32.8 (-45.24 to -8.94)**	0.5 (0.3 to 0.6)	0.3 (0.2 to 0.3)	**-42.6 (-53.21 to -22.52)**	--	--	--	--	--	--
Kidney cancer	2.7 (2.5 to 3.0)	3.4 (3.0 to 3.8)	**28.2 (12.75 to 40.97)**	4.0 (3.7 to 4.5)	3.9 (3.5 to 4.3)	-2.0 (-12.77 to 6.53)	1.6 (1.5 to 1.6)	2.1 (1.9 to 2.4)	**35.2 (19.54 to 50.02)**	5.6 (5.4 to 5.8)	5.5 (4.9 to 6.0)	-3.2 (-14.65 to 7.61)	1.1 (0.9 to 1.4)	1.3 (1.0 to 1.6)	18.8 (-10.14 to 51.92)	2.8 (2.3 to 3.5)	2.6 (2.0 to 3.2)	-4.4 (-23.55 to 16.91)
Bladder cancer	4.7 (4.0 to 5.4)	3.9 (3.3 to 4.5)	**-17.3 (-23.26 to -10.08)**	6.5 (5.5 to 7.4)	4.0 (3.4 to 4.6)	**-38.4 (-42.64 to -32.93)**	3.2 (2.6 to 3.4)	2.6 (2.1 to 3.0)	**-19.3 (-27.79 to -9.19)**	11.5 (9.3 to 12.2)	6.2 (5.1 to 7.1)	**-45.9 (-51.48 to -39.27)**	1.5 (1.1 to 2.1)	1.3 (0.9 to 1.8)	**-12.9 (-24.27 to 1.41)**	3.3 (2.3 to 4.6)	2.3 (1.6 to 3.3)	**-30.5 (-39.55 to -17.85)**
Brain and nervous system cancer	2.6 (2.3 to 3.1)	3.1 (2.6 to 3.5)	**18.8 (6.06 to 29.70)**	4.4 (3.8 to 5.2)	4.2 (3.5 to 4.8)	-6.3 (-14.68 to 1.11)	1.5 (1.2 to 1.9)	1.8 (1.4 to 2.2)	**22.9 (8.00 to 35.49)**	5.5 (4.3 to 6.8)	5.2 (4.0 to 6.3)	-5.8 (-16.58 to 3.27)	1.1 (1.0 to 1.4)	1.3 (0.9 to 1.5)	13.5 (-6.22 to 26.64)	3.5 (2.9 to 4.4)	3.2 (2.5 to 3.7)	**-8.9 (-19.90 to 0.59)**
Thyroid cancer	0.4 (0.3 to 0.4)	0.3 (0.3 to 0.4)	-12.6 (-25.59 to 5.33)	0.5 (0.4 to 0.6)	0.3 (0.3 to 0.4)	**-32.2 (-42.48 to -18.42)**	0.1 (0.1 to 0.1)	0.1 (0.1 to 0.1)	**20.7 (-13.96 to 38.57)**	0.4 (0.3 to 0.4)	0.3 (0.3 to 0.4)	**-13.7 (-38.55 to -0.95)**	0.3 (0.2 to 0.3)	0.2 (0.1 to 0.2)	**-25.7 (-39.10 to -3.11)**	0.6 (0.4 to 0.7)	0.4 (0.3 to 0.5)	**-38.7 (-50.27 to -19.24)**
Mesothelioma	0.6 (0.5 to 1.0)	1.9 (1.0 to 2.3)	**272.0 (32.48 to 375.47)**	0.8 (0.7 to 1.5)	2.2 (1.1 to 2.6)	**180.0 (5.45 to 254.27)**	0.4 (0.3 to 0.8)	1.6 (0.7 to 1.9)	**361.2 (26.61 to 506.90)**	1.3 (1.1 to 2.9)	4.0 (1.7 to 4.9)	**230.3 (-4.95 to 333.04)**	0.2 (0.1 to 0.3)	0.3 (0.2 to 0.4)	**90.9 (4.79 to 131.13)**	0.4 (0.4 to 0.7)	0.7 (0.5 to 0.9)	53.1 (-12.19 to 84.64)
Hodgkin disease	0.4 (0.3 to 0.5)	0.3 (0.2 to 0.4)	**-24.7 (-36.24 to -5.82)**	0.7 (0.5 to 0.8)	0.4 (0.3 to 0.5)	**-37.9 (-47.33 to -22.73)**	0.2 (0.1 to 0.3)	0.2 (0.1 to 0.2)	**-28.3 (-40.60 to -2.68)**	0.8 (0.5 to 1.1)	0.5 (0.3 to 0.7)	**-42.5 (-52.23 to -23.25)**	0.2 (0.1 to 0.2)	0.1 (0.1 to 0.2)	**-19.6 (-35.65 to -0.91)**	0.5 (0.4 to 0.7)	0.3 (0.2 to 0.4)	**-31.7 (-44.59 to -15.33)**
Non-Hodgkin lymphoma	3.5 (2.9 to 4.9)	4.7 (3.2 to 5.6)	37.1 (-23.12 to 59.11)	5.2 (4.4 to 7.4)	5.5 (3.6 to 6.6)	5.0 (-40.56 to 21.02)	1.8 (1.5 to 3.0)	2.6 (1.3 to 3.2)	45.2 (-33.43 to 70.52)	6.6 (5.3 to 10.8)	6.8 (3.3 to 8.2)	3.8 (-52.03 to 21.06)	1.7 (1.2 to 2.5)	2.1 (1.2 to 3.0)	29.1 (-24.38 to 54.29)	4.2 (3.1 to 6.3)	4.3 (2.5 to 6.0)	4.3 (-38.14 to 23.30)
Multiple myeloma	2.1 (1.6 to 2.9)	2.8 (2.1 to 3.8)	**34.1 (17.14 to 47.81)**	3.0 (2.2 to 4.2)	3.1 (2.4 to 4.2)	4.1 (-9.66 to 14.60)	1.0 (0.7 to 1.6)	1.5 (1.0 to 2.3)	**46.2 (8.93 to 70.67)**	3.7 (2.4 to 5.8)	3.8 (2.5 to 5.7)	2.5 (-23.14 to 20.22)	1.1 (0.7 to 1.6)	1.3 (0.9 to 2.0)	**22.8 (6.13 to 40.12)**	2.5 (1.7 to 3.8)	2.6 (1.8 to 3.8)	2.6 (-10.05 to 15.78)
Leukemia	3.5 (3.4 to 3.7)	4.1 (3.6 to 4.4)	**15.6 (2.23 to 25.65)**	5.4 (5.3 to 5.7)	4.8 (4.2 to 5.2)	**-12.3 (-22.80 to -4.62)**	1.9 (1.8 to 1.9)	2.4 (2.0 to 2.6)	**26.7 (7.24 to 39.11)**	7.0 (6.8 to 7.2)	6.2 (5.3 to 6.8)	**-10.9 (-24.62 to -2.14)**	1.6 (1.5 to 1.8)	1.7 (1.4 to 1.9)	3.0 (-21.30 to 18.06)	4.3 (4.1 to 4.7)	3.5 (2.9 to 4.0)	**-18.6 (-38.43 to -6.52)**
Other neoplasms	4.0 (3.6 to 4.7)	5.4 (4.3 to 5.9)	**35.6 (6.19 to 53.43)**	6.1 (5.6 to 7.2)	6.3 (5.0 to 6.9)	3.3 (-17.87 to 15.71)	1.7 (1.5 to 2.3)	2.4 (1.7 to 2.7)	**38.6 (-3.64 to 58.92)**	6.5 (5.7 to 8.5)	6.4 (4.6 to 7.2)	-1.1 (-31.00 to 12.82)	2.2 (1.9 to 2.8)	3.0 (2.2 to 3.4)	**33.6 (2.82 to 55.65)**	5.7 (5.0 to 6.9)	6.1 (4.8 to 7.0)	7.6 (-13.42 to 23.58)
**Cardiovascular diseases**	240.1 (225.0 to 245.5)	157.8 (149.1 to 172.3)	**-34.3 (-37.83 to -28.25)**	328.7 (308.3 to 335.9)	156.5 (147.9 to 170.2)	**-52.4 (-54.91 to -48.18)**	116.1 (113.5 to 118.1)	73.1 (67.8 to 77.8)	**-37.0 (-41.55 to -33.08)**	419.4 (409.9 to 426.9)	177.3 (164.4 to 188.7)	**-57.7 (-60.82 to -55.05)**	124.0 (110.0 to 128.6)	84.7 (77.8 to 100.1)	**-31.6 (-37.58 to -18.65)**	258.6 (228.3 to 269.1)	136.8 (125.8 to 163.2)	**-47.0 (-51.78 to -36.64)**
Rheumatic heart disease	2.1 (1.4 to 2.6)	1.2 (1.0 to 2.0)	**-42.1 (-52.47 to 9.61)**	3.0 (2.0 to 3.7)	1.3 (1.1 to 2.1)	**-57.2 (-64.64 to -20.03)**	0.5 (0.4 to 0.8)	0.3 (0.2 to 0.5)	**-41.2 (-52.73 to -17.65)**	2.0 (1.3 to 2.7)	0.8 (0.6 to 1.2)	**-58.8 (-66.52 to -43.19)**	1.6 (1.0 to 1.9)	0.9 (0.7 to 1.6)	-41.8 (-55.01 to 28.91)	3.7 (2.3 to 4.6)	1.6 (1.3 to 3.0)	**-55.2 (-65.35 to 0.68)**
Ischemic heart disease	147.6 (132.8 to 154.3)	80.7 (74.2 to 94.1)	**-45.3 (-48.92 to -36.19)**	204.5 (184.5 to 213.4)	81.5 (75.2 to 94.8)	**-60.1 (-62.72 to -53.84)**	79.5 (73.0 to 83.2)	42.0 (37.9 to 46.7)	**-47.2 (-51.47 to -40.53)**	285.4 (262.3 to 298.7)	102.6 (92.7 to 114.0)	**-64.0 (-66.90 to -59.57)**	68.1 (56.1 to 73.6)	38.7 (33.8 to 51.2)	**-43.0 (-49.38 to -22.91)**	143.4 (118.1 to 155.4)	63.1 (55.2 to 83.9)	**-55.9 (-60.87 to -39.60)**
Cerebrovascular disease	62.4 (53.5 to 68.2)	46.6 (41.3 to 55.1)	**-25.2 (-31.48 to -12.80)**	82.8 (71.3 to 90.9)	44.5 (39.5 to 52.4)	**-46.2 (-50.65 to -37.93)**	22.9 (19.5 to 27.5)	18.1 (13.9 to 21.6)	**-20.8 (-38.77 to -8.17)**	84.5 (71.6 to 101.4)	42.9 (32.8 to 51.3)	**-49.2 (-60.97 to -41.16)**	39.5 (30.5 to 43.7)	28.5 (24.7 to 37.2)	**-27.5 (-35.81 to -6.43)**	80.2 (62.3 to 89.5)	45.0 (39.1 to 58.7)	**-43.7 (-49.97 to -27.51)**
Ischemic stroke	39.9 (32.7 to 43.8)	28.0 (24.1 to 34.9)	**-29.7 (-38.60 to -8.53)**	51.8 (42.3 to 56.8)	25.8 (22.2 to 32.1)	**-49.9 (-56.25 to -34.82)**	14.4 (11.6 to 17.3)	10.7 (7.9 to 13.8)	**-25.3 (-42.64 to -2.46)**	53.4 (43.1 to 64.2)	24.9 (18.2 to 32.0)	**-53.2 (-63.88 to -39.24)**	25.6 (18.7 to 28.3)	17.3 (14.6 to 23.3)	**-31.8 (-41.75 to -0.92)**	49.6 (36.3 to 54.8)	26.1 (22.1 to 35.2)	**-47.0 (-54.63 to -22.51)**
Hemorrhagic stroke	22.4 (19.4 to 26.9)	18.6 (13.7 to 22.0)	**-16.8 (-35.60 to -5.26)**	31.1 (27.1 to 37.4)	18.7 (13.9 to 22.1)	**-39.8 (-52.86 to -31.97)**	8.5 (6.9 to 11.2)	7.4 (4.5 to 9.8)	-12.2 (-49.44 to 13.27)	31.1 (25.1 to 40.7)	18.0 (10.9 to 23.6)	**-41.5 (-65.65 to -25.16)**	13.9 (10.7 to 17.3)	11.2 (8.1 to 14.7)	**-19.2 (-36.87 to 0.45)**	30.6 (23.7 to 38.1)	18.9 (13.8 to 24.5)	**-38.1 (-51.14 to -24.10)**
Hypertensive heart disease	2.5 (2.1 to 3.2)	2.5 (1.8 to 3.1)	2.0 (-28.42 to 21.55)	3.5 (2.9 to 4.5)	2.6 (1.9 to 3.2)	**-24.7 (-46.81 to -9.70)**	1.1 (0.9 to 1.6)	1.1 (0.6 to 1.5)	-2.5 (-42.12 to 33.09)	4.0 (3.1 to 5.7)	2.7 (1.6 to 3.9)	**-32.5 (-59.59 to -7.87)**	1.4 (1.1 to 1.9)	1.5 (0.9 to 1.9)	6.2 (-25.71 to 37.58)	3.0 (2.4 to 4.2)	2.5 (1.6 to 3.2)	-17.3 (-40.86 to 7.82)
Cardiomyopathy and myocarditis	5.2 (4.3 to 6.0)	2.8 (2.3 to 3.7)	**-44.7 (-52.86 to -22.03)**	7.3 (6.0 to 8.5)	3.0 (2.4 to 3.9)	**-58.9 (-65.18 to -44.42)**	2.0 (1.4 to 2.4)	1.3 (0.9 to 1.9)	**-33.6 (-47.52 to -3.21)**	7.4 (5.4 to 9.2)	3.4 (2.4 to 5.0)	**-54.5 (-64.30 to -35.07)**	3.2 (2.5 to 3.9)	1.6 (1.1 to 2.1)	**-51.2 (-63.26 to -30.69)**	6.6 (5.1 to 8.1)	2.5 (1.8 to 3.4)	**-61.5 (-72.24 to -44.48)**
Atrial fibrillation and flutter	1.5 (1.3 to 1.7)	4.2 (3.5 to 4.8)	**183.6 (135.86 to 221.91)**	1.9 (1.7 to 2.2)	3.8 (3.1 to 4.4)	**100.0 (66.79 to 125.68)**	0.4 (0.4 to 0.5)	1.3 (0.9 to 1.5)	**183.0 (118.23 to 228.23)**	1.7 (1.4 to 1.9)	2.9 (2.2 to 3.4)	**73.6 (33.43 to 100.79)**	1.0 (0.9 to 1.3)	3.0 (2.3 to 3.5)	**184.0 (132.09 to 234.64)**	2.0 (1.7 to 2.4)	4.4 (3.5 to 5.2)	**119.7 (81.83 to 156.27)**
Aortic aneurysm	8.1 (6.9 to 9.9)	7.0 (5.7 to 8.8)	**-14.4 (-24.67 to -4.64)**	11.0 (9.4 to 13.3)	7.0 (5.8 to 8.8)	**-36.4 (-43.78 to -29.43)**	5.1 (4.4 to 6.1)	3.9 (3.2 to 4.4)	**-24.3 (-36.36 to -15.86)**	18.1 (15.6 to 21.4)	9.3 (7.7 to 10.6)	**-48.5 (-56.54 to -42.67)**	3.0 (1.9 to 4.5)	3.1 (2.0 to 4.8)	2.9 (-16.63 to 22.35)	6.3 (4.0 to 9.3)	5.1 (3.3 to 8.0)	**-17.8 (-33.05 to -2.82)**
Peripheral vascular disease	2.3 (2.1 to 2.5)	2.1 (1.8 to 2.4)	-7.1 (-18.78 to 3.37)	3.0 (2.7 to 3.3)	2.0 (1.7 to 2.3)	**-33.2 (-41.28 to -25.51)**	1.0 (0.9 to 1.1)	0.8 (0.7 to 0.9)	**-22.3 (-36.04 to -8.92)**	3.8 (3.4 to 4.2)	1.9 (1.6 to 2.2)	**-50.8 (-59.40 to -42.57)**	1.2 (1.1 to 1.5)	1.3 (1.0 to 1.6)	5.6 (-11.31 to 23.07)	2.4 (2.1 to 2.9)	2.0 (1.6 to 2.4)	**-17.1 (-29.47 to -4.27)**
Endocarditis	0.6 (0.4 to 0.7)	0.6 (0.5 to 0.9)	7.4 (-9.05 to 45.15)	0.8 (0.6 to 1.1)	0.7 (0.5 to 1.0)	-17.6 (-29.78 to 9.65)	0.3 (0.2 to 0.4)	0.3 (0.2 to 0.5)	**38.6 (-0.06 to 102.46)**	0.9 (0.6 to 1.3)	0.9 (0.6 to 1.4)	-3.5 (-29.45 to 37.68)	0.3 (0.2 to 0.5)	0.3 (0.2 to 0.4)	-16.1 (-32.72 to 20.12)	0.8 (0.5 to 1.1)	0.5 (0.4 to 0.8)	**-32.0 (-45.78 to 0.08)**
Other cardiovascular and circulatory diseases	7.9 (7.1 to 11.1)	10.0 (6.4 to 11.4)	27.6 (-32.46 to 44.57)	10.9 (9.8 to 15.2)	10.1 (6.4 to 11.4)	-5.8 (-50.17 to 6.38)	3.2 (2.9 to 4.9)	4.1 (2.2 to 4.7)	28.9 (-37.47 to 48.47)	11.6 (10.5 to 17.8)	10.0 (5.4 to 11.4)	**-12.9 (-57.35 to -0.17)**	4.7 (4.0 to 6.7)	5.9 (3.6 to 7.0)	26.9 (-30.70 to 48.58)	10.2 (8.6 to 14.5)	10.1 (6.2 to 12.0)	0.1 (-45.92 to 16.52)
**Chronic respiratory diseases**	31.6 (28.1 to 34.2)	34.2 (30.7 to 39.4)	8.5 (-2.84 to 26.90)	43.1 (38.5 to 46.6)	34.6 (31.1 to 39.6)	**-19.7 (-27.99 to -6.70)**	19.5 (15.6 to 21.0)	18.7 (16.4 to 24.5)	-3.6 (-16.46 to 29.96)	70.9 (56.4 to 76.0)	44.7 (39.0 to 58.1)	**-36.7 (-45.00 to -14.97)**	12.1 (11.3 to 14.5)	15.5 (11.8 to 17.2)	**28.8 (-9.76 to 42.74)**	27.3 (25.6 to 32.5)	27.3 (21.1 to 30.3)	0.2 (-29.22 to 10.70)
Chronic obstructive pulmonary disease	26.3 (23.2 to 28.2)	28.7 (25.7 to 34.1)	9.0 (-2.15 to 32.64)	35.7 (31.5 to 38.3)	28.7 (25.7 to 34.0)	**-19.3 (-27.43 to -2.76)**	16.7 (13.1 to 17.9)	15.7 (13.5 to 20.9)	-5.7 (-19.87 to 32.22)	60.4 (47.3 to 64.6)	37.2 (32.0 to 49.7)	**-38.1 (-47.37 to -13.59)**	9.7 (8.6 to 11.5)	13.0 (10.5 to 15.0)	**35.0 (-1.87 to 54.81)**	21.6 (19.2 to 25.8)	22.6 (18.3 to 26.1)	5.0 (-23.99 to 19.67)
Pneumoconiosis	0.5 (0.4 to 0.7)	0.7 (0.4 to 1.0)	**29.0 (-5.56 to 89.82)**	0.7 (0.5 to 0.9)	0.6 (0.4 to 1.0)	-5.7 (-30.52 to 36.80)	0.5 (0.3 to 0.7)	0.6 (0.4 to 1.0)	**33.2 (-4.14 to 99.07)**	1.8 (1.2 to 2.5)	1.5 (1.0 to 2.3)	-15.2 (-38.68 to 26.85)	<0.05 (0.0 to <0.05)	<0.05 (0.0 to <0.05)	**-32.8 (-54.53 to -11.03)**	0.1 (0.1 to 0.1)	<0.05 (0.0 to 0.1)	**-44.0 (-60.05 to -29.90)**
Silicosis	<0.05 (0.0 to <0.05)	<0.05 (0.0 to <0.05)	**-53.7 (-67.96 to -25.42)**	<0.05 (0.0 to 0.1)	<0.05 (0.0 to <0.05)	**-63.5 (-73.73 to -44.26)**	<0.05 (0.0 to <0.05)	<0.05 (0.0 to <0.05)	**-54.5 (-69.97 to -24.21)**	0.1 (0.1 to 0.2)	<0.05 (0.0 to 0.1)	**-69.1 (-78.99 to -49.66)**	<0.05 (0.0 to <0.05)	<0.05 (0.0 to <0.05)	**-35.9 (-53.67 to -13.94)**	<0.05 (0.0 to <0.05)	<0.05 (0.0 to <0.05)	**-31.5 (-47.07 to -15.86)**
Asbestosis	0.1 (0.1 to 0.1)	0.3 (0.2 to 0.5)	**188.4 (111.72 to 350.72)**	0.1 (0.1 to 0.2)	0.3 (0.2 to 0.5)	**112.9 (58.44 to 230.30)**	0.1 (0.1 to 0.1)	0.3 (0.2 to 0.5)	**191.0 (112.32 to 356.07)**	0.4 (0.2 to 0.5)	0.7 (0.4 to 1.1)	**89.1 (39.38 to 196.64)**	<0.05 (0.0 to <0.05)	<0.05 (0.0 to <0.05)	**73.7 (0.76 to 147.80)**	<0.05 (0.0 to <0.05)	<0.05 (0.0 to <0.05)	35.3 (-13.49 to 77.60)
Coal workers pneumoconiosis	0.2 (0.1 to 0.3)	0.2 (0.2 to 0.4)	20.3 (-13.70 to 75.21)	0.3 (0.2 to 0.4)	0.2 (0.1 to 0.3)	-13.1 (-37.65 to 24.78)	0.2 (0.1 to 0.3)	0.2 (0.2 to 0.4)	21.5 (-13.00 to 77.76)	0.7 (0.5 to 1.0)	0.6 (0.4 to 0.8)	-23.4 (-45.02 to 12.17)	<0.05 (0.0 to <0.05)	<0.05 (0.0 to <0.05)	**-65.7 (-76.25 to -53.92)**	<0.05 (0.0 to <0.05)	<0.05 (0.0 to <0.05)	**-57.1 (-69.25 to -45.39)**

Other pneumoconiosis	0.2 (0.1 to 0.2)	0.1 (0.1 to 0.2)	**-36.2 (-53.87 to -14.55)**	0.2 (0.2 to 0.3)	0.1 (0.1 to 0.2)	**-54.0 (-66.57 to -39.76)**	0.2 (0.1 to 0.2)	0.1 (0.1 to 0.2)	**-35.8 (-55.48 to -10.41)**	0.6 (0.4 to 0.8)	0.2 (0.1 to 0.4)	**-59.9 (-72.20 to -44.77)**	<0.05 (0.0 to <0.05)	<0.05 (0.0 to <0.05)	**-37.9 (-58.36 to -17.71)**	0.1 (0.0 to 0.1)	<0.05 (0.0 to <0.05)	**-51.5 (-66.25 to -37.64)**
Asthma	1.6 (1.2 to 1.9)	1.3 (0.9 to 1.9)	-15.2 (-33.53 to 20.05)	2.5 (1.9 to 3.0)	1.5 (1.1 to 2.1)	**-39.5 (-50.74 to -15.70)**	0.7 (0.5 to 0.7)	0.3 (0.3 to 0.4)	**-48.2 (-55.77 to -33.91)**	2.5 (2.0 to 2.8)	1.0 (0.8 to 1.2)	**-61.8 (-67.23 to -52.44)**	0.9 (0.6 to 1.3)	1.0 (0.6 to 1.5)	9.8 (-23.38 to 79.95)	2.5 (1.6 to 3.4)	1.9 (1.2 to 2.9)	**-22.2 (-41.75 to 26.64)**
Interstitial lung disease and pulmonary sarcoidosis	1.3 (0.9 to 2.1)	3.4 (1.5 to 5.1)	**200.4 (1.36 to 390.44)**	1.8 (1.2 to 2.9)	3.5 (1.6 to 5.2)	123.5 (-23.70 to 262.36)	0.9 (0.6 to 1.5)	2.0 (0.8 to 2.8)	164.6 (-11.43 to 334.65)	3.1 (2.3 to 5.3)	4.8 (2.0 to 6.8)	78.2 (-39.61 to 192.71)	0.4 (0.2 to 0.9)	1.4 (0.4 to 2.5)	**291.4 (5.44 to 565.07)**	1.0 (0.6 to 2.1)	2.5 (0.8 to 4.4)	198.4 (-14.54 to 400.46)
Other chronic respiratory diseases	1.9 (1.5 to 2.3)	0.2 (0.2 to 0.3)	**-89.1 (-91.15 to -86.85)**	2.5 (2.0 to 3.1)	0.2 (0.2 to 0.3)	**-91.6 (-93.06 to -89.99)**	0.8 (0.7 to 0.9)	0.1 (0.1 to 0.1)	**-87.3 (-89.76 to -82.55)**	3.1 (2.5 to 3.6)	0.3 (0.2 to 0.3)	**-91.7 (-93.25 to -88.80)**	1.0 (0.7 to 1.5)	0.1 (0.1 to 0.1)	**-90.6 (-93.51 to -88.38)**	2.1 (1.5 to 3.0)	0.2 (0.1 to 0.2)	**-92.0 (-94.36 to -90.34)**
**Cirrhosis**	3.9 (3.8 to 4.0)	6.7 (6.2 to 7.1)	**72.7 (60.46 to 83.56)**	6.3 (6.1 to 6.5)	8.9 (8.3 to 9.5)	**41.7 (31.71 to 51.08)**	2.0 (2.0 to 2.1)	4.1 (3.7 to 4.4)	**101.5 (84.95 to 117.38)**	7.5 (7.3 to 7.6)	11.6 (10.6 to 12.5)	**55.2 (42.17 to 67.67)**	1.9 (1.8 to 2.0)	2.6 (2.4 to 2.8)	**41.3 (27.02 to 54.53)**	5.2 (5.0 to 5.6)	6.4 (5.7 to 6.9)	**21.7 (9.69 to 33.27)**
Cirrhosis due to hepatitis B	0.4 (0.4 to 0.5)	0.7 (0.6 to 0.9)	**75.3 (35.01 to 114.75)**	0.7 (0.6 to 0.8)	0.9 (0.8 to 1.1)	**42.1 (9.32 to 74.01)**	0.2 (0.2 to 0.3)	0.4 (0.3 to 0.5)	**69.4 (27.79 to 112.58)**	0.8 (0.6 to 1.0)	1.0 (0.8 to 1.3)	**30.3 (-1.10 to 63.51)**	0.2 (0.2 to 0.3)	0.4 (0.3 to 0.5)	**83.8 (24.58 to 153.96)**	0.6 (0.4 to 0.7)	0.9 (0.6 to 1.1)	**55.5 (3.18 to 117.40)**
Cirrhosis due to hepatitis C	1.4 (1.3 to 1.6)	3.1 (2.8 to 3.5)	**121.0 (90.81 to 151.89)**	2.3 (2.1 to 2.6)	4.2 (3.7 to 4.6)	**79.5 (54.56 to 105.41)**	0.7 (0.6 to 0.8)	1.7 (1.4 to 2.0)	**154.4 (104.28 to 213.90)**	2.5 (2.0 to 2.9)	4.8 (4.1 to 5.6)	**96.4 (57.11 to 142.08)**	0.7 (0.6 to 0.9)	1.4 (1.2 to 1.6)	**93.5 (52.01 to 139.88)**	2.2 (1.8 to 2.6)	3.5 (3.0 to 4.1)	**62.2 (26.22 to 100.60)**
Cirrhosis due to alcohol use	1.7 (1.6 to 1.9)	2.1 (1.8 to 2.4)	**21.1 (1.87 to 40.16)**	2.7 (2.5 to 3.0)	2.7 (2.3 to 3.1)	-1.1 (-16.73 to 15.35)	1.0 (0.8 to 1.1)	1.6 (1.3 to 1.9)	**64.1 (35.44 to 101.86)**	3.6 (3.1 to 4.0)	4.5 (3.7 to 5.2)	**24.4 (2.33 to 53.72)**	0.8 (0.6 to 0.9)	0.5 (0.4 to 0.6)	**-34.4 (-47.97 to -17.68)**	2.0 (1.7 to 2.3)	1.1 (0.9 to 1.4)	**-44.8 (-57.55 to -30.88)**
Cirrhosis due to other causes	0.3 (0.3 to 0.4)	0.7 (0.6 to 0.9)	**142.3 (90.24 to 191.40)**	0.5 (0.5 to 0.6)	1.1 (0.9 to 1.2)	**100.2 (58.28 to 140.22)**	0.2 (0.1 to 0.2)	0.4 (0.3 to 0.5)	**171.7 (97.73 to 288.31)**	0.6 (0.5 to 0.7)	1.3 (1.0 to 1.6)	**116.6 (58.49 to 208.76)**	0.2 (0.1 to 0.2)	0.3 (0.3 to 0.4)	**117.9 (47.80 to 195.04)**	0.5 (0.4 to 0.6)	0.9 (0.7 to 1.1)	**85.2 (25.67 to 147.18)**
**Digestive diseases**	13.3 (12.6 to 15.2)	16.5 (14.7 to 20.4)	**23.5 (8.30 to 52.13)**	18.2 (17.3 to 20.8)	16.5 (14.7 to 20.1)	-9.8 (-21.15 to 9.20)	5.2 (4.6 to 5.8)	6.7 (5.8 to 9.5)	**30.1 (8.59 to 85.77)**	19.1 (16.7 to 21.3)	16.4 (14.1 to 22.7)	-14.1 (-28.25 to 20.95)	8.2 (7.5 to 9.8)	9.7 (8.3 to 12.1)	**19.4 (0.08 to 38.60)**	17.3 (16.0 to 20.9)	16.2 (13.8 to 19.8)	-6.2 (-22.32 to 7.07)
Peptic ulcer disease	4.2 (3.4 to 4.8)	3.1 (2.5 to 4.6)	-24.7 (-39.47 to 12.49)	5.7 (4.7 to 6.6)	3.1 (2.5 to 4.5)	**-44.9 (-55.20 to -20.21)**	1.9 (1.4 to 2.2)	1.6 (1.1 to 2.7)	-15.4 (-39.62 to 55.22)	6.8 (5.3 to 8.0)	3.7 (2.7 to 6.3)	**-44.3 (-59.98 to -0.16)**	2.3 (1.8 to 2.9)	1.6 (1.2 to 2.4)	**-31.4 (-46.52 to 6.67)**	4.9 (3.8 to 6.2)	2.6 (2.0 to 3.8)	**-46.2 (-57.69 to -19.12)**
Gastritis and duodenitis	0.1 (0.1 to 0.2)	0.2 (0.1 to 0.3)	**86.3 (21.30 to 139.97)**	0.2 (0.1 to 0.3)	0.2 (0.1 to 0.3)	37.7 (-10.21 to 78.70)	<0.05 (0.0 to 0.1)	0.1 (0.0 to 0.2)	**117.4 (16.70 to 225.72)**	0.2 (0.1 to 0.3)	0.3 (0.1 to 0.4)	50.0 (-18.25 to 122.04)	0.1 (0.0 to 0.1)	0.1 (0.0 to 0.2)	**63.0 (6.38 to 108.11)**	0.1 (0.1 to 0.3)	0.2 (0.1 to 0.3)	26.4 (-15.81 to 60.23)
Appendicitis	0.2 (0.2 to 0.3)	0.3 (0.2 to 0.5)	12.3 (-36.37 to 110.58)	0.4 (0.3 to 0.4)	0.3 (0.2 to 0.5)	-19.2 (-51.91 to 42.86)	0.1 (0.1 to 0.1)	0.1 (0.1 to 0.3)	52.3 (-33.67 to 195.43)	0.4 (0.2 to 0.5)	0.4 (0.2 to 0.7)	1.8 (-53.83 to 90.52)	0.1 (0.1 to 0.2)	0.1 (0.1 to 0.2)	-14.4 (-48.81 to 60.57)	0.4 (0.2 to 0.5)	0.2 (0.1 to 0.4)	-34.5 (-58.17 to 16.48)
Paralytic ileus and intestinal obstruction	1.3 (0.9 to 2.0)	2.2 (1.2 to 2.9)	**74.6 (7.79 to 116.52)**	1.7 (1.2 to 2.7)	2.2 (1.2 to 2.8)	27.7 (-21.02 to 57.88)	0.5 (0.3 to 0.8)	0.9 (0.4 to 1.2)	97.7 (-14.98 to 171.42)	1.8 (1.2 to 3.1)	2.2 (0.9 to 2.9)	26.0 (-45.05 to 72.44)	0.8 (0.5 to 1.4)	1.3 (0.6 to 2.0)	**62.6 (12.19 to 98.77)**	1.7 (1.0 to 2.9)	2.1 (1.1 to 3.2)	29.5 (-10.33 to 56.66)
Inguinal, femoral, and abdominal hernia	0.7 (0.5 to 1.1)	0.9 (0.4 to 2.3)	24.8 (-42.46 to 281.06)	1.0 (0.7 to 1.4)	0.8 (0.4 to 2.1)	-10.3 (-58.56 to 174.52)	0.3 (0.2 to 0.4)	0.4 (0.1 to 1.4)	51.8 (-48.66 to 509.96)	1.1 (0.6 to 1.6)	1.0 (0.3 to 3.3)	-4.6 (-67.30 to 281.95)	0.4 (0.2 to 0.7)	0.5 (0.2 to 1.1)	13.8 (-60.42 to 184.23)	0.9 (0.5 to 1.5)	0.7 (0.3 to 1.8)	-11.3 (-67.93 to 120.23)
Inflammatory bowel disease	0.9 (0.7 to 1.2)	1.2 (0.9 to 1.7)	**33.9 (11.79 to 70.39)**	1.2 (1.0 to 1.7)	1.2 (0.9 to 1.7)	-2.1 (-18.45 to 22.65)	0.3 (0.2 to 0.4)	0.4 (0.3 to 0.7)	**51.7 (-10.37 to 129.18)**	1.0 (0.8 to 1.5)	1.0 (0.7 to 1.6)	0.5 (-38.95 to 50.51)	0.6 (0.4 to 0.9)	0.7 (0.5 to 1.2)	**25.9 (3.00 to 61.42)**	1.3 (0.9 to 1.9)	1.3 (0.9 to 2.0)	-1.9 (-18.59 to 21.89)
Vascular intestinal disorders	1.6 (1.0 to 2.5)	2.5 (1.5 to 3.9)	**56.1 (4.79 to 120.00)**	2.2 (1.4 to 3.4)	2.5 (1.5 to 3.9)	15.4 (-21.95 to 63.61)	0.6 (0.3 to 1.2)	0.9 (0.3 to 1.8)	44.5 (-7.79 to 210.93)	2.3 (1.0 to 4.4)	2.2 (0.8 to 4.5)	-3.1 (-38.19 to 105.27)	1.0 (0.6 to 1.9)	1.6 (0.7 to 2.6)	**66.5 (-0.75 to 113.07)**	2.1 (1.2 to 4.0)	2.8 (1.2 to 4.4)	32.7 (-20.77 to 68.15)
Gallbladder and biliary diseases	0.9 (0.8 to 1.2)	1.8 (1.3 to 2.1)	**94.2 (27.51 to 146.97)**	1.2 (1.0 to 1.6)	1.7 (1.2 to 2.1)	39.9 (-9.07 to 77.04)	0.3 (0.3 to 0.4)	0.7 (0.4 to 0.9)	**116.5 (-4.36 to 189.81)**	1.2 (1.0 to 1.6)	1.7 (0.9 to 2.1)	39.1 (-37.39 to 84.97)	0.6 (0.5 to 0.8)	1.0 (0.7 to 1.3)	**82.4 (13.87 to 139.91)**	1.2 (1.0 to 1.7)	1.7 (1.1 to 2.1)	41.4 (-13.60 to 85.01)
Pancreatitis	0.9 (0.7 to 1.1)	1.1 (0.8 to 1.5)	**27.2 (0.12 to 73.09)**	1.3 (1.0 to 1.6)	1.2 (0.9 to 1.7)	-3.5 (-24.49 to 26.38)	0.4 (0.3 to 0.5)	0.5 (0.3 to 0.8)	**39.4 (0.86 to 91.42)**	1.4 (1.0 to 2.0)	1.4 (0.9 to 2.0)	0.2 (-26.63 to 35.50)	0.5 (0.3 to 0.7)	0.5 (0.3 to 0.8)	17.7 (-13.28 to 73.92)	1.1 (0.8 to 1.6)	1.0 (0.6 to 1.5)	-7.2 (-31.19 to 34.45)
Other digestive diseases	2.5 (2.1 to 3.0)	3.3 (2.7 to 4.1)	**29.2 (7.77 to 59.24)**	3.4 (2.9 to 4.0)	3.2 (2.6 to 4.0)	-6.3 (-22.04 to 14.88)	0.8 (0.6 to 1.0)	1.1 (0.7 to 1.5)	**36.5 (-9.36 to 86.16)**	2.9 (2.3 to 3.6)	2.5 (1.7 to 3.5)	-10.9 (-40.35 to 21.80)	1.7 (1.4 to 2.1)	2.2 (1.7 to 2.8)	**26.3 (5.79 to 54.32)**	3.6 (2.9 to 4.3)	3.6 (2.8 to 4.4)	-1.2 (-17.65 to 19.53)
**Neurological disorders**	34.5 (31.6 to 37.6)	49.9 (45.6 to 53.8)	**44.8 (29.46 to 59.43)**	45.4 (41.8 to 49.3)	46.8 (43.1 to 50.2)	3.2 (-7.08 to 13.31)	13.6 (12.3 to 14.8)	22.5 (20.2 to 24.7)	**66.1 (46.72 to 88.40)**	53.4 (48.4 to 58.1)	52.7 (47.5 to 57.7)	-1.1 (-12.28 to 12.56)	20.9 (18.3 to 23.7)	27.4 (24.1 to 30.7)	**31.3 (11.39 to 54.75)**	41.1 (36.4 to 46.2)	42.5 (37.7 to 47.2)	3.6 (-11.19 to 20.11)
Alzheimer disease and other dementias	27.2 (24.6 to 30.1)	41.1 (37.2 to 44.8)	**51.6 (32.51 to 70.58)**	34.5 (31.2 to 38.2)	36.8 (33.3 to 40.0)	6.9 (-6.42 to 20.11)	9.9 (8.7 to 11.0)	17.6 (15.6 to 19.7)	**79.7 (54.59 to 113.46)**	39.3 (34.5 to 43.9)	40.1 (35.5 to 44.8)	2.4 (-11.83 to 20.95)	17.3 (14.7 to 20.1)	23.5 (20.4 to 26.6)	**36.1 (12.65 to 65.86)**	32.1 (27.4 to 37.0)	34.4 (30.1 to 38.9)	7.7 (-10.59 to 30.45)
Parkinson disease	3.4 (2.8 to 3.9)	4.4 (3.5 to 4.9)	**26.9 (11.60 to 40.67)**	4.4 (3.6 to 5.0)	4.2 (3.4 to 4.6)	-5.7 (-16.77 to 4.10)	1.8 (1.6 to 1.9)	2.6 (2.2 to 2.9)	**42.7 (23.23 to 61.22)**	6.8 (5.8 to 7.2)	6.0 (5.1 to 6.8)	**-10.8 (-22.72 to 0.70)**	1.6 (1.1 to 1.9)	1.8 (1.2 to 2.1)	9.3 (-8.11 to 29.45)	3.2 (2.2 to 3.8)	2.8 (1.9 to 3.5)	-10.8 (-24.38 to 4.68)
Epilepsy	0.8 (0.7 to 1.0)	0.8 (0.6 to 0.9)	-0.1 (-21.45 to 17.80)	1.5 (1.4 to 1.9)	1.2 (1.0 to 1.5)	**-19.2 (-34.74 to -5.13)**	0.5 (0.4 to 0.6)	0.4 (0.3 to 0.6)	-2.3 (-22.44 to 21.02)	1.9 (1.5 to 2.4)	1.5 (1.1 to 1.9)	**-20.9 (-36.21 to -2.76)**	0.3 (0.3 to 0.5)	0.4 (0.2 to 0.4)	3.1 (-33.05 to 21.99)	1.2 (1.0 to 1.5)	1.0 (0.7 to 1.2)	**-16.9 (-42.65 to -2.91)**
Multiple sclerosis	0.8 (0.5 to 1.2)	0.9 (0.6 to 1.2)	15.7 (-22.09 to 35.42)	1.3 (0.9 to 2.0)	1.2 (0.8 to 1.6)	-8.0 (-37.44 to 8.62)	0.3 (0.2 to 0.5)	0.3 (0.1 to 0.5)	13.1 (-47.52 to 42.39)	1.0 (0.6 to 1.8)	0.9 (0.4 to 1.5)	-12.6 (-58.99 to 9.77)	0.5 (0.3 to 0.8)	0.6 (0.3 to 0.8)	17.7 (-33.20 to 41.18)	1.5 (1.0 to 2.4)	1.4 (0.8 to 2.2)	-4.6 (-43.56 to 14.43)
Other neurological disorders	2.3 (2.0 to 2.5)	2.7 (2.3 to 3.0)	**18.4 (9.01 to 26.59)**	3.7 (3.2 to 4.1)	3.4 (2.9 to 3.8)	**-6.6 (-13.95 to -0.07)**	1.2 (0.9 to 1.3)	1.5 (1.2 to 1.8)	**29.8 (14.47 to 41.76)**	4.4 (3.3 to 5.1)	4.2 (3.2 to 4.9)	-4.7 (-16.39 to 4.17)	1.1 (1.1 to 1.2)	1.2 (1.1 to 1.3)	6.9 (-4.30 to 17.13)	3.1 (3.0 to 3.3)	2.8 (2.5 to 3.0)	**-10.2 (-19.57 to -1.48)**
**Mental and substance use disorders**	1.2 (1.0 to 1.6)	2.2 (1.7 to 2.4)	**86.2 (10.21 to 110.72)**	2.2 (2.0 to 3.0)	3.7 (2.9 to 4.2)	**70.3 (-1.38 to 93.91)**	0.8 (0.7 to 1.1)	1.6 (1.0 to 1.8)	**114.9 (-10.08 to 151.89)**	3.0 (2.7 to 4.5)	5.7 (3.7 to 6.4)	**93.2 (-18.92 to 125.96)**	0.4 (0.3 to 0.6)	0.6 (0.5 to 0.8)	**37.6 (18.68 to 60.39)**	1.5 (1.2 to 2.0)	1.8 (1.5 to 2.4)	**25.5 (6.90 to 46.39)**
Schizophrenia	0.1 (0.1 to 0.1)	0.1 (0.1 to 0.1)	**-37.1 (-53.31 to 6.01)**	0.2 (0.1 to 0.2)	0.1 (0.1 to 0.1)	**-53.0 (-64.91 to -21.85)**	<0.05 (0.0 to 0.1)	<0.05 (0.0 to <0.05)	-39.7 (-63.98 to 25.45)	0.2 (0.1 to 0.2)	0.1 (0.0 to 0.1)	**-58.4 (-75.00 to -14.01)**	0.1 (0.0 to 0.1)	<0.05 (0.0 to 0.1)	**-34.4 (-51.68 to 8.20)**	0.2 (0.1 to 0.2)	0.1 (0.1 to 0.1)	**-47.6 (-60.20 to -16.74)**
Alcohol use disorders	0.4 (0.3 to 0.7)	0.6 (0.3 to 0.8)	54.6 (-21.04 to 84.07)	0.8 (0.6 to 1.2)	0.9 (0.5 to 1.2)	26.9 (-35.39 to 50.97)	0.3 (0.2 to 0.5)	0.4 (0.2 to 0.5)	67.0 (-34.15 to 104.91)	1.0 (0.8 to 1.8)	1.3 (0.5 to 1.5)	35.4 (-46.52 to 65.80)	0.2 (0.1 to 0.3)	0.2 (0.1 to 0.4)	36.9 (-4.27 to 78.97)	0.6 (0.3 to 1.1)	0.6 (0.3 to 1.2)	13.0 (-20.99 to 46.96)
Drug use disorders	0.6 (0.6 to 0.9)	1.5 (1.1 to 1.6)	**132.6 (16.77 to 164.61)**	1.3 (1.2 to 1.8)	2.7 (2.1 to 3.0)	**113.5 (6.99 to 143.31)**	0.5 (0.4 to 0.7)	1.2 (0.8 to 1.3)	**160.6 (-4.17 to 207.61)**	1.9 (1.7 to 2.8)	4.3 (3.0 to 4.9)	**139.2 (-10.28 to 182.96)**	0.2 (0.2 to 0.2)	0.3 (0.3 to 0.4)	**68.5 (43.82 to 101.45)**	0.7 (0.7 to 0.8)	1.1 (0.9 to 1.3)	**52.5 (30.06 to 82.87)**
Opioid use disorders	0.2 (0.2 to 0.3)	0.5 (0.4 to 0.6)	**151.6 (23.01 to 193.41)**	0.4 (0.4 to 0.6)	1.0 (0.8 to 1.1)	**131.9 (13.68 to 170.77)**	0.2 (0.1 to 0.2)	0.4 (0.3 to 0.5)	**179.8 (1.12 to 239.51)**	0.6 (0.6 to 1.0)	1.6 (1.1 to 1.9)	**158.0 (-4.33 to 213.32)**	0.1 (0.1 to 0.1)	0.1 (0.1 to 0.1)	**82.9 (50.31 to 121.38)**	0.2 (0.2 to 0.3)	0.4 (0.3 to 0.5)	**66.1 (35.13 to 101.74)**
Cocaine use disorders	<0.05 (0.0 to <0.05)	<0.05 (0.0 to 0.1)	**69.4 (-8.61 to 108.23)**	0.1 (0.0 to 0.1)	0.1 (0.1 to 0.1)	**56.8 (-14.29 to 93.31)**	<0.05 (0.0 to <0.05)	<0.05 (0.0 to <0.05)	**96.3 (-26.74 to 163.81)**	0.1 (0.1 to 0.1)	0.1 (0.1 to 0.1)	**82.0 (-30.85 to 145.27)**	<0.05 (0.0 to <0.05)	<0.05 (0.0 to <0.05)	**28.5 (1.26 to 63.32)**	<0.05 (0.0 to <0.05)	<0.05 (0.0 to 0.1)	17.7 (-8.15 to 49.88)
Amphetamine use disorders	<0.05 (0.0 to <0.05)	<0.05 (0.0 to <0.05)	**83.7 (1.74 to 121.97)**	<0.05 (0.0 to 0.1)	0.1 (0.1 to 0.1)	**69.0 (-7.36 to 105.34)**	<0.05 (0.0 to <0.05)	<0.05 (0.0 to <0.05)	**114.5 (-22.13 to 175.95)**	0.1 (0.0 to 0.1)	0.1 (0.1 to 0.1)	**97.7 (-27.24 to 155.32)**	<0.05 (0.0 to <0.05)	<0.05 (0.0 to <0.05)	**41.3 (10.41 to 81.32)**	<0.05 (0.0 to <0.05)	<0.05 (0.0 to 0.1)	**28.6 (-1.13 to 67.85)**
Other drug use disorders	0.4 (0.3 to 0.5)	0.8 (0.6 to 0.9)	**129.2 (15.28 to 163.91)**	0.8 (0.7 to 1.0)	1.6 (1.2 to 1.7)	**109.7 (5.09 to 142.43)**	0.3 (0.2 to 0.4)	0.7 (0.4 to 0.8)	**156.0 (-2.69 to 205.89)**	1.1 (1.0 to 1.6)	2.5 (1.7 to 2.8)	**134.2 (-10.26 to 180.72)**	0.1 (0.1 to 0.1)	0.2 (0.2 to 0.2)	**66.6 (41.69 to 99.91)**	0.4 (0.4 to 0.4)	0.6 (0.5 to 0.7)	**50.3 (27.13 to 81.26)**
Eating disorders	<0.05 (0.0 to <0.05)	<0.05 (0.0 to <0.05)	0.0 (-20.39 to 25.22)	<0.05 (0.0 to <0.05)	<0.05 (0.0 to <0.05)	-9.2 (-27.35 to 12.54)	<0.05 (0.0 to <0.05)	<0.05 (0.0 to <0.05)	-6.6 (-29.12 to 22.97)	<0.05 (0.0 to <0.05)	<0.05 (0.0 to <0.05)	-13.7 (-33.87 to 12.86)	<0.05 (0.0 to <0.05)	<0.05 (0.0 to <0.05)	2.0 (-22.95 to 32.56)	<0.05 (0.0 to <0.05)	<0.05 (0.0 to <0.05)	-8.1 (-29.58 to 18.54)
Anorexia nervosa	<0.05 (0.0 to <0.05)	<0.05 (0.0 to <0.05)	0.0 (-20.39 to 25.22)	<0.05 (0.0 to <0.05)	<0.05 (0.0 to <0.05)	-9.2 (-27.35 to 12.54)	<0.05 (0.0 to <0.05)	<0.05 (0.0 to <0.05)	-6.6 (-29.12 to 22.97)	<0.05 (0.0 to <0.05)	<0.05 (0.0 to <0.05)	-13.7 (-33.87 to 12.86)	<0.05 (0.0 to <0.05)	<0.05 (0.0 to <0.05)	2.0 (-22.95 to 32.56)	<0.05 (0.0 to <0.05)	<0.05 (0.0 to <0.05)	-8.1 (-29.58 to 18.54)
**Diabetes, urogenital, blood, and endocrine diseases**	16.9 (16.0 to 18.4)	17.8 (14.9 to 19.4)	5.6 (-13.27 to 16.06)	23.5 (22.2 to 25.6)	17.9 (15.2 to 19.4)	**-23.9 (-36.69 to -16.95)**	7.3 (6.9 to 7.8)	7.5 (6.4 to 8.2)	2.8 (-11.30 to 11.92)	27.3 (25.6 to 29.1)	18.4 (15.8 to 19.9)	**-32.6 (-41.54 to -26.82)**	9.6 (8.7 to 11.0)	10.3 (7.9 to 11.5)	7.8 (-20.73 to 23.18)	21.2 (19.3 to 24.1)	17.4 (13.6 to 19.4)	**-17.8 (-38.04 to -6.78)**
Diabetes mellitus	7.3 (6.7 to 7.7)	4.9 (4.5 to 5.2)	**-33.0 (-37.91 to -28.01)**	10.0 (9.3 to 10.5)	5.0 (4.6 to 5.3)	**-50.4 (-53.89 to -46.91)**	3.2 (3.1 to 3.2)	2.2 (2.1 to 2.4)	**-29.9 (-35.14 to -24.38)**	11.5 (11.1 to 11.8)	5.4 (5.0 to 5.8)	**-52.8 (-56.26 to -49.09)**	4.1 (3.6 to 4.5)	2.6 (2.3 to 2.9)	**-35.4 (-42.73 to -27.75)**	9.0 (8.0 to 9.9)	4.5 (4.0 to 5.0)	**-49.6 (-55.07 to -44.06)**
Acute glomerulonephritis	<0.05 (0.0 to <0.05)	<0.05 (0.0 to <0.05)	**-29.6 (-41.39 to -4.90)**	<0.05 (0.0 to <0.05)	<0.05 (0.0 to <0.05)	**-41.2 (-52.72 to -26.59)**	<0.05 (0.0 to <0.05)	<0.05 (0.0 to <0.05)	-23.4 (-40.59 to 19.35)	<0.05 (0.0 to <0.05)	<0.05 (0.0 to <0.05)	**-42.4 (-54.97 to -16.07)**	<0.05 (0.0 to <0.05)	<0.05 (0.0 to <0.05)	**-35.6 (-47.47 to -9.36)**	<0.05 (0.0 to <0.05)	<0.05 (0.0 to <0.05)	**-41.6 (-55.84 to -26.11)**
Chronic kidney disease	4.5 (3.8 to 4.9)	4.6 (4.0 to 5.4)	0.9 (-9.45 to 25.55)	6.1 (5.2 to 6.6)	4.4 (3.9 to 5.2)	**-27.5 (-34.86 to -9.05)**	2.1 (1.7 to 2.2)	2.1 (1.9 to 2.5)	0.4 (-10.42 to 23.45)	7.9 (6.5 to 8.3)	5.0 (4.5 to 6.0)	**-36.0 (-42.79 to -21.78)**	2.4 (1.8 to 2.7)	2.5 (2.0 to 3.1)	1.6 (-14.47 to 41.06)	5.1 (3.9 to 5.8)	4.0 (3.3 to 5.0)	**-21.6 (-33.73 to 9.03)**
Chronic kidney disease due to diabetes mellitus	1.0 (0.6 to 1.1)	0.7 (0.6 to 1.1)	-23.0 (-35.23 to 17.79)	1.3 (0.9 to 1.5)	0.7 (0.6 to 1.1)	**-42.9 (-51.51 to -12.74)**	0.5 (0.3 to 0.6)	0.4 (0.3 to 0.7)	-21.0 (-39.89 to 48.14)	1.7 (0.9 to 2.1)	0.9 (0.7 to 1.6)	**-49.4 (-61.53 to -5.05)**	0.5 (0.3 to 0.6)	0.4 (0.3 to 0.5)	**-23.9 (-38.56 to 4.46)**	1.0 (0.7 to 1.3)	0.6 (0.5 to 0.8)	**-38.8 (-49.73 to -17.43)**
Chronic kidney disease due to hypertension	1.3 (0.9 to 1.7)	1.3 (0.9 to 1.6)	0.6 (-19.47 to 15.49)	1.7 (1.3 to 2.2)	1.2 (0.9 to 1.5)	**-27.8 (-41.98 to -17.58)**	0.5 (0.4 to 0.8)	0.6 (0.3 to 0.7)	7.5 (-28.91 to 29.50)	2.0 (1.4 to 2.9)	1.4 (0.7 to 1.7)	**-31.1 (-54.47 to -17.12)**	0.7 (0.4 to 1.1)	0.7 (0.4 to 1.0)	-4.4 (-33.67 to 17.61)	1.5 (0.9 to 2.2)	1.1 (0.6 to 1.6)	**-26.1 (-48.29 to -9.16)**
Chronic kidney disease due to glomerulonephritis	0.3 (0.2 to 0.4)	0.3 (0.2 to 0.4)	-13.9 (-48.78 to 11.03)	0.5 (0.3 to 0.5)	0.3 (0.2 to 0.4)	**-39.7 (-62.18 to -22.58)**	0.2 (0.1 to 0.2)	0.1 (0.1 to 0.2)	-19.7 (-55.25 to 20.57)	0.6 (0.4 to 0.7)	0.3 (0.2 to 0.4)	**-46.9 (-69.06 to -21.82)**	0.2 (0.1 to 0.2)	0.1 (0.1 to 0.2)	-6.9 (-49.06 to 23.05)	0.4 (0.2 to 0.5)	0.2 (0.1 to 0.3)	**-32.1 (-60.32 to -11.30)**
Chronic kidney disease due to other causes	2.0 (1.6 to 2.3)	2.3 (1.8 to 2.9)	**15.6 (-1.24 to 54.60)**	2.7 (2.1 to 3.1)	2.2 (1.8 to 2.8)	**-17.4 (-29.58 to 11.23)**	0.9 (0.7 to 1.2)	1.1 (0.8 to 1.4)	11.6 (-7.97 to 39.74)	3.5 (2.5 to 4.4)	2.5 (2.0 to 3.2)	**-29.4 (-41.42 to -11.85)**	1.1 (0.6 to 1.3)	1.2 (0.9 to 1.7)	20.0 (-5.62 to 91.88)	2.2 (1.4 to 2.7)	2.0 (1.5 to 2.8)	-8.0 (-27.41 to 46.85)
Urinary diseases and male infertility	2.7 (2.4 to 4.1)	6.5 (3.6 to 7.7)	**146.9 (3.31 to 214.46)**	3.7 (3.2 to 5.4)	6.1 (3.4 to 7.2)	74.3 (-25.90 to 120.70)	1.0 (0.9 to 1.5)	2.4 (1.2 to 2.9)	**148.3 (-4.86 to 214.93)**	3.8 (3.4 to 5.9)	5.6 (2.8 to 6.7)	52.2 (-41.08 to 92.97)	1.7 (1.5 to 2.7)	4.1 (2.1 to 5.1)	**148.5 (1.48 to 234.15)**	3.6 (3.1 to 5.6)	6.4 (3.3 to 8.0)	89.4 (-19.89 to 152.75)
Interstitial nephritis and urinary tract infections	2.3 (2.0 to 3.5)	5.9 (3.3 to 7.1)	**168.4 (9.52 to 246.50)**	3.1 (2.6 to 4.7)	5.5 (3.1 to 6.6)	89.9 (-21.36 to 144.02)	0.8 (0.7 to 1.2)	2.1 (1.0 to 2.5)	**189.6 (5.09 to 270.51)**	2.9 (2.5 to 4.7)	4.9 (2.4 to 5.9)	75.9 (-35.84 to 124.90)	1.6 (1.3 to 2.5)	3.8 (1.9 to 4.7)	**160.0 (2.95 to 248.06)**	3.2 (2.7 to 5.0)	6.0 (3.0 to 7.4)	99.7 (-18.34 to 165.02)
Urolithiasis	0.1 (0.1 to 0.2)	0.1 (0.1 to 0.3)	12.5 (-46.84 to 160.58)	0.2 (0.1 to 0.3)	0.2 (0.1 to 0.3)	-16.3 (-60.02 to 90.47)	0.1 (0.0 to 0.1)	0.1 (0.0 to 0.1)	37.7 (-63.74 to 236.38)	0.2 (0.1 to 0.3)	0.2 (0.1 to 0.3)	-8.1 (-75.16 to 121.55)	0.1 (0.1 to 0.1)	0.1 (0.0 to 0.2)	0.6 (-44.44 to 136.58)	0.2 (0.1 to 0.3)	0.2 (0.1 to 0.3)	-20.8 (-55.35 to 79.20)
Other urinary diseases	0.3 (0.2 to 0.4)	0.4 (0.2 to 0.7)	55.7 (-36.18 to 199.85)	0.4 (0.2 to 0.6)	0.4 (0.2 to 0.7)	10.5 (-54.52 to 110.81)	0.2 (0.1 to 0.3)	0.2 (0.1 to 0.5)	31.1 (-57.55 to 191.98)	0.7 (0.4 to 1.1)	0.5 (0.2 to 1.1)	-16.5 (-72.20 to 83.79)	0.1 (0.1 to 0.2)	0.2 (0.1 to 0.3)	113.2 (-16.89 to 264.16)	0.2 (0.1 to 0.4)	0.3 (0.1 to 0.5)	56.6 (-35.04 to 161.72)
Gynecological diseases	<0.05 (0.0 to <0.05)	<0.05 (0.0 to <0.05)	**-22.7 (-35.80 to 0.38)**	0.1 (0.0 to 0.1)	<0.05 (0.0 to <0.05)	**-42.2 (-50.94 to -26.56)**	--	--	--	--	--	--	<0.05 (0.0 to <0.05)	<0.05 (0.0 to <0.05)	**-22.7 (-35.80 to 0.38)**	0.1 (0.1 to 0.1)	0.1 (0.1 to 0.1)	**-39.6 (-48.02 to -23.41)**
Uterine fibroids	<0.05 (0.0 to <0.05)	<0.05 (0.0 to <0.05)	**-21.8 (-35.62 to 2.34)**	<0.05 (0.0 to <0.05)	<0.05 (0.0 to <0.05)	**-42.3 (-51.50 to -25.77)**	--	--	--	--	--	--	<0.05 (0.0 to <0.05)	<0.05 (0.0 to <0.05)	**-21.8 (-35.62 to 2.34)**	<0.05 (0.0 to <0.05)	<0.05 (0.0 to <0.05)	**-39.4 (-48.42 to -22.60)**
Polycystic ovarian syndrome	<0.05 (0.0 to <0.05)	<0.05 (0.0 to <0.05)	**-33.9 (-43.03 to -21.08)**	<0.05 (0.0 to <0.05)	<0.05 (0.0 to <0.05)	**-40.6 (-48.67 to -30.76)**	--	--	--	--	--	--	<0.05 (0.0 to <0.05)	<0.05 (0.0 to <0.05)	**-33.9 (-43.03 to -21.08)**	<0.05 (0.0 to <0.05)	<0.05 (0.0 to <0.05)	**-40.7 (-48.71 to -30.91)**
Endometriosis	<0.05 (0.0 to <0.05)	<0.05 (0.0 to <0.05)	**-33.9 (-43.58 to -17.87)**	<0.05 (0.0 to <0.05)	<0.05 (0.0 to <0.05)	**-42.5 (-50.70 to -29.97)**	--	--	--	--	--	--	<0.05 (0.0 to <0.05)	<0.05 (0.0 to <0.05)	**-33.9 (-43.58 to -17.87)**	<0.05 (0.0 to <0.05)	<0.05 (0.0 to <0.05)	**-42.8 (-50.91 to -30.39)**
Genital prolapse	<0.05 (0.0 to <0.05)	<0.05 (0.0 to <0.05)	**-20.1 (-35.15 to 4.79)**	<0.05 (0.0 to <0.05)	<0.05 (0.0 to <0.05)	**-41.0 (-51.03 to -23.68)**	--	--	--	--	--	--	<0.05 (0.0 to <0.05)	<0.05 (0.0 to <0.05)	**-20.1 (-35.15 to 4.79)**	<0.05 (0.0 to <0.05)	<0.05 (0.0 to <0.05)	**-37.4 (-47.31 to -20.03)**
Other gynecological diseases	<0.05 (0.0 to <0.05)	<0.05 (0.0 to <0.05)	**-23.6 (-36.21 to -1.08)**	<0.05 (0.0 to <0.05)	<0.05 (0.0 to <0.05)	**-42.7 (-51.13 to -26.98)**	--	--	--	--	--	--	<0.05 (0.0 to <0.05)	<0.05 (0.0 to <0.05)	**-23.6 (-36.21 to -1.08)**	<0.05 (0.0 to 0.1)	<0.05 (0.0 to <0.05)	**-40.2 (-48.41 to -24.51)**
Hemoglobinopathies and hemolytic anemias	0.3 (0.2 to 0.4)	0.2 (0.2 to 0.3)	**-22.5 (-39.77 to -2.67)**	0.5 (0.3 to 0.6)	0.3 (0.2 to 0.4)	**-41.5 (-54.04 to -25.32)**	0.1 (0.1 to 0.2)	0.1 (0.1 to 0.2)	**-21.4 (-38.52 to 11.29)**	0.5 (0.3 to 0.7)	0.3 (0.2 to 0.4)	**-44.5 (-56.17 to -21.54)**	0.2 (0.1 to 0.2)	0.1 (0.1 to 0.2)	-23.2 (-43.18 to 3.48)	0.4 (0.3 to 0.6)	0.3 (0.2 to 0.4)	**-39.2 (-54.00 to -19.68)**
Thalassemias	<0.05 (0.0 to <0.05)	<0.05 (0.0 to <0.05)	**-43.2 (-54.94 to -26.65)**	<0.05 (0.0 to 0.1)	<0.05 (0.0 to <0.05)	**-44.6 (-56.32 to -27.73)**	<0.05 (0.0 to <0.05)	<0.05 (0.0 to <0.05)	**-42.8 (-53.12 to -24.53)**	<0.05 (0.0 to 0.1)	<0.05 (0.0 to <0.05)	**-44.2 (-54.35 to -25.44)**	<0.05 (0.0 to <0.05)	<0.05 (0.0 to <0.05)	**-43.5 (-57.56 to -19.09)**	<0.05 (0.0 to <0.05)	<0.05 (0.0 to <0.05)	**-44.9 (-58.89 to -19.37)**
Sickle cell disorders	<0.05 (0.0 to 0.1)	<0.05 (0.0 to 0.1)	-10.9 (-28.77 to 20.02)	0.1 (0.1 to 0.1)	0.1 (0.1 to 0.1)	**-27.5 (-41.92 to -2.22)**	<0.05 (0.0 to <0.05)	<0.05 (0.0 to <0.05)	-11.6 (-28.36 to 30.88)	0.1 (0.1 to 0.1)	0.1 (0.0 to 0.1)	**-28.6 (-41.97 to 5.35)**	<0.05 (0.0 to <0.05)	<0.05 (0.0 to <0.05)	-10.1 (-31.09 to 28.17)	0.1 (0.1 to 0.1)	0.1 (0.0 to 0.1)	-25.4 (-42.97 to 7.42)
G6PD deficiency	<0.05 (0.0 to <0.05)	<0.05 (0.0 to <0.05)	**-56.4 (-65.03 to -42.84)**	<0.05 (0.0 to <0.05)	<0.05 (0.0 to <0.05)	**-66.3 (-72.87 to -55.53)**	<0.05 (0.0 to <0.05)	<0.05 (0.0 to <0.05)	**-44.2 (-54.64 to -20.23)**	<0.05 (0.0 to <0.05)	<0.05 (0.0 to <0.05)	**-56.7 (-64.77 to -38.30)**	<0.05 (0.0 to <0.05)	<0.05 (0.0 to <0.05)	**-67.2 (-74.85 to -55.90)**	<0.05 (0.0 to <0.05)	<0.05 (0.0 to <0.05)	**-74.9 (-80.76 to -65.08)**
Other hemoglobinopathies and hemolytic anemias	0.2 (0.2 to 0.3)	0.2 (0.1 to 0.3)	**-22.9 (-41.13 to -2.81)**	0.3 (0.2 to 0.4)	0.2 (0.1 to 0.3)	**-44.7 (-57.26 to -30.45)**	0.1 (0.1 to 0.1)	0.1 (0.0 to 0.1)	**-21.3 (-39.87 to 10.34)**	0.4 (0.2 to 0.5)	0.2 (0.1 to 0.3)	**-48.7 (-60.71 to -27.77)**	0.1 (0.1 to 0.2)	0.1 (0.1 to 0.2)	-23.8 (-44.89 to 4.36)	0.3 (0.2 to 0.4)	0.2 (0.1 to 0.3)	**-41.8 (-56.92 to -21.42)**
Endocrine, metabolic, blood, and immune disorders	2.0 (1.4 to 2.2)	1.6 (1.4 to 2.5)	-16.6 (-30.46 to 30.43)	3.2 (2.2 to 3.4)	2.1 (1.8 to 3.0)	**-33.9 (-44.17 to -1.80)**	0.9 (0.6 to 1.0)	0.7 (0.6 to 1.2)	-20.1 (-39.91 to 46.80)	3.6 (2.2 to 3.9)	2.1 (1.7 to 3.3)	-40.4 (-54.65 to 7.99)	1.1 (0.7 to 1.2)	0.9 (0.8 to 1.4)	-13.1 (-27.03 to 22.91)	2.9 (1.9 to 3.4)	2.1 (1.6 to 2.9)	**-29.0 (-39.02 to -7.20)**
**Musculoskeletal disorders**	1.9 (1.8 to 2.3)	1.7 (1.2 to 2.0)	**-14.0 (-44.49 to 0.65)**	2.8 (2.5 to 3.2)	1.8 (1.3 to 2.1)	**-34.8 (-55.61 to -24.21)**	0.5 (0.4 to 0.7)	0.5 (0.3 to 0.6)	7.9 (-42.49 to 30.63)	1.8 (1.4 to 2.4)	1.3 (0.7 to 1.6)	**-24.5 (-57.94 to -8.83)**	1.5 (1.3 to 1.6)	1.1 (0.7 to 1.4)	**-21.1 (-56.88 to -5.75)**	3.4 (3.1 to 3.8)	2.2 (1.4 to 2.6)	**-36.7 (-63.90 to -24.54)**

Rheumatoid arthritis	1.3 (1.0 to 1.4)	0.7 (0.6 to 0.9)	**-40.0 (-52.67 to -28.29)**	1.7 (1.4 to 1.9)	0.8 (0.6 to 0.9)	**-54.6 (-64.10 to -46.26)**	0.2 (0.2 to 0.3)	0.2 (0.1 to 0.3)	-18.4 (-60.08 to 6.54)	0.8 (0.6 to 1.1)	0.5 (0.2 to 0.6)	**-43.2 (-71.74 to -25.52)**	1.0 (0.8 to 1.1)	0.6 (0.4 to 0.7)	**-44.8 (-60.74 to -30.31)**	2.3 (1.8 to 2.5)	1.0 (0.7 to 1.2)	**-55.6 (-68.16 to -44.58)**
Other musculoskeletal disorders	0.7 (0.6 to 0.9)	0.9 (0.6 to 1.1)	34.5 (-30.40 to 61.82)	1.0 (1.0 to 1.4)	1.0 (0.7 to 1.2)	-1.7 (-45.13 to 17.56)	0.3 (0.2 to 0.4)	0.3 (0.2 to 0.4)	33.7 (-33.43 to 73.70)	0.9 (0.7 to 1.4)	0.8 (0.5 to 1.1)	-7.0 (-50.95 to 19.42)	0.4 (0.4 to 0.6)	0.6 (0.3 to 0.7)	**35.8 (-45.41 to 66.01)**	1.2 (1.1 to 1.5)	1.2 (0.7 to 1.5)	0.8 (-51.68 to 22.69)
**Other non-communicable diseases**	3.9 (3.6 to 4.1)	3.6 (3.2 to 4.0)	-9.1 (-18.80 to 3.20)	9.0 (8.1 to 9.5)	5.8 (5.2 to 6.8)	**-35.1 (-43.87 to -22.78)**	1.9 (1.7 to 2.0)	1.5 (1.3 to 1.7)	**-21.0 (-30.95 to -6.32)**	9.5 (8.4 to 10.1)	5.7 (5.0 to 6.7)	**-39.7 (-48.41 to -25.67)**	2.0 (1.8 to 2.2)	2.1 (1.7 to 2.3)	2.3 (-11.31 to 16.99)	8.3 (7.3 to 9.2)	5.8 (5.0 to 6.9)	**-30.2 (-40.31 to -17.13)**
Congenital anomalies	2.3 (2.2 to 2.5)	1.6 (1.4 to 1.9)	**-28.6 (-37.43 to -17.77)**	5.8 (5.5 to 6.2)	3.6 (3.1 to 4.2)	**-37.6 (-46.42 to -26.22)**	1.2 (1.2 to 1.3)	0.9 (0.7 to 1.0)	**-29.6 (-38.46 to -18.28)**	6.2 (5.8 to 6.7)	3.8 (3.2 to 4.6)	**-38.2 (-47.30 to -26.72)**	1.1 (1.0 to 1.2)	0.8 (0.7 to 0.9)	**-27.5 (-38.32 to -14.31)**	5.3 (4.9 to 5.9)	3.4 (2.8 to 4.1)	**-37.2 (-47.34 to -25.01)**
Neural tube defects	0.1 (0.1 to 0.2)	0.1 (0.0 to 0.1)	**-51.7 (-65.81 to -38.03)**	0.4 (0.3 to 0.4)	0.2 (0.1 to 0.2)	**-53.9 (-67.80 to -40.34)**	0.1 (0.0 to 0.1)	<0.05 (0.0 to <0.05)	**-47.1 (-64.01 to -30.72)**	0.3 (0.2 to 0.4)	0.1 (0.1 to 0.2)	**-49.5 (-65.99 to -33.07)**	0.1 (0.1 to 0.1)	<0.05 (0.0 to <0.05)	**-54.8 (-68.10 to -39.66)**	0.4 (0.4 to 0.5)	0.2 (0.1 to 0.3)	**-56.9 (-69.78 to -41.76)**
Congenital heart anomalies	1.2 (1.1 to 1.3)	0.6 (0.6 to 0.7)	**-45.8 (-52.72 to -38.46)**	2.9 (2.8 to 3.1)	1.4 (1.2 to 1.6)	**-51.8 (-58.55 to -44.10)**	0.6 (0.6 to 0.7)	0.3 (0.3 to 0.4)	**-47.3 (-54.61 to -37.98)**	3.2 (2.9 to 3.5)	1.5 (1.3 to 1.8)	**-53.1 (-60.08 to -44.51)**	0.5 (0.5 to 0.6)	0.3 (0.3 to 0.4)	**-43.9 (-53.28 to -34.89)**	2.6 (2.4 to 2.9)	1.3 (1.1 to 1.6)	**-50.4 (-58.91 to -41.42)**
Cleft lip and cleft palate	<0.05 (0.0 to <0.05)	<0.05 (0.0 to <0.05)	**-69.9 (-90.83 to -39.83)**	<0.05 (0.0 to <0.05)	<0.05 (0.0 to <0.05)	**-70.6 (-91.07 to -41.23)**	<0.05 (0.0 to <0.05)	<0.05 (0.0 to <0.05)	**-70.3 (-93.34 to -24.82)**	<0.05 (0.0 to <0.05)	<0.05 (0.0 to <0.05)	**-70.9 (-93.51 to -26.44)**	<0.05 (0.0 to <0.05)	<0.05 (0.0 to <0.05)	**-66.4 (-90.98 to -38.33)**	<0.05 (0.0 to <0.05)	<0.05 (0.0 to <0.05)	**-67.2 (-91.19 to -39.82)**
Down syndrome	0.1 (0.1 to 0.2)	0.2 (0.1 to 0.2)	51.4 (-6.54 to 92.80)	0.2 (0.2 to 0.3)	0.3 (0.2 to 0.4)	24.8 (-24.46 to 59.19)	0.1 (0.0 to 0.1)	0.1 (0.0 to 0.1)	51.1 (-30.77 to 94.05)	0.2 (0.2 to 0.4)	0.3 (0.2 to 0.4)	23.9 (-44.19 to 59.75)	0.1 (0.0 to 0.1)	0.1 (0.0 to 0.1)	53.8 (-23.07 to 101.23)	0.2 (0.2 to 0.3)	0.3 (0.2 to 0.4)	27.3 (-38.41 to 66.93)
Chromosomal unbalanced rearrangements	0.1 (0.1 to 0.1)	0.1 (0.1 to 0.1)	9.8 (-22.98 to 47.74)	0.2 (0.2 to 0.3)	0.2 (0.2 to 0.3)	6.2 (-26.05 to 43.96)	<0.05 (0.0 to <0.05)	<0.05 (0.0 to 0.1)	19.4 (-28.31 to 61.40)	0.2 (0.1 to 0.3)	0.2 (0.1 to 0.3)	15.0 (-31.59 to 56.68)	0.1 (0.0 to 0.1)	0.1 (0.0 to 0.1)	4.5 (-29.04 to 42.74)	0.3 (0.2 to 0.5)	0.3 (0.2 to 0.4)	1.4 (-32.15 to 39.35)
Other congenital anomalies	0.8 (0.7 to 0.8)	0.7 (0.6 to 0.8)	**-14.4 (-27.74 to 1.89)**	2.0 (1.9 to 2.2)	1.5 (1.2 to 1.8)	**-26.4 (-39.38 to -10.12)**	0.4 (0.4 to 0.5)	0.4 (0.3 to 0.5)	-14.6 (-29.14 to 4.50)	2.3 (2.1 to 2.5)	1.7 (1.4 to 2.1)	**-26.0 (-39.29 to -8.38)**	0.3 (0.3 to 0.4)	0.3 (0.2 to 0.4)	-14.0 (-29.74 to 5.04)	1.7 (1.5 to 2.0)	1.3 (1.0 to 1.6)	**-27.1 (-42.66 to -9.04)**
Skin and subcutaneous diseases	0.9 (0.8 to 1.1)	1.7 (1.2 to 1.9)	**94.2 (18.16 to 120.95)**	1.2 (1.1 to 1.5)	1.6 (1.2 to 1.8)	**39.9 (-15.44 to 58.38)**	0.2 (0.2 to 0.3)	0.5 (0.3 to 0.6)	**125.6 (12.15 to 163.33)**	0.9 (0.8 to 1.1)	1.2 (0.8 to 1.4)	43.5 (-27.82 to 68.04)	0.6 (0.6 to 0.7)	1.2 (0.9 to 1.4)	**83.3 (19.67 to 115.57)**	1.3 (1.2 to 1.5)	1.9 (1.3 to 2.2)	**44.4 (-4.33 to 69.17)**
Cellulitis	0.2 (0.2 to 0.4)	0.7 (0.4 to 0.9)	**204.0 (6.36 to 342.71)**	0.3 (0.3 to 0.5)	0.7 (0.4 to 0.8)	**114.7 (-23.32 to 207.54)**	0.1 (0.1 to 0.1)	0.2 (0.1 to 0.3)	**192.0 (1.26 to 340.08)**	0.3 (0.2 to 0.4)	0.5 (0.3 to 0.7)	88.6 (-34.35 to 183.47)	0.2 (0.1 to 0.3)	0.5 (0.2 to 0.6)	**212.2 (7.72 to 379.03)**	0.3 (0.3 to 0.6)	0.7 (0.4 to 1.0)	**136.4 (-14.32 to 255.85)**
Pyoderma	0.1 (0.1 to 0.1)	0.6 (0.4 to 0.9)	**917.2 (436.19 to 1,328.89)**	0.1 (0.1 to 0.1)	0.6 (0.4 to 0.8)	**593.1 (266.30 to 875.13)**	<0.05 (0.0 to <0.05)	0.2 (0.1 to 0.3)	**704.2 (279.65 to 1,062.93)**	0.1 (0.1 to 0.1)	0.5 (0.3 to 0.6)	**414.2 (143.82 to 644.32)**	<0.05 (0.0 to 0.1)	0.4 (0.3 to 0.6)	**1,053.8 (506.16 to 1,597.04)**	0.1 (0.1 to 0.1)	0.7 (0.5 to 1.0)	**732.0 (346.08 to 1,132.74)**
Decubitus ulcer	0.5 (0.4 to 0.6)	0.3 (0.2 to 0.4)	**-41.2 (-57.80 to -16.14)**	0.6 (0.5 to 0.7)	0.3 (0.2 to 0.4)	**-57.7 (-69.52 to -40.06)**	0.1 (0.1 to 0.1)	0.1 (0.1 to 0.1)	-23.4 (-51.11 to 23.12)	0.4 (0.3 to 0.5)	0.2 (0.1 to 0.3)	**-52.7 (-69.71 to -24.14)**	0.4 (0.3 to 0.5)	0.2 (0.2 to 0.3)	**-46.0 (-62.23 to -17.06)**	0.8 (0.6 to 0.9)	0.3 (0.2 to 0.5)	**-57.0 (-69.74 to -34.03)**
Other skin and subcutaneous diseases	0.1 (0.1 to 0.1)	0.1 (0.1 to 0.2)	15.4 (-30.67 to 106.89)	0.1 (0.1 to 0.2)	0.1 (0.1 to 0.2)	-17.9 (-49.30 to 44.50)	<0.05 (0.0 to <0.05)	<0.05 (0.0 to <0.05)	32.7 (-28.83 to 132.42)	0.1 (0.0 to 0.1)	0.1 (0.0 to 0.1)	-14.7 (-53.25 to 50.01)	0.1 (0.0 to 0.1)	0.1 (0.0 to 0.1)	10.2 (-36.96 to 115.33)	0.1 (0.1 to 0.2)	0.1 (0.1 to 0.2)	-15.9 (-49.99 to 58.90)
Sudden infant death syndrome	0.7 (0.4 to 0.9)	0.2 (0.1 to 0.4)	**-66.2 (-83.20 to -23.75)**	2.1 (1.1 to 2.6)	0.6 (0.4 to 1.2)	**-66.9 (-83.55 to -25.33)**	0.4 (0.2 to 0.5)	0.1 (0.1 to 0.2)	**-69.0 (-83.02 to -27.58)**	2.5 (1.2 to 3.0)	0.7 (0.4 to 1.3)	**-69.6 (-83.37 to -29.10)**	0.3 (0.1 to 0.4)	0.1 (0.1 to 0.2)	**-60.9 (-83.95 to -8.74)**	1.7 (0.7 to 2.4)	0.6 (0.3 to 1.2)	**-61.7 (-84.28 to -10.63)**
**Injuries**	17.3 (16.7 to 18.7)	15.6 (13.7 to 16.8)	**-10.0 (-21.38 to -2.17)**	30.8 (29.7 to 32.8)	20.5 (18.7 to 21.9)	**-33.5 (-39.90 to -28.22)**	10.7 (10.2 to 11.4)	9.0 (7.9 to 9.9)	**-16.3 (-25.83 to -8.30)**	43.2 (40.9 to 45.8)	27.6 (24.7 to 30.3)	**-36.0 (-42.55 to -30.10)**	6.6 (6.0 to 7.7)	6.6 (5.3 to 7.4)	0.3 (-20.44 to 13.98)	18.7 (17.4 to 21.1)	13.4 (11.4 to 14.9)	**-28.0 (-39.59 to -20.13)**
**Transport injuries**	4.7 (4.4 to 4.9)	2.6 (2.3 to 2.8)	**-46.0 (-49.67 to -41.97)**	9.3 (8.8 to 9.7)	4.3 (3.9 to 4.6)	**-54.2 (-57.43 to -50.71)**	3.5 (3.4 to 3.6)	1.9 (1.7 to 2.0)	**-46.4 (-51.08 to -41.47)**	14.4 (14.0 to 14.9)	6.5 (6.0 to 7.2)	**-54.7 (-58.58 to -50.46)**	1.2 (1.0 to 1.4)	0.7 (0.6 to 0.9)	**-44.6 (-50.79 to -33.89)**	4.4 (3.5 to 5.0)	2.0 (1.7 to 2.6)	**-53.4 (-57.83 to -46.23)**
Road injuries	4.6 (4.3 to 4.8)	2.4 (2.2 to 2.7)	**-46.7 (-50.51 to -42.59)**	9.0 (8.5 to 9.4)	4.1 (3.7 to 4.4)	**-54.9 (-58.12 to -51.41)**	3.4 (3.2 to 3.5)	1.8 (1.6 to 1.9)	**-47.2 (-51.93 to -42.28)**	14.0 (13.4 to 14.4)	6.2 (5.7 to 6.8)	**-55.3 (-59.11 to -51.19)**	1.2 (1.0 to 1.4)	0.7 (0.5 to 0.9)	**-45.2 (-51.33 to -34.52)**	4.3 (3.4 to 4.8)	2.0 (1.6 to 2.5)	**-53.9 (-58.40 to -46.90)**
Pedestrian road injuries	1.4 (1.1 to 1.7)	0.7 (0.6 to 0.9)	**-49.3 (-55.55 to -39.08)**	2.5 (1.9 to 3.2)	1.0 (0.8 to 1.4)	**-60.0 (-64.61 to -52.97)**	0.9 (0.6 to 1.2)	0.5 (0.3 to 0.7)	**-47.4 (-55.65 to -37.39)**	3.7 (2.5 to 5.1)	1.5 (1.0 to 2.2)	**-59.5 (-65.40 to -52.05)**	0.5 (0.3 to 0.6)	0.2 (0.2 to 0.3)	**-52.5 (-59.89 to -37.69)**	1.5 (1.0 to 1.9)	0.6 (0.4 to 0.8)	**-61.8 (-66.96 to -52.89)**
Cyclist road injuries	0.3 (0.2 to 0.3)	0.1 (0.1 to 0.2)	**-48.4 (-58.35 to -39.57)**	0.6 (0.4 to 0.7)	0.2 (0.2 to 0.3)	**-58.6 (-66.20 to -51.96)**	0.2 (0.1 to 0.3)	0.1 (0.1 to 0.1)	**-50.0 (-61.04 to -35.94)**	0.9 (0.6 to 1.2)	0.4 (0.2 to 0.5)	**-60.5 (-69.16 to -50.88)**	0.1 (0.0 to 0.1)	<0.05 (0.0 to <0.05)	**-42.5 (-53.89 to -28.84)**	0.2 (0.2 to 0.3)	0.1 (0.1 to 0.1)	**-51.9 (-60.91 to -41.90)**
Motorcyclist road injuries	0.7 (0.5 to 1.0)	0.4 (0.2 to 0.5)	**-51.2 (-60.69 to -43.34)**	1.6 (1.1 to 2.1)	0.7 (0.5 to 0.9)	**-55.3 (-64.18 to -48.08)**	0.7 (0.5 to 0.9)	0.3 (0.2 to 0.4)	**-50.7 (-61.33 to -41.95)**	2.9 (2.0 to 3.8)	1.3 (0.9 to 1.7)	**-54.7 (-64.43 to -46.64)**	0.1 (0.0 to 0.1)	<0.05 (0.0 to <0.05)	**-57.0 (-64.13 to -46.38)**	0.2 (0.1 to 0.4)	0.1 (0.0 to 0.2)	**-62.6 (-68.79 to -54.94)**
Motor vehicle road injuries	2.1 (1.8 to 2.5)	1.2 (1.0 to 1.4)	**-42.9 (-48.50 to -36.06)**	4.3 (3.5 to 4.9)	2.1 (1.7 to 2.4)	**-50.9 (-55.48 to -45.12)**	1.5 (1.2 to 1.9)	0.9 (0.7 to 1.0)	**-44.5 (-51.53 to -35.89)**	6.4 (5.0 to 7.7)	3.0 (2.4 to 3.6)	**-51.9 (-57.92 to -44.74)**	0.6 (0.4 to 0.8)	0.4 (0.3 to 0.5)	**-38.7 (-47.76 to -29.29)**	2.2 (1.7 to 2.8)	1.2 (0.9 to 1.4)	**-48.2 (-54.78 to -41.01)**
Other road injuries	<0.05 (0.0 to <0.05)	<0.05 (0.0 to <0.05)	**-53.3 (-63.62 to -23.65)**	0.1 (0.0 to 0.1)	<0.05 (0.0 to <0.05)	**-62.1 (-70.46 to -38.05)**	<0.05 (0.0 to <0.05)	<0.05 (0.0 to <0.05)	**-67.5 (-78.07 to -36.07)**	0.1 (0.0 to 0.1)	<0.05 (0.0 to <0.05)	**-73.8 (-81.87 to -50.15)**	<0.05 (0.0 to <0.05)	<0.05 (0.0 to <0.05)	**-30.9 (-50.11 to -0.02)**	0.1 (0.0 to 0.1)	<0.05 (0.0 to <0.05)	**-42.7 (-57.61 to -16.10)**
Other transport injuries	0.1 (0.1 to 0.2)	0.1 (0.1 to 0.1)	**-23.0 (-35.13 to -10.95)**	0.3 (0.2 to 0.4)	0.2 (0.2 to 0.2)	**-34.2 (-44.37 to -24.33)**	0.1 (0.1 to 0.1)	0.1 (0.1 to 0.1)	**-23.4 (-38.65 to -7.75)**	0.5 (0.4 to 0.6)	0.3 (0.2 to 0.4)	**-34.8 (-47.75 to -21.74)**	<0.05 (0.0 to <0.05)	<0.05 (0.0 to <0.05)	**-20.8 (-35.61 to 0.67)**	0.1 (0.1 to 0.2)	0.1 (0.1 to 0.1)	**-32.6 (-44.48 to -15.24)**
**Unintentional injuries**	6.8 (6.3 to 8.0)	9.2 (7.4 to 10.1)	**35.7 (3.87 to 53.13)**	10.5 (9.9 to 12.1)	10.0 (8.3 to 10.9)	-4.8 (-24.08 to 6.30)	3.1 (2.9 to 3.6)	4.2 (3.3 to 4.7)	**35.2 (1.98 to 55.12)**	12.5 (11.6 to 14.4)	11.4 (9.1 to 12.7)	-8.6 (-29.04 to 4.25)	3.6 (3.2 to 4.7)	4.9 (3.7 to 5.7)	**36.5 (-1.24 to 62.36)**	8.4 (7.5 to 10.4)	8.5 (6.6 to 9.7)	1.6 (-22.77 to 18.39)
Falls	3.5 (3.2 to 4.9)	6.1 (4.3 to 7.0)	**75.5 (8.07 to 113.55)**	4.9 (4.4 to 6.7)	6.0 (4.3 to 6.9)	24.3 (-22.99 to 50.94)	1.3 (1.2 to 1.8)	2.6 (1.7 to 3.0)	**95.7 (12.30 to 135.90)**	5.2 (4.8 to 7.2)	6.4 (4.3 to 7.5)	25.2 (-27.48 to 51.10)	2.2 (1.9 to 3.3)	3.5 (2.3 to 4.2)	**63.9 (-0.44 to 105.37)**	4.4 (3.8 to 6.5)	5.4 (3.6 to 6.4)	26.5 (-22.80 to 57.72)
Drowning	0.3 (0.3 to 0.4)	0.2 (0.2 to 0.3)	**-34.1 (-44.25 to -11.39)**	0.7 (0.6 to 0.8)	0.4 (0.3 to 0.5)	**-45.0 (-53.05 to -31.36)**	0.2 (0.2 to 0.3)	0.2 (0.1 to 0.2)	**-33.3 (-45.50 to -9.01)**	1.1 (0.8 to 1.3)	0.6 (0.5 to 0.8)	**-44.2 (-53.58 to -29.20)**	0.1 (0.1 to 0.1)	0.1 (0.0 to 0.1)	**-36.6 (-48.68 to -17.87)**	0.3 (0.3 to 0.4)	0.2 (0.1 to 0.2)	**-47.8 (-57.77 to -34.63)**
Fire, heat, and hot substances	0.6 (0.5 to 0.7)	0.4 (0.3 to 0.5)	**-39.8 (-50.19 to -0.76)**	1.1 (0.8 to 1.2)	0.5 (0.4 to 0.7)	**-53.8 (-61.62 to -24.33)**	0.3 (0.2 to 0.4)	0.2 (0.2 to 0.3)	-38.0 (-49.91 to 12.59)	1.2 (0.9 to 1.5)	0.6 (0.5 to 0.9)	**-54.1 (-62.72 to -18.51)**	0.3 (0.2 to 0.4)	0.2 (0.1 to 0.2)	**-41.4 (-52.65 to -8.01)**	0.9 (0.7 to 1.0)	0.4 (0.3 to 0.6)	**-53.7 (-62.16 to -29.24)**
Poisonings	0.4 (0.3 to 0.5)	0.2 (0.1 to 0.3)	**-59.3 (-71.69 to -8.45)**	0.8 (0.5 to 0.9)	0.3 (0.2 to 0.5)	**-66.8 (-76.74 to -27.43)**	0.3 (0.1 to 0.3)	0.1 (0.1 to 0.2)	**-66.0 (-77.26 to -11.07)**	1.1 (0.6 to 1.3)	0.3 (0.2 to 0.7)	**-71.7 (-80.86 to -28.35)**	0.1 (0.1 to 0.2)	0.1 (0.1 to 0.1)	-43.9 (-63.30 to 7.73)	0.5 (0.3 to 0.6)	0.2 (0.2 to 0.4)	**-54.7 (-69.80 to -15.59)**
Exposure to mechanical forces	0.3 (0.2 to 0.3)	0.3 (0.2 to 0.3)	19.2 (-23.79 to 33.31)	0.5 (0.5 to 0.6)	0.5 (0.4 to 0.6)	4.1 (-35.80 to 17.86)	0.2 (0.2 to 0.3)	0.2 (0.2 to 0.3)	3.6 (-32.60 to 20.56)	0.9 (0.8 to 1.1)	0.8 (0.6 to 0.9)	-8.0 (-41.99 to 6.77)	<0.05 (0.0 to 0.1)	0.1 (0.1 to 0.1)	**96.3 (0.96 to 142.84)**	0.2 (0.2 to 0.3)	0.3 (0.2 to 0.3)	67.4 (-19.37 to 109.53)
Unintentional firearm injuries	<0.05 (0.0 to <0.05)	<0.05 (0.0 to <0.05)	**-47.8 (-53.65 to -40.59)**	<0.05 (0.0 to <0.05)	<0.05 (0.0 to <0.05)	**-54.1 (-59.32 to -47.77)**	<0.05 (0.0 to <0.05)	<0.05 (0.0 to <0.05)	**-60.1 (-65.60 to -52.36)**	0.1 (0.1 to 0.1)	<0.05 (0.0 to <0.05)	**-64.9 (-69.77 to -58.55)**	<0.05 (0.0 to <0.05)	<0.05 (0.0 to <0.05)	10.5 (-31.11 to 29.52)	<0.05 (0.0 to <0.05)	<0.05 (0.0 to <0.05)	-5.0 (-41.02 to 11.54)
Unintentional suffocation	<0.05 (0.0 to 0.1)	0.2 (0.1 to 0.2)	**299.9 (9.14 to 401.45)**	0.1 (0.1 to 0.2)	0.3 (0.1 to 0.3)	**243.0 (-5.56 to 333.44)**	<0.05 (0.0 to 0.1)	0.1 (0.0 to 0.1)	**343.9 (1.55 to 444.14)**	0.1 (0.1 to 0.3)	0.4 (0.2 to 0.5)	**281.9 (-12.47 to 372.44)**	<0.05 (0.0 to <0.05)	<0.05 (0.0 to 0.1)	233.3 (-35.20 to 351.00)	0.1 (0.0 to 0.1)	0.1 (0.0 to 0.2)	179.0 (-46.66 to 285.30)
Other exposure to mechanical forces	0.2 (0.2 to 0.2)	0.1 (0.1 to 0.2)	**-26.3 (-36.74 to -9.68)**	0.4 (0.4 to 0.4)	0.2 (0.2 to 0.3)	**-39.7 (-48.16 to -27.08)**	0.2 (0.2 to 0.2)	0.1 (0.1 to 0.1)	**-39.1 (-46.97 to -23.17)**	0.7 (0.6 to 0.8)	0.4 (0.3 to 0.5)	**-48.3 (-54.94 to -35.91)**	<0.05 (0.0 to <0.05)	<0.05 (0.0 to 0.1)	52.2 (-15.28 to 90.41)	0.1 (0.1 to 0.1)	0.1 (0.1 to 0.1)	20.1 (-32.71 to 49.12)
Adverse effects of medical treatment	0.9 (0.6 to 1.0)	0.8 (0.6 to 1.0)	-11.6 (-29.65 to 24.00)	1.3 (0.9 to 1.4)	0.8 (0.7 to 1.1)	**-33.2 (-46.28 to -6.68)**	0.3 (0.2 to 0.4)	0.3 (0.2 to 0.5)	7.5 (-20.94 to 41.63)	1.2 (0.8 to 1.5)	0.9 (0.6 to 1.2)	**-26.6 (-45.69 to -3.53)**	0.6 (0.4 to 0.7)	0.4 (0.4 to 0.7)	-21.1 (-44.12 to 19.08)	1.3 (0.8 to 1.5)	0.8 (0.7 to 1.3)	**-36.0 (-53.93 to -4.53)**
Animal contact	<0.05 (0.0 to <0.05)	<0.05 (0.0 to <0.05)	**104.9 (11.09 to 152.42)**	<0.05 (0.0 to 0.1)	<0.05 (0.0 to 0.1)	62.2 (-11.66 to 99.46)	<0.05 (0.0 to <0.05)	<0.05 (0.0 to <0.05)	**121.6 (7.73 to 169.43)**	<0.05 (0.0 to 0.1)	0.1 (0.0 to 0.1)	71.5 (-15.52 to 106.64)	<0.05 (0.0 to <0.05)	<0.05 (0.0 to <0.05)	**89.5 (2.70 to 149.21)**	<0.05 (0.0 to 0.1)	<0.05 (0.0 to 0.1)	50.8 (-14.83 to 96.70)
Venomous animal contact	<0.05 (0.0 to <0.05)	<0.05 (0.0 to <0.05)	36.2 (-10.95 to 84.05)	<0.05 (0.0 to <0.05)	<0.05 (0.0 to <0.05)	13.3 (-27.24 to 49.81)	<0.05 (0.0 to <0.05)	<0.05 (0.0 to <0.05)	47.0 (-20.59 to 88.32)	<0.05 (0.0 to <0.05)	<0.05 (0.0 to <0.05)	20.2 (-33.81 to 51.93)	<0.05 (0.0 to <0.05)	<0.05 (0.0 to <0.05)	26.4 (-20.30 to 93.69)	<0.05 (0.0 to <0.05)	<0.05 (0.0 to <0.05)	6.5 (-33.34 to 61.42)
Non-venomous animal contact	<0.05 (0.0 to <0.05)	<0.05 (0.0 to <0.05)	**144.6 (14.00 to 198.69)**	<0.05 (0.0 to <0.05)	<0.05 (0.0 to <0.05)	93.1 (-8.44 to 135.72)	<0.05 (0.0 to <0.05)	<0.05 (0.0 to <0.05)	**162.4 (7.48 to 225.42)**	<0.05 (0.0 to <0.05)	<0.05 (0.0 to 0.1)	101.0 (-17.51 to 147.74)	<0.05 (0.0 to <0.05)	<0.05 (0.0 to <0.05)	**130.8 (8.23 to 216.92)**	<0.05 (0.0 to <0.05)	<0.05 (0.0 to <0.05)	82.9 (-9.25 to 149.85)
Foreign body	0.6 (0.5 to 1.0)	1.1 (0.6 to 1.4)	**98.0 (11.48 to 175.36)**	1.0 (0.8 to 1.6)	1.3 (0.8 to 1.6)	38.0 (-17.66 to 86.84)	0.3 (0.2 to 0.6)	0.6 (0.2 to 0.8)	79.9 (-11.85 to 151.79)	1.4 (0.9 to 2.5)	1.6 (0.7 to 2.1)	24.4 (-35.44 to 71.17)	0.3 (0.2 to 0.4)	0.5 (0.3 to 0.7)	**124.3 (25.31 to 226.40)**	0.7 (0.5 to 1.2)	1.1 (0.6 to 1.5)	**62.2 (-0.69 to 128.82)**
Pulmonary aspiration and foreign body in airway	0.6 (0.5 to 0.9)	1.1 (0.6 to 1.4)	**99.3 (11.58 to 177.80)**	1.0 (0.8 to 1.6)	1.3 (0.8 to 1.6)	38.7 (-17.86 to 88.27)	0.3 (0.2 to 0.6)	0.6 (0.2 to 0.7)	80.9 (-12.02 to 154.15)	1.3 (0.9 to 2.4)	1.6 (0.7 to 2.0)	24.9 (-35.68 to 72.37)	0.3 (0.2 to 0.4)	0.5 (0.3 to 0.7)	**126.0 (25.61 to 230.10)**	0.7 (0.5 to 1.2)	1.1 (0.6 to 1.4)	**63.5 (-0.83 to 130.85)**
Foreign body in other body part	<0.05 (0.0 to <0.05)	<0.05 (0.0 to <0.05)	37.8 (-6.03 to 77.41)	<0.05 (0.0 to <0.05)	<0.05 (0.0 to <0.05)	6.0 (-24.29 to 30.44)	<0.05 (0.0 to <0.05)	<0.05 (0.0 to <0.05)	29.4 (-7.74 to 65.62)	<0.05 (0.0 to <0.05)	<0.05 (0.0 to <0.05)	-0.6 (-29.37 to 24.98)	<0.05 (0.0 to <0.05)	<0.05 (0.0 to <0.05)	48.9 (-22.30 to 110.32)	<0.05 (0.0 to <0.05)	<0.05 (0.0 to <0.05)	13.0 (-38.09 to 44.66)
Other unintentional injuries	0.1 (0.1 to 0.1)	0.1 (0.1 to 0.1)	**-38.4 (-45.16 to -31.21)**	0.2 (0.2 to 0.2)	0.1 (0.1 to 0.1)	**-50.6 (-56.13 to -44.50)**	0.1 (0.1 to 0.1)	<0.05 (0.0 to 0.1)	**-46.8 (-53.89 to -39.66)**	0.4 (0.3 to 0.4)	0.2 (0.1 to 0.2)	**-56.1 (-62.09 to -49.85)**	<0.05 (0.0 to <0.05)	<0.05 (0.0 to <0.05)	-12.2 (-31.86 to 3.09)	0.1 (0.1 to 0.1)	0.1 (0.1 to 0.1)	**-32.1 (-47.12 to -20.81)**
**Self-harm and interpersonal violence**	5.8 (5.1 to 6.2)	3.8 (3.4 to 4.5)	**-33.6 (-40.43 to -21.13)**	11.0 (9.6 to 11.8)	6.2 (5.5 to 7.4)	**-42.9 (-48.91 to -32.52)**	4.1 (3.4 to 4.5)	2.9 (2.4 to 3.5)	**-29.6 (-39.19 to -14.77)**	16.3 (13.3 to 17.9)	9.7 (8.2 to 11.8)	**-40.3 (-48.58 to -27.79)**	1.7 (1.5 to 1.9)	0.9 (0.8 to 1.2)	**-43.3 (-49.95 to -34.91)**	5.9 (5.2 to 6.5)	2.9 (2.5 to 3.5)	**-51.0 (-56.58 to -44.08)**
Self-harm	5.4 (4.8 to 5.9)	3.6 (3.2 to 4.3)	**-32.8 (-39.85 to -20.44)**	10.2 (8.9 to 11.1)	5.9 (5.1 to 7.0)	**-42.3 (-48.48 to -31.72)**	3.9 (3.2 to 4.3)	2.8 (2.3 to 3.3)	**-28.4 (-38.37 to -12.53)**	15.3 (12.5 to 17.0)	9.3 (7.7 to 11.2)	**-39.4 (-47.98 to -26.41)**	1.6 (1.4 to 1.7)	0.9 (0.8 to 1.1)	**-43.6 (-50.03 to -34.97)**	5.4 (4.8 to 6.0)	2.7 (2.3 to 3.3)	**-51.2 (-56.89 to -43.71)**
Interpersonal violence	0.4 (0.3 to 0.5)	0.2 (0.1 to 0.2)	**-46.2 (-53.95 to -32.71)**	0.7 (0.6 to 1.0)	0.3 (0.3 to 0.5)	**-52.4 (-58.87 to -40.51)**	0.2 (0.2 to 0.3)	0.1 (0.1 to 0.2)	**-49.0 (-56.35 to -29.80)**	1.0 (0.7 to 1.5)	0.5 (0.3 to 0.7)	**-54.4 (-61.02 to -37.73)**	0.1 (0.1 to 0.2)	0.1 (0.0 to 0.1)	**-40.0 (-53.13 to -25.19)**	0.4 (0.3 to 0.6)	0.2 (0.2 to 0.3)	**-48.1 (-59.52 to -34.37)**
Assault by firearm	0.1 (0.0 to 0.1)	<0.05 (0.0 to 0.1)	**-42.1 (-61.90 to -14.79)**	0.1 (0.1 to 0.2)	0.1 (0.0 to 0.1)	**-47.3 (-65.24 to -21.68)**	<0.05 (0.0 to 0.1)	<0.05 (0.0 to <0.05)	**-44.4 (-66.81 to -10.48)**	0.2 (0.1 to 0.3)	0.1 (0.1 to 0.2)	**-49.1 (-69.44 to -17.56)**	<0.05 (0.0 to <0.05)	<0.05 (0.0 to <0.05)	**-31.9 (-53.98 to -8.41)**	<0.05 (0.0 to 0.1)	<0.05 (0.0 to <0.05)	**-40.0 (-59.01 to -19.61)**
Assault by sharp object	<0.05 (0.0 to 0.1)	<0.05 (0.0 to 0.1)	**-23.0 (-45.71 to -1.81)**	0.1 (0.1 to 0.2)	0.1 (0.0 to 0.1)	**-29.3 (-49.78 to -9.90)**	<0.05 (0.0 to 0.1)	<0.05 (0.0 to <0.05)	-22.4 (-46.16 to 11.08)	0.1 (0.1 to 0.2)	0.1 (0.1 to 0.2)	**-28.5 (-50.38 to 2.05)**	<0.05 (0.0 to <0.05)	<0.05 (0.0 to <0.05)	**-23.0 (-52.12 to -0.04)**	0.1 (0.0 to 0.1)	<0.05 (0.0 to 0.1)	**-30.2 (-56.05 to -9.40)**
Assault by other means	0.2 (0.2 to 0.3)	0.1 (0.1 to 0.2)	**-51.8 (-58.34 to -37.33)**	0.5 (0.4 to 0.7)	0.2 (0.2 to 0.3)	**-58.2 (-63.86 to -45.70)**	0.2 (0.1 to 0.2)	0.1 (0.0 to 0.1)	**-55.5 (-62.74 to -34.86)**	0.7 (0.4 to 1.0)	0.3 (0.2 to 0.4)	**-60.9 (-67.36 to -43.44)**	0.1 (0.1 to 0.1)	<0.05 (0.0 to 0.1)	**-44.3 (-57.22 to -28.39)**	0.3 (0.3 to 0.5)	0.2 (0.1 to 0.2)	**-52.8 (-64.01 to -38.35)**
**Forces of nature, war, and legal intervention**	<0.05 (0.0 to <0.05)	--	**-100.0 (-100.00 to -100.00)**	<0.05 (0.0 to <0.05)	--	**-100.0 (-100.00 to -100.00)**	<0.05 (0.0 to <0.05)	--	**-100.0 (-100.00 to -100.00)**	<0.05 (0.0 to <0.05)	--	**-100.0 (-100.00 to -100.00)**	<0.05 (0.0 to <0.05)	--	**-100.0 (-100.00 to -100.00)**	<0.05 (0.0 to <0.05)	--	**-100.0 (-100.00 to -100.00)**
Collective violence and legal intervention	<0.05 (0.0 to <0.05)	--	**-100.0 (-100.00 to -100.00)**	<0.05 (0.0 to <0.05)	--	**-100.0 (-100.00 to -100.00)**	<0.05 (0.0 to <0.05)	--	**-100.0 (-100.00 to -100.00)**	<0.05 (0.0 to <0.05)	--	**-100.0 (-100.00 to -100.00)**	<0.05 (0.0 to <0.05)	--	**-100.0 (-100.00 to -100.00)**	<0.05 (0.0 to <0.05)	--	**-100.0 (-100.00 to -100.00)**

Web Table 5 : YLLs in England in 1990 and 2013 for all ages and age-standardized rates (per 100,000) by sex with 95% UI and percentage change. Percent changes in bold denote a statistically significant change.

Cause	Both sexes combined						Males						Females					
	YLLs (in thousands)			Age-standardized rate (per 100,000)			YLLs (in thousands)			Age-standardized rate (per 100,000)			YLLs (in thousands)			Age-standardized rate (per 100,000)		
	1990	2013	% change	1990	2013	% change	1990	2013	% change	1990	2013	% change	1990	2013	% change	1990	2013	% change
**All causes**	8,945.5 (8,905.2 to 8,985.8)	6,545.6 (6,274.9 to 6,812.2)	**-26.8 (-29.91 to -23.72)**	14,743.3 (14,669.8 to 14,817.7)	8,687.6 (8,298.8 to 9,077.8)	**-41.1 (-43.63 to -38.32)**	5,019.9 (4,984.0 to 5,053.5)	3,609.9 (3,412.1 to 3,813.2)	**-28.1 (-32.25 to -24.05)**	18,880.0 (18,726.3 to 19,025.1)	10,427.3 (9,852.4 to 11,016.9)	**-44.8 (-47.89 to -41.52)**	3,925.6 (3,893.9 to 3,957.2)	2,935.7 (2,780.1 to 3,120.4)	**-25.2 (-29.32 to -20.40)**	11,357.6 (11,227.4 to 11,499.1)	7,082.6 (6,669.9 to 7,525.0)	**-37.6 (-41.27 to -33.42)**
**Communicable, maternal, neonatal, and nutritional diseases**	685.7 (655.3 to 782.7)	501.7 (458.9 to 551.5)	**-26.7 (-35.90 to -19.42)**	1,398.8 (1,334.3 to 1,553.9)	816.0 (730.2 to 916.1)	**-41.6 (-50.05 to -33.12)**	349.8 (330.3 to 412.4)	253.0 (225.3 to 278.2)	**-27.5 (-38.62 to -19.01)**	1,611.3 (1,520.5 to 1,860.0)	907.3 (802.2 to 1,023.6)	**-43.5 (-52.54 to -34.92)**	336.0 (315.0 to 390.6)	248.7 (220.9 to 287.0)	**-25.9 (-34.94 to -18.05)**	1,207.1 (1,118.4 to 1,358.9)	725.4 (635.6 to 826.3)	**-39.8 (-48.05 to -31.22)**
**HIV/AIDS and tuberculosis**	26.9 (23.9 to 29.6)	15.5 (12.6 to 18.7)	**-42.4 (-54.12 to -29.38)**	50.6 (44.7 to 55.8)	24.4 (19.8 to 29.4)	**-51.6 (-61.65 to -40.53)**	19.1 (16.6 to 21.3)	10.4 (8.1 to 12.9)	**-45.4 (-58.37 to -30.95)**	74.3 (64.9 to 83.0)	33.3 (26.0 to 41.8)	**-55.0 (-65.51 to -42.64)**	7.9 (7.0 to 8.9)	5.1 (4.1 to 6.5)	**-34.9 (-48.60 to -17.72)**	28.6 (25.3 to 32.2)	15.9 (12.6 to 20.5)	**-44.2 (-56.14 to -28.14)**
Tuberculosis	12.2 (11.6 to 14.1)	6.5 (5.2 to 7.2)	**-46.8 (-58.20 to -39.70)**	21.0 (19.8 to 24.1)	9.0 (7.2 to 9.9)	**-57.2 (-66.11 to -51.81)**	7.6 (7.0 to 8.9)	3.9 (3.0 to 4.5)	**-47.9 (-59.19 to -39.42)**	28.0 (25.8 to 32.9)	11.2 (8.7 to 12.7)	**-59.9 (-68.18 to -53.80)**	4.7 (4.3 to 5.5)	2.6 (2.0 to 2.9)	**-45.0 (-58.26 to -36.55)**	15.4 (14.3 to 18.4)	7.1 (5.5 to 7.9)	**-54.1 (-65.03 to -47.34)**
HIV/AIDS	14.7 (11.9 to 17.0)	9.0 (6.4 to 11.8)	**-38.3 (-58.56 to -15.00)**	29.6 (24.1 to 34.1)	15.5 (11.1 to 20.0)	**-47.4 (-64.02 to -28.39)**	11.5 (9.3 to 13.5)	6.4 (4.5 to 8.9)	**-43.5 (-62.88 to -19.28)**	46.3 (37.2 to 54.2)	22.1 (15.6 to 30.4)	**-51.8 (-67.78 to -31.66)**	3.2 (2.4 to 3.9)	2.5 (1.7 to 3.8)	-19.4 (-47.17 to 20.34)	13.2 (10.1 to 16.1)	8.9 (5.8 to 13.3)	**-32.1 (-54.51 to 1.36)**
HIV/AIDS resulting in mycobacterial infection	1.2 (0.9 to 1.5)	0.5 (0.3 to 0.6)	**-60.1 (-73.70 to -44.55)**	2.4 (1.8 to 3.0)	0.8 (0.5 to 1.1)	**-66.0 (-77.07 to -53.31)**	1.0 (0.7 to 1.3)	0.4 (0.2 to 0.5)	**-62.8 (-75.93 to -46.39)**	3.9 (3.0 to 5.0)	1.2 (0.8 to 1.8)	**-68.3 (-79.14 to -54.61)**	0.2 (0.1 to 0.3)	0.1 (0.1 to 0.2)	**-47.3 (-65.94 to -20.09)**	0.8 (0.6 to 1.1)	0.4 (0.2 to 0.6)	**-55.5 (-70.61 to -33.66)**
HIV/AIDS resulting in other diseases	13.5 (10.9 to 15.7)	8.5 (6.1 to 11.2)	**-36.4 (-57.26 to -12.75)**	27.2 (22.2 to 31.5)	14.7 (10.6 to 19.0)	**-45.8 (-62.85 to -26.21)**	10.5 (8.4 to 12.4)	6.1 (4.2 to 8.4)	**-41.7 (-61.85 to -16.66)**	42.4 (34.1 to 50.0)	20.9 (14.7 to 28.7)	**-50.3 (-66.82 to -29.62)**	3.0 (2.3 to 3.7)	2.4 (1.6 to 3.6)	-17.5 (-45.94 to 23.48)	12.3 (9.5 to 15.0)	8.5 (5.5 to 12.7)	**-30.5 (-53.45 to 3.75)**
**Diarrhea, lower respiratory, and other common infectious diseases**	404.0 (381.1 to 495.8)	342.0 (313.1 to 393.2)	**-15.1 (-26.73 to -7.74)**	648.7 (612.2 to 783.6)	413.6 (379.1 to 459.9)	**-36.0 (-45.58 to -29.76)**	187.2 (173.7 to 243.1)	162.0 (142.3 to 182.5)	**-13.0 (-29.76 to -3.07)**	753.4 (699.4 to 958.5)	455.1 (402.2 to 505.8)	**-39.3 (-50.70 to -32.19)**	216.7 (202.9 to 271.7)	180.0 (158.3 to 222.2)	**-16.8 (-27.88 to -6.12)**	567.9 (532.2 to 697.3)	374.4 (332.9 to 436.8)	**-33.9 (-42.52 to -26.48)**
Diarrheal diseases	4.6 (4.3 to 5.2)	19.8 (17.9 to 21.9)	**330.9 (267.55 to 396.97)**	10.0 (9.1 to 11.6)	28.5 (25.5 to 32.0)	**186.2 (135.02 to 236.04)**	2.4 (2.1 to 2.8)	8.4 (7.3 to 9.5)	**256.5 (189.68 to 324.56)**	11.1 (9.8 to 13.3)	27.3 (23.8 to 31.2)	**147.3 (96.53 to 195.66)**	2.3 (2.0 to 2.6)	11.4 (10.1 to 13.1)	**409.9 (326.54 to 508.30)**	8.9 (7.8 to 10.6)	29.3 (25.7 to 33.8)	**230.4 (165.52 to 301.71)**
Intestinal infectious diseases	3.3 (1.4 to 7.0)	3.6 (1.3 to 7.9)	10.2 (-15.70 to 38.72)	8.2 (3.4 to 17.5)	8.7 (3.0 to 18.8)	5.0 (-21.49 to 34.96)	1.7 (0.7 to 3.8)	2.1 (0.7 to 4.6)	21.5 (-17.24 to 66.58)	8.5 (3.3 to 18.5)	9.8 (3.4 to 21.3)	15.4 (-22.41 to 61.00)	1.6 (0.6 to 3.3)	1.5 (0.6 to 3.4)	-0.6 (-30.24 to 27.66)	7.9 (3.1 to 16.8)	7.5 (2.8 to 16.8)	-5.0 (-35.53 to 24.14)
Typhoid fever	2.4 (0.8 to 5.5)	2.7 (0.9 to 6.1)	13.5 (-7.08 to 36.25)	6.0 (2.0 to 13.7)	6.4 (2.1 to 14.5)	7.7 (-12.76 to 30.80)	1.2 (0.4 to 2.9)	1.5 (0.5 to 3.4)	24.3 (-7.89 to 61.01)	6.1 (2.0 to 14.1)	7.1 (2.3 to 15.9)	17.8 (-13.44 to 56.39)	1.1 (0.4 to 2.5)	1.2 (0.4 to 2.7)	2.9 (-16.80 to 25.98)	5.8 (1.9 to 13.1)	5.7 (1.9 to 13.1)	-2.4 (-20.99 to 21.22)
Paratyphoid fever	0.4 (0.1 to 1.0)	0.7 (0.2 to 1.6)	**68.0 (34.19 to 105.76)**	1.1 (0.4 to 2.5)	1.8 (0.6 to 4.0)	**61.8 (28.23 to 101.75)**	0.2 (0.1 to 0.5)	0.4 (0.1 to 1.0)	**105.9 (50.04 to 166.41)**	1.1 (0.3 to 2.5)	2.1 (0.7 to 4.7)	**97.3 (42.45 to 159.91)**	0.2 (0.1 to 0.5)	0.3 (0.1 to 0.7)	**31.4 (3.85 to 64.20)**	1.1 (0.4 to 2.5)	1.4 (0.5 to 3.3)	**27.6 (0.15 to 61.01)**
Other intestinal infectious diseases	0.5 (0.2 to 0.9)	0.2 (0.1 to 0.5)	-42.7 (-89.10 to 76.54)	1.1 (0.4 to 2.3)	0.5 (0.1 to 1.3)	-45.5 (-91.21 to 84.82)	0.3 (0.1 to 0.7)	0.1 (0.0 to 0.4)	-31.1 (-94.17 to 187.82)	1.3 (0.3 to 3.4)	0.6 (0.1 to 2.0)	-33.9 (-95.41 to 186.04)	0.2 (0.0 to 0.5)	0.1 (0.0 to 0.3)	-28.0 (-95.61 to 243.92)	1.0 (0.1 to 2.8)	0.4 (0.0 to 1.6)	-28.0 (-96.81 to 271.18)
Lower respiratory infections	366.6 (344.4 to 457.4)	302.7 (275.1 to 353.7)	**-17.1 (-28.96 to -9.73)**	559.2 (525.8 to 689.4)	342.8 (312.9 to 386.9)	**-38.5 (-47.98 to -32.78)**	166.6 (153.6 to 223.0)	142.7 (124.5 to 163.2)	**-13.8 (-31.21 to -3.17)**	653.0 (602.9 to 859.0)	379.9 (332.3 to 427.7)	**-41.5 (-53.40 to -34.36)**	200.0 (186.8 to 254.9)	160.1 (140.0 to 202.2)	**-19.8 (-30.87 to -9.16)**	489.3 (458.0 to 613.4)	308.5 (273.1 to 371.4)	**-36.8 (-45.78 to -29.07)**
Upper respiratory infections	0.3 (0.2 to 0.3)	0.1 (0.1 to 0.2)	**-41.3 (-57.61 to -18.78)**	0.4 (0.4 to 0.5)	0.2 (0.2 to 0.3)	**-53.3 (-65.51 to -38.22)**	0.1 (0.1 to 0.2)	0.1 (0.1 to 0.1)	**-42.0 (-63.47 to -13.70)**	0.6 (0.4 to 0.7)	0.3 (0.2 to 0.3)	**-55.1 (-70.84 to -35.47)**	0.1 (0.1 to 0.2)	0.1 (0.0 to 0.1)	**-38.6 (-64.64 to -1.83)**	0.3 (0.3 to 0.5)	0.2 (0.1 to 0.2)	**-50.8 (-68.93 to -26.22)**
Otitis media	1.3 (1.2 to 1.5)	0.5 (0.4 to 0.5)	**-63.4 (-70.93 to -56.41)**	3.1 (2.8 to 3.6)	1.0 (0.9 to 1.2)	**-66.8 (-74.48 to -59.10)**	0.8 (0.7 to 0.9)	0.2 (0.2 to 0.3)	**-71.1 (-77.60 to -64.40)**	3.7 (3.2 to 4.5)	1.0 (0.8 to 1.2)	**-73.8 (-80.26 to -66.67)**	0.5 (0.5 to 0.6)	0.2 (0.2 to 0.3)	**-52.0 (-62.25 to -41.40)**	2.6 (2.2 to 3.1)	1.1 (0.9 to 1.3)	**-56.6 (-67.44 to -45.30)**
Meningitis	22.2 (19.5 to 25.7)	9.7 (8.4 to 11.7)	**-56.0 (-61.62 to -48.11)**	54.2 (46.6 to 64.3)	21.3 (17.9 to 26.4)	**-60.6 (-66.27 to -53.03)**	12.6 (10.0 to 15.9)	5.7 (4.6 to 7.5)	**-54.9 (-62.26 to -45.51)**	61.9 (47.6 to 80.4)	25.1 (19.7 to 34.9)	**-59.3 (-66.69 to -49.61)**	9.6 (8.4 to 10.4)	4.1 (3.6 to 5.1)	**-57.3 (-63.66 to -43.36)**	46.3 (40.9 to 51.0)	17.5 (15.0 to 21.7)	**-62.2 (-68.59 to -52.14)**
Pneumococcal meningitis	3.3 (2.8 to 3.7)	1.5 (1.3 to 1.8)	**-54.5 (-61.70 to -44.20)**	7.7 (6.6 to 9.0)	3.1 (2.6 to 3.9)	**-59.7 (-66.86 to -50.26)**	1.9 (1.5 to 2.3)	0.8 (0.7 to 1.1)	**-54.8 (-64.89 to -43.92)**	9.0 (7.0 to 11.2)	3.6 (2.8 to 4.9)	**-59.6 (-68.88 to -48.58)**	1.4 (1.1 to 1.6)	0.6 (0.5 to 0.8)	**-53.7 (-63.29 to -34.70)**	6.4 (5.3 to 7.6)	2.5 (2.1 to 3.2)	**-59.8 (-68.73 to -45.70)**
H influenzae type B meningitis	4.4 (3.7 to 5.7)	1.9 (1.5 to 2.4)	**-57.8 (-66.25 to -48.29)**	11.7 (9.8 to 15.3)	4.6 (3.6 to 6.1)	**-60.6 (-69.02 to -50.74)**	2.6 (1.9 to 3.7)	1.1 (0.8 to 1.6)	**-55.8 (-67.12 to -42.75)**	13.6 (9.7 to 19.9)	5.6 (3.9 to 8.2)	**-58.6 (-69.92 to -45.31)**	1.8 (1.5 to 2.1)	0.7 (0.6 to 0.9)	**-60.2 (-69.41 to -49.54)**	9.7 (7.9 to 11.6)	3.5 (2.8 to 4.5)	**-63.2 (-72.16 to -52.40)**
Meningococcal meningitis	7.0 (6.1 to 8.0)	3.1 (2.6 to 3.8)	**-55.8 (-62.42 to -46.43)**	17.0 (14.7 to 20.0)	6.7 (5.6 to 8.5)	**-60.5 (-67.09 to -51.73)**	3.9 (3.0 to 4.8)	1.8 (1.4 to 2.3)	**-55.0 (-64.27 to -43.61)**	19.3 (14.6 to 24.4)	7.7 (5.9 to 10.8)	**-59.8 (-68.90 to -47.76)**	3.0 (2.6 to 3.5)	1.3 (1.1 to 1.7)	**-56.5 (-65.26 to -40.20)**	14.7 (12.6 to 16.9)	5.7 (4.7 to 7.3)	**-61.2 (-69.62 to -49.05)**
Other meningitis	7.5 (6.6 to 8.5)	3.3 (2.9 to 4.0)	**-55.7 (-61.57 to -46.04)**	17.8 (15.4 to 20.7)	7.0 (5.9 to 8.7)	**-60.7 (-66.78 to -52.23)**	4.2 (3.4 to 5.2)	1.9 (1.6 to 2.5)	**-54.0 (-61.84 to -45.01)**	19.9 (15.9 to 25.6)	8.2 (6.6 to 11.0)	**-58.9 (-66.56 to -49.79)**	3.3 (2.8 to 3.8)	1.4 (1.2 to 1.8)	**-57.6 (-65.38 to -41.98)**	15.6 (13.2 to 18.0)	5.7 (4.7 to 7.3)	**-63.1 (-70.93 to -49.93)**
Encephalitis	3.4 (2.9 to 4.6)	3.8 (2.4 to 4.7)	15.3 (-40.77 to 54.74)	7.6 (6.5 to 10.4)	7.6 (4.8 to 9.5)	1.9 (-46.73 to 39.21)	1.9 (1.5 to 2.7)	2.0 (1.1 to 2.6)	10.3 (-44.10 to 49.21)	8.7 (7.0 to 12.3)	8.2 (4.7 to 10.7)	-3.4 (-50.67 to 33.68)	1.5 (1.3 to 2.2)	1.7 (1.0 to 2.2)	22.2 (-38.25 to 66.65)	6.6 (5.6 to 9.8)	6.9 (4.2 to 8.9)	9.4 (-43.38 to 51.85)
Diphtheria	0.1 (0.1 to 0.2)	<0.05 (0.0 to 0.1)	**-61.1 (-83.76 to -20.23)**	0.3 (0.2 to 0.6)	0.1 (0.1 to 0.2)	**-64.7 (-86.53 to -24.13)**	0.1 (0.0 to 0.1)	<0.05 (0.0 to <0.05)	**-59.3 (-87.71 to -1.46)**	0.4 (0.2 to 0.8)	0.1 (0.1 to 0.2)	**-62.8 (-89.86 to -3.09)**	<0.05 (0.0 to 0.1)	<0.05 (0.0 to <0.05)	**-56.0 (-87.94 to 16.55)**	0.3 (0.1 to 0.6)	0.1 (0.0 to 0.2)	**-59.6 (-90.12 to 14.60)**
Whooping cough	0.4 (0.3 to 0.6)	0.5 (0.2 to 0.9)	48.4 (-56.66 to 213.34)	0.9 (0.6 to 1.7)	1.2 (0.5 to 2.3)	51.9 (-61.20 to 237.47)	0.2 (0.1 to 0.4)	0.2 (0.1 to 0.5)	52.9 (-60.74 to 260.07)	0.9 (0.6 to 1.9)	1.2 (0.5 to 2.7)	56.3 (-64.09 to 285.55)	0.2 (0.1 to 0.3)	0.2 (0.1 to 0.4)	45.3 (-59.08 to 228.50)	0.9 (0.6 to 1.8)	1.1 (0.5 to 2.4)	49.0 (-64.41 to 257.82)
Tetanus	0.2 (0.2 to 0.3)	0.2 (0.1 to 0.3)	-6.1 (-52.44 to 11.50)	0.6 (0.5 to 0.7)	0.5 (0.2 to 0.6)	-13.6 (-57.62 to 5.48)	0.1 (0.1 to 0.2)	0.1 (0.1 to 0.1)	-5.7 (-56.59 to 16.06)	0.6 (0.5 to 0.8)	0.5 (0.2 to 0.6)	-14.2 (-60.99 to 7.00)	0.1 (0.1 to 0.2)	0.1 (0.1 to 0.1)	-6.2 (-51.71 to 11.68)	0.6 (0.5 to 0.7)	0.5 (0.2 to 0.6)	-12.7 (-56.84 to 6.80)
Measles	0.7 (0.3 to 1.3)	<0.05 (0.0 to <0.05)	**-96.2 (-98.39 to -92.34)**	1.9 (0.8 to 3.6)	0.1 (0.0 to 0.1)	**-96.3 (-98.43 to -92.52)**	0.3 (0.1 to 0.7)	<0.05 (0.0 to <0.05)	**-96.1 (-98.35 to -92.11)**	1.8 (0.8 to 3.6)	0.1 (0.0 to 0.1)	**-96.2 (-98.39 to -92.31)**	0.3 (0.2 to 0.6)	<0.05 (0.0 to <0.05)	**-96.2 (-98.43 to -92.55)**	1.9 (0.9 to 3.7)	0.1 (0.0 to 0.1)	**-96.3 (-98.47 to -92.72)**
Varicella and herpes zoster	1.0 (0.8 to 1.4)	0.9 (0.7 to 1.2)	-9.4 (-38.74 to 28.44)	2.2 (1.7 to 2.9)	1.7 (1.3 to 2.2)	-21.5 (-48.93 to 13.87)	0.5 (0.3 to 0.7)	0.4 (0.3 to 0.6)	-5.3 (-42.40 to 44.33)	2.2 (1.5 to 3.1)	1.7 (1.2 to 2.4)	-20.4 (-52.86 to 24.81)	0.6 (0.4 to 0.8)	0.5 (0.3 to 0.7)	-10.5 (-49.23 to 45.74)	2.2 (1.5 to 3.2)	1.7 (1.2 to 2.5)	-20.6 (-54.69 to 29.47)
**Neglected tropical diseases and malaria**	1.1 (1.0 to 1.3)	1.7 (1.2 to 1.9)	**50.8 (-6.11 to 70.34)**	2.4 (2.3 to 3.0)	3.2 (2.4 to 3.6)	**34.1 (-17.31 to 52.03)**	0.6 (0.5 to 0.7)	1.0 (0.8 to 1.1)	**69.5 (7.99 to 101.13)**	2.5 (2.3 to 3.1)	3.7 (2.7 to 4.3)	**46.3 (-12.84 to 72.71)**	0.5 (0.5 to 0.7)	0.7 (0.4 to 0.8)	31.2 (-42.45 to 50.11)	2.3 (2.2 to 3.0)	2.8 (1.6 to 3.2)	21.6 (-47.12 to 40.96)
Cysticercosis	0.1 (0.1 to 0.1)	<0.05 (0.0 to <0.05)	**-78.3 (-80.41 to -75.94)**	0.1 (0.1 to 0.2)	<0.05 (0.0 to <0.05)	**-81.6 (-83.43 to -79.65)**	<0.05 (0.0 to <0.05)	<0.05 (0.0 to <0.05)	**-78.9 (-81.02 to -76.50)**	0.2 (0.1 to 0.2)	<0.05 (0.0 to <0.05)	**-82.2 (-84.07 to -80.20)**	<0.05 (0.0 to <0.05)	<0.05 (0.0 to <0.05)	**-77.6 (-80.07 to -74.75)**	0.1 (0.1 to 0.1)	<0.05 (0.0 to <0.05)	**-80.9 (-83.03 to -78.53)**
Cystic echinococcosis	0.2 (0.2 to 0.2)	0.1 (0.1 to 0.1)	**-59.5 (-62.75 to -56.16)**	0.4 (0.4 to 0.5)	0.2 (0.1 to 0.2)	**-65.8 (-68.56 to -62.85)**	0.1 (0.1 to 0.1)	<0.05 (0.0 to 0.1)	**-60.8 (-64.19 to -56.99)**	0.5 (0.5 to 0.5)	0.2 (0.1 to 0.2)	**-67.3 (-70.19 to -64.01)**	0.1 (0.1 to 0.1)	<0.05 (0.0 to 0.1)	**-58.2 (-62.21 to -53.84)**	0.4 (0.4 to 0.4)	0.1 (0.1 to 0.2)	**-64.3 (-67.74 to -60.59)**
Dengue	0.3 (0.2 to 0.4)	0.2 (0.2 to 0.3)	-9.1 (-43.84 to 1.90)	0.6 (0.5 to 0.8)	0.5 (0.3 to 0.6)	**-16.3 (-49.17 to -4.76)**	0.1 (0.1 to 0.2)	0.1 (0.1 to 0.1)	-9.1 (-46.92 to 4.25)	0.6 (0.5 to 0.9)	0.5 (0.3 to 0.6)	**-16.5 (-52.42 to -3.13)**	0.1 (0.1 to 0.2)	0.1 (0.1 to 0.1)	-8.6 (-46.77 to 5.43)	0.6 (0.5 to 0.9)	0.5 (0.3 to 0.6)	**-15.6 (-51.06 to -2.08)**
Rabies	0.3 (0.2 to 0.4)	0.2 (0.1 to 0.3)	-17.2 (-54.01 to 1.18)	0.6 (0.5 to 0.8)	0.5 (0.3 to 0.5)	**-24.0 (-58.40 to -6.23)**	0.1 (0.1 to 0.2)	0.1 (0.1 to 0.1)	**-20.5 (-58.98 to 1.10)**	0.6 (0.4 to 0.9)	0.4 (0.2 to 0.6)	**-27.4 (-63.05 to -6.79)**	0.1 (0.1 to 0.2)	0.1 (0.1 to 0.1)	-13.2 (-52.47 to 4.12)	0.6 (0.4 to 0.9)	0.5 (0.2 to 0.6)	**-20.0 (-57.19 to -3.19)**
Other neglected tropical diseases	0.3 (0.3 to 0.4)	1.1 (0.7 to 1.3)	**301.7 (65.27 to 369.14)**	0.6 (0.6 to 1.0)	2.1 (1.3 to 2.4)	**234.0 (39.60 to 290.74)**	0.1 (0.1 to 0.2)	0.7 (0.5 to 0.8)	**371.0 (97.87 to 479.75)**	0.7 (0.6 to 0.9)	2.6 (1.8 to 3.1)	**284.2 (50.58 to 368.94)**	0.1 (0.1 to 0.2)	0.4 (0.1 to 0.5)	**226.7 (-44.19 to 289.39)**	0.6 (0.6 to 1.1)	1.7 (0.5 to 2.0)	**182.1 (-51.01 to 239.26)**
**Maternal disorders**	3.7 (3.3 to 4.0)	2.0 (1.7 to 2.3)	**-45.2 (-53.28 to -36.69)**	7.7 (7.0 to 8.3)	4.1 (3.5 to 4.7)	**-46.8 (-54.56 to -38.49)**	--	--	--	--	--	--	3.7 (3.3 to 4.0)	2.0 (1.7 to 2.3)	**-45.2 (-53.28 to -36.69)**	15.4 (14.1 to 16.7)	8.2 (7.0 to 9.4)	**-46.7 (-54.48 to -38.40)**
Maternal hemorrhage	0.4 (0.3 to 0.5)	0.2 (0.2 to 0.2)	**-51.4 (-62.79 to -37.87)**	0.8 (0.7 to 1.0)	0.4 (0.3 to 0.5)	**-53.0 (-63.99 to -39.82)**	--	--	--	--	--	--	0.4 (0.3 to 0.5)	0.2 (0.2 to 0.2)	**-51.4 (-62.79 to -37.87)**	1.7 (1.4 to 2.0)	0.8 (0.6 to 1.0)	**-52.9 (-63.92 to -39.75)**
Maternal sepsis and other maternal infections	0.4 (0.4 to 0.5)	0.2 (0.2 to 0.2)	**-55.1 (-65.40 to -43.13)**	0.9 (0.8 to 1.1)	0.4 (0.3 to 0.5)	**-56.3 (-66.20 to -44.57)**	--	--	--	--	--	--	0.4 (0.4 to 0.5)	0.2 (0.2 to 0.2)	**-55.1 (-65.40 to -43.13)**	1.8 (1.5 to 2.1)	0.8 (0.6 to 0.9)	**-56.3 (-66.13 to -44.43)**
Maternal hypertensive disorders	0.5 (0.4 to 0.6)	0.3 (0.2 to 0.3)	**-45.8 (-56.36 to -31.91)**	1.0 (0.8 to 1.2)	0.5 (0.5 to 0.6)	**-46.6 (-56.92 to -32.89)**	--	--	--	--	--	--	0.5 (0.4 to 0.6)	0.3 (0.2 to 0.3)	**-45.8 (-56.36 to -31.91)**	2.1 (1.7 to 2.4)	1.1 (0.9 to 1.3)	**-46.5 (-56.78 to -32.74)**
Obstructed labor	0.3 (0.2 to 0.3)	0.2 (0.1 to 0.2)	**-43.8 (-53.91 to -30.28)**	0.6 (0.5 to 0.7)	0.3 (0.3 to 0.4)	**-45.5 (-55.25 to -32.78)**	--	--	--	--	--	--	0.3 (0.2 to 0.3)	0.2 (0.1 to 0.2)	**-43.8 (-53.91 to -30.28)**	1.2 (1.0 to 1.3)	0.6 (0.5 to 0.7)	**-45.5 (-55.18 to -32.75)**
Complications of abortion	0.7 (0.6 to 0.8)	0.4 (0.3 to 0.5)	**-43.8 (-53.80 to -30.39)**	1.4 (1.2 to 1.6)	0.8 (0.6 to 0.9)	**-46.7 (-56.26 to -34.05)**	--	--	--	--	--	--	0.7 (0.6 to 0.8)	0.4 (0.3 to 0.5)	**-43.8 (-53.80 to -30.39)**	2.9 (2.5 to 3.2)	1.5 (1.2 to 1.8)	**-46.7 (-56.24 to -34.06)**
Indirect maternal deaths	0.4 (0.3 to 0.5)	0.2 (0.2 to 0.3)	**-39.5 (-53.24 to -23.59)**	0.8 (0.7 to 1.0)	0.5 (0.4 to 0.6)	**-40.9 (-54.13 to -25.52)**	--	--	--	--	--	--	0.4 (0.3 to 0.5)	0.2 (0.2 to 0.3)	**-39.5 (-53.24 to -23.59)**	1.7 (1.4 to 1.9)	1.0 (0.8 to 1.2)	**-40.8 (-54.03 to -25.45)**
Late maternal deaths	0.2 (0.2 to 0.3)	0.1 (0.1 to 0.2)	**-34.3 (-50.44 to -11.02)**	0.5 (0.4 to 0.6)	0.3 (0.2 to 0.4)	**-36.2 (-52.10 to -13.39)**	--	--	--	--	--	--	0.2 (0.2 to 0.3)	0.1 (0.1 to 0.2)	**-34.3 (-50.44 to -11.02)**	1.0 (0.8 to 1.2)	0.6 (0.5 to 0.8)	**-36.2 (-52.06 to -13.24)**
Maternal deaths aggravated by HIV/AIDS	<0.05 (0.0 to <0.05)	<0.05 (0.0 to <0.05)	14.5 (-3.58 to 35.64)	<0.05 (0.0 to <0.05)	<0.05 (0.0 to <0.05)	8.0 (-9.02 to 28.27)	--	--	--	--	--	--	<0.05 (0.0 to <0.05)	<0.05 (0.0 to <0.05)	14.5 (-3.58 to 35.64)	<0.05 (0.0 to <0.05)	<0.05 (0.0 to <0.05)	7.8 (-9.22 to 28.01)
Other maternal disorders	0.8 (0.7 to 0.9)	0.4 (0.4 to 0.5)	**-42.3 (-53.70 to -28.94)**	1.6 (1.4 to 1.8)	0.9 (0.8 to 1.1)	**-43.5 (-54.49 to -30.43)**	--	--	--	--	--	--	0.8 (0.7 to 0.9)	0.4 (0.4 to 0.5)	**-42.3 (-53.70 to -28.94)**	3.2 (2.8 to 3.6)	1.8 (1.5 to 2.1)	**-43.4 (-54.41 to -30.32)**
**Neonatal disorders**	223.0 (210.2 to 239.5)	121.0 (98.1 to 150.6)	**-45.7 (-56.49 to -31.86)**	638.7 (602.1 to 685.8)	338.7 (274.6 to 421.5)	**-46.9 (-57.50 to -33.43)**	129.9 (122.0 to 140.9)	69.4 (55.3 to 86.7)	**-46.5 (-57.48 to -32.48)**	726.3 (682.3 to 787.7)	379.2 (302.3 to 473.8)	**-47.7 (-58.44 to -34.00)**	93.1 (82.0 to 105.7)	51.7 (40.7 to 65.1)	**-44.4 (-55.44 to -30.39)**	546.9 (481.5 to 620.8)	296.3 (233.1 to 373.5)	**-45.7 (-56.48 to -32.02)**
Preterm birth complications	148.1 (133.3 to 173.1)	89.7 (70.6 to 113.6)	**-39.1 (-53.54 to -20.68)**	424.3 (382.0 to 495.7)	251.2 (197.5 to 318.1)	**-40.5 (-54.59 to -22.51)**	85.9 (76.0 to 105.9)	51.9 (39.2 to 66.5)	**-39.1 (-55.56 to -18.76)**	480.6 (425.2 to 591.9)	283.9 (214.5 to 363.4)	**-40.5 (-56.54 to -20.59)**	62.2 (52.0 to 74.5)	37.8 (28.5 to 48.4)	**-39.0 (-52.84 to -21.75)**	365.1 (305.2 to 437.3)	216.9 (163.7 to 277.5)	**-40.4 (-53.92 to -23.55)**
Neonatal encephalopathy due to birth asphyxia and trauma	55.6 (38.2 to 64.5)	18.0 (12.4 to 25.1)	**-67.2 (-76.66 to -50.67)**	159.2 (109.6 to 184.8)	50.4 (34.8 to 70.2)	**-67.9 (-77.20 to -51.80)**	32.1 (18.4 to 40.4)	9.5 (6.1 to 14.7)	**-69.3 (-80.11 to -47.35)**	179.8 (102.9 to 226.1)	52.1 (33.1 to 80.6)	**-70.0 (-80.56 to -48.52)**	23.4 (16.6 to 30.6)	8.5 (5.3 to 12.1)	**-63.7 (-73.98 to -49.20)**	137.5 (97.5 to 180.0)	48.5 (30.6 to 69.6)	**-64.5 (-74.59 to -50.40)**
Neonatal sepsis and other neonatal infections	7.9 (3.8 to 11.9)	5.3 (3.0 to 11.0)	-29.3 (-64.56 to 31.00)	22.5 (10.9 to 34.1)	14.9 (8.4 to 30.8)	-30.9 (-65.39 to 27.96)	4.9 (1.7 to 8.4)	3.1 (1.5 to 7.6)	-31.5 (-68.70 to 30.91)	27.3 (9.5 to 47.0)	17.0 (8.1 to 41.4)	-33.1 (-69.42 to 27.93)	3.0 (1.5 to 5.2)	2.2 (1.2 to 4.3)	-24.0 (-59.77 to 39.21)	17.6 (8.6 to 30.8)	12.6 (6.7 to 24.5)	-25.8 (-60.71 to 35.94)
Hemolytic disease and other neonatal jaundice	0.6 (0.3 to 0.9)	0.2 (0.1 to 0.4)	**-58.3 (-79.26 to -25.26)**	1.7 (1.0 to 2.7)	0.6 (0.4 to 1.1)	**-59.3 (-79.76 to -26.94)**	0.3 (0.2 to 0.6)	0.1 (0.1 to 0.3)	**-53.2 (-75.34 to -17.29)**	1.8 (0.9 to 3.1)	0.8 (0.4 to 1.4)	**-54.3 (-75.88 to -19.06)**	0.3 (0.1 to 0.5)	0.1 (0.0 to 0.2)	**-61.6 (-88.75 to -18.54)**	1.6 (0.7 to 3.2)	0.5 (0.2 to 1.0)	**-62.5 (-89.04 to -20.34)**
Other neonatal disorders	10.8 (8.4 to 16.1)	7.8 (5.1 to 11.0)	-27.1 (-55.05 to 5.62)	31.0 (24.1 to 46.1)	21.7 (14.4 to 30.6)	**-28.8 (-56.05 to 3.16)**	6.6 (4.7 to 10.5)	4.6 (2.8 to 7.2)	-27.9 (-58.89 to 8.83)	36.8 (26.3 to 58.6)	25.3 (15.4 to 39.5)	-29.5 (-59.79 to 6.37)	4.3 (3.1 to 6.9)	3.1 (1.8 to 4.6)	-24.9 (-61.62 to 11.30)	25.0 (18.1 to 40.2)	17.9 (10.6 to 26.2)	-26.6 (-62.50 to 8.72)
**Nutritional deficiencies**	11.9 (9.7 to 13.4)	5.4 (4.5 to 7.0)	**-54.2 (-59.80 to -40.27)**	18.5 (15.3 to 21.1)	6.5 (5.4 to 8.4)	**-65.0 (-68.95 to -56.08)**	4.2 (2.4 to 5.5)	2.1 (1.5 to 3.3)	**-49.5 (-59.24 to -29.31)**	16.4 (9.7 to 21.8)	5.8 (4.0 to 9.1)	**-64.3 (-70.93 to -50.85)**	7.7 (6.2 to 8.4)	3.4 (2.9 to 4.1)	**-56.4 (-63.46 to -39.47)**	19.9 (16.3 to 21.9)	7.0 (6.0 to 8.3)	**-65.0 (-70.20 to -53.48)**
Protein-energy malnutrition	1.7 (1.1 to 2.7)	1.0 (0.6 to 1.5)	**-37.0 (-59.04 to -15.60)**	3.1 (2.2 to 4.9)	1.5 (0.9 to 2.1)	**-51.1 (-66.63 to -35.24)**	0.7 (0.3 to 1.2)	0.5 (0.2 to 0.9)	-23.5 (-48.18 to 5.76)	2.8 (1.4 to 4.9)	1.6 (0.7 to 2.7)	**-43.4 (-60.46 to -23.89)**	1.0 (0.6 to 1.8)	0.5 (0.2 to 0.9)	**-45.7 (-68.42 to -22.89)**	3.2 (1.9 to 5.5)	1.4 (0.6 to 2.1)	**-56.2 (-73.26 to -39.64)**
Iodine deficiency	0.1 (0.1 to 0.2)	<0.05 (0.0 to 0.1)	**-45.7 (-75.11 to 2.64)**	0.2 (0.1 to 0.3)	0.1 (0.0 to 0.1)	**-57.6 (-79.01 to -24.86)**	<0.05 (0.0 to 0.1)	<0.05 (0.0 to <0.05)	-40.8 (-79.17 to 35.05)	0.2 (0.1 to 0.3)	0.1 (0.0 to 0.1)	**-54.3 (-82.87 to 1.70)**	0.1 (0.0 to 0.1)	<0.05 (0.0 to 0.1)	-44.5 (-82.68 to 32.91)	0.2 (0.1 to 0.3)	0.1 (0.0 to 0.1)	**-56.5 (-84.13 to -5.08)**
Iron-deficiency anemia	7.9 (5.7 to 9.5)	3.8 (2.9 to 4.9)	**-52.4 (-59.93 to -38.20)**	11.6 (8.4 to 14.1)	4.1 (3.2 to 5.4)	**-64.4 (-69.66 to -54.52)**	2.7 (1.5 to 3.5)	1.3 (0.7 to 2.2)	**-51.6 (-63.90 to -23.95)**	10.4 (5.7 to 13.6)	3.4 (1.9 to 5.8)	**-67.0 (-75.04 to -48.26)**	5.2 (3.5 to 6.5)	2.5 (1.9 to 3.2)	**-52.3 (-61.96 to -33.75)**	12.5 (8.1 to 15.8)	4.7 (3.5 to 6.0)	**-61.9 (-69.03 to -48.64)**
Other nutritional deficiencies	2.2 (1.1 to 3.3)	0.6 (0.3 to 1.2)	**-72.1 (-82.08 to -44.90)**	3.7 (1.9 to 5.5)	0.8 (0.5 to 1.5)	**-77.4 (-84.48 to -59.80)**	0.8 (0.3 to 1.2)	0.3 (0.1 to 0.6)	**-63.5 (-77.27 to -25.89)**	3.1 (1.3 to 5.1)	0.8 (0.4 to 1.9)	**-73.1 (-82.27 to -48.08)**	1.4 (0.6 to 2.5)	0.3 (0.1 to 0.7)	**-76.6 (-86.89 to -50.01)**	4.1 (1.8 to 6.5)	0.8 (0.4 to 1.6)	**-79.8 (-86.96 to -61.80)**
**Other communicable, maternal, neonatal, and nutritional diseases**	15.2 (13.2 to 20.4)	14.1 (10.3 to 17.0)	-4.9 (-37.73 to 22.66)	32.1 (27.6 to 43.4)	25.4 (18.3 to 31.0)	-18.5 (-47.38 to 6.65)	8.9 (7.5 to 12.8)	8.2 (5.6 to 10.1)	-4.4 (-39.60 to 26.67)	38.4 (32.3 to 55.9)	30.3 (20.3 to 37.6)	-18.7 (-49.02 to 9.11)	6.3 (5.4 to 8.7)	5.9 (4.2 to 7.1)	-5.0 (-37.34 to 23.00)	26.0 (21.9 to 36.2)	20.7 (14.5 to 25.6)	-18.2 (-47.40 to 9.03)
Sexually transmitted diseases excluding HIV	1.8 (1.3 to 2.5)	1.3 (0.9 to 1.8)	**-27.4 (-50.39 to 1.84)**	4.0 (2.6 to 5.9)	2.5 (1.6 to 4.0)	**-35.4 (-58.50 to -1.09)**	0.7 (0.4 to 1.0)	0.4 (0.2 to 0.6)	**-42.2 (-65.73 to -7.71)**	3.3 (2.0 to 5.2)	1.8 (1.0 to 3.2)	**-42.8 (-68.40 to -4.24)**	1.1 (0.9 to 1.5)	0.9 (0.7 to 1.2)	-18.6 (-43.54 to 12.71)	4.6 (3.1 to 6.7)	3.1 (2.1 to 4.6)	**-30.3 (-55.05 to 3.48)**
Syphilis	1.4 (1.0 to 2.1)	1.0 (0.7 to 1.5)	**-28.8 (-53.91 to 6.88)**	3.3 (2.0 to 5.2)	2.0 (1.2 to 3.4)	**-35.6 (-62.81 to 7.05)**	0.6 (0.4 to 0.9)	0.3 (0.2 to 0.6)	**-40.9 (-67.79 to -1.02)**	2.9 (1.6 to 4.8)	1.6 (0.8 to 3.0)	**-41.4 (-70.63 to 2.94)**	0.8 (0.6 to 1.2)	0.7 (0.5 to 0.9)	-20.4 (-47.74 to 15.29)	3.6 (2.2 to 5.7)	2.4 (1.5 to 3.8)	-31.2 (-58.70 to 10.95)
Chlamydial infection	0.1 (0.1 to 0.1)	0.1 (0.1 to 0.1)	-13.2 (-36.47 to 14.73)	0.2 (0.1 to 0.2)	0.1 (0.1 to 0.2)	**-26.9 (-46.18 to -3.61)**	--	--	--	--	--	--	0.1 (0.1 to 0.1)	0.1 (0.1 to 0.1)	-13.2 (-36.47 to 14.73)	0.3 (0.3 to 0.4)	0.2 (0.2 to 0.3)	**-25.9 (-45.45 to -2.05)**

Gonococcal infection	0.2 (0.1 to 0.2)	0.1 (0.1 to 0.2)	**-24.0 (-40.40 to -0.63)**	0.3 (0.3 to 0.4)	0.2 (0.2 to 0.2)	**-35.5 (-48.47 to -17.85)**	0.1 (0.0 to 0.1)	<0.05 (0.0 to <0.05)	**-50.3 (-62.19 to -14.67)**	0.2 (0.2 to 0.3)	0.1 (0.1 to 0.1)	**-53.8 (-68.88 to -26.73)**	0.1 (0.1 to 0.1)	0.1 (0.1 to 0.1)	-11.7 (-35.78 to 16.41)	0.4 (0.3 to 0.5)	0.3 (0.2 to 0.4)	**-25.2 (-45.29 to -0.97)**
Other sexually transmitted diseases	0.1 (0.1 to 0.1)	0.1 (0.1 to 0.1)	**-23.5 (-38.95 to -2.18)**	0.2 (0.2 to 0.3)	0.2 (0.1 to 0.2)	**-33.8 (-48.44 to -16.04)**	<0.05 (0.0 to <0.05)	<0.05 (0.0 to <0.05)	**-46.5 (-64.94 to -17.15)**	0.1 (0.1 to 0.2)	0.1 (0.0 to 0.1)	**-48.9 (-70.28 to -27.14)**	0.1 (0.1 to 0.1)	0.1 (0.1 to 0.1)	-13.5 (-36.72 to 14.52)	0.3 (0.3 to 0.4)	0.2 (0.2 to 0.3)	**-26.0 (-45.60 to -2.17)**
Hepatitis	2.0 (1.9 to 2.1)	2.7 (2.4 to 3.2)	**33.6 (17.85 to 58.56)**	4.0 (3.8 to 4.2)	4.5 (4.1 to 5.3)	**13.3 (0.04 to 33.88)**	1.5 (1.4 to 1.6)	1.8 (1.6 to 2.2)	**21.9 (3.98 to 48.18)**	6.1 (5.8 to 6.6)	6.2 (5.5 to 7.5)	1.9 (-12.69 to 23.23)	0.5 (0.4 to 0.5)	0.8 (0.7 to 1.0)	**71.1 (47.62 to 103.26)**	1.9 (1.8 to 2.1)	2.8 (2.5 to 3.3)	**48.0 (28.36 to 75.96)**
Acute hepatitis A	0.5 (0.2 to 0.9)	0.9 (0.3 to 1.4)	**69.4 (46.20 to 100.28)**	1.1 (0.5 to 1.9)	1.6 (0.7 to 2.6)	**46.1 (25.39 to 72.80)**	0.4 (0.1 to 0.6)	0.5 (0.2 to 0.9)	**53.8 (28.20 to 86.39)**	1.5 (0.6 to 2.7)	2.0 (0.8 to 3.4)	**31.7 (9.71 to 60.09)**	0.2 (0.1 to 0.3)	0.3 (0.1 to 0.5)	**103.2 (72.29 to 147.10)**	0.7 (0.3 to 1.1)	1.3 (0.6 to 1.9)	**76.6 (47.99 to 115.43)**
Acute hepatitis B	1.4 (1.0 to 1.7)	1.5 (1.0 to 2.2)	12.1 (-5.97 to 35.58)	2.6 (1.9 to 3.3)	2.4 (1.5 to 3.5)	-7.5 (-22.55 to 11.34)	1.1 (0.8 to 1.3)	1.1 (0.7 to 1.6)	3.5 (-14.76 to 30.24)	4.3 (3.1 to 5.3)	3.6 (2.3 to 5.1)	-15.3 (-30.62 to 6.85)	0.3 (0.2 to 0.4)	0.4 (0.3 to 0.6)	**44.6 (18.55 to 74.60)**	1.1 (0.7 to 1.5)	1.3 (0.7 to 1.9)	**21.4 (-0.15 to 46.51)**
Acute hepatitis C	0.1 (0.0 to 0.3)	0.3 (0.1 to 0.6)	**138.1 (77.32 to 231.57)**	0.2 (0.0 to 0.5)	0.4 (0.1 to 0.9)	**95.2 (44.50 to 166.75)**	0.1 (0.0 to 0.2)	0.2 (0.0 to 0.4)	**131.0 (56.62 to 239.64)**	0.3 (0.1 to 0.8)	0.6 (0.1 to 1.4)	**88.5 (29.28 to 172.09)**	<0.05 (0.0 to 0.1)	0.1 (0.0 to 0.2)	**161.7 (80.48 to 268.29)**	0.1 (0.0 to 0.3)	0.2 (0.0 to 0.5)	**116.2 (52.15 to 197.55)**
Other infectious diseases	11.4 (9.6 to 16.6)	10.1 (6.3 to 13.0)	-6.9 (-46.89 to 30.72)	24.2 (20.4 to 35.1)	18.4 (11.4 to 23.9)	-20.0 (-54.63 to 12.66)	6.7 (5.5 to 10.5)	6.0 (3.3 to 7.9)	-5.4 (-48.57 to 35.37)	29.0 (23.6 to 46.2)	22.2 (12.3 to 29.3)	-19.4 (-56.38 to 16.06)	4.7 (4.0 to 7.0)	4.1 (2.4 to 5.4)	-8.4 (-47.94 to 30.21)	19.5 (16.3 to 29.2)	14.7 (8.7 to 19.4)	-20.8 (-55.49 to 13.59)
**Non-communicable diseases**	7,645.4 (7,539.7 to 7,700.3)	5,650.4 (5,418.5 to 5,884.4)	**-26.1 (-29.26 to -22.99)**	12,081.0 (11,906.6 to 12,174.7)	7,176.7 (6,865.2 to 7,484.7)	**-40.6 (-43.27 to -37.99)**	4,223.4 (4,153.9 to 4,261.1)	3,076.3 (2,896.4 to 3,253.5)	**-27.2 (-31.32 to -22.84)**	15,386.1 (15,118.3 to 15,531.2)	8,488.9 (8,002.5 to 8,974.5)	**-44.8 (-47.91 to -41.55)**	3,422.0 (3,361.3 to 3,455.8)	2,574.2 (2,427.7 to 2,737.6)	**-24.8 (-28.94 to -19.83)**	9,506.7 (9,330.0 to 9,620.8)	5,999.4 (5,651.4 to 6,394.3)	**-36.9 (-40.61 to -32.36)**
**Neoplasms**	2,487.4 (2,419.0 to 2,583.5)	2,213.4 (2,086.6 to 2,327.9)	**-11.0 (-16.00 to -6.42)**	4,020.9 (3,909.4 to 4,163.4)	2,881.1 (2,716.1 to 3,037.3)	**-28.3 (-32.23 to -24.64)**	1,311.3 (1,272.2 to 1,354.9)	1,178.0 (1,092.5 to 1,265.4)	**-10.2 (-16.69 to -3.82)**	4,683.4 (4,545.2 to 4,837.8)	3,206.0 (2,968.3 to 3,439.8)	**-31.5 (-36.64 to -26.61)**	1,176.1 (1,115.9 to 1,269.6)	1,035.4 (935.8 to 1,123.1)	**-11.9 (-20.36 to -3.99)**	3,589.9 (3,391.8 to 3,846.5)	2,613.3 (2,381.7 to 2,831.1)	**-27.2 (-33.36 to -20.77)**
Esophageal cancer	90.4 (82.7 to 99.5)	109.2 (95.9 to 134.5)	**20.8 (5.29 to 41.21)**	142.1 (130.4 to 156.6)	137.8 (120.7 to 168.6)	-3.0 (-15.40 to 12.51)	59.8 (52.0 to 68.2)	80.9 (68.5 to 104.0)	**35.4 (13.71 to 64.41)**	211.3 (184.0 to 241.6)	217.3 (183.6 to 277.0)	2.9 (-13.50 to 23.85)	30.6 (29.2 to 33.0)	28.3 (25.2 to 32.0)	-7.5 (-17.88 to 5.05)	82.8 (79.0 to 88.9)	64.3 (57.0 to 73.2)	**-22.3 (-31.46 to -11.33)**
Stomach cancer	151.5 (138.8 to 165.4)	81.9 (72.7 to 93.1)	**-45.9 (-49.97 to -41.09)**	230.4 (211.6 to 250.8)	99.7 (88.9 to 113.0)	**-56.7 (-59.91 to -52.79)**	99.4 (96.7 to 101.6)	52.0 (47.7 to 58.0)	**-47.6 (-52.19 to -40.60)**	348.6 (339.3 to 356.1)	136.9 (125.4 to 153.5)	**-60.7 (-64.14 to -55.44)**	52.1 (39.8 to 66.7)	29.9 (22.2 to 39.5)	**-42.6 (-49.61 to -29.32)**	137.8 (105.5 to 175.7)	66.6 (49.6 to 87.4)	**-51.6 (-57.33 to -41.49)**
Liver cancer	27.8 (24.4 to 34.9)	48.7 (36.8 to 56.3)	**76.1 (30.37 to 108.67)**	45.1 (39.5 to 56.3)	63.2 (48.5 to 73.1)	**41.0 (6.19 to 66.01)**	17.5 (14.5 to 24.5)	30.6 (20.4 to 37.5)	**76.4 (9.01 to 119.46)**	62.6 (51.8 to 87.1)	83.9 (56.3 to 103.1)	35.5 (-15.56 to 67.26)	10.3 (9.5 to 13.2)	18.1 (12.7 to 20.7)	**77.2 (16.57 to 104.85)**	30.4 (28.1 to 38.3)	43.9 (31.2 to 50.1)	**45.2 (-1.64 to 67.47)**
Liver cancer due to hepatitis B	6.0 (4.8 to 7.8)	11.8 (8.8 to 14.8)	**100.3 (39.69 to 172.14)**	10.3 (8.4 to 13.4)	16.2 (12.4 to 20.4)	**60.2 (13.71 to 115.40)**	4.2 (3.1 to 5.9)	7.5 (4.9 to 10.1)	**83.0 (12.14 to 162.33)**	15.3 (11.5 to 21.5)	21.7 (14.2 to 29.1)	44.0 (-10.35 to 105.99)	1.8 (1.4 to 2.5)	4.3 (2.9 to 5.9)	**146.7 (46.08 to 292.28)**	5.8 (4.5 to 7.8)	11.1 (7.7 to 15.0)	**95.0 (20.54 to 206.16)**
Liver cancer due to hepatitis C	6.2 (4.9 to 7.9)	24.7 (18.5 to 30.2)	**305.5 (191.34 to 448.74)**	9.8 (7.8 to 12.5)	31.5 (23.8 to 38.7)	**227.0 (136.70 to 337.78)**	4.0 (2.9 to 5.7)	15.3 (9.9 to 20.1)	**293.5 (136.31 to 472.44)**	14.1 (10.4 to 20.0)	41.6 (27.1 to 54.5)	**202.1 (84.19 to 337.70)**	2.2 (1.7 to 3.0)	9.3 (6.7 to 11.5)	**337.4 (169.44 to 486.06)**	6.2 (4.7 to 8.4)	22.1 (16.1 to 27.5)	**263.3 (128.95 to 398.72)**
Liver cancer due to alcohol use	12.9 (10.8 to 16.5)	9.7 (6.7 to 12.3)	**-23.9 (-51.02 to 0.76)**	20.2 (16.9 to 25.9)	12.0 (8.2 to 15.3)	**-40.1 (-60.69 to -20.64)**	8.2 (6.3 to 11.7)	7.0 (4.2 to 9.5)	-13.1 (-52.39 to 26.41)	29.0 (22.1 to 41.0)	18.7 (11.1 to 25.4)	**-34.5 (-63.90 to -4.82)**	4.6 (3.9 to 5.8)	2.7 (1.8 to 3.4)	**-41.8 (-62.82 to -24.08)**	13.0 (10.9 to 15.9)	5.9 (4.1 to 7.5)	**-53.9 (-68.91 to -40.41)**
Liver cancer due to other causes	2.8 (2.3 to 3.5)	2.5 (1.7 to 3.2)	-10.5 (-40.73 to 20.40)	4.9 (3.9 to 6.0)	3.4 (2.5 to 4.4)	**-28.2 (-51.24 to -4.12)**	1.1 (0.8 to 1.6)	0.7 (0.4 to 1.0)	**-37.8 (-66.51 to -1.14)**	4.2 (3.0 to 5.9)	2.0 (1.2 to 2.8)	**-51.4 (-73.23 to -23.24)**	1.7 (1.3 to 2.2)	1.8 (1.1 to 2.5)	9.1 (-34.78 to 63.09)	5.5 (4.1 to 6.9)	4.8 (3.1 to 6.5)	-10.3 (-44.20 to 32.11)
Larynx cancer	16.9 (13.0 to 20.9)	12.7 (10.0 to 15.7)	**-25.1 (-34.62 to -15.93)**	27.4 (21.2 to 33.9)	16.3 (12.9 to 20.1)	**-40.6 (-47.57 to -33.65)**	14.0 (10.3 to 17.9)	10.0 (7.3 to 13.0)	**-28.2 (-38.70 to -17.56)**	49.5 (36.5 to 63.5)	27.0 (19.7 to 34.8)	**-45.5 (-53.09 to -37.80)**	2.9 (2.0 to 4.0)	2.6 (1.7 to 3.8)	-9.6 (-28.98 to 11.98)	8.7 (5.9 to 11.9)	6.6 (4.2 to 9.4)	**-24.6 (-40.75 to -6.92)**
Lung, bronchus, and trachea cancer	634.3 (618.4 to 667.2)	498.1 (455.5 to 540.7)	**-21.5 (-28.25 to -14.55)**	988.9 (961.6 to 1,034.8)	622.6 (569.2 to 676.9)	**-37.0 (-42.57 to -31.52)**	438.4 (427.7 to 446.1)	289.2 (254.7 to 321.6)	**-34.0 (-42.58 to -26.16)**	1,524.6 (1,487.6 to 1,551.2)	760.8 (668.6 to 845.9)	**-50.1 (-56.67 to -44.06)**	196.0 (184.9 to 229.4)	208.9 (170.9 to 231.3)	6.9 (-13.84 to 19.86)	566.2 (527.5 to 651.5)	502.2 (415.6 to 554.8)	**-11.1 (-26.18 to -0.78)**
Breast cancer	295.5 (247.5 to 344.5)	216.1 (173.1 to 259.1)	**-26.9 (-33.21 to -17.33)**	506.1 (422.8 to 585.9)	294.5 (238.9 to 354.6)	**-41.8 (-46.90 to -33.97)**	1.7 (0.8 to 3.5)	1.4 (0.8 to 2.5)	-17.0 (-36.69 to 14.89)	5.9 (2.7 to 12.5)	3.7 (2.1 to 6.8)	**-36.8 (-51.70 to -13.45)**	293.8 (244.5 to 343.3)	214.7 (171.7 to 257.7)	**-26.9 (-33.30 to -17.36)**	959.0 (794.8 to 1,106.5)	567.7 (460.5 to 684.6)	**-40.8 (-46.07 to -32.71)**
Cervical cancer	40.4 (31.7 to 43.2)	23.5 (19.3 to 27.2)	**-41.7 (-49.81 to -34.11)**	72.1 (56.6 to 77.2)	35.2 (28.8 to 41.0)	**-51.1 (-57.98 to -44.56)**	--	--	--	--	--	--	40.4 (31.7 to 43.2)	23.5 (19.3 to 27.2)	**-41.7 (-49.81 to -34.11)**	139.2 (109.3 to 149.0)	68.9 (56.3 to 80.2)	**-50.4 (-57.40 to -43.73)**
Uterine cancer	16.5 (15.5 to 20.7)	23.0 (17.5 to 26.7)	**40.6 (-2.60 to 63.73)**	25.5 (23.9 to 32.1)	28.6 (21.6 to 33.4)	12.8 (-21.55 to 31.42)	--	--	--	--	--	--	16.5 (15.5 to 20.7)	23.0 (17.5 to 26.7)	**40.6 (-2.60 to 63.73)**	46.7 (43.8 to 58.7)	54.5 (41.0 to 63.9)	17.4 (-18.39 to 37.03)
Prostate cancer	102.5 (79.5 to 136.3)	113.9 (74.3 to 154.9)	11.8 (-32.00 to 32.75)	142.4 (111.2 to 189.0)	127.3 (82.2 to 171.7)	-10.0 (-45.75 to 5.97)	102.5 (79.5 to 136.3)	113.9 (74.3 to 154.9)	11.8 (-32.00 to 32.75)	358.2 (279.3 to 475.5)	281.7 (183.3 to 382.8)	**-20.9 (-51.37 to -6.46)**	--	--	--	--	--	--
Colon and rectum cancer	300.9 (292.9 to 311.0)	246.6 (229.0 to 262.2)	**-18.0 (-24.22 to -12.44)**	466.3 (454.2 to 480.5)	305.4 (283.4 to 325.1)	**-34.5 (-39.34 to -29.87)**	158.9 (154.4 to 161.9)	145.2 (132.8 to 156.6)	**-8.6 (-15.83 to -1.30)**	562.5 (546.7 to 573.1)	385.5 (352.1 to 416.1)	**-31.5 (-36.87 to -25.91)**	142.0 (136.0 to 153.8)	101.4 (91.7 to 110.3)	**-28.5 (-36.12 to -21.88)**	392.4 (376.2 to 423.5)	233.2 (210.5 to 254.6)	**-40.5 (-46.89 to -34.91)**
Mouth cancer	24.6 (23.4 to 26.5)	26.6 (21.4 to 29.2)	8.2 (-18.37 to 18.83)	41.3 (39.3 to 44.3)	36.2 (29.1 to 39.8)	**-12.3 (-33.99 to -3.68)**	17.2 (16.4 to 18.7)	18.5 (13.4 to 20.6)	7.8 (-28.76 to 21.31)	62.7 (59.9 to 68.1)	52.5 (38.2 to 58.7)	**-16.1 (-43.98 to -5.46)**	7.4 (6.6 to 8.4)	8.1 (6.9 to 9.4)	9.3 (-7.26 to 22.86)	22.6 (20.2 to 25.4)	20.7 (17.5 to 24.1)	-8.3 (-23.07 to 3.42)
Nasopharynx cancer	5.9 (4.6 to 7.8)	3.4 (2.9 to 4.9)	**-42.0 (-48.27 to -29.73)**	10.9 (8.6 to 14.3)	5.2 (4.3 to 7.4)	**-52.1 (-56.93 to -42.26)**	4.0 (3.2 to 4.8)	2.3 (2.0 to 3.1)	**-40.8 (-48.29 to -26.35)**	15.1 (12.1 to 18.3)	7.3 (6.1 to 9.7)	**-52.0 (-57.90 to -40.73)**	2.0 (0.9 to 3.7)	1.1 (0.6 to 2.3)	**-44.5 (-53.31 to -28.67)**	6.9 (3.3 to 12.9)	3.3 (1.7 to 6.8)	**-52.7 (-59.62 to -40.09)**
Other pharynx cancer	11.5 (10.4 to 13.5)	12.6 (9.4 to 15.2)	10.3 (-20.90 to 31.41)	19.6 (17.8 to 22.9)	17.3 (13.0 to 20.8)	-11.3 (-35.76 to 5.46)	8.3 (7.5 to 10.3)	9.4 (6.3 to 11.8)	14.2 (-26.48 to 43.38)	30.1 (27.2 to 37.2)	26.6 (18.0 to 33.3)	-11.2 (-41.86 to 10.93)	3.2 (2.6 to 3.8)	3.2 (2.6 to 4.0)	0.9 (-12.69 to 17.08)	10.0 (8.2 to 12.1)	8.4 (6.9 to 10.4)	**-15.9 (-27.06 to -3.43)**
Gallbladder and biliary tract cancer	20.2 (15.3 to 23.7)	14.2 (10.8 to 17.7)	**-29.3 (-39.57 to -11.32)**	30.3 (23.2 to 35.7)	17.2 (13.1 to 21.5)	**-43.1 (-51.35 to -28.65)**	6.8 (5.0 to 8.0)	4.7 (3.4 to 6.0)	**-30.9 (-43.00 to -10.30)**	23.9 (17.5 to 27.9)	12.3 (9.0 to 15.8)	**-48.1 (-57.18 to -33.21)**	13.4 (9.3 to 16.7)	9.5 (6.5 to 12.2)	**-28.4 (-40.08 to -10.42)**	35.4 (25.3 to 44.1)	21.5 (14.8 to 27.5)	**-39.1 (-48.90 to -23.20)**
Pancreatic cancer	111.9 (109.2 to 116.3)	123.1 (114.5 to 131.0)	**10.1 (1.12 to 17.76)**	174.3 (170.0 to 180.9)	154.3 (143.2 to 164.5)	**-11.4 (-18.60 to -5.05)**	59.3 (57.8 to 60.5)	66.5 (61.0 to 71.9)	**12.1 (2.81 to 21.42)**	209.5 (204.4 to 213.9)	178.1 (163.3 to 192.9)	**-15.0 (-22.15 to -7.96)**	52.6 (50.6 to 57.3)	56.7 (50.8 to 61.6)	7.8 (-4.35 to 18.12)	145.0 (139.3 to 157.3)	132.0 (118.4 to 143.8)	**-8.9 (-19.25 to 0.32)**
Malignant skin melanoma	34.7 (25.2 to 50.5)	41.6 (27.9 to 59.3)	**20.1 (-15.05 to 35.24)**	61.5 (44.4 to 89.5)	60.1 (40.6 to 86.6)	-2.1 (-30.20 to 10.09)	17.2 (9.4 to 29.9)	23.9 (11.0 to 39.1)	**40.0 (-32.38 to 64.84)**	64.8 (35.5 to 112.6)	71.2 (32.9 to 117.6)	10.8 (-45.46 to 30.08)	17.5 (12.4 to 24.0)	17.7 (12.1 to 23.1)	1.5 (-13.68 to 14.65)	58.8 (41.5 to 80.4)	49.6 (34.2 to 65.2)	**-15.5 (-27.88 to -4.26)**
Non-melanoma skin cancer	6.5 (4.9 to 8.7)	6.8 (5.0 to 9.2)	5.3 (-2.73 to 14.39)	9.7 (7.3 to 13.0)	8.0 (5.9 to 10.8)	**-17.3 (-23.45 to -9.79)**	3.9 (2.4 to 5.9)	4.1 (2.6 to 6.3)	6.6 (-3.47 to 19.44)	14.1 (8.8 to 21.3)	11.0 (6.8 to 16.7)	**-22.2 (-29.34 to -12.47)**	2.6 (2.0 to 3.3)	2.7 (2.1 to 3.4)	3.6 (-9.95 to 17.89)	6.5 (5.1 to 8.1)	5.5 (4.3 to 7.0)	**-14.2 (-23.97 to -2.82)**
Ovarian cancer	83.1 (78.8 to 90.9)	75.7 (67.4 to 83.9)	**-8.9 (-18.79 to 0.84)**	139.2 (131.4 to 151.9)	100.1 (89.2 to 111.0)	**-28.0 (-35.92 to -20.25)**	--	--	--	--	--	--	83.1 (78.8 to 90.9)	75.7 (67.4 to 83.9)	**-8.9 (-18.79 to 0.84)**	264.2 (248.9 to 288.9)	193.5 (172.2 to 214.7)	**-26.7 (-34.72 to -18.61)**
Testicular cancer	4.9 (3.5 to 6.1)	3.1 (2.3 to 4.0)	**-37.2 (-48.72 to -16.68)**	9.9 (7.0 to 12.3)	5.7 (4.3 to 7.4)	**-42.2 (-52.83 to -23.69)**	4.9 (3.5 to 6.1)	3.1 (2.3 to 4.0)	**-37.2 (-48.72 to -16.68)**	19.9 (14.1 to 24.8)	11.4 (8.5 to 14.8)	**-42.5 (-53.10 to -24.00)**	--	--	--	--	--	--
Kidney cancer	53.9 (50.1 to 58.7)	59.8 (54.0 to 66.0)	**11.0 (-0.16 to 20.83)**	89.1 (82.5 to 96.5)	78.4 (70.5 to 86.9)	**-11.9 (-21.28 to -3.69)**	33.9 (32.8 to 34.9)	39.0 (34.4 to 43.5)	**15.0 (0.55 to 28.91)**	122.4 (118.3 to 125.9)	106.9 (94.0 to 119.3)	**-12.6 (-23.81 to -1.91)**	20.0 (16.4 to 24.5)	20.8 (16.4 to 24.9)	4.5 (-12.67 to 22.96)	60.7 (49.4 to 72.9)	52.4 (42.0 to 62.6)	**-13.4 (-24.97 to -0.23)**
Bladder cancer	71.9 (60.1 to 80.7)	48.0 (41.4 to 55.5)	**-33.1 (-37.72 to -26.23)**	106.0 (88.2 to 118.8)	55.8 (48.3 to 64.6)	**-47.3 (-51.01 to -41.53)**	51.4 (41.1 to 54.4)	33.3 (27.6 to 38.3)	**-35.2 (-41.28 to -26.79)**	179.3 (143.8 to 188.8)	84.6 (70.9 to 97.5)	**-52.8 (-57.00 to -46.46)**	20.5 (14.5 to 27.8)	14.8 (10.2 to 20.8)	**-27.7 (-37.37 to -12.24)**	52.3 (37.4 to 70.9)	31.6 (21.8 to 45.0)	**-39.4 (-47.78 to -25.32)**
Brain and nervous system cancer	75.8 (64.7 to 87.8)	80.3 (68.1 to 93.8)	6.0 (-1.74 to 12.92)	144.2 (122.3 to 165.6)	125.2 (106.6 to 145.8)	**-13.2 (-19.09 to -7.48)**	44.4 (34.6 to 53.9)	48.8 (38.1 to 60.7)	**10.1 (0.16 to 20.51)**	174.2 (135.5 to 211.0)	154.3 (120.9 to 191.7)	**-11.4 (-19.16 to -3.02)**	31.4 (25.8 to 38.3)	31.5 (24.8 to 37.3)	0.2 (-8.60 to 10.04)	116.3 (93.9 to 140.3)	97.1 (77.6 to 116.4)	**-16.4 (-23.57 to -8.57)**
Thyroid cancer	6.0 (5.0 to 7.1)	4.8 (4.1 to 5.6)	**-19.5 (-32.36 to -5.02)**	9.5 (7.9 to 11.1)	6.2 (5.2 to 7.1)	**-34.1 (-44.61 to -22.98)**	2.1 (1.9 to 2.5)	2.2 (1.9 to 2.5)	5.5 (-24.59 to 20.71)	7.7 (7.0 to 8.9)	6.2 (5.1 to 7.0)	**-18.7 (-41.48 to -7.06)**	3.9 (3.0 to 4.8)	2.6 (1.8 to 3.3)	**-33.0 (-46.63 to -11.14)**	10.7 (8.2 to 13.1)	6.2 (4.4 to 7.8)	**-42.3 (-54.51 to -24.52)**
Mesothelioma	11.6 (9.7 to 20.5)	32.6 (17.8 to 39.2)	**199.6 (21.22 to 275.10)**	18.9 (15.9 to 33.0)	41.1 (23.1 to 49.2)	**129.9 (-2.59 to 186.56)**	8.3 (6.6 to 17.1)	27.2 (12.9 to 33.2)	**259.3 (19.43 to 363.86)**	29.3 (23.4 to 59.5)	71.7 (34.8 to 87.3)	**166.6 (-8.29 to 243.06)**	3.3 (2.9 to 5.2)	5.4 (3.8 to 7.6)	**66.8 (-0.59 to 101.19)**	10.0 (8.5 to 15.8)	13.5 (9.6 to 18.9)	36.3 (-16.09 to 64.55)
Hodgkin disease	12.5 (9.9 to 16.1)	8.5 (6.6 to 11.2)	**-31.9 (-41.88 to -15.59)**	24.1 (18.9 to 30.9)	14.4 (11.2 to 18.9)	**-39.9 (-48.46 to -26.38)**	7.5 (4.9 to 10.4)	4.8 (3.6 to 6.8)	**-35.1 (-45.96 to -15.32)**	29.8 (19.5 to 41.1)	16.6 (12.2 to 23.2)	**-43.7 (-53.07 to -27.23)**	5.0 (3.7 to 6.4)	3.6 (2.5 to 4.7)	**-26.8 (-40.59 to -8.72)**	18.6 (13.7 to 24.0)	12.3 (8.5 to 15.9)	**-34.0 (-46.08 to -17.60)**
Non-Hodgkin lymphoma	72.0 (59.6 to 100.7)	83.0 (54.7 to 103.2)	16.4 (-33.35 to 34.59)	121.3 (100.2 to 168.9)	112.6 (73.8 to 142.3)	-6.3 (-45.54 to 9.97)	41.4 (32.6 to 64.5)	49.0 (24.4 to 60.9)	20.5 (-43.74 to 43.45)	153.6 (120.4 to 238.8)	140.8 (70.5 to 176.7)	-6.9 (-56.03 to 11.85)	30.6 (22.9 to 45.7)	34.0 (20.1 to 47.2)	11.9 (-33.37 to 32.45)	93.0 (68.8 to 136.8)	86.5 (51.7 to 121.7)	-6.4 (-43.37 to 12.02)
Multiple myeloma	35.8 (26.0 to 49.4)	43.4 (32.9 to 59.8)	**21.4 (3.27 to 35.29)**	55.0 (39.8 to 75.4)	53.6 (40.7 to 74.0)	-2.2 (-17.58 to 9.57)	19.2 (12.0 to 29.7)	24.5 (16.1 to 37.6)	**29.0 (-5.62 to 54.61)**	67.5 (42.1 to 104.8)	64.7 (42.8 to 100.2)	-3.2 (-28.75 to 15.71)	16.7 (11.4 to 24.8)	18.9 (12.9 to 28.4)	**13.3 (0.18 to 28.17)**	45.2 (30.8 to 66.8)	43.8 (30.0 to 64.8)	-3.1 (-14.96 to 9.90)
Leukemia	80.4 (77.8 to 83.2)	74.2 (65.6 to 80.7)	**-7.6 (-18.94 to 0.73)**	149.9 (144.8 to 155.4)	111.9 (98.3 to 122.1)	**-25.3 (-34.78 to -18.30)**	45.7 (44.2 to 47.1)	45.3 (38.6 to 49.6)	-0.8 (-16.76 to 9.33)	182.5 (175.6 to 189.0)	140.7 (119.8 to 154.4)	**-22.9 (-34.93 to -15.00)**	34.7 (32.8 to 37.4)	29.0 (24.6 to 33.1)	**-16.4 (-36.27 to -3.93)**	122.7 (115.8 to 131.3)	85.5 (73.5 to 98.2)	**-30.2 (-46.43 to -19.46)**
Other neoplasms	87.5 (79.8 to 101.8)	97.8 (79.9 to 107.1)	12.1 (-7.85 to 23.67)	160.1 (146.2 to 185.7)	147.1 (120.7 to 161.6)	-7.9 (-23.88 to 1.62)	43.7 (38.4 to 55.6)	48.1 (35.3 to 54.6)	10.5 (-17.43 to 23.98)	173.6 (153.1 to 222.6)	152.3 (111.1 to 173.5)	**-11.9 (-34.68 to -0.94)**	43.7 (38.5 to 52.0)	49.7 (39.3 to 55.8)	13.9 (-4.64 to 29.00)	147.8 (130.6 to 173.8)	142.2 (113.9 to 159.8)	-3.6 (-16.91 to 8.66)
**Cardiovascular diseases**	3,444.7 (3,249.6 to 3,517.0)	1,754.8 (1,653.2 to 1,893.2)	**-49.0 (-51.88 to -45.10)**	5,134.9 (4,847.7 to 5,254.3)	2,029.9 (1,908.5 to 2,188.3)	**-60.5 (-62.67 to -57.62)**	1,995.3 (1,951.9 to 2,024.1)	984.9 (907.9 to 1,057.4)	**-50.6 (-54.44 to -47.02)**	7,092.3 (6,938.2 to 7,192.5)	2,584.7 (2,380.7 to 2,780.3)	**-63.6 (-66.32 to -60.77)**	1,449.5 (1,265.0 to 1,523.9)	770.0 (703.9 to 933.6)	**-46.8 (-51.86 to -35.83)**	3,498.6 (3,019.6 to 3,724.4)	1,511.7 (1,375.9 to 1,858.5)	**-56.7 (-60.97 to -47.72)**
Rheumatic heart disease	36.8 (25.6 to 44.4)	16.8 (13.9 to 26.4)	**-53.9 (-61.95 to -16.85)**	58.3 (41.1 to 70.5)	21.2 (17.7 to 33.0)	**-63.1 (-69.40 to -35.13)**	11.4 (7.9 to 15.4)	5.4 (3.8 to 8.3)	**-52.1 (-60.87 to -36.03)**	41.3 (28.7 to 55.8)	15.3 (10.7 to 23.3)	**-62.8 (-69.31 to -50.76)**	25.5 (15.3 to 31.4)	11.4 (8.9 to 20.7)	**-54.2 (-64.93 to 1.66)**	72.0 (43.4 to 89.2)	26.4 (20.8 to 47.8)	**-62.3 (-71.04 to -17.70)**
Ischemic heart disease	2,238.9 (2,033.5 to 2,323.4)	957.6 (884.7 to 1,099.0)	**-57.2 (-60.24 to -50.49)**	3,368.5 (3,058.5 to 3,492.9)	1,122.2 (1,035.1 to 1,289.3)	**-66.7 (-69.09 to -61.53)**	1,421.6 (1,315.4 to 1,479.1)	599.3 (545.5 to 664.4)	**-57.8 (-61.14 to -52.27)**	5,035.2 (4,650.3 to 5,237.2)	1,578.5 (1,434.4 to 1,750.4)	**-68.6 (-71.07 to -64.52)**	817.3 (666.9 to 882.9)	358.3 (316.4 to 483.3)	**-56.0 (-61.12 to -38.61)**	1,972.1 (1,596.1 to 2,141.1)	702.2 (620.0 to 959.7)	**-64.3 (-68.62 to -49.68)**
Cerebrovascular disease	768.7 (674.5 to 850.9)	450.7 (395.1 to 521.3)	**-41.3 (-46.06 to -34.02)**	1,104.1 (973.0 to 1,226.5)	497.4 (433.2 to 572.4)	**-54.9 (-59.01 to -49.67)**	338.1 (287.1 to 409.1)	206.1 (155.6 to 248.7)	**-38.9 (-55.34 to -29.38)**	1,211.3 (1,029.9 to 1,464.4)	525.6 (398.7 to 634.7)	**-56.5 (-68.28 to -49.81)**	430.6 (336.7 to 482.6)	244.7 (212.6 to 318.1)	**-43.0 (-49.16 to -26.47)**	1,015.5 (799.7 to 1,148.6)	467.6 (405.9 to 605.8)	**-53.8 (-58.69 to -40.57)**
Ischemic stroke	430.5 (354.7 to 477.4)	232.6 (198.7 to 287.6)	**-45.8 (-53.40 to -29.50)**	583.0 (481.3 to 647.1)	236.7 (200.6 to 292.3)	**-59.3 (-65.09 to -47.20)**	186.5 (149.7 to 225.7)	104.4 (74.2 to 133.2)	**-43.7 (-58.31 to -26.33)**	663.6 (534.6 to 801.5)	254.3 (178.7 to 324.5)	**-61.5 (-71.58 to -50.00)**	244.0 (175.9 to 269.8)	128.1 (109.1 to 174.1)	**-47.0 (-54.83 to -21.24)**	518.0 (375.0 to 576.2)	218.3 (185.7 to 300.1)	**-57.4 (-63.86 to -36.03)**
Hemorrhagic stroke	338.2 (300.5 to 406.0)	218.2 (170.3 to 250.8)	**-35.3 (-48.66 to -27.64)**	521.1 (463.6 to 624.5)	260.7 (205.9 to 298.3)	**-49.9 (-59.84 to -44.14)**	151.6 (123.8 to 192.7)	101.7 (67.0 to 129.6)	**-32.4 (-57.71 to -17.11)**	547.8 (446.3 to 696.6)	271.2 (183.6 to 343.7)	**-50.1 (-68.02 to -39.35)**	186.6 (147.8 to 232.2)	116.5 (90.0 to 148.8)	**-37.4 (-48.96 to -23.43)**	497.5 (397.9 to 623.9)	249.3 (195.3 to 318.2)	**-49.8 (-58.02 to -39.85)**
Hypertensive heart disease	38.3 (31.9 to 49.3)	31.7 (23.0 to 40.0)	**-16.5 (-40.54 to 1.34)**	58.1 (48.3 to 74.9)	38.0 (27.6 to 48.3)	**-34.0 (-52.17 to -19.64)**	20.6 (15.6 to 28.8)	16.9 (10.5 to 24.9)	-16.4 (-47.64 to 16.17)	73.2 (55.7 to 103.2)	45.4 (28.6 to 66.5)	**-36.9 (-59.77 to -13.10)**	17.7 (14.1 to 24.4)	14.8 (10.1 to 19.1)	-15.9 (-38.30 to 9.94)	44.5 (35.5 to 61.3)	30.5 (20.9 to 39.4)	**-30.9 (-49.08 to -9.23)**
Cardiomyopathy and myocarditis	78.3 (65.5 to 91.7)	39.1 (30.6 to 50.5)	**-50.0 (-60.47 to -39.10)**	131.8 (110.9 to 155.5)	54.0 (42.6 to 68.2)	**-59.0 (-68.32 to -51.09)**	42.6 (32.3 to 52.7)	24.4 (16.6 to 33.5)	**-42.5 (-59.19 to -25.42)**	166.0 (126.5 to 204.5)	74.7 (51.2 to 100.7)	**-54.8 (-67.77 to -41.91)**	35.7 (28.0 to 44.1)	14.7 (10.4 to 19.5)	**-58.5 (-71.91 to -39.20)**	96.3 (74.9 to 121.5)	33.3 (23.5 to 43.7)	**-65.3 (-77.12 to -50.72)**
Atrial fibrillation and flutter	14.9 (13.4 to 16.7)	32.5 (26.7 to 36.8)	**118.9 (82.39 to 144.46)**	19.7 (17.8 to 22.1)	31.9 (26.3 to 35.9)	**62.1 (36.96 to 80.34)**	5.4 (4.7 to 6.0)	11.6 (8.8 to 13.5)	**115.4 (65.89 to 146.51)**	19.3 (16.9 to 21.5)	27.9 (21.1 to 32.5)	**44.6 (11.52 to 65.92)**	9.5 (8.2 to 11.3)	20.9 (16.6 to 24.4)	**121.0 (83.38 to 155.66)**	19.5 (16.9 to 23.4)	34.2 (26.9 to 39.7)	**75.4 (47.38 to 102.21)**
Aortic aneurysm	117.6 (101.6 to 139.2)	78.8 (66.4 to 97.0)	**-33.0 (-40.75 to -25.24)**	168.8 (146.3 to 198.3)	89.7 (75.5 to 110.0)	**-46.8 (-53.03 to -40.66)**	81.7 (69.3 to 96.9)	49.2 (40.2 to 56.6)	**-39.7 (-49.58 to -32.54)**	281.4 (238.3 to 333.8)	125.3 (102.7 to 144.4)	**-55.4 (-62.55 to -49.90)**	35.9 (23.1 to 53.4)	29.6 (19.5 to 46.6)	**-17.3 (-32.86 to -1.01)**	84.2 (54.1 to 125.0)	58.6 (38.9 to 92.7)	**-30.2 (-43.12 to -15.65)**
Peripheral vascular disease	25.2 (23.2 to 27.8)	18.8 (16.6 to 21.5)	**-25.4 (-34.16 to -16.86)**	34.5 (31.8 to 38.1)	19.7 (17.4 to 22.4)	**-43.0 (-49.59 to -36.43)**	13.6 (12.4 to 15.2)	8.8 (7.3 to 10.2)	**-35.4 (-47.15 to -23.84)**	48.1 (43.6 to 54.0)	21.6 (18.0 to 25.1)	**-55.0 (-63.11 to -46.79)**	11.7 (10.1 to 13.9)	10.1 (8.3 to 11.9)	**-13.6 (-25.90 to -1.37)**	24.9 (21.6 to 29.9)	17.5 (14.4 to 21.0)	**-29.5 (-38.85 to -19.90)**
Endocarditis	10.2 (7.7 to 12.9)	9.7 (7.7 to 13.4)	-5.2 (-18.62 to 21.20)	16.6 (12.7 to 21.1)	12.9 (10.2 to 17.5)	**-22.6 (-33.37 to -3.24)**	5.2 (3.7 to 7.2)	6.0 (4.1 to 8.7)	16.9 (-11.84 to 52.16)	19.2 (13.7 to 26.7)	17.2 (11.9 to 24.4)	-10.0 (-31.68 to 13.64)	5.1 (3.2 to 7.0)	3.6 (2.6 to 6.1)	-27.4 (-42.67 to 8.65)	14.4 (9.1 to 19.9)	8.9 (6.2 to 15.1)	**-38.1 (-51.26 to -4.67)**
Other cardiovascular and circulatory diseases	115.7 (104.9 to 163.2)	119.1 (77.4 to 133.8)	4.3 (-45.60 to 17.41)	174.5 (158.2 to 245.9)	143.0 (92.9 to 160.3)	**-16.9 (-56.96 to -6.38)**	55.2 (50.4 to 85.0)	57.2 (31.0 to 65.0)	5.4 (-49.24 to 20.74)	197.2 (179.9 to 303.4)	153.2 (83.1 to 174.2)	**-21.0 (-61.99 to -9.53)**	60.5 (51.3 to 86.4)	61.8 (38.6 to 75.4)	3.5 (-43.39 to 20.64)	155.3 (130.2 to 225.0)	132.6 (81.9 to 161.9)	**-13.5 (-53.39 to 0.55)**
**Chronic respiratory diseases**	467.9 (418.4 to 507.3)	399.2 (359.9 to 451.6)	**-14.6 (-23.70 to -3.06)**	694.2 (622.2 to 756.5)	464.6 (417.9 to 520.1)	**-33.0 (-40.07 to -24.98)**	294.2 (240.5 to 320.5)	226.2 (195.4 to 286.5)	**-22.9 (-34.69 to -1.63)**	1,041.8 (850.3 to 1,135.9)	576.8 (498.1 to 724.5)	**-44.5 (-52.84 to -29.96)**	173.8 (163.2 to 205.9)	173.0 (135.1 to 192.6)	-0.2 (-27.33 to 10.53)	465.0 (436.4 to 541.7)	375.6 (297.0 to 418.2)	**-19.1 (-40.26 to -10.27)**
Chronic obstructive pulmonary disease	377.7 (335.4 to 410.1)	323.9 (288.9 to 379.3)	**-14.1 (-23.05 to 1.09)**	543.9 (483.6 to 591.8)	367.2 (327.3 to 426.3)	**-32.4 (-39.25 to -21.41)**	242.8 (195.4 to 263.5)	183.7 (157.8 to 241.4)	**-24.1 (-35.31 to 0.55)**	847.8 (681.0 to 917.2)	460.5 (396.2 to 601.0)	**-45.5 (-53.50 to -28.62)**	134.9 (119.6 to 161.5)	140.2 (114.0 to 161.3)	4.2 (-24.39 to 18.65)	345.6 (304.5 to 412.1)	293.2 (242.9 to 337.1)	**-14.9 (-37.47 to -3.45)**
Pneumoconiosis	7.2 (5.1 to 9.7)	7.0 (4.6 to 10.1)	-2.1 (-25.78 to 35.52)	10.3 (7.4 to 13.9)	7.8 (5.3 to 11.1)	**-24.0 (-41.48 to 1.84)**	6.6 (4.6 to 9.2)	6.7 (4.3 to 9.8)	0.9 (-24.86 to 41.00)	23.5 (16.2 to 32.7)	16.5 (10.7 to 23.9)	**-29.6 (-47.31 to -2.21)**	0.5 (0.4 to 0.7)	0.3 (0.2 to 0.4)	**-38.1 (-53.49 to -25.40)**	1.6 (1.2 to 2.2)	1.0 (0.7 to 1.3)	**-38.5 (-53.14 to -26.23)**
Silicosis	0.5 (0.4 to 0.7)	0.2 (0.2 to 0.3)	**-57.4 (-67.52 to -41.75)**	0.9 (0.6 to 1.2)	0.3 (0.2 to 0.4)	**-60.6 (-69.06 to -49.77)**	0.5 (0.3 to 0.7)	0.2 (0.1 to 0.3)	**-61.3 (-72.32 to -43.57)**	1.8 (1.2 to 2.5)	0.5 (0.4 to 0.8)	**-69.3 (-77.54 to -56.80)**	<0.05 (0.0 to 0.1)	<0.05 (0.0 to <0.05)	**-14.9 (-33.72 to 1.72)**	0.2 (0.1 to 0.2)	0.2 (0.1 to 0.2)	-10.8 (-31.15 to 6.87)
Asbestosis	1.5 (1.0 to 2.1)	3.2 (2.2 to 4.9)	**118.7 (67.43 to 228.18)**	2.2 (1.5 to 3.0)	3.6 (2.4 to 5.4)	**68.6 (31.56 to 147.59)**	1.4 (1.0 to 2.0)	3.2 (2.1 to 4.8)	**122.1 (68.90 to 236.11)**	5.1 (3.4 to 7.0)	7.9 (5.2 to 11.8)	**56.6 (19.21 to 135.32)**	0.1 (0.0 to 0.1)	0.1 (0.0 to 0.1)	28.5 (-11.36 to 58.57)	0.2 (0.2 to 0.3)	0.2 (0.2 to 0.3)	12.1 (-17.73 to 34.27)
Coal workers pneumoconiosis	2.6 (1.8 to 3.6)	2.3 (1.5 to 3.4)	-11.8 (-35.99 to 21.00)	3.7 (2.6 to 5.0)	2.5 (1.6 to 3.6)	**-32.2 (-50.10 to -10.09)**	2.6 (1.8 to 3.6)	2.3 (1.5 to 3.4)	-11.1 (-35.96 to 22.99)	9.1 (6.2 to 12.6)	5.6 (3.6 to 8.2)	**-38.7 (-55.46 to -16.48)**	0.1 (0.0 to 0.1)	<0.05 (0.0 to <0.05)	**-39.5 (-55.48 to -25.56)**	0.2 (0.2 to 0.3)	0.2 (0.1 to 0.2)	**-29.2 (-47.25 to -13.33)**

Other pneumoconiosis	2.5 (1.8 to 3.3)	1.2 (0.8 to 1.7)	**-51.6 (-64.46 to -39.07)**	3.6 (2.7 to 4.7)	1.4 (0.9 to 1.8)	**-62.1 (-71.80 to -53.59)**	2.1 (1.5 to 3.0)	1.0 (0.6 to 1.5)	**-51.8 (-66.57 to -37.62)**	7.6 (5.2 to 10.7)	2.5 (1.6 to 3.6)	**-66.4 (-76.52 to -57.10)**	0.4 (0.3 to 0.5)	0.2 (0.1 to 0.3)	**-50.1 (-63.91 to -38.74)**	1.0 (0.7 to 1.4)	0.4 (0.3 to 0.6)	**-55.4 (-66.43 to -45.78)**
Asthma	37.9 (30.4 to 44.4)	22.5 (17.5 to 29.4)	**-40.6 (-49.47 to -19.56)**	69.0 (55.8 to 79.7)	33.7 (27.4 to 42.3)	**-51.0 (-57.53 to -36.50)**	18.2 (14.6 to 20.2)	7.9 (6.7 to 9.6)	**-56.6 (-62.19 to -48.92)**	71.6 (57.3 to 79.7)	26.3 (22.4 to 31.8)	**-63.2 (-67.89 to -57.19)**	19.7 (13.6 to 26.2)	14.6 (10.0 to 21.7)	**-25.5 (-41.13 to 17.86)**	66.5 (47.6 to 86.5)	40.3 (29.1 to 57.2)	**-39.2 (-50.09 to -9.46)**
Interstitial lung disease and pulmonary sarcoidosis	20.8 (14.8 to 32.9)	43.1 (20.3 to 62.8)	133.5 (-18.50 to 271.59)	31.6 (22.6 to 49.6)	51.8 (24.7 to 74.8)	83.8 (-34.42 to 190.72)	14.0 (10.5 to 24.0)	26.4 (11.4 to 37.2)	112.9 (-26.45 to 245.63)	49.3 (37.1 to 84.3)	68.4 (29.8 to 96.1)	56.2 (-45.24 to 152.25)	6.8 (3.8 to 13.9)	16.7 (5.6 to 28.9)	188.2 (-12.48 to 381.21)	19.1 (10.9 to 38.2)	37.9 (13.5 to 64.4)	130.7 (-26.71 to 280.60)
Other chronic respiratory diseases	24.4 (20.6 to 29.2)	2.7 (2.2 to 3.2)	**-89.0 (-90.84 to -87.34)**	39.4 (33.8 to 46.3)	4.0 (3.4 to 4.8)	**-89.7 (-91.71 to -88.21)**	12.5 (10.5 to 14.6)	1.5 (1.3 to 1.9)	**-87.7 (-89.73 to -84.88)**	49.5 (42.0 to 57.8)	5.0 (4.1 to 6.2)	**-89.9 (-91.63 to -87.92)**	11.9 (8.4 to 16.4)	1.1 (0.8 to 1.6)	**-90.5 (-93.09 to -88.77)**	32.2 (24.0 to 44.1)	3.2 (2.3 to 4.3)	**-90.2 (-92.87 to -88.25)**
**Cirrhosis**	96.8 (94.1 to 99.6)	176.6 (164.6 to 188.4)	**82.4 (69.89 to 95.13)**	172.8 (168.0 to 177.6)	260.6 (243.0 to 278.3)	**50.8 (40.64 to 61.31)**	55.1 (53.6 to 56.4)	114.1 (104.4 to 123.2)	**106.9 (89.83 to 124.90)**	208.1 (202.2 to 213.0)	345.2 (316.6 to 373.6)	**65.9 (51.95 to 80.56)**	41.7 (39.8 to 44.6)	62.5 (56.5 to 68.0)	**50.0 (35.57 to 64.52)**	139.8 (133.1 to 148.6)	178.7 (161.2 to 195.1)	**27.9 (15.84 to 40.07)**
Cirrhosis due to hepatitis B	10.2 (8.7 to 12.3)	18.3 (14.7 to 22.0)	**80.9 (38.83 to 123.81)**	18.0 (15.4 to 21.8)	26.7 (21.2 to 31.9)	**48.7 (13.56 to 83.76)**	5.7 (4.6 to 7.0)	10.1 (8.0 to 12.8)	**76.8 (33.30 to 123.34)**	21.5 (17.4 to 26.2)	30.3 (24.0 to 38.6)	**41.8 (7.11 to 78.87)**	4.5 (3.4 to 6.1)	8.3 (6.0 to 10.6)	**88.6 (23.02 to 166.47)**	14.9 (11.1 to 20.5)	23.1 (16.5 to 29.8)	**58.3 (2.74 to 125.00)**
Cirrhosis due to hepatitis C	36.7 (32.6 to 41.2)	82.9 (73.7 to 92.9)	**126.7 (94.40 to 161.17)**	65.9 (58.2 to 74.4)	122.3 (108.8 to 137.1)	**86.1 (59.31 to 114.72)**	18.7 (15.0 to 22.4)	48.2 (40.9 to 55.8)	**159.3 (105.55 to 221.20)**	70.9 (56.6 to 85.1)	145.9 (123.9 to 168.2)	**107.6 (64.82 to 157.11)**	17.9 (14.9 to 21.2)	34.8 (29.6 to 40.3)	**95.5 (50.00 to 141.77)**	61.2 (50.8 to 73.0)	99.3 (84.2 to 115.9)	**63.5 (24.33 to 101.95)**
Cirrhosis due to alcohol use	40.4 (35.7 to 44.8)	51.9 (43.4 to 59.7)	**28.7 (7.79 to 52.50)**	70.6 (61.6 to 78.7)	74.6 (62.0 to 86.0)	5.9 (-11.36 to 26.13)	25.6 (21.3 to 28.9)	42.1 (34.7 to 48.9)	**65.4 (34.81 to 105.73)**	95.6 (79.6 to 108.1)	124.8 (102.7 to 145.1)	**31.1 (6.71 to 63.14)**	14.9 (12.5 to 17.4)	9.8 (7.3 to 12.5)	**-33.6 (-51.21 to -15.86)**	47.1 (38.6 to 55.6)	26.3 (19.3 to 33.8)	**-43.9 (-60.05 to -27.93)**
Cirrhosis due to other causes	9.6 (8.3 to 11.3)	23.4 (20.1 to 26.9)	**146.4 (95.65 to 195.92)**	18.3 (16.0 to 21.5)	37.1 (32.0 to 42.4)	**103.5 (62.69 to 141.96)**	5.1 (4.0 to 6.5)	13.7 (10.8 to 17.2)	**173.4 (101.48 to 287.12)**	20.1 (15.8 to 25.6)	44.2 (35.3 to 55.0)	**123.6 (66.40 to 212.56)**	4.5 (3.4 to 5.7)	9.7 (7.6 to 12.1)	**121.0 (49.95 to 192.84)**	16.6 (12.6 to 21.0)	30.0 (23.8 to 37.3)	**83.5 (25.99 to 139.97)**
**Digestive diseases**	188.6 (181.4 to 215.4)	188.0 (168.3 to 223.8)	-0.3 (-13.73 to 13.96)	287.4 (276.8 to 328.6)	223.7 (199.8 to 260.8)	**-22.1 (-32.75 to -13.34)**	88.4 (81.4 to 102.1)	91.3 (79.9 to 117.0)	3.4 (-10.52 to 29.46)	322.8 (298.5 to 372.9)	244.7 (215.2 to 307.4)	**-24.1 (-35.32 to -7.81)**	100.2 (93.9 to 121.5)	96.7 (82.3 to 115.4)	-3.3 (-21.45 to 7.76)	256.5 (241.6 to 310.8)	202.8 (170.5 to 236.5)	**-20.7 (-36.58 to -12.27)**
Peptic ulcer disease	58.4 (49.4 to 68.4)	35.6 (28.3 to 47.9)	**-38.9 (-50.59 to -17.47)**	87.1 (74.3 to 101.9)	41.4 (33.0 to 54.2)	**-52.4 (-61.57 to -38.11)**	30.5 (25.1 to 36.4)	20.2 (14.7 to 30.2)	**-33.4 (-51.54 to 4.33)**	109.5 (90.0 to 130.2)	52.7 (38.3 to 77.0)	**-51.6 (-64.71 to -26.48)**	27.8 (21.6 to 36.0)	15.4 (11.4 to 21.9)	**-44.5 (-56.72 to -20.79)**	69.0 (54.2 to 89.6)	31.3 (23.0 to 43.0)	**-54.4 (-64.97 to -38.66)**
Gastritis and duodenitis	2.1 (1.5 to 3.4)	3.1 (1.8 to 4.4)	**54.7 (1.12 to 104.32)**	3.5 (2.6 to 5.7)	4.2 (2.4 to 5.9)	22.7 (-19.45 to 61.56)	1.1 (0.7 to 2.0)	1.9 (0.9 to 2.8)	**74.2 (1.86 to 150.54)**	4.3 (2.7 to 7.7)	5.6 (2.6 to 8.0)	33.7 (-20.59 to 89.99)	0.9 (0.5 to 1.8)	1.2 (0.6 to 2.1)	31.9 (-11.13 to 67.65)	2.8 (1.5 to 5.5)	2.9 (1.4 to 4.9)	7.3 (-27.71 to 37.06)
Appendicitis	5.1 (3.8 to 6.1)	4.1 (2.8 to 6.1)	-19.5 (-47.91 to 26.40)	9.3 (7.0 to 11.1)	5.7 (4.1 to 8.3)	**-38.4 (-57.75 to -9.31)**	2.5 (1.7 to 3.2)	2.4 (1.4 to 4.1)	-2.6 (-47.88 to 55.99)	9.9 (6.7 to 12.8)	6.9 (4.2 to 11.5)	-28.7 (-59.45 to 9.65)	2.6 (1.8 to 3.4)	1.7 (1.1 to 2.9)	-34.6 (-55.88 to 5.92)	8.8 (6.2 to 11.1)	4.6 (3.1 to 7.4)	**-47.2 (-62.84 to -19.68)**
Paralytic ileus and intestinal obstruction	16.4 (12.1 to 24.8)	23.6 (12.7 to 30.4)	46.3 (-9.36 to 79.49)	24.8 (18.2 to 37.2)	27.9 (15.0 to 35.5)	14.3 (-28.34 to 40.04)	7.2 (4.7 to 12.5)	11.2 (4.6 to 14.8)	59.1 (-22.32 to 115.29)	27.1 (17.6 to 46.6)	30.0 (12.4 to 39.4)	13.6 (-42.68 to 52.60)	9.1 (5.2 to 15.4)	12.4 (6.1 to 18.4)	37.2 (-5.13 to 64.14)	23.0 (13.1 to 38.9)	25.9 (13.0 to 37.9)	14.0 (-21.31 to 35.17)
Inguinal, femoral, and abdominal hernia	9.1 (6.6 to 13.1)	8.6 (3.9 to 21.5)	-3.3 (-54.87 to 185.27)	13.4 (9.7 to 19.3)	9.5 (4.4 to 22.9)	-27.1 (-64.98 to 104.61)	4.3 (2.5 to 6.5)	4.4 (1.7 to 14.2)	9.7 (-59.59 to 309.13)	15.7 (9.1 to 23.6)	11.2 (4.4 to 34.6)	-24.5 (-71.37 to 171.37)	4.8 (2.8 to 8.4)	4.1 (1.7 to 9.9)	-9.6 (-65.73 to 120.02)	11.7 (6.9 to 20.3)	8.2 (3.4 to 19.1)	-27.0 (-71.19 to 69.71)
Inflammatory bowel disease	13.5 (11.0 to 18.3)	14.2 (11.4 to 19.1)	5.9 (-13.64 to 29.79)	21.8 (17.8 to 29.5)	18.1 (14.4 to 23.7)	**-16.9 (-32.67 to 1.06)**	5.3 (4.1 to 7.4)	6.1 (4.2 to 9.0)	16.2 (-26.88 to 69.21)	20.2 (15.8 to 27.9)	17.3 (11.9 to 25.4)	-13.9 (-43.50 to 24.19)	8.2 (5.9 to 12.1)	8.1 (5.9 to 12.4)	-0.3 (-17.45 to 19.55)	23.0 (16.6 to 33.7)	18.6 (13.6 to 27.9)	**-18.6 (-32.94 to -3.76)**
Vascular intestinal disorders	22.8 (14.7 to 35.0)	28.7 (17.5 to 44.1)	27.5 (-11.41 to 78.64)	33.6 (21.7 to 51.7)	33.1 (20.5 to 50.8)	-0.1 (-29.82 to 37.56)	10.4 (4.6 to 20.1)	12.0 (4.7 to 24.2)	18.5 (-23.31 to 133.96)	37.0 (16.4 to 71.1)	31.1 (12.2 to 62.3)	-13.4 (-43.30 to 65.27)	12.4 (7.4 to 23.3)	16.7 (7.1 to 26.3)	37.8 (-16.94 to 72.90)	30.8 (18.6 to 57.6)	34.5 (15.0 to 55.8)	14.7 (-30.62 to 43.03)
Gallbladder and biliary diseases	11.9 (10.0 to 15.9)	17.9 (12.5 to 21.4)	**52.0 (-3.47 to 91.66)**	17.5 (14.7 to 23.5)	20.1 (14.1 to 23.9)	16.2 (-26.46 to 46.05)	5.1 (4.1 to 6.9)	8.3 (4.8 to 10.3)	64.2 (-21.85 to 113.39)	18.4 (14.8 to 24.9)	21.1 (12.5 to 26.2)	16.4 (-43.62 to 50.43)	6.8 (5.4 to 10.0)	9.7 (5.9 to 12.0)	43.6 (-15.35 to 86.98)	16.9 (13.5 to 24.8)	19.3 (11.7 to 24.0)	15.3 (-33.73 to 50.11)
Pancreatitis	16.5 (12.9 to 21.4)	18.2 (13.3 to 23.8)	10.3 (-13.48 to 34.96)	27.6 (21.7 to 35.5)	24.6 (18.0 to 31.6)	-10.7 (-29.47 to 7.65)	9.2 (6.5 to 12.8)	11.0 (7.1 to 14.7)	20.6 (-9.34 to 57.23)	34.1 (24.3 to 47.5)	32.2 (21.0 to 42.6)	-5.3 (-27.72 to 20.43)	7.4 (5.2 to 10.6)	7.2 (4.6 to 10.7)	-1.9 (-27.74 to 35.83)	21.4 (15.1 to 30.5)	17.3 (11.3 to 25.0)	-18.7 (-39.96 to 8.14)
Other digestive diseases	32.9 (27.9 to 39.2)	34.0 (27.7 to 41.0)	3.8 (-16.32 to 24.57)	48.8 (41.4 to 58.5)	39.1 (31.9 to 46.8)	**-19.6 (-35.82 to -3.48)**	12.7 (10.3 to 16.6)	13.8 (9.4 to 18.2)	8.8 (-29.23 to 40.99)	46.6 (37.6 to 60.7)	36.6 (24.9 to 47.8)	**-20.9 (-47.83 to 2.12)**	20.1 (16.4 to 23.8)	20.2 (16.0 to 24.9)	0.9 (-17.13 to 21.67)	49.2 (40.1 to 58.4)	40.3 (31.3 to 48.8)	**-17.9 (-33.74 to -1.05)**
**Neurological disorders**	381.4 (357.6 to 412.1)	454.2 (422.1 to 486.3)	**19.2 (8.65 to 28.53)**	567.0 (532.9 to 612.5)	511.6 (473.2 to 548.5)	**-9.7 (-17.12 to -2.96)**	176.8 (162.5 to 190.8)	228.1 (207.9 to 246.8)	**29.1 (16.87 to 42.26)**	686.0 (630.1 to 743.5)	597.6 (546.6 to 650.3)	**-12.8 (-20.63 to -3.98)**	204.6 (186.1 to 228.7)	226.1 (202.1 to 248.4)	10.7 (-3.67 to 24.97)	490.5 (452.4 to 542.6)	441.5 (396.0 to 484.0)	**-9.9 (-20.32 to -0.11)**
Alzheimer disease and other dementias	232.5 (211.4 to 253.8)	303.1 (276.9 to 327.0)	**30.6 (15.50 to 44.80)**	300.0 (273.1 to 326.8)	289.3 (265.3 to 311.1)	-3.4 (-14.31 to 6.87)	95.0 (84.9 to 104.8)	142.0 (126.6 to 156.5)	**50.0 (30.44 to 75.10)**	362.6 (323.5 to 401.0)	331.6 (297.0 to 365.4)	-8.3 (-20.16 to 6.84)	137.5 (119.0 to 157.0)	161.1 (141.5 to 180.2)	**17.6 (-1.76 to 40.41)**	268.0 (233.1 to 304.8)	258.4 (227.6 to 287.5)	-3.2 (-19.31 to 14.83)
Parkinson disease	37.9 (31.2 to 41.9)	40.7 (33.7 to 44.9)	7.4 (-5.02 to 17.82)	50.1 (41.4 to 55.6)	42.5 (35.4 to 46.8)	**-15.1 (-25.31 to -6.77)**	21.6 (18.3 to 22.5)	25.6 (21.6 to 28.7)	**18.9 (3.28 to 33.38)**	76.5 (65.1 to 80.1)	61.7 (51.9 to 69.0)	**-19.1 (-29.88 to -9.40)**	16.3 (11.4 to 18.9)	15.1 (10.2 to 18.6)	-7.6 (-21.27 to 7.08)	34.6 (24.5 to 40.3)	27.5 (18.9 to 33.9)	**-20.3 (-32.03 to -7.16)**
Epilepsy	32.4 (29.2 to 39.5)	27.4 (22.5 to 32.8)	**-15.2 (-29.54 to -1.10)**	68.1 (61.6 to 83.0)	51.1 (42.3 to 61.7)	**-24.8 (-37.23 to -12.53)**	19.7 (16.4 to 25.0)	16.6 (13.0 to 21.6)	**-15.6 (-30.50 to 3.14)**	83.5 (69.8 to 106.2)	62.1 (49.2 to 81.2)	**-25.4 (-37.93 to -9.11)**	12.7 (10.7 to 16.2)	10.9 (8.0 to 13.3)	**-14.2 (-38.23 to 0.06)**	52.8 (44.4 to 67.6)	40.2 (30.0 to 49.5)	**-23.6 (-43.96 to -11.09)**
Multiple sclerosis	21.2 (14.9 to 32.9)	23.2 (16.1 to 33.3)	10.2 (-24.68 to 31.76)	38.5 (26.8 to 59.8)	34.2 (23.7 to 49.7)	-10.5 (-38.34 to 6.95)	8.0 (4.4 to 14.2)	8.5 (3.8 to 14.7)	7.9 (-48.89 to 36.09)	30.3 (16.5 to 53.5)	25.9 (11.4 to 44.5)	-13.4 (-58.37 to 9.05)	13.2 (8.5 to 20.6)	14.7 (8.6 to 22.6)	12.0 (-31.78 to 35.60)	46.3 (29.9 to 72.1)	42.1 (25.1 to 64.9)	-8.4 (-42.85 to 11.28)
Other neurological disorders	57.4 (50.3 to 63.6)	59.8 (51.1 to 66.1)	4.2 (-4.43 to 11.83)	110.3 (96.5 to 122.6)	94.5 (81.5 to 106.0)	**-14.3 (-22.36 to -7.31)**	32.5 (24.6 to 38.2)	35.3 (27.8 to 42.5)	8.8 (-5.69 to 19.31)	133.0 (100.7 to 157.4)	116.2 (92.4 to 142.0)	**-12.6 (-24.80 to -2.81)**	24.9 (23.9 to 26.7)	24.4 (22.1 to 26.7)	-1.7 (-12.37 to 8.70)	88.9 (84.6 to 96.4)	73.3 (65.8 to 80.7)	**-17.4 (-27.29 to -7.49)**
**Mental and substance use disorders**	49.4 (44.9 to 68.3)	94.7 (73.2 to 105.6)	**94.0 (8.92 to 121.30)**	99.5 (90.7 to 138.6)	176.4 (136.7 to 196.1)	**79.5 (-0.38 to 104.99)**	34.2 (30.9 to 51.7)	72.7 (48.4 to 82.0)	**117.2 (-11.76 to 155.28)**	139.4 (125.8 to 209.8)	273.8 (185.8 to 309.2)	**100.7 (-17.66 to 135.81)**	15.2 (12.5 to 20.4)	22.0 (18.4 to 28.8)	**45.0 (25.16 to 66.91)**	59.6 (49.4 to 78.9)	78.7 (67.1 to 100.4)	**32.6 (13.99 to 52.86)**
Schizophrenia	1.7 (1.2 to 2.0)	0.9 (0.7 to 1.3)	**-47.1 (-60.12 to -9.72)**	2.7 (1.8 to 3.1)	1.1 (0.9 to 1.6)	**-57.2 (-67.99 to -26.79)**	0.8 (0.4 to 1.0)	0.4 (0.3 to 0.7)	**-48.4 (-68.38 to 7.57)**	3.1 (1.6 to 3.7)	1.1 (0.8 to 2.0)	**-60.3 (-75.60 to -17.24)**	0.9 (0.6 to 1.0)	0.5 (0.4 to 0.7)	**-44.8 (-56.81 to -16.89)**	2.3 (1.6 to 2.6)	1.1 (0.8 to 1.4)	**-53.2 (-62.44 to -31.56)**
Alcohol use disorders	14.3 (11.1 to 23.4)	21.3 (11.5 to 27.9)	52.9 (-21.79 to 82.37)	27.6 (21.4 to 45.1)	34.4 (18.7 to 44.9)	28.1 (-34.48 to 52.47)	9.0 (7.6 to 16.9)	14.2 (5.9 to 17.3)	63.7 (-34.63 to 101.11)	35.5 (29.8 to 66.6)	46.7 (19.3 to 56.8)	36.9 (-45.00 to 68.41)	5.3 (2.7 to 10.5)	7.1 (3.2 to 13.3)	36.5 (-5.33 to 78.11)	19.9 (10.3 to 39.1)	22.4 (10.1 to 41.8)	14.2 (-20.88 to 48.35)
Drug use disorders	33.0 (30.2 to 45.6)	72.1 (55.5 to 80.0)	**121.9 (10.72 to 153.22)**	68.5 (62.7 to 94.8)	140.2 (108.5 to 155.6)	**107.7 (4.07 to 137.47)**	24.2 (21.7 to 36.9)	58.0 (40.3 to 65.8)	**145.0 (-7.87 to 190.92)**	100.6 (89.9 to 153.0)	225.7 (157.2 to 256.2)	**129.8 (-11.89 to 172.98)**	8.7 (8.1 to 9.4)	14.1 (12.2 to 16.6)	**61.9 (37.79 to 94.56)**	36.1 (33.3 to 38.7)	54.2 (46.8 to 63.9)	**50.3 (27.43 to 80.55)**
Opioid use disorders	11.4 (10.2 to 15.9)	26.8 (20.0 to 30.3)	**140.0 (18.13 to 181.34)**	23.6 (21.1 to 32.9)	52.4 (39.3 to 59.0)	**125.6 (12.43 to 164.19)**	8.5 (7.3 to 13.0)	21.8 (14.9 to 25.2)	**163.4 (-0.07 to 222.33)**	35.2 (30.4 to 53.8)	85.2 (58.7 to 98.5)	**148.2 (-4.30 to 202.54)**	2.9 (2.6 to 3.2)	5.0 (4.2 to 6.0)	**75.6 (41.10 to 115.67)**	11.8 (10.6 to 13.1)	19.3 (16.2 to 23.1)	**63.7 (31.31 to 101.87)**
Cocaine use disorders	1.4 (1.2 to 1.8)	2.2 (1.7 to 2.6)	**62.1 (-12.57 to 101.94)**	2.8 (2.4 to 3.8)	4.3 (3.3 to 5.1)	**52.6 (-16.64 to 89.45)**	0.9 (0.7 to 1.3)	1.6 (1.1 to 1.9)	**85.3 (-28.56 to 150.85)**	3.7 (2.9 to 5.5)	6.2 (4.2 to 7.6)	**74.9 (-31.25 to 137.09)**	0.5 (0.4 to 0.6)	0.6 (0.5 to 0.7)	23.5 (-4.01 to 58.32)	2.0 (1.7 to 2.3)	2.3 (1.9 to 2.8)	15.7 (-9.88 to 49.49)
Amphetamine use disorders	1.2 (1.0 to 1.6)	2.0 (1.6 to 2.4)	**75.4 (-4.64 to 114.64)**	2.5 (2.1 to 3.2)	4.0 (3.1 to 4.7)	**64.5 (-9.76 to 102.93)**	0.7 (0.6 to 1.1)	1.4 (1.0 to 1.8)	**101.5 (-25.62 to 163.85)**	3.1 (2.5 to 4.7)	5.7 (3.9 to 7.0)	**89.5 (-28.16 to 149.65)**	0.4 (0.4 to 0.5)	0.6 (0.5 to 0.7)	**35.7 (3.46 to 78.73)**	1.8 (1.6 to 2.1)	2.3 (1.9 to 2.9)	26.6 (-4.16 to 67.60)
Other drug use disorders	19.1 (17.3 to 26.2)	41.1 (31.0 to 46.0)	**118.5 (9.52 to 152.99)**	39.6 (35.9 to 54.0)	79.5 (60.7 to 89.2)	**103.9 (3.24 to 135.91)**	14.1 (12.5 to 21.3)	33.1 (22.7 to 37.9)	**140.5 (-7.70 to 189.40)**	58.6 (51.8 to 88.2)	128.6 (89.1 to 147.3)	**124.9 (-13.18 to 170.54)**	5.0 (4.5 to 5.4)	7.9 (6.8 to 9.4)	**60.3 (35.04 to 92.92)**	20.4 (18.7 to 22.1)	30.2 (25.9 to 35.9)	**48.3 (24.52 to 79.00)**
Eating disorders	0.4 (0.3 to 0.4)	0.3 (0.3 to 0.4)	-3.3 (-22.39 to 20.76)	0.8 (0.7 to 0.9)	0.7 (0.6 to 0.8)	-10.6 (-28.13 to 9.95)	0.1 (0.1 to 0.1)	0.1 (0.1 to 0.1)	-9.5 (-30.85 to 18.65)	0.3 (0.2 to 0.4)	0.3 (0.2 to 0.3)	-15.1 (-34.91 to 10.40)	0.3 (0.2 to 0.3)	0.3 (0.2 to 0.3)	-1.4 (-24.24 to 27.54)	1.2 (1.0 to 1.5)	1.1 (0.9 to 1.3)	-9.4 (-29.54 to 16.85)
Anorexia nervosa	0.4 (0.3 to 0.4)	0.3 (0.3 to 0.4)	-3.3 (-22.39 to 20.76)	0.8 (0.7 to 0.9)	0.7 (0.6 to 0.8)	-10.6 (-28.13 to 9.95)	0.1 (0.1 to 0.1)	0.1 (0.1 to 0.1)	-9.5 (-30.85 to 18.65)	0.3 (0.2 to 0.4)	0.3 (0.2 to 0.3)	-15.1 (-34.91 to 10.40)	0.3 (0.2 to 0.3)	0.3 (0.2 to 0.3)	-1.4 (-24.24 to 27.54)	1.2 (1.0 to 1.5)	1.1 (0.9 to 1.3)	-9.4 (-29.54 to 16.85)
**Diabetes, urogenital, blood, and endocrine diseases**	263.4 (246.6 to 282.9)	209.2 (181.9 to 224.4)	**-20.5 (-30.83 to -14.49)**	427.4 (397.6 to 455.9)	269.7 (237.8 to 289.9)	**-36.9 (-44.00 to -32.42)**	130.2 (120.4 to 137.4)	101.4 (90.5 to 110.4)	**-22.1 (-28.99 to -16.04)**	492.8 (453.4 to 519.8)	289.9 (261.1 to 317.4)	**-41.2 (-46.30 to -36.21)**	133.2 (121.8 to 150.4)	107.8 (86.8 to 119.5)	**-18.9 (-35.98 to -9.16)**	378.9 (344.6 to 423.2)	251.3 (206.6 to 279.0)	**-33.6 (-45.13 to -26.15)**
Diabetes mellitus	108.9 (101.8 to 113.9)	59.7 (55.7 to 63.3)	**-45.2 (-48.76 to -41.57)**	165.9 (156.1 to 173.3)	73.4 (68.5 to 77.8)	**-55.8 (-58.66 to -52.73)**	54.0 (52.6 to 55.2)	31.6 (29.4 to 33.8)	**-41.5 (-45.50 to -37.13)**	194.4 (189.3 to 198.9)	86.0 (80.0 to 92.2)	**-55.7 (-58.82 to -52.36)**	54.9 (49.4 to 60.2)	28.1 (25.1 to 30.8)	**-48.7 (-54.44 to -43.29)**	143.8 (131.2 to 157.0)	61.7 (55.4 to 67.8)	**-57.1 (-61.51 to -52.52)**
Acute glomerulonephritis	0.4 (0.4 to 0.5)	0.3 (0.2 to 0.3)	**-30.6 (-51.40 to -21.72)**	0.8 (0.7 to 1.0)	0.6 (0.4 to 0.6)	**-34.6 (-58.18 to -23.49)**	0.2 (0.2 to 0.3)	0.2 (0.1 to 0.2)	**-28.5 (-50.11 to -9.97)**	0.9 (0.7 to 1.3)	0.6 (0.4 to 0.7)	**-36.2 (-58.95 to -23.61)**	0.2 (0.2 to 0.3)	0.1 (0.1 to 0.2)	**-32.7 (-54.77 to -19.05)**	0.8 (0.6 to 1.0)	0.5 (0.3 to 0.6)	**-33.7 (-60.01 to -19.85)**
Chronic kidney disease	62.3 (53.4 to 66.3)	48.0 (42.8 to 56.2)	**-22.8 (-31.36 to -3.17)**	94.2 (80.9 to 100.1)	56.3 (50.1 to 66.3)	**-40.1 (-46.86 to -24.22)**	32.5 (26.3 to 33.9)	24.6 (21.8 to 29.6)	**-24.3 (-32.79 to -6.84)**	119.1 (96.8 to 124.2)	64.8 (57.6 to 78.2)	**-45.5 (-51.65 to -33.26)**	29.8 (23.3 to 33.1)	23.4 (19.6 to 28.8)	**-21.1 (-32.86 to 10.40)**	77.0 (59.9 to 85.3)	49.3 (41.7 to 61.3)	**-35.8 (-45.19 to -10.37)**
Chronic kidney disease due to diabetes mellitus	12.6 (8.7 to 14.9)	8.3 (7.0 to 12.6)	**-33.8 (-42.70 to -3.28)**	18.7 (12.9 to 22.0)	9.9 (8.3 to 15.0)	**-46.8 (-53.76 to -23.23)**	6.7 (3.7 to 8.4)	4.4 (3.4 to 8.2)	-32.9 (-48.26 to 14.70)	24.2 (13.3 to 30.3)	11.6 (8.9 to 21.5)	**-51.6 (-62.50 to -16.47)**	6.0 (4.2 to 7.1)	3.9 (3.1 to 5.0)	**-34.2 (-44.47 to -12.54)**	15.2 (11.1 to 17.9)	8.5 (6.8 to 10.8)	**-43.7 (-51.93 to -26.03)**
Chronic kidney disease due to hypertension	16.5 (12.5 to 21.5)	12.7 (9.0 to 16.0)	**-23.0 (-38.38 to -13.43)**	24.3 (18.4 to 31.4)	14.4 (10.2 to 18.2)	**-40.6 (-52.44 to -33.45)**	8.2 (5.4 to 11.6)	6.6 (3.5 to 8.8)	**-20.0 (-46.15 to -4.28)**	29.7 (19.6 to 42.0)	17.1 (9.1 to 23.0)	**-42.5 (-61.35 to -30.78)**	8.3 (5.3 to 12.3)	6.1 (3.4 to 8.8)	**-26.0 (-47.28 to -8.77)**	20.2 (12.8 to 29.9)	12.1 (6.6 to 17.4)	**-39.9 (-56.95 to -25.88)**
Chronic kidney disease due to glomerulonephritis	5.9 (4.3 to 6.7)	3.4 (2.2 to 4.1)	**-42.4 (-59.38 to -28.21)**	10.0 (7.5 to 11.5)	4.4 (2.9 to 5.3)	**-56.1 (-67.19 to -46.38)**	3.3 (2.3 to 4.0)	1.8 (1.1 to 2.4)	**-46.6 (-63.27 to -26.93)**	12.7 (8.8 to 15.1)	5.0 (3.2 to 6.7)	**-60.1 (-71.34 to -48.23)**	2.5 (1.7 to 3.1)	1.6 (0.9 to 2.2)	**-36.3 (-60.02 to -16.53)**	7.9 (5.3 to 9.6)	3.9 (2.2 to 5.1)	**-50.8 (-67.69 to -36.20)**
Chronic kidney disease due to other causes	27.2 (21.5 to 30.8)	23.6 (19.1 to 30.0)	-13.1 (-26.26 to 16.63)	41.2 (32.9 to 46.5)	27.6 (22.7 to 35.3)	**-32.8 (-43.12 to -8.83)**	14.3 (10.2 to 17.3)	11.8 (9.3 to 15.8)	**-17.1 (-32.42 to 3.26)**	52.5 (37.9 to 63.9)	31.2 (24.8 to 41.9)	**-40.4 (-51.21 to -25.68)**	13.0 (8.2 to 15.8)	11.8 (8.7 to 16.2)	-8.3 (-27.50 to 46.68)	33.8 (22.0 to 40.9)	24.8 (18.2 to 33.7)	**-25.9 (-41.20 to 19.77)**
Urinary diseases and male infertility	33.9 (30.2 to 48.4)	59.5 (34.2 to 69.9)	83.0 (-18.47 to 128.93)	50.4 (45.0 to 71.7)	66.4 (38.8 to 78.0)	37.0 (-37.67 to 70.38)	13.9 (12.5 to 21.5)	24.1 (12.0 to 29.6)	80.1 (-27.34 to 131.28)	52.1 (46.9 to 80.8)	62.0 (31.3 to 76.3)	23.5 (-49.61 to 58.57)	19.9 (17.3 to 30.1)	35.4 (19.1 to 43.7)	86.9 (-16.47 to 149.16)	50.2 (43.9 to 73.7)	70.3 (39.3 to 86.9)	47.0 (-31.27 to 95.32)
Interstitial nephritis and urinary tract infections	27.2 (23.7 to 40.0)	52.8 (30.2 to 62.5)	104.0 (-12.07 to 158.36)	39.7 (34.7 to 58.2)	58.0 (33.5 to 68.4)	53.1 (-32.87 to 93.29)	10.0 (8.7 to 16.1)	20.5 (10.3 to 25.1)	114.6 (-17.99 to 172.31)	37.6 (32.5 to 60.3)	52.1 (26.3 to 63.4)	45.4 (-44.07 to 84.48)	17.2 (14.7 to 26.2)	32.3 (17.2 to 39.9)	99.5 (-13.44 to 162.39)	42.1 (36.4 to 62.7)	63.2 (34.6 to 77.8)	58.3 (-28.05 to 107.63)
Urolithiasis	2.3 (1.6 to 3.5)	2.0 (1.2 to 3.8)	-8.6 (-56.44 to 101.29)	3.7 (2.6 to 5.5)	2.6 (1.5 to 4.9)	-26.1 (-64.11 to 59.79)	0.9 (0.6 to 1.4)	0.9 (0.3 to 1.8)	9.1 (-67.36 to 150.94)	3.3 (2.1 to 5.3)	2.5 (0.9 to 5.0)	-17.7 (-74.55 to 85.57)	1.4 (0.9 to 2.2)	1.1 (0.6 to 2.2)	-17.7 (-53.62 to 76.34)	4.1 (2.7 to 6.3)	2.7 (1.5 to 5.2)	-30.7 (-60.70 to 45.13)
Other urinary diseases	4.3 (2.8 to 6.3)	4.6 (2.3 to 7.5)	11.3 (-51.60 to 103.81)	7.0 (4.6 to 10.2)	5.8 (2.9 to 9.1)	-14.0 (-61.05 to 51.40)	3.0 (1.6 to 4.5)	2.7 (1.0 to 5.2)	-2.2 (-65.00 to 98.74)	11.2 (6.2 to 17.1)	7.4 (3.0 to 14.0)	-29.1 (-73.31 to 41.89)	1.4 (0.9 to 2.2)	1.9 (0.9 to 2.9)	46.4 (-34.83 to 133.51)	4.0 (2.7 to 6.3)	4.4 (2.1 to 6.5)	13.7 (-45.91 to 75.79)
Gynecological diseases	0.8 (0.6 to 1.0)	0.5 (0.4 to 0.7)	**-34.9 (-43.38 to -19.85)**	1.3 (1.1 to 1.7)	0.7 (0.6 to 1.0)	**-46.0 (-52.85 to -35.38)**	--	--	--	--	--	--	0.8 (0.6 to 1.0)	0.5 (0.4 to 0.7)	**-34.9 (-43.38 to -19.85)**	2.5 (2.1 to 3.2)	1.4 (1.2 to 2.0)	**-44.8 (-51.83 to -34.54)**
Uterine fibroids	0.2 (0.2 to 0.3)	0.1 (0.1 to 0.2)	**-33.6 (-42.85 to -16.87)**	0.3 (0.3 to 0.4)	0.2 (0.2 to 0.3)	**-47.1 (-54.36 to -34.93)**	--	--	--	--	--	--	0.2 (0.2 to 0.3)	0.1 (0.1 to 0.2)	**-33.6 (-42.85 to -16.87)**	0.6 (0.5 to 0.8)	0.3 (0.3 to 0.5)	**-45.8 (-53.35 to -33.40)**
Polycystic ovarian syndrome	<0.05 (0.0 to <0.05)	<0.05 (0.0 to <0.05)	**-34.1 (-42.85 to -23.63)**	<0.05 (0.0 to <0.05)	<0.05 (0.0 to <0.05)	**-39.3 (-47.21 to -29.74)**	--	--	--	--	--	--	<0.05 (0.0 to <0.05)	<0.05 (0.0 to <0.05)	**-34.1 (-42.85 to -23.63)**	0.1 (0.1 to 0.1)	<0.05 (0.0 to 0.1)	**-39.3 (-47.18 to -29.69)**
Endometriosis	<0.05 (0.0 to 0.1)	<0.05 (0.0 to <0.05)	**-34.4 (-43.65 to -20.11)**	0.1 (0.1 to 0.1)	0.1 (0.0 to 0.1)	**-41.5 (-49.52 to -30.17)**	--	--	--	--	--	--	<0.05 (0.0 to 0.1)	<0.05 (0.0 to <0.05)	**-34.4 (-43.65 to -20.11)**	0.2 (0.2 to 0.3)	0.1 (0.1 to 0.2)	**-41.7 (-49.62 to -30.49)**
Genital prolapse	0.1 (0.1 to 0.2)	0.1 (0.1 to 0.1)	**-34.8 (-43.58 to -19.67)**	0.2 (0.2 to 0.3)	0.1 (0.1 to 0.2)	**-46.5 (-53.41 to -35.48)**	--	--	--	--	--	--	0.1 (0.1 to 0.2)	0.1 (0.1 to 0.1)	**-34.8 (-43.58 to -19.67)**	0.4 (0.3 to 0.5)	0.2 (0.2 to 0.3)	**-44.5 (-51.49 to -34.15)**
Other gynecological diseases	0.4 (0.3 to 0.5)	0.2 (0.2 to 0.3)	**-35.7 (-44.02 to -21.28)**	0.6 (0.5 to 0.8)	0.3 (0.3 to 0.5)	**-46.4 (-53.18 to -36.08)**	--	--	--	--	--	--	0.4 (0.3 to 0.5)	0.2 (0.2 to 0.3)	**-35.7 (-44.02 to -21.28)**	1.2 (1.0 to 1.5)	0.6 (0.6 to 0.9)	**-45.2 (-52.23 to -34.88)**
Hemoglobinopathies and hemolytic anemias	7.3 (5.1 to 9.3)	4.4 (3.3 to 6.4)	**-39.0 (-51.28 to -19.90)**	14.5 (10.3 to 18.6)	7.5 (5.6 to 11.0)	**-48.2 (-58.77 to -30.87)**	3.7 (2.2 to 5.3)	2.2 (1.4 to 3.5)	**-39.6 (-50.97 to -13.67)**	15.7 (9.5 to 22.7)	7.9 (4.9 to 12.3)	**-49.7 (-59.01 to -28.17)**	3.6 (2.2 to 4.9)	2.2 (1.5 to 3.5)	**-38.2 (-52.75 to -15.13)**	13.4 (8.5 to 18.5)	7.1 (4.8 to 11.3)	**-46.8 (-59.48 to -23.11)**
Thalassemias	1.2 (0.9 to 1.6)	0.7 (0.5 to 1.0)	**-42.8 (-54.94 to -25.05)**	3.0 (2.1 to 3.8)	1.7 (1.3 to 2.5)	**-44.0 (-56.17 to -25.90)**	0.6 (0.4 to 0.9)	0.4 (0.2 to 0.6)	**-42.5 (-53.02 to -23.18)**	3.0 (1.8 to 4.4)	1.7 (1.0 to 2.7)	**-43.7 (-54.31 to -23.41)**	0.6 (0.4 to 0.8)	0.3 (0.2 to 0.5)	**-43.0 (-57.55 to -16.18)**	3.0 (1.9 to 4.1)	1.6 (1.1 to 2.6)	**-44.3 (-58.86 to -16.36)**
Sickle cell disorders	2.0 (1.4 to 2.6)	1.5 (1.1 to 2.2)	**-24.7 (-39.58 to 1.48)**	4.4 (3.1 to 5.7)	2.8 (2.1 to 4.1)	**-35.8 (-48.81 to -13.23)**	1.1 (0.7 to 1.6)	0.8 (0.5 to 1.3)	**-26.9 (-40.55 to 5.79)**	4.9 (2.9 to 7.1)	3.1 (1.9 to 4.8)	**-37.1 (-48.80 to -9.19)**	0.9 (0.6 to 1.3)	0.7 (0.5 to 1.1)	-21.9 (-40.30 to 14.09)	4.0 (2.5 to 5.4)	2.6 (1.7 to 4.2)	**-33.7 (-49.63 to -1.26)**
G6PD deficiency	0.1 (0.1 to 0.1)	<0.05 (0.0 to <0.05)	**-66.7 (-73.21 to -56.13)**	0.2 (0.1 to 0.2)	0.1 (0.0 to 0.1)	**-71.4 (-77.18 to -61.98)**	<0.05 (0.0 to 0.1)	<0.05 (0.0 to <0.05)	**-58.6 (-66.12 to -41.43)**	0.2 (0.1 to 0.3)	0.1 (0.0 to 0.1)	**-64.2 (-70.86 to -49.21)**	<0.05 (0.0 to 0.1)	<0.05 (0.0 to <0.05)	**-75.1 (-80.94 to -64.55)**	0.2 (0.1 to 0.3)	<0.05 (0.0 to 0.1)	**-78.9 (-83.95 to -69.19)**
Other hemoglobinopathies and hemolytic anemias	3.9 (2.8 to 5.0)	2.2 (1.6 to 3.1)	**-44.6 (-56.17 to -27.67)**	6.9 (4.9 to 8.8)	2.9 (2.2 to 4.2)	**-57.4 (-66.04 to -43.66)**	1.9 (1.1 to 2.7)	1.0 (0.6 to 1.6)	**-45.6 (-56.60 to -21.00)**	7.6 (4.6 to 10.7)	3.0 (1.9 to 4.7)	**-59.8 (-67.63 to -41.85)**	2.0 (1.2 to 2.8)	1.1 (0.8 to 1.8)	**-43.4 (-57.09 to -25.18)**	6.3 (3.9 to 8.6)	2.8 (1.9 to 4.5)	**-55.2 (-65.63 to -38.24)**
Endocrine, metabolic, blood, and immune disorders	50.0 (36.7 to 53.6)	36.9 (31.9 to 50.9)	-25.6 (-36.40 to 3.36)	100.3 (75.0 to 108.4)	64.9 (55.8 to 87.7)	**-34.9 (-44.31 to -12.20)**	25.9 (17.0 to 28.1)	18.8 (15.3 to 29.1)	-26.4 (-41.75 to 18.29)	110.5 (73.6 to 120.8)	68.6 (55.7 to 104.2)	**-37.1 (-50.06 to -0.20)**	24.1 (16.1 to 28.6)	18.1 (14.1 to 25.2)	**-24.5 (-34.82 to -7.90)**	91.3 (63.1 to 109.9)	61.0 (47.1 to 82.1)	**-33.0 (-42.19 to -21.24)**
**Musculoskeletal disorders**	33.1 (30.2 to 38.8)	24.0 (18.9 to 28.7)	**-27.2 (-43.76 to -15.01)**	52.6 (47.7 to 61.7)	31.5 (24.8 to 37.6)	**-40.1 (-51.43 to -29.66)**	9.7 (7.4 to 13.4)	8.5 (5.1 to 10.5)	-12.0 (-46.27 to 9.11)	35.2 (26.9 to 48.7)	23.8 (14.6 to 29.7)	**-31.8 (-57.15 to -15.68)**	23.5 (20.9 to 26.0)	15.6 (10.4 to 19.1)	**-33.3 (-59.47 to -20.85)**	67.1 (60.6 to 73.8)	38.4 (27.2 to 47.1)	**-42.6 (-62.47 to -31.48)**

Rheumatoid arthritis	18.3<br>(14.7 to 20.7)	9.3<br>(7.2 to 11.0)	**-49.2<br>(-59.14 to -40.81)**	26.9<br>(21.8 to 30.6)	10.9<br>(8.5 to 13.0)	**-59.2<br>(-67.24 to -52.57)**	4.2<br>(2.9 to 5.4)	2.8<br>(1.5 to 3.8)	**-31.2<br>(-62.11 to -5.52)**	14.6<br>(10.0 to 19.1)	7.4<br>(3.9 to 10.2)	**-48.4<br>(-70.85 to -29.11)**	14.2<br>(11.1 to 15.8)	6.4<br>(4.5 to 7.9)	**-54.2<br>(-66.79 to -43.60)**	36.3<br>(28.1 to 40.5)	13.9<br>(9.8 to 16.9)	**-61.5<br>(-71.82 to -52.91)**
Other musculoskeletal disorders	14.8<br>(13.6 to 18.7)	14.8<br>(11.5 to 18.1)	0.4<br>(-32.12 to 19.48)	25.7<br>(23.5 to 32.0)	20.5<br>(16.3 to 25.0)	**-19.7<br>(-41.90 to -4.56)**	5.5<br>(4.3 to 8.1)	5.6<br>(3.3 to 7.1)	3.0<br>(-35.38 to 29.57)	20.6<br>(16.1 to 30.2)	16.4<br>(9.9 to 20.7)	**-19.7<br>(-48.37 to 0.11)**	9.3<br>(8.5 to 11.5)	9.2<br>(6.2 to 11.4)	-0.7<br>(-42.65 to 21.88)	30.8<br>(28.1 to 36.2)	24.5<br>(17.7 to 30.7)	**-19.9<br>(-48.48 to -1.24)**
**Other non-communicable diseases**	232.4<br>(206.3 to 246.8)	136.2<br>(116.0 to 163.6)	**-41.2<br>(-51.39 to -26.11)**	624.3<br>(550.7 to 664.8)	327.7<br>(272.0 to 404.8)	**-47.3<br>(-57.63 to -31.76)**	128.2<br>(110.7 to 137.3)	71.2<br>(59.5 to 86.9)	**-44.2<br>(-54.30 to -28.51)**	684.4<br>(587.6 to 734.6)	346.4<br>(283.3 to 430.5)	**-49.1<br>(-59.18 to -33.40)**	104.2<br>(89.0 to 116.7)	65.0<br>(54.6 to 80.2)	**-37.4<br>(-49.03 to -22.61)**	560.7<br>(473.5 to 634.7)	307.4<br>(249.3 to 391.3)	**-45.0<br>(-56.79 to -28.82)**
Congenital anomalies	158.3<br>(151.1 to 170.5)	100.0<br>(84.4 to 119.8)	**-36.8<br>(-46.27 to -24.29)**	428.6<br>(408.3 to 462.3)	254.3<br>(211.5 to 308.6)	**-40.7<br>(-50.19 to -27.92)**	86.0<br>(79.5 to 94.3)	54.1<br>(44.9 to 65.7)	**-37.1<br>(-47.12 to -24.31)**	457.4<br>(421.4 to 503.0)	270.6<br>(221.7 to 332.6)	**-40.8<br>(-50.51 to -27.83)**	72.3<br>(65.6 to 80.3)	46.0<br>(37.5 to 56.3)	**-36.4<br>(-47.21 to -23.40)**	398.9<br>(360.2 to 444.6)	237.3<br>(191.8 to 292.5)	**-40.5<br>(-51.19 to -27.46)**
Neural tube defects	10.4<br>(9.2 to 13.2)	4.8<br>(3.7 to 6.2)	**-53.0<br>(-67.32 to -38.91)**	28.8<br>(25.5 to 36.6)	13.0<br>(9.9 to 16.6)	**-54.6<br>(-68.59 to -40.65)**	4.2<br>(3.7 to 5.7)	2.2<br>(1.6 to 2.9)	**-48.4<br>(-65.49 to -31.28)**	22.9<br>(19.7 to 31.0)	11.3<br>(8.0 to 15.2)	**-50.1<br>(-66.72 to -32.91)**	6.1<br>(5.2 to 7.9)	2.7<br>(2.0 to 3.7)	**-56.0<br>(-69.36 to -40.31)**	35.0<br>(29.3 to 45.0)	14.7<br>(10.6 to 20.4)	**-57.6<br>(-70.54 to -41.88)**
Congenital heart anomalies	78.9<br>(74.8 to 85.7)	38.9<br>(32.9 to 46.5)	**-50.6<br>(-57.93 to -42.24)**	209.9<br>(198.2 to 228.7)	97.0<br>(81.4 to 116.7)	**-53.7<br>(-61.00 to -45.21)**	43.7<br>(39.4 to 48.2)	21.1<br>(17.7 to 25.3)	**-51.6<br>(-59.14 to -42.10)**	228.3<br>(205.8 to 253.2)	103.7<br>(85.7 to 125.8)	**-54.5<br>(-62.05 to -45.04)**	35.2<br>(31.8 to 39.4)	17.8<br>(14.5 to 21.8)	**-49.4<br>(-58.28 to -39.81)**	191.0<br>(171.9 to 213.9)	90.2<br>(73.1 to 111.6)	**-52.7<br>(-61.43 to -43.06)**
Cleft lip and cleft palate	0.2<br>(0.1 to 0.4)	<0.05<br>(0.0 to 0.1)	**-70.0<br>(-90.87 to -40.00)**	0.5<br>(0.3 to 1.2)	0.1<br>(0.1 to 0.3)	**-70.7<br>(-91.10 to -41.39)**	0.1<br>(0.1 to 0.3)	<0.05<br>(0.0 to 0.1)	**-70.3<br>(-93.38 to -24.70)**	0.7<br>(0.3 to 1.9)	0.2<br>(0.0 to 0.4)	**-70.9<br>(-93.55 to -26.40)**	0.1<br>(0.0 to 0.2)	<0.05<br>(0.0 to <0.05)	**-66.6<br>(-91.00 to -38.43)**	0.4<br>(0.2 to 1.0)	0.1<br>(0.0 to 0.2)	**-67.4<br>(-91.21 to -39.93)**
Down syndrome	5.0<br>(4.0 to 6.7)	7.1<br>(4.5 to 8.8)	44.4<br>(-15.23 to 85.77)	11.2<br>(8.9 to 15.4)	13.6<br>(8.6 to 17.3)	23.8<br>(-29.59 to 62.26)	2.5<br>(1.8 to 3.9)	3.5<br>(1.9 to 4.5)	43.9<br>(-37.04 to 86.97)	11.4<br>(8.3 to 17.9)	13.7<br>(6.9 to 17.7)	23.8<br>(-47.77 to 63.35)	2.5<br>(1.7 to 3.7)	3.6<br>(1.9 to 4.7)	47.3<br>(-30.82 to 94.52)	11.0<br>(7.7 to 16.8)	13.6<br>(7.1 to 18.0)	25.8<br>(-43.73 to 67.93)
Chromosomal unbalanced rearrangements	7.0<br>(5.7 to 9.7)	7.5<br>(5.3 to 9.9)	8.8<br>(-24.40 to 47.78)	19.8<br>(16.2 to 27.3)	20.7<br>(14.6 to 27.4)	6.0<br>(-26.52 to 44.28)	2.6<br>(2.2 to 3.9)	3.1<br>(2.0 to 4.2)	18.1<br>(-30.65 to 61.75)	14.5<br>(11.9 to 21.3)	16.3<br>(10.8 to 22.4)	15.1<br>(-32.82 to 58.47)	4.4<br>(3.1 to 6.7)	4.5<br>(2.8 to 6.5)	3.8<br>(-30.88 to 43.10)	25.4<br>(18.1 to 38.6)	25.3<br>(15.5 to 36.6)	1.2<br>(-32.96 to 39.87)
Other congenital anomalies	56.9<br>(52.8 to 61.3)	41.6<br>(33.9 to 51.6)	**-26.9<br>(-40.67 to -9.29)**	158.4<br>(146.9 to 171.0)	109.7<br>(88.7 to 137.5)	**-30.7<br>(-44.43 to -12.94)**	32.8<br>(29.8 to 36.0)	24.2<br>(19.5 to 30.7)	**-26.2<br>(-40.43 to -6.80)**	179.7<br>(163.2 to 197.5)	125.4<br>(100.2 to 160.8)	**-30.1<br>(-44.15 to -11.28)**	24.0<br>(20.9 to 27.4)	17.4<br>(13.6 to 22.2)	**-27.6<br>(-43.83 to -8.65)**	136.2<br>(118.2 to 155.8)	93.4<br>(72.0 to 120.3)	**-31.3<br>(-47.19 to -12.85)**
Skin and subcutaneous diseases	10.6<br>(10.0 to 13.3)	16.8<br>(12.1 to 18.6)	**58.6<br>(-6.21 to 76.99)**	16.1<br>(15.1 to 20.1)	19.5<br>(14.3 to 21.7)	22.3<br>(-27.62 to 35.69)	3.7<br>(3.4 to 5.0)	6.3<br>(4.0 to 7.4)	**73.9<br>(-14.90 to 108.15)**	13.8<br>(13.0 to 18.6)	17.1<br>(11.0 to 20.1)	25.1<br>(-38.15 to 49.69)	7.0<br>(6.4 to 8.4)	10.5<br>(7.5 to 11.9)	**50.8<br>(-1.98 to 74.39)**	17.3<br>(16.0 to 20.9)	21.2<br>(15.8 to 24.0)	22.9<br>(-20.98 to 41.42)
Cellulitis	3.3<br>(2.7 to 5.0)	6.9<br>(4.0 to 8.7)	**119.5<br>(-16.17 to 204.54)**	5.2<br>(4.3 to 7.7)	8.0<br>(4.7 to 10.0)	62.0<br>(-36.03 to 122.53)	1.3<br>(1.1 to 2.0)	2.7<br>(1.4 to 3.5)	**106.6<br>(-26.66 to 195.01)**	5.0<br>(4.1 to 7.5)	7.1<br>(3.8 to 9.2)	47.5<br>(-47.38 to 109.66)	2.0<br>(1.5 to 3.1)	4.3<br>(2.2 to 5.6)	**130.1<br>(-11.27 to 242.67)**	5.2<br>(4.1 to 8.0)	8.6<br>(4.7 to 11.2)	75.5<br>(-25.75 to 160.05)
Pyoderma	1.0<br>(0.8 to 1.4)	6.3<br>(4.4 to 8.4)	**525.8<br>(225.52 to 767.75)**	1.7<br>(1.5 to 2.4)	7.4<br>(5.3 to 9.8)	**331.7<br>(130.11 to 488.36)**	0.5<br>(0.4 to 0.7)	2.4<br>(1.6 to 3.4)	**413.3<br>(145.52 to 644.00)**	1.9<br>(1.5 to 2.6)	6.7<br>(4.4 to 9.3)	**260.6<br>(73.05 to 421.22)**	0.5<br>(0.4 to 0.7)	3.9<br>(2.5 to 5.5)	**628.3<br>(305.15 to 976.97)**	1.6<br>(1.3 to 2.1)	7.9<br>(5.3 to 11.1)	**395.3<br>(179.32 to 629.55)**
Decubitus ulcer	5.2<br>(4.3 to 5.9)	2.6<br>(2.0 to 3.6)	**-50.1<br>(-63.57 to -29.82)**	7.3<br>(6.0 to 8.3)	2.8<br>(2.2 to 3.9)	**-61.0<br>(-71.42 to -46.07)**	1.4<br>(1.1 to 1.9)	0.9<br>(0.6 to 1.3)	**-39.9<br>(-61.12 to -2.27)**	5.3<br>(4.1 to 6.8)	2.2<br>(1.6 to 3.3)	**-57.8<br>(-72.15 to -32.40)**	3.8<br>(2.9 to 4.3)	1.7<br>(1.3 to 2.6)	**-53.9<br>(-67.15 to -30.82)**	8.4<br>(6.4 to 9.7)	3.2<br>(2.4 to 4.9)	**-60.9<br>(-72.02 to -41.75)**
Other skin and subcutaneous diseases	1.1<br>(0.9 to 1.6)	1.0<br>(0.7 to 1.6)	-12.6<br>(-43.48 to 47.80)	1.9<br>(1.5 to 2.7)	1.3<br>(0.9 to 2.0)	-32.8<br>(-55.76 to 8.75)	0.4<br>(0.3 to 0.5)	0.4<br>(0.2 to 0.6)	-5.6<br>(-49.02 to 71.88)	1.6<br>(1.0 to 2.0)	1.1<br>(0.7 to 1.8)	-29.7<br>(-62.72 to 26.36)	0.7<br>(0.5 to 1.1)	0.6<br>(0.4 to 1.1)	-15.8<br>(-46.54 to 47.71)	2.2<br>(1.6 to 3.0)	1.4<br>(1.0 to 2.5)	-33.9<br>(-55.93 to 10.54)
Sudden infant death syndrome	63.4<br>(33.0 to 78.9)	19.4<br>(12.0 to 36.6)	**-66.2<br>(-83.19 to -23.74)**	179.6<br>(93.4 to 223.6)	53.9<br>(33.4 to 101.5)	**-66.9<br>(-83.54 to -25.32)**	38.5<br>(18.0 to 46.6)	10.8<br>(7.1 to 20.9)	**-69.0<br>(-83.01 to -27.57)**	213.1<br>(99.4 to 257.9)	58.8<br>(38.6 to 113.1)	**-69.6<br>(-83.37 to -29.09)**	24.9<br>(11.0 to 35.2)	8.6<br>(4.5 to 18.9)	**-60.9<br>(-83.95 to -8.73)**	144.5<br>(64.1 to 204.2)	48.9<br>(25.7 to 107.3)	**-61.7<br>(-84.28 to -10.63)**
**Injuries**	614.4<br>(585.8 to 639.6)	393.5<br>(366.1 to 424.1)	**-35.9<br>(-40.77 to -31.06)**	1,263.5<br>(1,204.7 to 1,312.0)	694.9<br>(647.4 to 751.6)	**-45.0<br>(-49.04 to -40.56)**	446.8<br>(418.4 to 464.3)	280.7<br>(253.3 to 308.5)	**-37.2<br>(-42.86 to -31.18)**	1,882.6<br>(1,773.3 to 1,954.4)	1,031.1<br>(933.3 to 1,132.8)	**-45.2<br>(-50.01 to -40.08)**	167.7<br>(157.3 to 183.2)	112.8<br>(100.1 to 125.6)	**-32.7<br>(-40.91 to -26.53)**	643.9<br>(602.1 to 693.7)	357.9<br>(324.1 to 397.7)	**-44.4<br>(-49.71 to -39.28)**
**Transport injuries**	217.5<br>(207.2 to 226.9)	101.6<br>(92.7 to 110.5)	**-53.3<br>(-56.75 to -49.41)**	472.8<br>(451.2 to 494.1)	201.9<br>(183.8 to 219.6)	**-57.3<br>(-60.55 to -53.50)**	169.7<br>(163.5 to 176.0)	78.9<br>(71.7 to 86.5)	**-53.5<br>(-57.58 to -48.87)**	737.4<br>(709.0 to 766.7)	313.1<br>(283.8 to 343.5)	**-57.5<br>(-61.34 to -53.15)**	47.8<br>(38.5 to 53.8)	22.8<br>(18.9 to 27.8)	**-52.3<br>(-56.92 to -46.22)**	206.3<br>(166.2 to 233.6)	89.3<br>(75.3 to 108.8)	**-56.7<br>(-60.92 to -51.47)**
Road injuries	210.7<br>(200.5 to 220.0)	96.9<br>(88.2 to 105.4)	**-54.0<br>(-57.50 to -50.23)**	458.5<br>(436.9 to 478.4)	192.8<br>(175.2 to 209.5)	**-58.0<br>(-61.21 to -54.20)**	164.3<br>(157.7 to 170.8)	75.1<br>(68.4 to 82.4)	**-54.3<br>(-58.21 to -49.74)**	714.7<br>(686.5 to 744.0)	298.7<br>(271.1 to 327.6)	**-58.2<br>(-61.94 to -53.82)**	46.4<br>(37.6 to 52.5)	21.8<br>(18.2 to 27.0)	**-53.0<br>(-57.60 to -46.93)**	200.3<br>(162.1 to 227.7)	85.4<br>(72.0 to 104.9)	**-57.3<br>(-61.63 to -52.19)**
Pedestrian road injuries	50.9<br>(37.8 to 65.9)	20.8<br>(15.7 to 28.5)	**-59.1<br>(-63.73 to -53.07)**	110.9<br>(82.9 to 142.9)	39.2<br>(29.6 to 53.8)	**-64.7<br>(-68.63 to -59.22)**	36.6<br>(23.6 to 51.4)	15.3<br>(10.3 to 22.5)	**-58.1<br>(-63.80 to -50.26)**	161.8<br>(105.2 to 227.2)	58.1<br>(38.7 to 85.5)	**-64.0<br>(-68.95 to -57.13)**	14.3<br>(10.1 to 18.5)	5.5<br>(3.8 to 7.5)	**-61.7<br>(-66.41 to -54.27)**	60.5<br>(42.8 to 78.5)	20.3<br>(13.9 to 27.9)	**-66.4<br>(-70.89 to -60.40)**
Cyclist road injuries	13.0<br>(9.8 to 16.6)	5.5<br>(3.9 to 6.9)	**-57.7<br>(-65.12 to -50.92)**	30.7<br>(23.0 to 39.1)	11.2<br>(8.0 to 13.9)	**-63.2<br>(-69.81 to -57.25)**	10.5<br>(7.3 to 13.8)	4.2<br>(2.6 to 5.5)	**-59.7<br>(-68.22 to -51.11)**	49.5<br>(34.5 to 64.7)	17.2<br>(10.9 to 22.6)	**-65.1<br>(-72.60 to -57.48)**	2.6<br>(1.8 to 3.4)	1.3<br>(0.8 to 1.7)	**-49.7<br>(-59.15 to -40.04)**	11.6<br>(8.4 to 15.2)	5.2<br>(3.4 to 6.9)	**-55.2<br>(-63.45 to -46.40)**
Motorcyclist road injuries	41.7<br>(29.9 to 53.6)	18.7<br>(12.9 to 23.8)	**-55.1<br>(-63.59 to -47.70)**	90.4<br>(64.5 to 116.4)	38.4<br>(26.4 to 49.1)	**-57.5<br>(-65.70 to -50.67)**	38.7<br>(27.0 to 50.6)	17.6<br>(11.7 to 22.4)	**-54.5<br>(-63.69 to -46.46)**	166.4<br>(115.3 to 216.6)	71.5<br>(48.1 to 91.6)	**-56.9<br>(-65.78 to -49.37)**	3.0<br>(1.3 to 5.6)	1.1<br>(0.5 to 2.1)	**-62.6<br>(-69.13 to -55.06)**	13.1<br>(5.6 to 24.4)	4.6<br>(2.1 to 8.6)	**-65.0<br>(-71.38 to -57.83)**
Motor vehicle road injuries	103.6<br>(85.6 to 119.1)	51.3<br>(42.2 to 58.7)	**-50.4<br>(-54.95 to -44.27)**	223.3<br>(185.1 to 257.5)	102.9<br>(84.8 to 117.5)	**-53.9<br>(-58.25 to -48.19)**	77.6<br>(61.2 to 92.6)	37.8<br>(29.7 to 44.7)	**-51.2<br>(-57.14 to -44.12)**	332.9<br>(264.2 to 398.3)	150.9<br>(118.1 to 178.1)	**-54.6<br>(-60.17 to -47.91)**	26.0<br>(19.3 to 31.7)	13.6<br>(10.4 to 16.6)	**-47.7<br>(-53.89 to -40.53)**	112.8<br>(84.0 to 136.4)	54.1<br>(41.4 to 66.0)	**-52.0<br>(-57.44 to -45.54)**
Other road injuries	1.5<br>(0.8 to 1.9)	0.6<br>(0.4 to 0.9)	**-60.7<br>(-69.62 to -35.48)**	3.2<br>(1.8 to 4.1)	1.1<br>(0.8 to 1.7)	**-65.1<br>(-73.10 to -43.64)**	1.0<br>(0.4 to 1.3)	0.3<br>(0.2 to 0.5)	**-72.3<br>(-80.49 to -49.35)**	4.1<br>(1.6 to 5.6)	1.0<br>(0.6 to 1.8)	**-75.3<br>(-82.39 to -56.52)**	0.5<br>(0.3 to 0.8)	0.3<br>(0.2 to 0.5)	**-39.7<br>(-55.18 to -13.88)**	2.3<br>(1.4 to 3.3)	1.2<br>(0.7 to 1.8)	**-46.8<br>(-60.71 to -23.98)**
Other transport injuries	6.7<br>(5.6 to 8.2)	4.7<br>(3.7 to 5.6)	**-29.8<br>(-40.88 to -18.87)**	14.4<br>(11.9 to 17.5)	9.1<br>(7.1 to 10.9)	**-36.5<br>(-46.49 to -26.69)**	5.4<br>(4.5 to 6.7)	3.8<br>(2.8 to 4.5)	**-29.8<br>(-43.84 to -16.03)**	22.7<br>(18.7 to 28.3)	14.4<br>(10.6 to 17.1)	**-36.6<br>(-49.32 to -24.01)**	1.4<br>(0.8 to 2.0)	1.0<br>(0.6 to 1.5)	**-29.5<br>(-41.73 to -12.53)**	6.0<br>(3.7 to 8.8)	3.9<br>(2.5 to 6.1)	**-35.9<br>(-46.88 to -20.41)**
**Unintentional injuries**	155.5<br>(146.9 to 172.2)	141.0<br>(123.7 to 154.2)	**-9.2<br>(-22.18 to 0.06)**	304.7<br>(287.0 to 334.0)	220.6<br>(196.4 to 242.1)	**-27.5<br>(-35.92 to -20.01)**	97.5<br>(89.6 to 108.3)	84.9<br>(72.0 to 95.5)	**-12.8<br>(-25.80 to -1.97)**	415.2<br>(381.2 to 461.4)	292.4<br>(251.4 to 328.3)	**-29.5<br>(-38.08 to -21.23)**	58.0<br>(52.4 to 67.9)	56.1<br>(46.7 to 63.4)	-3.0<br>(-21.26 to 10.21)	193.9<br>(177.0 to 222.1)	148.8<br>(127.6 to 168.2)	**-23.1<br>(-34.67 to -13.86)**
Falls	50.8<br>(46.9 to 66.3)	66.1<br>(48.1 to 76.2)	31.8<br>(-16.79 to 59.58)	83.6<br>(77.5 to 108.3)	83.5<br>(61.5 to 96.4)	1.0<br>(-35.79 to 22.03)	27.7<br>(25.7 to 36.2)	37.3<br>(25.3 to 43.7)	36.0<br>(-18.82 to 64.74)	110.0<br>(101.6 to 144.2)	110.9<br>(76.5 to 130.6)	1.9<br>(-37.91 to 23.66)	23.1<br>(20.4 to 33.2)	28.8<br>(19.7 to 34.0)	27.7<br>(-20.75 to 59.20)	57.0<br>(50.7 to 80.9)	56.1<br>(39.2 to 66.5)	0.6<br>(-36.80 to 25.63)
Drowning	16.3<br>(13.3 to 19.0)	9.2<br>(7.3 to 11.6)	**-43.4<br>(-51.39 to -32.76)**	38.1<br>(31.1 to 44.0)	19.4<br>(15.5 to 24.2)	**-49.0<br>(-56.42 to -39.81)**	12.8<br>(10.0 to 15.2)	7.4<br>(5.6 to 9.3)	**-42.7<br>(-51.59 to -31.88)**	59.5<br>(46.5 to 70.4)	30.8<br>(23.3 to 38.8)	**-48.2<br>(-56.40 to -39.07)**	3.5<br>(2.9 to 4.2)	1.9<br>(1.4 to 2.5)	**-45.9<br>(-56.34 to -32.82)**	16.4<br>(13.5 to 19.7)	7.9<br>(5.9 to 10.5)	**-51.9<br>(-61.70 to -39.44)**
Fire, heat, and hot substances	19.4<br>(15.4 to 21.8)	9.3<br>(7.8 to 12.8)	**-51.7<br>(-59.88 to -21.72)**	41.6<br>(32.8 to 47.1)	17.0<br>(14.1 to 23.1)	**-58.7<br>(-66.22 to -32.52)**	11.2<br>(8.6 to 13.2)	5.4<br>(4.4 to 7.9)	**-51.3<br>(-60.22 to -15.17)**	49.7<br>(38.0 to 59.1)	20.4<br>(16.3 to 29.1)	**-58.6<br>(-66.53 to -28.13)**	8.2<br>(6.1 to 9.5)	3.9<br>(3.0 to 5.4)	**-52.1<br>(-60.74 to -25.67)**	33.6<br>(25.0 to 39.0)	13.6<br>(10.7 to 19.0)	**-59.0<br>(-66.79 to -35.77)**
Poisonings	17.9<br>(11.0 to 19.8)	5.9<br>(4.6 to 11.0)	**-65.5<br>(-75.73 to -26.41)**	37.2<br>(22.9 to 41.3)	10.8<br>(8.6 to 20.1)	**-69.5<br>(-78.42 to -35.61)**	12.8<br>(6.5 to 14.3)	3.5<br>(2.7 to 7.6)	**-70.5<br>(-79.84 to -29.14)**	53.5<br>(27.1 to 59.7)	13.2<br>(10.3 to 28.5)	**-73.5<br>(-81.76 to -36.89)**	5.2<br>(2.9 to 5.9)	2.4<br>(1.8 to 4.4)	**-51.8<br>(-67.60 to -12.25)**	21.0<br>(11.6 to 23.9)	8.3<br>(6.4 to 15.4)	**-58.1<br>(-71.76 to -24.80)**
Exposure to mechanical forces	12.3<br>(11.5 to 15.3)	13.6<br>(9.6 to 15.4)	11.4<br>(-32.63 to 27.39)	27.5<br>(25.7 to 34.8)	29.1<br>(20.1 to 33.2)	6.5<br>(-37.36 to 23.81)	12.8<br>(9.7 to 12.8)	10.4<br>(6.9 to 12.0)	0.5<br>(-38.27 to 16.08)	45.7<br>(42.5 to 56.5)	43.4<br>(28.2 to 50.2)	-4.4<br>(-43.94 to 11.52)	1.9<br>(1.7 to 3.1)	3.2<br>(2.0 to 3.9)	74.4<br>(-19.59 to 122.79)	9.3<br>(8.1 to 15.4)	14.6<br>(8.4 to 18.2)	63.5<br>(-28.94 to 114.47)
Unintentional firearm injuries	1.0<br>(0.9 to 1.0)	0.4<br>(0.4 to 0.5)	**-52.9<br>(-58.63 to -46.13)**	2.1<br>(2.0 to 2.3)	0.9<br>(0.8 to 1.1)	**-55.3<br>(-60.96 to -48.98)**	0.8<br>(0.7 to 0.9)	0.3<br>(0.2 to 0.3)	**-63.5<br>(-68.99 to -56.60)**	3.5<br>(3.1 to 3.8)	1.2<br>(1.0 to 1.4)	**-65.7<br>(-71.10 to -59.61)**	0.2<br>(0.2 to 0.2)	0.2<br>(0.1 to 0.2)	-1.8<br>(-38.71 to 15.19)	0.8<br>(0.7 to 0.9)	0.7<br>(0.6 to 0.8)	-8.2<br>(-43.83 to 8.73)
Unintentional suffocation	2.3<br>(1.9 to 4.4)	7.5<br>(3.3 to 9.0)	**251.1<br>(-2.37 to 347.88)**	5.7<br>(4.7 to 10.9)	17.1<br>(7.7 to 20.7)	**222.1<br>(-9.58 to 315.59)**	1.5<br>(1.3 to 3.3)	5.6<br>(2.1 to 6.8)	**295.1<br>(-9.44 to 398.03)**	7.3<br>(6.0 to 15.7)	24.9<br>(9.3 to 30.4)	**262.2<br>(-17.08 to 359.25)**	0.8<br>(0.6 to 1.9)	1.9<br>(0.6 to 2.5)	179.6<br>(-47.74 to 294.52)	4.0<br>(3.0 to 9.6)	9.1<br>(2.9 to 12.1)	160.1<br>(-52.19 to 273.15)
Other exposure to mechanical forces	9.1<br>(8.5 to 10.1)	5.7<br>(5.0 to 6.7)	**-37.4<br>(-45.72 to -24.95)**	19.7<br>(18.4 to 22.0)	11.1<br>(9.6 to 13.1)	**-43.7<br>(-51.65 to -33.13)**	8.1<br>(7.1 to 8.7)	4.5<br>(3.9 to 5.5)	**-44.4<br>(-51.43 to -32.37)**	34.9<br>(30.7 to 37.9)	17.4<br>(15.1 to 21.2)	**-50.1<br>(-56.30 to -39.70)**	1.0<br>(0.9 to 1.4)	1.2<br>(0.8 to 1.4)	20.5<br>(-33.14 to 49.88)	4.6<br>(4.1 to 6.6)	4.8<br>(3.1 to 5.9)	6.4<br>(-42.14 to 34.26)
Adverse effects of medical treatment	15.6<br>(12.0 to 17.8)	11.8<br>(9.6 to 16.1)	-23.8<br>(-37.34 to 4.17)	27.2<br>(21.0 to 31.2)	16.9<br>(13.5 to 23.3)	**-37.4<br>(-48.47 to -14.24)**	6.7<br>(5.0 to 8.8)	5.7<br>(3.9 to 7.9)	-13.7<br>(-37.23 to 13.70)	26.6<br>(19.9 to 34.8)	17.8<br>(12.0 to 24.8)	**-32.5<br>(-51.08 to -11.22)**	8.9<br>(5.7 to 10.5)	6.1<br>(4.8 to 9.2)	**-30.8<br>(-48.97 to 2.12)**	27.6<br>(17.9 to 32.3)	16.1<br>(12.7 to 24.9)	**-40.9<br>(-55.75 to -12.56)**
Animal contact	0.6<br>(0.5 to 1.1)	1.0<br>(0.6 to 1.3)	71.1<br>(-6.28 to 109.62)	1.3<br>(1.0 to 2.5)	1.9<br>(1.1 to 2.4)	47.5<br>(-19.18 to 81.85)	0.3<br>(0.3 to 0.6)	0.6<br>(0.3 to 0.7)	88.8<br>(-5.79 to 127.41)	1.4<br>(1.2 to 2.5)	2.1<br>(1.0 to 2.5)	59.8<br>(-21.01 to 93.03)	0.3<br>(0.2 to 0.7)	0.4<br>(0.2 to 0.7)	53.8<br>(-11.44 to 101.61)	1.3<br>(0.8 to 3.1)	1.7<br>(0.6 to 2.6)	35.5<br>(-22.42 to 78.64)
Venomous animal contact	0.2<br>(0.2 to 0.4)	0.3<br>(0.2 to 0.4)	23.5<br>(-20.34 to 61.03)	0.5<br>(0.4 to 1.0)	0.6<br>(0.3 to 0.8)	8.5<br>(-30.99 to 39.79)	0.1<br>(0.1 to 0.2)	0.2<br>(0.1 to 0.2)	33.7<br>(-24.27 to 66.52)	0.6<br>(0.4 to 0.9)	0.6<br>(0.3 to 0.8)	16.7<br>(-34.42 to 44.63)	0.1<br>(0.1 to 0.3)	0.1<br>(0.0 to 0.2)	13.4<br>(-29.28 to 71.20)	0.5<br>(0.3 to 1.3)	0.5<br>(0.2 to 1.0)	0.5<br>(-38.48 to 51.96)
Non-venomous animal contact	0.4<br>(0.3 to 0.7)	0.7<br>(0.4 to 0.9)	**104.5<br>(-2.40 to 151.28)**	0.8<br>(0.6 to 1.5)	1.3<br>(0.7 to 1.7)	75.4<br>(-15.71 to 118.21)	0.2<br>(0.2 to 0.3)	0.4<br>(0.2 to 0.5)	**126.6<br>(-7.07 to 179.94)**	0.8<br>(0.7 to 1.4)	1.5<br>(0.7 to 1.8)	90.3<br>(-22.12 to 136.59)	0.2<br>(0.1 to 0.4)	0.3<br>(0.1 to 0.5)	84.4<br>(-5.58 to 152.22)	0.8<br>(0.4 to 2.0)	1.2<br>(0.4 to 1.8)	61.8<br>(-16.24 to 123.88)
Foreign body	17.6<br>(13.7 to 28.0)	21.6<br>(13.3 to 26.9)	26.6<br>(-17.79 to 64.51)	36.9<br>(28.8 to 58.7)	36.9<br>(23.5 to 47.3)	2.7<br>(-31.34 to 32.51)	11.6<br>(7.8 to 20.3)	12.9<br>(6.2 to 17.4)	14.0<br>(-30.94 to 47.68)	51.0<br>(34.1 to 88.7)	46.2<br>(22.9 to 64.0)	-6.6<br>(-41.41 to 20.87)	5.9<br>(4.2 to 9.9)	8.7<br>(4.9 to 12.2)	**52.5<br>(0.73 to 110.89)**	23.4<br>(16.7 to 39.1)	27.9<br>(16.2 to 39.2)	23.3<br>(-16.90 to 70.54)
Pulmonary aspiration and foreign body in airway	17.2<br>(13.2 to 27.6)	21.2<br>(12.9 to 26.4)	27.1<br>(-17.97 to 65.48)	36.1<br>(27.9 to 57.9)	36.1<br>(22.7 to 46.6)	2.9<br>(-31.46 to 33.17)	11.4<br>(7.6 to 20.0)	12.6<br>(6.0 to 17.1)	14.1<br>(-31.27 to 48.22)	50.0<br>(33.2 to 87.6)	45.3<br>(22.1 to 62.4)	-6.6<br>(-41.57 to 21.12)	5.8<br>(4.1 to 9.6)	8.6<br>(4.7 to 12.0)	**53.8<br>(0.45 to 113.46)**	22.7<br>(16.0 to 38.3)	27.2<br>(15.6 to 38.5)	24.3<br>(-17.38 to 72.81)
Foreign body in other body part	0.4<br>(0.3 to 0.6)	0.4<br>(0.3 to 0.5)	8.4<br>(-18.75 to 29.34)	0.8<br>(0.7 to 1.2)	0.8<br>(0.6 to 1.0)	-6.1<br>(-28.48 to 11.37)	0.2<br>(0.1 to 0.4)	0.2<br>(0.1 to 0.4)	8.0<br>(-23.30 to 32.50)	1.0<br>(0.6 to 1.7)	0.9<br>(0.5 to 1.3)	-6.9<br>(-34.03 to 13.36)	0.2<br>(0.1 to 0.2)	0.2<br>(0.1 to 0.2)	9.4<br>(-38.21 to 31.15)	0.7<br>(0.6 to 1.0)	0.7<br>(0.4 to 0.8)	-5.2<br>(-45.76 to 11.11)
Other unintentional injuries	4.9<br>(4.6 to 5.4)	2.5<br>(2.2 to 2.8)	**-49.6<br>(-56.13 to -42.49)**	11.1<br>(10.4 to 12.4)	5.0<br>(4.4 to 5.8)	**-54.8<br>(-61.30 to -47.43)**	4.0<br>(3.7 to 4.3)	1.8<br>(1.6 to 2.1)	**-53.6<br>(-60.63 to -46.13)**	17.9<br>(16.6 to 19.7)	7.5<br>(6.5 to 8.7)	**-58.1<br>(-64.89 to -50.47)**	0.9<br>(0.8 to 1.3)	0.6<br>(0.5 to 0.7)	**-32.2<br>(-47.10 to -18.95)**	4.3<br>(3.7 to 6.0)	2.5<br>(2.1 to 3.1)	**-40.8<br>(-55.79 to -26.40)**
**Self-harm and interpersonal violence**	241.4<br>(211.9 to 256.5)	150.8<br>(132.5 to 177.0)	**-37.5<br>(-44.27 to -26.20)**	485.7<br>(426.1 to 516.3)	272.5<br>(239.6 to 319.5)	**-43.9<br>(-50.05 to -33.56)**	179.5<br>(147.2 to 194.6)	116.9<br>(98.1 to 141.0)	**-34.8<br>(-44.08 to -20.79)**	729.6<br>(598.6 to 788.9)	425.6<br>(359.4 to 517.0)	**-41.6<br>(-49.90 to -28.95)**	61.9<br>(55.5 to 68.5)	33.9<br>(29.9 to 41.1)	**-45.1<br>(-51.27 to -37.12)**	243.5<br>(218.5 to 271.3)	119.8<br>(105.7 to 144.5)	**-50.8<br>(-56.31 to -43.85)**
Self-harm	223.7<br>(195.3 to 240.4)	142.0<br>(123.9 to 168.0)	**-36.4<br>(-43.67 to -24.56)**	446.7<br>(388.8 to 479.5)	254.4<br>(222.5 to 299.7)	**-43.0<br>(-49.65 to -32.24)**	167.1<br>(136.9 to 183.2)	110.9<br>(92.8 to 133.3)	**-33.5<br>(-43.22 to -19.53)**	676.2<br>(553.7 to 741.1)	401.5<br>(338.2 to 484.1)	**-40.5<br>(-49.16 to -27.60)**	56.5<br>(49.5 to 62.2)	31.1<br>(27.4 to 38.3)	**-45.0<br>(-51.46 to -36.06)**	219.0<br>(192.0 to 241.1)	107.8<br>(95.1 to 132.5)	**-50.8<br>(-56.65 to -42.80)**
Interpersonal violence	17.7<br>(13.7 to 24.3)	8.8<br>(6.5 to 11.8)	**-50.1<br>(-56.56 to -37.69)**	39.0<br>(30.3 to 53.5)	18.0<br>(13.3 to 24.1)	**-53.6<br>(-59.75 to -41.85)**	12.3<br>(8.2 to 17.7)	6.0<br>(3.9 to 9.2)	**-51.5<br>(-58.59 to -35.17)**	53.4<br>(35.7 to 77.0)	24.1<br>(15.6 to 36.6)	**-54.8<br>(-61.44 to -39.63)**	5.3<br>(4.2 to 7.5)	2.8<br>(1.9 to 3.7)	**-46.4<br>(-58.45 to -31.91)**	24.4<br>(19.3 to 34.3)	12.0<br>(8.1 to 15.7)	**-50.6<br>(-61.89 to -36.72)**
Assault by firearm	3.0<br>(1.9 to 4.6)	1.7<br>(1.0 to 2.7)	**-44.5<br>(-63.06 to -17.60)**	6.4<br>(4.1 to 9.8)	3.3<br>(2.0 to 5.4)	**-47.6<br>(-65.05 to -22.30)**	2.5<br>(1.4 to 4.0)	1.3<br>(0.7 to 2.3)	**-46.0<br>(-67.20 to -12.83)**	10.3<br>(5.9 to 16.8)	5.2<br>(2.6 to 9.2)	**-49.0<br>(-69.06 to -18.00)**	0.6<br>(0.3 to 0.9)	0.4<br>(0.2 to 0.6)	**-37.2<br>(-56.58 to -15.99)**	2.6<br>(1.4 to 4.1)	1.5<br>(0.8 to 2.4)	**-41.8<br>(-59.68 to -22.08)**
Assault by sharp object	2.6<br>(1.8 to 4.0)	1.9<br>(1.2 to 2.9)	**-26.0<br>(-47.21 to -5.68)**	5.5<br>(3.9 to 8.6)	3.9<br>(2.5 to 5.9)	**-29.7<br>(-49.65 to -10.13)**	1.7<br>(1.0 to 3.0)	1.3<br>(0.7 to 2.3)	-25.1<br>(-48.24 to 7.51)	7.4<br>(4.4 to 12.8)	5.2<br>(2.9 to 9.2)	**-28.6<br>(-50.50 to 1.81)**	0.8<br>(0.6 to 1.4)	0.6<br>(0.4 to 0.9)	**-26.6<br>(-53.08 to -4.63)**	3.7<br>(2.5 to 5.9)	2.5<br>(1.4 to 3.9)	**-31.0<br>(-55.42 to -10.45)**
Assault by other means	12.0<br>(9.1 to 15.7)	5.2<br>(3.9 to 7.2)	**-56.4<br>(-62.64 to -43.16)**	27.0<br>(20.6 to 35.4)	10.9<br>(8.0 to 14.6)	**-59.7<br>(-65.48 to -47.38)**	8.1<br>(5.3 to 11.6)	3.4<br>(2.2 to 5.3)	**-58.5<br>(-65.46 to -41.08)**	35.7<br>(23.3 to 51.3)	13.7<br>(8.8 to 21.5)	**-61.6<br>(-68.10 to -45.79)**	3.9<br>(3.1 to 5.5)	1.9<br>(1.2 to 2.6)	**-51.8<br>(-63.37 to -36.44)**	18.2<br>(14.3 to 25.9)	8.0<br>(5.2 to 10.9)	**-55.6<br>(-66.42 to -41.36)**
**Forces of nature, war, and legal intervention**	0.1<br>(0.0 to 0.4)	--	**-100.0<br>(-100.00 to -100.00)**	0.3<br>(0.1 to 1.0)	--	**-100.0<br>(-100.00 to -100.00)**	0.1<br>(0.0 to 0.2)	--	**-100.0<br>(-100.00 to -100.00)**	0.3<br>(0.0 to 1.4)	--	**-100.0<br>(-100.00 to -100.00)**	<0.05<br>(0.0 to 0.2)	--	**-100.0<br>(-100.00 to -100.00)**	0.2<br>(0.1 to 1.2)	--	**-100.0<br>(-100.00 to -100.00)**
Collective violence and legal intervention	0.1<br>(0.0 to 0.4)	--	**-100.0<br>(-100.00 to -100.00)**	0.3<br>(0.1 to 1.0)	--	**-100.0<br>(-100.00 to -100.00)**	0.1<br>(0.0 to 0.2)	--	**-100.0<br>(-100.00 to -100.00)**	0.3<br>(0.0 to 1.4)	--	**-100.0<br>(-100.00 to -100.00)**	<0.05<br>(0.0 to 0.2)	--	**-100.0<br>(-100.00 to -100.00)**	0.2<br>(0.1 to 1.2)	--	**-100.0<br>(-100.00 to -100.00)**

Web Table 6 : YLDs in England in 1990 and 2013 for all ages and age-standardized rates (per 100,000) by sex with 95% UI and percentage change. Percent changes in bold denote a statistically significant change.

Both sexes combined

Cause	YLDs (in thousands) 1990	YLDs (in thousands) 2013	YLDs (in thousands) % change	Age-standardized rate (per 100,000) 1990	Age-standardized rate (per 100,000) 2013	Age-standardized rate (per 100,000) % change
**All causes**	6,154.3 (4,609.0 to 7,885.7)	7,138.1 (5,339.5 to 9,166.7)	**16.0 (14.34 to 17.58)**	11,346.0 (8,474.7 to 14,613.8)	11,185.5 (8,321.8 to 14,423.8)	**-1.4 (-2.78 to -0.09)**
**Communicable, maternal, neonatal, and nutritional diseases**	247.4 (172.7 to 343.8)	284.2 (199.6 to 394.8)	**15.0 (8.25 to 20.82)**	587.0 (407.7 to 818.5)	614.8 (430.1 to 859.6)	4.9 (-0.94 to 9.69)
**HIV/AIDS and tuberculosis**	4.0 (2.6 to 5.6)	5.0 (3.5 to 6.9)	**27.7 (6.42 to 53.07)**	8.0 (5.3 to 11.3)	8.9 (6.1 to 12.2)	12.2 (-5.74 to 33.43)
Tuberculosis	2.7 (1.8 to 3.7)	3.4 (2.3 to 4.5)	**24.4 (16.07 to 33.43)**	5.6 (3.8 to 7.5)	6.2 (4.2 to 8.3)	**12.1 (4.07 to 20.52)**
HIV/AIDS	1.2 (0.5 to 2.2)	1.7 (0.9 to 2.8)	42.6 (-20.51 to 148.20)	2.4 (1.0 to 4.3)	2.7 (1.4 to 4.5)	18.6 (-33.43 to 105.62)
HIV/AIDS resulting in mycobacterial infection	0.1 (0.0 to 0.1)	<0.05 (0.0 to 0.1)	-20.5 (-58.06 to 31.87)	0.1 (0.0 to 0.2)	0.1 (0.0 to 0.1)	-30.2 (-63.02 to 15.11)
HIV/AIDS resulting in other diseases	1.2 (0.5 to 2.1)	1.6 (0.8 to 2.7)	46.5 (-19.83 to 166.65)	2.3 (0.9 to 4.2)	2.6 (1.3 to 4.4)	21.7 (-33.54 to 119.22)
**Diarrhea, lower respiratory, and other common infectious diseases**	28.2 (17.9 to 41.7)	29.3 (18.8 to 43.4)	**3.8 (1.32 to 6.36)**	60.1 (38.0 to 89.8)	55.3 (35.3 to 82.7)	**-8.0 (-10.28 to -5.68)**
Diarrheal diseases	2.6 (1.8 to 3.5)	2.6 (1.8 to 3.6)	3.3 (-4.06 to 11.51)	5.5 (3.8 to 7.6)	5.1 (3.5 to 7.1)	**-7.5 (-15.12 to 1.14)**
Intestinal infectious diseases	0.1 (0.0 to 0.1)	<0.05 (0.0 to 0.1)	**-30.4 (-47.76 to -16.46)**	0.1 (0.1 to 0.2)	0.1 (0.1 to 0.1)	**-35.8 (-51.80 to -22.60)**
Typhoid fever	<0.05 (0.0 to 0.1)	<0.05 (0.0 to <0.05)	**-26.3 (-35.17 to -17.29)**	0.1 (0.1 to 0.1)	0.1 (0.0 to 0.1)	**-32.3 (-40.89 to -23.36)**
Paratyphoid fever	<0.05 (0.0 to <0.05)	<0.05 (0.0 to <0.05)	5.1 (-8.02 to 20.83)	<0.05 (0.0 to <0.05)	<0.05 (0.0 to <0.05)	-1.4 (-13.42 to 13.38)
Other intestinal infectious diseases	<0.05 (0.0 to <0.05)	<0.05 (0.0 to <0.05)	**-62.5 (-93.42 to 6.78)**	<0.05 (0.0 to 0.1)	<0.05 (0.0 to <0.05)	**-65.6 (-93.92 to -3.47)**
Lower respiratory infections	1.4 (0.9 to 2.0)	1.4 (0.9 to 1.9)	-1.6 (-14.50 to 11.54)	2.1 (1.4 to 3.0)	1.6 (1.1 to 2.2)	**-22.5 (-31.74 to -13.47)**
Upper respiratory infections	8.4 (4.7 to 13.8)	9.3 (5.2 to 15.4)	**11.1 (8.66 to 13.79)**	18.4 (10.3 to 30.4)	18.6 (10.4 to 30.8)	1.0 (-1.45 to 3.30)
Otitis media	11.0 (6.4 to 17.7)	10.8 (6.3 to 17.1)	-2.2 (-8.00 to 3.37)	23.8 (14.0 to 38.5)	20.6 (12.1 to 32.9)	**-13.5 (-18.54 to -9.16)**
Meningitis	2.5 (1.7 to 3.5)	2.6 (1.8 to 3.6)	6.3 (-3.75 to 18.55)	5.3 (3.6 to 7.3)	5.0 (3.4 to 6.9)	-5.5 (-15.08 to 6.27)
Pneumococcal meningitis	0.7 (0.5 to 0.9)	0.7 (0.5 to 0.9)	5.2 (-7.40 to 18.36)	1.4 (1.0 to 1.9)	1.3 (0.9 to 1.7)	-8.5 (-19.93 to 4.24)
H influenzae type B meningitis	0.5 (0.3 to 0.8)	0.6 (0.4 to 0.8)	14.2 (-12.80 to 42.77)	1.2 (0.8 to 1.7)	1.2 (0.8 to 1.7)	3.6 (-22.06 to 30.20)
Meningococcal meningitis	0.5 (0.3 to 0.8)	0.5 (0.3 to 0.8)	5.6 (-15.32 to 28.33)	1.0 (0.6 to 1.6)	0.9 (0.6 to 1.4)	-7.3 (-27.01 to 13.43)
Other meningitis	0.8 (0.5 to 1.2)	0.8 (0.5 to 1.2)	3.3 (-13.58 to 23.47)	1.7 (1.2 to 2.5)	1.6 (1.0 to 2.3)	-7.3 (-22.85 to 11.87)
Encephalitis	0.3 (0.2 to 0.4)	0.4 (0.3 to 0.6)	**38.1 (25.12 to 50.57)**	0.6 (0.4 to 0.8)	0.7 (0.5 to 1.0)	**18.9 (6.92 to 30.32)**
Diphtheria	<0.05 (0.0 to <0.05)	<0.05 (0.0 to <0.05)	**-45.2 (-77.81 to 7.55)**	<0.05 (0.0 to <0.05)	<0.05 (0.0 to <0.05)	**-53.6 (-81.64 to -4.85)**
Whooping cough	0.4 (0.2 to 0.6)	0.2 (0.1 to 0.3)	**-46.5 (-50.19 to -42.56)**	1.0 (0.6 to 1.7)	0.5 (0.3 to 0.9)	**-47.8 (-51.42 to -43.93)**
Tetanus	<0.05 (0.0 to <0.05)	<0.05 (0.0 to <0.05)	-15.3 (-47.62 to 2.51)	<0.05 (0.0 to <0.05)	<0.05 (0.0 to <0.05)	**-23.4 (-53.73 to -3.99)**
Measles	0.1 (0.0 to 0.1)	<0.05 (0.0 to <0.05)	**-93.0 (-93.92 to -91.89)**	0.2 (0.1 to 0.2)	<0.05 (0.0 to <0.05)	**-93.2 (-94.07 to -92.09)**
Varicella and herpes zoster	1.6 (1.0 to 2.5)	1.9 (1.2 to 2.9)	**19.0 (8.27 to 30.79)**	3.0 (1.9 to 4.6)	3.0 (1.9 to 4.6)	0.7 (-8.43 to 10.63)
**Neglected tropical diseases and malaria**	0.4 (0.3 to 0.6)	0.3 (0.2 to 0.4)	**-38.3 (-44.55 to -31.98)**	0.8 (0.6 to 1.1)	0.4 (0.3 to 0.6)	**-48.6 (-53.63 to -43.91)**
Malaria	<0.05 (0.0 to <0.05)	<0.05 (0.0 to <0.05)	23.1 (-22.85 to 90.73)	<0.05 (0.0 to <0.05)	<0.05 (0.0 to <0.05)	12.3 (-30.25 to 74.48)
Cysticercosis	0.1 (0.1 to 0.1)	<0.05 (0.0 to <0.05)	**-67.6 (-80.54 to -53.19)**	0.2 (0.1 to 0.2)	<0.05 (0.0 to 0.1)	**-72.7 (-83.68 to -61.18)**
Cystic echinococcosis	0.3 (0.2 to 0.5)	0.2 (0.2 to 0.3)	**-32.0 (-37.43 to -26.14)**	0.6 (0.4 to 0.9)	0.4 (0.2 to 0.5)	**-43.9 (-48.13 to -39.44)**
Rabies	<0.05 (0.0 to <0.05)	<0.05 (0.0 to <0.05)	-11.7 (-50.68 to 11.50)	<0.05 (0.0 to <0.05)	<0.05 (0.0 to <0.05)	**-22.7 (-57.32 to -2.49)**
Other neglected tropical diseases	<0.05 (0.0 to <0.05)	<0.05 (0.0 to <0.05)	**298.8 (72.34 to 418.63)**	<0.05 (0.0 to <0.05)	<0.05 (0.0 to <0.05)	**251.1 (49.63 to 357.26)**
**Maternal disorders**	4.1 (2.7 to 6.0)	4.2 (2.7 to 6.1)	1.2 (-8.52 to 10.34)	8.6 (5.6 to 12.5)	8.6 (5.6 to 12.5)	0.7 (-8.83 to 9.90)
Maternal hemorrhage	2.3 (1.5 to 3.3)	2.2 (1.5 to 3.2)	-2.3 (-15.47 to 9.04)	4.7 (3.1 to 6.8)	4.6 (3.1 to 6.6)	-2.4 (-15.45 to 8.98)
Maternal sepsis and other maternal infections	<0.05 (0.0 to 0.1)	<0.05 (0.0 to 0.1)	0.5 (-15.50 to 23.87)	0.1 (0.0 to 0.1)	0.1 (0.0 to 0.1)	-3.2 (-18.24 to 16.94)
Maternal hypertensive disorders	0.9 (0.5 to 1.5)	1.0 (0.5 to 1.6)	5.6 (-2.81 to 14.63)	1.9 (1.0 to 3.0)	2.0 (1.1 to 3.3)	4.2 (-3.68 to 12.58)
Obstructed labor	0.1 (0.0 to 0.1)	0.1 (0.0 to 0.1)	**8.7 (5.28 to 11.84)**	0.1 (0.1 to 0.2)	0.1 (0.1 to 0.2)	**7.8 (4.48 to 11.01)**
Complications of abortion	<0.05 (0.0 to <0.05)	<0.05 (0.0 to <0.05)	**16.3 (13.00 to 19.69)**	<0.05 (0.0 to <0.05)	<0.05 (0.0 to <0.05)	**14.7 (11.39 to 17.95)**
Other maternal disorders	0.9 (0.5 to 1.2)	0.9 (0.6 to 1.3)	5.8 (-12.43 to 25.50)	1.8 (1.1 to 2.6)	1.9 (1.2 to 2.7)	5.3 (-12.72 to 24.91)
**Neonatal disorders**	52.7 (38.7 to 68.3)	76.7 (56.1 to 98.4)	**46.1 (19.53 to 68.36)**	117.7 (86.2 to 153.0)	153.5 (112.1 to 197.6)	**30.9 (6.77 to 50.76)**
Preterm birth complications	34.7 (24.9 to 45.4)	58.9 (42.3 to 76.0)	**70.5 (37.07 to 102.74)**	77.1 (55.3 to 101.1)	117.5 (83.9 to 151.7)	**53.2 (22.42 to 81.57)**
Neonatal encephalopathy due to birth asphyxia and trauma	10.5 (7.8 to 13.2)	8.8 (6.5 to 11.3)	**-16.4 (-29.40 to -2.13)**	24.0 (17.8 to 30.2)	18.1 (13.3 to 23.3)	**-24.6 (-36.51 to -11.62)**
Neonatal sepsis and other neonatal infections	<0.05 (0.0 to <0.05)	<0.05 (0.0 to <0.05)	**82.4 (32.14 to 156.37)**	<0.05 (0.0 to <0.05)	<0.05 (0.0 to <0.05)	**78.3 (29.15 to 150.57)**
Hemolytic disease and other neonatal jaundice	4.9 (2.9 to 8.3)	4.1 (2.5 to 6.0)	-12.2 (-57.40 to 43.87)	10.9 (6.4 to 18.5)	8.1 (5.0 to 12.1)	-21.8 (-62.55 to 26.96)
Other neonatal disorders	2.6 (1.7 to 3.8)	4.9 (3.2 to 7.3)	**96.6 (38.38 to 178.31)**	5.7 (3.8 to 8.4)	9.9 (6.3 to 14.6)	**76.1 (24.12 to 149.62)**
**Nutritional deficiencies**	145.3 (95.8 to 212.2)	154.7 (100.8 to 227.3)	**6.5 (0.77 to 11.17)**	362.4 (239.6 to 529.8)	357.9 (235.1 to 525.8)	-1.2 (-5.96 to 2.52)
Protein-energy malnutrition	<0.05 (0.0 to 0.1)	<0.05 (0.0 to <0.05)	-18.1 (-52.64 to 21.51)	<0.05 (0.0 to 0.1)	<0.05 (0.0 to 0.1)	**-40.9 (-64.36 to -14.49)**
Iodine deficiency	16.0 (9.3 to 25.9)	10.9 (6.2 to 18.1)	**-30.8 (-51.72 to -4.37)**	32.1 (18.7 to 52.0)	19.8 (11.3 to 32.8)	**-37.7 (-56.15 to -13.82)**
Iron-deficiency anemia	129.2 (85.3 to 190.1)	143.7 (94.3 to 212.0)	**11.2 (5.79 to 14.66)**	330.3 (218.3 to 485.3)	338.1 (223.1 to 495.5)	2.4 (-2.14 to 5.44)
Other nutritional deficiencies	<0.05 (0.0 to 0.1)	<0.05 (0.0 to <0.05)	**-63.3 (-79.51 to -21.67)**	0.1 (0.0 to 0.1)	<0.05 (0.0 to <0.05)	**-73.6 (-84.67 to -45.17)**
**Other communicable, maternal, neonatal, and nutritional diseases**	12.7 (7.9 to 20.4)	14.0 (8.5 to 22.8)	**10.6 (-2.56 to 30.17)**	29.5 (18.6 to 46.8)	30.3 (18.5 to 48.9)	2.8 (-12.19 to 26.06)
Sexually transmitted diseases excluding HIV	5.7 (3.0 to 10.6)	6.3 (3.5 to 11.5)	**11.0 (4.56 to 18.77)**	11.4 (6.1 to 21.2)	11.6 (6.3 to 21.2)	1.4 (-4.96 to 9.15)
Syphilis	0.4 (0.2 to 0.5)	0.6 (0.4 to 0.9)	**70.7 (50.08 to 96.30)**	0.6 (0.4 to 0.9)	0.9 (0.6 to 1.2)	**39.3 (22.22 to 62.19)**
Chlamydial infection	2.7 (1.4 to 4.8)	2.8 (1.5 to 5.1)	5.2 (-6.45 to 19.70)	5.6 (3.0 to 10.1)	5.7 (3.0 to 10.2)	0.6 (-11.20 to 14.87)
Gonococcal infection	0.6 (0.3 to 1.1)	0.6 (0.3 to 1.1)	-2.3 (-13.46 to 11.10)	1.3 (0.7 to 2.4)	1.3 (0.7 to 2.3)	-3.6 (-14.95 to 10.44)
Trichomoniasis	0.2 (0.1 to 0.5)	0.3 (0.1 to 0.6)	13.5 (-9.77 to 34.02)	0.5 (0.2 to 1.0)	0.5 (0.2 to 1.1)	7.5 (-15.58 to 27.40)
Genital herpes	1.6 (0.5 to 3.8)	1.8 (0.5 to 4.3)	**11.0 (3.85 to 18.32)**	2.9 (0.9 to 7.1)	2.8 (0.9 to 6.8)	-4.8 (-10.79 to 1.36)

Males

Cause	YLDs (in thousands) 1990	YLDs (in thousands) 2013	YLDs (in thousands) % change	Age-standardized rate (per 100,000) 1990	Age-standardized rate (per 100,000) 2013	Age-standardized rate (per 100,000) % change
**All causes**	2,762.5 (2,068.4 to 3,563.8)	3,302.0 (2,480.0 to 4,249.4)	**19.5 (17.50 to 21.44)**	11,006.3 (8,234.7 to 14,220.4)	10,772.1 (8,061.4 to 13,914.7)	**-2.1 (-3.77 to -0.62)**
**Communicable, maternal, neonatal, and nutritional diseases**	123.6 (87.2 to 170.8)	143.6 (101.2 to 196.5)	**16.4 (5.46 to 25.31)**	600.4 (422.2 to 832.1)	632.7 (443.4 to 865.8)	5.5 (-3.70 to 12.83)
**HIV/AIDS and tuberculosis**	2.5 (1.6 to 3.6)	3.2 (2.2 to 4.4)	**31.2 (4.22 to 62.83)**	9.9 (6.4 to 14.4)	11.0 (7.6 to 15.4)	12.6 (-10.13 to 38.82)
Tuberculosis	1.5 (1.0 to 2.0)	2.0 (1.3 to 2.7)	**34.2 (22.99 to 46.49)**	6.1 (4.1 to 8.3)	7.1 (4.8 to 9.6)	**17.6 (7.32 to 28.77)**
HIV/AIDS	1.0 (0.4 to 1.8)	1.2 (0.6 to 2.1)	33.1 (-26.84 to 132.96)	3.8 (1.5 to 6.9)	3.9 (2.0 to 6.7)	9.7 (-39.37 to 91.81)
HIV/AIDS resulting in mycobacterial infection	<0.05 (0.0 to 0.1)	<0.05 (0.0 to 0.1)	-23.6 (-60.18 to 26.55)	0.2 (0.1 to 0.3)	0.1 (0.0 to 0.2)	-34.0 (-65.67 to 10.08)
HIV/AIDS resulting in other diseases	0.9 (0.3 to 1.7)	1.2 (0.6 to 2.0)	37.0 (-26.33 to 151.01)	3.7 (1.4 to 6.7)	3.8 (2.0 to 6.5)	12.8 (-39.11 to 106.74)
**Diarrhea, lower respiratory, and other common infectious diseases**	14.2 (9.2 to 21.2)	14.8 (9.5 to 21.7)	**3.7 (-0.11 to 7.66)**	62.4 (40.2 to 92.9)	56.6 (36.3 to 84.2)	**-9.3 (-12.74 to -5.92)**
Diarrheal diseases	1.5 (1.0 to 2.1)	1.6 (1.1 to 2.2)	6.0 (-3.92 to 16.93)	7.0 (4.7 to 9.6)	6.4 (4.4 to 8.9)	-8.0 (-18.05 to 3.32)
Intestinal infectious diseases	<0.05 (0.0 to <0.05)	<0.05 (0.0 to <0.05)	**-22.9 (-47.85 to -0.88)**	0.1 (0.1 to 0.2)	0.1 (0.1 to 0.1)	**-30.2 (-52.78 to -9.79)**
Typhoid fever	<0.05 (0.0 to <0.05)	<0.05 (0.0 to <0.05)	**-18.5 (-33.11 to -2.96)**	0.1 (0.1 to 0.1)	0.1 (0.0 to 0.1)	**-26.6 (-40.32 to -12.56)**
Paratyphoid fever	<0.05 (0.0 to <0.05)	<0.05 (0.0 to <0.05)	**33.3 (9.47 to 61.86)**	<0.05 (0.0 to <0.05)	<0.05 (0.0 to <0.05)	**22.3 (0.14 to 49.99)**
Other intestinal infectious diseases	<0.05 (0.0 to <0.05)	<0.05 (0.0 to <0.05)	-54.4 (-96.53 to 89.28)	<0.05 (0.0 to 0.1)	<0.05 (0.0 to <0.05)	-58.9 (-96.95 to 67.99)
Lower respiratory infections	0.6 (0.4 to 0.8)	0.6 (0.4 to 0.8)	4.4 (-13.18 to 21.85)	2.2 (1.4 to 3.2)	1.6 (1.0 to 2.3)	**-27.1 (-38.18 to -16.41)**
Upper respiratory infections	4.0 (2.3 to 6.6)	4.6 (2.6 to 7.6)	**13.2 (9.38 to 17.01)**	17.9 (10.0 to 29.5)	18.2 (10.2 to 30.6)	1.9 (-1.65 to 5.41)
Otitis media	5.8 (3.4 to 9.3)	5.6 (3.3 to 8.9)	-3.2 (-11.87 to 5.63)	25.1 (14.8 to 40.6)	21.4 (12.5 to 34.4)	**-14.6 (-21.86 to -7.63)**
Meningitis	1.3 (0.9 to 1.8)	1.3 (0.8 to 1.7)	-1.6 (-14.12 to 13.53)	5.6 (3.8 to 7.9)	4.9 (3.3 to 6.8)	-11.9 (-23.45 to 2.23)
Pneumococcal meningitis	0.3 (0.2 to 0.5)	0.3 (0.2 to 0.5)	0.8 (-19.87 to 19.87)	1.4 (1.0 to 2.0)	1.3 (0.9 to 1.8)	-11.3 (-30.75 to 6.80)
H influenzae type B meningitis	0.3 (0.2 to 0.4)	0.3 (0.2 to 0.5)	7.2 (-27.04 to 49.06)	1.3 (0.8 to 2.0)	1.2 (0.8 to 1.9)	-2.2 (-34.11 to 37.00)
Meningococcal meningitis	0.2 (0.1 to 0.4)	0.2 (0.1 to 0.3)	-5.9 (-29.41 to 20.54)	1.0 (0.6 to 1.6)	0.8 (0.5 to 1.3)	-17.1 (-38.90 to 6.97)
Other meningitis	0.4 (0.3 to 0.6)	0.4 (0.3 to 0.6)	-5.3 (-26.71 to 21.78)	1.8 (1.2 to 2.7)	1.6 (1.0 to 2.2)	-14.4 (-33.70 to 11.25)
Encephalitis	0.2 (0.1 to 0.2)	0.2 (0.1 to 0.3)	**31.5 (17.64 to 46.69)**	0.7 (0.5 to 0.9)	0.7 (0.5 to 1.0)	**10.6 (-1.35 to 24.10)**
Diphtheria	<0.05 (0.0 to <0.05)	<0.05 (0.0 to <0.05)	-35.1 (-80.70 to 55.23)	<0.05 (0.0 to <0.05)	<0.05 (0.0 to <0.05)	-45.6 (-84.82 to 33.04)
Whooping cough	0.2 (0.1 to 0.3)	0.1 (0.1 to 0.1)	**-46.3 (-51.80 to -40.47)**	0.9 (0.5 to 1.5)	0.5 (0.3 to 0.8)	**-47.7 (-53.04 to -41.94)**
Tetanus	<0.05 (0.0 to <0.05)	<0.05 (0.0 to <0.05)	-15.8 (-59.76 to 5.64)	<0.05 (0.0 to <0.05)	<0.05 (0.0 to <0.05)	**-25.3 (-64.19 to -4.31)**
Measles	<0.05 (0.0 to <0.05)	<0.05 (0.0 to <0.05)	**-93.0 (-94.26 to -91.18)**	0.2 (0.1 to 0.2)	<0.05 (0.0 to <0.05)	**-93.1 (-94.41 to -91.40)**
Varicella and herpes zoster	0.7 (0.4 to 1.1)	0.8 (0.5 to 1.3)	**22.4 (5.73 to 40.28)**	2.8 (1.7 to 4.3)	2.8 (1.7 to 4.2)	0.1 (-13.67 to 14.34)
**Neglected tropical diseases and malaria**	0.2 (0.2 to 0.3)	0.2 (0.1 to 0.2)	**-28.9 (-39.31 to -18.38)**	0.9 (0.6 to 1.2)	0.5 (0.3 to 0.7)	**-42.6 (-50.76 to -34.68)**
Malaria	<0.05 (0.0 to <0.05)	<0.05 (0.0 to <0.05)	27.4 (-45.01 to 154.24)	<0.05 (0.0 to <0.05)	<0.05 (0.0 to <0.05)	16.5 (-49.73 to 131.75)
Cysticercosis	<0.05 (0.0 to 0.1)	<0.05 (0.0 to <0.05)	**-84.0 (-91.55 to -74.74)**	0.2 (0.1 to 0.3)	<0.05 (0.0 to <0.05)	**-86.3 (-92.70 to -78.51)**
Cystic echinococcosis	0.2 (0.1 to 0.2)	0.1 (0.1 to 0.2)	**-14.6 (-24.64 to -4.22)**	0.7 (0.5 to 0.9)	0.5 (0.3 to 0.6)	**-32.1 (-39.75 to -24.59)**
Rabies	<0.05 (0.0 to <0.05)	<0.05 (0.0 to <0.05)	-14.3 (-55.29 to 10.28)	<0.05 (0.0 to <0.05)	<0.05 (0.0 to <0.05)	**-25.8 (-61.48 to -4.08)**
Other neglected tropical diseases	<0.05 (0.0 to <0.05)	<0.05 (0.0 to <0.05)	**364.8 (131.59 to 531.13)**	<0.05 (0.0 to <0.05)	<0.05 (0.0 to <0.05)	**303.5 (96.63 to 448.98)**
**Maternal disorders**	--	--	--	--	--	--
Maternal hemorrhage	--	--	--	--	--	--
Maternal sepsis and other maternal infections	--	--	--	--	--	--
Maternal hypertensive disorders	--	--	--	--	--	--
Obstructed labor	--	--	--	--	--	--
Complications of abortion	--	--	--	--	--	--
Other maternal disorders	--	--	--	--	--	--
**Neonatal disorders**	27.9 (19.9 to 37.8)	38.8 (27.6 to 52.3)	**40.9 (-4.02 to 81.35)**	125.1 (89.2 to 169.9)	156.1 (110.3 to 210.4)	26.2 (-14.19 to 62.57)
Preterm birth complications	18.0 (12.6 to 24.3)	29.5 (20.5 to 40.0)	**65.3 (12.39 to 111.49)**	80.5 (56.1 to 108.7)	117.9 (82.0 to 160.4)	**48.3 (0.31 to 90.03)**
Neonatal encephalopathy due to birth asphyxia and trauma	5.5 (4.0 to 7.1)	4.6 (3.3 to 6.1)	-16.0 (-32.96 to 4.28)	25.2 (18.4 to 32.7)	19.0 (13.6 to 25.3)	**-24.0 (-39.69 to -4.98)**
Neonatal sepsis and other neonatal infections	<0.05 (0.0 to <0.05)	<0.05 (0.0 to <0.05)	**79.4 (22.34 to 162.83)**	<0.05 (0.0 to <0.05)	<0.05 (0.0 to <0.05)	**75.5 (19.65 to 157.04)**
Hemolytic disease and other neonatal jaundice	2.9 (1.6 to 6.5)	2.1 (1.2 to 3.4)	-16.4 (-76.21 to 43.29)	13.1 (7.2 to 29.3)	8.5 (4.6 to 13.7)	-25.5 (-78.78 to 28.09)
Other neonatal disorders	1.4 (0.8 to 2.3)	2.6 (1.5 to 4.4)	**91.3 (15.78 to 223.07)**	6.4 (3.8 to 10.3)	10.6 (6.2 to 17.5)	**71.3 (3.33 to 189.12)**
**Nutritional deficiencies**	70.9 (46.9 to 103.6)	77.9 (51.2 to 113.9)	**9.9 (7.04 to 13.66)**	364.7 (242.0 to 529.5)	370.2 (244.2 to 539.6)	1.5 (-0.65 to 4.20)
Protein-energy malnutrition	<0.05 (0.0 to <0.05)	<0.05 (0.0 to <0.05)	10.4 (-36.64 to 78.61)	<0.05 (0.0 to 0.1)	<0.05 (0.0 to 0.1)	-27.6 (-56.56 to 13.55)
Iodine deficiency	4.9 (2.7 to 8.3)	3.4 (1.9 to 6.3)	-28.7 (-54.03 to 22.09)	20.2 (11.0 to 34.0)	12.6 (6.8 to 23.1)	**-36.3 (-59.30 to 9.28)**
Iron-deficiency anemia	65.9 (43.7 to 96.2)	74.4 (49.2 to 109.0)	**12.9 (11.39 to 14.66)**	344.4 (228.8 to 499.8)	357.6 (237.7 to 518.7)	**3.8 (2.73 to 4.95)**
Other nutritional deficiencies	<0.05 (0.0 to <0.05)	<0.05 (0.0 to <0.05)	-47.0 (-73.05 to 21.46)	<0.05 (0.0 to 0.1)	<0.05 (0.0 to <0.05)	**-65.2 (-82.11 to -21.69)**
**Other communicable, maternal, neonatal, and nutritional diseases**	7.9 (5.0 to 12.3)	8.8 (5.6 to 13.6)	**11.0 (4.87 to 17.35)**	37.4 (23.7 to 57.6)	38.2 (24.8 to 58.2)	2.4 (-2.93 to 8.20)
Sexually transmitted diseases excluding HIV	3.3 (1.9 to 5.6)	3.7 (2.2 to 6.3)	**13.2 (2.94 to 24.08)**	13.4 (7.6 to 23.1)	13.8 (8.0 to 23.6)	3.1 (-6.35 to 13.47)
Syphilis	0.2 (0.2 to 0.4)	0.4 (0.3 to 0.6)	**83.2 (53.68 to 120.92)**	0.9 (0.6 to 1.3)	1.3 (0.9 to 1.8)	**44.2 (21.37 to 74.06)**
Chlamydial infection	2.1 (1.2 to 3.5)	2.2 (1.2 to 3.8)	7.2 (-7.30 to 23.27)	8.6 (4.9 to 14.9)	8.8 (4.8 to 15.3)	2.2 (-12.08 to 18.12)
Gonococcal infection	0.4 (0.2 to 0.6)	0.4 (0.2 to 0.6)	-3.2 (-15.95 to 12.74)	1.5 (0.8 to 2.6)	1.5 (0.8 to 2.5)	-5.5 (-18.39 to 10.54)
Trichomoniasis	--	--	--	--	--	--
Genital herpes	0.6 (0.2 to 1.5)	0.7 (0.2 to 1.7)	**14.9 (2.48 to 26.11)**	2.3 (0.7 to 5.7)	2.2 (0.7 to 5.3)	-4.2 (-14.27 to 4.76)

Females

Cause	YLDs (in thousands) 1990	YLDs (in thousands) 2013	YLDs (in thousands) % change	Age-standardized rate (per 100,000) 1990	Age-standardized rate (per 100,000) 2013	Age-standardized rate (per 100,000) % change
**All causes**	3,391.8 (2,535.3 to 4,345.8)	3,836.2 (2,864.8 to 4,918.2)	**13.1 (10.96 to 15.19)**	11,674.3 (8,692.1 to 15,028.7)	11,599.5 (8,600.8 to 14,953.7)	-0.7 (-2.56 to 0.98)
**Communicable, maternal, neonatal, and nutritional diseases**	123.8 (85.5 to 174.7)	140.5 (98.5 to 197.3)	**13.7 (5.52 to 22.27)**	572.1 (393.5 to 807.4)	595.6 (415.8 to 839.4)	4.3 (-2.77 to 11.60)
**HIV/AIDS and tuberculosis**	1.5 (1.0 to 2.1)	1.9 (1.3 to 2.5)	**22.5 (6.71 to 41.33)**	6.1 (4.2 to 8.4)	6.8 (4.7 to 9.3)	11.0 (-2.76 to 27.06)
Tuberculosis	1.2 (0.8 to 1.7)	1.4 (1.0 to 1.9)	**12.9 (1.73 to 24.72)**	5.1 (3.4 to 6.9)	5.3 (3.7 to 7.2)	4.9 (-6.17 to 16.30)
HIV/AIDS	0.3 (0.1 to 0.5)	0.4 (0.2 to 0.8)	**77.5 (0.43 to 205.33)**	1.0 (0.4 to 1.9)	1.4 (0.7 to 2.5)	49.5 (-15.00 to 156.21)
HIV/AIDS resulting in mycobacterial infection	<0.05 (0.0 to <0.05)	<0.05 (0.0 to <0.05)	-4.9 (-49.69 to 60.14)	<0.05 (0.0 to 0.1)	<0.05 (0.0 to 0.1)	-13.8 (-54.05 to 45.00)
HIV/AIDS resulting in other diseases	0.3 (0.1 to 0.5)	0.4 (0.2 to 0.7)	**81.3 (0.45 to 219.80)**	1.0 (0.4 to 1.9)	1.4 (0.7 to 2.4)	52.5 (-14.26 to 166.27)
**Diarrhea, lower respiratory, and other common infectious diseases**	14.0 (8.9 to 20.6)	14.5 (9.5 to 21.4)	**4.0 (0.48 to 7.92)**	57.9 (36.3 to 86.3)	53.9 (34.6 to 80.3)	**-6.8 (-10.21 to -3.29)**
Diarrheal diseases	1.0 (0.7 to 1.4)	1.0 (0.7 to 1.4)	-0.6 (-11.05 to 11.12)	4.1 (2.8 to 5.7)	3.8 (2.6 to 5.4)	-6.8 (-17.27 to 5.24)
Intestinal infectious diseases	<0.05 (0.0 to <0.05)	<0.05 (0.0 to <0.05)	**-36.4 (-57.40 to -17.89)**	0.1 (0.1 to 0.2)	0.1 (0.0 to 0.1)	**-40.4 (-60.81 to -22.55)**
Typhoid fever	<0.05 (0.0 to <0.05)	<0.05 (0.0 to <0.05)	**-33.0 (-44.69 to -21.72)**	0.1 (0.1 to 0.1)	0.1 (0.0 to 0.1)	**-37.5 (-48.12 to -27.33)**
Paratyphoid fever	<0.05 (0.0 to <0.05)	<0.05 (0.0 to <0.05)	**-17.7 (-31.53 to 0.97)**	<0.05 (0.0 to <0.05)	<0.05 (0.0 to <0.05)	**-21.1 (-34.11 to -4.82)**
Other intestinal infectious diseases	<0.05 (0.0 to <0.05)	<0.05 (0.0 to <0.05)	-51.2 (-97.19 to 122.88)	<0.05 (0.0 to 0.1)	<0.05 (0.0 to <0.05)	-54.2 (-97.41 to 101.81)
Lower respiratory infections	0.8 (0.6 to 1.2)	0.8 (0.5 to 1.1)	-5.2 (-22.75 to 14.69)	2.0 (1.4 to 2.8)	1.6 (1.1 to 2.2)	**-19.3 (-33.13 to -3.13)**
Upper respiratory infections	4.4 (2.5 to 7.2)	4.8 (2.7 to 7.8)	**9.2 (5.68 to 12.89)**	18.9 (10.6 to 31.3)	19.0 (10.6 to 31.2)	0.2 (-3.13 to 3.50)
Otitis media	5.2 (3.1 to 8.4)	5.2 (3.0 to 8.2)	-1.1 (-8.12 to 6.50)	22.7 (13.2 to 36.4)	19.8 (11.6 to 31.8)	**-12.5 (-18.55 to -6.02)**
Meningitis	1.2 (0.8 to 1.7)	1.4 (0.9 to 1.9)	**15.1 (-0.10 to 32.32)**	4.9 (3.4 to 6.9)	5.0 (3.4 to 6.9)	2.0 (-12.80 to 18.05)
Pneumococcal meningitis	0.3 (0.2 to 0.5)	0.4 (0.2 to 0.5)	10.2 (-5.88 to 30.70)	1.3 (0.9 to 1.8)	1.3 (0.9 to 1.7)	-5.2 (-20.08 to 13.29)
H influenzae type B meningitis	0.2 (0.2 to 0.4)	0.3 (0.2 to 0.5)	25.3 (-14.53 to 90.61)	1.1 (0.7 to 1.6)	1.2 (0.7 to 1.8)	13.1 (-23.77 to 73.95)
Meningococcal meningitis	0.2 (0.1 to 0.4)	0.3 (0.2 to 0.4)	19.1 (-11.18 to 64.47)	0.9 (0.5 to 1.5)	0.9 (0.6 to 1.5)	5.1 (-23.45 to 47.17)
Other meningitis	0.4 (0.3 to 0.6)	0.4 (0.3 to 0.6)	13.2 (-10.69 to 40.49)	1.6 (1.1 to 2.3)	1.6 (1.1 to 2.4)	1.2 (-21.56 to 25.66)
Encephalitis	0.2 (0.1 to 0.2)	0.2 (0.2 to 0.3)	**45.0 (27.10 to 65.01)**	0.6 (0.4 to 0.8)	0.7 (0.5 to 1.0)	**28.0 (10.54 to 46.64)**
Diphtheria	<0.05 (0.0 to <0.05)	<0.05 (0.0 to <0.05)	-47.3 (-85.98 to 34.96)	<0.05 (0.0 to <0.05)	<0.05 (0.0 to <0.05)	-54.3 (-88.10 to 23.00)
Whooping cough	0.2 (0.1 to 0.3)	0.1 (0.1 to 0.2)	**-46.6 (-51.22 to -41.27)**	1.1 (0.7 to 1.9)	0.6 (0.3 to 1.0)	**-47.9 (-52.38 to -42.61)**
Tetanus	<0.05 (0.0 to <0.05)	<0.05 (0.0 to <0.05)	-14.5 (-44.93 to 6.27)	<0.05 (0.0 to <0.05)	<0.05 (0.0 to <0.05)	**-20.9 (-50.74 to 1.80)**
Measles	<0.05 (0.0 to <0.05)	<0.05 (0.0 to <0.05)	**-93.0 (-94.21 to -91.41)**	0.2 (0.1 to 0.2)	<0.05 (0.0 to <0.05)	**-93.1 (-94.35 to -91.60)**
Varicella and herpes zoster	0.9 (0.6 to 1.4)	1.1 (0.7 to 1.7)	**16.7 (0.90 to 34.44)**	3.2 (2.0 to 5.0)	3.3 (2.0 to 5.1)	1.8 (-12.07 to 17.62)
**Neglected tropical diseases and malaria**	0.2 (0.2 to 0.3)	0.1 (0.1 to 0.2)	**-47.7 (-54.60 to -39.03)**	0.7 (0.5 to 1.0)	0.3 (0.2 to 0.4)	**-55.3 (-60.87 to -48.41)**
Malaria	<0.05 (0.0 to <0.05)	<0.05 (0.0 to <0.05)	25.6 (-32.57 to 139.04)	<0.05 (0.0 to <0.05)	<0.05 (0.0 to <0.05)	14.4 (-38.78 to 119.97)
Cysticercosis	<0.05 (0.0 to 0.1)	<0.05 (0.0 to <0.05)	**-46.6 (-66.67 to -11.17)**	0.1 (0.1 to 0.2)	0.1 (0.0 to 0.1)	**-54.8 (-73.11 to -24.17)**
Cystic echinococcosis	0.2 (0.1 to 0.2)	0.1 (0.1 to 0.1)	**-49.1 (-55.51 to -41.53)**	0.6 (0.4 to 0.8)	0.2 (0.2 to 0.3)	**-57.1 (-61.45 to -51.61)**
Rabies	<0.05 (0.0 to <0.05)	<0.05 (0.0 to <0.05)	-8.6 (-49.76 to 12.04)	<0.05 (0.0 to <0.05)	<0.05 (0.0 to <0.05)	**-18.9 (-55.74 to -0.17)**
Other neglected tropical diseases	<0.05 (0.0 to <0.05)	<0.05 (0.0 to <0.05)	**233.0 (-39.33 to 369.14)**	<0.05 (0.0 to <0.05)	<0.05 (0.0 to <0.05)	**196.8 (-45.89 to 314.95)**
**Maternal disorders**	4.1 (2.7 to 6.0)	4.2 (2.7 to 6.1)	1.2 (-8.52 to 10.34)	17.2 (11.2 to 25.1)	17.3 (11.3 to 25.1)	0.9 (-8.63 to 10.14)
Maternal hemorrhage	2.3 (1.5 to 3.3)	2.2 (1.5 to 3.2)	-2.3 (-15.47 to 9.04)	9.4 (6.3 to 13.7)	9.2 (6.2 to 13.2)	-2.2 (-15.27 to 9.28)
Maternal sepsis and other maternal infections	<0.05 (0.0 to 0.1)	<0.05 (0.0 to 0.1)	0.5 (-15.50 to 23.87)	0.1 (0.1 to 0.2)	0.1 (0.1 to 0.2)	-3.3 (-18.23 to 16.72)
Maternal hypertensive disorders	0.9 (0.5 to 1.5)	1.0 (0.5 to 1.6)	5.6 (-2.81 to 14.63)	3.8 (2.1 to 6.1)	4.0 (2.2 to 6.6)	4.3 (-3.53 to 12.70)
Obstructed labor	0.1 (0.0 to 0.1)	0.1 (0.0 to 0.1)	**8.7 (5.28 to 11.84)**	0.2 (0.1 to 0.4)	0.2 (0.1 to 0.4)	**8.0 (4.64 to 11.19)**
Complications of abortion	<0.05 (0.0 to <0.05)	<0.05 (0.0 to <0.05)	**16.3 (13.00 to 19.69)**	<0.05 (0.0 to 0.1)	<0.05 (0.0 to 0.1)	**14.8 (11.50 to 18.07)**
Other maternal disorders	0.9 (0.5 to 1.2)	0.9 (0.6 to 1.3)	5.8 (-12.43 to 25.50)	3.5 (2.3 to 5.2)	3.7 (2.4 to 5.5)	5.5 (-12.52 to 25.16)
**Neonatal disorders**	24.8 (18.1 to 32.0)	37.9 (26.4 to 49.8)	**53.1 (28.10 to 82.86)**	110.0 (79.9 to 141.9)	150.7 (104.8 to 199.0)	**37.4 (14.55 to 64.56)**
Preterm birth complications	16.7 (11.9 to 22.2)	29.5 (20.2 to 39.0)	**77.6 (39.59 to 118.70)**	73.5 (52.2 to 97.9)	116.9 (79.9 to 155.0)	**60.0 (25.34 to 97.56)**
Neonatal encephalopathy due to birth asphyxia and trauma	5.0 (3.6 to 6.5)	4.2 (3.0 to 5.5)	-16.2 (-35.08 to 4.14)	22.7 (16.6 to 29.5)	17.0 (12.2 to 22.6)	**-24.6 (-41.68 to -5.91)**
Neonatal sepsis and other neonatal infections	<0.05 (0.0 to <0.05)	<0.05 (0.0 to <0.05)	**88.2 (38.69 to 153.97)**	<0.05 (0.0 to <0.05)	<0.05 (0.0 to <0.05)	**83.8 (35.46 to 148.06)**
Hemolytic disease and other neonatal jaundice	2.0 (1.1 to 3.7)	1.9 (1.0 to 3.3)	6.3 (-49.66 to 112.32)	8.7 (4.7 to 16.3)	7.7 (3.8 to 13.0)	-4.8 (-55.15 to 89.60)
Other neonatal disorders	1.1 (0.7 to 1.9)	2.3 (1.3 to 3.6)	**108.2 (6.19 to 210.35)**	5.0 (3.1 to 8.5)	9.1 (5.3 to 14.4)	**86.9 (-4.72 to 179.17)**
**Nutritional deficiencies**	74.4 (48.6 to 109.4)	76.8 (49.2 to 113.0)	3.3 (-7.25 to 11.12)	358.9 (235.2 to 528.0)	344.4 (221.1 to 505.4)	-4.0 (-13.68 to 2.55)
Protein-energy malnutrition	<0.05 (0.0 to <0.05)	<0.05 (0.0 to <0.05)	-30.5 (-68.58 to 17.08)	0.1 (0.0 to 0.1)	<0.05 (0.0 to 0.1)	**-46.5 (-72.73 to -15.77)**
Iodine deficiency	11.0 (6.2 to 18.3)	7.5 (4.2 to 12.8)	**-30.7 (-55.00 to 3.30)**	43.7 (24.6 to 72.1)	27.0 (15.2 to 46.0)	**-37.1 (-59.12 to -6.78)**
Iron-deficiency anemia	63.3 (41.6 to 93.3)	69.3 (44.8 to 101.8)	**9.4 (-1.45 to 15.80)**	315.0 (207.8 to 466.2)	317.4 (205.3 to 466.0)	0.8 (-8.73 to 6.75)
Other nutritional deficiencies	<0.05 (0.0 to 0.1)	<0.05 (0.0 to <0.05)	**-69.2 (-85.89 to -32.03)**	0.1 (0.0 to 0.1)	<0.05 (0.0 to <0.05)	**-76.5 (-88.22 to -49.25)**
**Other communicable, maternal, neonatal, and nutritional diseases**	4.7 (2.7 to 8.2)	5.2 (2.7 to 9.3)	10.2 (-20.59 to 62.81)	21.4 (12.2 to 36.6)	22.1 (11.4 to 40.6)	4.3 (-31.69 to 72.30)
Sexually transmitted diseases excluding HIV	2.4 (1.1 to 5.0)	2.6 (1.2 to 5.3)	**8.0 (0.97 to 15.56)**	9.4 (4.4 to 19.1)	9.3 (4.4 to 18.9)	-0.9 (-7.66 to 6.28)
Syphilis	0.1 (0.1 to 0.2)	0.2 (0.1 to 0.3)	**48.7 (24.97 to 78.08)**	0.4 (0.3 to 0.6)	0.5 (0.3 to 0.7)	**26.5 (6.01 to 51.71)**
Chlamydial infection	0.6 (0.3 to 1.3)	0.6 (0.3 to 1.3)	-1.0 (-19.54 to 15.09)	2.6 (1.2 to 5.3)	2.5 (1.1 to 5.1)	-4.9 (-23.61 to 10.95)
Gonococcal infection	0.3 (0.1 to 0.5)	0.2 (0.1 to 0.5)	-0.7 (-18.07 to 23.45)	1.1 (0.5 to 2.2)	1.1 (0.5 to 2.3)	-0.2 (-18.71 to 25.43)
Trichomoniasis	0.2 (0.1 to 0.5)	0.3 (0.1 to 0.6)	13.5 (-9.77 to 34.02)	1.0 (0.4 to 2.0)	1.0 (0.4 to 2.2)	7.6 (-15.64 to 27.63)
Genital herpes	1.0 (0.3 to 2.4)	1.1 (0.3 to 2.6)	**8.8 (-0.12 to 18.83)**	3.5 (1.1 to 8.4)	3.3 (1.0 to 8.2)	-4.6 (-12.51 to 4.13)

Other sexually transmitted diseases	0.2 (0.1 to 0.3)	0.2 (0.2 to 0.3)	8.7 (-3.73 to 21.80)	0.4 (0.3 to 0.6)	0.4 (0.3 to 0.6)	2.2 (-9.93 to 14.90)	--	--	--	--	--	--	0.2 (0.1 to 0.3)	0.2 (0.2 to 0.3)	8.7 (-3.73 to 21.80)	0.9 (0.6 to 1.2)	0.9 (0.6 to 1.2)	2.4 (-9.82 to 15.30)
Hepatitis	1.5 (1.0 to 2.2)	1.5 (0.9 to 2.1)	**-4.2 (-9.26 to 0.27)**	3.2 (2.1 to 4.7)	2.7 (1.8 to 4.0)	**-14.3 (-18.51 to -10.36)**	0.8 (0.5 to 1.2)	0.8 (0.5 to 1.1)	**-5.7 (-12.44 to 0.92)**	3.4 (2.2 to 5.0)	2.9 (1.9 to 4.2)	**-16.4 (-22.13 to -10.65)**	0.7 (0.5 to 1.1)	0.7 (0.5 to 1.0)	-2.5 (-7.98 to 3.46)	2.9 (1.9 to 4.3)	2.6 (1.7 to 3.8)	**-12.0 (-17.05 to -6.44)**
Hepatitis A	1.0 (0.7 to 1.5)	1.1 (0.7 to 1.6)	3.3 (-1.19 to 7.53)	2.2 (1.4 to 3.3)	2.1 (1.3 to 3.0)	**-6.9 (-10.91 to -2.79)**	0.5 (0.3 to 0.8)	0.5 (0.3 to 0.8)	3.9 (-2.10 to 9.90)	2.2 (1.4 to 3.3)	2.1 (1.3 to 3.1)	**-6.8 (-12.35 to -1.42)**	0.5 (0.3 to 0.8)	0.5 (0.4 to 0.8)	2.7 (-3.43 to 8.76)	2.2 (1.4 to 3.3)	2.1 (1.3 to 3.0)	**-6.9 (-12.66 to -1.17)**
Hepatitis B	0.4 (0.2 to 0.6)	0.3 (0.2 to 0.4)	**-22.2 (-34.57 to -10.44)**	0.7 (0.5 to 1.1)	0.5 (0.3 to 0.7)	**-33.2 (-43.52 to -22.99)**	0.2 (0.2 to 0.4)	0.2 (0.1 to 0.3)	**-23.5 (-39.19 to -5.53)**	1.0 (0.6 to 1.5)	0.6 (0.4 to 1.0)	**-34.9 (-47.99 to -19.49)**	0.1 (0.1 to 0.2)	0.1 (0.1 to 0.2)	**-19.2 (-36.01 to 1.78)**	0.5 (0.3 to 0.7)	0.3 (0.2 to 0.5)	**-29.9 (-43.98 to -12.49)**
Hepatitis C	0.1 (0.1 to 0.2)	0.1 (0.1 to 0.1)	**-12.6 (-23.32 to -0.72)**	0.2 (0.1 to 0.3)	0.2 (0.1 to 0.2)	**-26.7 (-34.51 to -18.35)**	0.1 (0.0 to 0.1)	<0.05 (0.0 to 0.1)	**-14.3 (-26.82 to 1.75)**	0.2 (0.1 to 0.3)	0.2 (0.1 to 0.2)	**-29.7 (-39.26 to -18.40)**	0.1 (0.0 to 0.1)	0.1 (0.0 to 0.1)	-10.7 (-27.23 to 8.41)	0.2 (0.1 to 0.3)	0.2 (0.1 to 0.2)	**-23.5 (-35.70 to -9.81)**
Leprosy	--	<0.05 (0.0 to <0.05)	--	--	<0.05 (0.0 to <0.05)	--	--	<0.05 (0.0 to <0.05)	--	--	<0.05 (0.0 to <0.05)	--	--	<0.05 (0.0 to <0.05)	--	--	<0.05 (0.0 to <0.05)	--
Other infectious diseases	5.5 (3.5 to 8.2)	6.2 (3.8 to 9.9)	14.7 (-12.49 to 59.08)	14.9 (9.6 to 22.3)	16.0 (9.8 to 25.5)	7.9 (-19.30 to 52.02)	3.9 (2.5 to 5.7)	4.4 (2.8 to 6.4)	**13.0 (5.06 to 22.21)**	20.5 (13.4 to 30.0)	21.6 (14.2 to 31.3)	5.3 (-1.41 to 13.51)	1.6 (0.9 to 3.0)	1.9 (0.8 to 4.3)	22.9 (-47.57 to 178.21)	9.0 (5.1 to 17.0)	10.2 (4.4 to 23.9)	18.4 (-51.35 to 176.43)
**Non-communicable diseases**	5,355.2 (3,984.1 to 6,861.6)	6,411.9 (4,789.5 to 8,246.4)	**19.7 (17.92 to 21.53)**	9,854.7 (7,323.5 to 12,662.1)	9,982.4 (7,427.3 to 12,886.6)	**1.3 (-0.22 to 2.80)**	2,357.3 (1,759.6 to 3,037.8)	2,912.8 (2,174.9 to 3,745.3)	**23.6 (21.23 to 25.89)**	9,314.8 (6,946.4 to 12,021.4)	9,404.7 (6,985.8 to 12,100.7)	1.0 (-0.92 to 2.92)	2,998.0 (2,225.6 to 3,856.1)	3,499.0 (2,604.4 to 4,478.9)	**16.7 (14.19 to 18.97)**	10,406.9 (7,699.8 to 13,390.8)	10,563.1 (7,820.5 to 13,621.1)	1.5 (-0.66 to 3.32)
**Neoplasms**	92.8 (69.7 to 117.8)	126.7 (94.8 to 162.5)	**36.6 (27.27 to 45.24)**	143.2 (107.5 to 182.8)	152.8 (113.5 to 197.5)	**6.7 (-0.53 to 13.22)**	43.9 (32.9 to 55.1)	65.0 (48.0 to 82.9)	**48.1 (31.00 to 62.37)**	156.2 (116.8 to 196.2)	168.3 (124.4 to 216.2)	7.8 (-4.45 to 18.25)	48.8 (36.2 to 62.9)	61.7 (44.9 to 80.8)	**26.4 (14.86 to 37.10)**	139.2 (102.8 to 181.7)	142.3 (102.5 to 186.6)	2.2 (-6.41 to 10.52)
Esophageal cancer	1.3 (0.9 to 1.7)	1.8 (1.3 to 2.5)	**37.0 (10.12 to 71.34)**	2.0 (1.4 to 2.6)	2.1 (1.5 to 2.9)	7.1 (-13.73 to 32.43)	0.8 (0.5 to 1.0)	1.2 (0.8 to 1.7)	**56.3 (15.99 to 106.35)**	2.7 (1.8 to 3.6)	3.1 (2.0 to 4.5)	14.0 (-15.00 to 50.34)	0.6 (0.4 to 0.8)	0.6 (0.4 to 0.9)	12.3 (-18.38 to 53.05)	1.4 (1.0 to 1.9)	1.3 (0.8 to 1.8)	-8.4 (-31.92 to 21.88)
Stomach cancer	3.1 (2.3 to 4.0)	2.3 (1.7 to 3.1)	**-24.7 (-34.91 to -13.02)**	4.5 (3.3 to 5.9)	2.6 (1.9 to 3.4)	**-42.1 (-49.31 to -33.49)**	1.9 (1.4 to 2.5)	1.4 (1.0 to 1.8)	**-27.3 (-38.68 to -13.42)**	6.8 (4.9 to 8.7)	3.5 (2.5 to 4.6)	**-48.0 (-56.11 to -38.44)**	1.2 (0.8 to 1.7)	1.0 (0.6 to 1.4)	**-20.1 (-36.92 to 2.53)**	2.9 (1.9 to 4.1)	1.8 (1.2 to 2.6)	**-35.2 (-48.53 to -17.41)**
Liver cancer	0.4 (0.2 to 0.5)	0.8 (0.5 to 1.1)	**121.2 (48.01 to 192.66)**	0.5 (0.4 to 0.8)	0.9 (0.6 to 1.3)	**72.3 (18.86 to 126.55)**	0.2 (0.1 to 0.3)	0.5 (0.3 to 0.7)	**123.4 (31.46 to 218.29)**	0.8 (0.5 to 1.2)	1.2 (0.7 to 1.8)	**64.0 (-2.47 to 132.74)**	0.1 (0.1 to 0.2)	0.3 (0.2 to 0.4)	**122.1 (26.98 to 218.30)**	0.4 (0.2 to 0.5)	0.6 (0.4 to 0.9)	**77.8 (8.03 to 150.93)**
Liver cancer due to hepatitis B	0.1 (0.0 to 0.1)	0.2 (0.1 to 0.2)	**123.1 (44.43 to 220.93)**	0.1 (0.1 to 0.2)	0.2 (0.1 to 0.3)	**75.3 (16.71 to 149.90)**	<0.05 (0.0 to 0.1)	0.1 (0.1 to 0.2)	**110.1 (10.74 to 222.31)**	0.2 (0.1 to 0.3)	0.3 (0.1 to 0.4)	60.0 (-14.43 to 143.14)	<0.05 (0.0 to <0.05)	0.1 (0.0 to 0.1)	**154.6 (26.78 to 319.49)**	0.1 (0.0 to 0.1)	0.1 (0.1 to 0.2)	**100.3 (5.40 to 220.65)**
Liver cancer due to hepatitis C	0.1 (0.1 to 0.1)	0.4 (0.2 to 0.6)	**319.9 (174.51 to 497.35)**	0.1 (0.1 to 0.2)	0.4 (0.3 to 0.6)	**230.0 (121.10 to 368.18)**	0.1 (0.0 to 0.1)	0.2 (0.1 to 0.3)	**343.2 (128.75 to 624.33)**	0.2 (0.1 to 0.3)	0.6 (0.3 to 0.9)	**224.8 (70.00 to 422.36)**	<0.05 (0.0 to 0.1)	0.2 (0.1 to 0.3)	**304.5 (126.08 to 516.53)**	0.1 (0.1 to 0.2)	0.3 (0.2 to 0.5)	**229.0 (86.26 to 400.81)**
Liver cancer due to alcohol use	0.1 (0.1 to 0.2)	0.2 (0.1 to 0.3)	31.0 (-19.46 to 87.11)	0.2 (0.1 to 0.3)	0.2 (0.1 to 0.3)	1.1 (-38.17 to 44.12)	0.1 (0.1 to 0.2)	0.1 (0.1 to 0.2)	42.1 (-19.95 to 119.61)	0.4 (0.2 to 0.6)	0.4 (0.2 to 0.6)	2.4 (-42.44 to 57.38)	<0.05 (0.0 to 0.1)	<0.05 (0.0 to 0.1)	8.1 (-45.02 to 73.44)	0.1 (0.1 to 0.2)	0.1 (0.0 to 0.1)	-14.9 (-53.64 to 33.46)
Liver cancer due to other causes	<0.05 (0.0 to 0.1)	<0.05 (0.0 to 0.1)	-4.3 (-42.45 to 41.78)	0.1 (0.0 to 0.1)	<0.05 (0.0 to 0.1)	-25.9 (-53.54 to 7.91)	<0.05 (0.0 to <0.05)	<0.05 (0.0 to <0.05)	-25.5 (-62.99 to 21.34)	<0.05 (0.0 to 0.1)	<0.05 (0.0 to <0.05)	**-44.2 (-71.90 to -9.66)**	<0.05 (0.0 to <0.05)	<0.05 (0.0 to <0.05)	7.6 (-45.14 to 78.77)	0.1 (0.0 to 0.1)	0.1 (0.0 to 0.1)	-13.4 (-53.09 to 41.20)
Larynx cancer	0.9 (0.6 to 1.2)	0.8 (0.6 to 1.2)	-5.8 (-25.32 to 14.95)	1.4 (1.0 to 2.0)	1.0 (0.7 to 1.5)	**-26.9 (-41.40 to -11.56)**	0.7 (0.5 to 1.0)	0.7 (0.4 to 1.0)	-9.7 (-31.85 to 14.01)	2.6 (1.7 to 3.7)	1.8 (1.1 to 2.5)	**-32.8 (-48.81 to -16.18)**	0.2 (0.1 to 0.2)	0.2 (0.1 to 0.3)	13.0 (-20.31 to 56.94)	0.5 (0.3 to 0.7)	0.4 (0.2 to 0.7)	-8.3 (-34.29 to 25.93)
Tracheal, bronchus and lung cancer	9.0 (6.6 to 11.4)	8.7 (6.4 to 11.0)	-3.3 (-15.60 to 9.51)	13.4 (9.8 to 17.0)	10.1 (7.4 to 12.9)	**-24.3 (-33.70 to -14.65)**	5.9 (4.3 to 7.5)	4.7 (3.3 to 6.1)	**-20.9 (-33.74 to -8.02)**	20.7 (15.1 to 26.3)	11.9 (8.4 to 15.5)	**-42.4 (-51.67 to -33.00)**	3.0 (2.2 to 4.0)	4.0 (2.8 to 5.2)	**31.9 (-3.74 to 57.64)**	8.1 (5.9 to 10.5)	8.6 (6.0 to 11.3)	7.6 (-18.54 to 28.16)
Breast cancer	18.4 (13.2 to 25.0)	23.0 (16.1 to 31.7)	**25.0 (13.25 to 36.74)**	29.1 (20.9 to 39.3)	28.0 (19.6 to 38.6)	-3.8 (-12.96 to 5.47)	0.1 (0.1 to 0.2)	0.1 (0.1 to 0.2)	**42.0 (6.93 to 96.71)**	0.4 (0.2 to 0.6)	0.4 (0.2 to 0.6)	3.4 (-21.64 to 42.70)	18.3 (13.1 to 24.8)	22.9 (16.0 to 31.5)	**25.0 (13.20 to 36.63)**	53.1 (38.0 to 71.7)	52.6 (36.7 to 72.4)	-1.0 (-10.65 to 8.63)
Cervical cancer	1.8 (1.2 to 2.5)	1.2 (0.8 to 1.7)	**-34.1 (-47.07 to -18.44)**	3.3 (2.2 to 4.6)	1.8 (1.2 to 2.6)	**-44.5 (-55.88 to -30.42)**	--	--	--	--	--	--	1.8 (1.2 to 2.5)	1.2 (0.8 to 1.7)	**-34.1 (-47.07 to -18.44)**	6.4 (4.3 to 8.9)	3.6 (2.3 to 5.1)	**-43.8 (-55.56 to -29.35)**
Uterine cancer	1.9 (1.3 to 2.7)	2.8 (1.8 to 4.1)	**51.9 (3.73 to 102.36)**	3.0 (2.0 to 4.4)	3.5 (2.3 to 5.1)	19.2 (-19.07 to 59.88)	--	--	--	--	--	--	1.9 (1.3 to 2.7)	2.8 (1.8 to 4.1)	**51.9 (3.73 to 102.36)**	5.6 (3.6 to 8.1)	6.8 (4.3 to 9.8)	22.9 (-16.54 to 65.45)
Prostate cancer	12.2 (8.8 to 16.3)	24.2 (16.8 to 33.0)	**100.3 (44.07 to 142.25)**	17.1 (12.3 to 22.9)	26.8 (18.4 to 36.7)	**58.1 (13.03 to 90.21)**	12.2 (8.8 to 16.3)	24.2 (16.8 to 33.0)	**100.3 (44.07 to 142.25)**	42.1 (30.4 to 56.9)	59.6 (41.1 to 81.5)	**42.6 (2.52 to 72.19)**	--	--	--	--	--	--
Colon and rectum cancer	12.6 (9.4 to 16.2)	15.2 (11.2 to 19.9)	**21.0 (8.66 to 33.59)**	18.6 (13.9 to 24.1)	17.5 (12.8 to 22.9)	-5.7 (-15.17 to 4.06)	6.3 (4.7 to 8.2)	8.6 (6.3 to 11.3)	**36.7 (20.43 to 54.50)**	22.3 (16.7 to 29.1)	22.1 (16.2 to 29.0)	-1.1 (-12.58 to 11.61)	6.3 (4.6 to 8.1)	6.6 (4.7 to 8.6)	5.3 (-10.70 to 21.53)	15.8 (11.6 to 20.5)	13.5 (9.7 to 17.8)	**-14.4 (-26.68 to -1.77)**
Lip and oral cavity cancer	1.3 (0.9 to 1.8)	1.5 (1.1 to 2.1)	19.7 (-10.53 to 51.00)	2.0 (1.4 to 2.8)	1.9 (1.3 to 2.7)	-4.5 (-27.67 to 20.67)	0.8 (0.6 to 1.1)	0.9 (0.6 to 1.3)	14.1 (-26.12 to 58.19)	3.0 (2.1 to 4.1)	2.5 (1.6 to 3.6)	-14.3 (-44.91 to 18.17)	0.5 (0.3 to 0.7)	0.6 (0.4 to 0.9)	32.0 (-7.95 to 85.92)	1.3 (0.9 to 1.8)	1.4 (0.9 to 2.0)	9.2 (-22.13 to 49.22)
Nasopharynx cancer	0.2 (0.1 to 0.3)	0.1 (0.1 to 0.2)	**-32.7 (-50.76 to -8.91)**	0.3 (0.2 to 0.4)	0.2 (0.1 to 0.2)	**-45.6 (-59.47 to -27.73)**	0.1 (0.1 to 0.2)	0.1 (0.0 to 0.1)	**-30.8 (-51.46 to -2.75)**	0.4 (0.3 to 0.6)	0.2 (0.1 to 0.3)	**-45.3 (-60.97 to -23.40)**	0.1 (0.0 to 0.1)	<0.05 (0.0 to 0.1)	**-34.2 (-61.93 to 6.06)**	0.2 (0.1 to 0.4)	0.1 (0.0 to 0.2)	**-45.5 (-67.17 to -14.64)**
Other pharynx cancer	0.6 (0.4 to 0.9)	0.7 (0.5 to 1.1)	27.4 (-17.79 to 84.18)	1.0 (0.6 to 1.4)	1.0 (0.6 to 1.4)	1.4 (-34.30 to 46.59)	0.4 (0.3 to 0.7)	0.5 (0.3 to 0.8)	29.2 (-26.82 to 98.03)	1.5 (1.0 to 2.4)	1.5 (0.9 to 2.3)	-1.7 (-43.66 to 48.73)	0.2 (0.1 to 0.3)	0.2 (0.1 to 0.3)	28.2 (-29.86 to 117.14)	0.5 (0.3 to 0.8)	0.5 (0.3 to 0.8)	7.8 (-41.65 to 84.46)
Gallbladder and biliary tract cancer	0.4 (0.2 to 0.5)	0.3 (0.2 to 0.5)	-11.1 (-30.72 to 18.02)	0.5 (0.3 to 0.7)	0.3 (0.2 to 0.5)	**-31.5 (-45.60 to -9.93)**	0.1 (0.1 to 0.2)	0.1 (0.1 to 0.2)	-9.9 (-36.30 to 31.22)	0.4 (0.3 to 0.6)	0.3 (0.2 to 0.4)	**-36.2 (-54.31 to -7.77)**	0.2 (0.2 to 0.3)	0.2 (0.1 to 0.3)	-11.2 (-35.53 to 22.89)	0.6 (0.4 to 0.8)	0.4 (0.3 to 0.6)	**-27.1 (-45.03 to -2.26)**
Pancreatic cancer	1.2 (0.9 to 1.6)	1.6 (1.1 to 2.1)	**27.2 (9.82 to 46.27)**	1.8 (1.3 to 2.4)	1.8 (1.3 to 2.4)	-0.3 (-13.23 to 13.72)	0.6 (0.4 to 0.8)	0.8 (0.5 to 1.0)	**29.3 (6.11 to 56.85)**	2.1 (1.5 to 2.8)	2.0 (1.4 to 2.7)	-6.1 (-23.01 to 13.81)	0.6 (0.4 to 0.8)	0.8 (0.6 to 1.1)	**25.8 (0.09 to 53.55)**	1.6 (1.1 to 2.1)	1.7 (1.1 to 2.2)	4.4 (-16.27 to 26.85)
Malignant skin melanoma	2.5 (1.6 to 3.8)	4.0 (2.4 to 6.0)	**62.5 (22.27 to 97.17)**	4.3 (2.7 to 6.6)	5.5 (3.4 to 8.4)	**30.0 (-0.35 to 57.22)**	0.9 (0.5 to 1.7)	1.9 (0.8 to 3.4)	**105.8 (-0.03 to 171.75)**	3.4 (1.8 to 6.5)	5.3 (2.3 to 9.6)	**57.0 (-22.23 to 105.71)**	1.6 (1.0 to 2.4)	2.2 (1.3 to 3.3)	**38.6 (7.04 to 74.62)**	5.1 (3.1 to 7.9)	5.8 (3.4 to 8.9)	14.3 (-11.26 to 42.51)
Non-melanoma skin cancer	1.0 (0.6 to 1.5)	1.9 (1.2 to 2.9)	**90.8 (48.00 to 149.22)**	1.4 (0.9 to 2.1)	2.1 (1.3 to 3.1)	**48.1 (17.41 to 89.32)**	0.5 (0.3 to 0.8)	0.9 (0.5 to 1.6)	**97.2 (40.40 to 166.11)**	1.7 (1.0 to 2.8)	2.4 (1.3 to 3.8)	**37.5 (-1.01 to 84.81)**	0.5 (0.3 to 0.8)	0.9 (0.5 to 1.5)	**86.8 (22.84 to 182.92)**	1.2 (0.7 to 1.8)	1.8 (1.1 to 2.9)	**55.3 (6.70 to 122.25)**
Ovarian cancer	3.1 (2.2 to 4.0)	3.4 (2.4 to 4.5)	11.3 (-9.23 to 34.95)	5.0 (3.6 to 6.5)	4.3 (3.0 to 5.7)	-13.2 (-28.93 to 5.04)	--	--	--	--	--	--	3.1 (2.2 to 4.0)	3.4 (2.4 to 4.5)	11.3 (-9.23 to 34.95)	9.3 (6.6 to 12.1)	8.2 (5.7 to 10.9)	-11.2 (-27.20 to 7.27)
Testicular cancer	0.6 (0.4 to 1.0)	0.7 (0.4 to 1.0)	7.9 (-25.34 to 60.67)	1.3 (0.7 to 1.9)	1.2 (0.7 to 1.9)	-1.5 (-32.49 to 48.31)	0.6 (0.4 to 1.0)	0.7 (0.4 to 1.0)	7.9 (-25.34 to 60.67)	2.5 (1.5 to 3.9)	2.4 (1.4 to 3.8)	-1.9 (-32.66 to 47.49)	--	--	--	--	--	--
Kidney cancer	2.1 (1.5 to 2.8)	3.2 (2.3 to 4.4)	**56.7 (32.40 to 82.28)**	3.3 (2.4 to 4.5)	4.0 (2.9 to 5.4)	**22.0 (3.01 to 42.12)**	1.2 (0.9 to 1.6)	2.0 (1.4 to 2.7)	**63.4 (29.71 to 102.23)**	4.4 (3.1 to 5.9)	5.3 (3.7 to 7.2)	**21.4 (-3.38 to 49.24)**	0.8 (0.6 to 1.2)	1.2 (0.8 to 1.7)	**47.9 (12.22 to 92.70)**	2.4 (1.6 to 3.5)	2.9 (1.9 to 4.0)	20.1 (-6.29 to 50.41)
Bladder cancer	5.0 (3.5 to 6.7)	5.2 (3.7 to 7.0)	4.4 (-12.02 to 23.19)	7.3 (5.1 to 9.7)	5.8 (4.1 to 7.8)	**-19.7 (-32.01 to -5.68)**	3.6 (2.5 to 4.9)	3.7 (2.5 to 5.0)	3.3 (-17.55 to 28.97)	12.7 (8.6 to 17.2)	9.3 (6.3 to 12.6)	**-26.4 (-41.04 to -8.59)**	1.4 (0.8 to 2.0)	1.5 (0.9 to 2.2)	8.6 (-18.84 to 42.36)	3.3 (2.1 to 4.9)	2.9 (1.8 to 4.3)	-11.9 (-34.10 to 16.95)
Brain and nervous system cancer	1.3 (0.9 to 1.7)	1.8 (1.2 to 2.4)	**39.0 (19.22 to 61.62)**	2.3 (1.6 to 3.1)	2.6 (1.9 to 3.5)	12.1 (-2.75 to 28.42)	0.7 (0.5 to 1.0)	1.0 (0.6 to 1.3)	**34.0 (9.07 to 62.59)**	2.8 (1.8 to 3.8)	2.9 (2.0 to 4.0)	5.9 (-12.39 to 26.55)	0.6 (0.4 to 0.8)	0.8 (0.5 to 1.1)	**46.1 (16.30 to 79.78)**	1.9 (1.3 to 2.7)	2.3 (1.6 to 3.2)	**19.8 (-2.24 to 46.05)**
Thyroid cancer	0.7 (0.5 to 1.0)	0.8 (0.5 to 1.2)	19.5 (-5.87 to 49.18)	1.2 (0.8 to 1.7)	1.2 (0.8 to 1.7)	0.7 (-21.58 to 24.73)	0.2 (0.1 to 0.2)	0.2 (0.2 to 0.3)	**44.3 (-1.22 to 93.63)**	0.6 (0.4 to 0.9)	0.7 (0.5 to 1.0)	15.1 (-20.46 to 52.72)	0.5 (0.3 to 0.8)	0.6 (0.4 to 0.9)	11.8 (-17.80 to 46.26)	1.7 (1.1 to 2.5)	1.6 (1.0 to 2.5)	-2.7 (-29.29 to 26.81)
Mesothelioma	0.2 (0.1 to 0.4)	0.6 (0.3 to 0.8)	**236.0 (23.14 to 338.67)**	0.3 (0.2 to 0.5)	0.7 (0.3 to 0.9)	**155.0 (-1.86 to 231.61)**	0.1 (0.1 to 0.3)	0.5 (0.2 to 0.7)	**310.9 (15.19 to 457.84)**	0.4 (0.3 to 1.0)	1.2 (0.5 to 1.7)	196.1 (-14.56 to 301.38)	0.1 (0.0 to 0.1)	0.1 (0.1 to 0.2)	**90.8 (4.16 to 131.60)**	0.2 (0.1 to 0.3)	0.2 (0.1 to 0.3)	53.2 (-12.39 to 84.77)
Hodgkin lymphoma	0.7 (0.5 to 1.0)	0.7 (0.4 to 1.0)	-2.8 (-27.86 to 32.55)	1.4 (0.9 to 2.0)	1.2 (0.8 to 1.8)	-13.5 (-36.74 to 18.63)	0.4 (0.2 to 0.6)	0.4 (0.2 to 0.6)	-1.5 (-34.04 to 45.23)	1.6 (1.0 to 2.4)	1.4 (0.8 to 2.1)	-14.6 (-42.90 to 25.67)	0.3 (0.2 to 0.5)	0.3 (0.2 to 0.5)	-2.6 (-35.64 to 45.22)	1.2 (0.7 to 1.8)	1.0 (0.6 to 1.6)	-10.8 (-42.63 to 35.46)
Non-Hodgkin lymphoma	2.8 (1.9 to 4.1)	4.4 (2.6 to 6.2)	**59.6 (-10.93 to 97.52)**	4.5 (3.0 to 6.6)	5.5 (3.3 to 7.8)	24.6 (-29.82 to 54.08)	1.5 (1.0 to 2.3)	2.4 (1.2 to 3.3)	64.0 (-22.22 to 112.39)	5.4 (3.5 to 8.4)	6.5 (3.2 to 9.2)	22.3 (-41.05 to 59.50)	1.3 (0.8 to 2.1)	2.0 (1.1 to 3.1)	56.6 (-9.04 to 104.58)	3.8 (2.4 to 5.9)	4.7 (2.5 to 7.3)	27.2 (-25.68 to 66.11)
Multiple myeloma	1.7 (1.1 to 2.6)	2.6 (1.6 to 3.8)	**49.3 (5.40 to 101.68)**	2.6 (1.6 to 3.9)	3.0 (1.8 to 4.5)	17.3 (-17.15 to 55.89)	0.9 (0.4 to 1.5)	1.4 (0.7 to 2.2)	**65.6 (0.59 to 145.79)**	3.1 (1.6 to 5.2)	3.6 (1.8 to 5.8)	19.9 (-27.09 to 76.69)	0.9 (0.5 to 1.4)	1.2 (0.6 to 2.0)	37.0 (-13.07 to 113.19)	2.2 (1.2 to 3.6)	2.5 (1.3 to 4.2)	14.5 (-27.04 to 71.45)
Leukemia	2.9 (2.1 to 3.8)	4.4 (3.1 to 5.7)	**49.6 (23.42 to 77.92)**	4.8 (3.4 to 6.2)	5.5 (3.9 to 7.2)	14.7 (-4.76 to 35.38)	1.6 (1.1 to 2.1)	2.7 (1.8 to 3.6)	**64.6 (27.58 to 106.37)**	6.1 (4.4 to 8.0)	7.3 (5.0 to 9.8)	19.1 (-7.01 to 48.02)	1.3 (0.9 to 1.8)	1.7 (1.1 to 2.4)	**32.0 (-4.33 to 72.92)**	3.8 (2.7 to 5.1)	4.0 (2.6 to 5.4)	4.8 (-22.52 to 34.93)
Other neoplasms	3.1 (2.1 to 4.1)	8.0 (5.3 to 11.0)	**162.3 (99.38 to 233.63)**	5.2 (3.6 to 7.1)	10.5 (7.1 to 14.4)	**102.5 (58.21 to 149.97)**	1.4 (0.9 to 2.0)	3.5 (2.2 to 5.0)	**152.5 (73.94 to 231.65)**	5.4 (3.6 to 7.8)	10.0 (6.5 to 14.1)	**85.9 (29.84 to 140.81)**	1.7 (1.2 to 2.3)	4.5 (2.8 to 6.6)	**172.6 (92.59 to 278.14)**	5.0 (3.5 to 6.9)	11.0 (7.1 to 15.8)	**118.9 (64.60 to 190.68)**
**Cardiovascular diseases**	243.2 (170.6 to 322.6)	318.7 (222.5 to 419.0)	**31.5 (12.23 to 51.44)**	360.6 (252.9 to 481.5)	373.9 (261.0 to 493.3)	4.1 (-11.71 to 20.76)	130.8 (92.3 to 172.5)	170.7 (123.0 to 225.2)	**31.0 (14.33 to 50.12)**	465.9 (328.9 to 616.8)	444.9 (319.5 to 587.7)	-4.2 (-16.50 to 10.08)	112.4 (78.0 to 152.1)	147.9 (103.3 to 198.1)	**32.3 (8.71 to 56.50)**	276.7 (191.0 to 375.9)	311.0 (215.6 to 416.9)	13.1 (-7.77 to 35.07)
Rheumatic heart disease	0.5 (0.3 to 0.7)	0.6 (0.4 to 0.8)	**22.4 (-3.06 to 62.52)**	0.8 (0.5 to 1.1)	0.8 (0.5 to 1.1)	-1.5 (-23.98 to 35.00)	0.2 (0.1 to 0.3)	0.3 (0.2 to 0.4)	**31.8 (-2.58 to 99.00)**	0.8 (0.4 to 1.1)	0.8 (0.5 to 1.1)	-0.7 (-26.60 to 50.30)	0.3 (0.2 to 0.4)	0.3 (0.2 to 0.5)	16.6 (-12.70 to 48.86)	0.8 (0.5 to 1.2)	0.8 (0.5 to 1.1)	-1.1 (-28.56 to 26.29)
Ischemic heart disease	75.6 (51.9 to 101.4)	86.9 (61.3 to 117.2)	**15.3 (4.46 to 26.08)**	111.1 (76.0 to 149.7)	97.8 (68.5 to 132.5)	**-11.8 (-20.19 to -3.27)**	47.0 (31.8 to 64.0)	50.4 (34.7 to 68.8)	7.7 (-5.76 to 23.38)	165.4 (112.0 to 224.8)	127.4 (87.2 to 174.1)	**-22.7 (-32.38 to -11.67)**	28.6 (19.5 to 38.7)	36.5 (25.6 to 49.3)	**28.1 (12.56 to 45.05)**	68.4 (46.3 to 93.7)	72.2 (50.3 to 98.1)	5.9 (-7.02 to 19.71)
Cerebrovascular disease	59.7 (41.9 to 77.9)	85.2 (60.4 to 110.2)	**42.8 (34.43 to 50.41)**	88.5 (62.2 to 115.5)	101.0 (71.6 to 130.7)	**14.2 (7.89 to 20.12)**	29.3 (20.5 to 38.9)	46.0 (32.1 to 59.7)	**57.6 (43.47 to 72.20)**	103.6 (72.6 to 137.6)	119.9 (83.4 to 155.8)	**16.0 (5.89 to 27.08)**	30.4 (21.5 to 39.6)	39.1 (27.8 to 50.4)	**28.7 (17.93 to 38.31)**	77.0 (54.1 to 100.4)	84.4 (59.7 to 109.1)	**9.7 (0.97 to 17.69)**
Ischemic stroke	49.1 (34.1 to 65.6)	70.1 (49.0 to 92.2)	**42.9 (34.26 to 51.70)**	72.2 (50.2 to 96.4)	82.3 (57.5 to 108.2)	**14.0 (7.31 to 20.99)**	24.0 (16.4 to 32.5)	37.9 (26.3 to 49.7)	**57.9 (40.93 to 73.14)**	84.8 (57.9 to 114.7)	98.0 (68.1 to 129.0)	**15.9 (3.73 to 27.46)**	25.1 (17.6 to 33.4)	32.2 (22.8 to 42.6)	**28.7 (16.87 to 40.10)**	62.5 (43.7 to 83.2)	68.3 (48.1 to 90.5)	**9.4 (-0.46 to 18.16)**
Hemorrhagic stroke	10.6 (7.4 to 14.3)	15.1 (10.4 to 20.1)	**42.9 (24.78 to 65.07)**	16.3 (11.4 to 22.0)	18.8 (12.9 to 25.0)	**15.2 (0.75 to 33.32)**	5.2 (3.6 to 7.0)	8.2 (5.5 to 11.1)	**57.0 (28.08 to 89.57)**	18.8 (12.9 to 25.1)	21.9 (14.8 to 29.7)	16.8 (-3.47 to 39.91)	5.4 (3.6 to 7.6)	6.9 (4.6 to 9.5)	**30.3 (2.53 to 63.13)**	14.5 (9.8 to 20.2)	16.1 (10.8 to 21.9)	11.9 (-11.42 to 39.54)
Hypertensive heart disease	10.9 (7.4 to 15.0)	14.4 (9.7 to 20.2)	**33.0 (10.30 to 56.45)**	15.4 (10.5 to 21.2)	16.1 (10.8 to 22.6)	5.1 (-12.71 to 24.15)	4.7 (3.1 to 6.6)	7.0 (4.6 to 10.1)	**51.5 (15.21 to 100.02)**	16.8 (11.2 to 23.8)	18.0 (11.8 to 25.9)	7.6 (-17.85 to 42.11)	6.2 (4.1 to 8.6)	7.4 (5.0 to 10.3)	20.0 (-4.88 to 45.35)	14.0 (9.4 to 19.4)	14.3 (9.6 to 20.0)	2.9 (-18.80 to 24.17)
Cardiomyopathy and myocarditis	10.5 (7.2 to 14.3)	10.3 (7.2 to 13.9)	-1.8 (-13.88 to 10.27)	15.9 (10.9 to 21.6)	12.6 (8.8 to 17.1)	**-20.3 (-29.81 to -11.33)**	6.5 (4.4 to 8.9)	6.5 (4.5 to 8.8)	-0.3 (-16.71 to 15.68)	23.5 (16.0 to 32.1)	17.4 (12.2 to 23.6)	**-25.8 (-37.60 to -14.34)**	4.0 (2.7 to 5.4)	3.8 (2.6 to 5.1)	-3.7 (-17.65 to 13.71)	9.7 (6.7 to 13.3)	8.4 (5.7 to 11.4)	**-13.6 (-25.28 to 0.94)**
Atrial fibrillation and flutter	11.8 (8.2 to 16.3)	12.0 (8.2 to 16.5)	1.8 (-10.94 to 17.31)	16.5 (11.5 to 22.9)	13.3 (9.1 to 18.3)	**-19.2 (-28.72 to -7.25)**	6.3 (4.3 to 9.0)	5.5 (3.7 to 7.7)	-12.4 (-30.44 to 5.92)	22.1 (15.1 to 31.5)	13.8 (9.2 to 19.3)	**-37.1 (-49.52 to -24.64)**	5.5 (3.7 to 7.6)	6.5 (4.3 to 9.1)	18.7 (-5.43 to 45.53)	12.3 (8.3 to 17.2)	12.8 (8.4 to 18.0)	4.7 (-15.38 to 27.86)
Peripheral vascular disease	2.4 (1.1 to 4.3)	2.8 (1.3 to 4.9)	19.0 (-11.06 to 71.61)	3.2 (1.5 to 5.7)	2.9 (1.4 to 5.0)	-8.2 (-31.42 to 33.16)	1.1 (0.5 to 2.0)	1.5 (0.7 to 2.6)	30.3 (-14.10 to 98.43)	4.1 (1.9 to 7.2)	3.5 (1.6 to 6.3)	-13.1 (-41.61 to 30.46)	1.3 (0.5 to 2.5)	1.3 (0.6 to 2.4)	13.6 (-31.54 to 91.96)	2.6 (0.9 to 5.1)	2.3 (1.1 to 4.2)	-2.3 (-41.92 to 64.45)
Endocarditis	0.3 (0.2 to 0.4)	0.4 (0.2 to 0.6)	**49.9 (18.38 to 138.64)**	0.4 (0.2 to 0.6)	0.5 (0.3 to 0.7)	**22.8 (-1.38 to 89.55)**	0.1 (0.1 to 0.2)	0.2 (0.1 to 0.3)	**102.7 (44.97 to 211.94)**	0.4 (0.2 to 0.6)	0.6 (0.4 to 0.9)	**53.3 (11.23 to 129.89)**	0.1 (0.1 to 0.2)	0.2 (0.1 to 0.3)	11.5 (-20.40 to 85.09)	0.4 (0.2 to 0.6)	0.4 (0.2 to 0.6)	-3.7 (-30.44 to 60.76)
Other cardiovascular and circulatory diseases	71.6 (38.4 to 113.2)	106.1 (60.3 to 155.8)	55.6 (-13.02 to 156.01)	108.8 (57.7 to 170.7)	129.0 (72.8 to 187.3)	24.6 (-29.63 to 104.46)	35.5 (19.9 to 54.9)	53.3 (31.2 to 78.4)	**56.1 (-3.40 to 149.39)**	129.3 (72.0 to 200.4)	143.6 (83.8 to 210.3)	15.5 (-29.44 to 83.41)	36.1 (18.2 to 60.6)	52.8 (28.8 to 81.3)	56.1 (-21.30 to 168.90)	91.5 (45.6 to 151.8)	115.4 (61.7 to 173.8)	34.6 (-31.90 to 131.21)
**Chronic respiratory diseases**	280.7 (197.8 to 385.4)	318.8 (220.8 to 433.8)	**13.7 (3.96 to 23.34)**	485.2 (338.5 to 663.4)	451.4 (308.9 to 611.1)	-6.9 (-14.73 to 1.27)	132.9 (90.5 to 185.7)	161.4 (110.3 to 223.7)	**21.7 (11.02 to 34.23)**	501.3 (342.5 to 702.7)	481.7 (331.8 to 670.7)	-3.7 (-12.41 to 5.75)	147.8 (101.0 to 205.6)	157.4 (105.9 to 220.1)	6.6 (-7.20 to 18.43)	472.0 (324.0 to 655.0)	422.6 (285.6 to 593.3)	**-10.3 (-22.98 to 0.67)**
Chronic obstructive pulmonary disease	167.0 (110.7 to 235.0)	203.2 (134.9 to 287.5)	**21.9 (8.23 to 35.91)**	279.7 (184.9 to 394.4)	276.7 (183.7 to 390.2)	-0.9 (-12.25 to 10.28)	82.7 (50.8 to 121.4)	106.9 (66.6 to 155.2)	**29.7 (13.90 to 48.33)**	309.1 (189.8 to 456.4)	309.0 (194.0 to 448.5)	0.3 (-11.78 to 14.24)	84.4 (50.7 to 125.0)	96.4 (59.1 to 141.9)	**14.6 (-2.76 to 31.65)**	253.5 (151.8 to 374.0)	246.2 (152.4 to 367.2)	-2.6 (-17.54 to 12.22)
Pneumoconiosis	0.2 (0.1 to 0.3)	0.2 (0.1 to 0.2)	**-15.5 (-22.39 to -9.80)**	0.3 (0.2 to 0.4)	0.2 (0.1 to 0.3)	**-32.8 (-38.25 to -28.50)**	0.1 (0.1 to 0.2)	0.1 (0.1 to 0.1)	**-14.9 (-23.55 to -7.46)**	0.4 (0.3 to 0.6)	0.3 (0.2 to 0.4)	**-37.0 (-43.55 to -32.17)**	0.1 (0.0 to 0.1)	0.1 (0.0 to 0.1)	**-16.4 (-30.28 to -7.19)**	0.2 (0.1 to 0.3)	0.1 (0.1 to 0.2)	**-29.1 (-40.23 to -22.47)**
Silicosis	0.1 (0.0 to 0.1)	<0.05 (0.0 to 0.1)	**-16.2 (-20.15 to -12.60)**	0.1 (0.1 to 0.1)	0.1 (0.0 to 0.1)	**-32.9 (-35.94 to -30.09)**	<0.05 (0.0 to 0.1)	<0.05 (0.0 to 0.1)	**-22.3 (-26.91 to -18.26)**	0.2 (0.1 to 0.2)	0.1 (0.1 to 0.1)	**-42.6 (-45.94 to -39.71)**	<0.05 (0.0 to <0.05)	<0.05 (0.0 to <0.05)	**15.6 (10.59 to 20.52)**	<0.05 (0.0 to <0.05)	<0.05 (0.0 to <0.05)	-1.6 (-5.83 to 2.74)
Other pneumoconiosis	0.1 (0.1 to 0.2)	0.1 (0.1 to 0.2)	**-15.3 (-25.32 to -6.68)**	0.2 (0.1 to 0.3)	0.1 (0.1 to 0.2)	**-32.8 (-40.90 to -26.48)**	0.1 (0.0 to 0.1)	0.1 (0.0 to 0.1)	-9.3 (-25.91 to 2.43)	0.2 (0.1 to 0.4)	0.2 (0.1 to 0.3)	**-33.1 (-45.06 to -25.89)**	0.1 (0.0 to 0.1)	0.1 (0.0 to 0.1)	**-20.9 (-37.06 to -10.86)**	0.2 (0.1 to 0.3)	0.1 (0.1 to 0.2)	**-33.1 (-46.14 to -25.66)**
Asthma	101.4 (66.6 to 144.0)	112.9 (73.0 to 158.8)	**11.5 (2.30 to 22.49)**	185.2 (121.7 to 263.5)	171.2 (110.2 to 242.6)	-7.4 (-15.26 to 2.15)	45.7 (29.8 to 65.5)	53.1 (34.3 to 75.5)	**16.7 (0.37 to 34.14)**	175.4 (113.9 to 252.2)	168.6 (109.0 to 239.8)	-3.5 (-17.57 to 11.42)	55.7 (36.2 to 78.0)	59.8 (38.6 to 85.0)	7.6 (-9.06 to 23.70)	195.3 (127.4 to 276.0)	173.3 (111.4 to 246.9)	-11.0 (-26.53 to 3.16)
Interstitial lung disease and pulmonary sarcoidosis	0.8 (0.5 to 1.2)	1.0 (0.6 to 1.4)	**25.7 (7.43 to 45.20)**	1.3 (0.8 to 1.9)	1.3 (0.8 to 1.9)	2.4 (-12.18 to 17.35)	0.4 (0.3 to 0.6)	0.6 (0.3 to 0.8)	**34.8 (6.06 to 66.04)**	1.5 (1.0 to 2.3)	1.6 (1.0 to 2.3)	3.1 (-18.21 to 25.68)	0.4 (0.2 to 0.6)	0.4 (0.3 to 0.7)	16.7 (-3.82 to 38.75)	1.2 (0.7 to 1.7)	1.1 (0.7 to 1.7)	-0.3 (-17.46 to 18.48)

Other chronic respiratory diseases	11.2 (6.8 to 17.7)	1.5 (0.9 to 2.2)	**-86.8 (-88.88 to -84.48)**	18.7 (11.5 to 29.3)	2.0 (1.2 to 3.0)	**-89.2 (-90.86 to -87.32)**	4.0 (2.3 to 6.0)	0.8 (0.5 to 1.1)	**-80.6 (-84.43 to -77.38)**	14.9 (8.8 to 22.4)	2.2 (1.4 to 3.3)	**-85.0 (-87.87 to -82.42)**	7.3 (3.9 to 12.6)	0.7 (0.4 to 1.2)	**-90.2 (-92.12 to -88.17)**	21.8 (11.6 to 37.9)	1.8 (1.0 to 3.0)	**-91.7 (-93.32 to -89.91)**
**Cirrhosis**	2.9 (2.0 to 4.0)	4.4 (3.1 to 6.1)	**53.1 (45.69 to 61.08)**	5.2 (3.7 to 7.3)	6.6 (4.6 to 9.1)	**26.2 (20.17 to 32.34)**	1.7 (1.2 to 2.3)	2.8 (1.9 to 3.8)	**66.0 (55.33 to 78.41)**	6.4 (4.5 to 8.8)	8.5 (5.9 to 11.7)	**33.1 (24.62 to 42.94)**	1.2 (0.9 to 1.7)	1.7 (1.2 to 2.3)	**35.4 (25.88 to 45.56)**	4.2 (2.9 to 5.9)	4.8 (3.4 to 6.6)	**14.9 (6.91 to 23.02)**
Cirrhosis due to hepatitis B	0.3 (0.2 to 0.4)	0.5 (0.3 to 0.7)	**60.7 (23.81 to 104.07)**	0.5 (0.3 to 0.8)	0.7 (0.4 to 1.0)	**31.8 (0.93 to 67.39)**	0.2 (0.1 to 0.2)	0.3 (0.2 to 0.4)	**63.5 (22.46 to 115.71)**	0.6 (0.4 to 0.9)	0.8 (0.5 to 1.1)	**31.3 (-1.08 to 72.84)**	0.1 (0.1 to 0.2)	0.2 (0.1 to 0.3)	**59.7 (4.01 to 135.07)**	0.5 (0.3 to 0.7)	0.6 (0.4 to 0.9)	32.9 (-14.64 to 94.51)
Cirrhosis due to hepatitis C	1.0 (0.7 to 1.5)	2.1 (1.4 to 2.9)	**100.1 (69.48 to 133.13)**	1.9 (1.3 to 2.7)	3.1 (2.1 to 4.3)	**63.5 (38.36 to 90.05)**	0.5 (0.3 to 0.8)	1.2 (0.8 to 1.7)	**132.1 (77.55 to 215.69)**	2.0 (1.2 to 3.0)	3.6 (2.4 to 5.1)	**85.3 (42.18 to 149.07)**	0.5 (0.3 to 0.8)	0.9 (0.6 to 1.3)	**72.0 (33.64 to 114.11)**	1.8 (1.2 to 2.6)	2.6 (1.7 to 3.6)	**43.0 (11.15 to 76.83)**
Cirrhosis due to alcohol use	1.2 (0.9 to 1.7)	1.2 (0.8 to 1.8)	0.9 (-14.96 to 20.11)	2.2 (1.5 to 3.0)	1.8 (1.2 to 2.5)	**-18.4 (-31.74 to -2.69)**	0.8 (0.6 to 1.2)	1.0 (0.7 to 1.4)	16.8 (-6.17 to 49.28)	3.1 (2.1 to 4.4)	2.9 (1.9 to 4.1)	-8.4 (-26.27 to 16.81)	0.4 (0.3 to 0.6)	0.3 (0.2 to 0.4)	**-30.6 (-50.92 to -8.18)**	1.3 (0.8 to 1.9)	0.7 (0.5 to 1.1)	**-41.9 (-59.35 to -22.13)**
Cirrhosis due to other causes	0.3 (0.2 to 0.5)	0.6 (0.4 to 0.9)	**99.6 (60.34 to 140.93)**	0.6 (0.4 to 0.9)	1.1 (0.7 to 1.5)	**65.8 (35.86 to 97.46)**	0.2 (0.1 to 0.2)	0.4 (0.2 to 0.5)	**127.5 (68.28 to 208.75)**	0.7 (0.4 to 1.0)	1.2 (0.8 to 1.8)	**87.2 (40.28 to 147.26)**	0.2 (0.1 to 0.2)	0.3 (0.2 to 0.4)	**73.6 (21.78 to 131.17)**	0.6 (0.4 to 0.9)	0.9 (0.6 to 1.3)	**45.0 (5.33 to 88.91)**
**Digestive diseases**	93.8 (67.2 to 124.4)	113.5 (81.1 to 151.8)	**21.1 (11.66 to 30.58)**	157.9 (113.3 to 210.4)	162.9 (116.3 to 219.0)	3.2 (-5.09 to 11.39)	37.5 (26.9 to 49.5)	50.9 (36.3 to 67.3)	**35.7 (22.75 to 50.81)**	142.8 (102.5 to 188.6)	154.6 (109.7 to 205.2)	8.3 (-2.80 to 20.56)	56.2 (40.3 to 75.1)	62.7 (44.6 to 83.7)	**11.5 (3.06 to 21.78)**	171.1 (121.3 to 229.8)	170.2 (121.1 to 228.5)	-0.4 (-8.61 to 8.91)
Peptic ulcer disease	17.7 (12.2 to 24.6)	8.1 (5.6 to 11.3)	**-54.1 (-56.32 to -51.74)**	24.9 (17.1 to 34.7)	8.7 (6.0 to 12.1)	**-65.0 (-66.59 to -63.01)**	4.5 (3.1 to 6.2)	1.7 (1.1 to 2.5)	**-61.6 (-67.39 to -54.42)**	16.6 (11.4 to 23.0)	4.3 (2.9 to 6.2)	**-73.9 (-77.77 to -69.08)**	13.2 (9.1 to 18.5)	6.4 (4.4 to 8.8)	**-51.6 (-53.45 to -49.64)**	31.4 (21.5 to 44.0)	12.2 (8.4 to 17.0)	**-61.1 (-62.66 to -59.31)**
Gastritis and duodenitis	4.3 (2.9 to 6.1)	6.5 (4.5 to 9.1)	**51.6 (42.09 to 59.71)**	7.6 (5.3 to 10.7)	9.4 (6.5 to 13.1)	**23.8 (15.32 to 32.00)**	0.7 (0.5 to 1.1)	1.3 (0.8 to 1.9)	**81.4 (45.99 to 114.75)**	2.8 (1.9 to 4.2)	3.8 (2.5 to 5.5)	**35.0 (9.51 to 61.72)**	3.6 (2.5 to 5.0)	5.2 (3.6 to 7.3)	**45.9 (36.74 to 54.47)**	12.0 (8.4 to 16.7)	14.8 (10.3 to 20.5)	**23.7 (13.80 to 32.40)**
Appendicitis	0.4 (0.3 to 0.6)	0.5 (0.3 to 0.6)	6.4 (-5.52 to 17.90)	1.0 (0.7 to 1.3)	1.0 (0.7 to 1.3)	-1.2 (-13.38 to 11.22)	0.2 (0.2 to 0.3)	0.3 (0.2 to 0.4)	11.8 (-3.72 to 28.79)	1.0 (0.7 to 1.4)	1.1 (0.7 to 1.5)	3.6 (-12.83 to 21.23)	0.2 (0.1 to 0.3)	0.2 (0.1 to 0.3)	0.7 (-13.50 to 16.28)	0.9 (0.6 to 1.3)	0.9 (0.6 to 1.2)	-6.2 (-21.46 to 10.54)
Paralytic ileus and intestinal obstruction	0.2 (0.1 to 0.2)	0.3 (0.2 to 0.4)	**58.7 (43.34 to 74.13)**	0.3 (0.2 to 0.4)	0.4 (0.3 to 0.5)	**31.5 (20.92 to 41.28)**	0.1 (0.0 to 0.1)	0.1 (0.1 to 0.2)	**74.5 (56.30 to 95.66)**	0.3 (0.2 to 0.4)	0.4 (0.3 to 0.5)	**35.8 (21.24 to 51.72)**	0.1 (0.1 to 0.1)	0.1 (0.1 to 0.2)	**46.9 (26.24 to 69.47)**	0.3 (0.2 to 0.4)	0.4 (0.3 to 0.5)	**27.5 (15.69 to 39.62)**
Inguinal, femoral, and abdominal hernia	4.2 (2.1 to 7.8)	4.9 (2.5 to 9.0)	**16.0 (9.01 to 22.98)**	6.2 (3.1 to 11.6)	5.8 (2.9 to 10.7)	**-6.9 (-12.13 to -1.68)**	2.6 (1.3 to 4.8)	3.2 (1.6 to 5.9)	**22.7 (13.58 to 32.52)**	9.6 (4.8 to 17.6)	8.5 (4.3 to 15.7)	**-11.5 (-17.84 to -4.87)**	1.6 (0.8 to 3.0)	1.7 (0.8 to 3.1)	4.9 (-4.83 to 15.35)	3.7 (1.8 to 7.0)	3.4 (1.7 to 6.4)	**-8.3 (-15.57 to -0.48)**
Inflammatory bowel disease	40.8 (28.2 to 55.3)	58.3 (39.9 to 79.2)	**42.9 (29.37 to 55.90)**	74.1 (51.1 to 100.5)	88.4 (60.4 to 120.8)	**19.5 (7.72 to 30.63)**	20.1 (13.6 to 27.4)	30.0 (20.2 to 41.1)	**49.9 (32.15 to 70.72)**	77.1 (52.4 to 105.6)	93.7 (63.1 to 129.3)	**22.0 (7.34 to 39.31)**	20.7 (14.2 to 28.2)	28.3 (19.3 to 38.6)	**36.6 (20.88 to 56.23)**	71.6 (49.0 to 97.7)	83.4 (56.9 to 114.1)	**16.7 (3.18 to 34.10)**
Vascular intestinal disorders	0.1 (0.1 to 0.2)	0.2 (0.1 to 0.3)	**29.4 (8.17 to 55.29)**	0.2 (0.1 to 0.3)	0.2 (0.1 to 0.3)	3.3 (-11.34 to 19.72)	0.1 (0.0 to 0.1)	0.1 (0.1 to 0.1)	**39.8 (10.24 to 72.40)**	0.2 (0.1 to 0.3)	0.2 (0.2 to 0.3)	1.9 (-18.26 to 23.88)	0.1 (0.1 to 0.1)	0.1 (0.1 to 0.1)	22.4 (-6.77 to 58.26)	0.2 (0.1 to 0.3)	0.2 (0.1 to 0.3)	4.1 (-15.51 to 27.48)
Gallbladder and biliary diseases	5.7 (3.9 to 7.8)	9.0 (6.2 to 12.2)	**56.9 (35.39 to 72.85)**	9.3 (6.3 to 12.7)	11.7 (7.9 to 15.9)	**26.2 (9.12 to 39.43)**	1.9 (1.3 to 2.6)	3.6 (2.5 to 4.9)	**94.5 (67.39 to 119.10)**	6.8 (4.7 to 9.4)	10.1 (6.9 to 13.8)	**48.3 (28.45 to 66.70)**	3.9 (2.6 to 5.3)	5.3 (3.6 to 7.3)	**39.0 (17.39 to 58.00)**	11.4 (7.7 to 15.8)	13.1 (8.8 to 18.0)	14.8 (-3.76 to 30.84)
Pancreatitis	3.5 (2.4 to 4.6)	4.9 (3.4 to 6.5)	**42.1 (35.32 to 49.19)**	6.2 (4.3 to 8.3)	7.4 (5.1 to 9.9)	**19.0 (13.36 to 25.21)**	2.0 (1.3 to 2.6)	2.8 (2.0 to 3.8)	**45.7 (35.26 to 55.63)**	7.5 (5.2 to 10.0)	8.9 (6.2 to 12.0)	**18.5 (9.70 to 27.12)**	1.5 (1.0 to 2.0)	2.1 (1.4 to 2.7)	**37.5 (27.19 to 48.56)**	4.9 (3.4 to 6.6)	5.9 (4.1 to 7.8)	**19.4 (9.72 to 29.62)**
Other digestive diseases	16.8 (11.6 to 23.1)	21.0 (14.0 to 28.5)	**25.1 (3.48 to 52.37)**	28.2 (19.5 to 38.8)	29.9 (19.8 to 41.2)	6.6 (-11.96 to 30.12)	5.4 (3.6 to 7.7)	7.7 (4.3 to 11.0)	**43.1 (-4.44 to 89.19)**	20.7 (13.5 to 29.3)	23.5 (13.2 to 33.5)	14.1 (-23.59 to 50.98)	11.4 (7.6 to 15.7)	13.2 (8.9 to 18.3)	**16.9 (-0.44 to 43.15)**	34.5 (23.2 to 48.4)	35.9 (23.9 to 49.8)	4.4 (-11.72 to 27.72)
**Neurological disorders**	539.6 (375.2 to 726.5)	672.2 (476.2 to 895.3)	**24.7 (16.66 to 34.02)**	982.6 (674.1 to 1,340.4)	1,031.2 (720.0 to 1,395.7)	5.0 (-2.44 to 13.08)	181.3 (127.0 to 241.1)	235.4 (168.0 to 309.8)	**30.1 (18.84 to 42.28)**	732.4 (512.0 to 977.5)	760.0 (533.7 to 1,008.6)	3.9 (-5.55 to 14.44)	358.3 (245.4 to 478.7)	436.8 (306.9 to 582.7)	**22.1 (13.19 to 32.47)**	1,217.4 (814.2 to 1,672.2)	1,292.0 (895.5 to 1,754.2)	6.3 (-2.07 to 16.05)
Alzheimer disease and other dementias	117.7 (86.3 to 150.8)	160.3 (118.6 to 206.3)	**36.3 (20.24 to 50.79)**	152.9 (112.3 to 195.7)	153.1 (113.3 to 196.3)	0.2 (-11.40 to 10.40)	32.5 (24.1 to 41.7)	53.0 (38.8 to 68.1)	**63.5 (44.54 to 85.07)**	122.9 (91.2 to 157.2)	124.3 (90.9 to 159.8)	1.3 (-10.10 to 14.71)	85.3 (62.4 to 109.9)	107.3 (77.7 to 139.4)	**26.1 (6.42 to 45.76)**	168.1 (122.5 to 216.8)	173.0 (125.9 to 224.1)	3.1 (-12.40 to 18.64)
Parkinson disease	7.5 (4.6 to 10.9)	9.5 (5.9 to 13.8)	**27.2 (20.94 to 34.19)**	10.6 (6.6 to 15.4)	10.8 (6.7 to 15.6)	1.4 (-3.78 to 6.61)	3.4 (2.1 to 4.9)	4.7 (3.0 to 6.8)	**39.6 (30.09 to 47.99)**	11.9 (7.4 to 17.3)	12.0 (7.4 to 17.1)	0.4 (-6.51 to 6.81)	4.1 (2.5 to 6.0)	4.8 (2.9 to 6.9)	**17.1 (9.32 to 28.22)**	9.7 (6.0 to 14.0)	9.8 (6.0 to 14.2)	0.9 (-5.62 to 8.97)
Epilepsy	62.8 (39.6 to 89.4)	64.5 (39.1 to 92.4)	4.7 (-31.09 to 52.54)	129.6 (81.2 to 184.6)	117.9 (71.3 to 169.2)	-7.3 (-38.73 to 34.98)	32.9 (20.5 to 47.4)	34.2 (20.7 to 48.4)	5.9 (-30.29 to 56.73)	139.5 (86.8 to 200.8)	126.5 (76.5 to 180.3)	-7.5 (-39.05 to 36.84)	29.9 (18.8 to 42.6)	30.3 (18.6 to 44.0)	3.4 (-31.72 to 50.10)	120.1 (75.6 to 171.0)	109.3 (66.8 to 158.6)	-7.2 (-38.59 to 34.83)
Multiple sclerosis	14.1 (10.0 to 18.2)	25.8 (18.6 to 33.4)	**83.4 (54.96 to 111.26)**	27.1 (19.2 to 34.8)	41.3 (29.8 to 53.5)	**52.8 (29.45 to 76.02)**	4.7 (3.2 to 6.1)	8.7 (6.2 to 11.2)	**88.1 (62.00 to 126.29)**	18.2 (12.4 to 23.6)	28.2 (20.0 to 36.5)	**55.5 (34.04 to 86.14)**	9.5 (6.6 to 12.5)	17.1 (12.2 to 22.5)	**81.7 (45.85 to 123.54)**	35.8 (24.7 to 46.9)	54.0 (38.3 to 70.9)	**52.1 (22.10 to 87.18)**
Migraine	241.6 (144.9 to 357.6)	265.2 (161.2 to 387.2)	**10.0 (-0.99 to 21.29)**	491.7 (295.6 to 727.7)	481.3 (291.2 to 701.2)	-1.9 (-11.81 to 8.15)	76.5 (46.0 to 115.5)	84.3 (49.7 to 124.3)	10.5 (-3.71 to 26.85)	317.0 (189.5 to 478.0)	309.9 (183.1 to 456.5)	-2.0 (-15.11 to 12.98)	165.1 (97.5 to 242.4)	180.9 (109.2 to 263.3)	9.9 (-3.81 to 28.54)	664.5 (393.1 to 975.1)	651.8 (395.0 to 955.6)	-1.6 (-14.02 to 14.95)
Tension-type headache	15.5 (7.4 to 27.5)	21.7 (10.6 to 37.9)	**40.9 (25.33 to 68.15)**	30.6 (14.6 to 54.5)	38.3 (18.9 to 66.6)	**26.0 (11.68 to 49.91)**	5.8 (2.8 to 10.4)	9.4 (4.7 to 16.4)	**62.8 (40.64 to 100.73)**	23.7 (11.4 to 42.1)	33.8 (16.6 to 58.8)	**44.1 (24.43 to 77.74)**	9.6 (4.5 to 17.6)	12.2 (5.9 to 21.4)	**28.3 (8.18 to 56.02)**	37.3 (17.6 to 67.4)	42.7 (20.8 to 74.8)	15.8 (-3.34 to 41.35)
Medication overuse headache	54.4 (32.3 to 81.9)	94.8 (54.9 to 144.8)	**74.6 (57.01 to 93.91)**	104.5 (62.0 to 157.5)	156.6 (90.3 to 240.0)	**50.2 (34.17 to 66.48)**	14.8 (8.6 to 22.2)	27.5 (16.2 to 42.8)	**87.0 (60.83 to 111.66)**	58.5 (34.0 to 88.1)	91.8 (53.9 to 143.3)	**57.2 (35.81 to 78.30)**	39.6 (23.3 to 60.2)	67.3 (38.8 to 103.2)	**70.3 (49.42 to 95.66)**	149.1 (87.4 to 226.8)	220.5 (126.6 to 338.5)	**48.4 (30.14 to 70.61)**
Other neurological disorders	25.9 (18.9 to 33.4)	30.4 (22.2 to 39.5)	**17.3 (3.48 to 34.73)**	35.6 (26.0 to 45.9)	32.0 (23.4 to 41.6)	-10.1 (-20.69 to 3.30)	10.8 (7.2 to 14.4)	13.5 (9.3 to 18.6)	**26.1 (2.41 to 54.02)**	40.6 (27.1 to 54.7)	33.4 (22.9 to 45.9)	**-17.3 (-32.40 to 0.34)**	15.2 (11.0 to 19.9)	16.8 (12.1 to 22.1)	11.5 (-9.69 to 33.55)	32.8 (23.8 to 43.2)	30.9 (22.2 to 40.8)	-5.4 (-22.85 to 12.69)
**Mental and substance use disorders**	1,152.5 (823.0 to 1,547.8)	1,264.6 (903.6 to 1,682.0)	**9.7 (7.88 to 11.93)**	2,337.6 (1,668.4 to 3,142.9)	2,302.9 (1,641.4 to 3,110.0)	**-1.5 (-2.94 to -0.03)**	561.8 (403.3 to 744.0)	623.5 (445.6 to 824.7)	**11.0 (8.67 to 13.47)**	2,332.6 (1,675.6 to 3,094.7)	2,311.3 (1,655.4 to 3,056.1)	-0.9 (-2.85 to 0.92)	590.8 (418.6 to 802.5)	641.1 (452.0 to 866.4)	**8.5 (6.25 to 11.34)**	2,335.8 (1,639.2 to 3,153.1)	2,290.9 (1,616.9 to 3,115.0)	**-1.9 (-3.58 to 0.07)**
Schizophrenia	88.3 (63.1 to 109.6)	101.3 (72.8 to 125.0)	**14.8 (11.38 to 18.46)**	171.4 (122.4 to 213.6)	169.6 (121.0 to 210.2)	-1.1 (-4.18 to 2.17)	48.7 (35.0 to 60.6)	56.4 (40.3 to 69.9)	**15.8 (11.05 to 21.41)**	192.8 (138.6 to 240.2)	192.3 (136.6 to 239.9)	-0.2 (-4.58 to 4.45)	39.6 (28.2 to 48.6)	44.9 (32.4 to 55.2)	**13.5 (8.60 to 18.26)**	149.9 (106.7 to 184.9)	146.9 (105.7 to 181.4)	-2.0 (-6.36 to 2.36)
Alcohol use disorders	56.8 (37.2 to 81.1)	71.5 (47.2 to 101.1)	**26.1 (21.70 to 30.80)**	116.3 (76.3 to 165.8)	135.3 (89.3 to 192.0)	**16.3 (12.55 to 20.59)**	41.9 (27.5 to 59.9)	53.7 (35.4 to 76.1)	**28.2 (22.84 to 33.98)**	172.5 (113.3 to 246.5)	203.7 (134.1 to 290.7)	**18.1 (13.24 to 23.15)**	14.8 (9.8 to 20.9)	17.8 (11.8 to 25.1)	**20.1 (15.13 to 25.17)**	60.3 (39.6 to 84.9)	66.4 (43.9 to 94.1)	**10.3 (5.65 to 14.98)**
Drug use disorders	127.1 (88.5 to 169.1)	129.0 (89.8 to 170.8)	1.6 (-3.43 to 6.92)	263.4 (183.5 to 350.0)	250.4 (173.0 to 331.6)	**-4.9 (-9.68 to 0.19)**	87.5 (60.5 to 117.8)	89.8 (62.6 to 119.5)	2.7 (-3.41 to 9.94)	361.0 (249.5 to 486.3)	343.9 (238.4 to 457.7)	-4.6 (-10.38 to 2.15)	39.6 (28.0 to 52.7)	39.3 (27.2 to 52.2)	-0.9 (-7.29 to 5.55)	166.2 (117.4 to 220.8)	156.9 (108.6 to 209.1)	**-5.5 (-11.54 to 0.59)**
Opioid use disorders	49.9 (31.4 to 75.2)	49.5 (31.5 to 73.2)	-0.6 (-8.85 to 10.59)	100.8 (63.2 to 152.4)	89.4 (56.4 to 134.4)	**-11.1 (-18.30 to -1.84)**	39.3 (24.8 to 58.9)	38.9 (25.0 to 56.8)	-0.7 (-9.91 to 11.90)	159.0 (100.4 to 239.3)	140.5 (89.3 to 208.7)	**-11.4 (-19.57 to -1.00)**	10.6 (6.3 to 16.3)	10.6 (6.4 to 16.2)	-0.2 (-10.95 to 10.52)	43.0 (25.3 to 66.2)	38.8 (23.0 to 60.7)	**-9.8 (-19.16 to -0.63)**
Cocaine use disorders	17.1 (11.3 to 24.0)	18.7 (12.3 to 26.4)	**9.6 (4.62 to 14.60)**	35.8 (23.7 to 50.4)	37.6 (24.7 to 52.9)	**4.9 (0.01 to 9.90)**	11.3 (7.4 to 16.0)	12.5 (8.3 to 17.7)	**10.8 (4.40 to 18.04)**	47.3 (31.1 to 66.9)	50.0 (33.1 to 70.6)	**5.8 (-0.51 to 12.79)**	5.8 (3.8 to 8.1)	6.2 (4.1 to 8.7)	**7.2 (-0.52 to 14.85)**	24.2 (16.1 to 34.1)	24.9 (16.5 to 35.1)	2.8 (-4.91 to 9.98)
Amphetamine use disorders	18.7 (11.8 to 27.0)	18.3 (11.5 to 26.2)	-2.1 (-6.72 to 2.39)	39.2 (24.7 to 56.7)	36.9 (23.2 to 53.1)	**-5.8 (-10.39 to -1.37)**	11.0 (6.9 to 15.8)	11.2 (7.0 to 16.1)	1.6 (-4.58 to 7.79)	45.9 (28.7 to 65.9)	44.2 (27.7 to 63.9)	-3.6 (-9.51 to 2.55)	7.7 (4.9 to 11.1)	7.1 (4.5 to 10.2)	**-7.2 (-13.38 to -1.01)**	32.6 (20.7 to 47.2)	29.6 (18.6 to 42.6)	**-9.1 (-15.42 to -2.77)**
Cannabis use disorders	5.3 (3.6 to 7.7)	5.3 (3.5 to 7.6)	-1.5 (-6.97 to 4.02)	11.8 (8.0 to 16.9)	11.4 (7.6 to 16.4)	-4.0 (-9.79 to 1.75)	3.3 (2.3 to 4.8)	3.3 (2.2 to 4.8)	-0.9 (-6.61 to 5.48)	14.6 (9.9 to 21.1)	14.1 (9.4 to 20.3)	-3.7 (-9.49 to 2.80)	2.0 (1.3 to 2.9)	1.9 (1.3 to 2.8)	-2.6 (-10.24 to 5.15)	9.0 (6.0 to 12.9)	8.6 (5.7 to 12.4)	-4.7 (-12.36 to 3.33)
Other drug use disorders	36.1 (24.0 to 49.2)	37.3 (24.9 to 51.0)	3.6 (-6.97 to 15.27)	75.8 (50.4 to 103.4)	75.2 (49.9 to 103.0)	-0.6 (-10.73 to 10.74)	22.5 (14.9 to 31.1)	23.9 (15.8 to 32.5)	6.4 (-8.17 to 23.13)	94.1 (62.2 to 130.2)	95.1 (62.7 to 129.4)	1.4 (-12.60 to 17.42)	13.6 (9.1 to 18.6)	13.4 (8.9 to 18.5)	-0.7 (-14.85 to 13.66)	57.4 (38.4 to 79.0)	55.1 (36.7 to 75.9)	-3.7 (-17.48 to 10.50)
Depressive disorders	367.8 (226.8 to 552.9)	402.0 (251.8 to 604.6)	**9.5 (5.11 to 14.71)**	718.5 (441.8 to 1,087.3)	687.7 (427.7 to 1,037.2)	**-4.2 (-7.37 to -0.33)**	151.2 (92.2 to 230.8)	167.5 (103.2 to 254.2)	**10.9 (4.72 to 18.34)**	610.6 (370.5 to 929.3)	583.6 (357.4 to 887.6)	**-4.3 (-9.11 to 0.55)**	216.6 (134.5 to 323.4)	234.6 (147.0 to 349.0)	**8.5 (3.79 to 14.71)**	821.9 (508.3 to 1,238.6)	790.0 (495.2 to 1,186.8)	-3.7 (-7.02 to 1.25)
Major depressive disorder	291.8 (170.4 to 465.6)	315.1 (185.9 to 499.4)	**8.2 (2.90 to 14.88)**	573.7 (335.1 to 923.9)	543.2 (317.0 to 864.2)	**-5.2 (-9.03 to -0.26)**	123.1 (71.8 to 198.1)	134.7 (78.7 to 218.2)	**9.6 (2.19 to 18.85)**	499.7 (289.1 to 805.9)	472.5 (273.4 to 760.4)	**-5.4 (-10.99 to 0.71)**	168.7 (99.2 to 268.1)	180.4 (107.5 to 282.9)	**7.2 (1.30 to 15.23)**	644.8 (376.9 to 1,029.5)	612.9 (359.8 to 969.6)	-4.7 (-8.69 to 1.65)
Dysthymia	76.0 (50.0 to 110.5)	86.9 (57.2 to 127.3)	**14.4 (12.43 to 16.31)**	144.8 (95.4 to 210.5)	144.5 (95.1 to 210.5)	-0.2 (-1.08 to 0.70)	28.0 (18.2 to 40.7)	32.7 (21.3 to 47.6)	**16.7 (14.31 to 19.32)**	110.9 (72.4 to 161.1)	111.1 (72.4 to 161.1)	0.2 (-1.16 to 1.46)	47.9 (31.6 to 70.9)	54.2 (35.8 to 79.9)	**13.0 (10.94 to 15.00)**	177.1 (115.7 to 260.7)	177.1 (115.9 to 259.5)	-0.0 (-1.06 to 1.13)
Bipolar disorder	72.3 (44.8 to 108.5)	80.3 (49.8 to 119.6)	**11.1 (7.34 to 14.64)**	142.7 (88.2 to 215.7)	142.0 (87.4 to 213.7)	-0.5 (-3.30 to 2.52)	29.6 (18.4 to 44.9)	33.1 (20.4 to 49.6)	**11.7 (7.40 to 16.22)**	119.6 (74.1 to 181.9)	118.7 (73.4 to 179.3)	-0.7 (-4.41 to 3.40)	42.6 (26.2 to 63.9)	47.2 (29.2 to 70.1)	**10.7 (6.33 to 15.05)**	165.2 (101.1 to 248.6)	165.1 (100.7 to 250.1)	-0.1 (-3.73 to 3.89)
Anxiety disorders	225.1 (111.5 to 355.0)	250.4 (128.0 to 395.6)	**11.4 (6.16 to 16.49)**	454.2 (209.7 to 734.3)	453.5 (208.8 to 731.7)	-0.2 (-0.96 to 0.64)	76.1 (38.7 to 123.4)	85.6 (44.6 to 138.7)	**12.8 (6.76 to 20.12)**	313.5 (151.3 to 514.8)	314.3 (150.8 to 517.1)	0.2 (-1.13 to 1.61)	149.0 (73.3 to 235.7)	164.8 (83.3 to 261.4)	**10.7 (5.44 to 15.33)**	591.7 (262.3 to 964.6)	592.0 (262.7 to 961.9)	0.0 (-0.91 to 0.97)
Eating disorders	35.1 (20.6 to 54.8)	37.0 (22.2 to 57.1)	**5.8 (1.78 to 10.70)**	76.3 (45.1 to 118.3)	80.3 (48.2 to 123.1)	**5.4 (1.70 to 9.38)**	6.5 (3.5 to 11.4)	6.6 (3.7 to 11.4)	3.4 (-1.79 to 9.91)	28.0 (15.4 to 48.9)	28.9 (16.2 to 48.9)	3.6 (-1.84 to 10.11)	28.6 (17.0 to 43.9)	30.4 (18.1 to 46.7)	**6.3 (2.04 to 11.22)**	125.4 (74.8 to 191.6)	132.8 (79.6 to 201.6)	**6.1 (2.11 to 10.33)**
Anorexia nervosa	8.3 (4.4 to 13.8)	10.2 (5.6 to 17.0)	**23.0 (13.80 to 33.53)**	18.2 (9.8 to 30.2)	22.0 (12.0 to 36.4)	**21.1 (12.99 to 30.78)**	1.2 (0.7 to 2.0)	1.4 (0.8 to 2.3)	**11.9 (0.40 to 25.27)**	5.5 (3.1 to 9.0)	6.1 (3.5 to 10.1)	**11.6 (0.61 to 24.36)**	7.0 (3.6 to 11.9)	8.8 (4.7 to 14.9)	**25.0 (14.63 to 38.72)**	31.1 (16.2 to 52.2)	38.2 (20.3 to 64.5)	**23.1 (13.41 to 35.73)**
Bulimia nervosa	26.8 (15.1 to 44.3)	26.9 (15.1 to 43.7)	0.4 (-2.62 to 4.46)	58.1 (33.2 to 95.3)	58.3 (33.2 to 94.4)	0.4 (-2.22 to 3.81)	5.2 (2.7 to 10.0)	5.3 (2.7 to 9.9)	1.4 (-3.88 to 8.51)	22.5 (11.7 to 42.9)	22.8 (12.0 to 42.4)	1.7 (-3.92 to 8.33)	21.6 (12.2 to 34.9)	21.6 (12.4 to 34.6)	0.2 (-2.88 to 4.14)	94.3 (54.4 to 151.4)	94.6 (54.7 to 150.8)	0.5 (-2.20 to 3.82)
Autistic spectrum disorders	52.5 (36.7 to 71.4)	58.5 (41.0 to 79.5)	**11.4 (10.10 to 12.83)**	113.4 (79.2 to 154.2)	114.0 (79.8 to 154.8)	0.5 (-0.68 to 1.76)	39.0 (27.1 to 52.8)	43.7 (30.5 to 59.2)	**12.0 (10.47 to 13.48)**	170.5 (118.7 to 231.5)	170.8 (119.5 to 232.5)	0.2 (-1.18 to 1.60)	13.5 (9.6 to 18.3)	14.9 (10.4 to 20.2)	**9.9 (7.38 to 12.45)**	56.9 (40.2 to 76.8)	56.9 (39.8 to 77.2)	-0.0 (-2.31 to 2.31)
Autism	33.2 (22.1 to 45.3)	37.0 (24.8 to 50.1)	**11.4 (9.41 to 13.32)**	71.8 (47.8 to 98.3)	72.2 (48.3 to 97.8)	0.5 (-1.17 to 2.23)	23.9 (15.9 to 32.4)	26.7 (17.9 to 36.4)	**11.9 (9.64 to 14.26)**	104.7 (69.5 to 142.0)	104.9 (70.2 to 142.6)	0.2 (-1.77 to 2.32)	9.3 (6.3 to 12.8)	10.3 (6.9 to 14.1)	**10.0 (6.53 to 13.38)**	39.2 (26.4 to 53.9)	39.2 (26.2 to 54.1)	0.0 (-3.07 to 3.17)
Asperger syndrome	19.3 (13.4 to 26.7)	21.5 (15.0 to 29.7)	**11.6 (10.01 to 13.19)**	41.6 (28.8 to 57.3)	41.8 (29.2 to 57.7)	0.5 (-0.85 to 1.96)	15.1 (10.5 to 20.9)	16.9 (11.8 to 23.4)	**12.0 (10.23 to 13.85)**	65.8 (45.6 to 91.1)	65.9 (46.0 to 91.1)	0.1 (-1.47 to 1.76)	4.2 (2.9 to 5.8)	4.6 (3.2 to 6.3)	**9.9 (6.50 to 13.06)**	17.6 (12.3 to 24.4)	17.6 (12.2 to 24.3)	-0.0 (-2.95 to 3.00)
Attention-deficit/hyperactivity disorder	2.9 (1.6 to 4.6)	2.9 (1.7 to 4.6)	1.8 (-0.54 to 3.94)	7.6 (4.3 to 12.0)	7.7 (4.4 to 12.1)	0.4 (-1.84 to 2.50)	2.2 (1.3 to 3.5)	2.3 (1.3 to 3.6)	1.9 (-0.65 to 4.53)	11.4 (6.5 to 18.1)	11.5 (6.5 to 18.1)	0.3 (-2.28 to 2.84)	0.7 (0.4 to 1.1)	0.7 (0.4 to 1.1)	1.4 (-3.36 to 6.45)	3.7 (2.1 to 5.7)	3.7 (2.1 to 5.7)	0.3 (-4.37 to 5.43)
Conduct disorder	41.1 (25.2 to 61.9)	41.9 (25.6 to 63.1)	**2.2 (0.63 to 3.74)**	110.7 (68.4 to 167.1)	111.0 (68.1 to 166.6)	0.2 (-1.19 to 1.71)	29.4 (18.4 to 44.6)	30.0 (18.5 to 45.6)	**2.1 (0.32 to 4.09)**	154.6 (96.1 to 234.1)	154.7 (95.9 to 233.1)	0.1 (-1.67 to 1.94)	11.7 (7.0 to 17.5)	11.9 (7.2 to 18.0)	2.2 (-0.49 to 4.98)	64.9 (39.4 to 97.4)	65.1 (39.2 to 98.0)	0.2 (-2.36 to 2.84)
Idiopathic intellectual disability	17.0 (10.7 to 24.5)	12.7 (7.4 to 19.3)	**-25.7 (-43.57 to -8.99)**	36.4 (22.8 to 52.4)	24.2 (14.3 to 36.9)	**-33.4 (-49.41 to -18.10)**	7.7 (4.7 to 11.3)	6.2 (3.6 to 9.5)	-19.2 (-41.13 to 8.01)	33.5 (20.5 to 49.3)	24.0 (14.0 to 36.7)	**-28.0 (-47.31 to -3.68)**	9.3 (5.9 to 13.5)	6.5 (3.6 to 9.9)	**-30.6 (-50.36 to -11.89)**	39.1 (25.0 to 56.6)	24.4 (13.5 to 37.3)	**-37.6 (-55.46 to -20.89)**
Other mental and substance use disorders	66.7 (45.3 to 89.6)	76.9 (52.3 to 103.1)	**15.4 (13.63 to 17.17)**	126.6 (86.0 to 170.4)	127.3 (86.5 to 170.3)	0.6 (-0.78 to 1.98)	42.0 (28.4 to 56.4)	48.7 (32.7 to 65.4)	**16.0 (13.71 to 18.61)**	164.5 (111.5 to 221.6)	164.9 (111.2 to 222.4)	0.2 (-1.33 to 1.89)	24.7 (17.0 to 33.5)	28.3 (19.4 to 38.4)	**14.4 (11.89 to 16.99)**	90.6 (61.9 to 122.9)	90.7 (61.9 to 123.3)	0.1 (-1.92 to 2.12)
**Diabetes, urogenital, blood, and endocrine diseases**	518.5 (374.1 to 678.7)	758.5 (545.7 to 993.4)	**46.2 (40.70 to 53.00)**	953.6 (685.8 to 1,260.0)	1,138.9 (818.3 to 1,499.7)	**19.4 (15.10 to 24.36)**	213.6 (151.9 to 280.4)	344.1 (241.1 to 452.1)	**61.1 (50.95 to 72.37)**	821.2 (579.0 to 1,080.9)	1,025.9 (717.3 to 1,339.4)	**24.9 (17.54 to 33.51)**	304.9 (220.7 to 401.2)	414.4 (298.6 to 543.8)	**35.9 (29.54 to 43.92)**	1,105.0 (788.8 to 1,470.6)	1,263.4 (893.7 to 1,674.6)	**14.3 (9.57 to 19.73)**
Diabetes mellitus	138.4 (93.5 to 194.7)	300.5 (203.6 to 408.6)	**117.6 (93.38 to 141.08)**	232.4 (156.9 to 328.1)	406.6 (275.8 to 555.7)	**75.3 (56.88 to 94.11)**	59.1 (38.7 to 84.0)	148.7 (99.1 to 209.1)	**152.8 (111.67 to 200.06)**	222.0 (145.5 to 315.1)	427.9 (285.1 to 603.8)	**93.6 (62.84 to 129.35)**	79.3 (54.4 to 111.4)	151.9 (103.9 to 207.7)	**92.0 (66.34 to 118.69)**	239.2 (164.3 to 336.0)	386.7 (264.2 to 529.7)	**62.0 (40.79 to 82.49)**
Acute glomerulonephritis	<0.05 (0.0 to <0.05)	<0.05 (0.0 to <0.05)	**-19.5 (-22.75 to -15.98)**	<0.05 (0.0 to <0.05)	<0.05 (0.0 to <0.05)	**-23.2 (-26.31 to -20.16)**	<0.05 (0.0 to <0.05)	<0.05 (0.0 to <0.05)	**-17.1 (-20.83 to -12.80)**	<0.05 (0.0 to <0.05)	<0.05 (0.0 to <0.05)	**-24.6 (-27.87 to -21.13)**	<0.05 (0.0 to <0.05)	<0.05 (0.0 to <0.05)	**-22.4 (-26.88 to -17.65)**	<0.05 (0.0 to <0.05)	<0.05 (0.0 to <0.05)	**-22.5 (-26.45 to -18.54)**
Chronic kidney disease	196.1 (130.0 to 264.7)	236.6 (155.8 to 319.8)	**20.6 (17.21 to 23.25)**	355.5 (235.4 to 482.6)	355.1 (236.0 to 483.0)	-0.1 (-1.77 to 1.61)	84.8 (55.1 to 117.1)	104.8 (67.2 to 145.3)	**23.5 (19.04 to 27.06)**	326.2 (213.3 to 452.8)	321.9 (208.7 to 447.4)	-1.4 (-3.87 to 0.67)	111.2 (74.6 to 149.9)	131.7 (88.2 to 176.8)	**18.5 (14.64 to 22.20)**	384.5 (257.2 to 523.0)	387.9 (259.8 to 525.2)	0.9 (-1.47 to 3.64)
Chronic kidney disease due to diabetes mellitus	32.7 (20.3 to 45.7)	50.2 (31.6 to 69.3)	**54.3 (32.35 to 77.86)**	58.6 (36.9 to 83.1)	71.2 (45.3 to 99.0)	**21.9 (5.14 to 40.65)**	13.1 (8.1 to 19.1)	21.4 (13.1 to 30.4)	**64.6 (32.32 to 120.35)**	49.3 (30.6 to 72.3)	62.5 (38.4 to 88.8)	**27.9 (3.73 to 69.43)**	19.6 (11.9 to 27.5)	28.8 (18.1 to 40.1)	**48.1 (26.17 to 70.67)**	67.2 (41.0 to 94.9)	79.5 (50.6 to 111.1)	**18.9 (0.93 to 37.76)**
Chronic kidney disease due to hypertension	54.4 (34.8 to 75.5)	23.7 (15.6 to 32.6)	**-56.4 (-60.76 to -50.57)**	96.4 (61.9 to 134.2)	35.2 (23.2 to 48.9)	**-63.3 (-67.07 to -58.26)**	26.6 (16.4 to 37.4)	11.4 (7.3 to 16.1)	**-57.2 (-63.39 to -50.76)**	100.7 (62.5 to 141.7)	35.1 (22.7 to 49.8)	**-65.0 (-70.19 to -59.42)**	27.8 (18.0 to 38.7)	12.3 (8.2 to 17.4)	**-55.5 (-61.10 to -48.25)**	93.5 (60.6 to 132.5)	35.4 (23.2 to 50.3)	**-62.0 (-67.00 to -55.73)**
Chronic kidney disease due to glomerulonephritis	19.7 (13.2 to 26.9)	41.1 (26.9 to 56.7)	**109.0 (86.42 to 130.76)**	38.7 (26.1 to 52.9)	66.4 (43.5 to 92.3)	**71.4 (52.82 to 91.36)**	9.6 (6.1 to 13.5)	20.3 (13.0 to 28.9)	**112.0 (83.05 to 145.16)**	38.4 (25.0 to 53.9)	65.6 (42.1 to 93.0)	**71.0 (47.28 to 97.62)**	10.1 (6.7 to 13.9)	20.8 (13.4 to 28.9)	**106.7 (79.85 to 143.48)**	39.4 (26.3 to 55.1)	67.4 (43.2 to 95.9)	**71.2 (46.40 to 104.48)**
Chronic kidney disease due to other causes	89.3 (60.4 to 119.5)	121.6 (80.7 to 167.1)	**36.0 (24.31 to 46.61)**	161.7 (111.0 to 216.5)	182.3 (123.1 to 249.6)	**12.6 (3.51 to 21.20)**	35.5 (23.3 to 49.5)	51.8 (33.0 to 73.3)	**45.9 (26.15 to 61.81)**	137.7 (91.0 to 189.8)	158.6 (102.2 to 222.8)	**15.2 (0.13 to 27.54)**	53.8 (36.7 to 71.5)	69.8 (46.5 to 94.8)	**29.6 (19.10 to 42.02)**	184.4 (125.0 to 245.9)	205.6 (137.6 to 279.5)	**11.4 (2.93 to 21.96)**
Urinary diseases and male infertility	47.7 (31.0 to 67.7)	65.0 (42.1 to 92.0)	**36.3 (31.17 to 41.62)**	71.8 (46.6 to 102.4)	79.6 (51.0 to 112.7)	**10.8 (6.41 to 15.41)**	45.7 (29.9 to 64.9)	61.8 (40.3 to 87.1)	**35.2 (30.26 to 40.08)**	159.7 (104.5 to 227.4)	161.1 (104.6 to 229.2)	0.9 (-3.03 to 4.71)	2.0 (1.0 to 3.5)	3.2 (1.6 to 6.0)	**62.3 (44.59 to 81.28)**	6.1 (3.0 to 11.0)	8.3 (4.0 to 15.7)	**35.6 (21.08 to 51.42)**
Interstitial nephritis and urinary tract infections	0.2 (0.1 to 0.3)	0.3 (0.2 to 0.4)	**58.9 (33.10 to 87.43)**	0.3 (0.2 to 0.5)	0.4 (0.3 to 0.6)	**32.3 (12.89 to 56.47)**	0.1 (0.0 to 0.1)	0.1 (0.1 to 0.1)	**48.3 (20.80 to 82.07)**	0.2 (0.1 to 0.3)	0.2 (0.1 to 0.4)	11.4 (-9.34 to 35.12)	0.1 (0.1 to 0.2)	0.2 (0.1 to 0.3)	**64.3 (31.80 to 106.12)**	0.4 (0.3 to 0.6)	0.6 (0.4 to 0.9)	**42.6 (16.15 to 76.62)**
Urolithiasis	4.2 (2.0 to 7.8)	8.8 (4.4 to 16.9)	**109.8 (85.48 to 132.06)**	7.0 (3.3 to 13.0)	11.7 (5.6 to 22.5)	**67.1 (48.16 to 83.40)**	2.5 (1.2 to 4.7)	5.9 (3.0 to 11.3)	**141.6 (111.28 to 175.49)**	9.1 (4.5 to 17.5)	16.6 (8.2 to 31.5)	**81.6 (58.97 to 103.69)**	1.7 (0.8 to 3.1)	2.9 (1.4 to 5.5)	**64.9 (45.24 to 85.40)**	5.2 (2.5 to 9.7)	7.2 (3.4 to 14.1)	**37.4 (20.79 to 54.87)**

Benign prostatic hyperplasia	41.0 (26.9 to 57.7)	53.6 (35.0 to 74.9)	**30.5 (26.60 to 34.74)**	60.2 (39.5 to 84.6)	63.4 (41.4 to 88.8)	**5.3 (2.08 to 8.62)**	41.0 (26.9 to 57.7)	53.6 (35.0 to 74.9)	**30.5 (26.60 to 34.74)**	142.0 (93.2 to 199.4)	136.4 (89.1 to 191.0)	**-3.9 (-6.75 to -0.87)**	--	--	--	--	--	--
Male infertility due to other causes	1.4 (0.6 to 2.9)	1.5 (0.6 to 2.9)	3.6 (-11.53 to 20.25)	2.9 (1.2 to 6.0)	2.9 (1.3 to 5.8)	0.4 (-14.38 to 16.36)	1.4 (0.6 to 2.9)	1.5 (0.6 to 2.9)	3.6 (-11.53 to 20.25)	5.8 (2.4 to 11.9)	5.8 (2.5 to 11.6)	0.4 (-14.39 to 16.38)	--	--	--	--	--	--
Other urinary diseases	0.9 (0.3 to 1.7)	0.9 (0.4 to 1.8)	20.2 (-28.63 to 129.99)	1.4 (0.5 to 2.9)	1.2 (0.5 to 2.4)	-4.9 (-42.96 to 79.98)	0.7 (0.3 to 1.5)	0.8 (0.3 to 1.5)	19.2 (-35.53 to 151.69)	2.7 (0.9 to 5.4)	2.1 (0.9 to 4.3)	-10.7 (-51.26 to 85.64)	0.1 (0.1 to 0.3)	0.2 (0.1 to 0.3)	**31.2 (-1.12 to 101.16)**	0.4 (0.2 to 0.8)	0.5 (0.2 to 0.9)	9.4 (-17.04 to 66.48)
Gynecological diseases	59.7 (38.5 to 92.1)	68.1 (43.4 to 105.8)	**14.0 (8.16 to 20.37)**	120.4 (77.8 to 185.4)	121.4 (77.7 to 186.1)	0.8 (-4.13 to 6.82)	--	--	--	--	--	--	59.7 (38.5 to 92.1)	68.1 (43.4 to 105.8)	**14.0 (8.16 to 20.37)**	240.7 (155.5 to 370.4)	242.1 (155.0 to 370.8)	0.6 (-4.37 to 6.56)
Uterine fibroids	15.7 (7.7 to 28.8)	19.1 (9.2 to 35.6)	**21.8 (12.52 to 33.33)**	31.6 (15.6 to 57.9)	32.2 (15.5 to 60.0)	2.0 (-5.75 to 11.52)	--	--	--	--	--	--	15.7 (7.7 to 28.8)	19.1 (9.2 to 35.6)	**21.8 (12.52 to 33.33)**	63.3 (31.2 to 116.0)	64.0 (30.9 to 119.2)	1.3 (-6.41 to 10.72)
Polycystic ovarian syndrome	7.7 (3.6 to 14.7)	8.6 (4.0 to 16.2)	**12.3 (4.22 to 20.56)**	15.7 (7.4 to 30.1)	16.1 (7.5 to 30.2)	2.5 (-5.09 to 10.24)	--	--	--	--	--	--	7.7 (3.6 to 14.7)	8.6 (4.0 to 16.2)	**12.3 (4.22 to 20.56)**	31.4 (14.9 to 60.4)	32.1 (15.1 to 60.4)	2.2 (-5.34 to 9.90)
Female infertility due to other causes	0.3 (0.1 to 0.7)	0.4 (0.1 to 0.9)	25.6 (-52.68 to 172.21)	0.7 (0.2 to 1.5)	0.8 (0.2 to 1.7)	20.1 (-54.19 to 161.82)	--	--	--	--	--	--	0.3 (0.1 to 0.7)	0.4 (0.1 to 0.9)	25.6 (-52.68 to 172.21)	1.4 (0.5 to 3.0)	1.5 (0.5 to 3.4)	20.1 (-54.27 to 161.77)
Endometriosis	12.5 (8.3 to 17.4)	13.6 (9.0 to 19.0)	9.4 (-5.08 to 25.55)	25.3 (16.9 to 35.4)	25.2 (16.6 to 35.0)	-0.5 (-13.84 to 14.25)	--	--	--	--	--	--	12.5 (8.3 to 17.4)	13.6 (9.0 to 19.0)	9.4 (-5.08 to 25.55)	50.7 (33.8 to 70.8)	50.2 (33.1 to 69.9)	-0.8 (-14.07 to 13.91)
Genital prolapse	9.6 (4.7 to 18.0)	11.0 (5.4 to 20.5)	**15.2 (9.94 to 20.69)**	18.2 (9.0 to 34.4)	17.8 (8.7 to 33.1)	-2.3 (-6.89 to 2.64)	--	--	--	--	--	--	9.6 (4.7 to 18.0)	11.0 (5.4 to 20.5)	**15.2 (9.94 to 20.69)**	35.9 (17.8 to 67.8)	35.3 (17.2 to 65.6)	-1.9 (-6.58 to 3.09)
Premenstrual syndrome	10.2 (6.3 to 15.6)	10.9 (6.8 to 16.7)	7.0 (-10.28 to 27.52)	21.1 (12.9 to 32.0)	21.1 (13.0 to 32.4)	0.6 (-15.67 to 19.45)	--	--	--	--	--	--	10.2 (6.3 to 15.6)	10.9 (6.8 to 16.7)	7.0 (-10.28 to 27.52)	42.3 (25.8 to 64.3)	42.4 (26.2 to 65.0)	0.5 (-15.78 to 19.40)
Other gynecological diseases	3.8 (2.6 to 5.2)	4.5 (2.9 to 6.6)	**18.6 (3.93 to 59.01)**	7.8 (5.4 to 10.7)	8.3 (5.4 to 12.1)	6.1 (-7.11 to 44.03)	--	--	--	--	--	--	3.8 (2.6 to 5.2)	4.5 (2.9 to 6.6)	**18.6 (3.93 to 59.01)**	15.7 (10.9 to 21.5)	16.6 (10.9 to 24.3)	5.9 (-7.32 to 43.71)
Hemoglobinopathies and hemolytic anemias	51.7 (34.5 to 74.0)	53.3 (35.6 to 76.4)	**3.1 (-0.36 to 7.34)**	122.1 (81.7 to 174.6)	114.3 (76.6 to 162.9)	**-6.4 (-9.57 to -2.63)**	11.4 (7.6 to 16.8)	10.3 (7.0 to 15.0)	**-9.4 (-14.63 to -4.44)**	58.0 (38.6 to 84.9)	47.2 (31.9 to 68.1)	**-18.6 (-22.91 to -14.49)**	40.3 (27.0 to 57.5)	43.0 (28.7 to 61.4)	**6.7 (2.57 to 11.85)**	187.2 (125.9 to 268.2)	182.5 (122.1 to 257.7)	-2.5 (-6.31 to 2.18)
Thalassemias	0.1 (0.1 to 0.2)	0.1 (0.1 to 0.2)	3.5 (-10.08 to 19.42)	0.2 (0.1 to 0.4)	0.2 (0.1 to 0.5)	1.5 (-12.36 to 17.80)	<0.05 (0.0 to 0.1)	<0.05 (0.0 to 0.1)	2.8 (-9.03 to 16.35)	0.2 (0.1 to 0.4)	0.2 (0.1 to 0.4)	0.8 (-11.48 to 15.37)	<0.05 (0.0 to 0.1)	0.1 (0.0 to 0.1)	4.8 (-19.52 to 34.88)	0.2 (0.1 to 0.5)	0.3 (0.1 to 0.5)	2.9 (-22.05 to 34.05)
Thalassemia trait	28.8 (19.5 to 41.3)	30.7 (20.6 to 43.5)	**6.5 (2.87 to 10.19)**	67.0 (45.4 to 95.8)	64.3 (43.6 to 91.3)	**-4.0 (-6.76 to -0.94)**	4.9 (3.2 to 7.2)	4.0 (2.6 to 5.8)	**-17.8 (-26.17 to -9.46)**	25.6 (16.9 to 37.3)	19.1 (12.6 to 27.6)	**-25.2 (-31.25 to -19.11)**	23.9 (16.3 to 34.2)	26.7 (18.0 to 37.9)	**11.5 (7.85 to 15.45)**	108.7 (73.8 to 154.3)	110.0 (74.5 to 155.1)	1.2 (-1.78 to 4.63)
Sickle cell disorders	0.6 (0.4 to 0.9)	0.6 (0.4 to 0.9)	**10.1 (1.33 to 19.85)**	1.3 (0.9 to 2.0)	1.4 (0.9 to 2.0)	2.7 (-5.82 to 11.81)	0.3 (0.2 to 0.4)	0.3 (0.2 to 0.4)	10.8 (-2.77 to 25.52)	1.2 (0.8 to 1.9)	1.3 (0.9 to 1.9)	3.9 (-9.10 to 17.94)	0.3 (0.2 to 0.4)	0.3 (0.2 to 0.5)	**9.7 (-0.82 to 20.11)**	1.4 (0.9 to 2.1)	1.4 (0.9 to 2.1)	1.8 (-8.88 to 12.25)
Sickle cell trait	15.9 (10.4 to 23.2)	14.7 (9.6 to 21.2)	**-7.6 (-14.99 to -0.22)**	38.8 (25.5 to 56.1)	33.5 (22.1 to 48.0)	**-13.6 (-20.15 to -7.13)**	2.2 (1.5 to 3.3)	1.5 (1.0 to 2.3)	**-31.2 (-38.02 to -25.07)**	11.9 (7.8 to 17.5)	7.5 (4.9 to 11.2)	**-36.9 (-44.43 to -31.08)**	13.7 (9.0 to 19.7)	13.1 (8.6 to 19.0)	-3.7 (-11.82 to 4.96)	66.2 (43.8 to 95.8)	60.1 (39.5 to 86.2)	**-9.2 (-16.55 to -1.62)**
G6PD deficiency	0.1 (0.1 to 0.2)	0.1 (0.1 to 0.2)	**-17.8 (-36.91 to 2.96)**	0.3 (0.2 to 0.4)	0.2 (0.1 to 0.3)	**-30.1 (-47.80 to -9.67)**	<0.05 (0.0 to <0.05)	<0.05 (0.0 to <0.05)	-0.9 (-10.67 to 12.00)	0.1 (0.1 to 0.2)	0.1 (0.1 to 0.2)	**-11.6 (-21.24 to 1.43)**	0.1 (0.1 to 0.1)	0.1 (0.0 to 0.1)	**-22.0 (-45.74 to 4.69)**	0.5 (0.3 to 0.7)	0.3 (0.2 to 0.5)	**-35.6 (-57.77 to -9.65)**
G6PD trait	0.5 (0.3 to 0.8)	0.5 (0.3 to 0.8)	0.3 (-20.32 to 22.83)	1.3 (0.8 to 1.9)	1.2 (0.7 to 1.9)	-6.2 (-27.10 to 15.74)	--	--	--	--	--	--	0.5 (0.3 to 0.8)	0.5 (0.3 to 0.8)	0.3 (-20.32 to 22.83)	2.6 (1.5 to 3.9)	2.4 (1.4 to 3.8)	-6.2 (-27.16 to 15.82)
Other hemoglobinopathies and hemolytic anemias	5.7 (3.8 to 8.3)	6.6 (4.2 to 9.9)	**16.1 (2.40 to 35.90)**	13.3 (8.8 to 19.4)	13.5 (8.8 to 20.3)	1.8 (-9.88 to 19.17)	4.0 (2.6 to 5.7)	4.4 (2.9 to 6.5)	**11.9 (2.41 to 20.98)**	18.9 (12.7 to 27.3)	18.9 (12.5 to 27.4)	0.2 (-7.87 to 8.84)	1.7 (1.1 to 2.6)	2.2 (1.3 to 3.7)	26.7 (-7.02 to 95.11)	7.6 (4.8 to 11.5)	8.0 (4.6 to 14.1)	5.9 (-23.53 to 67.35)
Endocrine, metabolic, blood, and immune disorders	25.0 (17.2 to 33.9)	34.9 (24.0 to 47.2)	**39.9 (35.81 to 44.36)**	51.3 (35.1 to 70.0)	61.9 (42.2 to 83.7)	**20.7 (16.64 to 25.56)**	12.6 (8.7 to 17.2)	18.4 (12.7 to 25.0)	**46.8 (42.09 to 51.60)**	55.3 (38.2 to 76.0)	67.9 (46.6 to 92.2)	**22.7 (18.92 to 26.53)**	12.4 (8.5 to 16.9)	16.5 (11.3 to 22.5)	**32.9 (26.94 to 40.61)**	47.3 (32.2 to 65.5)	55.9 (37.8 to 76.8)	**18.4 (11.11 to 28.37)**
**Musculoskeletal disorders**	1,442.8 (1,039.9 to 1,887.5)	1,725.6 (1,240.6 to 2,278.4)	**19.6 (15.55 to 23.41)**	2,618.7 (1,884.4 to 3,433.3)	2,644.5 (1,882.8 to 3,515.1)	1.0 (-2.72 to 4.44)	570.7 (408.9 to 759.8)	699.3 (499.5 to 930.8)	**22.6 (16.75 to 28.88)**	2,215.9 (1,578.7 to 2,946.7)	2,234.8 (1,585.5 to 2,982.1)	0.9 (-4.36 to 6.48)	872.1 (633.1 to 1,139.8)	1,026.3 (736.4 to 1,340.8)	**17.7 (12.07 to 22.77)**	2,985.4 (2,147.1 to 3,934.7)	3,035.3 (2,172.5 to 3,999.5)	1.7 (-3.46 to 6.41)
Rheumatoid arthritis	69.8 (50.8 to 90.9)	71.5 (51.8 to 93.8)	2.5 (-1.69 to 7.02)	109.8 (79.7 to 143.2)	93.0 (67.1 to 122.4)	**-15.3 (-18.64 to -11.77)**	15.8 (11.5 to 20.7)	19.8 (14.3 to 25.9)	**24.9 (17.11 to 33.96)**	57.7 (41.8 to 75.4)	55.6 (40.1 to 73.1)	-3.6 (-9.56 to 3.29)	54.0 (39.2 to 70.4)	51.7 (37.2 to 68.1)	-4.1 (-8.91 to 0.76)	152.6 (110.7 to 200.4)	127.0 (90.8 to 167.6)	**-16.8 (-20.86 to -12.71)**
Osteoarthritis	109.8 (72.4 to 158.1)	136.1 (90.2 to 194.5)	**24.0 (17.10 to 30.01)**	169.0 (110.9 to 243.4)	165.9 (109.7 to 237.1)	-1.8 (-7.59 to 3.46)	36.7 (24.3 to 53.3)	48.9 (32.5 to 70.3)	**33.4 (23.60 to 43.92)**	132.9 (88.0 to 192.8)	130.0 (86.3 to 186.8)	-2.1 (-9.45 to 5.71)	73.1 (48.3 to 105.5)	87.3 (57.8 to 125.8)	**19.4 (11.53 to 27.55)**	197.3 (129.8 to 285.2)	197.6 (130.7 to 284.2)	0.3 (-6.93 to 7.55)
Low back and neck pain	1,077.7 (758.3 to 1,447.0)	1,278.4 (892.6 to 1,738.5)	**18.7 (13.38 to 23.72)**	2,008.3 (1,409.5 to 2,701.9)	2,028.6 (1,404.0 to 2,765.6)	1.0 (-3.89 to 5.51)	479.1 (333.9 to 645.8)	569.6 (394.4 to 766.8)	**19.0 (12.34 to 26.29)**	1,879.0 (1,306.0 to 2,529.5)	1,865.7 (1,287.6 to 2,516.5)	-0.7 (-6.42 to 5.60)	598.6 (421.4 to 799.6)	708.8 (494.6 to 959.0)	**18.4 (10.72 to 25.76)**	2,128.4 (1,502.7 to 2,857.2)	2,185.2 (1,517.9 to 2,956.0)	2.7 (-4.24 to 9.45)
Low back pain	700.6 (484.1 to 969.8)	845.2 (573.6 to 1,172.3)	**20.7 (14.08 to 27.96)**	1,299.0 (895.2 to 1,805.2)	1,334.9 (902.5 to 1,855.2)	2.8 (-3.14 to 9.50)	324.3 (223.1 to 455.0)	387.8 (262.7 to 537.2)	**19.7 (9.93 to 30.96)**	1,273.5 (873.2 to 1,787.9)	1,267.7 (857.4 to 1,751.8)	-0.4 (-8.73 to 10.05)	376.2 (257.6 to 520.3)	457.4 (309.7 to 632.7)	**21.6 (12.27 to 31.07)**	1,320.6 (905.9 to 1,833.9)	1,399.0 (939.3 to 1,947.4)	6.0 (-3.00 to 15.06)
Neck pain	377.1 (264.1 to 518.2)	433.2 (305.6 to 599.0)	**15.0 (3.45 to 21.90)**	709.3 (497.3 to 977.3)	693.7 (486.9 to 959.7)	-2.1 (-11.95 to 3.86)	154.8 (107.6 to 215.6)	181.8 (126.5 to 250.9)	**17.6 (9.51 to 26.07)**	605.5 (420.5 to 842.9)	598.0 (415.0 to 826.2)	-1.1 (-7.82 to 5.80)	222.3 (155.5 to 304.6)	251.4 (174.5 to 347.8)	**13.2 (-2.11 to 24.37)**	807.8 (563.5 to 1,110.4)	786.2 (543.9 to 1,091.0)	-2.5 (-16.24 to 7.16)
Gout	3.3 (2.3 to 4.5)	4.2 (2.9 to 5.7)	**26.8 (17.64 to 36.48)**	5.5 (3.8 to 7.4)	5.5 (3.8 to 7.5)	1.1 (-6.28 to 8.88)	2.3 (1.6 to 3.1)	3.0 (2.1 to 4.0)	**31.5 (20.31 to 43.87)**	8.4 (5.8 to 11.3)	8.3 (5.7 to 11.3)	-0.2 (-8.92 to 8.98)	1.1 (0.7 to 1.4)	1.2 (0.9 to 1.7)	**16.7 (5.13 to 30.42)**	3.0 (2.1 to 4.1)	3.0 (2.1 to 4.0)	-0.9 (-10.84 to 10.38)
Other musculoskeletal disorders	182.2 (120.1 to 258.6)	235.3 (156.6 to 338.0)	**29.2 (24.13 to 33.72)**	326.1 (214.8 to 464.3)	351.4 (231.5 to 504.3)	**7.8 (4.26 to 11.40)**	36.8 (21.7 to 58.4)	58.1 (35.2 to 90.2)	**58.3 (46.06 to 73.59)**	138.0 (81.6 to 218.6)	175.2 (105.2 to 272.7)	**27.5 (17.13 to 41.19)**	145.3 (97.2 to 203.9)	177.3 (118.8 to 249.7)	**22.0 (16.73 to 26.63)**	504.2 (337.0 to 711.9)	522.4 (347.6 to 734.5)	**3.7 (-0.27 to 7.00)**
**Other non-communicable diseases**	988.4 (665.0 to 1,430.1)	1,109.0 (750.4 to 1,586.1)	**12.3 (9.88 to 14.82)**	1,810.0 (1,210.7 to 2,643.3)	1,717.3 (1,155.3 to 2,497.0)	**-5.1 (-7.10 to -2.93)**	483.1 (323.6 to 700.2)	559.8 (379.4 to 807.9)	**16.0 (12.11 to 19.74)**	1,940.0 (1,299.9 to 2,817.1)	1,814.6 (1,223.6 to 2,633.3)	**-6.4 (-9.71 to -3.56)**	505.3 (340.1 to 723.4)	549.1 (370.4 to 782.0)	**8.7 (6.22 to 11.19)**	1,700.2 (1,142.1 to 2,455.5)	1,630.6 (1,099.5 to 2,352.3)	**-4.1 (-6.23 to -2.03)**
Congenital anomalies	79.3 (57.6 to 103.1)	96.6 (69.7 to 125.2)	**21.8 (14.89 to 29.11)**	165.2 (118.7 to 215.6)	175.5 (125.3 to 230.2)	**6.2 (0.12 to 12.70)**	40.2 (29.0 to 52.7)	49.5 (35.4 to 64.8)	**23.2 (13.48 to 33.60)**	171.3 (122.9 to 226.1)	182.5 (129.5 to 241.1)	6.5 (-2.01 to 16.04)	39.1 (28.6 to 50.1)	47.1 (34.3 to 60.9)	**20.4 (11.91 to 31.13)**	159.3 (114.7 to 205.7)	168.5 (121.6 to 220.4)	5.9 (-1.78 to 15.53)
Neural tube defects	4.9 (3.4 to 6.4)	5.3 (3.6 to 6.9)	8.5 (-4.11 to 21.63)	11.1 (7.8 to 14.6)	10.9 (7.5 to 14.3)	-2.0 (-13.68 to 10.03)	2.1 (1.5 to 2.9)	2.4 (1.6 to 3.2)	10.9 (-7.96 to 32.41)	9.7 (6.8 to 13.0)	9.7 (6.6 to 13.1)	0.3 (-17.08 to 20.03)	2.7 (1.9 to 3.6)	2.9 (2.0 to 3.8)	7.2 (-10.20 to 26.73)	12.5 (8.6 to 16.5)	12.1 (8.3 to 15.8)	-3.5 (-19.18 to 14.83)
Congenital heart anomalies	15.2 (6.5 to 26.3)	16.3 (6.9 to 27.8)	7.5 (-1.86 to 18.30)	33.2 (14.2 to 57.6)	32.0 (13.7 to 54.5)	-3.5 (-11.78 to 6.10)	7.6 (3.3 to 13.0)	8.1 (3.5 to 14.0)	7.5 (-4.64 to 22.41)	33.4 (14.5 to 57.1)	32.1 (13.9 to 54.9)	-3.9 (-14.63 to 9.33)	7.6 (3.2 to 13.2)	8.1 (3.5 to 14.2)	7.6 (-4.24 to 19.75)	32.9 (13.9 to 57.4)	31.8 (13.7 to 55.4)	-3.0 (-13.66 to 7.70)
Orofacial clefts	0.7 (0.4 to 1.0)	0.7 (0.4 to 0.9)	-1.9 (-13.98 to 10.66)	1.4 (1.0 to 2.1)	1.3 (0.8 to 1.8)	**-13.1 (-24.19 to -1.60)**	0.4 (0.2 to 0.5)	0.4 (0.2 to 0.5)	-1.7 (-17.05 to 16.63)	1.6 (1.0 to 2.3)	1.4 (0.9 to 2.0)	-13.1 (-26.76 to 3.90)	0.3 (0.2 to 0.5)	0.3 (0.2 to 0.4)	-1.6 (-19.13 to 20.18)	1.3 (0.9 to 1.9)	1.1 (0.8 to 1.7)	-12.7 (-28.54 to 6.86)
Down syndrome	9.9 (7.5 to 12.6)	12.8 (9.5 to 16.2)	**29.5 (11.98 to 49.78)**	19.1 (14.5 to 24.4)	20.6 (15.2 to 26.5)	8.1 (-6.26 to 25.25)	4.8 (3.5 to 6.1)	6.2 (4.5 to 8.1)	**31.7 (8.58 to 57.85)**	19.1 (14.3 to 24.7)	20.6 (15.0 to 27.1)	8.2 (-10.98 to 29.76)	5.1 (3.9 to 6.6)	6.5 (4.9 to 8.8)	**28.0 (4.39 to 60.34)**	19.1 (14.2 to 24.6)	20.6 (15.2 to 27.5)	8.5 (-11.57 to 35.12)
Turner syndrome	<0.05 (0.0 to 0.1)	<0.05 (0.0 to 0.1)	**14.7 (-0.97 to 34.99)**	0.1 (0.0 to 0.1)	0.1 (0.0 to 0.1)	3.9 (-9.99 to 22.54)	--	--	--	--	--	--	<0.05 (0.0 to 0.1)	<0.05 (0.0 to 0.1)	**14.7 (-0.97 to 34.99)**	0.2 (0.1 to 0.3)	0.2 (0.1 to 0.3)	4.0 (-9.96 to 22.58)
Klinefelter syndrome	<0.05 (0.0 to <0.05)	<0.05 (0.0 to <0.05)	**31.0 (7.55 to 57.04)**	<0.05 (0.0 to <0.05)	<0.05 (0.0 to 0.1)	**19.6 (-1.77 to 42.95)**	<0.05 (0.0 to <0.05)	<0.05 (0.0 to <0.05)	**31.0 (7.55 to 57.04)**	<0.05 (0.0 to 0.1)	0.1 (0.0 to 0.1)	**19.3 (-2.06 to 42.47)**	--	--	--	--	--	--
Chromosomal unbalanced rearrangements	19.7 (14.7 to 25.0)	25.5 (19.1 to 32.3)	**29.4 (16.09 to 46.22)**	38.2 (28.4 to 48.9)	41.2 (30.9 to 52.7)	8.2 (-2.44 to 22.26)	9.4 (7.0 to 12.0)	12.4 (9.2 to 16.0)	**32.9 (13.09 to 58.96)**	37.7 (28.1 to 48.7)	41.1 (30.2 to 53.5)	9.4 (-6.59 to 30.46)	10.4 (7.6 to 13.3)	13.1 (9.7 to 16.9)	**26.7 (6.93 to 48.84)**	38.6 (28.0 to 49.6)	41.4 (30.7 to 53.6)	7.5 (-9.64 to 26.38)
Other congenital anomalies	29.0 (19.2 to 40.4)	36.1 (23.4 to 50.6)	**24.5 (10.44 to 40.39)**	62.1 (41.1 to 86.5)	69.4 (45.2 to 97.8)	**11.8 (-1.15 to 26.60)**	16.0 (10.4 to 22.9)	20.0 (12.8 to 28.7)	**25.2 (8.76 to 44.88)**	69.7 (45.4 to 99.3)	77.5 (49.6 to 111.7)	11.4 (-3.46 to 29.10)	13.0 (8.6 to 18.1)	16.1 (10.3 to 23.0)	**23.9 (5.65 to 45.82)**	54.7 (36.1 to 76.7)	61.3 (39.9 to 87.4)	12.4 (-4.48 to 32.66)
Skin and subcutaneous diseases	354.2 (228.9 to 545.6)	376.3 (247.6 to 570.0)	**6.4 (2.06 to 10.61)**	750.7 (483.7 to 1,159.2)	718.5 (467.1 to 1,082.6)	**-4.2 (-7.97 to -0.57)**	181.8 (115.5 to 281.3)	191.1 (125.0 to 290.6)	5.4 (-1.70 to 11.11)	784.8 (496.7 to 1,216.2)	734.4 (473.5 to 1,124.2)	**-6.2 (-12.46 to -1.23)**	172.4 (113.7 to 257.2)	185.2 (122.7 to 274.4)	**7.5 (3.02 to 11.80)**	717.2 (469.9 to 1,090.6)	703.2 (461.4 to 1,059.3)	-1.9 (-6.16 to 2.36)
Dermatitis	104.1 (66.0 to 152.1)	113.9 (72.0 to 166.7)	**9.5 (7.64 to 11.13)**	226.1 (142.3 to 328.7)	224.7 (141.8 to 326.1)	-0.6 (-1.49 to 0.31)	48.3 (30.4 to 70.5)	53.2 (33.6 to 77.8)	**10.1 (7.99 to 12.09)**	212.2 (133.6 to 310.0)	211.2 (133.8 to 307.4)	-0.4 (-1.68 to 0.87)	55.8 (35.3 to 81.3)	60.8 (38.4 to 88.4)	**8.9 (6.88 to 10.66)**	239.7 (150.5 to 350.2)	238.3 (149.7 to 347.1)	-0.6 (-1.77 to 0.65)
Psoriasis	56.8 (38.8 to 79.5)	65.5 (44.6 to 91.5)	**15.3 (12.76 to 17.64)**	110.4 (75.2 to 153.5)	110.5 (75.5 to 153.2)	0.1 (-1.37 to 1.66)	28.7 (19.3 to 40.5)	33.6 (22.7 to 47.4)	**17.2 (14.26 to 20.35)**	115.0 (77.9 to 161.5)	115.3 (78.4 to 161.8)	0.3 (-1.67 to 2.20)	28.1 (19.0 to 39.5)	31.8 (21.6 to 45.0)	**13.2 (10.12 to 16.42)**	105.9 (71.8 to 148.2)	105.9 (72.4 to 148.1)	-0.0 (-2.33 to 2.21)
Cellulitis	2.0 (1.3 to 2.9)	2.6 (1.7 to 3.7)	**30.9 (18.50 to 44.72)**	3.6 (2.4 to 5.3)	3.8 (2.5 to 5.4)	3.8 (-4.97 to 13.66)	1.0 (0.7 to 1.5)	1.4 (0.9 to 2.0)	**31.3 (17.71 to 46.54)**	4.3 (2.8 to 6.1)	4.4 (2.8 to 6.3)	3.4 (-6.30 to 13.87)	0.9 (0.6 to 1.4)	1.2 (0.8 to 1.8)	**30.5 (15.70 to 46.63)**	3.0 (2.0 to 4.5)	3.2 (2.0 to 4.6)	4.3 (-6.89 to 16.66)
Pyoderma	0.3 (0.1 to 0.7)	0.4 (0.1 to 0.8)	**16.5 (12.70 to 20.89)**	0.7 (0.3 to 1.5)	0.7 (0.3 to 1.5)	**4.1 (1.12 to 7.26)**	0.2 (0.1 to 0.4)	0.2 (0.1 to 0.4)	**16.2 (11.37 to 21.02)**	0.7 (0.3 to 1.7)	0.8 (0.3 to 1.7)	2.3 (-1.17 to 5.97)	0.1 (0.1 to 0.3)	0.2 (0.1 to 0.4)	**17.0 (12.24 to 22.33)**	0.6 (0.2 to 1.2)	0.6 (0.2 to 1.3)	**6.2 (1.51 to 10.73)**
Scabies	0.8 (0.5 to 1.3)	0.7 (0.4 to 1.1)	**-14.6 (-22.20 to -6.41)**	1.8 (1.0 to 2.8)	1.4 (0.8 to 2.2)	**-21.5 (-28.36 to -13.78)**	0.5 (0.3 to 0.8)	0.4 (0.2 to 0.6)	**-25.1 (-33.88 to -13.75)**	2.2 (1.2 to 3.5)	1.5 (0.8 to 2.4)	**-30.9 (-38.97 to -20.78)**	0.3 (0.2 to 0.5)	0.3 (0.2 to 0.5)	1.7 (-9.62 to 16.09)	1.4 (0.8 to 2.2)	1.3 (0.7 to 2.0)	-6.6 (-17.32 to 5.86)
Fungal skin diseases	19.7 (8.0 to 41.3)	22.9 (9.3 to 47.8)	**16.5 (15.24 to 17.77)**	38.4 (15.5 to 81.8)	38.5 (15.5 to 81.9)	**0.4 (0.04 to 0.70)**	11.9 (4.9 to 25.1)	14.0 (5.7 to 29.3)	**17.9 (16.08 to 19.66)**	48.1 (19.7 to 103.1)	48.2 (19.8 to 103.2)	0.1 (-0.29 to 0.50)	7.8 (3.1 to 16.4)	8.9 (3.6 to 18.7)	**14.4 (13.39 to 15.41)**	29.1 (11.7 to 61.3)	29.1 (11.7 to 61.3)	0.0 (-0.47 to 0.51)
Viral skin diseases	30.0 (17.5 to 47.3)	32.3 (18.9 to 50.3)	**8.0 (5.54 to 10.87)**	70.0 (40.9 to 109.0)	70.3 (41.2 to 109.2)	0.5 (-0.53 to 1.69)	14.5 (8.3 to 22.7)	15.6 (8.9 to 24.5)	**7.9 (4.99 to 10.95)**	68.4 (39.0 to 106.2)	68.3 (39.0 to 106.1)	-0.1 (-1.76 to 1.54)	15.5 (9.0 to 24.4)	16.7 (9.9 to 26.1)	**8.0 (5.27 to 11.41)**	71.4 (42.0 to 110.7)	72.3 (42.7 to 111.7)	1.3 (-0.29 to 2.91)
Acne vulgaris	86.8 (40.8 to 161.2)	73.0 (34.5 to 135.7)	**-15.5 (-25.50 to -0.67)**	200.9 (94.6 to 372.7)	167.1 (79.3 to 309.8)	**-16.5 (-26.46 to -3.08)**	49.2 (22.8 to 92.8)	38.3 (17.9 to 70.9)	**-21.5 (-37.59 to -3.49)**	224.7 (104.1 to 425.0)	173.4 (81.4 to 320.5)	**-22.2 (-38.06 to -6.19)**	37.6 (17.4 to 69.9)	34.7 (16.2 to 64.9)	-7.3 (-21.40 to 9.80)	176.7 (81.6 to 329.9)	160.7 (74.6 to 300.7)	-8.7 (-22.32 to 8.03)
Alopecia areata	2.8 (1.8 to 4.1)	3.2 (2.1 to 4.8)	**18.1 (12.95 to 23.31)**	5.3 (3.4 to 7.8)	5.3 (3.4 to 7.8)	0.1 (-4.04 to 4.31)	1.5 (1.0 to 2.2)	1.8 (1.2 to 2.7)	**21.1 (14.11 to 28.28)**	6.0 (3.9 to 8.9)	5.9 (3.8 to 8.9)	-0.7 (-6.62 to 5.15)	1.3 (0.8 to 1.9)	1.5 (0.9 to 2.2)	**14.6 (6.89 to 23.04)**	4.7 (3.0 to 7.0)	4.7 (3.0 to 6.9)	-0.2 (-6.61 to 6.53)
Pruritus	0.1 (0.0 to 0.1)	0.1 (0.0 to 0.1)	12.5 (-5.48 to 32.59)	0.1 (0.1 to 0.2)	0.1 (0.1 to 0.2)	-6.9 (-21.90 to 9.70)	<0.05 (0.0 to 0.1)	<0.05 (0.0 to 0.1)	13.2 (-22.28 to 47.92)	0.1 (0.0 to 0.2)	0.1 (0.0 to 0.2)	-10.4 (-37.44 to 15.30)	<0.05 (0.0 to 0.1)	<0.05 (0.0 to 0.1)	12.8 (-8.81 to 41.67)	0.1 (0.1 to 0.3)	0.1 (0.1 to 0.3)	-4.0 (-23.07 to 19.97)
Urticaria	23.3 (15.1 to 33.4)	29.2 (18.6 to 42.6)	**25.6 (7.75 to 45.17)**	45.6 (29.2 to 65.8)	49.2 (31.4 to 71.7)	8.2 (-5.53 to 24.53)	12.0 (7.5 to 17.3)	15.6 (9.8 to 23.2)	**30.4 (7.99 to 62.66)**	49.8 (31.1 to 71.5)	53.7 (33.4 to 79.6)	8.1 (-9.18 to 36.64)	11.3 (7.2 to 16.3)	13.6 (8.7 to 19.5)	**21.2 (0.26 to 48.54)**	41.5 (26.2 to 60.7)	44.9 (28.2 to 64.6)	8.9 (-8.96 to 35.88)
Decubitus ulcer	3.7 (2.6 to 4.9)	3.6 (2.5 to 4.9)	-2.0 (-14.47 to 13.23)	5.3 (3.8 to 7.1)	4.1 (2.8 to 5.6)	**-22.7 (-31.71 to -12.03)**	1.8 (1.2 to 2.5)	1.8 (1.2 to 2.5)	0.4 (-14.82 to 17.48)	6.5 (4.5 to 8.9)	4.7 (3.3 to 6.5)	**-26.8 (-37.30 to -15.18)**	1.9 (1.3 to 2.5)	1.8 (1.2 to 2.5)	-3.8 (-24.15 to 25.20)	4.4 (3.1 to 5.8)	3.5 (2.4 to 4.9)	**-18.1 (-34.24 to 3.49)**
Other skin and subcutaneous diseases	24.0 (9.6 to 53.9)	28.9 (11.2 to 65.7)	**20.1 (15.35 to 23.80)**	42.7 (18.2 to 90.4)	42.7 (18.2 to 90.6)	-0.0 (-1.43 to 1.12)	12.3 (5.1 to 27.1)	15.3 (6.1 to 34.7)	**23.9 (17.12 to 29.40)**	47.0 (20.0 to 99.9)	46.9 (19.9 to 100.3)	-0.2 (-1.80 to 1.33)	11.7 (4.5 to 26.8)	13.6 (5.2 to 31.2)	**16.2 (12.24 to 20.35)**	38.8 (16.5 to 82.0)	38.7 (16.4 to 82.3)	-0.3 (-2.02 to 1.26)
Sense organ diseases	380.9 (257.0 to 539.5)	444.8 (299.3 to 627.9)	**16.9 (12.12 to 21.33)**	603.9 (406.9 to 865.3)	557.8 (372.5 to 800.1)	**-7.6 (-11.60 to -4.56)**	185.7 (125.2 to 266.8)	231.6 (157.7 to 328.5)	**24.9 (19.05 to 32.11)**	702.3 (476.1 to 1,008.4)	640.3 (436.8 to 920.4)	**-8.8 (-13.33 to -4.28)**	195.2 (130.5 to 274.8)	213.2 (141.5 to 301.9)	**9.3 (4.36 to 14.22)**	526.1 (345.7 to 747.9)	485.7 (316.1 to 698.1)	**-7.7 (-11.86 to -3.23)**
Glaucoma	5.8 (3.8 to 8.2)	5.1 (3.2 to 7.3)	-12.0 (-22.26 to 2.36)	8.7 (5.6 to 12.2)	6.0 (3.8 to 8.6)	**-30.3 (-38.49 to -19.63)**	1.9 (1.2 to 2.7)	1.8 (1.1 to 2.5)	-4.2 (-17.68 to 14.03)	6.9 (4.5 to 9.8)	4.8 (3.1 to 6.8)	**-31.1 (-39.72 to -19.31)**	4.0 (2.5 to 5.7)	3.3 (2.0 to 4.8)	**-15.5 (-27.31 to 0.73)**	9.9 (6.4 to 14.1)	7.1 (4.3 to 10.1)	**-28.2 (-38.08 to -16.32)**
Cataract	10.2 (6.6 to 14.8)	9.5 (6.0 to 13.9)	-7.5 (-17.74 to 3.78)	13.7 (8.8 to 19.6)	9.5 (6.1 to 13.9)	**-30.7 (-37.88 to -20.75)**	3.1 (1.9 to 4.6)	3.3 (2.0 to 5.1)	7.1 (-13.52 to 27.00)	11.6 (7.1 to 16.9)	8.0 (4.9 to 12.1)	**-31.0 (-42.06 to -20.11)**	7.1 (4.6 to 10.6)	6.1 (3.9 to 8.9)	**-13.6 (-26.99 to 1.76)**	15.1 (9.8 to 22.1)	10.6 (6.9 to 15.3)	**-29.2 (-39.08 to -15.57)**
Macular degeneration	14.4 (9.3 to 20.5)	17.3 (11.2 to 24.9)	**20.5 (10.66 to 33.72)**	20.2 (13.1 to 28.7)	18.3 (12.0 to 25.9)	**-9.4 (-15.79 to -1.65)**	4.5 (2.9 to 6.5)	6.5 (4.2 to 9.2)	**44.9 (29.26 to 63.24)**	16.7 (10.8 to 24.0)	16.0 (10.5 to 22.9)	-3.8 (-12.67 to 6.49)	9.9 (6.2 to 14.3)	10.9 (7.0 to 15.8)	**9.7 (-1.26 to 24.72)**	22.1 (14.4 to 31.3)	19.9 (13.0 to 28.3)	**-9.8 (-18.22 to 0.12)**
Uncorrected refractive error	78.7 (44.8 to 129.8)	88.9 (50.1 to 149.6)	**12.8 (4.49 to 21.81)**	134.0 (76.1 to 224.0)	123.8 (70.1 to 210.0)	**-7.7 (-14.70 to -0.39)**	39.5 (21.7 to 66.7)	47.4 (25.9 to 82.0)	**20.0 (5.89 to 36.40)**	152.0 (83.8 to 257.3)	141.0 (77.8 to 243.7)	-7.2 (-17.98 to 5.45)	39.2 (22.8 to 63.4)	41.5 (24.2 to 68.1)	5.9 (-3.17 to 14.50)	118.1 (68.8 to 192.8)	107.6 (62.4 to 179.2)	**-8.9 (-16.74 to -0.78)**
Age-related and other hearing loss	230.7 (153.3 to 329.0)	280.5 (187.4 to 401.7)	**21.7 (15.20 to 28.63)**	347.6 (233.9 to 499.2)	326.3 (216.1 to 466.4)	**-6.1 (-11.60 to -1.13)**	119.6 (80.1 to 171.2)	153.9 (103.1 to 218.1)	**28.9 (21.10 to 38.35)**	443.3 (301.0 to 633.1)	404.4 (268.4 to 576.3)	**-8.7 (-14.61 to -3.07)**	111.0 (71.6 to 161.1)	126.6 (81.9 to 184.0)	**14.1 (5.98 to 22.00)**	273.5 (177.6 to 401.2)	258.9 (166.5 to 384.5)	-5.3 (-12.64 to 1.90)
Other vision loss	10.8 (7.2 to 15.0)	9.1 (6.0 to 13.2)	**-16.0 (-22.79 to -7.24)**	17.6 (11.8 to 24.7)	11.9 (8.0 to 17.3)	**-32.3 (-37.56 to -25.39)**	5.0 (3.3 to 7.2)	4.6 (3.1 to 6.7)	-7.4 (-19.24 to 6.71)	19.0 (12.9 to 26.8)	13.3 (8.9 to 19.1)	**-30.3 (-37.95 to -20.53)**	5.8 (3.8 to 8.3)	4.5 (2.9 to 6.6)	**-23.2 (-30.27 to -15.95)**	16.6 (10.9 to 23.4)	10.8 (7.1 to 15.8)	**-35.0 (-41.59 to -28.37)**
Other sense organ diseases	30.3 (18.8 to 44.9)	34.5 (21.6 to 50.5)	**13.8 (10.92 to 16.65)**	62.1 (38.4 to 92.4)	61.9 (38.5 to 91.4)	-0.2 (-2.63 to 2.31)	12.1 (7.5 to 17.9)	14.1 (8.8 to 20.8)	**16.8 (12.60 to 20.71)**	52.7 (32.8 to 78.3)	52.9 (32.6 to 78.1)	0.2 (-3.38 to 3.69)	18.2 (11.2 to 27.0)	20.3 (12.7 to 29.7)	**11.9 (8.34 to 15.75)**	71.0 (43.8 to 105.8)	70.8 (44.2 to 104.3)	-0.2 (-3.25 to 3.26)
Oral disorders	174.0 (109.9 to 256.3)	191.2 (120.8 to 284.1)	**9.8 (5.86 to 13.79)**	290.2 (181.9 to 433.1)	265.6 (165.1 to 401.4)	**-8.6 (-11.84 to -5.32)**	75.4 (46.5 to 112.9)	87.6 (54.2 to 131.4)	**16.1 (11.16 to 21.18)**	281.6 (173.1 to 423.6)	257.5 (157.8 to 392.6)	**-8.7 (-12.69 to -4.41)**	98.7 (63.7 to 143.0)	103.7 (66.3 to 152.2)	**5.0 (0.18 to 9.60)**	297.6 (189.5 to 441.3)	273.3 (172.4 to 411.4)	**-8.3 (-12.27 to -4.51)**

Deciduous caries	0.4 (0.2 to 0.8)	0.4 (0.2 to 0.8)	3.0 (-1.79 to 7.56)	1.2 (0.5 to 2.3)	1.2 (0.5 to 2.3)	-0.6 (-5.14 to 3.89)	0.2 (0.1 to 0.4)	0.2 (0.1 to 0.4)	3.5 (-2.91 to 10.26)	1.2 (0.6 to 2.4)	1.2 (0.6 to 2.4)	-0.0 (-6.23 to 6.45)	0.2 (0.1 to 0.4)	0.2 (0.1 to 0.4)	2.3 (-4.37 to 9.45)	1.1 (0.5 to 2.2)	1.1 (0.5 to 2.2)	-1.1 (-7.59 to 5.75)
Permanent caries	10.6 (4.8 to 20.3)	12.0 (5.5 to 22.9)	**13.4 (8.19 to 18.43)**	22.1 (10.1 to 42.5)	22.4 (10.2 to 42.8)	1.4 (-3.21 to 5.77)	5.2 (2.4 to 10.1)	6.0 (2.8 to 11.5)	**15.3 (4.22 to 23.99)**	22.2 (10.0 to 43.0)	22.7 (10.4 to 43.5)	2.3 (-7.13 to 10.02)	5.4 (2.5 to 10.5)	6.0 (2.7 to 11.6)	**11.7 (5.00 to 21.40)**	22.0 (10.1 to 42.8)	22.2 (10.0 to 43.3)	0.6 (-5.42 to 8.48)
Periodontal diseases	31.4 (12.6 to 64.1)	39.8 (15.7 to 81.8)	**26.5 (19.54 to 35.85)**	56.4 (22.5 to 115.1)	58.3 (23.1 to 120.0)	3.3 (-2.35 to 10.65)	17.6 (7.0 to 36.2)	22.1 (8.8 to 46.0)	**25.7 (15.09 to 38.88)**	66.8 (26.4 to 137.3)	67.1 (26.6 to 139.0)	0.4 (-7.85 to 10.69)	13.8 (5.6 to 28.1)	17.7 (7.0 to 36.2)	**27.8 (19.28 to 38.44)**	46.9 (19.0 to 95.6)	49.9 (19.8 to 102.4)	**6.4 (-0.48 to 15.06)**
Edentulism and severe tooth loss	105.3 (71.0 to 143.6)	109.1 (74.6 to 150.0)	3.6 (-1.75 to 9.42)	158.5 (106.4 to 216.7)	131.7 (90.1 to 181.5)	**-16.9 (-21.28 to -12.31)**	41.3 (27.6 to 56.8)	46.5 (31.5 to 64.1)	**12.6 (5.30 to 20.61)**	146.6 (98.1 to 201.5)	121.8 (82.4 to 168.3)	**-16.9 (-22.32 to -11.03)**	64.0 (43.5 to 87.7)	62.6 (42.7 to 86.4)	-2.2 (-8.36 to 4.02)	168.6 (114.6 to 231.9)	140.9 (95.8 to 194.8)	**-16.4 (-21.67 to -10.93)**
Other oral disorders	26.2 (16.6 to 38.7)	29.9 (18.8 to 44.5)	**13.9 (10.84 to 17.16)**	51.9 (32.9 to 76.6)	52.0 (32.7 to 77.5)	0.1 (-2.66 to 2.99)	11.0 (7.0 to 16.3)	12.7 (8.1 to 18.8)	**15.3 (10.92 to 19.80)**	44.8 (28.5 to 66.3)	44.7 (28.4 to 66.3)	-0.2 (-3.91 to 3.88)	15.2 (9.6 to 22.5)	17.2 (10.8 to 25.6)	**13.0 (9.12 to 17.08)**	58.9 (37.2 to 87.2)	59.2 (37.1 to 88.1)	0.4 (-3.15 to 4.08)
**Injuries**	551.7 (422.5 to 706.4)	442.1 (319.0 to 593.2)	**-20.0 (-28.55 to -11.16)**	904.4 (691.4 to 1,159.4)	588.2 (424.6 to 789.4)	**-35.1 (-41.60 to -28.21)**	281.7 (214.7 to 363.0)	245.5 (177.7 to 328.7)	**-13.0 (-20.77 to -5.15)**	1,091.1 (832.1 to 1,405.2)	734.7 (533.4 to 984.5)	**-32.8 (-38.71 to -26.73)**	270.0 (206.7 to 343.4)	196.6 (141.2 to 264.2)	**-27.3 (-36.76 to -17.22)**	695.3 (533.6 to 887.7)	440.9 (316.1 to 596.7)	**-36.7 (-44.30 to -28.80)**
**Transport injuries**	80.7 (60.7 to 103.4)	40.8 (29.6 to 54.2)	**-49.5 (-54.61 to -43.75)**	150.4 (113.6 to 192.6)	61.5 (44.7 to 81.6)	**-59.2 (-63.41 to -54.43)**	58.7 (44.1 to 75.2)	31.3 (22.6 to 41.5)	**-46.8 (-52.05 to -40.78)**	229.7 (172.8 to 294.2)	97.8 (71.1 to 129.7)	**-57.5 (-61.69 to -52.74)**	22.0 (16.6 to 28.2)	9.5 (6.9 to 12.7)	**-56.7 (-61.35 to -51.65)**	75.7 (57.0 to 96.7)	26.9 (19.7 to 35.9)	**-64.5 (-68.44 to -60.09)**
Road injuries	74.8 (56.3 to 95.8)	32.2 (23.4 to 42.8)	**-57.0 (-61.20 to -52.14)**	140.0 (106.0 to 178.9)	49.0 (35.6 to 64.9)	**-65.0 (-68.57 to -61.00)**	54.4 (40.9 to 69.7)	24.5 (17.8 to 32.5)	**-55.0 (-59.40 to -49.95)**	213.5 (160.6 to 273.3)	77.3 (56.3 to 102.2)	**-63.8 (-67.39 to -59.83)**	20.4 (15.4 to 26.0)	7.7 (5.6 to 10.3)	**-62.1 (-66.00 to -57.75)**	70.6 (52.9 to 90.1)	22.0 (16.0 to 29.2)	**-68.9 (-72.27 to -64.99)**
Pedestrian road injuries	15.2 (11.4 to 19.8)	5.7 (4.0 to 7.8)	**-62.7 (-66.49 to -58.59)**	28.1 (21.0 to 36.5)	8.5 (6.1 to 11.7)	**-69.7 (-72.85 to -66.10)**	10.4 (7.8 to 13.6)	4.2 (3.0 to 5.7)	**-60.0 (-64.08 to -55.59)**	41.1 (30.8 to 53.4)	13.1 (9.3 to 17.8)	**-68.1 (-71.44 to -64.61)**	4.8 (3.6 to 6.2)	1.5 (1.1 to 2.0)	**-68.7 (-71.98 to -65.16)**	16.1 (12.1 to 21.0)	4.2 (3.0 to 5.7)	**-74.1 (-76.95 to -70.93)**
Cyclist road injuries	7.4 (5.6 to 9.5)	2.7 (1.9 to 3.6)	**-63.6 (-68.17 to -58.36)**	14.2 (10.7 to 18.2)	4.3 (3.1 to 5.7)	**-69.6 (-73.32 to -65.14)**	5.4 (4.1 to 6.9)	2.1 (1.5 to 2.7)	**-61.9 (-66.49 to -56.27)**	21.6 (16.4 to 27.7)	6.8 (4.9 to 9.0)	**-68.5 (-72.32 to -64.03)**	2.0 (1.5 to 2.5)	0.6 (0.4 to 0.8)	**-68.5 (-72.57 to -63.64)**	7.2 (5.4 to 9.3)	1.9 (1.4 to 2.6)	**-73.3 (-76.83 to -69.07)**
Motorcyclist road injuries	14.1 (10.5 to 18.0)	5.7 (4.1 to 7.8)	**-59.4 (-64.89 to -52.90)**	26.9 (20.1 to 34.3)	8.8 (6.3 to 11.9)	**-67.2 (-71.66 to -61.82)**	12.3 (9.2 to 15.8)	5.4 (3.8 to 7.3)	**-56.6 (-62.41 to -49.86)**	48.3 (36.1 to 61.8)	16.9 (12.1 to 22.8)	**-65.1 (-69.77 to -59.65)**	1.8 (1.3 to 2.3)	0.4 (0.3 to 0.5)	**-79.1 (-82.90 to -74.31)**	6.5 (4.8 to 8.4)	1.1 (0.8 to 1.5)	**-83.1 (-86.18 to -78.99)**
Motor vehicle road injuries	37.6 (28.5 to 47.9)	18.0 (13.2 to 23.6)	**-52.2 (-56.47 to -47.66)**	69.9 (52.8 to 89.0)	27.1 (20.0 to 35.5)	**-61.3 (-64.77 to -57.45)**	26.0 (19.7 to 33.0)	12.8 (9.4 to 16.9)	**-50.6 (-54.81 to -45.98)**	101.5 (77.0 to 128.9)	40.3 (29.8 to 52.7)	**-60.3 (-63.70 to -56.69)**	11.6 (8.8 to 14.8)	5.2 (3.8 to 6.8)	**-55.8 (-60.11 to -51.03)**	40.0 (30.3 to 50.8)	14.5 (10.7 to 19.1)	**-63.8 (-67.43 to -59.49)**
Other road injuries	0.5 (0.4 to 0.6)	0.2 (0.1 to 0.2)	**-69.1 (-72.69 to -64.85)**	0.9 (0.7 to 1.1)	0.2 (0.2 to 0.3)	**-74.7 (-77.79 to -71.16)**	0.3 (0.2 to 0.3)	0.1 (0.0 to 0.1)	**-76.0 (-78.75 to -72.69)**	1.1 (0.8 to 1.3)	0.2 (0.1 to 0.3)	**-80.6 (-82.91 to -77.94)**	0.2 (0.2 to 0.3)	0.1 (0.1 to 0.1)	**-60.7 (-65.47 to -55.12)**	0.8 (0.6 to 1.0)	0.3 (0.2 to 0.3)	**-67.4 (-71.52 to -62.39)**
Other transport injuries	5.9 (4.4 to 7.6)	8.6 (6.1 to 11.5)	**46.0 (35.51 to 56.46)**	10.5 (7.9 to 13.6)	12.5 (9.0 to 16.8)	**19.0 (10.85 to 27.34)**	4.2 (3.2 to 5.5)	6.7 (4.9 to 9.0)	**59.1 (47.92 to 70.95)**	16.2 (12.1 to 21.1)	20.5 (14.9 to 27.4)	**26.1 (17.40 to 35.39)**	1.6 (1.2 to 2.1)	1.8 (1.3 to 2.5)	**11.8 (1.92 to 21.74)**	5.1 (3.8 to 6.8)	4.9 (3.5 to 6.7)	-4.0 (-11.50 to 3.86)
**Unintentional injuries**	465.0 (357.0 to 595.0)	397.1 (286.2 to 533.2)	**-14.8 (-23.99 to -5.43)**	742.8 (570.4 to 953.6)	520.5 (375.0 to 699.8)	**-30.1 (-36.99 to -22.90)**	218.6 (166.8 to 281.9)	211.1 (152.7 to 283.1)	-3.6 (-11.59 to 4.33)	844.6 (644.9 to 1,088.7)	627.2 (454.6 to 842.1)	**-25.9 (-31.87 to -19.75)**	246.4 (188.7 to 312.6)	186.1 (133.7 to 250.1)	**-24.6 (-34.56 to -13.85)**	613.7 (472.1 to 784.3)	411.0 (294.8 to 555.7)	**-33.1 (-41.20 to -24.90)**
Falls	378.0 (289.4 to 481.8)	324.5 (234.1 to 433.4)	**-14.3 (-24.46 to -3.48)**	583.8 (448.0 to 746.0)	410.2 (295.6 to 550.0)	**-29.9 (-37.76 to -21.53)**	153.9 (118.0 to 198.2)	159.4 (115.8 to 213.5)	3.4 (-6.51 to 13.78)	589.9 (452.3 to 759.7)	462.5 (335.8 to 621.4)	**-21.7 (-29.32 to -13.83)**	224.1 (171.8 to 283.5)	165.1 (118.3 to 220.6)	**-26.4 (-36.71 to -15.03)**	543.2 (415.6 to 692.2)	352.8 (252.1 to 475.5)	**-35.1 (-43.43 to -26.22)**
Drowning	0.5 (0.4 to 0.6)	0.4 (0.3 to 0.5)	**-22.2 (-26.19 to -18.02)**	0.9 (0.7 to 1.1)	0.6 (0.4 to 0.7)	**-36.7 (-40.09 to -33.34)**	0.4 (0.3 to 0.5)	0.3 (0.2 to 0.4)	**-20.9 (-25.68 to -15.81)**	1.5 (1.1 to 1.8)	0.9 (0.7 to 1.2)	**-36.7 (-40.64 to -32.58)**	0.1 (0.1 to 0.1)	0.1 (0.1 to 0.1)	**-26.7 (-30.27 to -23.46)**	0.3 (0.2 to 0.4)	0.2 (0.1 to 0.3)	**-39.1 (-42.09 to -36.31)**
Fire, heat, and hot substances	9.2 (5.9 to 14.3)	7.0 (4.1 to 11.4)	**-24.2 (-30.62 to -18.24)**	16.5 (10.6 to 25.4)	10.3 (6.1 to 16.8)	**-37.6 (-43.27 to -32.30)**	5.1 (3.3 to 7.9)	4.1 (2.4 to 6.8)	**-19.2 (-27.01 to -11.61)**	20.1 (13.0 to 31.1)	13.0 (7.7 to 21.2)	**-35.4 (-41.76 to -29.24)**	4.1 (2.6 to 6.3)	2.9 (1.7 to 4.7)	**-30.4 (-36.65 to -24.40)**	13.2 (8.5 to 19.9)	7.8 (4.6 to 12.7)	**-41.5 (-47.31 to -35.71)**
Poisonings	0.6 (0.4 to 0.8)	0.3 (0.2 to 0.4)	**-41.3 (-45.68 to -36.98)**	1.1 (0.8 to 1.5)	0.5 (0.4 to 0.7)	**-51.1 (-54.95 to -47.64)**	0.4 (0.3 to 0.5)	0.2 (0.1 to 0.3)	**-41.5 (-46.86 to -35.82)**	1.4 (1.0 to 1.9)	0.7 (0.5 to 0.9)	**-52.1 (-56.56 to -47.44)**	0.2 (0.2 to 0.3)	0.1 (0.1 to 0.2)	**-40.8 (-47.43 to -33.86)**	0.8 (0.6 to 1.1)	0.4 (0.3 to 0.5)	**-49.7 (-55.20 to -43.57)**
Exposure to mechanical forces	44.4 (33.1 to 59.0)	39.6 (28.2 to 53.8)	**-11.1 (-17.53 to -4.38)**	86.7 (64.9 to 114.0)	64.9 (46.6 to 87.7)	**-25.3 (-30.76 to -19.60)**	38.1 (28.3 to 50.5)	31.2 (22.3 to 42.4)	**-18.1 (-24.21 to -12.23)**	152.7 (113.9 to 201.2)	104.3 (74.9 to 141.4)	**-31.8 (-36.78 to -26.84)**	6.4 (4.8 to 8.4)	8.4 (5.9 to 11.5)	**31.0 (19.51 to 43.13)**	24.0 (18.1 to 31.3)	26.7 (19.2 to 36.3)	**11.0 (0.96 to 21.51)**
Unintentional firearm injuries	0.5 (0.4 to 0.6)	0.3 (0.2 to 0.4)	**-35.5 (-41.50 to -28.92)**	0.9 (0.7 to 1.2)	0.5 (0.4 to 0.7)	**-44.7 (-49.98 to -39.02)**	0.4 (0.3 to 0.5)	0.2 (0.1 to 0.3)	**-47.1 (-52.38 to -41.37)**	1.4 (1.1 to 1.8)	0.7 (0.5 to 0.9)	**-54.9 (-59.41 to -49.98)**	0.1 (0.1 to 0.1)	0.1 (0.1 to 0.1)	3.6 (-6.50 to 14.96)	0.4 (0.3 to 0.5)	0.4 (0.3 to 0.5)	**-10.9 (-19.58 to -0.65)**
Unintentional suffocation	0.4 (0.3 to 0.6)	0.8 (0.6 to 1.1)	**100.3 (81.85 to 118.00)**	0.8 (0.6 to 1.1)	1.3 (1.0 to 1.8)	**63.7 (48.44 to 78.44)**	0.2 (0.2 to 0.3)	0.5 (0.4 to 0.6)	**92.9 (73.45 to 111.41)**	1.0 (0.8 to 1.3)	1.6 (1.2 to 2.1)	**56.7 (41.14 to 71.48)**	0.2 (0.1 to 0.2)	0.4 (0.3 to 0.5)	**111.5 (90.89 to 130.67)**	0.6 (0.5 to 0.8)	1.1 (0.8 to 1.5)	**75.1 (57.30 to 91.67)**
Other exposure to mechanical forces	43.6 (32.5 to 57.8)	38.5 (27.4 to 52.3)	**-11.9 (-18.32 to -5.24)**	85.0 (63.6 to 111.8)	63.0 (45.2 to 85.3)	**-25.9 (-31.33 to -20.25)**	37.5 (27.9 to 49.7)	30.6 (21.8 to 41.5)	**-18.6 (-24.65 to -12.70)**	150.3 (112.1 to 198.0)	102.0 (73.2 to 138.5)	**-32.2 (-37.16 to -27.20)**	6.1 (4.6 to 8.0)	7.9 (5.6 to 10.8)	**29.3 (17.79 to 41.62)**	23.0 (17.3 to 30.1)	25.3 (18.2 to 34.4)	**9.6 (-0.58 to 20.22)**
Adverse effects of medical treatment	2.7 (1.7 to 4.0)	3.3 (2.1 to 4.8)	**19.7 (15.38 to 24.15)**	4.7 (3.0 to 7.0)	4.7 (3.0 to 7.0)	-0.1 (-3.45 to 3.42)	1.3 (0.8 to 1.8)	1.6 (1.0 to 2.3)	**26.1 (20.33 to 32.65)**	4.8 (3.0 to 7.0)	4.8 (3.0 to 7.1)	-0.0 (-4.36 to 4.70)	1.5 (0.9 to 2.2)	1.7 (1.1 to 2.5)	**14.3 (9.04 to 19.70)**	4.7 (3.0 to 7.0)	4.7 (3.0 to 6.9)	-0.5 (-4.79 to 3.72)
Animal contact	1.9 (1.4 to 2.5)	2.4 (1.7 to 3.4)	**30.5 (22.39 to 38.60)**	3.5 (2.6 to 4.7)	3.8 (2.7 to 5.3)	**8.0 (1.15 to 14.66)**	1.0 (0.7 to 1.3)	1.3 (0.9 to 1.8)	**38.1 (28.70 to 47.80)**	3.9 (2.9 to 5.2)	4.3 (3.0 to 5.9)	**11.0 (3.49 to 18.84)**	0.9 (0.6 to 1.2)	1.1 (0.8 to 1.6)	**22.4 (14.41 to 30.70)**	3.2 (2.3 to 4.4)	3.4 (2.4 to 4.8)	4.6 (-2.49 to 11.72)
Venomous animal contact	0.3 (0.2 to 0.4)	0.3 (0.3 to 0.5)	**24.2 (15.77 to 32.71)**	0.5 (0.4 to 0.7)	0.6 (0.4 to 0.8)	**6.5 (-0.93 to 13.94)**	0.1 (0.1 to 0.2)	0.2 (0.1 to 0.2)	**29.5 (18.39 to 40.65)**	0.6 (0.4 to 0.8)	0.6 (0.5 to 0.8)	**8.4 (-1.10 to 18.26)**	0.1 (0.1 to 0.2)	0.2 (0.1 to 0.2)	**18.8 (7.13 to 30.46)**	0.5 (0.4 to 0.7)	0.5 (0.4 to 0.7)	4.3 (-6.05 to 14.82)
Non-venomous animal contact	1.6 (1.1 to 2.1)	2.1 (1.4 to 2.9)	**31.6 (22.91 to 39.99)**	3.0 (2.2 to 4.1)	3.2 (2.3 to 4.6)	**8.3 (1.00 to 15.37)**	0.8 (0.6 to 1.1)	1.1 (0.8 to 1.6)	**39.6 (29.39 to 50.05)**	3.3 (2.4 to 4.4)	3.7 (2.6 to 5.2)	**11.4 (3.11 to 20.01)**	0.8 (0.5 to 1.0)	0.9 (0.6 to 1.3)	**23.0 (14.66 to 32.28)**	2.7 (1.9 to 3.7)	2.8 (2.0 to 4.1)	4.7 (-2.90 to 12.78)
Foreign body	2.7 (1.9 to 3.7)	2.6 (1.7 to 3.7)	-4.6 (-12.36 to 3.35)	5.3 (3.7 to 7.3)	4.3 (2.7 to 6.3)	**-18.6 (-26.58 to -11.08)**	1.8 (1.2 to 2.5)	1.8 (1.2 to 2.5)	-0.4 (-7.93 to 7.52)	7.2 (4.9 to 10.0)	6.0 (3.8 to 8.7)	**-16.4 (-24.07 to -9.01)**	0.9 (0.7 to 1.3)	0.8 (0.5 to 1.2)	**-12.3 (-21.27 to -3.30)**	3.5 (2.5 to 4.8)	2.6 (1.7 to 3.9)	**-24.1 (-33.22 to -15.41)**
Pulmonary aspiration and foreign body in airway	0.5 (0.4 to 0.6)	0.4 (0.3 to 0.5)	**-7.0 (-14.44 to 1.42)**	0.9 (0.7 to 1.1)	0.7 (0.5 to 0.8)	**-24.5 (-31.03 to -17.53)**	0.3 (0.2 to 0.4)	0.3 (0.2 to 0.4)	-4.6 (-12.98 to 4.11)	1.3 (1.0 to 1.6)	1.0 (0.8 to 1.2)	**-23.4 (-30.65 to -15.92)**	0.1 (0.1 to 0.2)	0.1 (0.1 to 0.2)	**-12.4 (-20.91 to -2.73)**	0.5 (0.4 to 0.6)	0.3 (0.3 to 0.4)	**-29.1 (-36.16 to -21.38)**
Foreign body in eyes	0.4 (0.2 to 0.6)	0.4 (0.2 to 0.6)	-1.8 (-9.89 to 4.75)	0.8 (0.5 to 1.2)	0.7 (0.4 to 1.1)	**-12.9 (-20.87 to -6.49)**	0.2 (0.1 to 0.4)	0.2 (0.1 to 0.4)	1.8 (-5.27 to 8.16)	1.0 (0.6 to 1.5)	0.9 (0.5 to 1.4)	**-11.1 (-18.78 to -4.87)**	0.1 (0.1 to 0.2)	0.1 (0.1 to 0.2)	**-7.8 (-17.91 to 1.10)**	0.6 (0.3 to 0.9)	0.5 (0.3 to 0.8)	**-16.6 (-26.44 to -7.96)**
Foreign body in other body part	1.9 (1.3 to 2.6)	1.8 (1.1 to 2.6)	-4.6 (-14.63 to 4.75)	3.6 (2.4 to 5.2)	3.0 (1.8 to 4.5)	**-18.4 (-27.85 to -9.62)**	1.2 (0.8 to 1.7)	1.2 (0.7 to 1.8)	0.2 (-9.58 to 9.95)	4.9 (3.2 to 7.0)	4.1 (2.5 to 6.3)	**-15.6 (-24.85 to -6.71)**	0.7 (0.5 to 0.9)	0.6 (0.4 to 0.8)	**-13.4 (-24.14 to -2.43)**	2.4 (1.7 to 3.4)	1.8 (1.1 to 2.7)	**-25.0 (-35.34 to -14.72)**
Other unintentional injuries	25.1 (17.9 to 34.0)	17.0 (12.0 to 23.5)	**-32.2 (-36.48 to -28.11)**	40.4 (28.9 to 54.7)	21.2 (14.9 to 29.4)	**-47.6 (-51.03 to -44.39)**	16.9 (12.0 to 22.9)	11.1 (7.8 to 15.4)	**-34.3 (-38.91 to -28.81)**	63.2 (45.1 to 86.0)	30.7 (21.6 to 42.6)	**-51.5 (-54.84 to -47.43)**	8.2 (5.9 to 11.1)	5.9 (4.2 to 8.2)	**-28.0 (-33.49 to -21.68)**	20.7 (15.0 to 28.0)	12.4 (8.8 to 17.1)	**-40.3 (-44.75 to -35.41)**
**Self-harm and interpersonal violence**	5.9 (4.4 to 7.6)	4.1 (2.9 to 5.5)	**-30.9 (-35.81 to -25.60)**	10.9 (8.2 to 14.0)	6.1 (4.4 to 8.2)	**-43.5 (-47.65 to -39.09)**	4.3 (3.2 to 5.5)	3.1 (2.2 to 4.2)	**-27.1 (-31.76 to -21.98)**	16.5 (12.3 to 21.3)	9.6 (6.9 to 12.9)	**-41.8 (-45.46 to -37.78)**	1.6 (1.2 to 2.1)	1.0 (0.7 to 1.3)	**-40.9 (-46.14 to -34.92)**	5.7 (4.3 to 7.4)	2.9 (2.1 to 3.8)	**-50.1 (-54.62 to -44.77)**
Self-harm	2.3 (1.7 to 3.0)	1.9 (1.3 to 2.5)	**-18.6 (-22.69 to -14.24)**	4.1 (3.0 to 5.4)	2.7 (1.9 to 3.6)	**-33.7 (-37.03 to -30.14)**	1.8 (1.3 to 2.4)	1.6 (1.1 to 2.1)	**-13.9 (-18.41 to -8.96)**	6.8 (5.0 to 9.2)	4.7 (3.3 to 6.3)	**-32.1 (-35.72 to -28.19)**	0.5 (0.4 to 0.6)	0.3 (0.2 to 0.4)	**-35.9 (-41.65 to -30.50)**	1.6 (1.2 to 2.1)	0.9 (0.6 to 1.2)	**-45.3 (-50.12 to -40.46)**
Interpersonal violence	3.6 (2.7 to 4.6)	2.2 (1.6 to 3.0)	**-38.7 (-44.48 to -32.31)**	6.8 (5.2 to 8.7)	3.4 (2.5 to 4.6)	**-49.4 (-54.30 to -43.99)**	2.5 (1.9 to 3.2)	1.6 (1.1 to 2.1)	**-36.7 (-42.14 to -30.67)**	9.6 (7.3 to 12.4)	5.0 (3.6 to 6.6)	**-48.6 (-53.05 to -43.74)**	1.2 (0.9 to 1.5)	0.7 (0.5 to 0.9)	**-42.9 (-49.57 to -35.48)**	4.1 (3.2 to 5.3)	2.0 (1.4 to 2.7)	**-51.9 (-57.68 to -45.30)**
Assault by firearm	0.4 (0.3 to 0.5)	0.2 (0.2 to 0.3)	**-43.0 (-47.02 to -38.54)**	0.7 (0.5 to 0.9)	0.3 (0.2 to 0.4)	**-53.2 (-56.55 to -49.53)**	0.3 (0.2 to 0.3)	0.2 (0.1 to 0.2)	**-42.8 (-47.19 to -38.46)**	1.0 (0.8 to 1.3)	0.5 (0.4 to 0.6)	**-54.1 (-57.63 to -50.66)**	0.1 (0.1 to 0.1)	0.1 (0.0 to 0.1)	**-43.4 (-47.58 to -39.07)**	0.4 (0.3 to 0.5)	0.2 (0.1 to 0.2)	**-51.8 (-55.48 to -47.93)**
Assault by sharp object	0.3 (0.2 to 0.4)	0.2 (0.2 to 0.3)	**-25.6 (-30.39 to -20.68)**	0.6 (0.4 to 0.8)	0.4 (0.3 to 0.5)	**-38.2 (-42.21 to -34.09)**	0.2 (0.2 to 0.3)	0.2 (0.1 to 0.2)	**-24.9 (-29.94 to -19.56)**	0.8 (0.6 to 1.1)	0.5 (0.4 to 0.7)	**-39.0 (-43.07 to -34.57)**	0.1 (0.1 to 0.1)	0.1 (0.1 to 0.1)	**-26.9 (-31.82 to -21.91)**	0.3 (0.3 to 0.5)	0.2 (0.2 to 0.3)	**-37.8 (-42.27 to -33.33)**
Assault by other means	2.9 (2.2 to 3.8)	1.8 (1.3 to 2.4)	**-39.5 (-45.68 to -32.76)**	5.5 (4.2 to 7.0)	2.8 (2.0 to 3.7)	**-50.1 (-55.26 to -44.32)**	2.0 (1.5 to 2.5)	1.2 (0.9 to 1.7)	**-37.1 (-42.86 to -30.84)**	7.8 (5.9 to 10.0)	4.0 (2.9 to 5.3)	**-48.9 (-53.64 to -43.65)**	1.0 (0.7 to 1.2)	0.5 (0.4 to 0.7)	**-44.5 (-51.53 to -36.37)**	3.4 (2.6 to 4.3)	1.6 (1.1 to 2.1)	**-53.3 (-59.40 to -46.13)**
**Forces of nature, war, and legal intervention**	0.1 (0.1 to 0.2)	0.1 (0.0 to 0.1)	**-40.2 (-60.33 to -17.37)**	0.3 (0.1 to 0.5)	0.1 (0.1 to 0.2)	**-50.6 (-67.36 to -31.45)**	0.1 (0.0 to 0.1)	<0.05 (0.0 to 0.1)	**-42.4 (-62.40 to -19.87)**	0.3 (0.2 to 0.7)	0.2 (0.1 to 0.2)	**-52.1 (-68.99 to -34.07)**	<0.05 (0.0 to 0.1)	<0.05 (0.0 to <0.05)	**-35.5 (-58.07 to -6.87)**	0.2 (0.1 to 0.3)	0.1 (0.0 to 0.1)	**-46.7 (-65.48 to -22.85)**
Exposure to forces of nature	<0.05 (0.0 to <0.05)	<0.05 (0.0 to <0.05)	**-82.1 (-92.88 to -60.59)**	<0.05 (0.0 to 0.1)	<0.05 (0.0 to <0.05)	**-85.1 (-94.11 to -66.76)**	<0.05 (0.0 to <0.05)	<0.05 (0.0 to <0.05)	**-82.6 (-93.20 to -63.17)**	0.1 (0.0 to 0.1)	<0.05 (0.0 to <0.05)	**-85.5 (-94.35 to -69.23)**	<0.05 (0.0 to <0.05)	<0.05 (0.0 to <0.05)	**-55.9 (-92.30 to -47.52)**	<0.05 (0.0 to <0.05)	<0.05 (0.0 to <0.05)	**-51.5 (-93.62 to -54.58)**
Collective violence and legal intervention	0.1 (0.0 to 0.2)	0.1 (0.0 to 0.1)	**-31.9 (-56.37 to -3.08)**	0.2 (0.1 to 0.4)	0.1 (0.1 to 0.2)	**-42.8 (-63.45 to -18.25)**	0.1 (0.0 to 0.1)	<0.05 (0.0 to 0.1)	**-34.4 (-57.91 to -6.45)**	0.3 (0.1 to 0.6)	0.1 (0.1 to 0.2)	**-44.5 (-64.44 to -20.04)**	<0.05 (0.0 to 0.1)	<0.05 (0.0 to <0.05)	-26.5 (-54.06 to 6.25)	0.1 (0.1 to 0.3)	0.1 (0.0 to 0.1)	**-38.2 (-61.81 to -9.22)**

Web Table 7 : DALYs in England in 1990 and 2013 for all ages and age-standardized rates (per 100,000) by sex with 95% UI and percentage change. Percent changes in bold denote a statistically significant change.																		
	Both sexes combined						Males						Females					
Cause	DALYs (in thousands)			Age-standardized rate (per 100,000)			DALYs (in thousands)			Age-standardized rate (per 100,000)			DALYs (in thousands)			Age-standardized rate (per 100,000)		
	1990	2013	% change	1990	2013	% change	1990	2013	% change	1990	2013	% change	1990	2013	% change	1990	2013	% change
**All causes**	15,099.8 (13,546.5 to 16,839.5)	13,683.7 (11,869.3 to 15,675.8)	**-9.5 (-12.92 to -6.23)**	26,089.4 (23,211.1 to 29,349.4)	19,873.1 (17,015.0 to 23,060.8)	**-23.9 (-27.09 to -20.90)**	7,782.4 (7,078.2 to 8,588.9)	6,911.9 (6,053.3 to 7,860.9)	**-11.3 (-15.27 to -7.59)**	29,886.2 (27,049.3 to 33,154.3)	21,199.4 (18,401.6 to 24,253.8)	**-29.1 (-32.62 to -25.90)**	7,317.4 (6,463.5 to 8,273.8)	6,771.9 (5,803.1 to 7,846.8)	**-7.5 (-11.37 to -3.94)**	23,031.9 (20,048.5 to 26,369.0)	18,682.1 (15,715.2 to 21,997.2)	**-19.0 (-22.51 to -15.80)**
**Communicable, maternal, neonatal, and nutritional diseases**	933.1 (841.0 to 1,054.7)	785.9 (692.9 to 905.1)	**-15.8 (-23.71 to -9.48)**	1,985.8 (1,778.2 to 2,243.2)	1,430.8 (1,227.2 to 1,687.2)	**-28.0 (-35.32 to -21.16)**	473.3 (427.0 to 544.3)	396.6 (345.3 to 456.6)	**-16.2 (-25.87 to -9.03)**	2,211.7 (1,990.4 to 2,518.6)	1,540.0 (1,316.3 to 1,813.0)	**-30.4 (-38.37 to -23.07)**	459.8 (413.3 to 526.0)	389.3 (338.0 to 453.0)	**-15.3 (-23.37 to -8.78)**	1,779.2 (1,576.1 to 2,041.4)	1,321.0 (1,109.6 to 1,581.5)	**-25.8 (-33.66 to -18.93)**
**HIV/AIDS and tuberculosis**	30.9 (27.5 to 34.0)	20.5 (17.1 to 24.6)	**-33.5 (-44.91 to -20.41)**	58.5 (51.9 to 64.5)	33.3 (27.7 to 40.1)	**-43.0 (-52.90 to -31.37)**	21.5 (18.8 to 24.0)	13.6 (10.9 to 16.6)	**-36.9 (-49.44 to -22.45)**	84.2 (73.7 to 94.0)	44.3 (35.9 to 54.9)	**-47.2 (-57.66 to -35.29)**	9.4 (8.3 to 10.5)	7.0 (5.6 to 8.5)	**-25.7 (-39.20 to -9.62)**	34.7 (30.5 to 38.9)	22.7 (18.2 to 28.1)	**-34.5 (-46.22 to -19.40)**
Tuberculosis	15.0 (13.8 to 17.0)	9.9 (8.4 to 11.3)	**-33.9 (-44.79 to -27.02)**	26.5 (24.2 to 30.1)	15.2 (12.8 to 17.6)	**-42.8 (-51.95 to -36.85)**	9.0 (8.2 to 10.4)	5.9 (4.8 to 6.8)	**-34.5 (-46.16 to -26.21)**	34.0 (30.9 to 39.0)	18.3 (15.0 to 21.3)	**-46.2 (-55.35 to -39.66)**	5.9 (5.4 to 6.9)	4.0 (3.3 to 4.6)	**-32.9 (-45.19 to -24.99)**	20.5 (18.5 to 23.7)	12.4 (10.2 to 14.6)	**-39.6 (-50.25 to -32.49)**
HIV/AIDS	15.9 (12.8 to 18.3)	10.6 (7.6 to 14.2)	**-32.9 (-53.50 to -9.16)**	32.0 (25.9 to 36.8)	18.1 (13.0 to 24.0)	**-43.1 (-59.84 to -22.90)**	12.5 (9.9 to 14.6)	7.6 (5.4 to 10.6)	**-38.4 (-58.25 to -14.14)**	50.2 (40.1 to 58.6)	26.0 (18.4 to 35.7)	**-47.8 (-64.31 to -27.78)**	3.5 (2.6 to 4.3)	3.0 (2.0 to 4.4)	-12.9 (-41.13 to 27.77)	14.2 (10.7 to 17.5)	10.3 (6.8 to 15.0)	-26.9 (-50.47 to 6.72)
HIV/AIDS resulting in mycobacterial infection	1.2 (0.9 to 1.6)	0.5 (0.3 to 0.7)	**-58.6 (-71.84 to -42.96)**	2.5 (1.9 to 3.1)	0.9 (0.6 to 1.2)	**-64.6 (-75.76 to -51.68)**	1.0 (0.8 to 1.3)	0.4 (0.3 to 0.6)	**-61.3 (-73.98 to -45.45)**	4.1 (3.1 to 5.2)	1.3 (0.9 to 1.9)	**-67.0 (-77.57 to -53.77)**	0.2 (0.2 to 0.3)	0.1 (0.1 to 0.2)	**-45.7 (-63.71 to -19.22)**	0.9 (0.6 to 1.2)	0.4 (0.3 to 0.6)	**-54.0 (-68.95 to -31.79)**
HIV/AIDS resulting in other diseases	14.7 (11.8 to 17.0)	10.1 (7.2 to 13.6)	**-30.7 (-52.06 to -6.12)**	29.5 (23.8 to 34.0)	17.2 (12.4 to 22.8)	**-41.3 (-58.44 to -20.63)**	11.5 (9.1 to 13.5)	7.2 (5.1 to 10.0)	**-36.3 (-56.94 to -11.36)**	46.1 (36.8 to 53.8)	24.7 (17.4 to 33.8)	**-46.1 (-63.09 to -25.59)**	3.2 (2.4 to 4.0)	2.9 (1.9 to 4.2)	-10.7 (-39.59 to 30.27)	13.3 (10.0 to 16.4)	9.9 (6.6 to 14.5)	-25.1 (-49.19 to 9.19)
**Diarrhea, lower respiratory, and other common infectious diseases**	432.2 (404.5 to 523.2)	371.3 (339.5 to 424.0)	**-13.9 (-25.19 to -7.02)**	708.8 (661.0 to 842.8)	468.9 (427.0 to 521.8)	**-33.7 (-42.71 to -27.74)**	201.5 (185.9 to 259.0)	176.7 (155.9 to 199.8)	**-11.8 (-27.61 to -2.57)**	815.8 (752.0 to 1,027.1)	511.7 (453.7 to 574.3)	**-37.0 (-47.99 to -30.13)**	230.7 (215.8 to 285.9)	194.6 (171.9 to 236.5)	**-15.5 (-26.06 to -5.54)**	625.7 (582.2 to 760.1)	428.4 (378.1 to 493.6)	**-31.4 (-39.72 to -24.54)**
Diarrheal diseases	7.2 (6.2 to 8.3)	22.4 (20.3 to 24.9)	**214.7 (170.14 to 266.06)**	15.5 (13.4 to 18.1)	33.6 (30.3 to 37.5)	**117.5 (85.20 to 154.40)**	3.9 (3.3 to 4.6)	10.0 (8.8 to 11.3)	**158.2 (117.89 to 203.93)**	18.1 (15.3 to 21.6)	33.7 (29.5 to 38.3)	**87.5 (55.55 to 119.80)**	3.3 (2.9 to 3.7)	12.5 (11.0 to 14.1)	**281.9 (222.84 to 355.37)**	13.0 (11.3 to 15.2)	33.1 (29.2 to 37.7)	**155.5 (111.27 to 205.51)**
Intestinal infectious diseases	3.3 (1.4 to 7.0)	3.7 (1.3 to 7.9)	9.4 (-16.30 to 37.74)	8.3 (3.5 to 17.6)	8.7 (3.1 to 18.8)	4.3 (-21.80 to 33.44)	1.8 (0.7 to 3.8)	2.1 (0.8 to 4.6)	20.7 (-17.85 to 65.65)	8.6 (3.4 to 18.6)	9.9 (3.5 to 21.4)	14.6 (-23.15 to 59.91)	1.6 (0.6 to 3.3)	1.6 (0.6 to 3.4)	-1.4 (-30.87 to 26.29)	8.0 (3.3 to 16.9)	7.6 (2.9 to 16.9)	-5.6 (-35.87 to 22.68)
Typhoid fever	2.4 (0.8 to 5.5)	2.7 (0.9 to 6.1)	12.7 (-7.54 to 34.93)	6.0 (2.1 to 13.8)	6.4 (2.1 to 14.5)	6.9 (-13.10 to 29.52)	1.3 (0.4 to 2.9)	1.5 (0.5 to 3.4)	23.4 (-8.36 to 59.50)	6.2 (2.1 to 14.2)	7.1 (2.3 to 16.0)	17.0 (-13.61 to 54.59)	1.2 (0.4 to 2.6)	1.2 (0.4 to 2.7)	2.1 (-17.08 to 24.68)	5.9 (2.0 to 13.2)	5.7 (1.9 to 13.2)	-3.1 (-21.25 to 19.64)
Paratyphoid fever	0.4 (0.1 to 1.0)	0.7 (0.2 to 1.6)	**67.1 (33.46 to 104.34)**	1.1 (0.4 to 2.5)	1.8 (0.6 to 4.0)	**61.0 (27.93 to 100.11)**	0.2 (0.1 to 0.5)	0.4 (0.1 to 1.0)	**105.0 (49.74 to 164.73)**	1.1 (0.4 to 2.5)	2.1 (0.7 to 4.8)	**96.4 (42.14 to 157.94)**	0.2 (0.1 to 0.5)	0.3 (0.1 to 0.7)	**30.7 (3.40 to 63.10)**	1.1 (0.4 to 2.5)	1.4 (0.5 to 3.3)	**27.0 (-0.25 to 59.67)**
Other intestinal infectious diseases	0.5 (0.2 to 0.9)	0.2 (0.1 to 0.5)	-43.1 (-89.25 to 74.93)	1.2 (0.4 to 2.4)	0.5 (0.1 to 1.3)	-45.9 (-91.25 to 83.84)	0.3 (0.1 to 0.7)	0.1 (0.0 to 0.4)	-31.5 (-94.22 to 186.15)	1.4 (0.3 to 3.4)	0.6 (0.1 to 2.0)	-34.4 (-95.41 to 181.78)	0.2 (0.0 to 0.5)	0.1 (0.0 to 0.3)	-28.5 (-95.62 to 241.16)	1.0 (0.1 to 2.9)	0.4 (0.0 to 1.7)	-28.6 (-96.82 to 266.50)
Lower respiratory infections	367.9 (346.1 to 458.8)	304.1 (276.3 to 354.9)	**-17.1 (-28.87 to -9.72)**	561.3 (527.9 to 691.2)	344.4 (314.3 to 388.2)	**-38.4 (-47.91 to -32.75)**	167.1 (154.1 to 223.5)	143.3 (125.0 to 163.9)	**-13.7 (-31.11 to -3.18)**	655.2 (605.7 to 860.8)	381.4 (333.6 to 429.4)	**-41.4 (-53.33 to -34.33)**	200.8 (187.6 to 255.8)	160.8 (140.7 to 202.9)	**-19.8 (-30.79 to -9.16)**	491.3 (460.0 to 615.3)	310.1 (274.8 to 372.8)	**-36.7 (-45.64 to -29.06)**
Upper respiratory infections	8.7 (5.0 to 14.1)	9.5 (5.4 to 15.6)	**9.4 (6.82 to 12.07)**	18.9 (10.8 to 30.8)	18.8 (10.6 to 31.1)	-0.4 (-2.92 to 1.93)	4.2 (2.4 to 6.8)	4.6 (2.6 to 7.7)	**11.1 (7.02 to 14.82)**	18.5 (10.5 to 30.1)	18.5 (10.5 to 30.8)	0.0 (-3.64 to 3.25)	4.5 (2.6 to 7.3)	4.8 (2.7 to 7.9)	**7.9 (4.10 to 11.73)**	19.3 (11.0 to 31.6)	19.1 (10.8 to 31.4)	-0.8 (-4.30 to 2.53)
Otitis media	12.3 (7.7 to 19.0)	11.2 (6.8 to 17.6)	**-8.9 (-15.21 to -3.02)**	27.0 (17.0 to 41.8)	21.7 (13.1 to 33.9)	**-20.0 (-25.80 to -14.70)**	6.5 (4.1 to 10.2)	5.8 (3.5 to 9.2)	**-11.5 (-20.21 to -2.82)**	28.8 (18.3 to 44.7)	22.4 (13.5 to 35.5)	**-22.6 (-30.04 to -15.34)**	5.8 (3.6 to 8.9)	5.4 (3.3 to 8.5)	-5.9 (-12.88 to 1.82)	25.2 (15.7 to 39.1)	20.9 (12.7 to 32.8)	**-17.2 (-23.51 to -10.44)**
Meningitis	24.6 (21.8 to 28.2)	12.3 (10.8 to 14.5)	**-49.8 (-55.80 to -42.49)**	59.5 (52.0 to 69.2)	26.3 (22.5 to 32.0)	**-55.7 (-61.57 to -48.80)**	13.9 (11.1 to 17.2)	6.9 (5.8 to 8.7)	**-50.0 (-57.49 to -40.88)**	67.5 (53.1 to 85.9)	30.0 (24.5 to 39.3)	**-55.4 (-62.52 to -46.38)**	10.8 (9.3 to 11.7)	5.4 (4.7 to 6.6)	**-49.4 (-56.22 to -35.52)**	51.3 (44.9 to 56.3)	22.5 (19.3 to 27.0)	**-56.0 (-62.72 to -46.14)**
Pneumococcal meningitis	3.9 (3.5 to 4.5)	2.2 (1.9 to 2.6)	**-44.5 (-52.19 to -34.56)**	9.1 (7.9 to 10.5)	4.4 (3.7 to 5.2)	**-51.9 (-59.33 to -43.19)**	2.2 (1.8 to 2.7)	1.2 (1.0 to 1.4)	**-46.5 (-56.64 to -35.55)**	10.5 (8.3 to 12.8)	4.9 (4.0 to 6.2)	**-53.0 (-62.48 to -42.45)**	1.7 (1.5 to 2.0)	1.0 (0.8 to 1.2)	**-41.5 (-51.40 to -25.47)**	7.7 (6.6 to 9.1)	3.8 (3.2 to 4.5)	**-50.4 (-59.00 to -37.90)**
H influenzae type B meningitis	4.9 (4.3 to 6.2)	2.5 (2.0 to 3.0)	**-50.1 (-59.74 to -40.78)**	12.9 (10.9 to 16.4)	5.8 (4.7 to 7.3)	**-54.7 (-64.02 to -44.85)**	2.9 (2.1 to 4.1)	1.4 (1.1 to 1.9)	**-49.5 (-62.28 to -36.10)**	14.9 (10.8 to 21.4)	6.8 (5.1 to 9.4)	**-53.8 (-65.96 to -40.12)**	2.1 (1.7 to 2.4)	1.0 (0.8 to 1.2)	**-50.4 (-60.59 to -38.33)**	10.7 (8.7 to 12.7)	4.7 (3.8 to 5.9)	**-55.7 (-65.65 to -44.40)**
Meningococcal meningitis	7.4 (6.5 to 8.5)	3.6 (3.1 to 4.3)	**-52.0 (-58.69 to -43.01)**	18.0 (15.6 to 21.1)	7.6 (6.4 to 9.5)	**-57.7 (-64.18 to -49.32)**	4.2 (3.3 to 5.1)	2.0 (1.6 to 2.5)	**-52.3 (-61.28 to -41.06)**	20.4 (15.6 to 25.6)	8.6 (6.7 to 11.6)	**-57.6 (-66.74 to -46.06)**	3.3 (2.8 to 3.7)	1.6 (1.3 to 2.0)	**-51.4 (-60.71 to -36.28)**	15.6 (13.4 to 18.0)	6.6 (5.6 to 8.1)	**-57.5 (-66.32 to -46.02)**
Other meningitis	8.3 (7.3 to 9.3)	4.2 (3.6 to 4.9)	**-50.0 (-56.10 to -40.12)**	19.5 (17.0 to 22.3)	8.6 (7.4 to 10.3)	**-56.1 (-61.91 to -47.89)**	4.6 (3.9 to 5.6)	2.3 (1.9 to 2.8)	**-49.6 (-57.47 to -40.55)**	21.8 (17.7 to 27.2)	9.7 (8.0 to 12.4)	**-55.2 (-62.73 to -46.43)**	3.7 (3.1 to 4.2)	1.8 (1.6 to 2.2)	**-50.3 (-58.49 to -36.15)**	17.3 (14.7 to 19.7)	7.4 (6.2 to 9.1)	**-57.2 (-65.21 to -45.33)**
Encephalitis	3.7 (3.2 to 4.9)	4.2 (2.8 to 5.1)	17.1 (-34.99 to 53.08)	8.3 (7.1 to 11.0)	8.3 (5.5 to 10.3)	3.0 (-42.29 to 37.48)	2.0 (1.7 to 2.9)	2.2 (1.4 to 2.8)	11.8 (-39.37 to 48.19)	9.4 (7.6 to 13.0)	8.9 (5.5 to 11.4)	-2.6 (-47.01 to 32.32)	1.6 (1.4 to 2.4)	2.0 (1.3 to 2.5)	24.1 (-31.58 to 64.83)	7.2 (6.1 to 10.4)	7.7 (5.0 to 9.6)	10.6 (-38.61 to 49.25)
Diphtheria	0.1 (0.1 to 0.2)	<0.05 (0.0 to 0.1)	**-61.1 (-83.75 to -20.23)**	0.3 (0.2 to 0.6)	0.1 (0.1 to 0.2)	**-64.7 (-86.52 to -24.11)**	0.1 (0.0 to 0.1)	<0.05 (0.0 to <0.05)	**-59.3 (-87.71 to -1.47)**	0.4 (0.2 to 0.8)	0.1 (0.1 to 0.2)	**-62.8 (-89.86 to -3.11)**	<0.05 (0.0 to 0.1)	<0.05 (0.0 to <0.05)	**-56.0 (-87.93 to 16.56)**	0.3 (0.1 to 0.6)	0.1 (0.0 to 0.2)	**-59.6 (-90.12 to 14.59)**
Whooping cough	0.7 (0.5 to 1.0)	0.7 (0.4 to 1.1)	-4.4 (-52.62 to 74.41)	1.9 (1.3 to 2.8)	1.7 (0.9 to 2.9)	-7.1 (-55.79 to 74.14)	0.4 (0.2 to 0.5)	0.3 (0.2 to 0.6)	0.6 (-55.48 to 100.35)	1.9 (1.3 to 2.9)	1.7 (0.9 to 3.1)	-1.9 (-57.88 to 101.22)	0.4 (0.3 to 0.6)	0.3 (0.2 to 0.6)	-8.6 (-54.79 to 67.92)	2.0 (1.4 to 3.1)	1.7 (1.0 to 3.1)	-11.5 (-58.37 to 67.54)
Tetanus	0.2 (0.2 to 0.3)	0.2 (0.1 to 0.3)	-6.1 (-52.43 to 11.48)	0.6 (0.5 to 0.7)	0.5 (0.2 to 0.6)	-13.6 (-57.62 to 5.47)	0.1 (0.1 to 0.2)	0.1 (0.1 to 0.1)	-5.7 (-56.60 to 16.05)	0.6 (0.5 to 0.8)	0.5 (0.2 to 0.6)	-14.2 (-60.99 to 6.99)	0.1 (0.1 to 0.2)	0.1 (0.1 to 0.1)	-6.2 (-51.69 to 11.67)	0.6 (0.5 to 0.7)	0.5 (0.2 to 0.6)	-12.7 (-56.83 to 6.79)
Measles	0.7 (0.4 to 1.3)	<0.05 (0.0 to 0.1)	**-95.9 (-98.06 to -92.51)**	2.1 (1.0 to 3.8)	0.1 (0.0 to 0.1)	**-96.0 (-98.11 to -92.69)**	0.4 (0.2 to 0.7)	<0.05 (0.0 to <0.05)	**-95.9 (-98.06 to -92.25)**	2.0 (1.0 to 3.8)	0.1 (0.0 to 0.1)	**-96.0 (-98.11 to -92.45)**	0.4 (0.2 to 0.7)	<0.05 (0.0 to <0.05)	**-95.9 (-98.08 to -92.65)**	2.1 (1.0 to 3.9)	0.1 (0.0 to 0.1)	**-96.0 (-98.12 to -92.83)**
Varicella and herpes zoster	2.7 (2.0 to 3.6)	2.9 (2.1 to 3.9)	7.3 (-9.14 to 23.08)	5.2 (3.9 to 6.9)	4.7 (3.5 to 6.4)	-9.3 (-25.09 to 6.62)	1.2 (0.9 to 1.6)	1.3 (0.9 to 1.8)	10.2 (-11.33 to 33.24)	5.0 (3.8 to 6.7)	4.5 (3.3 to 6.1)	-9.8 (-29.87 to 12.57)	1.5 (1.1 to 2.1)	1.6 (1.1 to 2.2)	5.5 (-16.14 to 28.20)	5.4 (3.9 to 7.2)	5.0 (3.5 to 6.8)	-8.2 (-27.93 to 13.83)
**Neglected tropical diseases and malaria**	1.6 (1.4 to 1.8)	1.9 (1.5 to 2.1)	**25.3 (-15.04 to 41.74)**	3.2 (2.9 to 3.7)	3.7 (2.7 to 4.1)	13.4 (-24.48 to 27.91)	0.8 (0.7 to 0.9)	1.1 (0.9 to 1.3)	**41.8 (-1.86 to 66.48)**	3.4 (3.1 to 4.0)	4.2 (3.1 to 4.8)	**23.3 (-19.98 to 44.20)**	0.8 (0.7 to 0.9)	0.8 (0.5 to 0.9)	8.2 (-42.63 to 22.96)	3.1 (2.8 to 3.7)	3.1 (1.9 to 3.6)	2.9 (-48.11 to 18.03)
Malaria	<0.05 (0.0 to <0.05)	<0.05 (0.0 to <0.05)	23.1 (-22.85 to 90.73)	<0.05 (0.0 to <0.05)	<0.05 (0.0 to <0.05)	12.3 (-30.25 to 74.48)	<0.05 (0.0 to <0.05)	<0.05 (0.0 to <0.05)	27.4 (-45.01 to 154.24)	<0.05 (0.0 to <0.05)	<0.05 (0.0 to <0.05)	16.5 (-49.73 to 131.75)	<0.05 (0.0 to <0.05)	<0.05 (0.0 to <0.05)	25.6 (-32.57 to 139.04)	<0.05 (0.0 to <0.05)	<0.05 (0.0 to <0.05)	14.4 (-38.78 to 119.97)
Cysticercosis	0.2 (0.1 to 0.2)	<0.05 (0.0 to 0.1)	**-72.9 (-79.56 to -66.21)**	0.3 (0.2 to 0.4)	0.1 (0.1 to 0.1)	**-77.2 (-82.83 to -71.77)**	0.1 (0.1 to 0.1)	<0.05 (0.0 to <0.05)	**-81.8 (-86.65 to -77.14)**	0.3 (0.3 to 0.5)	0.1 (0.0 to 0.1)	**-84.5 (-88.70 to -80.56)**	0.1 (0.1 to 0.1)	<0.05 (0.0 to <0.05)	**-62.4 (-71.87 to -48.84)**	0.3 (0.2 to 0.3)	0.1 (0.1 to 0.1)	**-68.2 (-76.51 to -56.57)**
Cystic echinococcosis	0.6 (0.5 to 0.7)	0.3 (0.3 to 0.4)	**-43.3 (-47.44 to -38.68)**	1.1 (0.9 to 1.3)	0.5 (0.4 to 0.6)	**-53.1 (-56.36 to -49.58)**	0.3 (0.2 to 0.4)	0.2 (0.1 to 0.2)	**-33.9 (-40.92 to -26.43)**	1.2 (0.9 to 1.4)	0.6 (0.5 to 0.8)	**-46.8 (-52.34 to -41.08)**	0.3 (0.2 to 0.4)	0.1 (0.1 to 0.2)	**-52.8 (-56.62 to -48.40)**	1.0 (0.8 to 1.2)	0.4 (0.3 to 0.5)	**-60.1 (-63.04 to -57.03)**
Dengue	0.3 (0.2 to 0.4)	0.2 (0.2 to 0.3)	-9.1 (-43.84 to 1.90)	0.6 (0.5 to 0.8)	0.5 (0.3 to 0.6)	**-16.3 (-49.17 to -4.76)**	0.1 (0.1 to 0.2)	0.1 (0.1 to 0.1)	-9.1 (-46.92 to 4.25)	0.6 (0.5 to 0.9)	0.5 (0.3 to 0.6)	**-16.5 (-52.42 to -3.13)**	0.1 (0.1 to 0.2)	0.1 (0.1 to 0.1)	-8.6 (-46.77 to 5.43)	0.6 (0.5 to 0.9)	0.5 (0.3 to 0.6)	**-15.6 (-51.06 to -2.08)**
Rabies	0.3 (0.2 to 0.4)	0.2 (0.1 to 0.3)	-17.2 (-54.01 to 1.18)	0.6 (0.5 to 0.8)	0.5 (0.3 to 0.5)	**-24.0 (-58.40 to -6.23)**	0.1 (0.1 to 0.2)	0.1 (0.1 to 0.1)	**-20.5 (-58.98 to 1.10)**	0.6 (0.4 to 0.9)	0.4 (0.2 to 0.6)	**-27.4 (-63.05 to -6.79)**	0.1 (0.1 to 0.2)	0.1 (0.1 to 0.1)	-13.2 (-52.47 to 4.12)	0.6 (0.4 to 0.9)	0.5 (0.2 to 0.6)	**-20.0 (-57.19 to -3.19)**
Other neglected tropical diseases	0.3 (0.3 to 0.4)	1.1 (0.7 to 1.3)	**301.7 (65.27 to 369.13)**	0.6 (0.6 to 1.0)	2.1 (1.3 to 2.4)	**234.0 (39.60 to 290.74)**	0.1 (0.1 to 0.2)	0.7 (0.5 to 0.8)	**371.0 (97.87 to 479.75)**	0.7 (0.6 to 0.9)	2.6 (1.8 to 3.1)	**284.2 (50.59 to 368.94)**	0.1 (0.1 to 0.2)	0.4 (0.1 to 0.5)	**226.7 (-44.19 to 289.39)**	0.6 (0.6 to 1.1)	1.7 (0.5 to 2.0)	**182.1 (-51.01 to 239.26)**
**Maternal disorders**	7.8 (6.4 to 9.6)	6.2 (4.7 to 8.0)	**-20.9 (-29.25 to -13.07)**	16.2 (13.3 to 20.1)	12.7 (9.7 to 16.6)	**-22.0 (-30.30 to -14.13)**	--	--	--	--	--	--	7.8 (6.4 to 9.6)	6.2 (4.7 to 8.0)	**-20.9 (-29.25 to -13.07)**	32.6 (26.7 to 40.4)	25.5 (19.5 to 33.3)	**-21.9 (-30.17 to -14.00)**
Maternal hemorrhage	2.7 (1.9 to 3.7)	2.4 (1.7 to 3.4)	**-10.0 (-21.36 to 0.13)**	5.5 (3.9 to 7.7)	5.0 (3.4 to 7.0)	**-10.3 (-21.65 to -0.07)**	--	--	--	--	--	--	2.7 (1.9 to 3.7)	2.4 (1.7 to 3.4)	**-10.0 (-21.36 to 0.13)**	11.1 (7.9 to 15.4)	10.0 (6.9 to 14.0)	**-10.1 (-21.48 to 0.19)**
Maternal sepsis and other maternal infections	0.5 (0.4 to 0.5)	0.2 (0.2 to 0.3)	**-51.0 (-61.35 to -38.61)**	1.0 (0.8 to 1.1)	0.5 (0.4 to 0.5)	**-52.5 (-62.34 to -40.54)**	--	--	--	--	--	--	0.5 (0.4 to 0.5)	0.2 (0.2 to 0.3)	**-51.0 (-61.35 to -38.61)**	2.0 (1.7 to 2.3)	0.9 (0.8 to 1.1)	**-52.4 (-62.27 to -40.50)**
Maternal hypertensive disorders	1.4 (1.0 to 2.0)	1.2 (0.8 to 1.8)	**-12.9 (-22.85 to -2.99)**	2.9 (2.0 to 4.2)	2.5 (1.6 to 3.8)	**-14.3 (-23.90 to -4.68)**	--	--	--	--	--	--	1.4 (1.0 to 2.0)	1.2 (0.8 to 1.8)	**-12.9 (-22.85 to -2.99)**	5.8 (4.0 to 8.4)	5.0 (3.2 to 7.6)	**-14.2 (-23.80 to -4.56)**
Obstructed labor	0.3 (0.3 to 0.4)	0.2 (0.2 to 0.3)	**-35.8 (-45.64 to -23.15)**	0.7 (0.6 to 0.8)	0.4 (0.4 to 0.5)	**-37.4 (-46.95 to -25.08)**	--	--	--	--	--	--	0.3 (0.3 to 0.4)	0.2 (0.2 to 0.3)	**-35.8 (-45.64 to -23.15)**	1.4 (1.2 to 1.6)	0.9 (0.7 to 1.0)	**-37.3 (-46.88 to -25.02)**
Complications of abortion	0.7 (0.6 to 0.8)	0.4 (0.3 to 0.5)	**-42.9 (-52.81 to -29.68)**	1.5 (1.3 to 1.6)	0.8 (0.6 to 0.9)	**-45.8 (-55.27 to -33.46)**	--	--	--	--	--	--	0.7 (0.6 to 0.8)	0.4 (0.3 to 0.5)	**-42.9 (-52.81 to -29.68)**	2.9 (2.5 to 3.2)	1.6 (1.3 to 1.9)	**-45.8 (-55.26 to -33.43)**
Indirect maternal deaths	0.4 (0.3 to 0.5)	0.2 (0.2 to 0.3)	**-39.5 (-53.24 to -23.59)**	0.8 (0.7 to 1.0)	0.5 (0.4 to 0.6)	**-40.9 (-54.13 to -25.52)**	--	--	--	--	--	--	0.4 (0.3 to 0.5)	0.2 (0.2 to 0.3)	**-39.5 (-53.24 to -23.59)**	1.7 (1.4 to 1.9)	1.0 (0.8 to 1.2)	**-40.8 (-54.03 to -25.45)**
Late maternal deaths	0.2 (0.2 to 0.3)	0.1 (0.1 to 0.2)	**-34.3 (-50.44 to -11.02)**	0.5 (0.4 to 0.6)	0.3 (0.2 to 0.4)	**-36.2 (-52.10 to -13.39)**	--	--	--	--	--	--	0.2 (0.2 to 0.3)	0.1 (0.1 to 0.2)	**-34.3 (-50.44 to -11.02)**	1.0 (0.8 to 1.2)	0.6 (0.5 to 0.8)	**-36.2 (-52.06 to -13.24)**
Maternal deaths aggravated by HIV/AIDS	<0.05 (0.0 to <0.05)	<0.05 (0.0 to <0.05)	14.5 (-3.58 to 35.64)	<0.05 (0.0 to <0.05)	<0.05 (0.0 to <0.05)	8.0 (-9.02 to 28.27)	--	--	--	--	--	--	<0.05 (0.0 to <0.05)	<0.05 (0.0 to <0.05)	14.5 (-3.58 to 35.64)	<0.05 (0.0 to <0.05)	<0.05 (0.0 to <0.05)	7.8 (-9.22 to 28.01)
Other maternal disorders	1.6 (1.3 to 2.0)	1.3 (1.0 to 1.8)	**-17.2 (-30.85 to -1.72)**	3.4 (2.7 to 4.2)	2.8 (2.0 to 3.7)	**-18.2 (-31.61 to -2.94)**	--	--	--	--	--	--	1.6 (1.3 to 2.0)	1.3 (1.0 to 1.8)	**-17.2 (-30.85 to -1.72)**	6.8 (5.5 to 8.5)	5.5 (4.1 to 7.4)	**-18.0 (-31.48 to -2.77)**
**Neonatal disorders**	275.7 (256.4 to 296.6)	197.8 (164.5 to 233.9)	**-28.3 (-39.05 to -16.19)**	756.4 (708.3 to 810.8)	492.2 (412.0 to 586.0)	**-34.9 (-44.93 to -22.80)**	157.7 (146.0 to 170.4)	108.2 (89.4 to 130.2)	**-31.4 (-43.36 to -19.33)**	851.4 (793.6 to 917.0)	535.3 (442.8 to 641.1)	**-37.1 (-47.77 to -25.41)**	117.9 (105.3 to 132.2)	89.6 (72.5 to 107.2)	**-24.0 (-36.35 to -11.01)**	656.8 (586.1 to 737.4)	447.1 (362.1 to 537.4)	**-31.9 (-43.14 to -19.40)**
Preterm birth complications	182.9 (164.1 to 209.4)	148.7 (123.0 to 177.5)	**-18.5 (-32.17 to -3.00)**	501.3 (451.4 to 574.4)	368.6 (305.7 to 442.6)	**-26.3 (-39.28 to -10.46)**	104.0 (91.2 to 123.4)	81.4 (65.4 to 97.7)	**-21.4 (-36.68 to -3.01)**	561.1 (494.1 to 668.3)	401.8 (322.7 to 487.2)	**-28.1 (-42.90 to -10.34)**	78.9 (67.4 to 91.6)	67.3 (54.4 to 80.8)	**-14.5 (-27.79 to 0.08)**	438.6 (374.1 to 512.2)	333.8 (267.2 to 402.3)	**-23.8 (-36.21 to -9.30)**
Neonatal encephalopathy due to birth asphyxia and trauma	66.1 (49.2 to 75.3)	26.7 (20.9 to 34.0)	**-59.1 (-68.14 to -44.66)**	183.2 (134.6 to 210.1)	68.4 (52.4 to 88.7)	**-62.3 (-71.10 to -47.79)**	37.7 (23.7 to 45.6)	14.1 (10.5 to 19.4)	**-61.5 (-71.95 to -41.55)**	205.0 (126.9 to 250.2)	71.1 (52.1 to 100.3)	**-64.4 (-74.46 to -44.77)**	28.4 (21.4 to 35.7)	12.6 (9.5 to 16.5)	**-55.3 (-65.16 to -42.83)**	160.3 (119.8 to 203.1)	65.6 (48.5 to 87.0)	**-58.9 (-68.29 to -46.80)**
Neonatal sepsis and other neonatal infections	7.9 (3.8 to 11.9)	5.3 (3.0 to 11.0)	-29.3 (-64.50 to 31.01)	22.6 (10.9 to 34.1)	14.9 (8.5 to 30.8)	-30.9 (-65.32 to 27.97)	4.9 (1.7 to 8.4)	3.1 (1.5 to 7.6)	-31.5 (-68.66 to 30.91)	27.3 (9.5 to 47.0)	17.0 (8.1 to 41.4)	-33.1 (-69.37 to 27.94)	3.0 (1.5 to 5.2)	2.2 (1.2 to 4.3)	-24.0 (-59.73 to 39.22)	17.6 (8.6 to 30.8)	12.6 (6.7 to 24.5)	-25.8 (-60.67 to 35.96)
Hemolytic disease and other neonatal jaundice	5.5 (3.5 to 8.8)	4.3 (2.7 to 6.3)	-18.5 (-57.02 to 22.93)	12.6 (8.2 to 20.1)	8.8 (5.5 to 12.7)	-28.1 (-61.85 to 5.81)	3.2 (1.9 to 6.8)	2.3 (1.3 to 3.6)	-21.4 (-75.04 to 30.07)	14.9 (8.7 to 31.0)	9.3 (5.5 to 14.6)	-30.3 (-77.39 to 14.72)	2.2 (1.4 to 3.9)	2.0 (1.0 to 3.4)	-5.5 (-50.83 to 74.40)	10.3 (6.4 to 17.5)	8.2 (4.1 to 13.7)	-17.5 (-56.10 to 47.64)
Other neonatal disorders	13.4 (10.4 to 19.7)	12.7 (8.6 to 17.5)	-3.7 (-38.15 to 35.78)	36.7 (28.4 to 53.8)	31.6 (21.4 to 43.3)	-12.6 (-44.38 to 23.68)	8.0 (5.6 to 12.4)	7.3 (4.5 to 11.5)	-7.1 (-43.27 to 38.41)	43.1 (30.2 to 67.2)	36.0 (22.2 to 56.9)	-14.9 (-48.76 to 27.23)	5.4 (3.9 to 8.7)	5.4 (3.3 to 8.0)	2.8 (-48.51 to 49.75)	30.1 (21.7 to 48.1)	27.0 (16.4 to 39.8)	-7.8 (-53.70 to 34.33)
**Nutritional deficiencies**	157.2 (107.7 to 224.0)	160.1 (106.2 to 232.7)	1.7 (-3.96 to 6.67)	381.0 (258.3 to 548.3)	364.4 (241.7 to 532.2)	**-4.5 (-9.22 to -0.51)**	75.0 (51.1 to 108.0)	80.0 (53.1 to 115.7)	**6.5 (2.92 to 11.00)**	381.1 (258.4 to 546.3)	376.0 (249.9 to 544.9)	-1.4 (-4.18 to 1.80)	82.1 (56.3 to 117.2)	80.1 (52.7 to 116.5)	-2.6 (-12.48 to 4.95)	378.8 (255.4 to 548.2)	351.3 (228.3 to 512.1)	**-7.3 (-16.41 to -0.95)**
Protein-energy malnutrition	1.7 (1.2 to 2.8)	1.1 (0.6 to 1.6)	**-36.7 (-58.95 to -15.09)**	3.1 (2.2 to 4.9)	1.5 (0.9 to 2.1)	**-51.0 (-66.59 to -34.93)**	0.7 (0.3 to 1.2)	0.5 (0.2 to 0.9)	-23.0 (-47.92 to 6.54)	2.8 (1.5 to 4.9)	1.6 (0.8 to 2.7)	**-43.2 (-60.33 to -23.62)**	1.0 (0.6 to 1.8)	0.6 (0.2 to 0.9)	**-45.4 (-68.28 to -22.54)**	3.3 (1.9 to 5.6)	1.4 (0.6 to 2.2)	**-56.1 (-73.22 to -39.33)**
Iodine deficiency	16.1 (9.4 to 26.0)	11.0 (6.3 to 18.2)	**-30.9 (-51.61 to -4.45)**	32.2 (19.0 to 52.1)	19.9 (11.4 to 32.9)	**-37.8 (-56.09 to -14.06)**	5.0 (2.7 to 8.4)	3.5 (1.9 to 6.3)	-28.9 (-53.98 to 21.50)	20.4 (11.1 to 34.2)	12.7 (6.9 to 23.2)	**-36.5 (-59.43 to 8.71)**	11.1 (6.2 to 18.3)	7.5 (4.2 to 12.8)	**-30.8 (-54.95 to 3.02)**	43.9 (24.7 to 72.2)	27.0 (15.3 to 46.1)	**-37.2 (-59.02 to -6.95)**
Iron-deficiency anemia	137.1 (92.9 to 198.1)	147.4 (98.3 to 215.4)	**7.4 (2.33 to 11.16)**	341.9 (230.0 to 497.3)	342.2 (226.9 to 499.3)	0.0 (-4.49 to 3.13)	68.6 (46.3 to 99.0)	75.7 (50.4 to 110.2)	**10.3 (7.93 to 12.56)**	354.8 (239.0 to 509.8)	360.9 (240.8 to 521.9)	**1.7 (-0.07 to 3.34)**	68.5 (46.3 to 99.2)	71.7 (47.4 to 104.0)	4.5 (-5.78 to 11.24)	327.5 (219.2 to 480.2)	322.1 (210.1 to 470.2)	-1.7 (-11.00 to 4.09)
Other nutritional deficiencies	2.2 (1.1 to 3.4)	0.6 (0.4 to 1.2)	**-72.0 (-81.98 to -44.62)**	3.7 (2.0 to 5.5)	0.8 (0.5 to 1.5)	**-77.4 (-84.47 to -59.65)**	0.8 (0.3 to 1.3)	0.3 (0.1 to 0.6)	**-63.2 (-77.19 to -25.16)**	3.2 (1.3 to 5.2)	0.8 (0.4 to 1.9)	**-73.0 (-82.25 to -47.75)**	1.5 (0.6 to 2.5)	0.3 (0.1 to 0.7)	**-76.4 (-86.82 to -49.63)**	4.1 (1.8 to 6.7)	0.8 (0.4 to 1.6)	**-79.7 (-86.97 to -61.63)**
**Other communicable, maternal, neonatal, and nutritional diseases**	27.9 (21.8 to 36.4)	28.1 (20.7 to 38.0)	1.5 (-21.76 to 20.83)	61.6 (48.0 to 80.5)	55.7 (41.0 to 76.7)	-8.9 (-29.90 to 10.75)	16.8 (13.1 to 22.5)	17.0 (12.7 to 21.9)	2.2 (-21.06 to 20.40)	75.8 (59.0 to 101.2)	68.5 (51.1 to 90.0)	-8.9 (-29.43 to 6.14)	11.1 (8.5 to 14.9)	11.1 (7.8 to 15.7)	0.7 (-26.45 to 32.68)	47.4 (36.3 to 64.1)	42.8 (29.4 to 63.1)	-8.9 (-36.03 to 28.44)
Sexually transmitted diseases excluding HIV	7.5 (4.8 to 12.4)	7.6 (4.7 to 12.9)	1.0 (-9.21 to 11.34)	15.4 (9.9 to 25.6)	14.1 (8.7 to 23.7)	-8.8 (-20.38 to 2.50)	3.9 (2.5 to 6.4)	4.1 (2.5 to 6.7)	3.1 (-8.51 to 14.52)	16.7 (10.8 to 26.8)	15.6 (9.8 to 25.3)	-6.5 (-19.07 to 5.51)	3.6 (2.2 to 6.1)	3.5 (2.1 to 6.2)	-1.3 (-14.70 to 10.39)	14.0 (8.7 to 23.8)	12.5 (7.3 to 22.2)	**-11.4 (-24.74 to 0.82)**
Syphilis	1.8 (1.3 to 2.5)	1.6 (1.2 to 2.2)	-8.0 (-33.91 to 23.07)	3.9 (2.6 to 5.9)	2.9 (2.0 to 4.4)	-23.2 (-49.38 to 12.92)	0.8 (0.6 to 1.2)	0.8 (0.6 to 1.1)	-4.3 (-32.50 to 30.18)	3.8 (2.6 to 5.7)	3.0 (2.0 to 4.4)	-20.7 (-47.31 to 13.88)	1.0 (0.7 to 1.3)	0.9 (0.6 to 1.1)	-11.1 (-36.75 to 21.17)	4.0 (2.6 to 6.1)	2.9 (2.0 to 4.4)	-25.3 (-52.33 to 12.73)

Chlamydial infection	2.8 (1.5 to 4.9)	2.9 (1.6 to 5.1)	4.6 (-6.92 to 18.71)	5.8 (3.2 to 10.3)	5.8 (3.1 to 10.3)	-0.3 (-11.82 to 13.76)	2.1 (1.2 to 3.5)	2.2 (1.2 to 3.8)	7.2 (-7.30 to 23.27)	8.6 (4.9 to 14.9)	8.8 (4.8 to 15.3)	2.2 (-12.08 to 18.12)	0.7 (0.4 to 1.3)	0.7 (0.3 to 1.3)	-2.8 (-21.11 to 12.91)	2.9 (1.5 to 5.6)	2.7 (1.3 to 5.3)	-7.5 (-25.45 to 8.15)
Gonococcal infection	0.8 (0.5 to 1.3)	0.7 (0.4 to 1.2)	-7.4 (-18.17 to 2.72)	1.6 (1.0 to 2.7)	1.5 (0.8 to 2.5)	**-10.0 (-21.01 to 1.03)**	0.4 (0.3 to 0.7)	0.4 (0.2 to 0.6)	-9.6 (-20.98 to 3.97)	1.8 (1.1 to 2.9)	1.6 (0.9 to 2.6)	**-11.8 (-23.57 to 1.48)**	0.4 (0.2 to 0.6)	0.4 (0.2 to 0.6)	-4.6 (-21.71 to 14.01)	1.5 (0.9 to 2.6)	1.4 (0.7 to 2.6)	-7.3 (-24.21 to 12.73)
Trichomoniasis	0.2 (0.1 to 0.5)	0.3 (0.1 to 0.6)	13.5 (-9.77 to 34.02)	0.5 (0.2 to 1.0)	0.5 (0.2 to 1.1)	7.5 (-15.58 to 27.40)	--	--	--	--	--	--	0.2 (0.1 to 0.5)	0.3 (0.1 to 0.6)	13.5 (-9.77 to 34.02)	1.0 (0.4 to 2.0)	1.0 (0.4 to 2.2)	7.6 (-15.64 to 27.63)
Genital herpes	1.6 (0.5 to 3.8)	1.8 (0.5 to 4.3)	**11.0 (3.85 to 18.32)**	2.9 (0.9 to 7.1)	2.8 (0.9 to 6.8)	-4.8 (-10.79 to 1.36)	0.6 (0.2 to 1.5)	0.7 (0.2 to 1.7)	**14.9 (2.48 to 26.11)**	2.3 (0.7 to 5.7)	2.2 (0.7 to 5.3)	-4.2 (-14.27 to 4.76)	1.0 (0.3 to 2.4)	1.1 (0.3 to 2.6)	**8.8 (-0.12 to 18.83)**	3.5 (1.1 to 8.4)	3.3 (1.0 to 8.2)	-4.6 (-12.51 to 4.13)
Other sexually transmitted diseases	0.3 (0.3 to 0.4)	0.3 (0.2 to 0.4)	-3.4 (-13.43 to 6.47)	0.7 (0.5 to 0.9)	0.6 (0.4 to 0.8)	**-10.7 (-19.88 to -0.74)**	<0.05 (0.0 to <0.05)	<0.05 (0.0 to <0.05)	**-46.5 (-64.94 to -17.15)**	0.1 (0.1 to 0.2)	0.1 (0.0 to 0.1)	**-48.9 (-70.28 to -27.14)**	0.3 (0.2 to 0.4)	0.3 (0.2 to 0.4)	1.9 (-8.92 to 12.76)	1.2 (0.9 to 1.5)	1.1 (0.8 to 1.5)	-5.3 (-15.03 to 5.14)
Hepatitis	3.5 (3.0 to 4.2)	4.1 (3.5 to 4.9)	**17.3 (8.13 to 30.52)**	7.2 (6.0 to 8.6)	7.2 (6.1 to 8.6)	1.1 (-6.57 to 11.82)	2.3 (2.0 to 2.7)	2.6 (2.2 to 3.1)	**12.2 (0.56 to 28.61)**	9.6 (8.3 to 11.1)	9.1 (7.8 to 10.8)	-4.6 (-14.11 to 8.46)	1.2 (0.9 to 1.5)	1.5 (1.2 to 1.9)	**27.3 (15.81 to 41.75)**	4.8 (3.8 to 6.2)	5.4 (4.3 to 6.7)	**11.9 (2.13 to 24.31)**
Hepatitis A	1.6 (1.1 to 2.1)	1.9 (1.3 to 2.7)	**24.5 (12.20 to 38.38)**	3.4 (2.4 to 4.5)	3.7 (2.6 to 5.0)	**10.5 (1.20 to 21.37)**	0.9 (0.6 to 1.2)	1.1 (0.7 to 1.5)	**23.3 (10.18 to 38.59)**	3.8 (2.6 to 5.3)	4.1 (2.7 to 5.7)	8.3 (-2.36 to 19.91)	0.7 (0.5 to 0.9)	0.9 (0.6 to 1.2)	**26.1 (12.12 to 42.64)**	3.0 (2.1 to 4.1)	3.4 (2.4 to 4.5)	**13.7 (2.18 to 27.38)**
Hepatitis B	1.7 (1.3 to 2.1)	1.8 (1.3 to 2.5)	4.8 (-10.27 to 22.21)	3.3 (2.5 to 4.1)	2.9 (2.0 to 4.0)	**-13.0 (-25.40 to 1.55)**	1.3 (1.0 to 1.6)	1.3 (0.9 to 1.8)	-1.5 (-17.33 to 19.02)	5.2 (4.0 to 6.4)	4.2 (2.9 to 5.8)	**-19.0 (-31.82 to -2.19)**	0.4 (0.3 to 0.5)	0.5 (0.3 to 0.7)	**24.9 (4.54 to 45.96)**	1.5 (1.1 to 2.0)	1.6 (1.0 to 2.3)	5.1 (-11.46 to 22.12)
Hepatitis C	0.2 (0.1 to 0.4)	0.4 (0.2 to 0.7)	**55.1 (11.12 to 110.42)**	0.4 (0.2 to 0.8)	0.6 (0.2 to 1.1)	30.4 (-6.08 to 74.88)	0.1 (0.1 to 0.3)	0.2 (0.1 to 0.5)	**64.9 (14.41 to 136.39)**	0.6 (0.3 to 1.0)	0.8 (0.3 to 1.6)	35.4 (-6.25 to 91.35)	0.1 (0.1 to 0.1)	0.1 (0.1 to 0.2)	**41.2 (-1.47 to 91.25)**	0.3 (0.2 to 0.5)	0.4 (0.2 to 0.7)	22.3 (-13.45 to 64.71)
Leprosy	--	<0.05 (0.0 to <0.05)	--	--	<0.05 (0.0 to <0.05)	--	--	<0.05 (0.0 to <0.05)	--	--	<0.05 (0.0 to <0.05)	--	--	<0.05 (0.0 to <0.05)	--	--	<0.05 (0.0 to <0.05)	--
Other infectious diseases	16.9 (13.5 to 22.8)	16.4 (11.4 to 21.2)	-0.7 (-34.55 to 31.75)	39.0 (31.0 to 51.9)	34.4 (24.2 to 45.7)	-10.2 (-39.74 to 19.99)	10.5 (8.3 to 14.9)	10.3 (7.1 to 13.2)	0.5 (-32.22 to 28.00)	49.5 (38.4 to 69.2)	43.7 (31.0 to 56.2)	-10.0 (-38.00 to 12.44)	6.3 (5.0 to 8.9)	6.0 (3.7 to 9.0)	-1.9 (-42.43 to 52.91)	28.6 (22.2 to 40.5)	24.9 (15.3 to 40.7)	-10.1 (-48.89 to 50.20)
**Non-communicable diseases**	13,000.6 (11,649.4 to 14,501.9)	12,062.3 (10,431.5 to 13,863.2)	**-7.3 (-11.00 to -4.06)**	21,935.7 (19,422.1 to 24,742.2)	17,159.1 (14,600.3 to 20,042.1)	**-21.9 (-25.27 to -18.89)**	6,580.6 (5,976.0 to 7,252.8)	5,989.1 (5,234.3 to 6,823.2)	**-9.1 (-13.38 to -5.33)**	24,700.8 (22,300.4 to 27,401.3)	17,893.5 (15,442.9 to 20,583.8)	**-27.6 (-31.36 to -24.39)**	6,420.0 (5,654.9 to 7,274.7)	6,073.2 (5,173.0 to 7,044.6)	**-5.5 (-9.45 to -1.88)**	19,913.6 (17,255.9 to 22,904.1)	16,562.4 (13,840.5 to 19,562.1)	**-16.9 (-20.49 to -13.66)**
**Neoplasms**	2,580.2 (2,504.9 to 2,677.7)	2,340.1 (2,196.9 to 2,468.1)	**-9.3 (-14.34 to -4.81)**	4,164.1 (4,039.4 to 4,309.0)	3,033.9 (2,855.8 to 3,204.4)	**-27.1 (-31.06 to -23.46)**	1,355.2 (1,314.7 to 1,400.8)	1,243.0 (1,150.6 to 1,336.4)	**-8.3 (-15.12 to -1.82)**	4,839.5 (4,696.5 to 4,998.8)	3,374.3 (3,118.4 to 3,624.2)	**-30.3 (-35.57 to -25.30)**	1,224.9 (1,161.4 to 1,326.5)	1,097.1 (988.1 to 1,187.5)	**-10.4 (-18.91 to -2.46)**	3,729.1 (3,522.0 to 3,998.5)	2,755.6 (2,508.0 to 2,983.2)	**-26.1 (-32.37 to -19.62)**
Esophageal cancer	91.7 (83.9 to 100.9)	111.0 (97.5 to 136.5)	**21.1 (5.56 to 41.37)**	144.1 (132.4 to 158.6)	140.0 (122.5 to 171.0)	-2.9 (-15.31 to 12.63)	60.5 (52.6 to 68.9)	82.1 (69.4 to 105.7)	**35.7 (13.97 to 64.47)**	214.0 (186.3 to 244.5)	220.4 (186.0 to 281.1)	3.0 (-13.41 to 24.09)	31.2 (29.7 to 33.7)	28.9 (25.8 to 32.7)	-7.1 (-17.77 to 5.25)	84.2 (80.2 to 90.6)	65.6 (58.2 to 74.6)	**-22.1 (-31.27 to -11.08)**
Stomach cancer	154.6 (141.7 to 168.8)	84.3 (74.9 to 95.6)	**-45.5 (-49.57 to -40.56)**	234.9 (215.7 to 255.8)	102.3 (91.1 to 115.7)	**-56.5 (-59.67 to -52.50)**	101.3 (98.5 to 103.6)	53.4 (48.9 to 59.6)	**-47.3 (-51.81 to -40.26)**	355.4 (345.9 to 363.2)	140.4 (128.5 to 157.4)	**-60.5 (-63.91 to -55.17)**	53.3 (40.6 to 68.2)	30.9 (22.8 to 40.6)	**-42.1 (-49.14 to -28.74)**	140.6 (107.4 to 179.1)	68.4 (51.0 to 89.5)	**-51.3 (-56.99 to -41.14)**
Liver cancer	28.2 (24.7 to 35.3)	49.4 (37.5 to 57.2)	**76.7 (30.89 to 109.35)**	45.6 (39.9 to 57.0)	64.1 (49.4 to 74.2)	**41.4 (6.34 to 66.45)**	17.8 (14.7 to 24.9)	31.0 (20.7 to 38.0)	**77.0 (9.42 to 120.23)**	63.4 (52.5 to 88.3)	85.1 (57.1 to 104.5)	35.9 (-15.49 to 67.69)	10.4 (9.6 to 13.4)	18.4 (12.9 to 21.1)	**77.8 (16.83 to 105.62)**	30.8 (28.4 to 38.7)	44.5 (31.6 to 50.8)	**45.5 (-1.32 to 68.14)**
Liver cancer due to hepatitis B	6.1 (4.9 to 7.9)	12.0 (8.9 to 15.0)	**100.6 (39.89 to 172.55)**	10.4 (8.4 to 13.5)	16.4 (12.5 to 20.6)	**60.3 (13.72 to 115.42)**	4.3 (3.2 to 6.0)	7.6 (5.0 to 10.3)	**83.3 (12.24 to 162.70)**	15.5 (11.6 to 21.7)	21.9 (14.3 to 29.5)	44.1 (-10.32 to 106.01)	1.8 (1.4 to 2.5)	4.3 (2.9 to 6.0)	**146.8 (46.06 to 292.05)**	5.9 (4.6 to 8.0)	11.2 (7.8 to 15.2)	**95.0 (20.39 to 205.93)**
Liver cancer due to hepatitis C	6.3 (5.0 to 8.0)	25.0 (18.8 to 30.7)	**305.6 (191.21 to 449.16)**	9.9 (7.9 to 12.7)	31.9 (24.1 to 39.3)	**227.0 (136.75 to 338.72)**	4.0 (3.0 to 5.7)	15.6 (10.0 to 20.4)	**294.1 (136.67 to 474.04)**	14.3 (10.5 to 20.3)	42.2 (27.4 to 55.4)	**202.3 (84.40 to 338.19)**	2.2 (1.7 to 3.0)	9.5 (6.9 to 11.7)	**336.7 (168.82 to 484.69)**	6.3 (4.8 to 8.5)	22.4 (16.4 to 28.0)	**262.7 (128.24 to 398.55)**
Liver cancer due to alcohol use	13.0 (10.9 to 16.7)	9.9 (6.8 to 12.5)	**-23.3 (-50.65 to 1.57)**	20.4 (17.1 to 26.2)	12.2 (8.4 to 15.5)	**-39.6 (-60.35 to -20.16)**	8.3 (6.4 to 11.8)	7.2 (4.3 to 9.7)	-12.4 (-51.79 to 27.20)	29.4 (22.4 to 41.5)	19.0 (11.4 to 25.9)	**-34.0 (-63.48 to -4.17)**	4.7 (4.0 to 5.9)	2.7 (1.8 to 3.4)	**-41.3 (-62.42 to -23.49)**	13.1 (11.0 to 16.1)	6.0 (4.2 to 7.6)	**-53.6 (-68.79 to -40.21)**
Liver cancer due to other causes	2.9 (2.3 to 3.5)	2.5 (1.8 to 3.3)	-10.4 (-40.75 to 20.73)	4.9 (4.0 to 6.0)	3.5 (2.5 to 4.5)	**-28.2 (-51.26 to -4.05)**	1.1 (0.8 to 1.6)	0.7 (0.4 to 1.0)	**-37.7 (-66.47 to -0.89)**	4.2 (3.0 to 6.0)	2.0 (1.2 to 2.8)	**-51.3 (-73.23 to -23.13)**	1.7 (1.3 to 2.2)	1.8 (1.2 to 2.5)	9.0 (-35.05 to 63.07)	5.5 (4.2 to 6.9)	4.9 (3.1 to 6.6)	-10.3 (-44.20 to 32.00)
Larynx cancer	17.8 (13.7 to 22.0)	13.5 (10.7 to 16.8)	**-24.1 (-33.82 to -14.87)**	28.8 (22.2 to 35.7)	17.3 (13.7 to 21.4)	**-39.9 (-47.07 to -32.97)**	14.7 (10.8 to 18.7)	10.7 (7.7 to 13.8)	**-27.3 (-37.94 to -16.50)**	52.2 (38.2 to 66.4)	28.7 (21.0 to 37.2)	**-44.9 (-52.59 to -36.96)**	3.1 (2.1 to 4.2)	2.8 (1.8 to 4.0)	-8.5 (-28.19 to 13.42)	9.2 (6.2 to 12.6)	7.0 (4.5 to 10.0)	**-23.8 (-39.98 to -5.89)**
Tracheal, bronchus and lung cancer	643.3 (626.7 to 675.7)	506.8 (463.0 to 548.8)	**-21.2 (-28.06 to -14.28)**	1,002.3 (975.3 to 1,049.1)	632.7 (578.3 to 687.1)	**-36.9 (-42.43 to -31.32)**	444.3 (433.0 to 452.3)	293.9 (258.7 to 326.9)	**-33.8 (-42.40 to -25.97)**	1,545.3 (1,505.5 to 1,572.9)	772.7 (678.3 to 860.7)	**-50.0 (-56.58 to -43.96)**	199.0 (187.9 to 233.0)	212.9 (174.3 to 235.9)	7.2 (-13.69 to 20.23)	574.3 (535.3 to 661.0)	510.9 (422.6 to 564.9)	**-10.9 (-26.04 to -0.43)**
Breast cancer	313.9 (263.6 to 364.2)	239.1 (192.3 to 284.4)	**-23.8 (-30.08 to -14.38)**	535.2 (448.9 to 615.5)	322.6 (263.1 to 385.1)	**-39.7 (-44.88 to -32.04)**	1.8 (0.8 to 3.7)	1.5 (0.9 to 2.8)	-13.6 (-34.26 to 19.22)	6.3 (3.0 to 13.2)	4.0 (2.4 to 7.4)	**-34.5 (-50.00 to -10.58)**	312.1 (262.2 to 362.7)	237.6 (191.5 to 282.5)	**-23.8 (-30.14 to -14.42)**	1,012.1 (847.5 to 1,162.0)	620.3 (507.2 to 742.2)	**-38.7 (-44.00 to -30.87)**
Cervical cancer	42.2 (33.1 to 45.2)	24.7 (20.4 to 28.7)	**-41.4 (-49.54 to -33.63)**	75.4 (59.2 to 81.0)	37.0 (30.2 to 43.0)	**-50.8 (-57.70 to -44.20)**	--	--	--	--	--	--	42.2 (33.1 to 45.2)	24.7 (20.4 to 28.7)	**-41.4 (-49.54 to -33.63)**	145.6 (114.3 to 156.4)	72.4 (59.0 to 84.2)	**-50.1 (-57.13 to -43.39)**
Uterine cancer	18.4 (17.0 to 23.3)	25.9 (19.4 to 30.4)	**41.7 (-2.22 to 65.65)**	28.5 (26.3 to 35.7)	32.1 (24.2 to 37.7)	13.4 (-21.27 to 32.75)	--	--	--	--	--	--	18.4 (17.0 to 23.3)	25.9 (19.4 to 30.4)	**41.7 (-2.22 to 65.65)**	52.2 (48.3 to 65.1)	61.3 (46.3 to 71.9)	17.9 (-18.14 to 37.99)
Prostate cancer	114.7 (89.5 to 151.1)	138.1 (93.3 to 186.2)	21.1 (-23.89 to 43.41)	159.4 (125.2 to 209.2)	154.1 (102.5 to 205.7)	-2.7 (-39.58 to 14.27)	114.7 (89.5 to 151.1)	138.1 (93.3 to 186.2)	21.1 (-23.89 to 43.41)	400.3 (313.3 to 527.5)	341.4 (229.1 to 456.2)	**-14.2 (-45.70 to 1.21)**	--	--	--	--	--	--
Colon and rectum cancer	313.5 (304.9 to 324.6)	261.8 (243.1 to 278.4)	**-16.5 (-22.59 to -10.92)**	484.8 (471.5 to 500.8)	322.9 (300.0 to 343.9)	**-33.4 (-38.34 to -28.84)**	165.2 (160.5 to 168.8)	153.9 (140.9 to 165.6)	**-6.9 (-14.17 to 0.51)**	584.8 (567.9 to 597.6)	407.5 (373.0 to 439.4)	**-30.3 (-35.80 to -24.60)**	148.2 (142.4 to 160.4)	108.0 (97.7 to 117.5)	**-27.1 (-34.88 to -20.31)**	408.2 (391.8 to 440.7)	246.7 (221.9 to 269.2)	**-39.5 (-46.07 to -33.75)**
Lip and oral cavity cancer	25.9 (24.6 to 27.9)	28.1 (22.6 to 30.8)	8.8 (-17.92 to 19.47)	43.3 (41.3 to 46.5)	38.1 (30.6 to 41.9)	**-12.0 (-33.64 to -3.30)**	18.0 (17.2 to 19.5)	19.4 (14.0 to 21.7)	8.1 (-28.79 to 21.61)	65.7 (62.8 to 71.2)	55.0 (39.9 to 61.5)	**-16.1 (-44.06 to -5.56)**	7.9 (7.0 to 8.9)	8.7 (7.4 to 10.1)	10.5 (-6.03 to 24.66)	23.9 (21.3 to 26.8)	22.1 (18.7 to 25.6)	-7.5 (-22.12 to 4.67)
Nasopharynx cancer	6.1 (4.8 to 8.0)	3.6 (3.0 to 5.0)	**-41.8 (-48.00 to -29.30)**	11.2 (8.8 to 14.7)	5.4 (4.4 to 7.6)	**-51.9 (-56.73 to -42.09)**	4.1 (3.3 to 4.9)	2.4 (2.0 to 3.2)	**-40.5 (-48.12 to -25.90)**	15.6 (12.4 to 18.7)	7.5 (6.3 to 10.0)	**-51.8 (-57.86 to -40.72)**	2.0 (0.9 to 3.8)	1.1 (0.6 to 2.4)	**-44.3 (-53.12 to -27.70)**	7.1 (3.3 to 13.3)	3.4 (1.8 to 7.0)	**-52.6 (-59.57 to -39.97)**
Other pharynx cancer	12.1 (10.9 to 14.2)	13.4 (9.9 to 16.1)	11.1 (-20.14 to 32.51)	20.5 (18.6 to 24.2)	18.3 (13.6 to 21.9)	-10.8 (-35.39 to 6.16)	8.7 (7.8 to 10.9)	10.0 (6.6 to 12.5)	14.8 (-26.25 to 44.38)	31.7 (28.5 to 39.3)	28.1 (18.9 to 35.3)	-10.8 (-41.73 to 11.61)	3.3 (2.7 to 4.0)	3.4 (2.8 to 4.2)	2.0 (-12.11 to 17.76)	10.5 (8.6 to 12.6)	8.9 (7.3 to 11.0)	**-15.1 (-25.98 to -2.08)**
Gallbladder and biliary tract cancer	20.5 (15.6 to 24.1)	14.6 (11.1 to 18.1)	**-29.0 (-39.23 to -11.00)**	30.8 (23.6 to 36.3)	17.6 (13.4 to 21.9)	**-42.9 (-51.19 to -28.40)**	6.9 (5.1 to 8.1)	4.8 (3.5 to 6.2)	**-30.6 (-42.73 to -10.00)**	24.3 (17.9 to 28.3)	12.6 (9.2 to 16.2)	**-47.9 (-56.96 to -32.99)**	13.6 (9.5 to 17.0)	9.8 (6.7 to 12.5)	**-28.1 (-39.88 to -9.87)**	36.0 (25.6 to 44.9)	21.9 (15.1 to 28.0)	**-38.9 (-48.79 to -22.85)**
Pancreatic cancer	113.2 (110.5 to 117.7)	124.7 (115.9 to 132.6)	**10.2 (1.33 to 17.96)**	176.1 (171.9 to 182.8)	156.1 (144.8 to 166.4)	**-11.3 (-18.52 to -4.93)**	59.9 (58.4 to 61.2)	67.2 (61.7 to 72.7)	**12.3 (2.99 to 21.65)**	211.7 (206.5 to 216.2)	180.1 (165.1 to 194.8)	**-14.9 (-22.05 to -7.84)**	53.3 (51.2 to 58.0)	57.5 (51.5 to 62.5)	8.0 (-4.21 to 18.14)	146.6 (140.7 to 159.2)	133.6 (119.8 to 145.5)	**-8.8 (-19.11 to 0.33)**
Malignant skin melanoma	37.2 (27.0 to 53.9)	45.6 (30.6 to 64.9)	**22.9 (-12.92 to 38.30)**	65.7 (47.6 to 95.1)	65.6 (44.4 to 93.9)	-0.0 (-28.71 to 12.23)	18.1 (10.0 to 31.6)	25.8 (11.8 to 41.8)	**43.3 (-31.39 to 68.33)**	68.2 (37.4 to 118.9)	76.5 (35.1 to 125.3)	13.1 (-44.25 to 32.72)	19.0 (13.3 to 26.0)	19.9 (13.4 to 25.8)	4.5 (-11.22 to 18.66)	63.9 (44.7 to 87.1)	55.4 (37.8 to 72.5)	**-13.2 (-25.86 to -1.25)**
Non-melanoma skin cancer	7.4 (5.7 to 9.8)	8.7 (6.4 to 11.4)	**16.4 (6.20 to 28.68)**	11.1 (8.4 to 14.7)	10.1 (7.4 to 13.3)	**-9.2 (-17.10 to -0.02)**	4.4 (2.8 to 6.6)	5.1 (3.2 to 7.7)	**16.6 (4.69 to 32.06)**	15.8 (10.1 to 23.9)	13.3 (8.5 to 20.2)	**-15.8 (-24.09 to -5.27)**	3.1 (2.4 to 3.8)	3.6 (2.7 to 4.6)	**16.5 (0.00 to 36.83)**	7.6 (6.1 to 9.5)	7.3 (5.6 to 9.3)	-3.7 (-16.18 to 10.36)
Ovarian cancer	86.1 (81.8 to 94.3)	79.0 (70.2 to 87.4)	-8.2 (-18.44 to 1.55)	144.2 (136.1 to 157.3)	104.4 (92.8 to 116.0)	**-27.5 (-35.57 to -19.52)**	--	--	--	--	--	--	86.1 (81.8 to 94.3)	79.0 (70.2 to 87.4)	-8.2 (-18.44 to 1.55)	273.4 (257.8 to 298.5)	201.7 (179.3 to 224.0)	**-26.2 (-34.37 to -18.00)**
Testicular cancer	5.5 (3.9 to 7.0)	3.7 (2.8 to 4.9)	**-32.3 (-45.15 to -9.66)**	11.1 (7.8 to 13.9)	6.9 (5.1 to 9.1)	**-37.7 (-49.43 to -17.93)**	5.5 (3.9 to 7.0)	3.7 (2.8 to 4.9)	**-32.3 (-45.15 to -9.66)**	22.4 (15.7 to 28.1)	13.8 (10.3 to 18.2)	**-38.0 (-49.74 to -18.27)**	--	--	--	--	--	--
Kidney cancer	56.0 (51.9 to 60.7)	63.0 (56.9 to 69.9)	**12.7 (1.19 to 22.38)**	92.4 (85.7 to 99.9)	82.4 (74.0 to 91.6)	**-10.7 (-20.12 to -2.48)**	35.1 (33.9 to 36.3)	41.0 (36.0 to 45.8)	**16.7 (1.84 to 30.76)**	126.8 (122.2 to 130.8)	112.3 (98.8 to 125.5)	**-11.4 (-22.76 to -0.72)**	20.9 (17.0 to 25.5)	22.1 (17.4 to 26.5)	6.2 (-11.57 to 25.02)	63.1 (51.3 to 75.6)	55.3 (44.3 to 65.8)	**-12.1 (-24.05 to 1.19)**
Bladder cancer	76.9 (64.8 to 85.8)	53.2 (46.1 to 61.3)	**-30.7 (-35.62 to -23.75)**	113.2 (94.8 to 126.4)	61.6 (53.5 to 71.1)	**-45.5 (-49.44 to -39.54)**	55.1 (44.2 to 58.6)	37.0 (30.6 to 42.7)	**-32.7 (-39.13 to -23.53)**	192.1 (153.8 to 204.2)	93.9 (77.7 to 108.5)	**-51.1 (-55.51 to -44.24)**	21.8 (15.4 to 29.7)	16.2 (11.2 to 22.8)	**-25.5 (-35.36 to -9.76)**	55.7 (39.8 to 75.3)	34.5 (23.7 to 48.5)	**-37.8 (-46.32 to -23.40)**
Brain and nervous system cancer	77.1 (65.9 to 89.2)	82.1 (69.7 to 96.0)	**6.5 (-1.19 to 13.55)**	146.5 (124.4 to 168.5)	127.8 (109.1 to 149.3)	**-12.8 (-18.77 to -6.93)**	45.1 (35.2 to 54.8)	49.8 (38.9 to 61.9)	**10.5 (0.55 to 20.86)**	176.9 (137.7 to 214.6)	157.2 (123.2 to 195.7)	**-11.1 (-18.94 to -2.81)**	32.0 (26.2 to 39.0)	32.3 (25.5 to 38.3)	1.0 (-8.02 to 10.86)	118.2 (95.6 to 142.8)	99.4 (79.3 to 119.0)	**-15.9 (-22.96 to -7.94)**
Thyroid cancer	6.7 (5.5 to 7.9)	5.7 (4.7 to 6.6)	**-15.6 (-28.80 to -0.73)**	10.6 (8.9 to 12.6)	7.4 (6.2 to 8.7)	**-30.2 (-41.26 to -18.78)**	2.3 (2.1 to 2.7)	2.5 (2.1 to 2.8)	8.3 (-22.78 to 24.35)	8.3 (7.6 to 9.7)	6.9 (5.8 to 7.9)	**-16.1 (-40.19 to -3.64)**	4.4 (3.4 to 5.3)	3.2 (2.2 to 4.0)	**-27.9 (-42.51 to -6.11)**	12.4 (9.6 to 15.0)	7.8 (5.5 to 9.8)	**-37.0 (-50.27 to -18.86)**
Mesothelioma	11.8 (9.8 to 20.9)	33.2 (18.1 to 39.9)	**200.1 (21.19 to 275.96)**	19.2 (16.1 to 33.6)	41.7 (23.4 to 50.0)	**130.3 (-2.47 to 186.96)**	8.4 (6.7 to 17.4)	27.7 (13.1 to 33.8)	**260.0 (19.21 to 364.63)**	29.8 (23.8 to 60.4)	72.9 (35.3 to 88.8)	**167.0 (-8.35 to 243.75)**	3.3 (2.9 to 5.3)	5.5 (3.9 to 7.7)	**67.2 (-0.50 to 101.65)**	10.1 (8.7 to 16.1)	13.7 (9.7 to 19.3)	36.5 (-16.05 to 64.79)
Hodgkin lymphoma	13.2 (10.4 to 16.8)	9.2 (7.2 to 12.0)	**-30.4 (-40.57 to -13.72)**	25.5 (19.9 to 32.7)	15.6 (12.2 to 20.3)	**-38.5 (-47.24 to -24.68)**	7.9 (5.2 to 11.1)	5.2 (3.8 to 7.3)	**-33.4 (-44.59 to -13.72)**	31.4 (20.7 to 44.0)	18.0 (13.2 to 25.1)	**-42.3 (-51.89 to -25.38)**	5.3 (3.9 to 6.8)	3.9 (2.7 to 5.1)	**-25.5 (-39.71 to -7.03)**	19.8 (14.5 to 25.3)	13.3 (9.2 to 17.3)	**-32.6 (-45.15 to -16.14)**
Non-Hodgkin lymphoma	74.8 (62.0 to 103.9)	87.4 (57.6 to 108.5)	18.0 (-32.08 to 36.62)	125.8 (103.8 to 174.2)	118.2 (77.9 to 149.0)	-5.2 (-44.75 to 11.21)	42.8 (33.7 to 66.8)	51.4 (25.7 to 64.2)	21.9 (-42.89 to 45.11)	159.0 (125.1 to 247.9)	147.3 (74.6 to 186.4)	-5.9 (-55.39 to 13.03)	32.0 (23.8 to 47.6)	36.0 (21.4 to 50.1)	13.7 (-32.38 to 35.33)	96.8 (71.4 to 142.2)	91.2 (54.7 to 128.4)	-5.2 (-42.44 to 13.46)
Multiple myeloma	37.6 (27.4 to 51.9)	45.9 (34.8 to 63.3)	**22.6 (3.99 to 37.06)**	57.6 (41.6 to 79.2)	56.6 (42.9 to 77.9)	-1.4 (-16.66 to 10.44)	20.0 (12.5 to 31.1)	25.9 (17.0 to 39.5)	**30.4 (-5.71 to 56.69)**	70.5 (44.0 to 110.2)	68.3 (45.1 to 104.5)	-2.3 (-28.70 to 16.62)	17.5 (12.0 to 26.2)	20.0 (13.8 to 29.7)	**14.3 (0.57 to 29.63)**	47.4 (32.4 to 70.4)	46.3 (31.8 to 68.0)	-2.4 (-14.55 to 10.68)
Leukemia	83.3 (80.6 to 86.2)	78.6 (69.4 to 85.5)	-5.6 (-17.42 to 3.10)	154.7 (149.1 to 160.0)	117.4 (103.0 to 128.0)	**-24.1 (-33.72 to -16.90)**	47.3 (45.8 to 48.9)	47.9 (40.8 to 52.5)	1.4 (-15.02 to 11.75)	188.6 (181.8 to 195.7)	148.0 (125.7 to 162.3)	**-21.5 (-34.03 to -13.60)**	36.0 (34.0 to 38.7)	30.7 (26.1 to 35.2)	**-14.7 (-35.30 to -1.92)**	126.5 (119.3 to 135.0)	89.4 (77.2 to 102.6)	**-29.2 (-45.63 to -18.26)**
Other neoplasms	90.5 (82.5 to 105.2)	105.8 (86.3 to 116.3)	17.1 (-3.90 to 29.69)	165.3 (150.7 to 191.4)	157.6 (129.1 to 173.6)	-4.5 (-21.26 to 5.54)	45.1 (39.7 to 57.3)	51.6 (38.0 to 59.0)	14.8 (-14.99 to 29.50)	179.1 (157.9 to 228.1)	162.3 (118.0 to 185.7)	-9.0 (-32.70 to 2.34)	45.4 (39.9 to 54.0)	54.2 (42.9 to 61.4)	**19.6 (-0.20 to 36.00)**	152.8 (135.6 to 180.5)	153.2 (122.1 to 173.1)	0.4 (-13.88 to 13.13)
**Cardiovascular diseases**	3,687.9 (3,472.0 to 3,803.8)	2,073.5 (1,929.9 to 2,248.8)	**-43.8 (-46.99 to -39.64)**	5,495.5 (5,187.1 to 5,667.2)	2,403.9 (2,233.5 to 2,601.7)	**-56.3 (-58.80 to -53.11)**	2,126.0 (2,067.5 to 2,180.9)	1,155.6 (1,068.2 to 1,242.1)	**-45.6 (-49.54 to -42.05)**	7,558.2 (7,347.8 to 7,752.3)	3,029.6 (2,800.5 to 3,260.1)	**-59.9 (-62.80 to -57.18)**	1,561.9 (1,367.0 to 1,646.3)	917.9 (835.3 to 1,082.8)	**-41.2 (-46.50 to -30.81)**	3,775.3 (3,282.3 to 3,999.6)	1,822.7 (1,649.9 to 2,168.3)	**-51.7 (-56.23 to -43.01)**
Rheumatic heart disease	37.3 (26.1 to 44.9)	17.4 (14.5 to 27.1)	**-52.9 (-60.80 to -16.07)**	59.1 (41.8 to 71.3)	22.0 (18.3 to 33.9)	**-62.3 (-68.49 to -34.46)**	11.6 (8.1 to 15.6)	5.7 (4.0 to 8.5)	**-50.6 (-59.31 to -34.46)**	42.1 (29.5 to 56.5)	16.0 (11.5 to 24.0)	**-61.7 (-68.12 to -49.79)**	25.8 (15.6 to 31.6)	11.7 (9.2 to 21.1)	**-53.4 (-63.97 to 1.98)**	72.8 (44.3 to 90.0)	27.2 (21.6 to 48.7)	**-61.6 (-70.28 to -17.14)**
Ischemic heart disease	2,314.5 (2,100.9 to 2,403.6)	1,044.6 (964.3 to 1,191.5)	**-54.8 (-57.95 to -48.31)**	3,479.6 (3,155.9 to 3,612.3)	1,220.0 (1,127.1 to 1,384.8)	**-64.9 (-67.37 to -59.90)**	1,468.6 (1,356.1 to 1,529.4)	649.7 (594.1 to 717.8)	**-55.7 (-59.10 to -50.40)**	5,200.6 (4,804.0 to 5,416.0)	1,705.9 (1,560.1 to 1,882.6)	**-67.2 (-69.64 to -63.15)**	845.9 (692.2 to 911.5)	394.9 (348.9 to 523.7)	**-53.2 (-58.54 to -36.10)**	2,040.5 (1,654.3 to 2,213.3)	774.4 (680.7 to 1,041.8)	**-61.9 (-66.45 to -47.86)**
Cerebrovascular disease	828.4 (735.5 to 913.9)	535.9 (475.1 to 607.0)	**-35.3 (-40.66 to -27.78)**	1,192.7 (1,062.7 to 1,320.1)	598.4 (529.1 to 673.2)	**-49.8 (-54.40 to -44.69)**	367.4 (316.8 to 438.5)	252.1 (198.7 to 299.0)	**-31.2 (-47.36 to -21.62)**	1,314.9 (1,134.9 to 1,572.3)	645.4 (508.4 to 767.3)	**-50.8 (-62.13 to -44.00)**	461.0 (368.4 to 514.9)	283.8 (249.7 to 357.4)	**-38.3 (-44.49 to -22.74)**	1,092.4 (882.1 to 1,224.4)	551.9 (479.6 to 691.6)	**-49.4 (-54.26 to -36.61)**
Ischemic stroke	479.6 (405.4 to 530.2)	302.6 (260.6 to 363.4)	**-36.7 (-44.52 to -20.29)**	655.2 (555.6 to 725.4)	318.9 (272.9 to 380.8)	**-51.2 (-57.38 to -38.82)**	210.6 (171.7 to 252.1)	142.3 (110.8 to 174.8)	**-32.1 (-47.69 to -14.84)**	748.3 (611.5 to 894.4)	352.3 (275.2 to 432.8)	**-52.7 (-63.40 to -41.05)**	269.1 (203.0 to 295.7)	160.3 (136.6 to 207.5)	**-40.0 (-47.97 to -15.70)**	580.5 (439.3 to 643.8)	286.6 (242.4 to 372.9)	**-50.3 (-56.84 to -30.27)**
Hemorrhagic stroke	348.7 (309.8 to 417.8)	233.3 (185.4 to 267.1)	**-33.0 (-46.29 to -25.41)**	537.4 (478.3 to 641.5)	279.5 (224.5 to 317.8)	**-47.9 (-57.72 to -42.35)**	156.8 (128.9 to 199.2)	109.8 (74.7 to 138.1)	**-29.4 (-54.71 to -14.39)**	566.5 (464.8 to 717.0)	293.1 (203.5 to 365.6)	**-47.9 (-65.83 to -37.51)**	191.9 (152.3 to 237.9)	123.4 (97.0 to 156.0)	**-35.6 (-47.41 to -21.83)**	512.0 (412.4 to 638.9)	265.4 (212.8 to 332.8)	**-48.1 (-56.69 to -38.40)**
Hypertensive heart disease	49.2 (42.4 to 60.9)	46.2 (36.3 to 55.2)	-5.8 (-26.12 to 8.12)	73.5 (63.4 to 91.0)	54.1 (42.4 to 65.5)	**-26.0 (-41.88 to -14.72)**	25.2 (19.9 to 33.3)	23.9 (17.0 to 32.0)	-4.1 (-33.26 to 24.05)	90.0 (71.5 to 119.0)	63.4 (45.1 to 84.7)	**-28.9 (-49.91 to -8.11)**	24.0 (19.8 to 30.5)	22.2 (16.4 to 27.2)	-6.8 (-27.12 to 12.61)	58.5 (48.6 to 74.6)	44.9 (33.5 to 55.0)	**-23.0 (-39.74 to -6.36)**
Cardiomyopathy and myocarditis	88.8 (75.4 to 102.9)	49.4 (40.4 to 61.0)	**-44.3 (-54.01 to -34.33)**	147.6 (125.9 to 172.6)	66.6 (54.1 to 81.2)	**-54.8 (-63.37 to -48.05)**	49.2 (38.5 to 59.8)	30.9 (22.8 to 40.5)	**-37.0 (-51.42 to -22.37)**	189.5 (149.7 to 230.2)	92.1 (67.8 to 119.6)	**-51.2 (-62.50 to -40.15)**	39.7 (31.6 to 48.3)	18.5 (14.1 to 23.5)	**-53.1 (-65.30 to -33.21)**	106.0 (84.2 to 131.6)	41.6 (31.2 to 52.5)	**-60.6 (-71.53 to -45.66)**
Atrial fibrillation and flutter	26.7 (22.5 to 31.5)	44.5 (37.0 to 50.7)	**67.3 (44.48 to 89.09)**	36.2 (30.5 to 43.0)	45.2 (37.6 to 51.6)	**25.2 (8.60 to 40.70)**	11.7 (9.4 to 14.6)	17.1 (13.7 to 19.9)	**46.8 (22.02 to 70.88)**	41.4 (33.3 to 51.3)	41.7 (33.4 to 48.6)	1.2 (-15.87 to 17.35)	15.0 (12.8 to 17.5)	27.4 (22.0 to 31.4)	**83.6 (53.40 to 111.28)**	31.8 (27.0 to 37.4)	47.0 (38.2 to 54.1)	**48.1 (24.33 to 69.74)**
Aortic aneurysm	117.6 (101.6 to 139.2)	78.8 (66.4 to 97.0)	**-33.0 (-40.75 to -25.24)**	168.8 (146.3 to 198.3)	89.7 (75.5 to 110.0)	**-46.8 (-53.03 to -40.66)**	81.7 (69.3 to 96.9)	49.2 (40.2 to 56.6)	**-39.7 (-49.58 to -32.54)**	281.4 (238.3 to 333.8)	125.3 (102.7 to 144.4)	**-55.4 (-62.55 to -49.90)**	35.9 (23.1 to 53.4)	29.6 (19.5 to 46.6)	**-17.3 (-32.86 to -1.01)**	84.2 (54.1 to 125.0)	58.6 (38.9 to 92.7)	**-30.2 (-43.12 to -15.65)**
Peripheral vascular disease	27.6 (25.0 to 30.6)	21.6 (18.7 to 24.9)	**-21.7 (-30.69 to -12.20)**	37.7 (34.2 to 41.6)	22.5 (19.6 to 25.9)	**-40.2 (-47.20 to -32.73)**	14.7 (13.3 to 16.5)	10.2 (8.5 to 12.2)	**-30.5 (-43.31 to -18.41)**	52.2 (47.0 to 58.2)	25.1 (20.9 to 29.9)	**-51.8 (-60.66 to -43.47)**	12.9 (10.8 to 15.0)	11.4 (9.3 to 13.6)	**-11.6 (-22.87 to 0.57)**	27.4 (23.0 to 32.2)	19.9 (16.3 to 23.8)	**-27.5 (-36.52 to -17.46)**
Endocarditis	10.5 (7.9 to 13.2)	10.1 (8.0 to 13.9)	-3.9 (-17.51 to 23.24)	17.0 (12.9 to 21.5)	13.3 (10.5 to 18.1)	**-21.6 (-32.24 to -1.67)**	5.3 (3.8 to 7.4)	6.2 (4.3 to 9.1)	18.7 (-10.65 to 54.65)	19.6 (14.0 to 27.3)	17.8 (12.4 to 25.3)	-8.8 (-30.54 to 15.70)	5.2 (3.3 to 7.2)	3.8 (2.7 to 6.3)	-26.3 (-41.62 to 10.51)	14.8 (9.4 to 20.4)	9.2 (6.5 to 15.7)	**-37.2 (-50.43 to -3.60)**
Other cardiovascular and circulatory diseases	187.3 (149.9 to 243.1)	225.1 (166.0 to 281.4)	21.6 (-24.14 to 51.13)	283.3 (226.0 to 370.6)	272.0 (200.0 to 338.1)	-2.9 (-39.52 to 20.86)	90.6 (73.1 to 123.0)	110.5 (77.9 to 137.7)	23.4 (-26.33 to 55.29)	326.5 (263.2 to 441.3)	296.9 (208.6 to 369.2)	-8.0 (-45.24 to 15.65)	96.6 (75.8 to 131.7)	114.6 (82.1 to 147.2)	20.1 (-23.39 to 51.80)	246.8 (194.1 to 334.1)	247.9 (178.4 to 315.4)	1.8 (-35.50 to 29.16)
**Chronic respiratory diseases**	748.6 (655.5 to 861.0)	717.9 (602.4 to 842.6)	-4.1 (-12.48 to 2.49)	1,179.4 (1,021.4 to 1,371.5)	916.0 (759.5 to 1,088.9)	**-22.4 (-29.14 to -17.06)**	427.0 (371.6 to 488.0)	387.6 (326.9 to 470.7)	-9.2 (-17.76 to 5.14)	1,543.1 (1,341.5 to 1,772.3)	1,058.5 (887.5 to 1,292.3)	**-31.4 (-37.83 to -21.21)**	321.6 (270.6 to 384.1)	330.4 (263.3 to 397.6)	2.8 (-13.77 to 11.75)	937.0 (775.0 to 1,135.2)	798.2 (634.0 to 976.1)	**-14.7 (-27.75 to -7.13)**
Chronic obstructive pulmonary disease	544.8 (478.9 to 616.5)	527.2 (448.8 to 620.4)	-3.2 (-10.98 to 5.80)	823.6 (721.7 to 941.2)	643.9 (540.5 to 768.5)	**-21.9 (-28.42 to -15.25)**	325.5 (279.6 to 372.3)	290.6 (239.6 to 362.2)	-10.7 (-20.80 to 7.02)	1,156.9 (994.0 to 1,326.8)	769.6 (629.5 to 952.2)	**-33.5 (-41.23 to -20.62)**	219.3 (182.1 to 262.5)	236.6 (191.7 to 285.3)	8.0 (-9.18 to 18.60)	599.1 (488.5 to 723.9)	539.4 (431.9 to 663.1)	**-9.9 (-23.35 to -0.57)**

Pneumoconiosis	7.4 (5.3 to 9.9)	7.2 (4.8 to 10.3)	-2.4 (-25.44 to 33.80)	10.6 (7.7 to 14.2)	8.0 (5.5 to 11.3)	**-24.2 (-41.25 to 0.50)**	6.8 (4.7 to 9.4)	6.8 (4.4 to 9.9)	0.7 (-24.64 to 39.65)	23.9 (16.6 to 33.1)	16.7 (11.0 to 24.2)	**-29.7 (-47.20 to -2.94)**	0.6 (0.5 to 0.8)	0.4 (0.3 to 0.5)	**-35.5 (-49.12 to -23.80)**	1.8 (1.4 to 2.4)	1.1 (0.9 to 1.4)	**-37.5 (-49.90 to -26.38)**
Silicosis	0.6 (0.4 to 0.8)	0.3 (0.2 to 0.4)	**-53.6 (-63.74 to -38.77)**	0.9 (0.7 to 1.2)	0.4 (0.3 to 0.5)	**-58.1 (-66.47 to -48.02)**	0.5 (0.4 to 0.7)	0.2 (0.2 to 0.3)	**-57.9 (-68.26 to -41.45)**	2.0 (1.4 to 2.6)	0.6 (0.4 to 0.9)	**-67.0 (-74.69 to -55.36)**	0.1 (0.0 to 0.1)	<0.05 (0.0 to 0.1)	-9.9 (-26.46 to 4.25)	0.2 (0.2 to 0.3)	0.2 (0.1 to 0.2)	-9.7 (-28.17 to 5.69)
Asbestosis	1.5 (1.0 to 2.1)	3.2 (2.2 to 4.9)	**118.7 (67.43 to 228.18)**	2.2 (1.5 to 3.0)	3.6 (2.4 to 5.4)	**68.6 (31.56 to 147.59)**	1.4 (1.0 to 2.0)	3.2 (2.1 to 4.8)	**122.1 (68.90 to 236.11)**	5.1 (3.4 to 7.0)	7.9 (5.2 to 11.8)	**56.6 (19.21 to 135.32)**	0.1 (0.0 to 0.1)	0.1 (0.0 to 0.1)	28.5 (-11.36 to 58.57)	0.2 (0.2 to 0.3)	0.2 (0.2 to 0.3)	12.1 (-17.73 to 34.27)
Coal workers pneumoconiosis	2.6 (1.8 to 3.6)	2.3 (1.5 to 3.4)	-11.8 (-35.99 to 21.00)	3.7 (2.6 to 5.0)	2.5 (1.6 to 3.6)	**-32.2 (-50.10 to -10.09)**	2.6 (1.8 to 3.6)	2.3 (1.5 to 3.4)	-11.1 (-35.96 to 22.99)	9.1 (6.2 to 12.6)	5.6 (3.6 to 8.2)	**-38.7 (-55.46 to -16.48)**	0.1 (0.0 to 0.1)	<0.05 (0.0 to <0.05)	**-39.5 (-55.48 to -25.56)**	0.2 (0.2 to 0.3)	0.2 (0.1 to 0.2)	**-29.2 (-47.25 to -13.33)**
Other pneumoconiosis	2.6 (2.0 to 3.4)	1.3 (0.9 to 1.8)	**-49.8 (-61.99 to -37.82)**	3.8 (2.9 to 5.0)	1.5 (1.1 to 2.0)	**-60.5 (-69.51 to -52.34)**	2.2 (1.5 to 3.0)	1.1 (0.7 to 1.5)	**-50.5 (-65.09 to -36.67)**	7.8 (5.5 to 10.9)	2.7 (1.7 to 3.8)	**-65.4 (-75.03 to -56.39)**	0.4 (0.3 to 0.6)	0.2 (0.2 to 0.3)	**-45.7 (-57.90 to -34.83)**	1.2 (0.9 to 1.6)	0.6 (0.4 to 0.8)	**-52.1 (-61.42 to -43.48)**
Asthma	139.3 (103.0 to 182.9)	135.4 (94.9 to 182.0)	-3.0 (-11.35 to 5.52)	254.2 (187.5 to 333.5)	205.0 (143.2 to 276.9)	**-19.5 (-26.45 to -11.92)**	63.9 (47.5 to 83.8)	61.0 (42.1 to 83.0)	-4.7 (-16.76 to 8.85)	247.0 (184.0 to 324.2)	194.9 (134.9 to 265.6)	**-21.2 (-31.60 to -9.92)**	75.4 (54.7 to 100.2)	74.4 (52.2 to 99.4)	-1.3 (-14.49 to 11.15)	261.8 (189.8 to 349.2)	213.6 (149.4 to 288.0)	**-18.4 (-29.99 to -7.50)**
Interstitial lung disease and pulmonary sarcoidosis	21.6 (15.5 to 33.6)	44.1 (21.3 to 63.7)	128.4 (-16.90 to 260.62)	32.9 (23.8 to 50.9)	53.1 (26.1 to 76.0)	79.6 (-33.07 to 180.68)	14.4 (10.8 to 24.4)	26.9 (12.0 to 37.7)	109.8 (-25.14 to 238.18)	50.8 (38.4 to 85.7)	70.0 (31.5 to 97.7)	54.1 (-44.17 to 147.37)	7.2 (4.2 to 14.3)	17.2 (6.2 to 29.2)	176.3 (-11.14 to 356.43)	20.2 (12.0 to 39.5)	39.0 (14.7 to 65.4)	121.0 (-25.59 to 262.55)
Other chronic respiratory diseases	35.6 (28.4 to 45.4)	4.1 (3.3 to 5.2)	**-88.4 (-89.94 to -86.77)**	58.1 (46.7 to 73.8)	6.0 (4.9 to 7.5)	**-89.6 (-91.12 to -88.10)**	16.5 (14.2 to 19.6)	2.3 (1.9 to 2.9)	**-86.0 (-87.87 to -83.83)**	64.4 (55.9 to 76.7)	7.2 (5.9 to 8.9)	**-88.8 (-90.34 to -87.09)**	19.1 (13.0 to 28.0)	1.8 (1.2 to 2.6)	**-90.4 (-92.38 to -88.71)**	54.0 (37.3 to 78.1)	5.0 (3.5 to 6.9)	**-90.8 (-92.56 to -89.02)**
**Cirrhosis**	99.7 (96.9 to 102.8)	181.0 (169.0 to 192.5)	**81.5 (69.31 to 94.05)**	178.1 (173.0 to 183.3)	267.2 (249.4 to 284.6)	**50.0 (40.15 to 60.25)**	56.8 (55.1 to 58.2)	116.8 (107.4 to 126.2)	**105.7 (88.95 to 123.35)**	214.5 (208.1 to 219.9)	353.7 (325.0 to 381.9)	**64.9 (51.51 to 79.25)**	42.9 (41.1 to 45.9)	64.2 (57.9 to 69.9)	**49.6 (35.62 to 63.72)**	144.0 (137.4 to 152.7)	183.5 (165.4 to 200.0)	**27.5 (15.79 to 39.48)**
Cirrhosis due to hepatitis B	10.5 (9.0 to 12.6)	18.8 (15.0 to 22.5)	**80.3 (38.82 to 122.52)**	18.6 (15.8 to 22.4)	27.3 (21.8 to 32.8)	**48.2 (13.61 to 82.92)**	5.9 (4.8 to 7.1)	10.3 (8.2 to 13.1)	**76.5 (33.58 to 122.29)**	22.1 (18.0 to 26.8)	31.0 (24.6 to 39.5)	**41.5 (7.37 to 78.24)**	4.6 (3.5 to 6.2)	8.5 (6.1 to 10.9)	**87.7 (22.95 to 165.04)**	15.3 (11.5 to 21.2)	23.7 (16.9 to 30.5)	**57.5 (2.27 to 123.70)**
Cirrhosis due to hepatitis C	37.7 (33.6 to 42.5)	85.0 (75.8 to 95.3)	**126.0 (94.11 to 159.68)**	67.8 (59.9 to 76.8)	125.4 (111.9 to 140.6)	**85.4 (58.96 to 113.75)**	19.3 (15.5 to 23.1)	49.4 (41.9 to 57.2)	**158.5 (105.04 to 219.41)**	72.9 (58.3 to 87.5)	149.5 (126.9 to 172.7)	**106.9 (64.89 to 155.94)**	18.5 (15.3 to 21.8)	35.7 (30.4 to 41.2)	**94.8 (49.98 to 140.20)**	63.1 (52.5 to 75.1)	101.9 (86.6 to 118.4)	**62.9 (24.28 to 100.68)**
Cirrhosis due to alcohol use	41.7 (36.9 to 46.1)	53.2 (44.4 to 61.1)	**27.9 (7.35 to 51.10)**	72.8 (63.7 to 81.0)	76.3 (63.4 to 88.1)	5.2 (-11.72 to 24.71)	26.4 (22.1 to 29.7)	43.1 (35.5 to 49.8)	**63.8 (33.66 to 103.63)**	98.8 (82.3 to 111.3)	127.6 (105.1 to 148.0)	**29.8 (5.77 to 60.98)**	15.3 (12.8 to 17.8)	10.1 (7.6 to 12.8)	**-33.5 (-50.86 to -16.10)**	48.4 (39.6 to 57.0)	27.0 (20.0 to 34.8)	**-43.8 (-59.80 to -28.28)**
Cirrhosis due to other causes	9.9 (8.6 to 11.7)	24.1 (20.6 to 27.7)	**144.9 (94.93 to 193.64)**	19.0 (16.6 to 22.3)	38.1 (33.0 to 43.6)	**102.2 (62.12 to 140.24)**	5.3 (4.1 to 6.7)	14.1 (11.2 to 17.6)	**171.9 (100.97 to 283.63)**	20.7 (16.3 to 26.4)	45.5 (36.3 to 56.3)	**122.3 (65.98 to 209.54)**	4.6 (3.5 to 5.9)	10.0 (7.8 to 12.5)	**119.3 (49.02 to 189.79)**	17.2 (13.1 to 21.6)	30.9 (24.6 to 38.5)	**82.1 (25.63 to 137.34)**
**Digestive diseases**	282.4 (253.6 to 317.4)	301.5 (260.8 to 347.6)	6.8 (-3.97 to 16.83)	445.4 (395.8 to 502.0)	386.6 (329.8 to 447.4)	**-13.2 (-22.06 to -6.78)**	126.0 (113.5 to 141.8)	142.1 (121.5 to 169.4)	**12.9 (-0.26 to 31.22)**	465.6 (418.3 to 526.2)	399.2 (340.0 to 474.5)	**-14.2 (-24.36 to -2.58)**	156.5 (138.5 to 180.1)	159.4 (136.6 to 182.7)	1.9 (-11.15 to 9.85)	427.6 (371.2 to 495.1)	373.1 (314.1 to 436.8)	**-12.7 (-23.92 to -6.27)**
Peptic ulcer disease	76.1 (65.7 to 86.9)	43.7 (36.0 to 55.8)	**-42.5 (-51.24 to -26.38)**	111.9 (96.8 to 128.5)	50.1 (40.9 to 62.8)	**-55.2 (-62.19 to -44.11)**	35.0 (29.7 to 41.0)	22.0 (16.3 to 32.0)	**-37.0 (-52.55 to -5.23)**	126.1 (106.9 to 147.9)	57.1 (42.3 to 81.5)	**-54.6 (-65.44 to -33.27)**	41.0 (33.4 to 49.8)	21.8 (17.1 to 28.1)	**-46.8 (-54.98 to -30.84)**	100.4 (82.1 to 122.2)	43.5 (34.2 to 55.0)	**-56.6 (-63.63 to -45.52)**
Gastritis and duodenitis	6.4 (4.8 to 8.3)	9.6 (7.3 to 12.4)	**52.0 (29.89 to 68.72)**	11.1 (8.3 to 14.5)	13.7 (10.3 to 17.7)	**23.0 (5.47 to 36.52)**	1.8 (1.4 to 2.7)	3.2 (2.0 to 4.2)	**76.0 (23.63 to 125.22)**	7.1 (5.4 to 10.5)	9.4 (6.0 to 12.4)	33.4 (-3.94 to 70.46)	4.5 (3.2 to 6.1)	6.5 (4.6 to 8.6)	**42.7 (27.57 to 53.04)**	14.8 (10.6 to 19.8)	17.8 (12.7 to 23.5)	**20.4 (7.84 to 29.87)**
Appendicitis	5.6 (4.3 to 6.5)	4.5 (3.3 to 6.6)	-17.6 (-43.90 to 24.52)	10.3 (8.0 to 12.0)	6.6 (5.0 to 9.2)	**-34.9 (-52.51 to -9.04)**	2.7 (1.9 to 3.4)	2.6 (1.6 to 4.3)	-1.6 (-43.04 to 51.33)	10.9 (7.7 to 13.8)	8.0 (5.2 to 12.5)	-25.7 (-53.85 to 8.54)	2.8 (2.0 to 3.6)	1.9 (1.3 to 3.1)	**-32.2 (-52.13 to 5.51)**	9.7 (7.0 to 12.0)	5.5 (4.0 to 8.3)	**-43.3 (-57.86 to -18.15)**
Paralytic ileus and intestinal obstruction	16.6 (12.2 to 25.0)	23.9 (13.0 to 30.7)	46.5 (-8.83 to 79.28)	25.1 (18.5 to 37.5)	28.3 (15.4 to 35.9)	14.5 (-27.74 to 39.95)	7.3 (4.8 to 12.6)	11.3 (4.7 to 14.9)	59.3 (-21.50 to 114.74)	27.4 (18.0 to 46.9)	30.4 (12.8 to 39.8)	13.9 (-41.79 to 52.43)	9.2 (5.3 to 15.5)	12.6 (6.3 to 18.6)	**37.3 (-4.55 to 64.06)**	23.3 (13.4 to 39.1)	26.3 (13.3 to 38.4)	14.2 (-20.79 to 35.00)
Inguinal, femoral, and abdominal hernia	13.3 (9.9 to 18.5)	13.5 (7.9 to 26.3)	2.0 (-36.21 to 123.54)	19.6 (14.7 to 27.1)	15.3 (9.1 to 28.8)	-21.2 (-50.19 to 64.83)	6.9 (4.8 to 10.0)	7.6 (4.2 to 17.5)	12.5 (-34.20 to 176.97)	25.4 (17.5 to 36.3)	19.7 (10.9 to 43.4)	-21.0 (-52.71 to 87.05)	6.4 (4.3 to 10.0)	5.8 (3.1 to 11.6)	-6.8 (-52.87 to 87.08)	15.5 (10.6 to 24.1)	11.6 (6.2 to 22.2)	-23.1 (-60.02 to 48.87)
Inflammatory bowel disease	54.3 (40.9 to 69.0)	72.5 (53.2 to 94.0)	**33.6 (21.41 to 45.11)**	95.9 (71.7 to 122.7)	106.5 (77.3 to 139.1)	**11.0 (0.87 to 20.51)**	25.4 (18.8 to 33.0)	36.1 (26.3 to 47.3)	**42.5 (26.83 to 62.62)**	97.3 (71.9 to 126.6)	111.0 (80.2 to 146.2)	**14.3 (1.83 to 28.80)**	28.9 (21.8 to 36.8)	36.4 (26.8 to 47.0)	**26.0 (13.15 to 40.91)**	94.5 (70.7 to 120.7)	102.0 (74.0 to 133.4)	8.0 (-3.32 to 21.47)
Vascular intestinal disorders	23.0 (14.9 to 35.1)	28.9 (17.7 to 44.3)	27.5 (-11.07 to 78.45)	33.9 (21.9 to 51.9)	33.4 (20.8 to 51.0)	-0.1 (-29.56 to 37.34)	10.5 (4.7 to 20.2)	12.1 (4.8 to 24.2)	18.6 (-23.03 to 133.41)	37.2 (16.5 to 71.5)	31.4 (12.4 to 62.6)	-13.3 (-43.11 to 64.83)	12.5 (7.5 to 23.4)	16.8 (7.2 to 26.4)	37.7 (-16.69 to 72.54)	31.0 (18.9 to 57.8)	34.7 (15.3 to 56.0)	14.6 (-30.41 to 42.73)
Gallbladder and biliary diseases	17.6 (15.0 to 21.5)	26.9 (20.6 to 31.8)	**53.1 (14.96 to 79.98)**	26.8 (22.8 to 32.5)	31.8 (24.4 to 37.9)	19.2 (-10.09 to 40.00)	7.0 (5.8 to 8.8)	11.9 (8.4 to 14.2)	**71.6 (6.78 to 108.86)**	25.2 (20.9 to 31.9)	31.2 (22.3 to 37.1)	24.6 (-20.09 to 50.50)	10.7 (8.8 to 13.9)	15.0 (11.1 to 18.4)	**41.4 (0.35 to 68.94)**	28.4 (23.3 to 36.4)	32.3 (24.0 to 40.1)	14.6 (-17.30 to 36.73)
Pancreatitis	20.0 (16.2 to 25.2)	23.1 (18.1 to 29.4)	15.8 (-3.97 to 36.40)	33.8 (27.5 to 42.3)	32.0 (25.2 to 39.6)	-5.2 (-20.93 to 9.72)	11.1 (8.2 to 14.8)	13.8 (9.8 to 17.6)	**25.0 (-0.05 to 54.03)**	41.7 (30.8 to 55.4)	41.1 (29.4 to 51.8)	-1.0 (-20.36 to 19.56)	8.9 (6.6 to 12.2)	9.3 (6.6 to 12.8)	4.8 (-17.22 to 35.48)	26.3 (19.8 to 35.7)	23.2 (17.0 to 31.3)	-11.5 (-29.54 to 9.76)
Other digestive diseases	49.7 (41.2 to 58.4)	55.0 (44.1 to 66.3)	10.9 (-8.88 to 33.45)	76.9 (63.2 to 90.9)	69.0 (54.2 to 83.9)	-10.1 (-27.20 to 8.28)	18.2 (14.3 to 23.1)	21.5 (14.5 to 27.9)	18.9 (-20.69 to 55.28)	67.3 (53.2 to 85.2)	60.1 (40.4 to 77.8)	-10.2 (-40.53 to 16.50)	31.5 (25.2 to 37.3)	33.5 (26.7 to 41.7)	6.5 (-7.39 to 27.89)	83.7 (66.3 to 100.2)	76.2 (60.0 to 95.9)	-8.8 (-21.08 to 10.24)
**Neurological disorders**	921.1 (755.8 to 1,103.9)	1,126.4 (923.0 to 1,348.6)	**22.3 (15.58 to 29.16)**	1,549.6 (1,236.1 to 1,901.0)	1,542.7 (1,226.9 to 1,903.6)	-0.5 (-6.17 to 5.39)	358.1 (301.0 to 420.5)	463.5 (391.9 to 542.7)	**29.5 (21.00 to 38.11)**	1,418.4 (1,188.8 to 1,668.2)	1,357.6 (1,124.0 to 1,623.2)	-4.3 (-10.79 to 2.35)	562.9 (452.0 to 682.6)	662.9 (527.9 to 806.4)	**17.8 (9.88 to 26.61)**	1,707.9 (1,316.3 to 2,152.8)	1,733.6 (1,322.2 to 2,189.6)	1.5 (-5.21 to 9.67)
Alzheimer disease and other dementias	350.3 (314.2 to 390.7)	463.4 (412.8 to 514.6)	**32.4 (19.79 to 43.68)**	452.9 (406.5 to 504.7)	442.4 (394.3 to 491.0)	-2.2 (-11.61 to 5.78)	127.4 (114.5 to 140.1)	195.0 (174.8 to 216.0)	**53.3 (38.57 to 71.78)**	485.5 (436.8 to 534.3)	455.9 (409.7 to 504.5)	-6.0 (-14.89 to 5.43)	222.8 (193.5 to 256.9)	268.4 (230.0 to 307.1)	**20.7 (3.87 to 36.39)**	436.1 (378.9 to 500.6)	431.4 (370.4 to 493.9)	-0.9 (-14.35 to 12.00)
Parkinson disease	45.4 (38.6 to 50.8)	50.2 (42.5 to 57.2)	**10.6 (-0.65 to 19.49)**	60.8 (52.0 to 67.9)	53.3 (45.0 to 61.0)	**-12.3 (-21.75 to -5.17)**	25.0 (21.0 to 27.0)	30.3 (25.0 to 34.4)	**21.6 (6.80 to 34.07)**	88.4 (74.2 to 95.6)	73.7 (60.4 to 83.7)	**-16.5 (-26.86 to -7.93)**	20.4 (15.3 to 23.2)	19.9 (14.5 to 23.6)	-2.7 (-14.45 to 9.26)	44.3 (33.5 to 50.7)	37.3 (28.1 to 44.6)	**-15.8 (-25.63 to -5.46)**
Epilepsy	95.2 (71.2 to 122.5)	91.9 (66.2 to 121.2)	-2.7 (-26.61 to 25.83)	197.7 (148.2 to 254.8)	169.0 (122.2 to 223.0)	-13.9 (-34.67 to 11.17)	52.6 (39.6 to 67.7)	50.7 (37.4 to 66.1)	-2.9 (-25.69 to 24.09)	223.0 (167.9 to 287.1)	188.6 (138.7 to 247.1)	-14.8 (-34.58 to 8.85)	42.6 (31.5 to 55.7)	41.2 (28.9 to 54.8)	-2.5 (-28.21 to 29.35)	172.9 (127.3 to 226.2)	149.5 (105.4 to 198.9)	-12.8 (-35.51 to 15.36)
Multiple sclerosis	35.4 (28.6 to 47.6)	49.1 (38.7 to 60.0)	**39.3 (12.51 to 56.32)**	65.6 (52.8 to 87.5)	75.4 (59.7 to 92.6)	**15.5 (-6.10 to 29.77)**	12.7 (9.1 to 19.1)	17.3 (11.9 to 23.4)	**37.5 (-7.75 to 60.65)**	48.6 (35.0 to 72.5)	54.1 (37.5 to 72.8)	12.7 (-23.62 to 31.38)	22.7 (17.9 to 31.1)	31.8 (24.5 to 39.9)	**40.9 (16.68 to 63.09)**	82.0 (64.6 to 111.6)	96.1 (73.4 to 120.5)	**17.8 (-1.39 to 36.86)**
Migraine	241.6 (144.9 to 357.6)	265.2 (161.2 to 387.2)	**10.0 (-0.99 to 21.29)**	491.7 (295.6 to 727.7)	481.3 (291.2 to 701.2)	-1.9 (-11.81 to 8.15)	76.5 (46.0 to 115.5)	84.3 (49.7 to 124.3)	10.5 (-3.71 to 26.85)	317.0 (189.5 to 478.0)	309.9 (183.1 to 456.5)	-2.0 (-15.11 to 12.98)	165.1 (97.5 to 242.4)	180.9 (109.2 to 263.3)	9.9 (-3.81 to 28.54)	664.5 (393.1 to 975.1)	651.8 (395.0 to 955.6)	-1.6 (-14.02 to 14.95)
Tension-type headache	15.5 (7.4 to 27.5)	21.7 (10.6 to 37.9)	**40.9 (25.33 to 68.15)**	30.6 (14.6 to 54.5)	38.3 (18.9 to 66.6)	**26.0 (11.68 to 49.91)**	5.8 (2.8 to 10.4)	9.4 (4.7 to 16.4)	**62.8 (40.64 to 100.73)**	23.7 (11.4 to 42.1)	33.8 (16.6 to 58.8)	**44.1 (24.43 to 77.74)**	9.6 (4.5 to 17.6)	12.2 (5.9 to 21.4)	**28.3 (8.18 to 56.02)**	37.3 (17.6 to 67.4)	42.7 (20.8 to 74.8)	15.8 (-3.34 to 41.35)
Medication overuse headache	54.4 (32.3 to 81.9)	94.8 (54.9 to 144.8)	**74.6 (57.01 to 93.91)**	104.5 (62.0 to 157.5)	156.6 (90.3 to 240.0)	**50.2 (34.17 to 66.48)**	14.8 (8.6 to 22.2)	27.5 (16.2 to 42.8)	**87.0 (60.83 to 111.66)**	58.5 (34.0 to 88.1)	91.8 (53.9 to 143.3)	**57.2 (35.81 to 78.30)**	39.6 (23.3 to 60.2)	67.3 (38.8 to 103.2)	**70.3 (49.42 to 95.66)**	149.1 (87.4 to 226.8)	220.5 (126.6 to 338.5)	**48.4 (30.14 to 70.61)**
Other neurological disorders	83.3 (72.2 to 93.5)	90.1 (77.0 to 103.0)	**8.2 (-0.28 to 16.92)**	145.9 (126.7 to 162.9)	126.4 (109.6 to 142.5)	**-13.3 (-20.70 to -6.13)**	43.3 (32.7 to 51.0)	48.8 (38.6 to 59.0)	**13.0 (-1.92 to 25.88)**	173.7 (131.6 to 205.6)	149.6 (120.0 to 182.7)	**-13.7 (-25.76 to -3.36)**	40.1 (35.8 to 45.0)	41.3 (35.5 to 47.6)	3.2 (-9.21 to 14.56)	121.6 (111.2 to 134.7)	104.2 (91.6 to 117.5)	**-14.2 (-24.47 to -4.52)**
**Mental and substance use disorders**	1,202.0 (871.3 to 1,596.3)	1,359.3 (997.6 to 1,780.2)	**13.2 (10.13 to 15.74)**	2,437.1 (1,767.0 to 3,239.1)	2,479.2 (1,817.9 to 3,290.1)	1.8 (-0.66 to 3.99)	596.0 (436.7 to 777.8)	696.2 (522.4 to 896.5)	**17.0 (9.71 to 20.92)**	2,472.0 (1,810.8 to 3,236.4)	2,585.1 (1,939.0 to 3,332.6)	**4.7 (-1.48 to 8.29)**	606.0 (433.3 to 822.0)	663.1 (472.9 to 891.0)	**9.5 (7.17 to 12.19)**	2,395.4 (1,699.8 to 3,212.0)	2,369.6 (1,690.6 to 3,186.4)	-1.0 (-2.75 to 1.05)
Schizophrenia	90.0 (65.0 to 111.1)	102.2 (73.7 to 125.8)	**13.5 (10.14 to 17.20)**	174.1 (125.0 to 215.9)	170.7 (122.2 to 211.3)	-2.0 (-5.08 to 1.15)	49.5 (35.8 to 61.2)	56.8 (40.8 to 70.3)	**14.7 (9.66 to 20.07)**	195.9 (140.9 to 243.1)	193.5 (137.6 to 240.9)	-1.2 (-5.55 to 3.40)	40.5 (29.2 to 49.6)	45.4 (32.9 to 55.8)	**12.2 (7.27 to 16.82)**	152.2 (108.9 to 187.2)	148.0 (106.8 to 182.6)	-2.8 (-7.17 to 1.42)
Alcohol use disorders	71.1 (51.4 to 97.2)	92.9 (68.0 to 124.5)	**30.9 (13.68 to 39.08)**	143.9 (103.8 to 196.7)	169.7 (123.3 to 228.7)	**18.1 (3.50 to 24.72)**	51.0 (36.3 to 70.3)	68.0 (48.6 to 91.7)	**33.8 (10.97 to 43.80)**	208.0 (148.0 to 286.1)	250.4 (177.5 to 338.3)	**20.7 (1.44 to 28.42)**	20.1 (14.6 to 27.7)	24.9 (17.8 to 33.7)	**24.1 (10.67 to 36.70)**	80.2 (57.9 to 110.7)	88.8 (63.2 to 120.0)	**11.0 (-1.45 to 21.06)**
Drug use disorders	160.1 (122.6 to 205.0)	201.2 (157.3 to 246.5)	**26.2 (5.23 to 37.36)**	331.9 (254.5 to 424.5)	390.6 (305.5 to 478.1)	**18.2 (-1.56 to 28.47)**	111.7 (85.2 to 143.0)	147.8 (114.9 to 180.1)	**33.1 (1.07 to 47.59)**	461.6 (352.7 to 591.1)	569.6 (443.5 to 695.7)	**24.2 (-5.34 to 37.84)**	48.4 (36.5 to 61.3)	53.4 (41.1 to 66.4)	**10.6 (3.26 to 18.45)**	202.3 (152.5 to 256.8)	211.1 (162.5 to 263.1)	4.6 (-2.29 to 11.90)
Opioid use disorders	61.3 (42.3 to 86.4)	76.3 (57.7 to 101.8)	**25.6 (5.87 to 42.48)**	124.4 (85.7 to 176.0)	141.7 (106.8 to 189.9)	**15.0 (-3.68 to 31.11)**	47.8 (33.2 to 67.0)	60.7 (45.2 to 80.0)	**28.2 (1.86 to 47.55)**	194.3 (134.5 to 272.6)	225.7 (168.3 to 299.1)	**17.3 (-7.46 to 35.62)**	13.5 (9.2 to 19.1)	15.6 (11.3 to 21.4)	**16.4 (5.17 to 28.23)**	54.8 (37.2 to 77.8)	58.1 (42.1 to 80.3)	6.6 (-3.64 to 17.55)
Cocaine use disorders	18.4 (12.6 to 25.4)	20.9 (14.6 to 28.5)	**13.4 (6.65 to 19.38)**	38.7 (26.4 to 53.3)	41.8 (29.2 to 57.2)	**8.3 (1.96 to 14.08)**	12.2 (8.3 to 16.8)	14.1 (9.9 to 19.3)	**16.0 (5.88 to 24.55)**	51.0 (34.8 to 70.4)	56.3 (39.4 to 76.8)	**10.6 (0.95 to 18.88)**	6.2 (4.3 to 8.6)	6.8 (4.7 to 9.3)	**8.5 (1.04 to 15.71)**	26.2 (18.0 to 36.2)	27.2 (18.7 to 37.5)	3.8 (-3.37 to 10.60)
Amphetamine use disorders	19.8 (13.0 to 28.3)	20.3 (13.5 to 28.3)	2.6 (-3.92 to 8.13)	41.7 (27.2 to 59.3)	40.9 (27.1 to 57.2)	-1.6 (-7.73 to 3.72)	11.7 (7.6 to 16.5)	12.6 (8.4 to 17.6)	**7.7 (-2.68 to 15.77)**	49.0 (31.6 to 68.9)	49.9 (33.3 to 69.9)	2.2 (-7.63 to 9.63)	8.1 (5.3 to 11.6)	7.7 (5.1 to 10.9)	-4.8 (-11.08 to 1.41)	34.4 (22.5 to 49.2)	31.9 (20.9 to 45.2)	**-7.1 (-13.35 to -0.94)**
Cannabis use disorders	5.3 (3.6 to 7.7)	5.3 (3.5 to 7.6)	-1.5 (-6.97 to 4.02)	11.8 (8.0 to 16.9)	11.4 (7.6 to 16.4)	-4.0 (-9.79 to 1.75)	3.3 (2.3 to 4.8)	3.3 (2.2 to 4.8)	-0.9 (-6.61 to 5.48)	14.6 (9.9 to 21.1)	14.1 (9.4 to 20.3)	-3.7 (-9.49 to 2.80)	2.0 (1.3 to 2.9)	1.9 (1.3 to 2.8)	-2.6 (-10.24 to 5.15)	9.0 (6.0 to 12.9)	8.6 (5.7 to 12.4)	-4.7 (-12.36 to 3.33)
Other drug use disorders	55.2 (42.7 to 70.3)	78.4 (62.8 to 93.3)	**43.1 (6.75 to 62.82)**	115.3 (89.1 to 147.0)	154.7 (123.4 to 184.8)	**35.1 (1.38 to 53.16)**	36.6 (28.2 to 47.0)	57.0 (44.6 to 67.5)	**57.3 (0.72 to 84.84)**	152.7 (117.6 to 195.9)	223.7 (175.0 to 265.1)	**48.1 (-3.92 to 73.94)**	18.5 (14.0 to 23.6)	21.4 (16.7 to 26.4)	**15.8 (2.35 to 30.22)**	77.8 (58.4 to 99.4)	85.3 (66.3 to 106.0)	10.1 (-2.74 to 23.63)
Depressive disorders	367.8 (226.8 to 552.9)	402.0 (251.8 to 604.6)	**9.5 (5.11 to 14.71)**	718.5 (441.8 to 1,087.3)	687.7 (427.7 to 1,037.2)	**-4.2 (-7.37 to -0.33)**	151.2 (92.2 to 230.8)	167.5 (103.2 to 254.2)	**10.9 (4.72 to 18.34)**	610.6 (370.5 to 929.3)	583.6 (357.4 to 887.6)	**-4.3 (-9.11 to 0.55)**	216.6 (134.5 to 323.4)	234.6 (147.0 to 349.0)	**8.5 (3.79 to 14.71)**	821.9 (508.3 to 1,238.6)	790.0 (495.2 to 1,186.8)	-3.7 (-7.02 to 1.25)
Major depressive disorder	291.8 (170.4 to 465.6)	315.1 (185.9 to 499.4)	**8.2 (2.90 to 14.88)**	573.7 (335.1 to 923.9)	543.2 (317.0 to 864.2)	**-5.2 (-9.03 to -0.26)**	123.1 (71.8 to 198.1)	134.7 (78.7 to 218.2)	**9.6 (2.19 to 18.85)**	499.7 (289.1 to 805.9)	472.5 (273.4 to 760.4)	**-5.4 (-10.99 to 0.71)**	168.7 (99.2 to 268.1)	180.4 (107.5 to 282.9)	**7.2 (1.30 to 15.23)**	644.8 (376.9 to 1,029.5)	612.9 (359.8 to 969.6)	-4.7 (-8.69 to 1.65)
Dysthymia	76.0 (50.0 to 110.5)	86.9 (57.2 to 127.3)	**14.4 (12.43 to 16.31)**	144.8 (95.4 to 210.5)	144.5 (95.1 to 210.5)	-0.2 (-1.08 to 0.70)	28.0 (18.2 to 40.7)	32.7 (21.3 to 47.6)	**16.7 (14.31 to 19.32)**	110.9 (72.4 to 161.1)	111.1 (72.4 to 161.1)	0.2 (-1.16 to 1.46)	47.9 (31.6 to 70.9)	54.2 (35.8 to 79.9)	**13.0 (10.94 to 15.00)**	177.1 (115.7 to 260.7)	177.1 (115.9 to 259.5)	-0.0 (-1.06 to 1.13)
Bipolar disorder	72.3 (44.8 to 108.5)	80.3 (49.8 to 119.6)	**11.1 (7.34 to 14.64)**	142.7 (88.2 to 215.7)	142.0 (87.4 to 213.7)	-0.5 (-3.30 to 2.52)	29.6 (18.4 to 44.9)	33.1 (20.4 to 49.6)	**11.7 (7.40 to 16.22)**	119.6 (74.1 to 181.9)	118.7 (73.4 to 179.3)	-0.7 (-4.41 to 3.40)	42.6 (26.2 to 63.9)	47.2 (29.2 to 70.1)	**10.7 (6.33 to 15.05)**	165.2 (101.1 to 248.6)	165.1 (100.7 to 250.1)	-0.1 (-3.73 to 3.89)
Anxiety disorders	225.1 (111.5 to 355.0)	250.4 (128.0 to 395.6)	**11.4 (6.16 to 16.49)**	454.2 (209.7 to 734.3)	453.5 (208.8 to 731.7)	-0.2 (-0.96 to 0.64)	76.1 (38.7 to 123.4)	85.6 (44.6 to 138.7)	**12.8 (6.76 to 20.12)**	313.5 (151.3 to 514.8)	314.3 (150.8 to 517.1)	0.2 (-1.13 to 1.61)	149.0 (73.3 to 235.7)	164.8 (83.3 to 261.4)	**10.7 (5.44 to 15.33)**	591.7 (262.3 to 964.6)	592.0 (262.7 to 961.9)	0.0 (-0.91 to 0.97)
Eating disorders	35.4 (21.0 to 55.2)	37.4 (22.6 to 57.4)	**5.7 (1.77 to 10.54)**	77.1 (45.9 to 119.0)	81.0 (48.8 to 123.8)	**5.2 (1.59 to 9.10)**	6.5 (3.6 to 11.4)	6.7 (3.7 to 11.4)	3.3 (-1.94 to 9.67)	28.3 (15.7 to 49.2)	29.1 (16.4 to 49.2)	3.4 (-1.84 to 9.75)	28.9 (17.3 to 44.1)	30.7 (18.4 to 47.0)	**6.2 (2.04 to 11.08)**	126.6 (76.2 to 192.9)	134.0 (80.6 to 202.7)	**5.9 (2.02 to 10.05)**
Anorexia nervosa	8.6 (4.8 to 14.1)	10.5 (5.9 to 17.3)	**21.8 (12.91 to 31.40)**	19.0 (10.6 to 30.9)	22.7 (12.7 to 37.1)	**19.7 (11.65 to 28.74)**	1.3 (0.8 to 2.1)	1.4 (0.8 to 2.3)	**10.6 (-0.27 to 23.77)**	5.8 (3.4 to 9.3)	6.3 (3.8 to 10.3)	**10.1 (-0.66 to 22.66)**	7.3 (3.9 to 12.2)	9.1 (5.0 to 15.2)	**23.9 (13.71 to 36.80)**	32.4 (17.5 to 53.4)	39.3 (21.4 to 65.7)	**21.7 (12.41 to 33.09)**
Bulimia nervosa	26.8 (15.1 to 44.3)	26.9 (15.1 to 43.7)	0.4 (-2.62 to 4.46)	58.1 (33.2 to 95.3)	58.3 (33.2 to 94.4)	0.4 (-2.22 to 3.81)	5.2 (2.7 to 10.0)	5.3 (2.7 to 9.9)	1.4 (-3.88 to 8.51)	22.5 (11.7 to 42.9)	22.8 (12.0 to 42.4)	1.7 (-3.92 to 8.33)	21.6 (12.2 to 34.9)	21.6 (12.4 to 34.6)	0.2 (-2.88 to 4.14)	94.3 (54.4 to 151.4)	94.6 (54.7 to 150.8)	0.5 (-2.20 to 3.82)
Autistic spectrum disorders	52.5 (36.7 to 71.4)	58.5 (41.0 to 79.5)	**11.4 (10.10 to 12.83)**	113.4 (79.2 to 154.2)	114.0 (79.8 to 154.8)	0.5 (-0.68 to 1.76)	39.0 (27.1 to 52.8)	43.7 (30.5 to 59.2)	**12.0 (10.47 to 13.48)**	170.5 (118.7 to 231.5)	170.8 (119.5 to 232.5)	0.2 (-1.18 to 1.60)	13.5 (9.6 to 18.3)	14.9 (10.4 to 20.2)	**9.9 (7.38 to 12.45)**	56.9 (40.2 to 76.8)	56.9 (39.8 to 77.2)	-0.0 (-2.31 to 2.31)
Autism	33.2 (22.1 to 45.3)	37.0 (24.8 to 50.1)	**11.4 (9.41 to 13.32)**	71.8 (47.8 to 98.3)	72.2 (48.3 to 97.8)	0.5 (-1.17 to 2.23)	23.9 (15.9 to 32.4)	26.7 (17.9 to 36.4)	**11.9 (9.64 to 14.26)**	104.7 (69.5 to 142.0)	104.9 (70.2 to 142.6)	0.2 (-1.77 to 2.32)	9.3 (6.3 to 12.8)	10.3 (6.9 to 14.1)	**10.0 (6.53 to 13.38)**	39.2 (26.4 to 53.9)	39.2 (26.2 to 54.1)	0.0 (-3.07 to 3.17)
Asperger syndrome	19.3 (13.4 to 26.7)	21.5 (15.0 to 29.7)	**11.6 (10.01 to 13.19)**	41.6 (28.8 to 57.3)	41.8 (29.2 to 57.7)	0.5 (-0.85 to 1.96)	15.1 (10.5 to 20.9)	16.9 (11.8 to 23.4)	**12.0 (10.23 to 13.85)**	65.8 (45.6 to 91.1)	65.9 (46.0 to 91.1)	0.1 (-1.47 to 1.76)	4.2 (2.9 to 5.8)	4.6 (3.2 to 6.3)	**9.9 (6.50 to 13.06)**	17.6 (12.3 to 24.4)	17.6 (12.2 to 24.3)	-0.0 (-2.95 to 3.00)
Attention-deficit/hyperactivity disorder	2.9 (1.6 to 4.6)	2.9 (1.7 to 4.6)	1.8 (-0.54 to 3.94)	7.6 (4.3 to 12.0)	7.7 (4.4 to 12.1)	0.4 (-1.84 to 2.50)	2.2 (1.3 to 3.5)	2.3 (1.3 to 3.6)	1.9 (-0.65 to 4.53)	11.4 (6.5 to 18.1)	11.5 (6.5 to 18.1)	0.3 (-2.28 to 2.84)	0.7 (0.4 to 1.1)	0.7 (0.4 to 1.1)	1.4 (-3.36 to 6.45)	3.7 (2.1 to 5.7)	3.7 (2.1 to 5.7)	0.3 (-4.37 to 5.43)
Conduct disorder	41.1 (25.2 to 61.9)	41.9 (25.6 to 63.1)	**2.2 (0.63 to 3.74)**	110.7 (68.4 to 167.1)	111.0 (68.1 to 166.6)	0.2 (-1.19 to 1.71)	29.4 (18.4 to 44.6)	30.0 (18.5 to 45.6)	**2.1 (0.32 to 4.09)**	154.6 (96.1 to 234.1)	154.7 (95.9 to 233.1)	0.1 (-1.67 to 1.94)	11.7 (7.0 to 17.5)	11.9 (7.2 to 18.0)	2.2 (-0.49 to 4.98)	64.9 (39.4 to 97.4)	65.1 (39.2 to 98.0)	0.2 (-2.36 to 2.84)
Idiopathic intellectual disability	17.0 (10.7 to 24.5)	12.7 (7.4 to 19.3)	**-25.7 (-43.57 to -8.99)**	36.4 (22.8 to 52.4)	24.2 (14.3 to 36.9)	**-33.4 (-49.41 to -18.10)**	7.7 (4.7 to 11.3)	6.2 (3.6 to 9.5)	-19.2 (-41.13 to 8.01)	33.5 (20.5 to 49.3)	24.0 (14.0 to 36.7)	**-28.0 (-47.31 to -3.68)**	9.3 (5.9 to 13.5)	6.5 (3.6 to 9.9)	**-30.6 (-50.36 to -11.89)**	39.1 (25.0 to 56.6)	24.4 (13.5 to 37.3)	**-37.6 (-55.46 to -20.89)**
Other mental and substance use disorders	66.7 (45.3 to 89.6)	76.9 (52.3 to 103.1)	**15.4 (13.63 to 17.17)**	126.6 (86.0 to 170.4)	127.3 (86.5 to 170.3)	0.6 (-0.78 to 1.98)	42.0 (28.4 to 56.4)	48.7 (32.7 to 65.4)	**16.0 (13.71 to 18.61)**	164.5 (111.5 to 221.6)	164.9 (111.2 to 222.4)	0.2 (-1.33 to 1.89)	24.7 (17.0 to 33.5)	28.3 (19.4 to 38.4)	**14.4 (11.89 to 16.99)**	90.6 (61.9 to 122.9)	90.7 (61.9 to 123.3)	0.1 (-1.92 to 2.12)
**Diabetes, urogenital, blood, and endocrine diseases**	782.0 (639.1 to 941.2)	967.7 (752.7 to 1,205.1)	**23.5 (16.10 to 30.45)**	1,381.0 (1,114.0 to 1,685.7)	1,408.6 (1,081.3 to 1,769.9)	1.8 (-4.10 to 7.26)	343.8 (280.7 to 411.3)	445.5 (342.2 to 555.5)	**29.3 (19.51 to 38.91)**	1,314.0 (1,071.0 to 1,573.2)	1,315.8 (1,005.2 to 1,638.4)	-0.1 (-7.49 to 7.41)	438.1 (354.5 to 532.6)	522.2 (404.3 to 655.3)	**19.0 (10.85 to 26.83)**	1,483.9 (1,169.3 to 1,843.7)	1,514.6 (1,152.2 to 1,930.9)	1.9 (-3.82 to 7.50)
Diabetes mellitus	247.2 (201.5 to 303.1)	360.2 (262.9 to 468.9)	**45.1 (27.60 to 63.21)**	398.3 (321.0 to 493.4)	480.0 (349.3 to 629.6)	**20.1 (5.51 to 34.98)**	113.1 (92.2 to 137.5)	180.3 (130.9 to 240.6)	**58.8 (35.24 to 84.76)**	416.4 (337.6 to 508.8)	513.9 (368.7 to 690.0)	**23.0 (4.83 to 43.71)**	134.2 (107.8 to 166.6)	180.0 (131.9 to 236.4)	**33.7 (16.52 to 54.09)**	383.0 (304.2 to 478.7)	448.4 (326.4 to 591.2)	**16.7 (1.89 to 32.77)**
Acute glomerulonephritis	0.4 (0.4 to 0.5)	0.3 (0.2 to 0.3)	**-30.5 (-51.19 to -21.70)**	0.9 (0.7 to 1.1)	0.6 (0.4 to 0.6)	**-34.5 (-57.94 to -23.48)**	0.2 (0.2 to 0.3)	0.2 (0.1 to 0.2)	**-28.4 (-49.85 to -10.05)**	0.9 (0.7 to 1.3)	0.6 (0.4 to 0.7)	**-36.1 (-58.67 to -23.61)**	0.2 (0.2 to 0.3)	0.1 (0.1 to 0.2)	**-32.6 (-54.54 to -19.06)**	0.8 (0.6 to 1.0)	0.5 (0.3 to 0.6)	**-33.6 (-59.75 to -19.86)**
Chronic kidney disease	258.3 (191.3 to 326.9)	284.5 (204.2 to 369.2)	**9.9 (5.11 to 14.46)**	449.6 (328.8 to 577.4)	411.4 (291.4 to 537.6)	**-8.7 (-12.14 to -5.28)**	117.3 (87.4 to 149.9)	129.4 (91.1 to 169.4)	**10.0 (3.35 to 16.94)**	445.3 (332.4 to 571.4)	386.7 (272.3 to 511.4)	**-13.4 (-18.74 to -8.39)**	141.0 (102.9 to 179.7)	155.1 (111.3 to 199.9)	**9.9 (4.95 to 15.08)**	461.6 (332.3 to 600.1)	437.2 (308.7 to 571.6)	**-5.4 (-9.09 to -1.51)**

Chronic kidney disease due to diabetes mellitus	45.3 (32.9 to 58.7)	58.5 (39.5 to 77.6)	**29.1 (11.78 to 53.45)**	77.3 (55.1 to 101.7)	81.0 (54.7 to 108.8)	4.8 (-8.63 to 23.28)	19.8 (13.8 to 26.1)	25.8 (17.2 to 35.5)	**31.0 (7.65 to 85.05)**	73.5 (51.1 to 97.0)	74.1 (49.6 to 102.2)	1.2 (-16.51 to 41.65)	25.5 (17.9 to 33.6)	32.7 (22.1 to 44.1)	**28.2 (11.67 to 45.75)**	82.4 (56.3 to 109.9)	88.0 (58.8 to 119.2)	6.8 (-7.27 to 22.42)
Chronic kidney disease due to hypertension	71.0 (50.3 to 91.9)	36.4 (27.3 to 46.4)	**-48.5 (-54.21 to -41.56)**	120.7 (84.9 to 159.9)	49.6 (36.6 to 63.9)	**-58.7 (-63.36 to -53.50)**	34.9 (24.3 to 45.8)	17.9 (13.1 to 23.0)	**-48.2 (-56.28 to -38.89)**	130.5 (91.5 to 171.6)	52.2 (37.7 to 67.8)	**-59.8 (-65.87 to -52.98)**	36.1 (25.7 to 47.4)	18.4 (13.4 to 24.2)	**-48.7 (-56.23 to -39.13)**	113.7 (79.9 to 153.3)	47.5 (34.0 to 63.1)	**-58.0 (-63.94 to -50.47)**
Chronic kidney disease due to glomerulonephritis	25.6 (19.0 to 32.6)	44.5 (30.2 to 60.1)	**73.5 (49.80 to 96.15)**	48.7 (35.9 to 62.8)	70.8 (48.0 to 96.4)	**44.7 (25.71 to 63.93)**	12.9 (9.3 to 17.0)	22.1 (14.6 to 30.8)	**70.1 (43.55 to 100.24)**	51.2 (36.9 to 66.8)	70.6 (47.2 to 98.6)	**37.7 (16.08 to 62.52)**	12.6 (9.3 to 16.3)	22.4 (15.1 to 30.6)	**77.3 (52.36 to 108.67)**	47.3 (34.0 to 62.9)	71.3 (47.0 to 99.9)	**50.5 (28.37 to 78.83)**
Chronic kidney disease due to other causes	116.5 (87.6 to 147.7)	145.1 (104.2 to 190.3)	**24.3 (14.42 to 33.77)**	202.9 (151.1 to 259.6)	209.9 (149.9 to 276.1)	3.2 (-4.18 to 10.91)	49.8 (37.1 to 63.8)	63.6 (44.4 to 85.1)	**27.5 (13.31 to 39.51)**	190.2 (142.2 to 243.6)	189.8 (131.8 to 254.2)	-0.5 (-11.01 to 9.18)	66.8 (49.3 to 85.4)	81.5 (58.1 to 107.0)	**22.0 (12.08 to 32.98)**	218.2 (158.0 to 281.3)	230.4 (161.6 to 303.0)	5.5 (-2.43 to 14.95)
Urinary diseases and male infertility	81.6 (62.8 to 103.8)	124.5 (85.4 to 157.6)	**53.9 (6.07 to 77.36)**	122.2 (94.5 to 155.6)	145.9 (100.3 to 186.7)	20.3 (-15.78 to 37.22)	59.6 (43.1 to 79.6)	85.9 (59.7 to 112.8)	**44.6 (13.70 to 58.85)**	211.9 (153.9 to 281.6)	223.1 (154.4 to 295.6)	5.6 (-17.62 to 15.60)	21.9 (18.9 to 31.3)	38.6 (21.5 to 47.2)	83.3 (-12.19 to 140.30)	56.3 (48.7 to 78.1)	78.6 (45.0 to 96.4)	44.6 (-27.43 to 88.76)
Interstitial nephritis and urinary tract infections	27.4 (23.8 to 40.1)	53.1 (30.4 to 62.8)	103.7 (-11.76 to 157.65)	40.0 (35.0 to 58.4)	58.4 (34.0 to 68.8)	52.9 (-32.47 to 92.73)	10.1 (8.7 to 16.1)	20.6 (10.4 to 25.2)	114.1 (-17.63 to 171.67)	37.8 (32.7 to 60.4)	52.3 (26.6 to 63.7)	45.2 (-43.83 to 84.00)	17.3 (14.8 to 26.3)	32.5 (17.4 to 40.1)	99.2 (-12.98 to 161.49)	42.6 (36.8 to 63.1)	63.8 (35.2 to 78.6)	58.0 (-27.60 to 106.61)
Urolithiasis	6.5 (4.4 to 10.1)	10.8 (5.9 to 18.5)	**65.0 (17.74 to 112.71)**	10.6 (7.1 to 16.6)	14.2 (7.6 to 24.6)	32.8 (-6.37 to 69.67)	3.4 (2.1 to 5.6)	6.8 (3.6 to 12.1)	**102.5 (47.53 to 156.82)**	12.4 (7.8 to 20.8)	19.1 (10.0 to 33.7)	**52.6 (9.32 to 90.15)**	3.2 (2.1 to 4.5)	4.0 (2.3 to 6.4)	26.3 (-12.94 to 75.50)	9.3 (6.1 to 13.7)	10.0 (5.4 to 16.4)	6.4 (-28.30 to 45.42)
Benign prostatic hyperplasia	41.0 (26.9 to 57.7)	53.6 (35.0 to 74.9)	**30.5 (26.60 to 34.74)**	60.2 (39.5 to 84.6)	63.4 (41.4 to 88.8)	**5.3 (2.08 to 8.62)**	41.0 (26.9 to 57.7)	53.6 (35.0 to 74.9)	**30.5 (26.60 to 34.74)**	142.0 (93.2 to 199.4)	136.4 (89.1 to 191.0)	**-3.9 (-6.75 to -0.87)**	--	--	--	--	--	--
Male infertility due to other causes	1.4 (0.6 to 2.9)	1.5 (0.6 to 2.9)	3.6 (-11.53 to 20.25)	2.9 (1.2 to 6.0)	2.9 (1.3 to 5.8)	0.4 (-14.38 to 16.36)	1.4 (0.6 to 2.9)	1.5 (0.6 to 2.9)	3.6 (-11.53 to 20.25)	5.8 (2.4 to 11.9)	5.8 (2.5 to 11.6)	0.4 (-14.39 to 16.38)	--	--	--	--	--	--
Other urinary diseases	5.2 (3.2 to 6.9)	5.6 (2.8 to 8.9)	11.3 (-46.54 to 104.51)	8.4 (5.2 to 11.2)	7.0 (3.6 to 11.1)	-13.7 (-56.98 to 55.27)	3.7 (1.9 to 5.1)	3.5 (1.5 to 6.5)	0.5 (-58.19 to 107.50)	13.9 (7.2 to 19.0)	9.5 (4.1 to 17.6)	-26.6 (-68.35 to 48.44)	1.5 (0.9 to 2.4)	2.1 (0.9 to 3.2)	43.8 (-31.54 to 128.53)	4.4 (2.9 to 6.7)	4.9 (2.4 to 7.1)	12.3 (-43.43 to 72.76)
Gynecological diseases	60.5 (39.3 to 92.9)	68.6 (43.9 to 106.3)	**13.3 (7.48 to 19.50)**	121.7 (79.1 to 186.7)	122.1 (78.4 to 186.8)	0.3 (-4.78 to 6.12)	--	--	--	--	--	--	60.5 (39.3 to 92.9)	68.6 (43.9 to 106.3)	**13.3 (7.48 to 19.50)**	243.2 (157.9 to 372.8)	243.4 (156.3 to 372.1)	0.1 (-4.96 to 5.93)
Uterine fibroids	15.9 (7.9 to 29.0)	19.2 (9.3 to 35.7)	**21.0 (11.78 to 32.21)**	31.9 (15.9 to 58.3)	32.4 (15.7 to 60.2)	1.5 (-6.39 to 10.38)	--	--	--	--	--	--	15.9 (7.9 to 29.0)	19.2 (9.3 to 35.7)	**21.0 (11.78 to 32.21)**	63.9 (31.8 to 116.6)	64.3 (31.2 to 119.6)	0.8 (-6.98 to 9.69)
Polycystic ovarian syndrome	7.7 (3.6 to 14.7)	8.6 (4.0 to 16.2)	**12.2 (4.14 to 20.43)**	15.7 (7.4 to 30.2)	16.1 (7.5 to 30.3)	2.4 (-5.21 to 10.12)	--	--	--	--	--	--	7.7 (3.6 to 14.7)	8.6 (4.0 to 16.2)	**12.2 (4.14 to 20.43)**	31.5 (14.9 to 60.5)	32.2 (15.1 to 60.5)	2.1 (-5.46 to 9.81)
Female infertility due to other causes	0.3 (0.1 to 0.7)	0.4 (0.1 to 0.9)	25.6 (-52.68 to 172.21)	0.7 (0.2 to 1.5)	0.8 (0.2 to 1.7)	20.1 (-54.19 to 161.82)	--	--	--	--	--	--	0.3 (0.1 to 0.7)	0.4 (0.1 to 0.9)	25.6 (-52.68 to 172.21)	1.4 (0.5 to 3.0)	1.5 (0.5 to 3.4)	20.1 (-54.27 to 161.77)
Endometriosis	12.5 (8.4 to 17.5)	13.6 (9.0 to 19.1)	9.3 (-5.20 to 25.30)	25.4 (17.0 to 35.5)	25.2 (16.6 to 35.1)	-0.6 (-13.93 to 14.05)	--	--	--	--	--	--	12.5 (8.4 to 17.5)	13.6 (9.0 to 19.1)	9.3 (-5.20 to 25.30)	50.9 (34.0 to 71.0)	50.3 (33.2 to 70.0)	-0.9 (-14.16 to 13.70)
Genital prolapse	9.7 (4.9 to 18.1)	11.1 (5.5 to 20.6)	**14.4 (9.33 to 19.91)**	18.4 (9.2 to 34.6)	17.9 (8.8 to 33.2)	-2.9 (-7.51 to 1.92)	--	--	--	--	--	--	9.7 (4.9 to 18.1)	11.1 (5.5 to 20.6)	**14.4 (9.33 to 19.91)**	36.3 (18.2 to 68.2)	35.5 (17.4 to 65.8)	-2.4 (-7.14 to 2.44)
Premenstrual syndrome	10.2 (6.3 to 15.6)	10.9 (6.8 to 16.7)	7.0 (-10.28 to 27.52)	21.1 (12.9 to 32.0)	21.1 (13.0 to 32.4)	0.6 (-15.67 to 19.45)	--	--	--	--	--	--	10.2 (6.3 to 15.6)	10.9 (6.8 to 16.7)	7.0 (-10.28 to 27.52)	42.3 (25.8 to 64.3)	42.4 (26.2 to 65.0)	0.5 (-15.78 to 19.40)
Other gynecological diseases	4.2 (3.0 to 5.5)	4.7 (3.2 to 6.8)	**13.7 (0.53 to 50.84)**	8.4 (6.0 to 11.3)	8.6 (5.7 to 12.5)	2.2 (-9.89 to 37.58)	--	--	--	--	--	--	4.2 (3.0 to 5.5)	4.7 (3.2 to 6.8)	**13.7 (0.53 to 50.84)**	16.9 (12.0 to 22.7)	17.2 (11.5 to 24.9)	2.3 (-9.87 to 37.72)
Hemoglobinopathies and hemolytic anemias	59.0 (41.5 to 81.7)	57.7 (39.8 to 80.4)	-2.2 (-6.86 to 2.43)	136.6 (95.8 to 190.0)	121.8 (83.6 to 169.8)	**-11.0 (-14.88 to -6.98)**	15.1 (10.9 to 20.6)	12.6 (8.9 to 17.3)	**-16.9 (-22.83 to -9.45)**	73.7 (52.8 to 101.1)	55.1 (39.0 to 76.6)	**-25.4 (-30.16 to -19.62)**	43.9 (30.3 to 61.5)	45.2 (30.6 to 63.5)	2.9 (-1.87 to 8.36)	200.5 (138.2 to 282.9)	189.5 (128.6 to 265.1)	**-5.6 (-9.65 to -0.83)**
Thalassemias	1.3 (1.0 to 1.7)	0.8 (0.6 to 1.2)	**-39.5 (-51.03 to -21.97)**	3.3 (2.4 to 4.1)	1.9 (1.5 to 2.8)	**-40.6 (-52.22 to -22.55)**	0.7 (0.4 to 1.0)	0.4 (0.3 to 0.6)	**-39.4 (-49.78 to -19.82)**	3.3 (2.1 to 4.8)	2.0 (1.3 to 3.0)	**-40.4 (-50.71 to -20.35)**	0.6 (0.4 to 0.9)	0.4 (0.3 to 0.6)	**-39.4 (-53.39 to -13.38)**	3.2 (2.1 to 4.4)	1.9 (1.3 to 3.0)	**-40.7 (-54.69 to -13.75)**
Thalassemia trait	28.8 (19.5 to 41.3)	30.7 (20.6 to 43.5)	**6.5 (2.87 to 10.19)**	67.0 (45.4 to 95.8)	64.3 (43.6 to 91.3)	**-4.0 (-6.76 to -0.94)**	4.9 (3.2 to 7.2)	4.0 (2.6 to 5.8)	**-17.8 (-26.17 to -9.46)**	25.6 (16.9 to 37.3)	19.1 (12.6 to 27.6)	**-25.2 (-31.25 to -19.11)**	23.9 (16.3 to 34.2)	26.7 (18.0 to 37.9)	**11.5 (7.85 to 15.45)**	108.7 (73.8 to 154.3)	110.0 (74.5 to 155.1)	1.2 (-1.78 to 4.63)
Sickle cell disorders	2.6 (1.9 to 3.2)	2.1 (1.7 to 2.8)	-17.0 (-28.58 to 3.62)	5.8 (4.3 to 7.0)	4.2 (3.2 to 5.5)	**-27.0 (-37.14 to -8.78)**	1.4 (0.9 to 1.9)	1.1 (0.8 to 1.6)	**-19.4 (-30.24 to 6.63)**	6.2 (4.0 to 8.2)	4.4 (3.1 to 6.2)	**-28.8 (-38.53 to -6.42)**	1.2 (0.9 to 1.6)	1.0 (0.8 to 1.5)	-14.1 (-28.35 to 11.68)	5.4 (3.7 to 6.8)	4.0 (3.0 to 5.6)	**-24.4 (-36.97 to -2.19)**
Sickle cell trait	15.9 (10.4 to 23.2)	14.7 (9.6 to 21.2)	**-7.6 (-14.99 to -0.22)**	38.8 (25.5 to 56.1)	33.5 (22.1 to 48.0)	**-13.6 (-20.15 to -7.13)**	2.2 (1.5 to 3.3)	1.5 (1.0 to 2.3)	**-31.2 (-38.02 to -25.07)**	11.9 (7.8 to 17.5)	7.5 (4.9 to 11.2)	**-36.9 (-44.43 to -31.08)**	13.7 (9.0 to 19.7)	13.1 (8.6 to 19.0)	-3.7 (-11.82 to 4.96)	66.2 (43.8 to 95.8)	60.1 (39.5 to 86.2)	**-9.2 (-16.55 to -1.62)**
G6PD deficiency	0.2 (0.2 to 0.3)	0.1 (0.1 to 0.2)	**-37.3 (-49.83 to -23.39)**	0.5 (0.4 to 0.6)	0.3 (0.2 to 0.4)	**-46.6 (-57.98 to -33.38)**	0.1 (0.0 to 0.1)	<0.05 (0.0 to 0.1)	**-36.6 (-45.35 to -23.42)**	0.3 (0.2 to 0.4)	0.2 (0.1 to 0.2)	**-43.4 (-51.22 to -31.91)**	0.1 (0.1 to 0.2)	0.1 (0.1 to 0.1)	**-37.4 (-54.70 to -18.10)**	0.6 (0.5 to 0.9)	0.3 (0.2 to 0.5)	**-48.2 (-64.15 to -29.96)**
G6PD trait	0.5 (0.3 to 0.8)	0.5 (0.3 to 0.8)	0.3 (-20.32 to 22.83)	1.3 (0.8 to 1.9)	1.2 (0.7 to 1.9)	-6.2 (-27.10 to 15.74)	--	--	--	--	--	--	0.5 (0.3 to 0.8)	0.5 (0.3 to 0.8)	0.3 (-20.32 to 22.83)	2.6 (1.5 to 3.9)	2.4 (1.4 to 3.8)	-6.2 (-27.16 to 15.82)
Other hemoglobinopathies and hemolytic anemias	9.6 (7.2 to 12.4)	8.8 (6.3 to 12.3)	-9.0 (-21.82 to 7.42)	20.1 (15.0 to 26.6)	16.4 (11.6 to 23.2)	**-18.7 (-29.74 to -4.11)**	5.9 (4.3 to 7.8)	5.5 (3.8 to 7.5)	-7.0 (-17.32 to 4.51)	26.5 (19.2 to 35.7)	22.0 (15.3 to 30.5)	**-17.2 (-26.02 to -7.74)**	3.7 (2.7 to 4.9)	3.3 (2.3 to 4.9)	-11.7 (-33.03 to 23.21)	13.9 (9.9 to 18.5)	10.8 (7.2 to 17.0)	-22.1 (-41.48 to 17.83)
Endocrine, metabolic, blood, and immune disorders	74.9 (59.6 to 85.5)	71.8 (58.9 to 88.0)	-4.0 (-14.42 to 18.73)	151.6 (121.4 to 173.8)	126.8 (104.0 to 154.4)	**-16.3 (-25.11 to 1.26)**	38.4 (28.6 to 44.5)	37.2 (30.2 to 48.3)	-2.8 (-16.26 to 30.43)	165.8 (124.4 to 192.6)	136.5 (110.3 to 175.9)	-17.4 (-28.32 to 9.66)	36.5 (28.3 to 42.8)	34.6 (28.2 to 43.5)	-5.1 (-14.08 to 9.55)	138.5 (109.2 to 163.2)	117.0 (95.0 to 145.3)	**-15.6 (-23.93 to -4.45)**
**Musculoskeletal disorders**	1,476.0 (1,073.4 to 1,920.6)	1,749.6 (1,265.0 to 2,301.8)	**18.5 (14.67 to 22.29)**	2,671.3 (1,937.6 to 3,487.7)	2,676.0 (1,914.7 to 3,547.8)	0.2 (-3.47 to 3.57)	580.4 (418.5 to 769.5)	707.7 (506.8 to 938.7)	**22.0 (16.20 to 28.26)**	2,251.1 (1,613.5 to 2,990.1)	2,258.7 (1,603.9 to 3,004.8)	0.4 (-4.75 to 5.88)	895.5 (657.2 to 1,164.7)	1,041.9 (750.8 to 1,355.6)	**16.3 (10.42 to 21.54)**	3,052.5 (2,215.8 to 4,004.5)	3,073.7 (2,208.0 to 4,041.1)	0.7 (-4.49 to 5.40)
Rheumatoid arthritis	88.1 (68.6 to 109.5)	80.8 (60.8 to 102.9)	**-8.5 (-14.06 to -3.19)**	136.7 (106.1 to 170.5)	103.9 (77.5 to 133.3)	**-24.1 (-28.40 to -19.76)**	20.0 (15.4 to 25.1)	22.6 (17.1 to 28.8)	**12.9 (0.40 to 23.09)**	72.3 (55.4 to 90.7)	63.1 (47.6 to 80.6)	**-12.8 (-22.10 to -5.12)**	68.1 (53.3 to 84.9)	58.2 (43.4 to 74.6)	**-14.7 (-20.47 to -8.74)**	188.9 (146.7 to 236.5)	140.8 (104.1 to 181.2)	**-25.5 (-30.28 to -20.66)**
Osteoarthritis	109.8 (72.4 to 158.1)	136.1 (90.2 to 194.5)	**24.0 (17.10 to 30.01)**	169.0 (110.9 to 243.4)	165.9 (109.7 to 237.1)	-1.8 (-7.59 to 3.46)	36.7 (24.3 to 53.3)	48.9 (32.5 to 70.3)	**33.4 (23.60 to 43.92)**	132.9 (88.0 to 192.8)	130.0 (86.3 to 186.8)	-2.1 (-9.45 to 5.71)	73.1 (48.3 to 105.5)	87.3 (57.8 to 125.8)	**19.4 (11.53 to 27.55)**	197.3 (129.8 to 285.2)	197.6 (130.7 to 284.2)	0.3 (-6.93 to 7.55)
Low back and neck pain	1,077.7 (758.3 to 1,447.0)	1,278.4 (892.6 to 1,738.5)	**18.7 (13.38 to 23.72)**	2,008.3 (1,409.5 to 2,701.9)	2,028.6 (1,404.0 to 2,765.6)	1.0 (-3.89 to 5.51)	479.1 (333.9 to 645.8)	569.6 (394.4 to 766.8)	**19.0 (12.34 to 26.29)**	1,879.0 (1,306.0 to 2,529.5)	1,865.7 (1,287.6 to 2,516.5)	-0.7 (-6.42 to 5.60)	598.6 (421.4 to 799.6)	708.8 (494.6 to 959.0)	**18.4 (10.72 to 25.76)**	2,128.4 (1,502.7 to 2,857.2)	2,185.2 (1,517.9 to 2,956.0)	2.7 (-4.24 to 9.45)
Low back pain	700.6 (484.1 to 969.8)	845.2 (573.6 to 1,172.3)	**20.7 (14.08 to 27.96)**	1,299.0 (895.2 to 1,805.2)	1,334.9 (902.5 to 1,855.2)	2.8 (-3.14 to 9.50)	324.3 (223.1 to 455.0)	387.8 (262.7 to 537.2)	**19.7 (9.93 to 30.96)**	1,273.5 (873.2 to 1,787.9)	1,267.7 (857.4 to 1,751.8)	-0.4 (-8.73 to 10.05)	376.2 (257.6 to 520.3)	457.4 (309.7 to 632.7)	**21.6 (12.27 to 31.07)**	1,320.6 (905.9 to 1,833.9)	1,399.0 (939.3 to 1,947.4)	6.0 (-3.00 to 15.06)
Neck pain	377.1 (264.1 to 518.2)	433.2 (305.6 to 599.0)	**15.0 (3.45 to 21.90)**	709.3 (497.3 to 977.3)	693.7 (486.9 to 959.7)	-2.1 (-11.95 to 3.86)	154.8 (107.6 to 215.6)	181.8 (126.5 to 250.9)	**17.6 (9.51 to 26.07)**	605.5 (420.5 to 842.9)	598.0 (415.0 to 826.2)	-1.1 (-7.82 to 5.80)	222.3 (155.5 to 304.6)	251.4 (174.5 to 347.8)	**13.2 (-2.11 to 24.37)**	807.8 (563.5 to 1,110.4)	786.2 (543.9 to 1,091.0)	-2.5 (-16.24 to 7.16)
Gout	3.3 (2.3 to 4.5)	4.2 (2.9 to 5.7)	**26.8 (17.64 to 36.48)**	5.5 (3.8 to 7.4)	5.5 (3.8 to 7.5)	1.1 (-6.28 to 8.88)	2.3 (1.6 to 3.1)	3.0 (2.1 to 4.0)	**31.5 (20.31 to 43.87)**	8.4 (5.8 to 11.3)	8.3 (5.7 to 11.3)	-0.2 (-8.92 to 8.98)	1.1 (0.7 to 1.4)	1.2 (0.9 to 1.7)	**16.7 (5.13 to 30.42)**	3.0 (2.1 to 4.1)	3.0 (2.1 to 4.0)	-0.9 (-10.84 to 10.38)
Other musculoskeletal disorders	197.0 (134.3 to 273.5)	250.1 (171.3 to 352.3)	**26.9 (21.38 to 31.58)**	351.8 (239.8 to 490.0)	372.0 (252.6 to 524.3)	**5.7 (1.96 to 9.18)**	42.3 (27.4 to 63.7)	63.7 (41.1 to 95.8)	**50.5 (36.12 to 63.72)**	158.6 (101.9 to 239.1)	191.6 (122.0 to 288.3)	**20.9 (9.72 to 32.69)**	154.6 (106.2 to 213.1)	186.4 (128.0 to 258.4)	**20.5 (15.25 to 25.24)**	534.9 (367.5 to 742.2)	547.0 (373.4 to 758.5)	2.2 (-1.94 to 5.39)
**Other non-communicable diseases**	1,220.8 (895.7 to 1,662.4)	1,245.2 (884.5 to 1,718.6)	1.8 (-2.67 to 6.02)	2,434.3 (1,837.1 to 3,252.7)	2,045.0 (1,485.7 to 2,787.8)	**-16.1 (-21.14 to -11.26)**	611.3 (452.5 to 830.3)	631.0 (453.3 to 872.0)	3.1 (-2.17 to 8.48)	2,624.4 (1,990.6 to 3,509.6)	2,161.1 (1,574.3 to 2,964.9)	**-17.8 (-22.79 to -12.37)**	609.5 (444.9 to 829.8)	614.2 (433.9 to 845.7)	0.6 (-3.48 to 4.14)	2,260.9 (1,701.4 to 3,019.5)	1,938.0 (1,414.8 to 2,645.4)	**-14.4 (-19.67 to -9.37)**
Congenital anomalies	237.7 (213.8 to 263.4)	196.6 (164.2 to 232.5)	**-17.3 (-26.43 to -7.50)**	593.8 (540.7 to 655.1)	429.7 (360.9 to 509.7)	**-27.7 (-36.47 to -17.50)**	126.2 (111.8 to 141.5)	103.6 (85.1 to 124.3)	**-18.0 (-27.53 to -7.52)**	628.7 (560.2 to 698.6)	453.0 (373.2 to 541.9)	**-28.0 (-36.94 to -17.28)**	111.4 (98.9 to 125.9)	93.0 (77.8 to 110.1)	**-16.6 (-25.62 to -6.56)**	558.2 (496.2 to 628.6)	405.8 (342.5 to 485.9)	**-27.3 (-36.58 to -16.78)**
Neural tube defects	15.2 (13.3 to 18.3)	10.1 (8.1 to 12.1)	**-33.5 (-47.28 to -21.84)**	39.9 (35.1 to 48.4)	23.8 (19.1 to 28.7)	**-40.0 (-53.06 to -28.76)**	6.4 (5.4 to 7.8)	4.5 (3.5 to 5.6)	**-28.6 (-45.82 to -14.92)**	32.6 (27.8 to 40.1)	21.0 (16.3 to 26.0)	**-35.2 (-51.35 to -21.07)**	8.9 (7.5 to 10.7)	5.6 (4.3 to 6.9)	**-36.8 (-49.93 to -23.42)**	47.5 (40.4 to 58.0)	26.8 (20.7 to 33.2)	**-43.4 (-56.00 to -30.32)**
Congenital heart anomalies	94.0 (83.2 to 107.0)	55.2 (43.4 to 68.7)	**-41.4 (-49.85 to -32.42)**	243.1 (218.2 to 274.8)	129.0 (102.9 to 158.1)	**-47.0 (-54.65 to -38.45)**	51.3 (45.1 to 58.6)	29.3 (23.0 to 36.3)	**-43.0 (-52.19 to -33.26)**	261.7 (231.2 to 297.3)	135.7 (107.4 to 166.7)	**-48.2 (-56.57 to -38.80)**	42.8 (37.0 to 49.7)	25.9 (20.1 to 32.8)	**-39.5 (-49.15 to -29.31)**	223.9 (195.9 to 256.6)	122.0 (95.6 to 152.3)	**-45.5 (-54.49 to -35.62)**
Orofacial clefts	0.9 (0.6 to 1.2)	0.7 (0.5 to 1.0)	**-17.3 (-39.79 to -0.77)**	2.0 (1.4 to 2.8)	1.4 (1.0 to 2.0)	**-29.1 (-50.69 to -12.49)**	0.5 (0.3 to 0.8)	0.4 (0.3 to 0.5)	-19.3 (-47.69 to 5.22)	2.3 (1.5 to 3.8)	1.5 (1.0 to 2.2)	**-30.7 (-57.76 to -7.66)**	0.4 (0.3 to 0.5)	0.3 (0.2 to 0.5)	-13.5 (-35.19 to 5.76)	1.7 (1.2 to 2.5)	1.3 (0.8 to 1.8)	**-25.4 (-47.62 to -7.87)**
Down syndrome	14.9 (12.2 to 18.0)	19.9 (16.1 to 23.8)	**34.1 (8.25 to 54.32)**	30.3 (24.8 to 36.8)	34.3 (27.4 to 41.2)	13.5 (-10.76 to 31.25)	7.3 (5.8 to 9.1)	9.8 (7.5 to 12.2)	**35.1 (-2.02 to 61.70)**	30.5 (24.4 to 38.4)	34.3 (25.7 to 42.8)	13.2 (-20.58 to 35.90)	7.6 (6.2 to 9.4)	10.1 (7.8 to 12.4)	**33.6 (2.75 to 54.57)**	30.1 (24.5 to 38.1)	34.2 (26.1 to 42.0)	14.2 (-15.83 to 32.77)
Turner syndrome	<0.05 (0.0 to 0.1)	<0.05 (0.0 to 0.1)	**14.7 (-0.97 to 34.99)**	0.1 (0.0 to 0.1)	0.1 (0.0 to 0.1)	3.9 (-9.99 to 22.54)	--	--	--	--	--	--	<0.05 (0.0 to 0.1)	<0.05 (0.0 to 0.1)	**14.7 (-0.97 to 34.99)**	0.2 (0.1 to 0.3)	0.2 (0.1 to 0.3)	4.0 (-9.96 to 22.58)
Klinefelter syndrome	<0.05 (0.0 to <0.05)	<0.05 (0.0 to <0.05)	**31.0 (7.55 to 57.04)**	<0.05 (0.0 to <0.05)	<0.05 (0.0 to 0.1)	**19.6 (-1.77 to 42.95)**	<0.05 (0.0 to <0.05)	<0.05 (0.0 to <0.05)	**31.0 (7.55 to 57.04)**	<0.05 (0.0 to 0.1)	0.1 (0.0 to 0.1)	**19.3 (-2.06 to 42.47)**	--	--	--	--	--	--
Chromosomal unbalanced rearrangements	26.7 (21.7 to 32.4)	33.0 (26.2 to 40.6)	**23.7 (9.83 to 40.12)**	57.9 (47.5 to 69.7)	62.0 (49.2 to 75.8)	7.1 (-6.59 to 23.53)	12.0 (9.5 to 14.8)	15.5 (12.0 to 19.4)	**29.3 (8.26 to 52.38)**	52.1 (41.8 to 64.7)	57.4 (45.1 to 72.3)	10.5 (-9.53 to 31.61)	14.7 (11.7 to 18.1)	17.6 (13.8 to 21.7)	**19.5 (2.86 to 37.92)**	63.9 (50.5 to 79.2)	66.7 (52.3 to 82.3)	4.6 (-12.58 to 22.17)
Other congenital anomalies	85.9 (74.7 to 98.4)	77.6 (61.9 to 96.6)	-9.7 (-23.64 to 5.45)	220.5 (194.8 to 249.5)	179.2 (145.0 to 222.3)	**-18.8 (-31.82 to -3.96)**	48.8 (41.8 to 56.7)	44.2 (35.0 to 55.5)	-9.5 (-23.53 to 7.21)	249.4 (216.6 to 285.9)	203.0 (161.3 to 253.5)	**-18.6 (-31.95 to -2.52)**	37.0 (31.6 to 43.4)	33.4 (26.2 to 42.2)	-9.7 (-25.11 to 7.21)	190.9 (164.6 to 221.3)	154.7 (121.7 to 194.3)	**-18.9 (-33.96 to -2.36)**
Skin and subcutaneous diseases	364.8 (239.7 to 556.1)	393.0 (264.6 to 586.8)	**8.0 (2.73 to 12.18)**	766.7 (500.0 to 1,174.9)	738.0 (487.0 to 1,102.6)	**-3.6 (-7.61 to 0.04)**	185.5 (119.2 to 284.9)	197.4 (131.5 to 297.0)	**6.8 (-0.75 to 13.01)**	798.7 (510.4 to 1,229.9)	751.5 (491.6 to 1,140.4)	**-5.7 (-11.90 to -0.57)**	179.3 (121.0 to 264.1)	195.6 (133.3 to 285.6)	**9.2 (3.57 to 13.89)**	734.5 (487.3 to 1,107.8)	724.3 (483.8 to 1,080.0)	-1.3 (-5.80 to 2.98)
Dermatitis	104.1 (66.0 to 152.1)	113.9 (72.0 to 166.7)	**9.5 (7.64 to 11.13)**	226.1 (142.3 to 328.7)	224.7 (141.8 to 326.1)	-0.6 (-1.49 to 0.31)	48.3 (30.4 to 70.5)	53.2 (33.6 to 77.8)	**10.1 (7.99 to 12.09)**	212.2 (133.6 to 310.0)	211.2 (133.8 to 307.4)	-0.4 (-1.68 to 0.87)	55.8 (35.3 to 81.3)	60.8 (38.4 to 88.4)	**8.9 (6.88 to 10.66)**	239.7 (150.5 to 350.2)	238.3 (149.7 to 347.1)	-0.6 (-1.77 to 0.65)
Psoriasis	56.8 (38.8 to 79.5)	65.5 (44.6 to 91.5)	**15.3 (12.76 to 17.64)**	110.4 (75.2 to 153.5)	110.5 (75.5 to 153.2)	0.1 (-1.37 to 1.66)	28.7 (19.3 to 40.5)	33.6 (22.7 to 47.4)	**17.2 (14.26 to 20.35)**	115.0 (77.9 to 161.5)	115.3 (78.4 to 161.8)	0.3 (-1.67 to 2.20)	28.1 (19.0 to 39.5)	31.8 (21.6 to 45.0)	**13.2 (10.12 to 16.42)**	105.9 (71.8 to 148.2)	105.9 (72.4 to 148.1)	-0.0 (-2.33 to 2.21)
Cellulitis	5.3 (4.2 to 7.2)	9.5 (6.5 to 11.8)	**84.4 (-3.21 to 140.63)**	8.8 (7.0 to 11.8)	11.8 (8.3 to 14.6)	36.5 (-22.41 to 73.61)	2.4 (1.9 to 3.2)	4.0 (2.7 to 5.2)	**71.8 (-8.51 to 126.70)**	9.3 (7.2 to 12.5)	11.5 (7.9 to 14.9)	26.0 (-29.73 to 62.38)	2.9 (2.3 to 4.0)	5.4 (3.3 to 6.9)	**95.7 (-0.89 to 169.15)**	8.2 (6.4 to 11.4)	11.8 (7.5 to 14.7)	47.2 (-16.93 to 99.25)
Pyoderma	1.3 (1.1 to 1.8)	6.7 (4.8 to 8.7)	**409.1 (187.69 to 614.78)**	2.4 (1.9 to 3.4)	8.1 (5.9 to 10.5)	**244.6 (101.22 to 379.61)**	0.7 (0.5 to 0.9)	2.6 (1.9 to 3.6)	**309.9 (114.20 to 496.45)**	2.6 (2.0 to 3.8)	7.5 (5.4 to 10.2)	**188.8 (55.68 to 314.80)**	0.7 (0.5 to 0.9)	4.0 (2.7 to 5.6)	**505.1 (243.71 to 802.61)**	2.2 (1.7 to 3.1)	8.5 (6.0 to 11.6)	**296.8 (137.45 to 481.69)**
Scabies	0.8 (0.5 to 1.3)	0.7 (0.4 to 1.1)	**-14.6 (-22.20 to -6.41)**	1.8 (1.0 to 2.8)	1.4 (0.8 to 2.2)	**-21.5 (-28.36 to -13.78)**	0.5 (0.3 to 0.8)	0.4 (0.2 to 0.6)	**-25.1 (-33.88 to -13.75)**	2.2 (1.2 to 3.5)	1.5 (0.8 to 2.4)	**-30.9 (-38.97 to -20.78)**	0.3 (0.2 to 0.5)	0.3 (0.2 to 0.5)	1.7 (-9.62 to 16.09)	1.4 (0.8 to 2.2)	1.3 (0.7 to 2.0)	-6.6 (-17.32 to 5.86)
Fungal skin diseases	19.7 (8.0 to 41.3)	22.9 (9.3 to 47.8)	**16.5 (15.24 to 17.77)**	38.4 (15.5 to 81.8)	38.5 (15.5 to 81.9)	**0.4 (0.04 to 0.70)**	11.9 (4.9 to 25.1)	14.0 (5.7 to 29.3)	**17.9 (16.08 to 19.66)**	48.1 (19.7 to 103.1)	48.2 (19.8 to 103.2)	0.1 (-0.29 to 0.50)	7.8 (3.1 to 16.4)	8.9 (3.6 to 18.7)	**14.4 (13.39 to 15.41)**	29.1 (11.7 to 61.3)	29.1 (11.7 to 61.3)	0.0 (-0.47 to 0.51)
Viral skin diseases	30.0 (17.5 to 47.3)	32.3 (18.9 to 50.3)	**8.0 (5.54 to 10.87)**	70.0 (40.9 to 109.0)	70.3 (41.2 to 109.2)	0.5 (-0.53 to 1.69)	14.5 (8.3 to 22.7)	15.6 (8.9 to 24.5)	**7.9 (4.99 to 10.95)**	68.4 (39.0 to 106.2)	68.3 (39.0 to 106.1)	-0.1 (-1.76 to 1.54)	15.5 (9.0 to 24.4)	16.7 (9.9 to 26.1)	**8.0 (5.27 to 11.41)**	71.4 (42.0 to 110.7)	72.3 (42.7 to 111.7)	1.3 (-0.29 to 2.91)
Acne vulgaris	86.8 (40.8 to 161.2)	73.0 (34.5 to 135.7)	**-15.5 (-25.50 to -0.67)**	200.9 (94.6 to 372.7)	167.1 (79.3 to 309.8)	**-16.5 (-26.46 to -3.08)**	49.2 (22.8 to 92.8)	38.3 (17.9 to 70.9)	**-21.5 (-37.59 to -3.49)**	224.7 (104.1 to 425.0)	173.4 (81.4 to 320.5)	**-22.2 (-38.06 to -6.19)**	37.6 (17.4 to 69.9)	34.7 (16.2 to 64.9)	-7.3 (-21.40 to 9.80)	176.7 (81.6 to 329.9)	160.7 (74.6 to 300.7)	-8.7 (-22.32 to 8.03)
Alopecia areata	2.8 (1.8 to 4.1)	3.2 (2.1 to 4.8)	**18.1 (12.95 to 23.31)**	5.3 (3.4 to 7.8)	5.3 (3.4 to 7.8)	0.1 (-4.04 to 4.31)	1.5 (1.0 to 2.2)	1.8 (1.2 to 2.7)	**21.1 (14.11 to 28.28)**	6.0 (3.9 to 8.9)	5.9 (3.8 to 8.9)	-0.7 (-6.62 to 5.15)	1.3 (0.8 to 1.9)	1.5 (0.9 to 2.2)	**14.6 (6.89 to 23.04)**	4.7 (3.0 to 7.0)	4.7 (3.0 to 6.9)	-0.2 (-6.61 to 6.53)
Pruritus	0.1 (0.0 to 0.1)	0.1 (0.0 to 0.1)	12.5 (-5.48 to 32.59)	0.1 (0.1 to 0.2)	0.1 (0.1 to 0.2)	-6.9 (-21.90 to 9.70)	<0.05 (0.0 to 0.1)	<0.05 (0.0 to 0.1)	13.2 (-22.28 to 47.92)	0.1 (0.0 to 0.2)	0.1 (0.0 to 0.2)	-10.4 (-37.44 to 15.30)	<0.05 (0.0 to 0.1)	<0.05 (0.0 to 0.1)	12.8 (-8.81 to 41.67)	0.1 (0.1 to 0.3)	0.1 (0.1 to 0.3)	-4.0 (-23.07 to 19.97)
Urticaria	23.3 (15.1 to 33.4)	29.2 (18.6 to 42.6)	**25.6 (7.75 to 45.17)**	45.6 (29.2 to 65.8)	49.2 (31.4 to 71.7)	8.2 (-5.53 to 24.53)	12.0 (7.5 to 17.3)	15.6 (9.8 to 23.2)	**30.4 (7.99 to 62.66)**	49.8 (31.1 to 71.5)	53.7 (33.4 to 79.6)	8.1 (-9.18 to 36.64)	11.3 (7.2 to 16.3)	13.6 (8.7 to 19.5)	**21.2 (0.26 to 48.54)**	41.5 (26.2 to 60.7)	44.9 (28.2 to 64.6)	8.9 (-8.96 to 35.88)
Decubitus ulcer	8.9 (7.4 to 10.4)	6.2 (4.7 to 7.9)	**-30.4 (-42.61 to -16.13)**	12.6 (10.5 to 14.9)	6.9 (5.3 to 8.8)	**-44.9 (-54.46 to -33.87)**	3.2 (2.6 to 4.0)	2.6 (1.9 to 3.4)	**-18.1 (-33.70 to 2.37)**	11.8 (9.4 to 14.6)	6.9 (5.1 to 9.0)	**-41.1 (-52.26 to -26.75)**	5.6 (4.6 to 6.6)	3.5 (2.7 to 4.7)	**-37.3 (-50.43 to -17.15)**	12.7 (10.4 to 14.9)	6.8 (5.2 to 9.0)	**-46.4 (-57.33 to -31.39)**
Other skin and subcutaneous diseases	25.1 (10.7 to 55.0)	29.8 (12.3 to 67.1)	**18.3 (12.20 to 22.72)**	44.6 (20.0 to 92.3)	43.9 (19.5 to 91.8)	-1.7 (-4.89 to 0.45)	12.7 (5.5 to 27.5)	15.6 (6.5 to 35.0)	**22.8 (15.34 to 28.82)**	48.5 (21.5 to 101.5)	48.0 (21.2 to 101.5)	-1.4 (-4.16 to 0.57)	12.4 (5.2 to 27.5)	14.2 (5.9 to 31.8)	**13.9 (7.94 to 19.18)**	41.0 (18.6 to 84.1)	40.1 (17.7 to 83.7)	-2.4 (-6.33 to 0.46)
Sense organ diseases	380.9 (257.0 to 539.5)	444.8 (299.3 to 627.9)	**16.9 (12.12 to 21.33)**	603.9 (406.9 to 865.3)	557.8 (372.5 to 800.1)	**-7.6 (-11.60 to -4.56)**	185.7 (125.2 to 266.8)	231.6 (157.7 to 328.5)	**24.9 (19.05 to 32.11)**	702.3 (476.1 to 1,008.4)	640.3 (436.8 to 920.4)	**-8.8 (-13.33 to -4.28)**	195.2 (130.5 to 274.8)	213.2 (141.5 to 301.9)	**9.3 (4.36 to 14.22)**	526.1 (345.7 to 747.9)	485.7 (316.1 to 698.1)	**-7.7 (-11.86 to -3.23)**
Glaucoma	5.8 (3.8 to 8.2)	5.1 (3.2 to 7.3)	-12.0 (-22.26 to 2.36)	8.7 (5.6 to 12.2)	6.0 (3.8 to 8.6)	**-30.3 (-38.49 to -19.63)**	1.9 (1.2 to 2.7)	1.8 (1.1 to 2.5)	-4.2 (-17.68 to 14.03)	6.9 (4.5 to 9.8)	4.8 (3.1 to 6.8)	**-31.1 (-39.72 to -19.31)**	4.0 (2.5 to 5.7)	3.3 (2.0 to 4.8)	**-15.5 (-27.31 to 0.73)**	9.9 (6.4 to 14.1)	7.1 (4.3 to 10.1)	**-28.2 (-38.08 to -16.32)**

Cataract	10.2 (6.6 to 14.8)	9.5 (6.0 to 13.9)	-7.5 (-17.74 to 3.78)	13.7 (8.8 to 19.6)	9.5 (6.1 to 13.9)	**-30.7 (-37.88 to -20.75)**	3.1 (1.9 to 4.6)	3.3 (2.0 to 5.1)	7.1 (-13.52 to 27.00)	11.6 (7.1 to 16.9)	8.0 (4.9 to 12.1)	**-31.0 (-42.06 to -20.11)**	7.1 (4.6 to 10.6)	6.1 (3.9 to 8.9)	**-13.6 (-26.99 to 1.76)**	15.1 (9.8 to 22.1)	10.6 (6.9 to 15.3)	**-29.2 (-39.08 to -15.57)**
Macular degeneration	14.4 (9.3 to 20.5)	17.3 (11.2 to 24.9)	**20.5 (10.66 to 33.72)**	20.2 (13.1 to 28.7)	18.3 (12.0 to 25.9)	**-9.4 (-15.79 to -1.65)**	4.5 (2.9 to 6.5)	6.5 (4.2 to 9.2)	**44.9 (29.26 to 63.24)**	16.7 (10.8 to 24.0)	16.0 (10.5 to 22.9)	-3.8 (-12.67 to 6.49)	9.9 (6.2 to 14.3)	10.9 (7.0 to 15.8)	**9.7 (-1.26 to 24.72)**	22.1 (14.4 to 31.3)	19.9 (13.0 to 28.3)	**-9.8 (-18.22 to 0.12)**
Uncorrected refractive error	78.7 (44.8 to 129.8)	88.9 (50.1 to 149.6)	**12.8 (4.49 to 21.81)**	134.0 (76.1 to 224.0)	123.8 (70.1 to 210.0)	**-7.7 (-14.70 to -0.39)**	39.5 (21.7 to 66.7)	47.4 (25.9 to 82.0)	**20.0 (5.89 to 36.40)**	152.0 (83.8 to 257.3)	141.0 (77.8 to 243.7)	-7.2 (-17.98 to 5.45)	39.2 (22.8 to 63.4)	41.5 (24.2 to 68.1)	5.9 (-3.17 to 14.50)	118.1 (68.8 to 192.8)	107.6 (62.4 to 179.2)	**-8.9 (-16.74 to -0.78)**
Age-related and other hearing loss	230.7 (153.3 to 329.0)	280.5 (187.4 to 401.7)	**21.7 (15.20 to 28.63)**	347.6 (233.9 to 499.2)	326.3 (216.1 to 466.4)	**-6.1 (-11.60 to -1.13)**	119.6 (80.1 to 171.2)	153.9 (103.1 to 218.1)	**28.9 (21.10 to 38.35)**	443.3 (301.0 to 633.1)	404.4 (268.4 to 576.3)	**-8.7 (-14.61 to -3.07)**	111.0 (71.6 to 161.1)	126.6 (81.9 to 184.0)	**14.1 (5.98 to 22.00)**	273.5 (177.6 to 401.2)	258.9 (166.5 to 384.5)	-5.3 (-12.64 to 1.90)
Other vision loss	10.8 (7.2 to 15.0)	9.1 (6.0 to 13.2)	**-16.0 (-22.79 to -7.24)**	17.6 (11.8 to 24.7)	11.9 (8.0 to 17.3)	**-32.3 (-37.56 to -25.39)**	5.0 (3.3 to 7.2)	4.6 (3.1 to 6.7)	-7.4 (-19.24 to 6.71)	19.0 (12.9 to 26.8)	13.3 (8.9 to 19.1)	**-30.3 (-37.95 to -20.53)**	5.8 (3.8 to 8.3)	4.5 (2.9 to 6.6)	**-23.2 (-30.27 to -15.95)**	16.6 (10.9 to 23.4)	10.8 (7.1 to 15.8)	**-35.0 (-41.59 to -28.37)**
Other sense organ diseases	30.3 (18.8 to 44.9)	34.5 (21.6 to 50.5)	**13.8 (10.92 to 16.65)**	62.1 (38.4 to 92.4)	61.9 (38.5 to 91.4)	-0.2 (-2.63 to 2.31)	12.1 (7.5 to 17.9)	14.1 (8.8 to 20.8)	**16.8 (12.60 to 20.71)**	52.7 (32.8 to 78.3)	52.9 (32.6 to 78.1)	0.2 (-3.38 to 3.69)	18.2 (11.2 to 27.0)	20.3 (12.7 to 29.7)	**11.9 (8.34 to 15.75)**	71.0 (43.8 to 105.8)	70.8 (44.2 to 104.3)	-0.2 (-3.25 to 3.26)
Oral disorders	174.0 (109.9 to 256.3)	191.2 (120.8 to 284.1)	**9.8 (5.86 to 13.79)**	290.2 (181.9 to 433.1)	265.6 (165.1 to 401.4)	**-8.6 (-11.84 to -5.32)**	75.4 (46.5 to 112.9)	87.6 (54.2 to 131.4)	**16.1 (11.16 to 21.18)**	281.6 (173.1 to 423.6)	257.5 (157.8 to 392.6)	**-8.7 (-12.69 to -4.41)**	98.7 (63.7 to 143.0)	103.7 (66.3 to 152.2)	**5.0 (0.18 to 9.60)**	297.6 (189.5 to 441.3)	273.3 (172.4 to 411.4)	**-8.3 (-12.27 to -4.51)**
Deciduous caries	0.4 (0.2 to 0.8)	0.4 (0.2 to 0.8)	3.0 (-1.79 to 7.56)	1.2 (0.5 to 2.3)	1.2 (0.5 to 2.3)	-0.6 (-5.14 to 3.89)	0.2 (0.1 to 0.4)	0.2 (0.1 to 0.4)	3.5 (-2.91 to 10.26)	1.2 (0.6 to 2.4)	1.2 (0.6 to 2.4)	-0.0 (-6.23 to 6.45)	0.2 (0.1 to 0.4)	0.2 (0.1 to 0.4)	2.3 (-4.37 to 9.45)	1.1 (0.5 to 2.2)	1.1 (0.5 to 2.2)	-1.1 (-7.59 to 5.75)
Permanent caries	10.6 (4.8 to 20.3)	12.0 (5.5 to 22.9)	**13.4 (8.19 to 18.43)**	22.1 (10.1 to 42.5)	22.4 (10.2 to 42.8)	1.4 (-3.21 to 5.77)	5.2 (2.4 to 10.1)	6.0 (2.8 to 11.5)	**15.3 (4.22 to 23.99)**	22.2 (10.0 to 43.0)	22.7 (10.4 to 43.5)	2.3 (-7.13 to 10.02)	5.4 (2.5 to 10.5)	6.0 (2.7 to 11.6)	**11.7 (5.00 to 21.40)**	22.0 (10.1 to 42.8)	22.2 (10.0 to 43.3)	0.6 (-5.42 to 8.48)
Periodontal diseases	31.4 (12.6 to 64.1)	39.8 (15.7 to 81.8)	**26.5 (19.54 to 35.85)**	56.4 (22.5 to 115.1)	58.3 (23.1 to 120.0)	3.3 (-2.35 to 10.65)	17.6 (7.0 to 36.2)	22.1 (8.8 to 46.0)	**25.7 (15.09 to 38.88)**	66.8 (26.4 to 137.3)	67.1 (26.6 to 139.0)	0.4 (-7.85 to 10.69)	13.8 (5.6 to 28.1)	17.7 (7.0 to 36.2)	**27.8 (19.28 to 38.44)**	46.9 (19.0 to 95.6)	49.9 (19.8 to 102.4)	**6.4 (-0.48 to 15.06)**
Edentulism and severe tooth loss	105.3 (71.0 to 143.6)	109.1 (74.6 to 150.0)	3.6 (-1.75 to 9.42)	158.5 (106.4 to 216.7)	131.7 (90.1 to 181.5)	**-16.9 (-21.28 to -12.31)**	41.3 (27.6 to 56.8)	46.5 (31.5 to 64.1)	**12.6 (5.30 to 20.61)**	146.6 (98.1 to 201.5)	121.8 (82.4 to 168.3)	**-16.9 (-22.32 to -11.03)**	64.0 (43.5 to 87.7)	62.6 (42.7 to 86.4)	-2.2 (-8.36 to 4.02)	168.6 (114.6 to 231.9)	140.9 (95.8 to 194.8)	**-16.4 (-21.67 to -10.93)**
Other oral disorders	26.2 (16.6 to 38.7)	29.9 (18.8 to 44.5)	**13.9 (10.84 to 17.16)**	51.9 (32.9 to 76.6)	52.0 (32.7 to 77.5)	0.1 (-2.66 to 2.99)	11.0 (7.0 to 16.3)	12.7 (8.1 to 18.8)	**15.3 (10.92 to 19.80)**	44.8 (28.5 to 66.3)	44.7 (28.4 to 66.3)	-0.2 (-3.91 to 3.88)	15.2 (9.6 to 22.5)	17.2 (10.8 to 25.6)	**13.0 (9.12 to 17.08)**	58.9 (37.2 to 87.2)	59.2 (37.1 to 88.1)	0.4 (-3.15 to 4.08)
Sudden infant death syndrome	63.4 (33.0 to 78.9)	19.4 (12.0 to 36.6)	**-66.2 (-83.19 to -23.74)**	179.6 (93.4 to 223.6)	53.9 (33.4 to 101.5)	**-66.9 (-83.54 to -25.32)**	38.5 (18.0 to 46.6)	10.8 (7.1 to 20.9)	**-69.0 (-83.01 to -27.57)**	213.1 (99.4 to 257.9)	58.8 (38.6 to 113.1)	**-69.6 (-83.37 to -29.09)**	24.9 (11.0 to 35.2)	8.6 (4.5 to 18.9)	**-60.9 (-83.95 to -8.73)**	144.5 (64.1 to 204.2)	48.9 (25.7 to 107.3)	**-61.7 (-84.28 to -10.63)**
**Injuries**	1,166.1 (1,036.4 to 1,323.9)	835.6 (708.5 to 985.1)	**-28.4 (-33.41 to -22.77)**	2,167.9 (1,948.5 to 2,432.0)	1,283.2 (1,113.9 to 1,484.2)	**-40.8 (-44.54 to -36.73)**	728.4 (657.9 to 811.1)	526.2 (453.1 to 611.2)	**-27.8 (-32.67 to -22.36)**	2,973.7 (2,699.1 to 3,290.8)	1,765.8 (1,541.8 to 2,028.1)	**-40.6 (-44.62 to -36.36)**	437.7 (374.7 to 511.4)	309.4 (252.2 to 376.4)	**-29.4 (-36.38 to -22.03)**	1,339.2 (1,171.8 to 1,535.5)	798.7 (668.2 to 951.4)	**-40.4 (-45.19 to -35.08)**
**Transport injuries**	298.1 (275.4 to 321.9)	142.5 (127.6 to 158.5)	**-52.2 (-55.21 to -48.87)**	623.3 (579.3 to 668.7)	263.3 (236.8 to 289.3)	**-57.7 (-60.50 to -54.48)**	228.4 (211.9 to 245.3)	110.1 (98.6 to 122.9)	**-51.8 (-55.18 to -47.79)**	967.1 (901.0 to 1,035.2)	410.8 (369.2 to 454.7)	**-57.5 (-60.59 to -53.89)**	69.8 (59.3 to 77.5)	32.3 (27.6 to 37.6)	**-53.7 (-57.11 to -49.21)**	282.0 (240.1 to 312.3)	116.2 (100.2 to 135.1)	**-58.8 (-62.08 to -54.69)**
Road injuries	285.5 (263.8 to 307.8)	129.2 (116.2 to 142.3)	**-54.8 (-57.61 to -51.50)**	598.4 (556.7 to 641.3)	241.7 (218.4 to 264.4)	**-59.6 (-62.23 to -56.35)**	218.8 (202.9 to 234.7)	99.6 (89.5 to 110.6)	**-54.5 (-57.63 to -50.65)**	928.2 (864.1 to 989.8)	376.0 (339.2 to 415.2)	**-59.5 (-62.37 to -55.86)**	66.8 (56.7 to 74.1)	29.5 (25.2 to 34.5)	**-55.8 (-59.18 to -51.49)**	270.8 (230.9 to 299.7)	107.4 (92.8 to 125.9)	**-60.3 (-63.77 to -56.37)**
Pedestrian road injuries	66.2 (52.2 to 82.3)	26.5 (21.1 to 34.3)	**-60.0 (-63.42 to -55.15)**	139.0 (109.2 to 173.3)	47.7 (37.7 to 62.8)	**-65.7 (-68.90 to -61.34)**	47.0 (33.6 to 62.5)	19.5 (14.3 to 26.6)	**-58.5 (-62.90 to -52.21)**	202.9 (144.5 to 269.3)	71.2 (51.3 to 98.6)	**-64.9 (-68.73 to -59.45)**	19.1 (14.7 to 23.9)	7.0 (5.3 to 9.0)	**-63.4 (-67.09 to -57.69)**	76.6 (57.9 to 96.9)	24.5 (18.2 to 31.6)	**-68.0 (-71.53 to -63.08)**
Cyclist road injuries	20.4 (16.8 to 24.2)	8.2 (6.5 to 9.8)	**-59.9 (-64.66 to -55.10)**	44.8 (36.6 to 53.3)	15.6 (12.1 to 18.6)	**-65.3 (-69.69 to -60.89)**	15.9 (12.6 to 19.5)	6.3 (4.7 to 7.8)	**-60.4 (-66.00 to -54.35)**	71.1 (56.0 to 87.6)	24.0 (17.8 to 29.8)	**-66.2 (-71.27 to -60.92)**	4.5 (3.7 to 5.5)	1.9 (1.4 to 2.4)	**-57.9 (-63.89 to -51.88)**	18.8 (15.4 to 22.8)	7.1 (5.3 to 9.0)	**-62.1 (-67.80 to -56.51)**
Motorcyclist road injuries	55.8 (44.1 to 68.8)	24.4 (18.3 to 29.7)	**-56.2 (-62.50 to -50.62)**	117.3 (91.9 to 145.1)	47.2 (34.8 to 58.3)	**-59.7 (-65.91 to -54.38)**	51.1 (39.5 to 63.6)	22.9 (17.0 to 28.2)	**-55.0 (-61.90 to -48.92)**	214.7 (163.8 to 267.9)	88.4 (64.7 to 109.1)	**-58.8 (-65.50 to -53.00)**	4.7 (3.0 to 7.5)	1.5 (0.8 to 2.5)	**-68.9 (-74.28 to -63.62)**	19.6 (12.0 to 31.2)	5.7 (3.0 to 9.7)	**-71.1 (-76.80 to -65.79)**
Motor vehicle road injuries	141.2 (121.7 to 158.8)	69.3 (58.9 to 78.3)	**-50.9 (-54.58 to -46.28)**	293.3 (252.4 to 330.4)	130.0 (110.7 to 146.1)	**-55.6 (-59.20 to -51.13)**	103.5 (85.0 to 119.0)	50.6 (41.0 to 58.4)	**-51.1 (-55.73 to -45.60)**	434.4 (355.6 to 500.2)	191.2 (154.0 to 220.4)	**-55.9 (-60.41 to -50.53)**	37.6 (30.4 to 43.6)	18.7 (15.3 to 22.0)	**-50.2 (-54.56 to -45.34)**	152.8 (123.3 to 177.3)	68.6 (55.7 to 80.2)	**-55.1 (-59.23 to -50.40)**
Other road injuries	2.0 (1.3 to 2.4)	0.7 (0.6 to 1.1)	**-63.0 (-69.81 to -45.82)**	4.1 (2.7 to 5.0)	1.3 (1.0 to 2.0)	**-67.5 (-73.72 to -53.01)**	1.2 (0.6 to 1.6)	0.3 (0.2 to 0.5)	**-73.5 (-79.61 to -57.80)**	5.1 (2.7 to 6.6)	1.2 (0.8 to 2.0)	**-76.7 (-82.11 to -63.31)**	0.8 (0.6 to 1.0)	0.4 (0.3 to 0.6)	**-46.0 (-57.28 to -28.18)**	3.0 (2.2 to 4.1)	1.4 (0.9 to 2.1)	**-52.2 (-62.82 to -36.14)**
Other transport injuries	12.6 (10.7 to 14.7)	13.3 (10.7 to 16.5)	5.4 (-6.43 to 16.76)	24.8 (21.3 to 29.0)	21.6 (17.6 to 26.4)	**-13.1 (-22.30 to -4.04)**	9.6 (8.2 to 11.4)	10.5 (8.4 to 13.1)	9.3 (-4.91 to 22.98)	38.9 (33.3 to 46.0)	34.9 (27.9 to 43.0)	**-10.5 (-22.26 to 0.51)**	3.0 (2.3 to 3.8)	2.8 (2.1 to 3.6)	-6.9 (-16.73 to 5.20)	11.2 (8.5 to 14.5)	8.8 (6.7 to 11.6)	**-21.1 (-29.64 to -10.02)**
**Unintentional injuries**	620.5 (511.4 to 752.5)	538.1 (426.7 to 674.7)	**-13.3 (-21.31 to -5.51)**	1,047.5 (875.2 to 1,258.7)	741.1 (594.4 to 920.7)	**-29.3 (-34.83 to -23.68)**	316.2 (263.4 to 380.1)	296.0 (237.2 to 370.7)	**-6.5 (-13.07 to 0.11)**	1,259.8 (1,059.8 to 1,509.7)	919.6 (749.1 to 1,140.0)	**-27.1 (-32.04 to -22.05)**	304.3 (246.0 to 371.2)	242.1 (188.0 to 306.7)	**-20.4 (-30.09 to -10.49)**	807.5 (664.2 to 977.5)	559.8 (440.7 to 703.6)	**-30.7 (-37.66 to -23.39)**
Falls	428.8 (338.7 to 530.7)	390.6 (296.3 to 499.7)	-8.9 (-20.43 to 2.17)	667.4 (528.0 to 828.2)	493.6 (374.3 to 634.9)	**-26.1 (-34.70 to -17.59)**	181.6 (144.7 to 225.5)	196.7 (150.8 to 251.9)	8.3 (-3.51 to 18.67)	699.9 (558.1 to 867.9)	573.4 (439.4 to 734.4)	**-18.1 (-26.87 to -10.20)**	247.2 (194.1 to 307.0)	193.9 (145.7 to 250.2)	**-21.5 (-33.34 to -9.57)**	600.2 (471.1 to 750.2)	408.9 (304.3 to 531.1)	**-31.9 (-41.08 to -22.60)**
Drowning	16.8 (13.8 to 19.4)	9.6 (7.7 to 11.9)	**-42.8 (-50.52 to -32.51)**	39.0 (31.9 to 44.9)	20.0 (16.0 to 24.8)	**-48.7 (-56.06 to -39.73)**	13.2 (10.4 to 15.6)	7.6 (5.9 to 9.6)	**-42.0 (-50.70 to -31.54)**	60.9 (47.9 to 72.1)	31.7 (24.3 to 39.7)	**-47.9 (-55.89 to -39.05)**	3.6 (3.0 to 4.3)	1.9 (1.5 to 2.6)	**-45.3 (-55.62 to -32.59)**	16.7 (13.8 to 20.0)	8.1 (6.1 to 10.7)	**-51.6 (-61.22 to -39.43)**
Fire, heat, and hot substances	28.6 (23.6 to 33.8)	16.3 (12.8 to 21.6)	**-42.9 (-51.21 to -21.68)**	58.0 (48.3 to 67.8)	27.3 (21.7 to 35.8)	**-52.8 (-59.84 to -34.12)**	16.3 (13.3 to 19.5)	9.5 (7.3 to 12.8)	**-41.3 (-50.57 to -16.26)**	69.7 (56.6 to 83.0)	33.4 (26.0 to 44.6)	**-52.0 (-59.69 to -30.49)**	12.3 (9.6 to 15.0)	6.8 (5.3 to 9.0)	**-44.9 (-52.90 to -25.91)**	46.8 (36.5 to 55.7)	21.4 (17.1 to 28.1)	**-54.1 (-61.08 to -37.03)**
Poisonings	18.5 (11.6 to 20.5)	6.2 (5.0 to 11.2)	**-64.8 (-74.78 to -27.13)**	38.3 (23.9 to 42.4)	11.4 (9.1 to 20.6)	**-69.0 (-77.70 to -36.38)**	13.1 (6.8 to 14.6)	3.7 (2.9 to 7.8)	**-69.8 (-78.91 to -29.65)**	54.9 (28.4 to 61.2)	13.9 (11.0 to 29.2)	**-73.0 (-81.08 to -37.53)**	5.4 (3.1 to 6.1)	2.5 (1.9 to 4.5)	**-51.4 (-66.62 to -13.69)**	21.8 (12.4 to 24.7)	8.8 (6.7 to 15.8)	**-57.9 (-71.10 to -26.09)**
Exposure to mechanical forces	56.8 (45.5 to 71.2)	53.2 (41.9 to 67.6)	**-6.2 (-17.15 to -0.13)**	114.2 (92.1 to 142.1)	94.0 (75.5 to 116.9)	**-17.7 (-28.68 to -11.36)**	48.5 (39.0 to 61.0)	41.6 (32.7 to 52.7)	**-14.1 (-23.02 to -8.71)**	198.4 (160.3 to 248.1)	147.7 (117.9 to 184.1)	**-25.5 (-34.72 to -20.55)**	8.3 (6.6 to 10.5)	11.6 (8.9 to 14.8)	**40.1 (13.92 to 56.13)**	33.4 (26.7 to 42.3)	41.3 (32.0 to 51.8)	24.4 (-3.92 to 41.19)
Unintentional firearm injuries	1.4 (1.3 to 1.6)	0.7 (0.6 to 0.9)	**-47.2 (-52.14 to -41.92)**	3.0 (2.7 to 3.4)	1.5 (1.3 to 1.7)	**-52.1 (-56.69 to -47.24)**	1.1 (1.0 to 1.3)	0.5 (0.4 to 0.6)	**-58.3 (-62.75 to -52.86)**	4.9 (4.4 to 5.5)	1.8 (1.6 to 2.2)	**-62.5 (-66.61 to -57.77)**	0.3 (0.2 to 0.3)	0.3 (0.2 to 0.3)	0.2 (-21.23 to 12.34)	1.2 (1.1 to 1.3)	1.1 (0.9 to 1.2)	-9.2 (-31.98 to 2.75)
Unintentional suffocation	2.7 (2.3 to 4.8)	8.4 (4.2 to 9.9)	**224.9 (7.07 to 304.71)**	6.5 (5.4 to 11.7)	18.4 (9.0 to 22.0)	**199.9 (-3.79 to 279.79)**	1.8 (1.5 to 3.5)	6.1 (2.6 to 7.3)	**263.8 (-0.89 to 350.01)**	8.4 (7.0 to 16.7)	26.4 (10.8 to 32.0)	**234.9 (-12.34 to 319.30)**	0.9 (0.7 to 2.0)	2.3 (1.0 to 2.9)	163.3 (-30.30 to 254.57)	4.6 (3.6 to 10.2)	10.2 (4.1 to 13.3)	145.3 (-42.10 to 240.64)
Other exposure to mechanical forces	52.6 (41.6 to 67.0)	44.1 (33.2 to 58.3)	**-16.3 (-22.22 to -10.04)**	104.7 (83.6 to 131.8)	74.2 (56.7 to 96.8)	**-29.3 (-34.17 to -24.24)**	45.6 (36.1 to 58.0)	35.1 (26.4 to 46.4)	**-23.2 (-28.93 to -17.28)**	185.1 (147.4 to 233.8)	119.4 (91.3 to 156.5)	**-35.6 (-40.24 to -30.84)**	7.1 (5.6 to 9.0)	9.1 (6.8 to 12.0)	**27.8 (15.09 to 39.42)**	27.6 (21.8 to 34.9)	30.1 (22.9 to 39.2)	8.9 (-3.51 to 19.50)
Adverse effects of medical treatment	18.4 (14.4 to 20.9)	15.1 (12.5 to 19.4)	-17.4 (-30.03 to 6.56)	32.0 (25.2 to 36.5)	21.7 (17.7 to 28.1)	**-31.9 (-42.33 to -12.10)**	7.9 (6.1 to 10.0)	7.3 (5.4 to 9.6)	-7.5 (-28.49 to 15.46)	31.4 (24.3 to 39.6)	22.6 (16.6 to 29.6)	**-27.6 (-44.30 to -9.75)**	10.4 (7.2 to 12.2)	7.8 (6.2 to 11.1)	-24.4 (-41.28 to 4.00)	32.4 (22.5 to 37.7)	20.8 (16.6 to 29.9)	**-35.1 (-48.78 to -10.69)**
Animal contact	2.5 (1.9 to 3.2)	3.4 (2.6 to 4.4)	**39.7 (17.26 to 51.04)**	4.9 (3.8 to 6.3)	5.7 (4.5 to 7.4)	**18.1 (-2.76 to 28.90)**	1.3 (1.0 to 1.6)	1.9 (1.5 to 2.4)	**50.1 (21.45 to 62.28)**	5.3 (4.2 to 6.8)	6.5 (5.0 to 8.2)	**23.3 (-1.66 to 33.84)**	1.2 (0.9 to 1.6)	1.5 (1.1 to 2.0)	**28.8 (7.70 to 41.47)**	4.5 (3.4 to 6.4)	5.1 (3.7 to 6.7)	12.3 (-7.83 to 25.04)
Venomous animal contact	0.5 (0.4 to 0.7)	0.6 (0.5 to 0.8)	**23.4 (-0.82 to 41.82)**	1.1 (0.9 to 1.6)	1.2 (0.9 to 1.4)	7.0 (-15.17 to 24.14)	0.3 (0.2 to 0.4)	0.4 (0.3 to 0.4)	**31.1 (-1.98 to 47.67)**	1.1 (0.9 to 1.5)	1.3 (1.0 to 1.5)	12.1 (-17.90 to 27.55)	0.3 (0.2 to 0.4)	0.3 (0.2 to 0.4)	15.7 (-8.41 to 43.57)	1.0 (0.7 to 1.8)	1.1 (0.7 to 1.5)	1.8 (-21.54 to 28.90)
Non-venomous animal contact	1.9 (1.5 to 2.5)	2.8 (2.1 to 3.7)	**44.2 (22.26 to 55.06)**	3.8 (2.9 to 5.0)	4.6 (3.5 to 6.0)	**21.3 (1.01 to 31.88)**	1.0 (0.8 to 1.3)	1.6 (1.2 to 2.0)	**55.3 (26.33 to 67.91)**	4.1 (3.2 to 5.3)	5.2 (3.9 to 6.7)	**26.4 (1.47 to 37.06)**	0.9 (0.7 to 1.3)	1.2 (0.9 to 1.7)	**32.5 (12.37 to 46.36)**	3.5 (2.6 to 4.9)	4.0 (3.0 to 5.5)	15.6 (-3.59 to 29.81)
Foreign body	20.3 (16.3 to 30.5)	24.2 (15.7 to 29.5)	22.1 (-16.58 to 54.55)	42.2 (33.8 to 63.8)	41.2 (27.4 to 51.4)	-0.2 (-29.74 to 25.92)	13.4 (9.4 to 22.1)	14.6 (8.0 to 19.2)	11.8 (-27.18 to 41.33)	58.1 (41.0 to 95.4)	52.2 (29.3 to 70.3)	-8.0 (-38.64 to 16.21)	6.9 (5.1 to 10.8)	9.6 (5.7 to 13.1)	**42.9 (-1.56 to 92.47)**	26.8 (20.0 to 42.4)	30.5 (18.6 to 42.7)	16.6 (-18.26 to 56.84)
Pulmonary aspiration and foreign body in airway	17.7 (13.7 to 28.1)	21.6 (13.3 to 26.8)	26.1 (-17.68 to 63.45)	37.0 (28.7 to 58.9)	36.8 (23.4 to 47.3)	2.2 (-31.35 to 31.70)	11.7 (7.9 to 20.4)	12.9 (6.3 to 17.4)	13.5 (-30.41 to 46.59)	51.3 (34.5 to 89.2)	46.3 (23.0 to 63.3)	-7.1 (-41.25 to 20.00)	5.9 (4.2 to 9.8)	8.7 (4.8 to 12.1)	**52.1 (0.09 to 109.74)**	23.1 (16.5 to 38.8)	27.6 (15.9 to 38.8)	23.0 (-17.65 to 70.52)
Foreign body in eyes	0.4 (0.2 to 0.6)	0.4 (0.2 to 0.6)	-1.8 (-9.89 to 4.75)	0.8 (0.5 to 1.2)	0.7 (0.4 to 1.1)	**-12.9 (-20.87 to -6.49)**	0.2 (0.1 to 0.4)	0.2 (0.1 to 0.4)	1.8 (-5.27 to 8.16)	1.0 (0.6 to 1.5)	0.9 (0.5 to 1.4)	**-11.1 (-18.78 to -4.87)**	0.1 (0.1 to 0.2)	0.1 (0.1 to 0.2)	**-7.8 (-17.91 to 1.10)**	0.6 (0.3 to 0.9)	0.5 (0.3 to 0.8)	**-16.6 (-26.44 to -7.96)**
Foreign body in other body part	2.2 (1.6 to 3.0)	2.2 (1.5 to 3.1)	-2.4 (-12.05 to 6.54)	4.4 (3.2 to 6.1)	3.7 (2.5 to 5.3)	**-16.1 (-24.79 to -8.25)**	1.4 (1.0 to 2.0)	1.5 (1.0 to 2.1)	1.4 (-8.75 to 10.32)	5.9 (4.1 to 8.2)	5.0 (3.3 to 7.2)	**-14.2 (-23.46 to -6.23)**	0.8 (0.6 to 1.1)	0.7 (0.5 to 1.0)	**-8.9 (-21.41 to 1.06)**	3.1 (2.3 to 4.1)	2.5 (1.8 to 3.4)	**-20.6 (-31.95 to -11.79)**
Other unintentional injuries	29.9 (22.7 to 38.6)	19.5 (14.5 to 26.0)	**-35.1 (-39.06 to -31.35)**	51.5 (40.1 to 65.6)	26.2 (19.9 to 34.4)	**-49.2 (-52.24 to -46.23)**	20.8 (15.9 to 26.8)	12.9 (9.7 to 17.2)	**-38.0 (-42.18 to -33.48)**	81.1 (63.0 to 103.5)	38.2 (29.1 to 50.2)	**-53.0 (-56.04 to -49.56)**	9.1 (6.8 to 12.0)	6.5 (4.8 to 8.8)	**-28.5 (-33.77 to -22.43)**	25.0 (19.2 to 32.3)	14.9 (11.2 to 19.6)	**-40.5 (-45.48 to -35.38)**
**Self-harm and interpersonal violence**	247.3 (216.9 to 262.3)	154.9 (136.3 to 181.0)	**-37.3 (-43.98 to -26.26)**	496.5 (436.2 to 527.7)	278.6 (245.7 to 326.0)	**-43.9 (-49.93 to -33.80)**	183.7 (150.9 to 199.5)	120.0 (101.4 to 144.6)	**-34.6 (-43.69 to -20.92)**	746.1 (612.5 to 808.0)	435.2 (368.4 to 527.5)	**-41.6 (-49.71 to -29.23)**	63.5 (57.0 to 69.9)	34.9 (30.8 to 42.1)	**-45.0 (-51.02 to -37.23)**	249.2 (223.9 to 276.9)	122.7 (108.7 to 147.4)	**-50.8 (-56.21 to -44.01)**
Self-harm	226.0 (197.4 to 243.3)	143.9 (125.6 to 169.6)	**-36.3 (-43.41 to -24.50)**	450.8 (392.5 to 484.7)	257.1 (225.2 to 302.1)	**-42.9 (-49.51 to -32.25)**	168.9 (138.5 to 185.0)	112.5 (94.4 to 134.9)	**-33.3 (-42.90 to -19.48)**	683.1 (559.3 to 747.7)	406.2 (343.5 to 489.0)	**-40.4 (-49.01 to -27.65)**	57.0 (50.1 to 62.7)	31.4 (27.7 to 38.6)	**-44.9 (-51.27 to -36.08)**	220.6 (193.8 to 242.8)	108.7 (96.0 to 133.5)	**-50.7 (-56.54 to -42.82)**
Interpersonal violence	21.3 (17.2 to 27.5)	11.0 (8.6 to 14.1)	**-48.1 (-53.93 to -37.53)**	45.7 (37.0 to 59.5)	21.5 (16.7 to 27.7)	**-52.9 (-58.43 to -42.85)**	14.8 (10.6 to 20.3)	7.5 (5.5 to 10.6)	**-49.0 (-55.16 to -35.03)**	63.0 (45.0 to 87.0)	29.0 (20.8 to 41.2)	**-53.9 (-59.55 to -41.15)**	6.5 (5.3 to 8.6)	3.5 (2.5 to 4.4)	**-45.8 (-56.03 to -33.88)**	28.6 (23.3 to 38.4)	14.0 (10.0 to 17.9)	**-50.8 (-60.63 to -39.11)**
Assault by firearm	3.4 (2.3 to 5.0)	1.9 (1.2 to 2.9)	**-44.3 (-60.74 to -20.85)**	7.1 (4.8 to 10.4)	3.7 (2.3 to 5.7)	**-48.2 (-63.82 to -26.33)**	2.7 (1.7 to 4.3)	1.5 (0.8 to 2.5)	**-45.7 (-64.57 to -16.31)**	11.3 (7.0 to 17.9)	5.7 (3.1 to 9.7)	**-49.5 (-67.45 to -21.70)**	0.7 (0.4 to 1.0)	0.4 (0.2 to 0.6)	**-38.3 (-54.49 to -20.72)**	3.0 (1.8 to 4.5)	1.7 (0.9 to 2.6)	**-43.2 (-58.83 to -26.80)**
Assault by sharp object	2.9 (2.2 to 4.3)	2.1 (1.5 to 3.1)	**-26.0 (-45.15 to -7.91)**	6.1 (4.6 to 9.1)	4.2 (2.9 to 6.3)	**-30.6 (-48.74 to -13.05)**	2.0 (1.2 to 3.2)	1.4 (0.9 to 2.4)	**-25.2 (-45.90 to 3.71)**	8.2 (5.2 to 13.5)	5.7 (3.4 to 9.7)	**-29.8 (-49.47 to -2.63)**	0.9 (0.7 to 1.5)	0.7 (0.4 to 1.0)	**-26.7 (-50.58 to -7.35)**	4.0 (2.9 to 6.2)	2.7 (1.6 to 4.1)	**-31.7 (-54.24 to -13.03)**
Assault by other means	15.0 (11.9 to 18.7)	7.0 (5.5 to 9.1)	**-53.1 (-58.52 to -41.71)**	32.5 (25.8 to 40.9)	13.6 (10.6 to 17.8)	**-58.0 (-63.09 to -47.64)**	10.1 (7.2 to 13.6)	4.6 (3.4 to 6.6)	**-54.3 (-60.39 to -39.85)**	43.5 (30.8 to 59.1)	17.6 (12.7 to 25.4)	**-59.3 (-64.89 to -46.52)**	4.9 (4.0 to 6.5)	2.4 (1.7 to 3.1)	**-50.4 (-59.78 to -37.97)**	21.6 (17.7 to 29.3)	9.6 (6.8 to 12.5)	**-55.3 (-64.59 to -43.49)**
**Forces of nature, war, and legal intervention**	0.2 (0.1 to 0.5)	0.1 (0.0 to 0.1)	**-66.9 (-89.07 to -52.10)**	0.5 (0.3 to 1.3)	0.1 (0.1 to 0.2)	**-73.9 (-91.88 to -61.94)**	0.1 (0.1 to 0.3)	<0.05 (0.0 to 0.1)	**-64.0 (-89.37 to -45.37)**	0.6 (0.3 to 1.7)	0.2 (0.1 to 0.2)	**-71.2 (-91.89 to -55.03)**	0.1 (0.0 to 0.2)	<0.05 (0.0 to <0.05)	**-67.5 (-91.94 to -47.36)**	0.4 (0.2 to 1.4)	0.1 (0.0 to 0.1)	**-74.5 (-93.97 to -58.10)**
Exposure to forces of nature	<0.05 (0.0 to <0.05)	<0.05 (0.0 to <0.05)	**-82.1 (-92.88 to -60.59)**	<0.05 (0.0 to 0.1)	<0.05 (0.0 to <0.05)	**-85.1 (-94.11 to -66.76)**	<0.05 (0.0 to <0.05)	<0.05 (0.0 to <0.05)	**-82.6 (-93.20 to -63.17)**	0.1 (0.0 to 0.1)	<0.05 (0.0 to <0.05)	**-85.5 (-94.35 to -69.23)**	<0.05 (0.0 to <0.05)	<0.05 (0.0 to <0.05)	**-55.9 (-92.30 to -47.52)**	<0.05 (0.0 to <0.05)	<0.05 (0.0 to <0.05)	**-51.5 (-93.62 to -54.58)**
Collective violence and legal intervention	0.2 (0.1 to 0.4)	0.1 (0.0 to 0.1)	**-65.3 (-89.26 to -49.89)**	0.5 (0.3 to 1.2)	0.1 (0.1 to 0.2)	**-72.7 (-91.96 to -60.10)**	0.1 (0.1 to 0.3)	<0.05 (0.0 to 0.1)	**-61.9 (-89.39 to -40.53)**	0.6 (0.3 to 1.7)	0.1 (0.1 to 0.2)	**-69.4 (-92.00 to -50.94)**	0.1 (0.0 to 0.2)	<0.05 (0.0 to <0.05)	**-66.2 (-92.15 to -45.22)**	0.4 (0.2 to 1.4)	0.1 (0.0 to 0.1)	**-73.6 (-94.22 to -56.62)**

Web Table 8: DALYs (in thousands) attributable-risk factors or risk factor clusters and median percent change of age-standardized DALYs PAFs in England for both sexes, males, and females.

	1990 DALYs (in thousands) Male	2013 DALYs (in thousands) Male	median percent change of age-standardized DALYs PAF Male	1990 DALYs (in thousands) Female	2013 DALYs (in thousands) Female	median percent change of age-standardized DALYs PAF Female	1990 DALYs (in thousands) Both sexes	2013 DALYs (in thousands) Both sexes	median percent change of age-standardized DALYs PAF Both sexes
All risk factors	4,178<br>(3,942 - 4,431)	3,032<br>(2,733 - 3,363)	-19.02%<br>(-21.32% - -16.90%)	3,086<br>(2,849 - 3,338)	2,385<br>(2,122 - 2,670)	-15.60%<br>(-18.09% - -12.64%)	7,264<br>(6,793 - 7,759)	5,417<br>(4,872 - 5,987)	-17.80%<br>(-19.56% - -16.14%)
Environmental risks	654<br>(598 - 720)	421<br>(360 - 480)	-27.37%<br>(-36.72% - -19.04%)	350<br>(319 - 381)	227<br>(195 - 260)	-26.79%<br>(-33.14% - -20.29%)	1,005<br>(927 - 1,092)	648<br>(565 - 730)	-27.99%<br>(-34.49% - -22.36%)
Unsafe water, sanitation, and handwashing	2<br>(1 - 2)	3<br>(2 - 4)	104.78%<br>(67.07% - 142.24%)	1<br>(1 - 2)	4<br>(3 - 5)	113.28%<br>(66.78% - 165.72%)	3<br>(2 - 4)	7<br>(5 - 9)	107.84%<br>(70.75% - 149.71%)
Unsafe water source									
Unsafe sanitation									
No handwashing with soap	2<br>(1 - 2)	3<br>(2 - 4)	104.78%<br>(67.07% - 142.24%)	1<br>(1 - 2)	4<br>(3 - 5)	113.28%<br>(66.78% - 165.72%)	3<br>(2 - 4)	7<br>(5 - 9)	107.84%<br>(70.75% - 149.71%)
Air pollution	400<br>(388 - 411)	141<br>(128 - 155)	-62.66%<br>(-65.50% - -59.75%)	244<br>(227 - 254)	101<br>(90 - 113)	-57.81%<br>(-61.82% - -52.75%)	644<br>(620 - 662)	242<br>(220 - 265)	-61.22%<br>(-64.06% - -58.24%)
Ambient particulate matter pollution	397<br>(385 - 409)	132<br>(119 - 146)	-64.82%<br>(-67.61% - -61.98%)	242<br>(225 - 252)	93<br>(83 - 106)	-60.75%<br>(-64.71% - -55.66%)	639<br>(616 - 658)	225<br>(204 - 247)	-63.63%<br>(-66.39% - -60.55%)
Household air pollution from solid fuels									
Ambient ozone pollution	3<br>(1 - 4)	9<br>(7 - 13)	296.55%<br>(114.90% - 663.72%)	2<br>(1 - 3)	8<br>(5 - 10)	369.39%<br>(150.14% - 803.88%)	4<br>(2 - 7)	17<br>(12 - 23)	334.01%<br>(141.02% - 738.00%)
Other environmental risks	48<br>(26 - 79)	25<br>(14 - 40)	-44.04%<br>(-61.29% - -15.88%)	28<br>(14 - 48)	19<br>(10 - 31)	-27.07%<br>(-48.68% - 12.79%)	76<br>(41 - 125)	44<br>(24 - 71)	-38.23%<br>(-56.14% - -7.59%)
Residential radon	24<br>(13 - 38)	16<br>(9 - 27)	-24.78%<br>(-58.65% - 26.26%)	11<br>(6 - 17)	12<br>(6 - 19)	17.72%<br>(-36.24% - 95.83%)	34<br>(19 - 55)	28<br>(16 - 44)	-11.53%<br>(-51.09% - 49.22%)
Lead exposure	24<br>(7 - 53)	9<br>(2 - 21)	-65.02%<br>(-73.50% - -56.07%)	18<br>(6 - 37)	7<br>(2 - 17)	-61.43%<br>(-70.46% - -49.84%)	42<br>(13 - 90)	16<br>(4 - 38)	-63.77%<br>(-71.43% - -54.95%)
Occupational risks	233<br>(184 - 303)	270<br>(211 - 324)	32.80%<br>(-1.42% - 63.11%)	84<br>(64 - 108)	108<br>(83 - 136)	33.36%<br>(10.58% - 58.04%)	317<br>(253 - 401)	377<br>(299 - 455)	30.09%<br>(3.65% - 52.61%)
Occupational asthmagens	8<br>(6 - 11)	8<br>(5 - 11)	9.55%<br>(-5.47% - 25.57%)	4<br>(3 - 5)	4<br>(3 - 6)	21.91%<br>(1.10% - 42.79%)	12<br>(9 - 16)	12<br>(8 - 17)	10.61%<br>(0.59% - 22.52%)
Occupational particulate matter, gases, and fumes	17<br>(13 - 23)	15<br>(10 - 21)	-2.00%<br>(-12.39% - 8.18%)	3<br>(2 - 4)	4<br>(2 - 5)	13.74%<br>(-1.58% - 29.73%)	21<br>(15 - 27)	18<br>(13 - 25)	-2.22%<br>(-11.28% - 6.26%)
Occupational noise	12<br>(6 - 20)	12<br>(6 - 21)	14.46%<br>(-4.56% - 31.18%)	3<br>(1 - 5)	4<br>(2 - 8)	31.19%<br>(3.82% - 62.30%)	15<br>(8 - 26)	16<br>(8 - 28)	13.36%<br>(-2.67% - 26.93%)
Occupational injuries	46<br>(23 - 83)	50<br>(26 - 85)	46.04%<br>(-44.69% - 212.97%)	24<br>(12 - 42)	29<br>(15 - 46)	36.85%<br>(-41.93% - 166.20%)	71<br>(42 - 112)	79<br>(48 - 118)	33.50%<br>(-31.98% - 135.50%)
Occupational ergonomic factors	67<br>(45 - 94)	72<br>(49 - 100)	30.11%<br>(16.44% - 45.80%)	38<br>(26 - 54)	51<br>(34 - 72)	43.57%<br>(29.59% - 58.55%)	105<br>(72 - 148)	123<br>(83 - 171)	32.75%<br>(22.39% - 44.91%)
Occupational carcinogens	82<br>(64 - 134)	117<br>(72 - 142)	57.41%<br>(-23.89% - 100.23%)	14<br>(11 - 22)	22<br>(14 - 27)	63.02%<br>(-8.41% - 101.57%)	96<br>(77 - 151)	139<br>(90 - 165)	58.00%<br>(-17.57% - 98.08%)
Occupational exposure to asbestos	74<br>(55 - 127)	113<br>(67 - 137)	70.90%<br>(-23.33% - 121.17%)	11<br>(9 - 20)	19<br>(11 - 25)	77.85%<br>(-11.18% - 126.27%)	85<br>(65 - 141)	132<br>(83 - 158)	72.16%<br>(-16.78% - 120.57%)
Occupational exposure to trichloroethylene	0<br>(0 - 0)	0<br>(0 - 0)	5.04%<br>(-8.33% - 18.87%)	0<br>(0 - 0)	0<br>(0 - 0)	9.31%<br>(-6.72% - 26.89%)	0<br>(0 - 0)	0<br>(0 - 0)	3.95%<br>(-8.91% - 15.40%)
Occupational exposure to arsenic	0<br>(0 - 0)	0<br>(0 - 0)	-40.24%<br>(-48.83% - -32.28%)	0<br>(0 - 0)	0<br>(0 - 0)	1.44%<br>(-16.36% - 17.71%)	0<br>(0 - 0)	0<br>(0 - 0)	-34.77%<br>(-42.43% - -26.99%)
Occupational exposure to benzene	0<br>(0 - 0)	0<br>(0 - 0)	18.68%<br>(-0.97% - 31.80%)	0<br>(0 - 0)	0<br>(0 - 1)	9.41%<br>(-10.18% - 27.66%)	1<br>(1 - 1)	1<br>(1 - 1)	14.10%<br>(-0.29% - 26.86%)
Occupational exposure to beryllium	0<br>(0 - 0)	0<br>(0 - 0)	-28.09%<br>(-38.69% - -18.55%)	0<br>(0 - 0)	0<br>(0 - 0)	10.86%<br>(-3.54% - 23.48%)	0<br>(0 - 0)	0<br>(0 - 0)	-13.50%<br>(-23.06% - -5.03%)
Occupational exposure to cadmium	0<br>(0 - 0)	0<br>(0 - 0)	-38.31%<br>(-47.08% - -30.41%)	0<br>(0 - 0)	0<br>(0 - 0)	7.46%<br>(-9.73% - 21.84%)	0<br>(0 - 0)	0<br>(0 - 0)	-31.41%<br>(-39.49% - -23.54%)
Occupational exposure to chromium	0<br>(0 - 0)	0<br>(0 - 0)	-41.12%<br>(-49.17% - -34.06%)	0<br>(0 - 0)	0<br>(0 - 0)	9.33%<br>(-8.01% - 22.61%)	0<br>(0 - 0)	0<br>(0 - 0)	-32.13%<br>(-39.02% - -25.18%)
Occupational exposure to diesel engine exhaust	3<br>(3 - 4)	2<br>(1 - 2)	-38.65%<br>(-47.28% - -30.39%)	1<br>(1 - 1)	1<br>(0 - 1)	3.02%<br>(-15.61% - 19.89%)	4<br>(3 - 4)	2<br>(2 - 3)	-32.25%<br>(-39.71% - -24.66%)
Occupational exposure to second-hand smoke	3<br>(2 - 3)	2<br>(1 - 2)	-32.06%<br>(-40.79% - -23.90%)	1<br>(1 - 1)	1<br>(1 - 1)	22.89%<br>(4.30% - 36.60%)	4<br>(3 - 4)	3<br>(2 - 3)	-17.04%<br>(-24.50% - -9.11%)
Occupational exposure to formaldehyde	0<br>(0 - 0)	0<br>(0 - 0)	-6.55%<br>(-20.16% - 5.00%)	0<br>(0 - 0)	0<br>(0 - 0)	-5.36%<br>(-19.54% - 10.03%)	0<br>(0 - 0)	0<br>(0 - 0)	-8.04%<br>(-18.90% - 1.09%)
Occupational exposure to nickel	1<br>(1 - 1)	0<br>(0 - 1)	-43.43%<br>(-52.01% - -35.73%)	0<br>(0 - 0)	0<br>(0 - 0)	0.49%<br>(-17.23% - 15.75%)	1<br>(1 - 1)	1<br>(0 - 1)	-37.28%<br>(-45.40% - -29.23%)
Occupational exposure to polycyclic aromatic hydrocarbons	0<br>(0 - 1)	0<br>(0 - 0)	-36.71%<br>(-45.47% - -28.63%)	0<br>(0 - 0)	0<br>(0 - 0)	12.40%<br>(-5.28% - 27.35%)	1<br>(0 - 1)	0<br>(0 - 0)	-27.70%<br>(-35.59% - -19.41%)
Occupational exposure to silica	2<br>(2 - 2)	1<br>(1 - 1)	-32.23%<br>(-41.42% - -23.69%)	0<br>(0 - 0)	0<br>(0 - 0)	-6.14%<br>(-22.11% - 5.96%)	2<br>(2 - 2)	1<br>(1 - 2)	-30.90%<br>(-39.21% - -22.67%)

Occupational exposure to sulfuric acid	0 (0 - 1)	0 (0 - 0)	-33.21% (-43.49% - -20.94%)	0 (0 - 0)	0 (0 - 0)	-4.38% (-27.86% - 21.32%)	0 (0 - 1)	0 (0 - 0)	-31.83% (-41.79% - -19.76%)
Behavioral risks	3,350 (3,162 - 3,524)	2,248 (2,041 - 2,464)	-24.29% (-27.12% - -21.44%)	2,168 (2,013 - 2,322)	1,566 (1,400 - 1,739)	-21.33% (-24.45% - -17.81%)	5,517 (5,190 - 5,829)	3,814 (3,475 - 4,159)	-23.35% (-25.68% - -20.99%)
Child and maternal malnutrition	77 (55 - 108)	79 (54 - 114)	33.56% (25.12% - 42.03%)	76 (54 - 107)	75 (50 - 108)	13.44% (2.00% - 23.10%)	152 (108 - 215)	154 (104 - 224)	22.61% (14.46% - 30.25%)
Childhood underweight	0 (0 - 0)	0 (0 - 0)	-41.94% (-66.34% - -12.73%)	0 (0 - 0)	0 (0 - 0)	-58.97% (-76.12% - -32.93%)	0 (0 - 0)	0 (0 - 0)	-51.54% (-70.21% - -25.36%)
Suboptimal breastfeeding	8 (2 - 12)	3 (1 - 5)	-41.11% (-61.67% - -16.29%)	6 (2 - 10)	2 (1 - 4)	-51.27% (-68.67% - -28.45%)	14 (4 - 22)	6 (2 - 9)	-46.63% (-63.78% - -24.21%)
Discontinued breastfeeding	0 (0 - 0)	0 (0 - 0)	144.76% (-71.89% - 827.84%)	0 (0 - 0)	0 (0 - 0)	166.05% (-67.71% - 920.78%)	0 (0 - 0)	0 (0 - 0)	148.48% (-68.96% - 879.04%)
Non-exclusive breastfeeding	8 (2 - 12)	3 (1 - 5)	-41.11% (-61.67% - -16.29%)	6 (2 - 10)	2 (1 - 4)	-51.27% (-68.67% - -28.45%)	14 (4 - 22)	6 (2 - 9)	-46.63% (-63.78% - -24.21%)
Iron deficiency	69 (46 - 99)	76 (50 - 110)	43.54% (37.35% - 50.73%)	69 (47 - 100)	72 (48 - 105)	21.26% (8.96% - 30.16%)	138 (93 - 198)	148 (99 - 216)	31.42% (23.93% - 38.01%)
Vitamin A deficiency	0 (0 - 0)	0 (0 - 0)	-17.86% (-60.85% - 44.26%)	0 (0 - 0)	0 (0 - 0)	-17.05% (-63.32% - 57.63%)	0 (0 - 0)	0 (0 - 0)	-19.17% (-61.07% - 34.15%)
Zinc deficiency	0 (0 - 0)	0 (0 - 0)	-3.45% (-55.97% - 110.22%)	0 (0 - 0)	0 (0 - 0)	-1.94% (-60.94% - 136.34%)	0 (0 - 1)	0 (0 - 0)	-4.24% (-57.55% - 112.75%)
Tobacco smoke	1,524 (1,404 - 1,648)	847 (756 - 943)	-40.99% (-47.37% - -34.82%)	856 (766 - 949)	612 (523 - 689)	-26.79% (-35.85% - -17.67%)	2,380 (2,230 - 2,538)	1,459 (1,338 - 1,595)	-36.09% (-40.97% - -31.43%)
Smoking	1,480 (1,353 - 1,609)	835 (744 - 933)	-40.00% (-46.59% - -33.44%)	829 (734 - 925)	605 (517 - 682)	-25.27% (-34.80% - -15.47%)	2,309 (2,152 - 2,471)	1,440 (1,320 - 1,576)	-34.90% (-40.00% - -29.85%)
Secondhand smoke	44 (37 - 52)	11 (10 - 13)	-72.73% (-76.83% - -67.79%)	27 (23 - 32)	7 (6 - 8)	-73.43% (-77.67% - -68.05%)	71 (62 - 81)	18 (16 - 20)	-73.33% (-76.44% - -69.93%)
Alcohol and drug use	473 (400 - 550)	531 (455 - 609)	34.80% (21.75% - 45.42%)	301 (255 - 351)	257 (219 - 295)	-5.92% (-11.40% - -0.68%)	774 (658 - 894)	789 (681 - 894)	15.96% (8.02% - 22.71%)
Alcohol use	352 (293 - 413)	356 (297 - 418)	16.72% (7.24% - 25.14%)	250 (207 - 295)	197 (164 - 230)	-16.68% (-22.28% - -11.36%)	602 (505 - 701)	553 (464 - 641)	0.60% (-5.85% - 6.12%)
Drug use	126 (98 - 157)	194 (158 - 227)	95.71% (56.86% - 122.58%)	54 (41 - 67)	71 (58 - 85)	47.97% (34.42% - 63.98%)	180 (140 - 223)	265 (219 - 312)	74.64% (49.98% - 95.50%)
Dietary risks	1,636 (1,454 - 1,803)	904 (773 - 1,033)	-41.03% (-44.83% - -37.05%)	994 (832 - 1,137)	566 (459 - 695)	-39.63% (-45.44% - -30.35%)	2,630 (2,307 - 2,935)	1,470 (1,248 - 1,688)	-41.04% (-44.34% - -36.35%)
Diet low in fruits	427 (250 - 606)	231 (130 - 339)	-42.34% (-51.64% - -33.38%)	242 (132 - 356)	139 (70 - 218)	-40.13% (-54.15% - -24.24%)	669 (385 - 951)	370 (209 - 547)	-42.34% (-50.38% - -34.44%)
Diet low in vegetables	288 (226 - 357)	147 (114 - 183)	-45.35% (-50.64% - -39.52%)	194 (147 - 244)	104 (79 - 138)	-44.62% (-52.55% - -31.84%)	482 (380 - 594)	251 (198 - 309)	-45.63% (-50.10% - -40.34%)
Diet low in whole grains	247 (177 - 322)	132 (92 - 174)	-41.71% (-46.18% - -36.55%)	160 (110 - 208)	93 (63 - 128)	-36.91% (-44.35% - -25.81%)	407 (291 - 529)	225 (157 - 300)	-40.65% (-45.03% - -35.44%)
Diet low in nuts and seeds	333 (242 - 430)	146 (104 - 192)	-52.43% (-56.48% - -46.62%)	172 (123 - 227)	82 (56 - 116)	-49.55% (-56.87% - -33.80%)	505 (368 - 649)	228 (162 - 301)	-52.27% (-56.22% - -46.07%)
Diet low in milk	17 (5 - 30)	16 (4 - 29)	-1.58% (-14.58% - 11.36%)	14 (4 - 25)	11 (3 - 19)	-22.18% (-32.80% - -11.21%)	32 (9 - 54)	27 (7 - 47)	-10.65% (-19.63% - -1.20%)
Diet high in red meat	19 (10 - 27)	25 (15 - 37)	52.92% (19.15% - 94.04%)	20 (11 - 28)	23 (14 - 33)	27.53% (-3.01% - 68.37%)	38 (22 - 55)	48 (30 - 68)	39.62% (14.47% - 71.51%)
Diet high in processed meat	281 (177 - 401)	182 (103 - 270)	-27.64% (-58.06% - 11.76%)	125 (77 - 181)	81 (38 - 133)	-28.11% (-63.05% - 16.57%)	406 (266 - 573)	262 (157 - 385)	-29.38% (-53.54% - -0.31%)
Diet high in sugar-sweetened beverages	12 (5 - 21)	18 (9 - 30)	93.59% (4.25% - 262.52%)	11 (5 - 20)	15 (7 - 25)	50.68% (-25.49% - 191.97%)	23 (12 - 38)	33 (18 - 53)	66.88% (11.52% - 150.70%)
Diet low in fiber	259 (169 - 346)	133 (84 - 186)	-44.11% (-63.77% - -18.57%)	128 (82 - 174)	61 (34 - 93)	-49.19% (-70.68% - -23.53%)	386 (273 - 502)	194 (132 - 263)	-47.07% (-62.90% - -30.06%)
Diet suboptimal in calcium	30 (25 - 38)	33 (24 - 44)	10.57% (-20.69% - 32.59%)	12 (6 - 19)	10 (3 - 18)	-11.35% (-65.99% - 69.52%)	43 (35 - 50)	43 (32 - 54)	5.06% (-22.25% - 34.53%)
Diet low in seafood omega-3 fatty acids	106 (62 - 158)	48 (27 - 73)	-51.06% (-61.77% - -36.27%)	50 (27 - 78)	24 (13 - 39)	-51.38% (-63.62% - -28.08%)	157 (93 - 229)	72 (42 - 107)	-52.25% (-60.78% - -40.47%)
Diet low in polyunsaturated fatty acids	148 (117 - 178)	59 (45 - 75)	-56.94% (-63.14% - -49.43%)	75 (55 - 95)	30 (21 - 42)	-59.17% (-68.61% - -43.45%)	224 (177 - 269)	89 (70 - 111)	-58.33% (-64.27% - -51.28%)
Diet high in trans fatty acids	216 (142 - 292)	28 (8 - 54)	-86.37% (-94.69% - -77.24%)	101 (64 - 140)	13 (4 - 26)	-86.85% (-95.01% - -77.19%)	317 (215 - 422)	42 (14 - 78)	-86.83% (-94.01% - -78.85%)
Diet high in sodium	336 (136 - 605)	165 (66 - 302)	-48.12% (-55.13% - -41.82%)	164 (53 - 334)	86 (26 - 185)	-45.51% (-55.52% - -33.28%)	500 (192 - 941)	251 (92 - 488)	-47.64% (-54.35% - -41.07%)
Sexual abuse and violence	22 (12 - 35)	16 (9 - 25)	-10.74% (-24.72% - 16.62%)	84 (60 - 114)	65 (44 - 92)	-14.33% (-20.57% - -7.89%)	106 (76 - 143)	81 (56 - 112)	-10.91% (-16.91% - -2.57%)
Childhood sexual abuse	22 (12 - 35)	16 (9 - 25)	-10.74% (-24.72% - 16.62%)	25 (13 - 40)	19 (10 - 32)	-13.24% (-21.87% - -2.84%)	47 (27 - 74)	35 (20 - 56)	-12.16% (-20.13% - 3.79%)
Intimate Partner Violence	--	--	--	64 (47 - 89)	49 (34 - 71)	-16.34% (-23.14% - -9.46%)	64 (47 - 89)	49 (34 - 71)	-11.42% (-18.97% - -4.03%)
Unsafe sex	14 (11 - 17)	10 (7 - 14)	-8.43% (-29.05% - 14.67%)	48 (39 - 53)	31 (26 - 36)	-31.90% (-39.95% - -24.13%)	62 (53 - 70)	41 (35 - 49)	-25.17% (-33.94% - -16.42%)
Low physical activity	378 (319 - 451)	207 (174 - 244)	-41.94% (-46.48% - -36.85%)	305 (263 - 349)	188 (160 - 228)	-35.45% (-41.88% - -24.77%)	683 (588 - 797)	396 (340 - 461)	-39.57% (-44.24% - -33.74%)

Metabolic	2,225 (2,094 - 2,380)	1,392 (1,227 - 1,577)	-33.01% (-36.15% - -29.77%)	1,810 (1,609 - 1,984)	1,228 (1,049 - 1,427)	-26.08% (-30.64% - -19.03%)	4,035 (3,727 - 4,349)	2,621 (2,299 - 2,973)	-30.43% (-33.42% - -27.27%)
High fasting plasma glucose	417 (350 - 488)	340 (272 - 417)	-10.99% (-23.13% - 2.71%)	409 (335 - 490)	343 (273 - 424)	-8.41% (-21.31% - 5.26%)	826 (693 - 968)	683 (556 - 832)	-9.92% (-20.47% - 1.26%)
High total cholesterol	859 (616 - 1,110)	346 (258 - 466)	-56.96% (-60.40% - -51.86%)	367 (236 - 538)	155 (100 - 243)	-57.05% (-63.42% - -43.25%)	1,226 (863 - 1,644)	501 (362 - 684)	-57.62% (-61.23% - -51.39%)
High systolic blood pressure	1,429 (1,262 - 1,573)	597 (512 - 686)	-55.80% (-58.82% - -52.75%)	1,044 (890 - 1,166)	473 (398 - 570)	-51.11% (-55.60% - -43.70%)	2,474 (2,188 - 2,716)	1,070 (920 - 1,235)	-54.05% (-56.93% - -50.71%)
High body-mass index	778 (653 - 897)	703 (587 - 824)	-0.37% (-7.63% - 8.16%)	626 (521 - 725)	601 (494 - 714)	3.32% (-5.15% - 13.21%)	1,404 (1,188 - 1,610)	1,304 (1,090 - 1,525)	0.39% (-6.15% - 7.93%)
Low bone mineral density	104 (81 - 132)	90 (67 - 120)	-16.49% (-28.03% - -3.57%)	186 (145 - 230)	123 (93 - 160)	-32.34% (-43.12% - -21.04%)	290 (225 - 363)	214 (160 - 281)	-27.07% (-37.86% - -15.51%)
Low glomerular filtration rate	291 (219 - 372)	218 (163 - 276)	-18.37% (-26.12% - -10.91%)	315 (238 - 402)	249 (189 - 312)	-12.06% (-20.19% - -3.49%)	606 (461 - 767)	467 (354 - 584)	-15.12% (-22.80% - -7.82%)

Web Table 9: **GBD 2013 region-level covariates for the nine regions of England***
Education (years per capita)
Lag distributed income per capita (LDI)
Cholesterol (total, mean per capita)
Systolic blood pressure (mmHg)
Diabetes fasting plasma glucose (mmol/L)
Mean body mass index (BMI)
Prevalence of obesity
Prevalence of obesity (age-standardized)
Prevalence of overweight and obesity
Prevalence of smoking
Alcohol (liters per capita)
Diphtheria-tetanus-pertussis (DTP3) immunization coverage (proportion)
Tetanus Toxoid vaccine coverage smooth (proportion)
Measles vaccine coverage (proportion)
Antenatal care (1 visit) coverage (proportion)
Antenatal care (4 visits) coverage (proportion)
Hemoglobinopathies prevalence x excess mortality
Hemoglobinopathies prevalence x excess mortality (excluding G6PD deficiency)
Log age-standardized melanoma incidence
Age-standardized death rate for endocrine, metabolic, blood, and immune disorders
Age-standardized prevalence of chronic kidney disease (CKD) stage 3
Age-standardized prevalence of chronic kidney disease (CKD) stage 4
Seroprevalence of varicella
Animal fats (kcal per capita)
Fruits (kcal per capita)
Lognormal milk (kcal per capita)
Nuts and seeds (kcal per capita)
Percent of total calories consumed as animal fat
Percent of total calories consumed as fruits
Percent of total calories consumed as legumes
Percent of total calories consumed as milk
Percent of total calories consumed as nuts and seeds
Percent of total calories consumed as pig meat
Percent of total calories consumed as PUFA3
Percent of total calories consumed as PUFA6
Percent of total calories consumed as red meat
Percent of total calories consumed as red meat (without pig meat)
Percent of total calories consumed as saturated fat
Percent of total calories consumed as saturated and animal fats
Percent of total calories consumed as sugar
Percent of total calories consumed as vegetables
Percent of total calories consumed as non-wheat whole grains
Pig meat (kg per capita)
Polyunsaturated fatty acid (PUFA) omega 3 - seafood (kcal per capita)
Polyunsaturated fatty acid (PUFA) omega 6 - vegetable oils (kcal per capita)
Pulses and legumes (kcal per capita)
Red meat (kcal per capita)
Red meat without pig meat (kcal per capita)
Saturated fats (kcal per capita)
Saturated fats and animal fats (kcal per capita)
Stimulants (kcal per capita)
Sugar (kcal per capita)
Total calories (kcal per capita)
Vegetables (kcal per capita)
Whole grains (kcal per capita)
*The nine regions of England include: Greater London, West Midlands, East Midlands, South West England, South East England, North West England, North East England, East of England, Yorkshire and the Humber
