# Supplementary material for: Changes in health in England, with analysis by English regions and areas of deprivation, 1990–2013: a systematic analysis for the Global Burden of Disease Study 2013
Source: Lancet. 2015 Dec 5;386(10010):2257–74. doi: 10.1016/S0140-6736(15)00195-6 (PMC4672153; doi:10.1016/S0140-6736(15)00195-6)
Supplement: Supplementary Newton_PullOutFig2 [file mmc3.pdf]

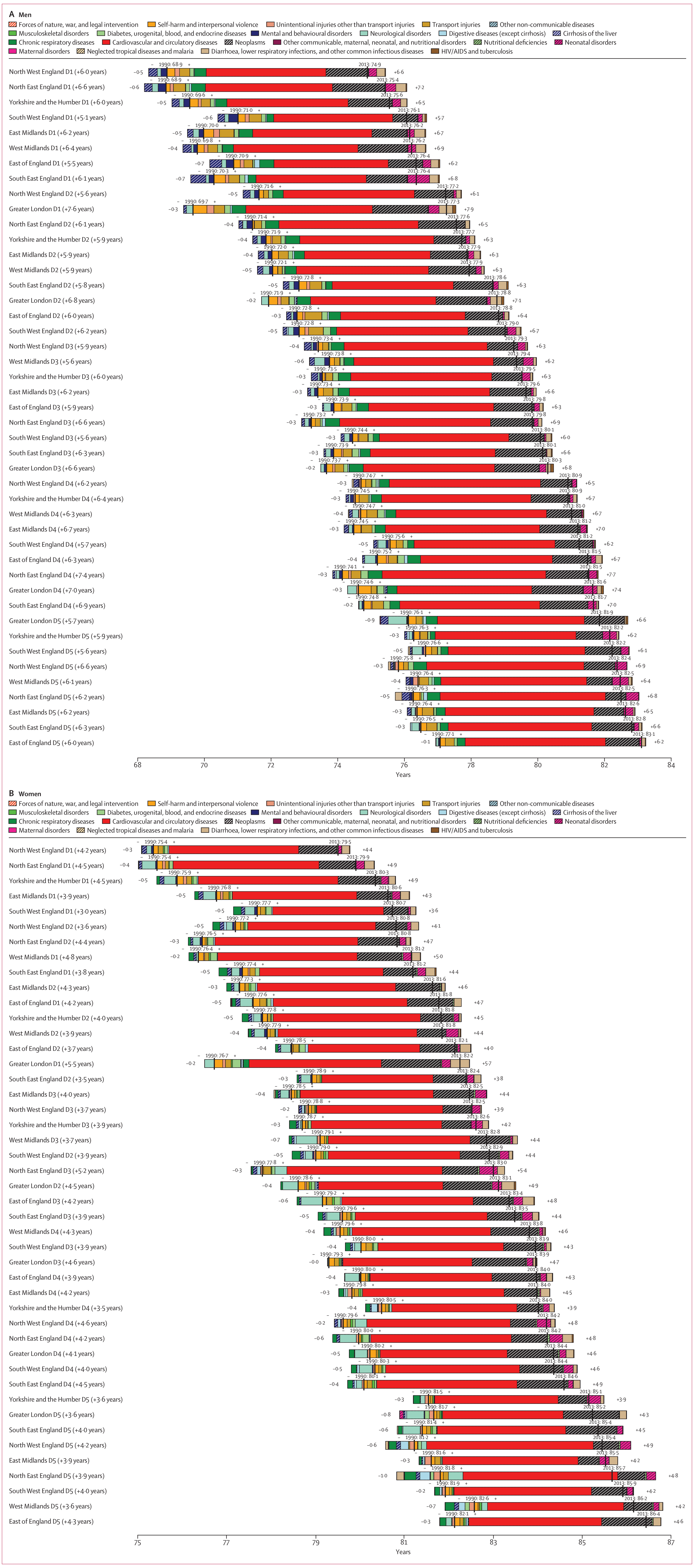

Figure appendix 2: **Change in life expectancy at birth for men (A) and women (B) from 1990 to 2013 in deprivation areas of English regions by broad cause group**
At the top of each bar in black, the life expectancy at birth in 1990 and in 2013 are noted. The number to the right of each bar associated with the life expectancy at birth in 1990 indicates the extension to life expectancy contributed by improvement in mortality rates from specific causes, whereas the number to the left of the same bar is the reduction to life expectancy at birth from increasing mortality rates from specific causes. The number beside each country name is the gain in life expectancy at birth from 1990 to 2013.
